# Synthesis and Biological Evaluation of Deoxycyclophellitols as Human Retaining β‐Glucosidase Inhibitors

**DOI:** 10.1002/chem.202402988

**Published:** 2024-11-03

**Authors:** Yevhenii Radchenko, Qin Su, Sybrin S. Schröder, Luke van Gijlswijk, Marta Artola, Johannes M. F. G. Aerts, Jeroen D. C. Codée, Herman S. Overkleeft

**Affiliations:** ^1^ Leiden Institute of Chemistry Leiden University, Einsteinweg 55 2333 CC Leiden The Netherlands

**Keywords:** Cyclophellitol, Glucosidase, Inhibitors and activity-based probes (ABPs), Deoxygenation, Carbasugar

## Abstract

Cyclophellitol is a potent and selective mechanism‐based retaining β‐glucosidase inhibitor that has served as a versatile starting point for the development of activity‐based glycosidase probes (ABPs). We developed routes of synthesis of eight mono‐ and dideoxycyclophellitols and cyclophellitol aziridines, the latter as ABPs carrying either a biotin or fluorophore linked to the aziridine nitrogen. We reveal the potency of these 24 compounds as inhibitors of the three human retaining β‐glucosidases, GBA1, GBA2 and GBA3. We show that 3,6‐dideoxy‐β‐*galacto*‐cyclophellitol aziridine selectively captures GBA3 over GBA1 and GBA2 in extracts of cells overexpressing both GBA2 and GBA3. We also identify a probe that selectively labels GBA1 and GBA2 over GBA3 at lower concentrations. In sum, the here‐presented studies reveal new chemistries to prepare chiral, substituted cyclitol epoxides and aziridines, add to the growing suite of cyclophellitols varying in configuration and substitution pattern, and yielded a reagent that may find use to investigate the physiological role and therapeutic relevance of the most elusive of the three retaining β‐glucosidases: GBA3.

## Graphical abstract

A series of deoxygenated cyclophellitol derivatives was synthesized and screened against the three human retaining exo‐β‐glucosidases, GBA1, GBA2, and GBA3. From this screen an activity‐based probe (ABP) was identified that, in a concentration‐dependent manner, selectively captures GBA3 over GBA1 and GBA2 in extracts of HEK293T cells overexpressing both GBA2 and GBA3.

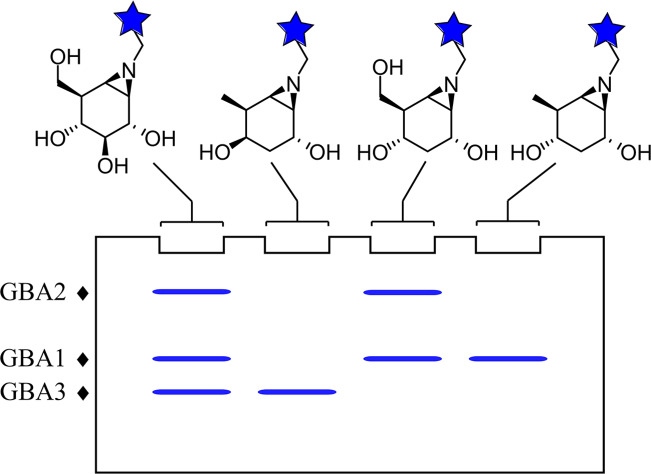

## Introduction

**Figure 1:**
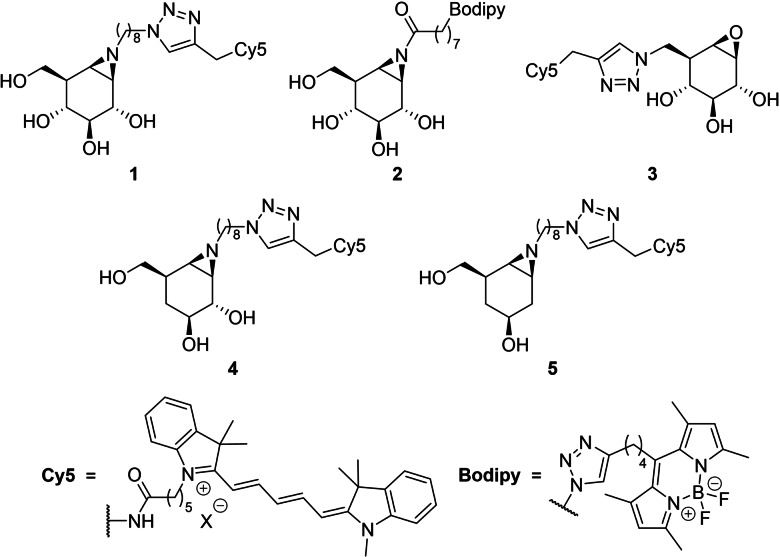
Structures of cyclophellitol‐based ABPs **1**–**5** reported previously.

Glycosidases, enzymes that catalyze the hydrolysis of oligosaccharides and glycoconjugates, are involved in a wide range of human pathologies and are therefore promising therapeutic targets. For instance, the three human exo‐β‐glucosidases, GBA1, GBA2 and GBA3 have been associated with Gaucher disease, neurodegenerative diseases, and cancer.[^1^, ^2^, ^3^] Cyclophellitol derivatives act as mechanism‐based covalent and irreversible retaining glycosidase inhibitors, by mimicking the transition state conformation of the substrate in the active site.^[4]^ Upon protonation by the active site general acid/base residue, the epoxide is opened by attack of the active site nucleophile resulting in the formation of a stable enzyme‐inhibitor ester adduct.^[5]^ Cyclophellitol derivatives functionalized with a reporter group are used for activity‐based protein profiling (ABPP) of retaining glycosidases.^[4]^ Cyclophellitol aziridines **1** and **2** (Figure 1) are broad‐spectrum retaining β‐glucosidase activity‐based probes (ABPs) that label GBA1, GBA2, and GBA3 at nanomolar concentrations.[^6^, ^7^] Altering configuration and/or substitution pattern of cyclophellitols may lead to unexpected activity and selectivity and thus support the investigation of new inhibitors and ABPs. For instance, conduritol‐configured *N*‐substituted aziridine^[8]^ and C6‐functionalized ABP **3** ^[9]^ selectively react with GBA1 over GBA2 and GBA3. As well, recently an *arabino*‐configured aziridine derivative has been revealed as a GBA2 selective ABP.^[10]^

In this context, we considered that deoxygenated cyclophellitol derivatives may have an altered activity and selectivity profile when compared to cyclophellitol. This hypothesis is supported by our previous findings on the altered reactivity of 2‐ and 4‐deoxygenated cyclophellitol ABPs (**4**–**5**, Figure 1).^[11]^ In the here‐presented work we report the synthesis of a series of 3‐ and 6‐deoxygenated derivatives, their inhibitory activity on GBA1, GBA2, and GBA3, and their use in activity‐based protein profiling. We also reveal a 3,6‐dideoxy‐β‐*galacto*‐cyclophellitol aziridine ABP to report with some selectivity on GBA3 in GBA2/GBA3‐overexpressing cells and put this reagent forward as a starting point for the development of selective probes to study endogenous GBA3 in samples also containing GBA1 and GBA2.

Figure 1 should be inserted here, as it relates to the introduction section

## Results and Discussion

**Scheme 1:**
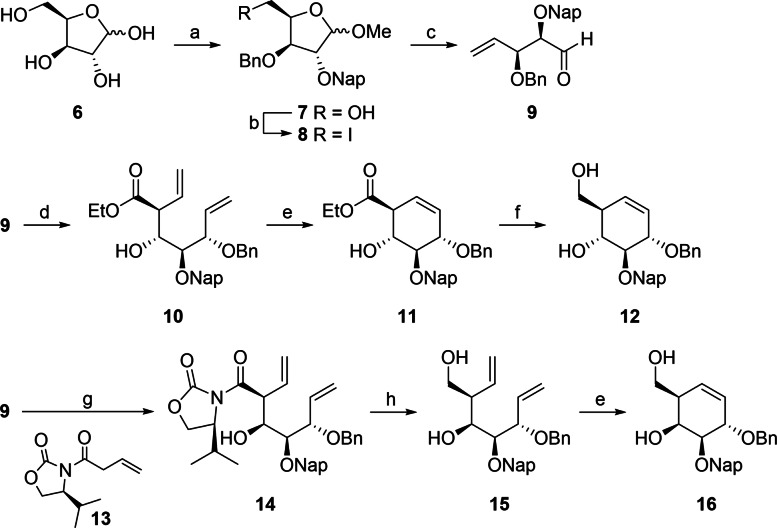
Reagents and conditions: (a) i. MeOH, AcCl; ii. PhCH(OCH_3_)_2_, CSA, MeCN; iii. NapBr, NaH, TBAI, DMF, −5 °C→r.t.; iv. pTSA, TFA, MeOH; v. TrCl, TEA, DMAP, DMF; vi. BnBr, NaH, TBAI, DMF, −5 °C→r.t.; vii. pTSA, TFA, DCM/MeOH, 41 % over 7 steps. (b) I_2_, PPh_3_, imidazole, THF, reflux, 95 %. (c) Zn dust, THF/H_2_O, sonication, 92 %. (d) Ethyl 4‐bromocrotonate, La(OTf)_3_, In powder, H_2_O, 28 %. (e) 2nd generation Grubbs catalyst, DCM, reflux, 96 % for **11**, 85 % for **16**. (f) DIBAL−H, 0 °C→r.t., NaBH_4_, THF, 84 %. (g) **13**, Bu_2_BOTf, TEA, −78 °C→−5 °C, H_2_O_2_, THF, 89 %. (h) LiBH_4_, THF, 0 °C, 88 %.

As the first objective, routes of synthesis to obtain key building blocks **12** and **16** were developed. For this, D‐xylose **6** was subjected to a multi‐step procedure resulting in an anomeric mixture of semi‐orthogonally protected methyl xylosides **7** without intermediate purification steps (Scheme 1). The latter was iodinated at the primary position under Garegg conditions^[12]^ to yield the mixture of anomers **8**. Although it has been reported that the reactivity of the two anomers is different under Vasella fragmentation conditions,^[13]^ this mixture was subjected to zinc treatment without prior separation, leading to the formation of **9** as the single product. Aldehyde **9** was used as a building block for the synthesis of both the *glucose*‐ and *galactose*‐configured cyclohexenes. Lanthanum triflate‐catalyzed Barbier reaction of **9** with ethyl 4‐bromocrotonate resulted in compound **10**. This reaction was previously reported by Madsen and co‐workers for the dibenzyl variety of aldehyde **9**,^[14]^ and the Bn‐for‐Nap‐substitution was envisioned to have a minor effect. The reaction of **9** however proved to be comparatively less stereoselective and separation of the two stereoisomers of **10** proved difficult. Pure fractions of **10** were subjected to ring‐closing metathesis (RCM) to give cyclohexene **11**, and ensuing ester reduction provided *glucose*‐configured cyclitol **12**. Impure **10** was submitted to the same transformations and the desired product could be isolated at this stage, yielding more of cyclitol **12**. *En route* to the galactose‐configured cyclitols, aldehyde **9** was submitted to aldol reaction with the Evans templated oxazolidinone **13**.^[15]^ This nucleophilic addition resulted in **14** as a single diastereomer, from which the Evans template was then removed reductively to give diol **15**, after which RCM yielded *galactose*‐configured cyclitol **16**.

**Scheme 2:**
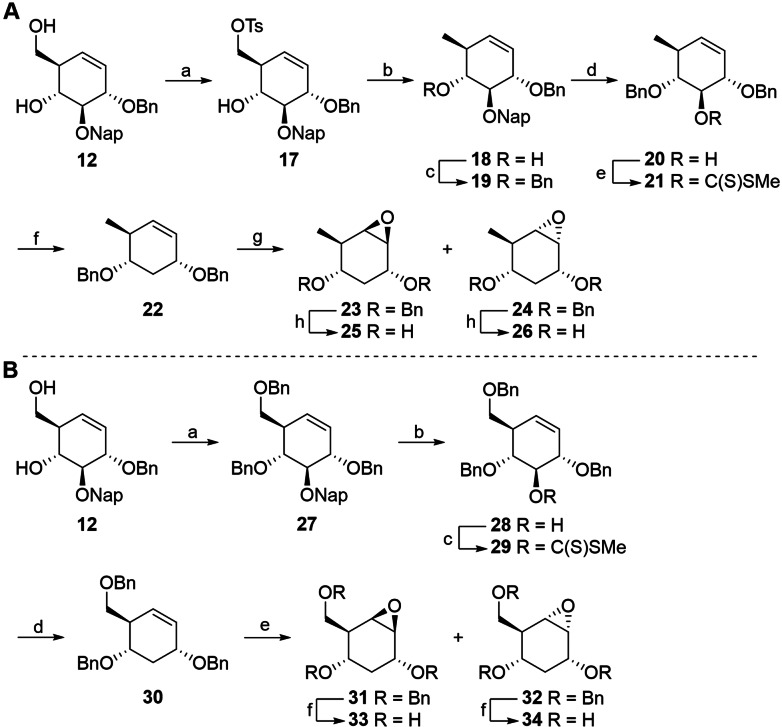
(A) Synthesis of *glucose*‐configured 3,6‐dideoxycyclophellitols. Reagents and conditions: (a) i. TsCl, TEA, DCM, 0 °C→r.t., 89 %. (b) LiAlH_4_, THF, 0 °C→r.t., 76 %. (c) BnBr, NaH, TBAI, DMF, 0 °C→r.t., 95 %. (d) DDQ, β‐pinene, DCM/H_2_O, 0 °C→r.t., 91 %. (e) *i*‐Pr_2_NH, *n*‐BuLi, **20**, CS_2_, MeI, THF, −78 °C →4 °C. (f) Bu_3_SnH, AIBN, toluene, reflux, 56 % over 2 steps. (g) *m*‐CPBA, DCM, **23**: 69 %, **24**: 19 %, or Oxone^®^, NaHCO_3_, Na_2_EDTA, AcCF_3_, MeCN, 0 °C, **23**: 71 %, **24**: 17 %. (h) Pd(OH)_2_/C, H_2_ 1 atm., 1,4‐dioxane, Milli‐Q, MeOH, 50 % for **25**, 52 % for **26**. (B) Synthesis of *glucose*‐configured 3‐deoxycyclophellitols. Reagents and conditions: (a) BnBr, NaH, TBAI, DMF, 0 °C→r.t., 98 %. (b) DDQ, DCM/H_2_O, 0 °C, 89 %. (c) CS_2_, LDA, MeI, THF, −78 °C →3 °C, 63 %. (d) Bu_3_SnH, AIBN, toluene, reflux, 89 %. (e) Oxone^®^, NaHCO_3_, Na_2_EDTA, AcCF_3_, MeCN, 0 °C, **31**: 39 %, **32**: 36 %. (f) Pd(OH)_2_/C, H_2_ 1 atm., 1,4‐dioxane, Milli‐Q, MeOH, 69 % for **33**, 76 % for **34**.

For the synthesis of 3,6‐dideoxy *glucose*‐configured cyclophellitols, compound **12** was selectively tosylated at the primary position (to give **17**) after which hydride substitution of the so‐formed leaving group resulted in 6‐deoxy derivative **18** (Scheme 2). The latter product was benzylated (**18** to **19**) and selectively denaphthylated to result in alcohol **20**. This was then subjected to the Barton‐McCombie deoxygenation protocol for which it was transformed to xanthate **21**, after which a radical deoxygenation gave the desired 3,6‐dideoxy cycloalkene **22**. Epoxidation of olefin **22** resulted in a mixture of stereoisomers **23** and **24**. The prevalence of the top‐side‐attack product **23** may be attributed to the steric hindrance of the alternative bottom‐side trajectory caused by the bulky benzyl substituents. The stereoisomers were separated by silica gel column chromatography and hydrogenated to give epoxides **25** and **26** respectively.

**Scheme 3:**
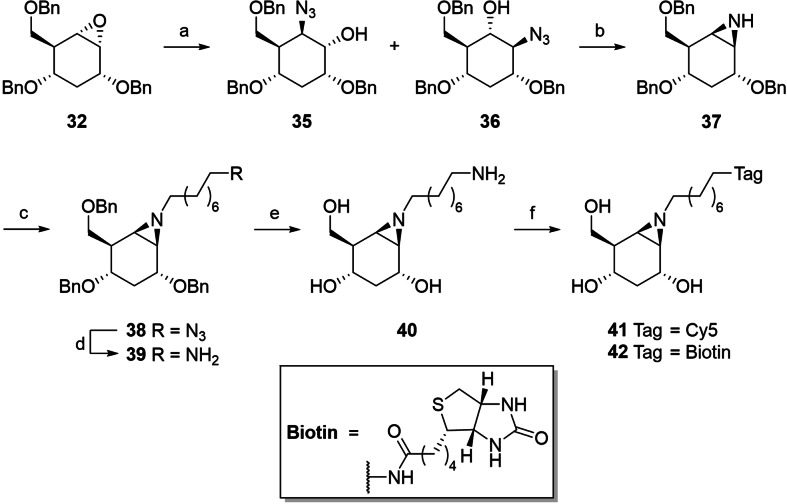
Synthesis of *glucose*‐configured 3‐deoxy‐β‐aziridines. Reagents and conditions: (a) NaN_3_, LiClO_4_, DMF, 95 °C, 88 % in total. (b) PPh_3_ (beads), MeCN, 60 °C, 91 %. (c) 8‐Azido‐1‐octanol, pyridine, Tf_2_O, DCM, **37**, DiPEA, 83 %. (d) PPh_3_ (beads), H_2_O, MeCN, 70 °C, quant. (e) Li, NH_3_(liq.), −70 °C→−55 °C, 58 %. (f) Cy5COOH, PFPOC(O)CF_3_, DiPEA, DMF, **40**, 32 % for **41**, or biotin‐OSu, DiPEA, 37 % for **42**.

To obtain the 3‐deoxy *glucose*‐configured derivatives, diol **12** was dibenzylated to produce **27**, followed by selective denaphthylation to result in alcohol **28**. The Barton‐McCombie deoxygenation protocol was applied, transforming the alcohol to xanthate (**28** to **29**), followed by reduction to result in 3‐deoxy derivative **30**. Epoxidation of the olefin and subsequent separation of the formed stereoisomers resulted in equal amounts of epoxides **31** and **32**, presumably due to the similar degree of shielding of both trajectories of attack. The thus obtained stereoisomers were hydrogenated to yield epoxides **33** and **34**, respectively. *Galactose*‐configured building block **16** was subjected to the same transformations as **12** to result in the corresponding 3‐deoxy and 3,6‐dideoxy *galactose*‐configured epoxides (Supporting information, Scheme S1). To produce the target aziridines, the epoxide ring in **32** was opened by nucleophilic azide substitution to give the mixture of azides **35** and **36** that was then subjected to reductive ring closure to give aziridine **37** (Scheme 3). The latter was then coupled with 8–azidooctanyltriflate to provide azide **38**, which was reduced to **39**. Amine **39** was deprotected using dissolving‐metal reduction conditions after which the product was desalted on a weak acidic cation exchange resin to result in compound **40**. This amine was then used in coupling reactions to install Cy5 and biotin reporter groups, resulting in compounds **41** and **42**, respectively.

The synthesis and subsequent functionalization of aziridines were then performed for other *glucose*‐ and *galactose*‐configured deoxycycloalkenes (Supporting information, Schemes S2–S8) which gave us access to the comprehensive series of 3‐deoxy and 3,6‐dideoxy derivatives as depicted in Figure 2.

**Figure 2:**
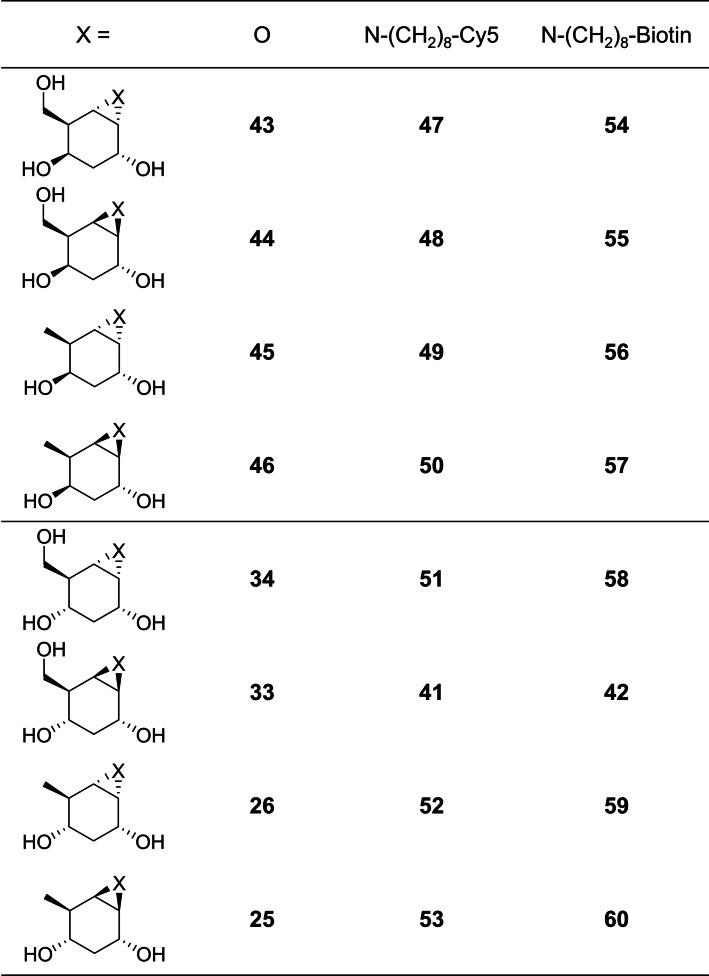
Overview all compounds subjected to the GBA1, GBA2, and GBA3 enzyme inhibition assays.

**Figure 3:**
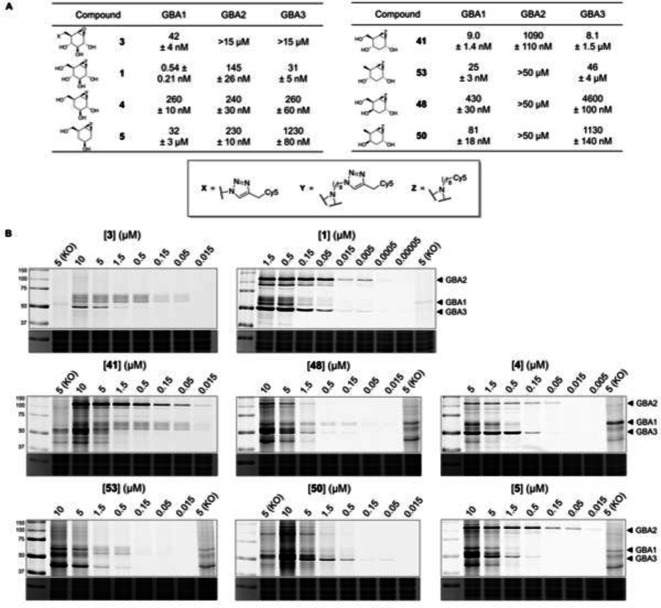
*In vitro* profiling of deoxygenated cyclophellitol aziridines. (A) IC_50_ values for inhibition of rhGBA1, GBA2 and/or GBA3 as measured in fluorogenic substrate assays (incubation time 30 min). (B) ABP labeling in lysates of GBA2/GBA3 overexpressing HEK293T cells at pH 6.0. KO: lysates of GBA1/GBA2 KO HEK293T cells were incubated with the specified ABP concentration. Lower panels: Coomassie brilliant blue (CBB) loading controls.

Next, all new compounds were screened on their GBA1/GBA2/GBA3 inhibitory potency in a fluorogenic substrate assay (enzyme‐mediated hydrolysis of the fluorogenic substrate, 4‐methylumbelliferyl β‐D‐glucopyranoside; 4‐MU‐β‐D‐Glu). For comparison, the previously reported cyclophellitols **4** and **5** as well as ABPs **1** and **3** were included in these assays. The IC_50_ values were determined after incubation of each individual compound with either recombinant human GBA1 (rhGBA1) or lysates of HEK293T cells overexpressing GBA2 or GBA3. In accordance with the literature data, ABP **1** inhibits all three enzymes at nanomolar concentrations, whereas ABP **3** proved selective for GBA1 (Figure 3A).[^6^, ^9^] All epoxides (IC_50_ >100 μM) and α‐configured aziridines (IC_50_ >50 μM, both ‐ 3 h incubation time) proved inactive in these assays. The inactivity of the α‐configured compounds is expected, since all three target enzymes act on β‐configured glucosides. The inactivity of the β–epoxides can be explained by the generally lower reactivity of cyclitol epoxides compared with their linker‐functionalized aziridine counterparts.^[6]^ All β‐configured aziridines demonstrated activity for one or more enzymes. The 4‐deoxy (**4**) and 2,4‐dideoxy (**5**) derivatives have drastically lower affinity for GBA1 when compared to ABP **1**, whereas inhibitory potency for GBA2 and GBA3 is less affected. This results in an altered selectivity pattern with a certain preference of 2,4‐dideoxy derivative **5** for GBA2 and GBA3, compared with the parent ‘all‐oxygenated’ ABP **1**. In contrast, 3,6‐dideoxy derivative **53** shows a high affinity for GBA1 and does not inhibit either GBA2 or GBA3. Another interesting observation is that *galactose*‐configured 3,6‐dideoxy derivative **50** has a higher affinity for both GBA1 and GBA3 than 3‐deoxy derivative **48** in this assay.

The fluorescent compounds that proved active in the fluorogenic substrate assay were tested as ABPs in HEK293T cell lysates that contain endogenous GBA1 and overexpressed human GBA2 and GBA3 (Figure 3B). As a negative control, lysates of HEK293T cells with knocked‐out (KO) expression of GBA1 and GBA2 were incubated with a high concentration of each ABP. The observed selectivity patterns of most compounds were in line with the results of the fluorogenic substrate assay except for **50**, which labeled GBA1 less potently than expected. The monodeoxy compounds **41**, **48**, and **4** label all three target glucosidases and thus demonstrate less difference compared with the selectivity profile of **1**. However, **41** labels GBA3 only at high concentrations and is selective for GBA1 and GBA2 at lower concentrations. In contrast, the dideoxy derivatives all discriminate between the target enzymes, labeling mainly one out of three (**53**: GBA1, **50**: GBA3, and **5**: GBA2 and, to a lesser extent, GBA3), alongside significant off‐target labeling at higher concentrations. For instance, besides labeling GBA2 and GBA3 (104.6 kDa and 53.6 kDa respectively), ABP **5** labels three major off‐target proteins (~45 kDa, ~55 kDa, and ~65 kDa), and these bands appear also in the experiments with the other deoxygenated ABPs (Figure 3B). Possibly, the deoxygenated ABPs are more hydrophobic compared to ABPs **1** and **3**, causing them to more readily associate with hydrophobic protein patches and subsequently engage in reactions.

**Figure 4:**
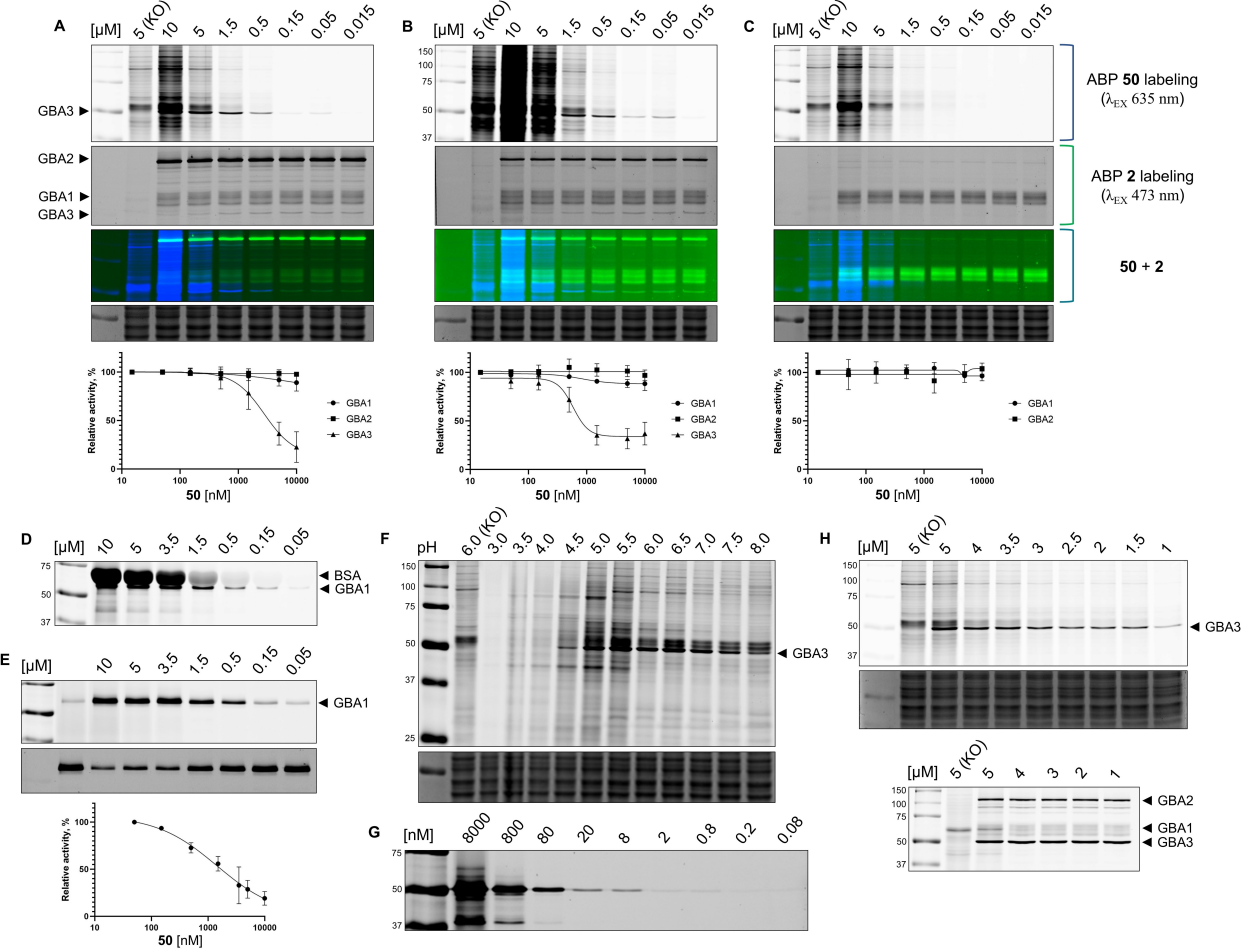
ABP labeling with **50** followed by cABPP with **2** (300 nM) in lysates of HEK293T GBA2/GBA3 overexpressing cells (incubation time (A) 30 min; (B) 3 h); (C) HEK293T WT cells (incubation time 30 min). (D) ABP labeling with **50** in rhGBA1 at pH 6.0 supplemented with 0.1 % (w/v) BSA. (E) cABPP. The sample for lane 2 was pre‐incubated with **2** (300 nM) followed by **50** (10 μM). Samples for lanes 3–9 were incubated with **50** followed by **2** (300 nM). (F) Effect of pH value (3.0–8.0) on labeling with **50** (3.5 μM) in lysates of HEK293T GBA2/GBA3 overexpressing cells. (G) Detection limit. rhGBA3 (2 pmol) was incubated with a decreasing amount of **50**. (H) Comparison of labeling selectivity with **50** (top) and **1** (bottom) in lysates of HEK293T GBA2/GBA3 overexpressing cells at varying ABP concentrations (1–5 μM). KO ‐ lysates of GBA1/GBA2 KO HEK293T cells were incubated the specified ABP concentration. Lower panels: Coomassie brilliant blue (CBB) loading controls.

Some selected compounds were next assessed by competitive ABPP (cABPP) with Bodipy‐tagged compound **2**, that allows read‐out and quantification independent of Cy5 excitation and emission ranges. The cABPP experiments confirmed the apparent selectivity of **5** for GBA2/3 over GBA1, while compound **53** was confirmed to target GBA1 selectively over GBA2 and GBA3 (Supporting information, Figure S1). Special attention was given to compound **50** to investigate the discrepancy in results on GBA1 from the fluorogenic substrate assays and ABPP assays. Competitive ABPP with **2** confirmed that GBA1 was not inhibited after incubation of cell lysates of GBA2/GBA3 overexpressing HEK293T cells with **50** for 30 min, nor for 3 h (Figure 4A and B). It was hypothesized that GBA1 was not inhibited due to the competition with unnaturally high levels of GBA2 and GBA3, however after incubation of **50** with wild‐type HEK293T cell lysates, GBA1 activity remained unchanged independent of compound concentration (Figure 4C).

Next, ABPP with **50** was performed on recombinant human GBA1. In contrast to the cell lysate ABPP, **50** labeled GBA1 in these conditions at various pH values (Supporting Information, Figure S2). It was hypothesized that binding of **50** to rhGBA1 is non‐specific, and to investigate this possibility, an ABP labeling on rhGBA1 supplemented with an excess of bovine serum albumin (BSA, 0.1 % w/v) was carried out. Despite the intensive labeling of BSA, effective labeling of rhGBA1 by **50** was retained (Figure 4D). Pre‐incubation of rhGBA1 with **2** outcompeted labeling by **50**, and incubation with **50** outcompeted labeling by **2**, which suggests the same binding site for these two ABPs (Figure 4E). Intriguingly, ABP **48** (the structurally closed analogue of **50**) labeled GBA1 in HEK293T cell lysates, in line with the results of the fluorogenic substrate assay (Supporting Information, Figure S2).

The optimal conditions for selective GBA3 labeling with **50** were investigated next. The labeling in lysates of GBA2/GBA3 overexpressing HEK293T cells was performed at a range of pH values from which pH 6.0 was identified to provide optimal contrast between the GBA3 band and the off‐target bands (Figure 4F). The sensitivity of detection and labeling was further demonstrated by incubating 2 pmol of recombinant human GBA3 with **50** at decreasing concentrations (Figure 4G). As illustrated in Figure 4H, the concentration range of 1.5 to 3.5 μM of **50** is suitable for selective labeling of GBA3 in these cell lysates. In comparison, the broad‐spectrum ABP **1** modifies all three GBAs at these concentrations. Finally, cABPP of **50** with potent β‐galactosidase and β‐glucosidase inhibitors indicated that the labeled off‐target proteins are not from these enzyme families (Supporting Information, Figure S3).

## Conclusions

We have developed a synthetic strategy towards 3‐deoxy and 3,6‐dideoxy cyclophellitol derivatives and obtained a series of 24 compounds that, along with previously reported deoxy derivatives **4** and **5**, were screened for activity and selectivity against the three human exo‐β‐glucosidases, GBA1, GBA2, and GBA3. The reactivity of compounds for each enzyme was accessed *in vitro* in fluorogenic substrate assay and ABPP. Despite the overlap in enzymatic activities of the target enzymes, the assays revealed candidate compounds with potentially advantageous selectivity profiles. In the biological system tested, ABP **50** can be used for selective labeling of GBA3 over GBA1/GBA2, whereas **41** selectively labels GBA1/GBA2 over GBA3 at lower concentrations. These complementing ABPs may prove useful for measuring GBA3 activity in more complex biological samples and thus contribute to the investigation of the physiological role and therapeutic relevance of GBA3. Compound **53** selectively labels GBA1 among the three tested enzymes, whereas **5** labels GBA2/GBA3 and not GBA1. These properties may be useful to facilitate the research on the role of GBA2 and GBA3 in Gaucher disease and other disorders.

## Conflict of Interests

The authors declare no conflict of interest.

1

## Supporting information

As a service to our authors and readers, this journal provides supporting information supplied by the authors. Such materials are peer reviewed and may be re‐organized for online delivery, but are not copy‐edited or typeset. Technical support issues arising from supporting information (other than missing files) should be addressed to the authors.

Supporting Information

## Data Availability

The data that support the findings of this study are available in the supplementary material of this article.
