# Supplementary material for: Synthesis and Biological Evaluation of Deoxycyclophellitols as Human Retaining β‐Glucosidase Inhibitors
Source: Chemistry. 2024 Nov 3;30(70):e202402988. doi: 10.1002/chem.202402988 (PMC11639629; doi:10.1002/chem.202402988)

Chemistry–A European Journal

Supporting Information

Synthesis and Biological Evaluation of Deoxycyclophellitols as Human Retaining β-Glucosidase Inhibitors

Yevhenii Radchenko, Qin Su, Sybrin S. Schröder, Luke van Gijlswijk, Marta Artola, Johannes M. F. G. Aerts, Jeroen D. C. Codée, and Herman S. Overkleeft*

Table of contents

Synthesis experimental procedures 2

Biochemical experiments 30

IC_{50} values determination graphs 32

NMR spectra 33

Synthesis experimental procedures

General experimental details

All reagents were purchased from certified commercial suppliers and used as received. Ethyl acetate (EtOAc) and acetone (Ac) were purified by distillation before use. Other solvents used for work-up and purification were of technical grade and used as received. Acetonitrile (MeCN), tetrahydrofuran (THF), methanol (MeOH), dimethylformamide (DMF), toluene and dichloromethane (DCM) were of reagent grade and were dried and stored over activated molecular sieves before use. All reactions were performed at room temperature in the atmosphere of nitrogen unless indicated otherwise. All starting materials in reactions that require anhydrous conditions were co-evaporated with toluene trice to remove traces of water. When possible, all reactions were monitored by TLC analysis using aluminum TLC plates of silica gel coated with fluorescent indicator F254, with detection by UV absorption and subsequent visualization by spraying with a solution of $(NH_4)_6Mo_7O_{24}\cdot 4H_2O$ (25 g/L) and $(NH_4)_4Ce(SO_4)_4\cdot 2H_2O$ (10 g/L) in 10% sulfuric acid or a solution of $KMnO_4$ (20 g/L) and K_2CO_3 (10 g/L) in water, followed by charring at 150 °C. Silica gel column chromatography was performed using Macherey-Nagel Silica 60 M silica gel (particle size of 40 – 63 µm) with the indicated eluents. LC/MS analysis was performed on a Surveyor HPLC (Thermo Finnigan) system coupled to a LCQ Advantage Max (Thermo Finnigan) system equipped with a C18 column (Gemini C18, 50 mm x 4.6 mm, 5 µm particle size, Phenomenex) or Vanquish UHPLC (Thermo Scientific) system coupled to a LCQ Fleet (Thermo Scientific) system equipped with a C18 column (NUCLEODUR C18 Gravity, 50 mm x 4.6 mm, 3 µm particle size, Macherey Nagel). For reversed phase HPLC purifications an Agilent Technologies 1260 Infinity II system coupled to an Agilent InfinityLab LC/MSD XT system equipped with a semi-preparative column (Gemini C18, 250 x 10 mm, 5 µm particle size, Phenomenex) was used. The applied buffers were (A) 50 mM NH_4HCO_3/MilliQ and (B) MeCN. ^1H NMR and ^{13}C NMR spectra were recorded on a Bruker AV-400 (400 and 101 MHz respectively), a Bruker AV-500 (500 and 126 MHz respectively), a Bruker DMX-600 (500 and 151 MHz respectively) or a Bruker-850 (850 and 214 MHz respectively) spectrometer in the given solvent. Chemical shifts are reported in ppm (δ) relative to the residual solvent peak or tetramethylsilane (0 ppm) as internal standard. ^{13}C NMR spectra are proton decoupled. High-resolution mass spectrometry (HRMS) analysis was performed with Q-Exactive HF Orbitrap (Thermo Scientific) equipped with an electrospray ion source by injection of 2 µl of a 1 µM solution via Ultimate 3000 nano UPLC (Dionex) system, with an external calibration (Thermo Scientific). Source voltage of 3.5 kV, capillary temperature 275 °C, no sheath gas. Resolution = 240.000 at m/z = 400. Mass range m/z = 160 - 2000 or up to a maximum of 6000. Eluent used: MeCN:H_2O (1:1 v/v) supplemented with 0.1% formic acid.

General procedure A. Epoxidation

An alkene (1 eq.) was dissolved in DCM (10 mL/mmol), then *meta*-chloroperoxybenzoic acid (freshly purified by extraction with $NaHCO_3$ (aq., sat), 2 eq.) was added and the mixture was stirred at room temperature until TLC analysis indicated full conversion of starting material (6.5-18 hours). The reaction mixture was diluted with DCM, washed with $NaHSO_3$ (aq., 10% w/v), $NaHCO_3$ (aq., sat.), brine, then dried over anhydrous $MgSO_4$ and filtered, after which the filtrate was concentrated and the product purified by silica gel column chromatography to afford isomeric epoxides.

General procedure B. Epoxidation

An alkene (1 eq.) was dissolved in MeCN (8 mL/mmol) and ethylenedinitrilotetraacetic acid disodium salt (1 mL/mmol, aq., 0.4 mM) was added. The reaction mixture was cooled down to 0 °C, and a ground-together mixture of Oxone® (5 eq.) and $NaHCO_3$ (7 eq.) was added in 5 equal portions over 1 hour. After the first portion, 1,1,1-trifluoroacetone (15 eq.) was quickly added with a pre-cooled syringe to avoid evaporation. Following the addition of the last portion, the reaction mixture was stirred for 1 hour at 0 °C. When at this stage TLC analysis indicated the presence of starting material, an additional amount of 1,1,1-trifluoroacetone (15 eq.) was added and the mixture was stirred for 1 hour. When TLC analysis indicated full conversion of starting material, the reaction mixture was diluted with EtOAc, washed with water, and the combined aqueous layers extracted with EtOAc. The combined organic layers were washed with brine, then dried over anhydrous $MgSO_4$ and filtered, the filtrate concentrated and the product purified by silica gel column chromatography.

General procedure C. Reductive debenzylation by hydrogenation

A protected epoxide (1 eq.) was dissolved in 2 mL of 1,4-dioxane, 1 mL of Milli-Q, and 0.5 mL of MeOH. Then palladium hydroxide on carbon (0.25 eq.) was added and the reaction mixture was purged with argon, charged with hydrogen gas and stirred until TLC analysis indicated full conversion. The reaction mixture was purged with argon and filtered, after which the filtrate was concentrated and the product purified by silica gel column chromatography.

General procedure D. Azidolysis of epoxides

An epoxide (1 eq.) was dissolved in DMF (12 mL/mmol), then sodium azide (5 eq.) and lithium perchlorate (10 eq.) were added, and the mixture was stirred at 95 °C for 16 hours. The reaction mixture was analyzed with TLC and if the presence of the starting material was detected (when indicated), additional amounts of sodium azide (5 eq.) and lithium perchlorate (10 eq.) were added, and the mixture was stirred for the indicated time. Then the reaction mixture was diluted with Et_2O, washed with water and brine, then dried over anhydrous $MgSO_4$ and filtered, after which the filtrate was concentrated and the product purified by silica gel column chromatography.

General procedure E. Aziridine formation

A mixture of azides (1 eq.) was dissolved in MeCN (18 mL/mmol) and polymer-bound triphenylphosphine (3 eq, 3 mmol/g) was added, and the mixture was stirred at 60 °C until TLC indicated the full conversion of starting material (18-40 hours). Then the reaction mixture was filtered, the filtrate concentrated and the product purified by silica gel column chromatography.

General procedure F. Linker installation

8-Azido-1-octanol (3 eq.) was dissolved in DCM (8 mL/mmol), pyridine (3.6 eq.) was added and the mixture was cooled to -50 °C. Trifluoromethanesulfonic anhydride solution in DCM (4 eq, 1 M) was added dropwise and the reaction mixture was allowed to warm up to -20 °C over 50 minutes, then diluted with DCM, washed trice with ice-cold water, dried over anhydrous $MgSO_4$ and concentrated to result in a yellow oil, which was co-evaporated with toluene before further use.

Aziridine (1 eq.) was combined with DiPEA (3.6 eq.) and a solution of the activated linker in DCM (20 mL/mmol of the aziridine) was added. The mixture was stirred overnight, then diluted with EtOAc, washed with $NaHCO_3$ (aq., 0.3 M), brine, then dried over anhydrous $MgSO_4$ and filtered, after which the filtrate was concentrated, and the product purified by silica gel column chromatography.

General procedure G. Staudinger azide reduction

An azide (1 eq.) was dissolved in MeCN (30 mL/mmol), then polymer-bound triphenylphosphine (2.3 eq, 3 mmol/g) and water (10 eq.) were added, and the mixture was stirred at 70 °C for 20 hours, followed by addition of water (3 mL/mmol of starting material) and stirring at 70 °C for 4 hours. The thus obtained reaction mixture was filtered and the filtrate was concentrated and the product used without further purification.

General procedure H. Dissolving-metal reductive debenzylation

Lithium (30 eq.) was added to a flask with condensed ammonia (7 mL/mmol of Li) at -60 °C and the resulting blue reaction mixture was stirred at this temperature for 15 minutes. A benzylated starting material (1 eq.) in THF (20 mL/mmol) was added dropwise to the reaction mixture at -70 °C, and the mixture was allowed to warm up to -55 °C over 40 minutes. If the reaction mixture discolored prematurely, an additional amount of lithium (15 eq.) was added. After 40 minutes of stirring, the mixture was quenched by portion wise addition of Milli-Q and concentrated, then re-dissolved in a small amount of Milli-Q and eluted over a column of Amberlite® CG-50 ion-exchange resin (NH_4^+ form) with 0.1 M → 0.5 M NH_4OH/Milli-Q.

General procedure I. Introduction of Cy5

Cy5COOH (1.35 eq.) was lyophilized in an Eppendorf tube and dissolved in DMF (20 mL/mmol), then DiPEA (5.4 eq.) and pentafluorophenyl trifluoroacetate (2.7 eq.) were added and the mixture was stirred for 2 hours, followed by addition of water (2 eq.) and stirring for 45 minutes. The thus obtained reaction mixture was transferred to an Eppendorf tube containing a starting amine (1 eq.) and DiPEA (3 eq.). The reaction mixture was stirred for 6 hours before being concentrated and eluted over a silica gel column (DCM/Ac/MeOH + TEA, 16:8:1 + 1% TEA → 2:1:1 + 4% TEA). Fractions containing the product mass (monitored by TLC/MS) were purified by reverse-phase HPLC (linear gradient of (A) 50 mM NH_4HCO_3/Milli-Q and (B) MeCN.

General procedure J. Introduction of biotin

A debenzylated amine (1 eq.) was lyophilized in an Eppendorf tube and dissolved in DMF (33 mL/mmol), then biotin-OSu (1.5 eq.) and DiPEA (3 eq.) were added, and the mixture was stirred for 24 hours. The reaction mixture was diluted with Milli-Q and lyophilized followed by reverse-phase HPLC purification (linear gradient of (A) 50 mM NH_4HCO_3/Milli-Q and (B) MeCN).

Compound **7**

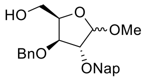

D-Xylose **6** (72.1 g, 480 mmol) was dispersed in 1000 mL of MeOH, acetyl chloride (22.2 mL, 312 mmol) was added, and the mixture was stirred for 7 hours, then neutralized with $NaHCO_3$ and filtered. The concentrated filtrate was re-dispersed in 1200 mL of EtOAc/MeOH (5:1), filtered through a glass filter and concentrated. The thus obtained yellow oil was dissolved in 700 mL of MeCN, and 10-camphorsulfonic acid (5.57 g, 23.98 mmol)

and benzaldehyde dimethyl acetal (83 mL, 552 mmol) were added. The resulting reaction mixture was stirred for 24 hours, then TEA (4.0 mL, 28.80 mmol) was added, the mixture was concentrated, re-dissolved in DCM, washed with water, brine, then dried over anhydrous $MgSO_4$ and filtered, after which the filtrate was concentrated. The thus obtained white amorphous mass was re-dispersed in 1000 mL of pentane/EtOAc (3:1) and filtered. The filtrate was concentrated, re-dissolved in 1500 mL of DMF, 2-(bromomethyl)naphthalene (103 g, 466 mmol) and tetrabutylammonium iodide (4.92 g, 13.32 mmol) were added, and the reaction mixture was cooled down to -5 °C on an ice-salt bath. Sodium hydride (60% dispersion in mineral oil, 23.1 g, 577 mmol) was added portion wise and the mixture was allowed to warm up to room temperature and stirred for 24 hours, then quenched with water, concentrated and re-dissolved in EtOAc, washed with water, brine, then dried over anhydrous $MgSO_4$ and filtered, after which the filtrate was concentrated. The thus obtained concentrate was re-dispersed in 1500 mL of MeOH, *p*-toluenesulfonic acid (19.40 g, 102 mmol) was added and the mixture was stirred for 20 hours, after which ^{1}H NMR analysis of an aliquot indicated low conversion of starting material. Trifluoroacetic acid (1.7 mL, 22.2 mmol) was added, and the mixture was stirred for 20 hours, then TEA (18.5 mL, 133 mmol) was added, the reaction mixture was concentrated, re-dissolved in Et_2O, washed with HCl (aq., 1 M), $NaHCO_3$ (aq., sat.), water, and brine, then dried over anhydrous $MgSO_4$ and filtered, after which the filtrate was concentrated. The thus obtained brown oil was re-dissolved in 1000 mL of DMF, trityl chloride (110 g, 394 mmol), TEA (59.5 mL, 427 mmol) and 4-dimethylaminopyridine (2.01 g, 16.43 mmol) were added. After 20 hours of stirring, trityl chloride (26 g, 93.1 mmol) was added and the reaction mixture was stirred for 48 hours, then concentrated, re-dissolved in Et_2O, washed with water, brine, then dried over anhydrous $MgSO_4$ and filtered, after which the filtrate was concentrated. The thus obtained compound was re-dissolved in 1000 mL of DMF, benzyl bromide (47.7 mL, 402 mmol) and tetrabutylammonium iodide (4.05 g, 10.98 mmol) were added and the mixture was cooled down to -5 °C. Sodium hydride (60% dispersion in mineral oil, 20.5 g, 512 mmol) was added portion wise and the mixture was allowed to warm up to room temperature and stirred for 15 hours. After that time, it was quenched with water, concentrated and re-dissolved in Et_2O, washed with water, brine, then dried over anhydrous $MgSO_4$ and filtered, after which the filtrate was concentrated. The concentrate was dissolved in 800 mL of DCM/MeOH (1:1), then *p*-toluenesulfonic acid (22.1 g, 116 mmol) was added and the mixture was stirred for 20 hours, after which an ^{1}H NMR analysis of an aliquot indicated a presence of starting material. Trifluoroacetic acid (2.0 mL, 29.8 mmol) was added, and the mixture was stirred for 72 hours. After this time, the reaction mixture was quenched with $NaHCO_3$ (aq., sat.), the layers were separated, and the aqueous layer was thoroughly extracted with EtOAc. The combined organic layers were washed with brine, then dried over anhydrous $MgSO_4$ and filtered, after which the filtrate was concentrated. Purification with silica gel column chromatography (pentane/EtOAc, 10:1 → 3:1) afforded partially separated anomers **7** (78 g, 41% over 7 steps). **α-anomer**: ^{1}H NMR (500 MHz, $CDCl_3$) δ 7.89 – 7.77 (m, 4H), 7.56 – 7.45 (m, 3H), 7.42 – 7.25 (m, 5H), 4.88 – 4.79 (m, 2H), 4.79 – 4.68 (m, 2H), 4.62 (d, *J* = 11.8 Hz, 1H), 4.49 (dd, *J* = 7.7, 6.4 Hz, 1H), 4.25 (dt, *J* = 7.8, 4.0 Hz, 1H), 4.13 (dd, *J* = 6.5, 4.2 Hz, 1H), 3.79 (m, 2H), 3.41 (s, 3H), 2.46 (s, 1H). ^{13}C NMR (126 MHz, $CDCl_3$) δ 137.61, 135.00, 133.24, 133.16, 128.57 (2C), 128.31, 127.97, 127.96, 127.75 (3C), 127.10, 126.29, 126.15, 126.07, 100.21, 84.59, 82.24, 76.32, 72.80 (2C), 62.27, 55.14. HRMS m/z: $[M+Na]^+$ calculated for $C_{24}H_{26}O_5$ 417.1673, found 417.1669. **β-anomer**: ^{1}H NMR (500 MHz, $CDCl_3$) δ 7.90 – 7.82 (m, 3H), 7.80 (dd, *J* = 1.9, 1.0 Hz, 1H), 7.56 – 7.47 (m, 2H), 7.48 (dd, *J* = 8.4, 1.7 Hz, 1H), 7.39 – 7.24 (m, 5H), 4.97 (d, *J* = 1.9 Hz, 1H), 4.77 (dd, *J* = 12.0, 0.9 Hz, 1H), 4.71 (dd, *J* = 11.9, 0.8 Hz, 1H), 4.66 (d, *J* = 12.1 Hz, 1H), 4.52 (d, *J* = 12.0 Hz, 1H), 4.37 (dt, *J* = 6.9, 4.8 Hz, 1H), 4.25 (dd, *J* = 6.8, 3.9 Hz, 1H), 4.19 (dd, *J* = 3.9, 1.9 Hz, 1H), 3.84 (m, 2H), 3.43 (s, 3H), 2.64 (d, *J* = 6.2 Hz, 1H). ^{13}C NMR (126 MHz, $CDCl_3$) δ 137.50, 134.96, 133.28, 133.12, 128.58 (2C), 128.36, 128.03, 127.96, 127.84 (2C), 127.78, 126.78, 126.32, 126.14, 125.78, 108.05, 87.26, 82.87, 80.67, 72.51, 72.41, 62.33, 55.73. HRMS m/z: $[M+Na]^+$ calculated for $C_{24}H_{26}O_5$ 417.1673, found 417.1670.

Compound **8**

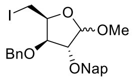

The mixture of isomers **7** (21.0 g, 53.2 mmol) was dissolved in 400 mL of THF, then triphenylphosphine (21.0 g, 80 mmol) and imidazole (7.25 g, 106 mmol) were added, and the reaction mixture was heated to 50 °C. Iodine (20.3 g, 80 mmol) was added portion wise as a solution in 150 mL of THF and the reaction mixture was refluxed for 20 hours, after which TLC analysis indicated full conversion of starting material. The mixture was concentrated, re-dissolved in EtOAc, washed with HCl (aq., 3 M), $NaHCO_3$ (aq., sat.), $Na_2S_2O_3$ (sat.), water, brine, then dried over anhydrous $MgSO_4$ and concentrated. The solid was re-dispersed in pentane/EtOAc (7:1) and filtered through a glass filter, and this procedure was repeated 2 more times for the precipitate. The combined filtrates were concentrated and the product purified by silica gel column chromatography (pentane/EtOAc, 35:1 → 10:1) to afford partially separated anomers **8** (25.5 g, 95%).
α-anomer: ^{1}H NMR (500 MHz, $CDCl_3$) δ 7.90 – 7.81 (m, 4H), 7.56 – 7.48 (m, 3H), 7.38 – 7.28 (m, 5H), 4.92 (d, *J* = 4.2 Hz, 1H), 4.84 (d, *J* = 12.1 Hz, 1H), 4.74 (d, *J* = 12.1 Hz, 1H), 4.66 (d, *J* = 11.7 Hz, 1H), 4.59 (d, *J* = 11.7 Hz, 1H), 4.47 (q, *J* = 6.5 Hz, 1H), 4.30 – 4.24 (m, 1H), 4.13 – 4.07 (m, 1H), 3.46 (d, *J* = 0.9 Hz, 3H), 3.40 (ddd, *J* = 10.2, 5.9, 0.9 Hz, 1H), 3.25 (ddd, *J* = 10.2, 7.5, 0.9 Hz, 1H). ^{13}C NMR (126 MHz, $CDCl_3$) δ 137.76, 135.01, 133.25, 133.18, 128.45 (2C), 128.35, 127.96, 127.86, 127.80 (2C), 127.78, 127.09, 126.31, 126.17, 126.06, 100.91, 83.84, 81.80, 77.69, 72.90, 72.63, 55.61, 3.08. HRMS m/z: $[M+Na]^+$ calculated for $C_{24}H_{25}IO_4$ 527.0690, found 527.0687. **β-anomer:** ^{1}H NMR (500 MHz, $CDCl_3$) δ 7.91 – 7.85 (m, 3H), 7.82 – 7.78

(m, 1H), 7.58 – 7.50 (m, 2H), 7.48 (dd, *J* = 8.4, 1.7 Hz, 1H), 7.42 – 7.31 (m, 5H), 5.06 – 5.02 (m, 1H), 4.77 – 4.68 (m, 2H), 4.68 – 4.61 (m, 1H), 4.61 – 4.53 (m, 2H), 4.17 – 4.11 (m, 2H), 3.49 (dd, *J* = 10.1, 6.2 Hz, 1H), 3.48 (s, 3H), 3.43 (dd, *J* = 10.0, 8.3 Hz, 1H). ^{13}C NMR (126 MHz, $CDCl_3$) δ 137.48, 134.82, 133.26, 133.12, 128.50 (2C), 128.38, 128.04 (2C), 128.01, 127.96, 127.79, 126.74, 126.33, 126.16, 125.70, 108.36, 86.72, 82.06, 82.00, 72.74, 72.27, 56.14, 4.70. HRMS m/z: $[M+Na]^+$ calculated for $C_{24}H_{25}IO_4$ 527.0690, found 527.0688.

Compound **9**

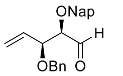

Iodide **8** (3.45 g, 6.84 mmol) was dissolved in 80 mL of THF/water (9:1 v/v), then the mixture was purged with nitrogen gas upon sonication over 15 minutes. Zinc powder was activated by stirring with HCl (aq., 3M), followed by filtration, washing with water, 1,4-dioxane, Et_2O, and drying. The thereby activated metal powder (4.02 g, 61.6 mmol) was added to the solution and the mixture was sonicated at room temperature under an inert atmosphere for 50 minutes with continuous monitoring of the reaction progress with TLC analysis. When TLC indicated full conversion of starting material, the precipitate was removed by vacuum filtration over a pad of celite and the filtrate was concentrated and the product purified by silica gel column chromatography (pentane/EtOAc, 12:1) to afford aldehyde **9** (2.19 g, 92%).

^{1}H NMR (400 MHz, $CDCl_3$) δ 9.70 (d, *J* = 1.5 Hz, 1H), 7.86 – 7.76 (m, 3H), 7.75 (s, 1H), 7.52 – 7.43 (m, 3H), 7.38 – 7.22 (m, 6H), 5.96 (ddd, *J* = 17.6, 10.5, 7.6 Hz, 1H), 5.41 – 5.27 (m, 2H), 4.92 (d, *J* = 12.2 Hz, 1H), 4.77 (d, *J* = 12.2 Hz, 1H), 4.64 (d, *J* = 12.1 Hz, 1H), 4.35 (d, *J* = 12.0 Hz, 1H), 4.18 (dd, *J* = 7.7, 4.1 Hz, 1H), 3.88 (dd, *J* = 4.1, 1.5 Hz, 1H). ^{13}C NMR (101 MHz, $CDCl_3$) δ 202.58, 137.63, 134.57, 133.92, 133.25, 128.49 (2C), 128.07, 128.05, 127.90, 127.84, 127.24, 126.35, 126.26, 126.06, 120.08, 85.13, 80.00, 73.62, 70.77. HRMS m/z: $[M+Na]^+$ calculated for $C_{23}H_{22}O_3$ 369.1461, found 369.1460.

Compound **10**

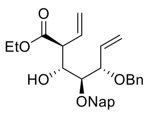

To aldehyde **9** (3.58 g, 10.33 mmol) dispersed in 24 mL of water and ethyl 4-bromocrotonate (11.4 mL, 62.0 mmol, 75% w/w) was added. Upon vigorous stirring of the reaction mixture, lanthanum(III) trifluoromethanesulfonate (12.7 g, 21.70 mmol) and indium powder (1.37 g, 11.89 mmol) were added and then 22 mL of water was added. After 4 hours of vigorous stirring, an additional amount of indium powder (1.37 g, 11.89 mmol) was added. When after 72 hours of vigorous stirring TLC analysis indicated the presence of starting material, more indium powder (0.210 g, 1.83 mmol) was added. After an additional 72 hours of vigorous stirring the reaction mixture was diluted with Et_2O, washed with HCl (aq., 1 M), water, brine, then concentrated and purified with silica gel column chromatography (petroleum ether/EtOAc, 9:1 → 6:1) to afford compound **10** (1.33 g, 28%) as well as poorly separable mixture of **10** and its stereoisomer (2.79 g, content of **10** is 80% according to ^{1}H NMR).

^{1}H NMR (400 MHz, $CDCl_3$) δ 7.88 – 7.77 (m, 3H), 7.75 (d, *J* = 1.6 Hz, 1H), 7.55 – 7.43 (m, 3H), 7.38 – 7.23 (m, 5H), 5.85 (ddd, *J* = 17.3, 10.3, 7.9 Hz, 1H), 5.73 (ddd, *J* = 17.1, 10.2, 9.3 Hz, 1H), 5.49 – 5.38 (m, 2H), 5.23 – 5.11 (m, 2H), 5.05 (dt, *J* = 17.1, 1.1 Hz, 1H), 4.76 (d, *J* = 11.4 Hz, 1H), 4.65 (d, *J* = 11.6 Hz, 1H), 4.42 (d, *J* = 11.6 Hz, 1H), 4.24 (t, *J* = 7.8 Hz, 1H), 4.10 (q, *J* = 7.1 Hz, 2H), 4.00 (d, *J* = 7.0 Hz, 1H), 3.80 – 3.71 (m, 1H), 3.61 (dd, *J* = 7.7, 1.2 Hz, 1H), 3.33 (t, *J* = 9.2 Hz, 1H), 1.21 (t, *J* = 7.1 Hz, 3H). ^{13}C NMR (101 MHz, $CDCl_3$) δ 172.64, 138.42, 136.00, 135.00, 133.37, 133.08, 132.98, 128.49 (2C), 128.22, 128.02, 127.96 (2C), 127.82, 127.69, 126.67, 126.23, 126.09, 126.02, 120.18, 120.07, 83.09, 79.71, 74.80, 72.28, 70.91, 60.98, 55.22, 14.20. HRMS m/z: $[M+Na]^+$ calculated for $C_{29}H_{32}O_5$ 483.2142, found 483.2140.

Compound **11**

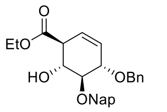

Diene **10** (0.400 g, 0.87 mmol) was dissolved in 12 mL of DCM, then 2nd generation Grubbs catalyst (0.018 g, 0.022 mmol) was added and the mixture was refluxed under continuous flow of nitrogen gas in the dark for 14 hours, after which it was concentrated and the product purified by silica gel column chromatography (pentane/EtOAc, 8:1 → 5:1) to afford compound **11** (0.360 g, 96%).

^{1}H NMR (500 MHz, $CDCl_3$) δ 7.88 – 7.79 (m, 4H), 7.50 (dtd, *J* = 6.8, 4.8, 3.1 Hz, 3H), 7.40 – 7.28 (m, 5H), 5.84 (dt, *J* = 10.2, 2.5 Hz, 1H), 5.71 (dt, *J* = 10.2, 2.2 Hz, 1H), 5.14 (d, *J* = 11.6 Hz, 1H), 5.00 (d, *J* = 11.6 Hz, 1H), 4.75 – 4.66 (m, 2H), 4.28 – 4.18 (m, 4H), 3.75 (dd, *J* = 9.8, 7.5 Hz, 1H), 3.30 (m, 1H), 3.14 (s, 1H), 1.29 (t, *J* = 7.1 Hz, 3H). ^{13}C NMR (126 MHz, $CDCl_3$) δ 172.04, 138.09, 135.96, 133.38, 133.08, 128.55 (2C), 128.34, 128.29, 128.01, 127.95 (2C), 127.89, 127.77, 126.70, 126.18, 125.98, 125.95, 124.13, 82.49, 79.31, 74.95, 71.93, 70.52, 61.37, 50.19, 14.22. HRMS m/z: $[M+Na]^+$ calculated for $C_{27}H_{28}O_5$ 455.1829, found 455.1827.

Compound **12**

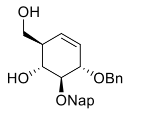

Ester **11** (1.90 g, 4.39 mmol) was dissolved in 30 mL of THF, then the mixture was cooled to 0 °C and diisobutylaluminum hydride solution in THF (24.2 mL, 24.16 mmol, 1 M) was added portion wise over 30 minutes. The reaction mixture was stirred for 1 hour at 0 °C and 2 hours at room temperature, after which 10 mL of EtOAc was added dropwise, followed by 20 mL of water. To the thus obtained viscous reaction mixture, sodium

borohydride (1.00 g, 26.4 mmol) was added, and the mixture was stirred overnight, then diluted with EtOAc and washed with Rochelle's salt (aq., sat.). The aqueous layer was extracted with EtOAc, then the combined organic layers were washed with brine, then dried over anhydrous $MgSO_4$ and filtered, after which the filtrate was concentrated and the product purified by silica gel column chromatography (pentane/EtOAc, 7:1 → 1.5:1) to afford compound **12** (1.43 g, 84%).
^{1}H NMR (500 MHz, $CDCl_3$) δ 7.87 – 7.81 (m, 2H), 7.84 – 7.79 (m, 1H), 7.78 (d, *J* = 1.6 Hz, 1H), 7.54 – 7.44 (m, 3H), 7.41 – 7.32 (m, 4H), 7.35 – 7.28 (m, 1H), 5.79 (dt, *J* = 10.2, 2.5 Hz, 1H), 5.51 (dt, *J* = 10.2, 2.0 Hz, 1H), 5.18 (d, *J* = 11.5 Hz, 1H), 4.92 (d, *J* = 11.5 Hz, 1H), 4.73 (d, *J* = 11.5 Hz, 1H), 4.65 (d, *J* = 11.5 Hz, 1H), 4.24 (m, 1H), 3.77 (dd, *J* = 10.6, 4.2 Hz, 1H), 3.77 – 3.66 (m, 3H), 3.12 (s, 1H), 2.62 (s, 1H), 2.54 – 2.47 (m, 1H). ^{13}C NMR (126 MHz, $CDCl_3$) δ 138.18, 135.90, 133.40, 133.11, 128.61, 128.48, 128.04, 128.01, 127.93, 127.83, 127.50, 127.49, 126.84, 126.30, 126.10, 125.96, 83.32, 80.32, 74.94, 72.78, 71.52, 65.37, 45.28. HRMS m/z: $[M+Na]^+$ calculated for $C_{25}H_{26}O_4$ 413.1723, found 413.1726.

Compound **14**

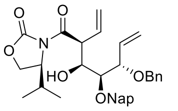

(S)-3-((E)-But-2-enoyl)-4-isopropyloxazolidin-2-one **13** (0.62 g, 3.15 mmol) was dissolved in 6 mL of DCM and the solution was cooled down to -78 °C. A solution of dibutylboryl trifluoromethanesulfonate (3.15 mL, 3.15 mmol, 1 M) was added portion wise followed by portion wise addition of TEA (0.49 mL, 3.48 mmol) and stirring for 1 hour at -78 °C. Aldehyde **9** (0.95 g, 2.74 mmol) in 5 mL of DCM was added portion wise at -78 °C, then the reaction mixture was allowed to warm up to -45 °C over 1 hour, then was kept at -25 °C for 1.5 hours and for another 1.5 hours at -15 °C. Finally, the bath was warmed to -5 °C and 5 mL phosphate buffer (pH 7.0, 1 M) was added, followed by dropwise addition of 0.40 mL of hydrogen peroxide solution (35% w/w) and stirring for an additional 45 minutes. After this, the reaction mixture was diluted with $NaHCO_3$ (aq., sat.) and extracted with DCM. The combined organic extracts were washed with brine, then dried over anhydrous $MgSO_4$ and filtered, after which the filtrate was concentrated and the product purified by silica gel column chromatography (pentane/EtOAc, 12:1 → 5:1) to afford compound **14** (1.33 g, 89%).
^{1}H NMR (400 MHz, CDCl3) δ 7.88 – 7.75 (m, 4H), 7.52 – 7.38 (m, 3H), 7.37 – 7.27 (m, 5H), 6.16 – 6.02 (m, 1H), 5.93 (ddd, J = 17.3, 10.2, 9.0 Hz, 1H), 5.49 – 5.36 (m, 3H), 5.29 (dd, J = 10.2, 1.5 Hz, 1H), 5.05 (dd, J = 9.0, 7.4 Hz, 1H), 4.85 (dd, J = 11.2, 0.8 Hz, 1H), 4.74 – 4.62 (m, 2H), 4.50 (ddd, J = 8.3, 7.4, 2.2 Hz, 1H), 4.44 (d, J = 11.7 Hz, 1H), 4.33 (m, 1H), 3.93 (dt, J = 8.7, 3.5 Hz, 1H), 3.65 (ddd, J = 8.3, 6.3, 3.6 Hz, 2H), 3.36 (d, J = 2.3 Hz, 1H), 2.87 (t, J = 8.8 Hz, 1H), 2.18 (m, 1H), 0.68 (dd, J = 7.0, 1.9 Hz, 6H). ^{13}C NMR (101 MHz, CDCl3) δ 172.63, 153.63, 137.79, 135.53, 134.58, 133.64, 133.23, 132.91, 128.53 (2C), 128.01 (3C), 127.96, 127.91, 127.70, 126.27, 126.02, 125.93, 125.64, 120.62, 119.37, 82.00, 80.05, 73.28, 71.24, 70.77, 62.20, 57.93, 50.29, 27.88, 17.76, 14.39. HRMS m/z: $[M+NH_4]^+$ calculated for $C_{33}H_{37}NO_6$ 561.2959, found 561.2953.

Compound **15**

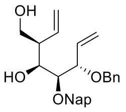

Compound **14** (2.96 g, 5.44 mmol) was dissolved in 25 mL of THF, and the solution was cooled to 0 °C. Then, water (1.1 mL, 62 mmol) and a solution of lithium borohydride in THF (3.3 mL, 6.54 mmol, 2 M) were added and the reaction mixture was stirred for 2.5 hours at 0 °C, after which TLC analysis indicated full conversion of starting material. The reaction was quenched with 7.5 mL of NaOH (aq., 3 M), then diluted with Et_2O and washed with water, brine, then dried over anhydrous $MgSO_4$ and filtered, after which the filtrate was concentrated and the product purified by silica gel column chromatography (pentane/EtOAc, 7:1 → 3:1) to afford compound **15** as white solid (2.01 g, 88%).
^{1}H NMR (400 MHz, CDCl3) δ 7.89 – 7.79 (m, 3H), 7.74 (d, J = 1.6 Hz, 1H), 7.57 – 7.46 (m, 2H), 7.43 (dd, J = 8.4, 1.7 Hz, 1H), 7.40 – 7.27 (m, 5H), 6.17 – 6.00 (m, 2H), 5.52 – 5.39 (m, 2H), 5.29 (dd, J = 10.4, 2.2 Hz, 1H), 5.18 (dd, J = 17.4, 2.2 Hz, 1H), 4.76 (d, J = 11.4 Hz, 1H), 4.73 – 4.65 (m, 2H), 4.41 (d, J = 11.8 Hz, 1H), 4.30 – 4.22 (m, 1H), 4.14 (dt, J = 9.0, 2.3 Hz, 1H), 3.80 (m, 2H), 3.61 (dd, J = 9.0, 3.7 Hz, 1H), 3.48 (dd, J = 2.6, 1.1 Hz, 1H), 2.67 (m, 1H), 2.40 (s, 1H). ^{13}C NMR (101 MHz, CDCl3) δ 137.41, 135.47, 135.44, 134.12, 133.27, 133.06, 128.63 (2C), 128.24, 128.09, 128.03 (2C), 127.97, 127.77, 126.61, 126.22, 126.04, 125.92, 119.58, 119.12, 79.81, 79.40, 73.39, 72.51, 71.06, 65.61, 47.47. HRMS m/z: $[M+Na]^+$ calculated for $C_{27}H_{30}O_4$ 441.2036, found 441.2034.

Compound **16**

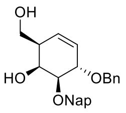

Diene **15** (3.31 g, 7.92 mmol) was dissolved in 112 mL of DCM and the solution was purged with argon flow upon sonification. 2nd Generation Grubbs catalyst (0.235 g, 0.260 mmol) was added and the reaction mixture was refluxed under continuous flow of nitrogen gas in the dark for 3.5 hours, after which TLC analysis indicated full conversion of starting material. The reaction mixture was concentrated and the product purified by silica gel column chromatography (pentane/EtOAc, 3:1 → 1:1) to yield compound **16** (2.55 g, 85%) as a solid.
^{1}H NMR (500 MHz, CDCl3) δ 7.88 – 7.75 (m, 4H), 7.52 – 7.45 (m, 3H), 7.41 – 7.35 (m, 2H), 7.34 (ddd, J = 7.3, 6.5, 1.5 Hz, 2H), 7.34 – 7.26 (m, 1H), 5.88 (dt, J = 10.2, 2.6 Hz, 1H), 5.61 – 5.54 (m, 1H), 4.90 (s, 2H), 4.74 (s, 2H), 4.41 – 4.33 (m, 2H), 3.83 (qd, J = 10.9, 4.9 Hz, 2H), 3.72 (dd, J = 7.6, 2.2 Hz, 1H), 2.82 (s, 1H), 2.78 (s, 1H), 2.48 (dt, J = 6.6, 3.1 Hz, 1H). ^{13}C NMR (126 MHz, CDCl3) δ 138.66, 135.58, 133.34, 133.15, 128.52 (2C), 128.43, 128.02, 127.92 (2C), 127.83, 127.75, 127.59, 126.93,

126.79, 126.33, 126.16, 125.95, 81.79, 76.80, 72.45, 72.28, 70.41, 63.79, 42.05. HRMS m/z: $[M+Na]^+$ calculated for $C_{25}H_{26}O_4$ 413.1723, found 413.1722.

Compound **17**

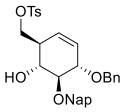

Alcohol **12** (3.05 g, 7.81 mmol) was dissolved in 75 mL of DCM and the solution was cooled to 0 °C, then TEA (3.3 mL, 23.43 mmol) and *p*-toluenesulfonyl chloride (2.23 g, 11.72 mmol) were added and the reaction mixture was stirred for 15 hours on an ice bath and 2 hours at room temperature, then diluted with EtOAc, washed with citric acid (aq., 0.5 M), water, and $NaHCO_3$ (aq., sat.). The aqueous layer was extracted with EtOAc, then the combined organic layers were washed with brine, dried over anhydrous $MgSO_4$ and filtered, after which the filtrate was concentrated and the product purified by silica gel column chromatography (pentane/EtOAc, 10:1 → 3:1) to afford compound **17** (3.80 g, 89%).

^{1}H NMR (500 MHz, $CDCl_3$) δ 7.87 – 7.73 (m, 6H), 7.53 – 7.45 (m, 2H), 7.44 (dd, *J* = 8.4, 1.7 Hz, 1H), 7.40 – 7.27 (m, 7H), 5.80 (ddd, *J* = 10.2, 2.8, 2.1 Hz, 1H), 5.57 (dt, *J* = 10.3, 2.0 Hz, 1H), 5.16 (dd, *J* = 11.6, 0.8 Hz, 1H), 4.88 (d, *J* = 11.6 Hz, 1H), 4.72 (d, *J* = 11.5 Hz, 1H), 4.64 (d, *J* = 11.5 Hz, 1H), 4.24 – 4.17 (m, 2H), 4.14 (dd, *J* = 9.5, 5.4 Hz, 1H), 3.68 – 3.57 (m, 2H), 2.70 (d, *J* = 1.7 Hz, 1H), 2.54 (tdd, *J* = 5.5, 4.2, 2.4 Hz, 1H), 2.43 (s, 3H). ^{13}C NMR (126 MHz, $CDCl_3$) δ 144.95, 138.08, 135.82, 133.38, 133.10, 132.83, 129.97, 128.62, 128.47, 128.04, 128.02, 127.96, 127.83, 126.80, 126.42, 126.31, 126.12, 125.88, 83.29, 80.08, 74.94, 71.62, 69.63, 68.99, 43.15, 21.74. HRMS m/z: $[M+Na]^+$ calculated for $C_{32}H_{32}O_6S$ 567.1812, found 567.1811

Compound **18**

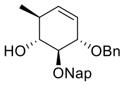

Tosylate **17** (6.40 g, 11.75 mmol) was dissolved in 100 mL of THF, then the reaction mixture was cooled to 0 °C and lithium aluminium hydride solution (23.5 mL, 23.50 mmol, 1 M) was added portion wise. After 5 hours of stirring at room temperature, the reaction mixture was cooled back to 0 °C and quenched with 1 mL of NaOH solution (aq., 10% w/w). The thus obtained suspension was dried over anhydrous $MgSO_4$ and filtered, after which the filtrate was concentrated and the product purified by silica gel column chromatography (pentane/EtOAc, 15:1 → 8:1) to afford compound **18** (3.35 g, 76%).

^{1}H NMR (500 MHz, $CDCl_3$) δ 7.88 – 7.82 (m, 2H), 7.85 – 7.79 (m, 1H), 7.81 – 7.77 (m, 1H), 7.54 – 7.46 (m, 3H), 7.43 – 7.37 (m, 2H), 7.37 (dd, *J* = 8.3, 6.2 Hz, 2H), 7.36 – 7.29 (m, 1H), 5.68 (dt, *J* = 10.1, 2.4 Hz, 1H), 5.52 (dt, *J* = 10.1, 2.0 Hz, 1H), 5.21 (d, *J* = 11.5 Hz, 1H), 4.92 (d, *J* = 11.5 Hz, 1H), 4.75 (d, *J* = 11.5 Hz, 1H), 4.67 (d, *J* = 11.5 Hz, 1H), 4.27 (m, 1H), 3.67 (ddd, *J* = 10.0, 7.7, 0.8 Hz, 1H), 3.39 (t, *J* = 9.5 Hz, 1H), 2.80 (s, 1H), 2.35 (ddd, *J* = 9.5, 6.6, 3.2 Hz, 1H), 1.19 – 1.14 (m, 3H). ^{13}C NMR (126 MHz, $CDCl_3$) δ 138.36, 136.06, 133.43, 133.12, 132.98, 128.59, 128.47, 128.04, 128.00, 127.87, 127.83, 126.80, 126.26, 126.05, 125.97, 124.59, 83.80, 80.89, 75.21, 74.92, 71.38, 37.94, 18.25. HRMS m/z: $[M+Na]^+$ calculated for $C_{25}H_{26}O_3$ 397.1774, found 397.1771.

Compound **19**

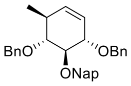

Alcohol **18** (2.15 g, 5.74 mmol) was dissolved in 40 mL of DMF, then benzyl bromide (0.75 mL, 6.32 mmol) and tetrabutylammonium iodide (0.021 g, 0.057 mmol) were added. The reaction mixture was cooled to 0 °C and sodium hydride (60% dispersion in mineral oil, 0.26 g, 6.60 mmol) was added portion wise and the mixture was allowed to warm up to room temperature and stirred for 3 hours. After this, it was re-cooled to 0 °C, quenched with water, diluted with Et_2O, washed with water, brine, then dried over anhydrous $MgSO_4$ and filtered, after which the filtrate was concentrated and the product purified by silica gel column chromatography (pentane/Ac, 30:1) to afford compound **19** (2.54 g, 95%).

^{1}H NMR (400 MHz, $CDCl_3$) δ 7.95 – 7.78 (m, 4H), 7.62 – 7.50 (m, 3H), 7.49 – 7.32 (m, 10H), 5.71 (dt, *J* = 10.1, 2.5 Hz, 1H), 5.58 (dt, *J* = 10.1, 1.9 Hz, 1H), 5.18 (s, 2H), 5.12 (d, *J* = 10.9 Hz, 1H), 4.81 (s, 2H), 4.74 (d, *J* = 11.0 Hz, 1H), 4.38 (m, 1H), 3.96 (dd, *J* = 10.1, 7.8 Hz, 1H), 3.39 (t, *J* = 9.7 Hz, 1H), 2.62 – 2.50 (m, 1H), 1.20 (d, *J* = 7.0 Hz, 3H). ^{13}C NMR (101 MHz, $CDCl_3$) δ 138.79, 138.63, 136.51, 133.43, 133.01, 132.79, 128.48, 128.46, 128.10, 128.07, 128.02, 127.89, 127.74, 127.70, 127.68, 126.60, 126.22, 126.02, 125.81, 125.20, 85.15, 84.49, 81.27, 75.61, 75.33, 71.99, 38.30, 18.19. HRMS m/z: $[M+Na]^+$ calculated for $C_{32}H_{32}O_3$ 487.2244, found 487.2240.

Compound **20**

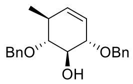

Compound **19** (2.44 g, 5.25 mmol) was dissolved in 120 mL of DCM, then water (12 mL) and β-pinene (2.5 mL, 15.76 mmol) were added. The reaction mixture was cooled to 0 °C and DDQ (1.79 g, 7.88 mmol) was added portion wise and the mixture was allowed to warm up to room temperature and stirred for 5 hours. Then it was diluted with EtOAc, washed with $NaHCO_3$ (aq., sat.), brine, dried over anhydrous $MgSO_4$ and filtered, after which the filtrate was concentrated and the product purified by silica gel column chromatography (pentane/EtOAc, 30:1 → 15:1) to afford compound **20** (1.55 g, 91%).

^{1}H NMR (400 MHz, $CDCl_3$) δ 7.47 – 7.31 (m, 10H), 5.65 (dt, J = 10.1, 2.4 Hz, 1H), 5.52 (dt, J = 10.1, 2.0 Hz, 1H), 4.91 (d, J = 11.1 Hz, 1H), 4.84 – 4.72 (m, 3H), 4.16 (m, 1H), 3.96 (ddd, J = 9.8, 7.8, 1.5 Hz, 1H), 3.25 (dd, J = 10.1, 9.1 Hz, 1H), 2.93 (d, J = 1.8 Hz, 1H), 2.54 – 2.41 (m, 1H), 1.18 (d, J = 7.0 Hz, 3H). ^{13}C NMR (101 MHz, $CDCl_3$) δ 138.61, 138.48, 132.83, 128.50, 128.42, 127.98, 127.82, 127.81, 127.63, 125.02, 84.70, 80.52, 76.44, 74.90, 71.66, 37.39, 18.34. HRMS m/z: $[M+Na]^+$ calculated for $C_{21}H_{24}O_3$ 347.1618, found 347.1614.

Compound **21**

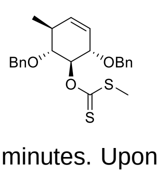

Diisopropylamine (1.3 mL, 9.25 mmol) was dissolved in 11 mL of THF, the solution was cooled to -75 °C and *n*-butyllithium solution in hexanes (5.5 mL, 8.86 mmol, 1.6 M) was added dropwise and the mixture was stirred for 10 minutes at -75 °C, followed by 30 minutes at 0 °C and cooling back to -75 °C. Alcohol **20** (1.25 g, 3.85 mmol) was added dropwise to the reaction mixture in 8 mL of THF, and the latter stirred at -75 °C for another 10 minutes. Upon dropwise addition of carbon disulfide (4.0 mL, 65.5 mmol), the reaction mixture changed color from yellow to orange. After the mixture was stirred at -75 °C for 3 hours, iodomethane (1.7 mL, 27 mmol) was added dropwise and the reaction mixture was allowed to warm up to 0 °C over 3.5 hours followed by overnight stirring at 4 °C. Next day, the reaction was quenched with 5 mL of $NaHCO_3$ (aq., sat.), diluted with Et_2O and washed with water. The aqueous layer was extracted with Et_2O, then the combined organic layers were washed with brine, dried over anhydrous $MgSO_4$ and filtered, after which the filtrate was concentrated and the product purified by silica gel column chromatography (pentane/EtOAc, 80:1 → 10:1) to afford impurified xanthate **21** fractions (1.16 g) and re-isolated starting material **20** (0.37 g). Xanthate was impurified with an unidentified yellow substance (visible with the TLC analysis) that is poorly separable via silica gel column chromatography but does not show up in the NMR spectrum.
^{1}H NMR (400 MHz, $CDCl_3$) δ 7.40 – 7.24 (m, 10H), 6.44 (dd, J = 10.2, 7.9 Hz, 1H), 5.65 (dt, J = 10.1, 2.4 Hz, 1H), 5.56 (dt, J = 10.1, 2.0 Hz, 1H), 4.77 (d, J = 10.6 Hz, 1H), 4.70 – 4.57 (m, 2H), 4.51 (d, J = 10.6 Hz, 1H), 4.42 (m, 1H), 3.44 (dd, J = 10.2, 9.1 Hz, 1H), 2.62 (s, 3H), 2.62 – 2.51 (m, 1H), 1.12 (d, J = 7.0 Hz, 3H). ^{13}C NMR (101 MHz, $CDCl_3$) δ 215.82, 138.22, 138.07, 133.15, 128.46, 128.41, 128.34, 128.02, 127.85, 127.75, 125.01, 85.46, 82.62, 78.00, 75.21, 71.02, 37.84, 19.42, 18.06.

Compound **22**

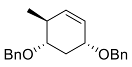

Impure xanthate **21** was split equally over two reaction flasks (0.58 g of starting material each). To each flask was added toluene (10 mL) and the solution was purged with nitrogen gas flow over 10 minutes. Tributyltin hydride (1.05 mL, 3.91 mmol) and azobisisobutyronitrile solution in toluene (0.65 mL, 0.130 mmol, 0.2 M) were added to each flask and the mixture was refluxed over 1 hour. When TLC indicated full conversion of xanthate, both reaction mixtures were combined and directly loaded on a silica gel column, then eluted with (pentane/EtOAc, 100:1 → 40:1) to afford alkene **22** (0.67 g, 56% over two steps, 80% over 2 steps based on re-isolated starting material).
^{1}H NMR (300 MHz, $CDCl_3$) δ 7.46 – 7.26 (m, 10H), 5.82 – 5.71 (m, 1H), 5.59 (dt, J = 10.1, 2.0 Hz, 1H), 4.74 (d, J = 11.5 Hz, 1H), 4.72 – 4.57 (m, 2H), 4.51 (d, J = 11.5 Hz, 1H), 4.20 (m, 1H), 3.22 (ddd, J = 11.9, 8.8, 3.1 Hz, 1H), 2.62 (m, 1H), 2.42 (m, J = 10.8, 5.5, 2.4 Hz, 1H), 1.69 (td, J = 11.8, 10.1 Hz, 1H), 1.14 (d, J = 7.0 Hz, 3H). ^{13}C NMR (75 MHz, $CDCl_3$) δ 138.70, 133.62, 128.47, 128.45, 127.87, 127.74, 127.68, 127.63, 127.46, 79.96, 74.51, 70.89, 70.06, 37.67, 34.18, 18.41. HRMS m/z: $[M+Na]^+$ calculated for $C_{21}H_{24}O_2$ 331.1669, found 331.1671.

Compounds **23** and **24**

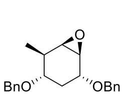

23

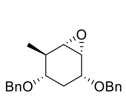

24

Method A: Alkene **22** (0.440 g, 1.43 mmol) was treated according to General procedure A. Purification by silica gel column chromatography (pentane/Ac, 70:1 → 40:1) afforded titled compounds (**23**: 317 mg, 69%; **24**: 88 mg, 19%).

Method B: Alkene **22** (0.230 g, 0.75 mmol) was treated according to General procedure B. Purification by silica gel column chromatography (pentane/Ac, 70:1 → 40:1) afforded titled compounds (**23**: 172 mg, 71%; **24**: 40 mg, 17%).

23: ^{1}H NMR (500 MHz, $CDCl_3$) δ 7.43 – 7.28 (m, 10H), 4.75 – 4.65 (m, 2H), 4.62 (d, J = 11.4 Hz, 1H), 4.41 (d, J = 11.4 Hz, 1H), 3.78 (ddd, J = 10.8, 6.1, 0.9 Hz, 1H), 3.27 (dd, J = 3.7, 1.3 Hz, 1H), 3.22 (ddd, J = 3.7, 1.9, 0.9 Hz, 1H), 3.09 (ddd, J = 12.0, 9.2, 3.0 Hz, 1H), 2.41 (dddd, J = 12.0, 6.1, 3.0, 1.3 Hz, 1H), 2.02 (dqd, J = 8.8, 6.8, 2.0 Hz, 1H), 1.44 (td, J = 11.8, 10.7 Hz, 1H), 1.28 (d, J = 6.8 Hz, 3H). ^{13}C NMR (126 MHz, $CDCl_3$) δ 138.47, 137.94, 128.58 (2C), 128.47 (2C), 127.91, 127.88 (2C), 127.80 (2C), 127.75, 75.61, 73.25, 71.69, 71.40, 59.73, 56.05, 37.33, 34.11, 14.95. HRMS m/z: $[M+Na]^+$ calculated for $C_{21}H_{24}O_3$ 347.1618, found 347.1614. **24**: ^{1}H NMR (400 MHz, $CDCl_3$) δ 7.45 – 7.27 (m, 10H), 4.76 – 4.66 (m, 2H), 4.63 (d, J = 11.5 Hz, 1H), 4.38 (d, J = 11.5 Hz, 1H), 3.87 (ddd, J = 11.2, 5.2, 1.7 Hz, 1H), 3.35 – 3.29 (m, 1H), 2.93 – 2.82 (m, 2H), 2.16 (dddd, J = 11.7, 5.1, 3.1, 1.1 Hz, 1H), 2.03 (dq, J = 9.5, 7.3 Hz, 1H), 1.62 (q, J = 11.7 Hz, 1H), 1.14 (d, J = 7.2 Hz, 3H). ^{13}C NMR (101 MHz, $CDCl_3$) δ 138.37, 138.35, 128.56 (2C), 128.50 (2C), 127.92 (2C), 127.82 (3C), 127.80, 79.56, 74.02, 70.59, 70.44, 56.77, 53.94, 35.54, 28.50, 16.89.

Compound **25**

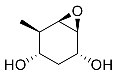

Epoxide **23** (0.048 g, 0.148 mmol) was treated according to General procedure C. Purification by silica gel column chromatography (DCM/MeOH, 30:1) afforded compound **25** (0.0106 g, 50%).

^{1}H NMR (500 MHz, $CDCl_3$) δ 3.96 – 3.85 (m, 1H), 3.19 (tt, *J* = 6.5, 3.7 Hz, 2H), 3.07 (dd, *J* = 3.7, 1.4 Hz, 1H), 2.04 (dddd, *J* = 7.3, 4.3, 3.1, 1.5 Hz, 1H), 1.85 – 1.71 (m, 1H), 1.43 – 1.28 (m, 1H), 1.19 (dd, *J* = 6.9, 1.8 Hz, 3H). ^{13}C NMR (126 MHz, $CDCl_3$) δ 68.78, 67.14, 60.39, 59.03, 40.59, 39.73, 14.95.

Compound **26**

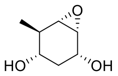

Epoxide **24** (0.025 g, 0.077 mmol) was treated according to General procedure C. Purification by silica gel column chromatography (DCM/MeOH, 30:1) afforded compound **26** (0.0057 g, 52%).

^{1}H NMR (500 MHz, MeOD) δ 4.04 (ddd, *J* = 11.0, 5.4, 1.7 Hz, 1H), 3.19 (dtd, *J* = 3.9, 1.1, 0.5 Hz, 1H), 3.05 (ddd, *J* = 12.4, 9.3, 3.3 Hz, 1H), 2.89 (d, *J* = 3.9 Hz, 1H), 1.79 (dddd, *J* = 11.6, 5.4, 3.3, 1.1 Hz, 1H), 1.69 (dq, *J* = 9.5, 7.3 Hz, 1H), 1.55 – 1.46 (m, 1H), 1.15 (d, *J* = 7.3 Hz, 3H). ^{13}C NMR (126 MHz, MeOD) δ 73.04, 68.31, 58.22, 57.22, 38.61, 36.81, 16.93. HRMS m/z: $[M+Na]^+$ calculated for $C_7H_{12}O_3$ 167.0679, found 167.0679.

Compound **27**

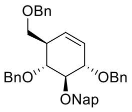

Alcohol **12** (2.30 g, 5.89 mmol) was dissolved in 32 mL of DMF, then benzyl bromide (1.7 mL, 14.14 mmol) and tetrabutylammonium iodide (0.044 g, 0.118 mmol) were added. The reaction mixture was cooled to 0 °C and sodium hydride (60% dispersion in mineral oil, 0.57 g, 14.14 mmol) was added portion wise and the mixture was allowed to warm up to 10 °C over 3 hours. Afterwards, it was re-cooled to 0 °C, quenched with water, diluted with Et_2O, washed with water, brine, dried over anhydrous $MgSO_4$ and filtered, after which the filtrate was concentrated and the product purified by silica gel column chromatography (pentane/Ac, 40:1→ 15:1) to afford compound **27** (3.3 g, 98%).

^{1}H NMR (400 MHz, $CDCl_3$) δ 7.96 – 7.77 (m, 4H), 7.62 – 7.50 (m, 3H), 7.48 – 7.32 (m, 13H), 7.28 (dt, *J* = 6.5, 2.4 Hz, 2H), 5.89 – 5.74 (m, 2H), 5.17 (s, 2H), 5.05 (d, *J* = 10.9 Hz, 1H), 4.81 (s, 2H), 4.61 – 4.46 (m, 3H), 4.40 (ddt, *J* = 7.6, 3.6, 1.8 Hz, 1H), 3.97 (dd, *J* = 10.1, 7.8 Hz, 1H), 3.81 (t, *J* = 9.8 Hz, 1H), 3.63 (d, *J* = 4.0 Hz, 2H), 2.72 – 2.60 (m, 1H). ^{13}C NMR (101 MHz, $CDCl_3$) δ 138.68, 138.59, 138.29, 136.48, 133.43, 133.01, 129.33, 128.49 (2C), 128.46 (2C), 128.43 (2C), 128.11 (3C), 128.02, 127.94 (2C), 127.87 (2C), 127.74 (2C), 127.70, 127.68, 126.93, 126.58, 126.20, 126.03, 125.82, 85.38, 80.93, 78.48, 77.36, 75.38 (2C), 73.18, 72.07, 69.22, 44.49. HRMS m/z: $[M+NH_4]^+$ calculated for $C_{39}H_{38}O_4$ 588.3108, found 588.3106.

Compound **28**

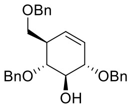

Compound **27** (3.20 g, 5.61 mmol) was dissolved in 52 mL of DCM, then water (3 mL) was added. The reaction mixture was cooled to 0 °C, and DDQ (1.53 g, 6.73 mmol) was added portion wise and the mixture was stirred at this temperature until TLC indicated full conversion of starting material (3 hours). Then, the reaction mixture was diluted with EtOAc, washed with $NaHCO_3$ (aq., sat.), brine, dried over anhydrous $MgSO_4$ and filtered, after which the filtrate was concentrated and the product purified by silica gel column chromatography (pentane/EtOAc, 20:1 → 4:1) to afford compound **28** (2.14 g, 89%).

^{1}H NMR (400 MHz, $CDCl_3$) δ 7.45 – 7.28 (m, 15H), 5.81 – 5.67 (m, 2H), 4.83 (d, *J* = 11.1 Hz, 1H), 4.76 (s, 2H), 4.61 – 4.44 (m, 3H), 4.20 – 4.12 (m, 1H), 3.95 (ddd, *J* = 10.0, 7.8, 2.1 Hz, 1H), 3.69 – 3.55 (m, 3H), 2.76 (d, *J* = 2.1 Hz, 1H), 2.61 – 2.49 (m, 1H). ^{13}C NMR (101 MHz, $CDCl_3$) δ 138.61, 138.48, 138.17, 129.53, 128.56 (2C), 128.52 (2C), 128.48 (2C), 128.10 (2C), 127.93 (4C), 127.88, 127.80, 127.76, 126.70, 80.28, 78.82, 76.80, 74.97, 73.24, 71.80, 69.35, 43.77. HRMS m/z: $[M+Na]^+$ calculated for $C_{28}H_{30}O_4$ 453.2036, found 453.2032.

Compound **29**

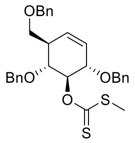

Alcohol **28** (2.04 g, 4.74 mmol) was dissolved in 16 mL of THF, then carbon disulfide (4.9 mL, 81.0 mmol) was added and the reaction mixture was cooled to -85 °C. Lithium diisopropylamide solution in THF/hexanes (4.7 mL, 9.48 mmol, 2 M) was added dropwise over 10 minutes and the mixture was allowed to warm up to -55 °C over 2 hours, then re-cooled to -85 °C and iodomethane (2.1 mL, 33.2 mmol) was added dropwise and the reaction mixture was allowed to warm up to 3 °C over 4 hours. Then, the reaction was quenched with 5 mL of $NaHCO_3$ (aq., sat.), diluted with Et_2O and washed with water. The aqueous layer was extracted with Et_2O, and the combined organic layers were washed with brine, dried over anhydrous $MgSO_4$ and filtered, after which the filtrate was concentrated and the product purified by silica gel column chromatography (pentane/EtOAc, 100:1 → 7:1) to afford xanthate **29** (1.55 g, 63%) and re-isolated starting material **28** (0.70 g, 34%).

^{1}H NMR (400 MHz, $CDCl_3$) δ 7.39 – 7.26 (m, 13H), 7.20 – 7.16 (m, 2H), 6.44 (dd, *J* = 10.2, 7.9 Hz, 1H), 5.80 – 5.69 (m, 2H), 4.71 – 4.58 (m, 3H), 4.51 – 4.37 (m, 3H), 4.32 (d, *J* = 10.6 Hz, 1H), 3.88 (dd, *J* = 10.2, 9.4 Hz, 1H), 3.61 – 3.47 (m, 2H), 2.67 – 2.61 (m, 1H), 2.60 (s, 3H). ^{13}C NMR (101 MHz, $CDCl_3$) δ 215.85, 138.20, 138.17, 138.02, 129.86, 128.55 (2C), 128.49 (2C),

128.42 (4C), 128.08 (2C), 128.00 (2C), 127.88, 127.86, 127.79, 126.79, 85.76, 77.67, 76.82, 75.20, 73.27, 71.15, 68.72, 44.06, 19.43.

Compound **30**

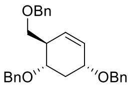

Xanthate **29** (1.55 g, 2.98 mmol) was dissolved in toluene (6 mL) and the solution was purged with nitrogen gas flow over 10 minutes. Tributyltin hydride (2.4 mL, 8.93 mmol) and azobisisobutyronitrile solution in toluene (1.5 mL, 0.30 mmol, 0.2 M) were added and the mixture was refluxed over 2 hours. When TLC analysis indicated full conversion of the xanthate, the reaction mixture was concentrated and the product purified by silica gel column chromatography (pentane/EtOAc, 50:1 → 10:1) to afford compound **30** (1.10 g, 89%).

^{1}H NMR (400 MHz, $CDCl_3$) δ 7.49 – 7.27 (m, 15H), 5.90 (ddt, *J* = 10.2, 3.0, 1.5 Hz, 1H), 5.80 (dt, *J* = 10.2, 1.9 Hz, 1H), 4.73 – 4.35 (m, 6H), 4.22 (dtt, *J* = 11.2, 3.7, 1.8 Hz, 1H), 3.70 – 3.55 (m, 3H), 2.65 (dddd, *J* = 11.5, 5.7, 3.3, 1.3 Hz, 1H), 2.55 (dddd, *J* = 12.6, 7.4, 3.6, 1.8 Hz, 1H), 1.71 (td, *J* = 11.8, 10.2 Hz, 1H). ^{13}C NMR (101 MHz, $CDCl_3$) δ 138.64, 138.53, 138.50, 129.95, 129.09, 128.49 (2C), 128.45 (2C), 128.43 (2C), 127.94 (2C), 127.80 (2C), 127.78 (2C), 127.72, 127.67, 127.65, 74.13, 74.10, 73.19, 70.98, 70.15, 69.67, 43.83, 34.26. HRMS m/z: $[M+Na]^+$ calculated for $C_{28}H_{30}O_3$ 437.2087, found 437.2092.

Compounds **31** and **32**

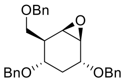

31

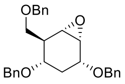

32

Alkene **30** was split over 2 flasks of (0.47 g, 1.13 mmol) and each of them was treated according to General procedure B. Then both reaction mixtures were combined and purification by silica gel column chromatography (pentane/EtOAc, 20:1 → 10:1) afforded titled compounds (**31**: 380 mg, 39%; **32**: 350 mg, 36%).

31: ^{1}H NMR (400 MHz, $CDCl_3$) δ 7.44 – 7.27 (m, 15H), 4.77 – 4.66 (m, 2H), 4.60 (s, 3H), 4.35 (d, *J* = 11.4 Hz, 1H), 3.89 (dd, *J* = 8.9, 3.8 Hz, 1H), 3.81 (ddd, *J* = 10.5, 6.2, 0.9 Hz, 1H), 3.64 (t, *J* = 8.9 Hz, 1H), 3.59 (ddd, *J* = 3.8, 2.0, 0.9 Hz, 1H), 3.33 (dd, *J* = 3.7, 1.1 Hz, 1H), 3.30 – 3.21 (m, 1H), 2.42 (dddd, *J* = 12.1, 6.2, 3.1, 1.3 Hz, 1H), 2.30 (tdd, *J* = 8.9, 3.8, 1.9 Hz, 1H), 1.57 – 1.45 (m, 1H). ^{13}C NMR (101 MHz, $CDCl_3$) δ 138.45, 138.20, 137.86, 128.59 (2C), 128.46 (2C), 128.43 (2C), 127.94, 127.83 (4C), 127.77, 127.68 (2C), 127.61, 73.28, 72.83, 71.44, 71.40, 71.29, 68.94, 56.34, 55.33, 42.87, 33.71. HRMS m/z: $[M+Na]^+$ calculated for $C_{28}H_{30}O_4$ 453.2036, found 453.2031. **32**: ^{1}H NMR (400 MHz, $CDCl_3$) δ 7.44 – 7.22 (m, 15H), 4.71 (d, *J* = 2.5 Hz, 2H), 4.58 (d, *J* = 11.4 Hz, 1H), 4.52 – 4.37 (m, 2H), 4.29 (d, *J* = 11.4 Hz, 1H), 3.90 (ddd, *J* = 11.3, 5.2, 1.6 Hz, 1H), 3.67 – 3.59 (m, 2H), 3.39 – 3.31 (m, 2H), 3.22 (d, *J* = 4.0 Hz, 1H), 2.19 (dddd, *J* = 11.7, 4.8, 3.3, 1.1 Hz, 1H), 2.11 (dt, *J* = 9.9, 3.7 Hz, 1H), 1.62 (q, *J* = 11.7 Hz, 1H). ^{13}C NMR (101 MHz, $CDCl_3$) δ 138.43, 138.30, 138.24, 128.57 (2C), 128.51 (4C), 128.00 (2C), 127.88 (2C), 127.83 (5C), 73.85, 73.32, 73.29, 70.77, 70.50, 68.62, 54.91, 53.86, 41.86, 28.49.

Compounds **33**

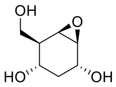

Epoxide **31** (0.030 g, 0.070 mmol) was treated according to General procedure C. Purification by silica gel column chromatogr aphy (DCM/MeOH, 20:1→ 10:1) afforded compound **33** (0.0077 g, 69%).

^{1}H NMR (400 MHz, MeOD) δ 3.93 (dt, *J* = 10.1, 5.1 Hz, 2H), 3.67 (dd, *J* = 10.6, 8.5 Hz, 1H), 3.43 (ddd, *J* = 3.3, 2.2, 0.8 Hz, 1H), 3.36 (ddd, *J* = 11.4, 8.5, 3.1 Hz, 1H), 3.11 (dd, *J* = 3.7, 1.1 Hz, 1H), 2.03 (dddd, *J* = 12.2, 6.0, 3.1, 1.1 Hz, 1H), 1.91 (tdd, *J* = 8.5, 5.1, 2.2 Hz, 1H), 1.40 (ddd, *J* = 12.3, 11.1, 9.8 Hz, 1H). ^{13}C NMR (101 MHz, MeOD) δ 66.80, 65.15, 62.46, 58.17, 56.50, 47.21, 40.11. HRMS m/z: $[M+Na]^+$ calculated for $C_7H_{12}O_4$ 183.0628, found 183.0630.

Compounds **34**

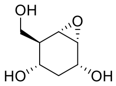

Epoxide **32** (0.035 g, 0.081 mmol) was treated according to General procedure C. Purification by silica gel column chromatography (DCM/MeOH, 10:1 → 5:1) afforded compound **34** (0.0099 g, 76%).

^{1}H NMR (400 MHz, MeOD) δ 4.04 (ddd, *J* = 11.1, 5.4, 1.6 Hz, 1H), 3.85 (dd, *J* = 10.8, 3.8 Hz, 1H), 3.67 (dd, *J* = 10.8, 6.4 Hz, 1H), 3.37 (ddd, *J* = 12.3, 9.6, 3.7 Hz, 1H), 3.23 – 3.19 (m, 1H), 3.18 (d, *J* = 4.0 Hz, 1H), 1.82 (dddd, *J* = 11.6, 4.9, 3.5, 1.0 Hz, 1H), 1.75 (ddd, *J* = 9.9, 6.4, 3.8 Hz, 1H), 1.53 (dt, *J* = 12.2, 11.3 Hz, 1H). ^{13}C NMR (101 MHz, MeOD) δ 68.07, 67.23, 62.18, 57.09, 55.72, 46.42, 36.56. HRMS m/z: $[M+Na]^+$ calculated for $C_7H_{12}O_4$ 183.0628, found 183.0629.

Compounds **35** and **36**

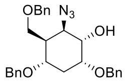

35

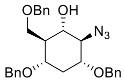

36

Epoxide **32** (0.370 g, 0.86 mmol) was treated according to General procedure D. Purification by silica gel column chromatography (pentane/DCM/Ac, 25:5:1 → 20:5:1) and (DCM/Ac, 60:1) yielded partially separated titled compounds (0.360 g in total, 88%).

35: ^{1}H NMR (500 MHz, $CDCl_3$) δ 7.45 – 7.26 (m, 15H), 4.67 – 4.49 (m, 5H), 4.38 (d, *J* = 11.5 Hz, 1H), 4.27 (t, *J* = 3.7 Hz, 1H), 4.12 – 4.05 (m, 1H), 3.97 – 3.89 (m, 1H), 3.65 (ddd, *J* = 11.8, 4.7, 2.9 Hz, 1H), 3.56 – 3.48 (m, 1H), 3.40 (td, *J* = 10.8, 4.2 Hz, 1H), 2.59 – 2.46 (m, 2H), 2.27 (dt, *J* = 11.9, 4.4 Hz, 1H), 1.81 – 1.70 (m, 1H). ^{13}C NMR (126 MHz, $CDCl_3$) δ 138.42 (2C), 137.94, 128.67 (2C), 128.50 (2C), 128.42 (2C), 128.09, 127.89 (2C), 127.86 (2C), 127.76, 127.71

(2C), 127.65, 74.12, 73.37, 73.24, 70.96, 70.83, 68.13, 68.09, 61.69, 40.57, 30.66. HRMS m/z: $[M+Na]^+$ calculated for $C_{28}H_{31}N_3O_4$ 496.2207, found 496.2215. **36**: ^1H NMR (500 MHz, $CDCl_3$) δ 7.42 – 7.27 (m, 13H), 7.26 – 7.24 (m, 2H), 4.72 – 4.44 (m, 5H), 4.37 (d, *J* = 11.3 Hz, 1H), 3.90 (dd, *J* = 9.1, 2.7 Hz, 1H), 3.69 (dd, *J* = 9.1, 5.1 Hz, 1H), 3.46 (t, *J* = 10.0 Hz, 1H), 3.38 (t, *J* = 9.6 Hz, 1H), 3.36 – 3.23 (m, 2H), 3.20 (bs, 1H), 2.56 (dt, *J* = 12.2, 4.3 Hz, 1H), 1.80 (tdd, *J* = 10.5, 5.1, 2.7 Hz, 1H), 1.45 (q, *J* = 11.8 Hz, 1H). ^{13}C NMR (126 MHz, $CDCl_3$) δ 138.13, 138.07, 137.90, 128.62 (2C), 128.57 (4C), 128.01 (3C), 127.95, 127.93 (2C), 127.91, 127.85 (2C), 75.93, 73.56, 72.05, 71.85, 71.59, 71.27, 70.77, 67.56, 48.53, 35.01. HRMS m/z: $[M+Na]^+$ calculated for $C_{28}H_{31}N_3O_4$ 496.2207, found 496.2218.

Compound **37**

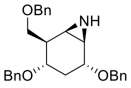

The mixture of compounds **35** and **36** (0.290 g, 0.61 mmol) was treated according to General procedure E. Purification by silica gel column chromatography (pentane/EtOAc + 1% TEA, 5:1 → 4:1) yielded compound **37** (0.240 g, 91%).

^1H NMR (500 MHz, $CDCl_3$) δ 7.44 – 7.26 (m, 15H), 4.72 – 4.63 (m, 2H), 4.62 – 4.52 (m, 3H), 4.32 (d, *J* = 11.4 Hz, 1H), 3.91 (dd, *J* = 8.8, 3.7 Hz, 1H), 3.74 (dd, *J* = 10.4, 6.0 Hz, 1H), 3.62 (t, *J* = 9.1 Hz, 1H), 3.15 (ddd, *J* = 11.5, 9.5, 3.1 Hz, 1H), 2.71 (dd, *J* = 6.0, 3.3 Hz, 1H), 2.44 (d, *J* = 6.0 Hz, 1H), 2.36 (ddd, *J* = 12.0, 6.0, 3.0 Hz, 1H), 2.25 (tt, *J* = 9.5, 3.5 Hz, 1H), 1.50 – 1.41 (m, 1H). ^{13}C NMR (126 MHz, $CDCl_3$) δ 138.70, 138.48, 138.31, 128.52 (2C), 128.41 (2C), 128.40 (2C), 127.80 (4C), 127.76, 127.72 (2C), 127.65, 127.56, 75.09, 73.16, 72.33, 71.24, 71.13, 70.35, 42.08, 34.55, 34.46, 34.36. HRMS m/z: $[M+H]^+$ calculated for $C_{28}H_{31}NO_3$ 430.2377, found 430.2374.

Compound **38**

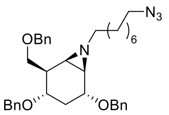

Compound **37** (0.290 g, 0.61 mmol) was treated according to General procedure F. Purification by silica gel column chromatography (pentane/EA, 15:1 → 10:1) yielded compound **38** (0.269 g, 83%).

^1H NMR (500 MHz, $CDCl_3$) δ 7.44 – 7.22 (m, 15H), 4.71 – 4.63 (m, 2H), 4.60 – 4.51 (m, 3H), 4.31 (d, *J* = 11.4 Hz, 1H), 3.95 – 3.88 (m, 1H), 3.74 – 3.67 (m, 1H), 3.56 – 3.49 (m, 1H), 3.25 (t, *J* = 6.9 Hz, 2H), 3.07 (ddt, *J* = 13.0, 11.3, 2.2 Hz, 1H), 2.46 (dt, *J* = 11.7, 7.6 Hz, 1H), 2.33 (ddd, *J* = 12.2, 6.1, 3.2 Hz, 1H), 2.22 – 2.14 (m, 1H), 2.06 (dd, *J* = 6.3, 3.5 Hz, 1H), 1.97 (dt, *J* = 11.7, 7.3 Hz, 1H), 1.76 (d, *J* = 6.2 Hz, 1H), 1.63 – 1.50 (m, 4H), 1.41 – 1.24 (m, 9H). ^{13}C NMR (126 MHz, $CDCl_3$) δ 138.66, 138.56, 138.34, 128.53 (2C), 128.41 (2C), 128.38 (2C), 127.82 (2C), 127.76 (5C), 127.62, 127.56, 74.96, 73.42, 73.24, 71.20, 71.09, 70.56, 61.33, 51.51, 43.33, 43.17, 42.16, 34.55, 29.48, 29.37, 29.17, 28.88, 27.34, 26.74. HRMS m/z: $[M+H]^+$ calculated for $C_{36}H_{46}N_4O_3$ 583.3643, found 583.3640.

Compound **39**

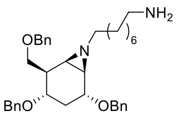

Compound **38** (0.240 g, 0.56 mmol) was treated according to General procedure G to yield compound **39** (0.071 g, quant.).

^1H NMR (500 MHz, $CDCl_3$) δ 7.41 – 7.23 (m, 15H), 4.70 – 4.61 (m, 2H), 4.58 – 4.49 (m, 3H), 4.29 (d, *J* = 11.4 Hz, 1H), 3.90 (dd, *J* = 8.5, 3.6 Hz, 1H), 3.69 (dd, *J* = 10.8, 6.0 Hz, 1H), 3.55 – 3.47 (m, 1H), 3.06 (ddd, *J* = 12.1, 9.6, 3.0 Hz, 1H), 2.71 – 2.63 (m, 2H), 2.44 (dt, *J* = 11.5, 7.7 Hz, 1H), 2.31 (ddd, *J* = 12.0, 6.0, 3.1 Hz, 1H), 2.16 (tt, *J* = 9.7, 3.6 Hz, 1H), 2.04 (dd, *J* = 6.2, 3.5 Hz, 1H), 1.96 (dt, *J* = 11.5, 7.3 Hz, 1H), 1.74 (d, *J* = 6.2 Hz, 1H), 1.58 (bs, 2H), 1.55 – 1.47 (m, 2H), 1.47 – 1.39 (m, 2H), 1.39 – 1.23 (m, 9H). ^{13}C NMR (126 MHz, $CDCl_3$) δ 138.63, 138.54, 138.32, 128.51 (2C), 128.39 (2C), 128.36 (2C), 127.81 (2C), 127.78 (2C), 127.74 (3C), 127.60, 127.53, 74.95, 73.42, 73.23, 71.18, 71.07, 70.54, 61.37, 43.29, 43.14, 42.23, 42.15, 34.54, 33.72, 29.63, 29.51, 29.41, 27.41, 26.91. HRMS m/z: $[M+H]^+$ calculated for $C_{36}H_{48}N_2O_3$ 557.3738, found 557.3736.

Compound **40**

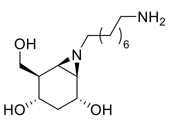

Compound **39** (0.040 g, 0.072 mmol) was treated according to General procedure H to yield pure compound **40** (0.012 g, 58%) as well as impure fractions (0.010 g).

^1H NMR (500 MHz, MeOD) δ 3.95 (dd, *J* = 10.1, 5.2 Hz, 1H), 3.86 (dd, *J* = 10.1, 5.9 Hz, 1H), 3.62 (dd, *J* = 10.1, 8.5 Hz, 1H), 3.28 (ddd, *J* = 11.6, 8.8, 3.1 Hz, 1H), 2.68 – 2.61 (m, 2H), 2.36 (dt, *J* = 11.7, 7.8 Hz, 1H), 2.13 (dt, *J* = 11.7, 7.0 Hz, 1H), 2.01 (dd, *J* = 6.3, 3.8 Hz, 1H), 1.98 – 1.92 (m, 1H), 1.79 (tdd, *J* = 8.7, 5.2, 3.8 Hz, 1H), 1.68 (d, *J* = 6.3 Hz, 1H), 1.61 – 1.43 (m, 4H), 1.39 – 1.25 (m, 9H). ^{13}C NMR (126 MHz, MeOD) δ 68.10, 66.65, 63.82, 62.27, 46.61, 46.41, 43.78, 42.38, 41.26, 33.29, 30.57, 30.49, 30.32, 28.35, 27.92.

Compound **41**

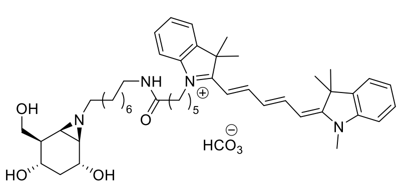

Compound **40** (0.005 g, 0.017 mmol) was treated according to General procedure I to yield compound **41** (0.0045 g, 32%).

^{1}H NMR (850 MHz, MeOD) δ 8.29 – 8.22 (m, 2H), 7.52 – 7.48 (m, 2H), 7.44 – 7.39 (m, 2H), 7.32 – 7.24 (m, 4H), 6.63 (t, *J* = 12.3 Hz, 1H), 6.31 – 6.25 (m, 2H), 4.11 (t, *J* = 7.5 Hz, 2H), 3.94 (dd, *J* = 10.1, 5.1 Hz, 1H), 3.84 (dd, *J* = 10.1, 5.8 Hz, 1H), 3.63 (s, 3H), 3.60 (dd, *J* = 10.2, 8.5 Hz, 1H), 3.26 (ddd, *J* = 11.7, 8.8, 3.0 Hz, 1H), 3.12 (t, *J* = 7.2 Hz, 2H), 2.36 – 2.31 (m, 1H), 2.21 – 2.17 (m, 2H), 2.10 (ddd, *J* = 11.7, 8.4, 6.4 Hz, 1H), 1.99 (dd, *J* = 6.3, 3.8 Hz, 1H), 1.96 – 1.92 (m, 1H), 1.85 – 1.81 (m, 2H), 1.79 (tdd, *J* = 8.8, 5.1, 3.8 Hz, 1H), 1.73 (s, 9H), 1.71 – 1.66 (m, 3H), 1.57 – 1.52 (m, 2H), 1.48 – 1.43 (m, 4H), 1.36 – 1.26 (m, 9H). ^{13}C NMR (214 MHz, MeOD) δ 175.69, 175.37, 174.68, 155.56, 155.53, 144.25, 143.58, 142.65, 142.51, 129.77, 129.75, 126.62, 126.28, 126.24, 123.43, 123.30, 112.05, 111.84, 104.36, 104.29, 68.11, 66.60, 63.79, 62.25, 50.55, 50.51, 46.63, 46.44, 44.77, 43.81, 41.32, 40.38, 36.69, 31.50, 30.59, 30.38, 30.31, 28.35, 28.20, 27.96, 27.94, 27.80, 27.37, 26.57. HRMS m/z: [M]$^+$ calculated for $C_{47}H_{67}N_4O_4$ 751.5157, found 751.5157.

Compound **42**

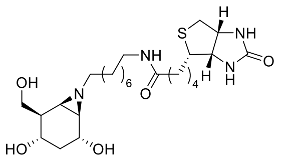

Compound **40** (0.0058 g, 0.020 mmol) was treated according to General procedure J to yield compound **42** (0.0039 g, 37%).

^{1}H NMR (600 MHz, MeOD) δ 4.62 (bs, 1H), 4.49 (ddd, *J* = 7.9, 5.0, 0.9 Hz, 1H), 4.30 (dd, *J* = 7.9, 4.5 Hz, 1H), 3.95 (dd, *J* = 10.1, 5.1 Hz, 1H), 3.86 (dd, *J* = 10.1, 5.9 Hz, 1H), 3.61 (dd, *J* = 10.1, 8.5 Hz, 1H), 3.27 (ddd, *J* = 11.6, 8.8, 3.1 Hz, 1H), 3.23 – 3.12 (m, 3H), 2.93 (dd, *J* = 12.8, 5.0 Hz, 1H), 2.71 (d, *J* = 12.7 Hz, 1H), 2.36 (dt, *J* = 11.7, 7.5 Hz, 1H), 2.19 (td, *J* = 7.3, 1.1 Hz, 2H), 2.16 – 2.10 (m, 1H), 2.01 (dd, *J* = 6.3, 3.8 Hz, 1H), 1.95 (ddd, *J* = 12.1, 5.8, 3.3 Hz, 1H), 1.82 – 1.53 (m, 8H), 1.53 – 1.40 (m, 4H), 1.39 – 1.25 (m, 9H). ^{13}C NMR (151 MHz, MeOD) δ 175.97, 166.12, 68.09, 66.61, 63.79, 63.39, 62.27, 61.62, 57.03, 46.64, 46.43, 43.82, 41.31, 41.05, 40.35, 36.83, 30.58, 30.41, 30.34, 30.32, 29.79, 29.52, 28.35, 27.94, 26.96. HRMS m/z: [M+H]$^+$ calculated for $C_{25}H_{44}N_4O_5S$ 513.3105, found 513.3106.

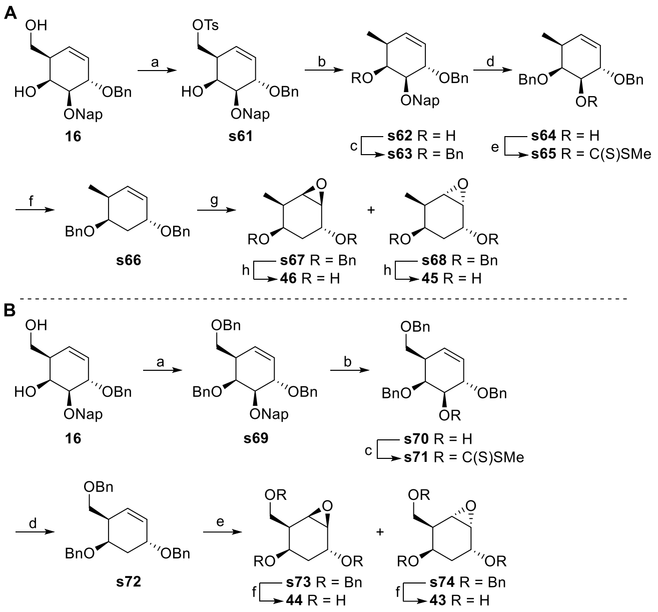

Scheme S1. (A) Synthesis of *galactose*-configured 3,6-dideoxycyclophellitols. Reagents and conditions: a) i. TsCl, TEA, DCM, 0 °C → r.t., 93%. b) $LiAlH_4$, THF, 0 °C, quant. c) BnBr, NaH, TBAI, DMF, 0 °C → r.t., 99%. d) DDQ, β-pinene, DCM/H_2O, 0 °C → r.t., 76%. e) *i*-Pr_2NH, *n*-BuLi, **s64**, CS_2, MeI, THF, -78 °C → -10 °C, 95%. f) Bu_3SnH, AIBN, toluene, 100 °C, 70%. g) *m*-CPBA, DCM, **s67**: 68%, **s68**: 22%, or Oxone®, $NaHCO_3$, Na_2EDTA, $AcCF_3$, MeCN, 0 °C, **s67**: 30%, **s68**: 52%. h) $Pd(OH)_2/C$, H_2 1 atm., 1,4-dioxane, Milli-Q, MeOH, 48% for **46**, 24% for **45**. (B) Synthesis of *galactose*-configured 3-deoxycyclophellitols. Reagents and conditions: a) BnBr, NaH, TBAI, DMF, 0 °C → r.t., 84%. b) DDQ, DCM/H_2O, 0 °C → r.t., 79%. c) *i*-Pr_2NH, *n*-BuLi, **s70**, CS_2, MeI, THF, -78 °C → -10 °C, 97%. d) Bu_3SnH, AIBN, toluene, 100 °C, 64%. e) *m*-CPBA, DCM, **s73**: 45%, **s74**: 16%, or Oxone®, $NaHCO_3$, Na_2EDTA, $AcCF_3$, MeCN, 0 °C, **s73**: 26%, **s74**: 56%. f) $Pd(OH)_2/C$, H_2 1 atm., 1,4-dioxane, Milli-Q, 62% for **44**, 44% for **43**.

Compound **s61**

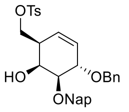

Diol **16** (1.29 g, 3.30 mmol) was dissolved in 22 mL of DCM. Then freshly purified *p*-toluenesulfonyl chloride (0.79 g, 4.13 mmol) and TEA (1.15 mL, 8.26 mmol) were added and the reaction mixture was stirred overnight. Afterwards, the mixture was diluted with DCM and washed with HCl (aq., 1 M), the aqueous layer was extracted with DCM and the combined organic layers were washed with $NaHCO_3$ (aq., sat.) and brine, then dried over anhydrous $MgSO_4$ and filtered, after which the filtrate was concentrated and the product purified by silica gel column chromatography (pentane/EtOAc, 20:1 → 3.5:1) to afford compound **s61** as a yellow oil (1.68 g, 93%).

^{1}H NMR (400 MHz, $CDCl_3$) δ 7.93 – 7.76 (m, 6H), 7.58 – 7.48 (m, 3H), 7.44 – 7.27 (m, 7H), 5.85 (dt, *J* = 10.2, 2.7 Hz, 1H), 5.46 – 5.38 (m, 1H), 4.91 – 4.80 (m, 2H), 4.80 – 4.69 (m, 2H), 4.37 (dt, *J* = 7.5, 2.5 Hz, 1H), 4.32 – 4.22 (m, 2H), 4.11 (dd, *J* = 9.5, 7.1 Hz, 1H), 3.73 – 3.66 (m, 1H), 2.74 (d, *J* = 12.9 Hz, 2H), 2.41 (s, 3H). ^{13}C NMR (101 MHz, $CDCl_3$) δ 144.80, 138.43, 135.40, 133.15, 132.93, 132.61, 129.81 (2C), 128.32 (3C), 128.18, 127.86, 127.82 (2C), 127.75 (2C), 127.65, 127.56, 126.52, 126.14, 125.95, 125.77, 123.67, 81.53, 76.54, 72.16, 72.04, 69.86, 66.80, 40.20, 21.51.

Compound **s62**

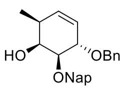

Tosylate **s61** (1.68 g, 3.06 mmol) was dissolved in 31 mL of THF, then the solution was cooled to 0 °C and lithium aluminium hydride solution (4.6 mL, 4.61 mmol, 1 M) was added portion wise. The reaction mixture was stirred for 2.5 hours at 0 °C after which TLC analysis indicated full conversion of starting material. The reaction was quenched with NH_4Cl (aq., sat.) and diluted with Et_2O, washed with HCl (aq., 1 M), the aqueous layer was extracted with Et_2O. The combined organic layers were washed with $NaHCO_3$ (aq., sat.), brine, dried over anhydrous $MgSO_4$ and filtered, after which the filtrate was concentrated and the product purified by silica gel column chromatography (pentane/EtOAc, 5:1) to afford compound **s62** as a yellow oil (1.13 g, quant.)

^{1}H NMR (300 MHz, $CDCl_3$) δ 7.75 (dtd, *J* = 12.5, 6.0, 3.5 Hz, 4H), 7.48 – 7.38 (m, 3H), 7.38 – 7.19 (m, 5H), 5.69 (dt, *J* = 10.1, 2.6 Hz, 1H), 5.44 – 5.35 (m, 1H), 4.82 (s, 2H), 4.69 (s, 2H), 4.34 (ddt, *J* = 7.5, 3.0, 2.1 Hz, 1H), 4.05 – 3.98 (m, 1H), 3.67 (dd, *J* = 7.8, 2.1 Hz, 1H), 2.50 (s, 1H), 2.41 – 2.27 (m, 1H), 1.09 (d, *J* = 7.4 Hz, 3H). ^{13}C NMR (75 MHz, $CDCl_3$) δ 138.75, 135.78, 133.24, 132.98, 131.19, 128.34 (2C), 128.19, 127.90, 127.80 (2C), 127.69, 127.52, 126.51, 126.11, 125.91, 125.85, 125.16, 82.55, 77.01, 72.08, 72.01, 70.81, 34.98, 16.26. HRMS m/z: $[M+Na]^+$ calculated for $C_{25}H_{26}O_3$ 397.1774, found 397.1771.

Compound **s63**

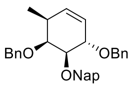

Alcohol **s62** (1.05 g, 2.8 mmol) was dissolved in 14 mL of DMF, then benzyl bromide (0.40 mL, 3.36 mmol) and tetrabutylammonium iodide (0.010 g, 0.028 mmol) were added. The reaction mixture was cooled to 0 °C and sodium hydride (60% dispersion in mineral oil, 0.135 g, 3.36 mmol) was added portion wise and the mixture was allowed to warm up to room temperature and stir overnight. Afterwards, it was re-cooled to 0 °C, quenched with water, diluted with Et_2O, washed with water, brine, then dried over anhydrous $MgSO_4$ and filtered, after which the filtrate was concentrated and the product purified by silica gel column chromatography (pentane/Et_2O, 20:1 → 6.5:1) to afford compound **s63** as yellow oil (1.24 g, 99%).

^{1}H NMR (400 MHz, $CDCl_3$) δ 7.82 – 7.74 (m, 3H), 7.74 – 7.67 (m, 1H), 7.48 (dd, *J* = 8.4, 1.7 Hz, 1H), 7.45 – 7.37 (m, 2H), 7.34 (ddt, *J* = 8.1, 5.4, 2.1 Hz, 4H), 7.31 – 7.19 (m, 6H), 5.71 (dt, *J* = 10.1, 2.7 Hz, 1H), 5.44 (d, *J* = 10.1 Hz, 1H), 4.95 – 4.83 (m, 3H), 4.75 – 4.64 (m, 3H), 4.50 – 4.43 (m, 1H), 3.86 (dt, *J* = 3.4, 1.5 Hz, 1H), 3.78 (dd, *J* = 7.6, 1.8 Hz, 1H), 2.45 – 2.35 (m, 1H), 1.06 (d, *J* = 7.3 Hz, 3H). ^{13}C NMR (101 MHz, $CDCl_3$) δ 139.05, 138.88, 136.36, 133.30, 132.90, 132.00, 128.33 (2C), 128.21 (2C), 128.06, 128.03 (2C), 127.90, 127.79 (2C), 127.69, 127.47, 127.44, 126.15, 126.05, 125.78, 125.73, 125.40, 83.06, 77.93, 77.53, 73.79, 72.35, 72.14, 35.77, 16.63.

Compound **s64**

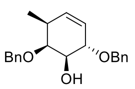

Compound **s63** (1.54 g, 3.36 mmol) was dissolved in 60.5 mL of DCM, then water (6.7 mL) and β-pinene (1.75 mL, 11.2 mmol) were added. The reaction mixture was cooled to 0 °C and DDQ (1.13 g, 4.96 mmol) was added slowly and portion wise over 10 minutes under vigorous stirring. After the last addition of DDQ, the reaction mixture was allowed to warm up to room temperature and stirred for 6.5 hours after which TLC analysis indicated full conversion of starting material. The reaction mixture was diluted with DCM and washed with $NaHCO_3$ (aq., sat.) until the water layer did not color upon extraction, then washed with brine, dried over anhydrous $MgSO_4$ and filtered, after which the filtrate was concentrated and the product purified by silica gel column chromatography (Pentane/Et_2O, 20:1 → 3:1) to afford compound **s64** as yellow oil (0.83 g, 76%).

^{1}H NMR (400 MHz, $CDCl_3$) δ 7.41 – 7.26 (m, 10H), 5.76 (dd, *J* = 9.9, 2.5 Hz, 1H), 5.50 (d, *J* = 12.0 Hz, 1H), 4.83 (d, *J* = 11.7 Hz, 1H), 4.73 (d, *J* = 11.7 Hz, 2H), 4.64 (s, 1H), 4.30 – 4.25 (m, 1H), 3.94 – 3.89 (m, 1H), 3.86 – 3.82 (m, 1H), 2.58 – 2.48 (m, 1H), 2.31 (d, *J* = 3.8 Hz, 1H), 1.11 (d, *J* = 7.4 Hz, 3H). ^{13}C NMR (101 MHz, $CDCl_3$) δ 139.04, 138.61, 132.58, 128.62 (2C),

128.46 (2C), 128.05 (2C), 128.04 (2C), 127.88, 127.70, 124.35, 80.16, 78.34, 75.29, 74.59, 71.49, 35.84, 16.67. HRMS m/z: $[M+Na]^+$ calculated for $C_{21}H_{24}O_3$ 347.1618, found 347.1616.

Compound **s65**

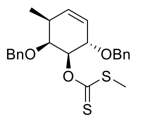

Diisopropylamine (1.56 mL, 11.10 mmol) was dissolved in 50 mL of THF, the solution was cooled to -78 °C and *n*-butyllithium solution (4.2 mL, 10.58 mmol, 2.5 M) was added dropwise and stirred for 15 minutes. Alcohol **s64** (1.64 g, 5.04 mmol) was added dropwise as a solution in 5 mL of THF and the mixture was stirred at -78 °C for another 10 minutes. Carbon disulfide (5.2 mL, 86 mmol) was added and the mixture was stirred for 1 hour at -78 °C. Next, iodomethane (2.5 mL, 40.3 mmol) was added to the reaction mixture and the reaction was allowed to warm up to -10 °C over 4 hours, after which TLC indicated near full conversion of starting material. The reaction was quenched with $NaHCO_3$ (aq., sat.), diluted with Et_2O and washed with water, brine, dried over anhydrous $MgSO_4$ and filtered, after which the filtrate was concentrated and the product purified by silica gel column chromatography (pentane/Et_2O, 67:1 → 12.5:1) to yield compound **s65** as yellow oil (1.99 g, 95%).

^{1}H NMR (300 MHz, $CDCl_3$) δ 7.36 – 7.21 (m, 10H), 5.97 (dd, *J* = 7.9, 2.0 Hz, 1H), 5.74 – 5.67 (m, 1H), 5.52 – 5.45 (m, 1H), 4.73 – 4.64 (m, 3H), 4.64 – 4.57 (m, 1H), 4.49 (d, *J* = 11.5 Hz, 1H), 4.11 – 4.06 (m, 1H), 2.64 – 2.56 (m, 1H), 2.54 (s, 3H), 1.05 (d, *J* = 7.3 Hz, 3H). ^{13}C NMR (75 MHz, $CDCl_3$) δ 215.57, 138.41, 138.28, 132.28, 128.36 (2C), 128.31 (2C), 128.04 (2C), 127.82 (2C), 127.67, 127.60, 125.05, 86.16, 77.68, 74.52, 74.38, 71.77, 35.36, 19.20, 16.32.

Compound **s66**

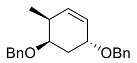

Xanthate **s65** (2.00 g, 4.80 mmol) was dissolved in 48 mL of toluene and the solution was purged with nitrogen gas flow over 10 minutes. Tributyltin hydride (3.9 mL, 14.46 mmol) was added to the solution and heated to 70 °C, followed by the addition of azobisisobutyronitrile solution in toluene (2.4 mL, 0.48 mmol, 0.2 M). The reaction mixture was heated further to 100 °C and stirred at this temperature for 2 hours, after which TLC indicated full conversion of starting material. The reaction mixture was concentrated and purified over two silica gel chromatography columns (pentane/Et_2O, 1:0 → 33:1, then pentane/$CHCl_3$, 3:2) to obtain compound **s66** as yellow oil (1.04 g, 70%).

^{1}H NMR (400 MHz, $CDCl_3$) δ 7.38 – 7.23 (m, 10H), 5.82 – 5.70 (m, 2H), 4.62 – 4.49 (m, 4H), 4.12 – 4.05 (m, 1H), 3.90 (ddd, *J* = 9.7, 4.9, 3.0 Hz, 1H), 2.57 (m, 1H), 2.02 (ddd, *J* = 13.4, 9.6, 4.8 Hz, 1H), 1.91 (ddd, *J* = 13.5, 4.9, 3.0 Hz, 1H), 1.02 (d, *J* = 7.2 Hz, 3H). ^{13}C NMR (101 MHz, $CDCl_3$) δ 138.92, 138.90, 134.75, 128.46 (2C), 128.43 (2C), 127.79 (2C), 127.76 (2C), 127.61, 127.58, 125.82, 74.25, 72.28, 70.66, 70.57, 33.74, 30.26, 14.22. HRMS m/z: $[M+NH_4]^+$ calculated for $C_{21}H_{24}O_2$ 326.2115, found 326.2110.

Compounds **s67** and **s68**

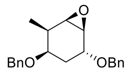

s67

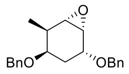

s68

Method A: Alkene **s66** (0.311 g, 1.00 mmol) was treated according to General procedure A. Purification by silica gel column chromatography (pentane/Et_2O, 13:1) afforded titled compounds (**s67**: 0.233 g, 68%; **s68**: 0.075 g, 22%).

Method B: Alkene **s66** (0.066 g, 0.210 mmol) was treated according to General procedure B. Purification by silica gel column chromatography (pentane/Et_2O, 13:1) afforded titled compounds (**s67**: 0.022 g, 30%; **s68**: 0.038 g, 52%).

s67: ^{1}H NMR (400 MHz, $CDCl_3$) δ 7.38 – 7.24 (m, 10H), 4.63 (d, *J* = 11.6 Hz, 1H), 4.57 – 4.39 (m, 3H), 4.11 – 4.05 (m, 1H), 3.76 (ddd, *J* = 10.9, 6.4, 3.2 Hz, 1H), 3.18 (dd, *J* = 3.5, 1.2 Hz, 2H), 2.52 – 2.40 (m, 1H), 1.77 (ddd, *J* = 13.7, 10.9, 3.6 Hz, 1H), 1.72 – 1.64 (m, 1H), 1.11 (d, *J* = 7.1 Hz, 3H). ^{13}C NMR (101 MHz, $CDCl_3$) δ 138.67, 138.15, 128.59 (2C), 128.46 (2C), 127.92, 127.87 (2C), 127.69 (2C), 127.64, 73.68, 71.93, 71.19, 70.58, 56.50, 54.29, 30.50, 26.89, 9.81. **s68**: ^{1}H NMR (400 MHz, $CDCl_3$) δ 7.43 – 7.22 (m, 10H), 4.74 – 4.63 (m, 2H), 4.47 (d, *J* = 11.7 Hz, 1H), 4.33 (d, *J* = 11.7 Hz, 1H), 4.08 (ddd, *J* = 9.7, 5.7, 2.3 Hz, 1H), 3.55 (ddt, *J* = 5.9, 4.2, 1.5 Hz, 1H), 3.38 (ddd, *J* = 3.8, 2.1, 0.8 Hz, 1H), 2.99 – 2.96 (m, 1H), 2.18 – 2.04 (m, 2H), 1.54 (ddd, *J* = 13.5, 9.7, 1.6 Hz, 1H), 1.11 (d, *J* = 7.6 Hz, 3H). ^{13}C NMR (101 MHz, $CDCl_3$) δ 138.59, 138.51, 128.54 (2C), 128.47 (2C), 127.96 (2C), 127.81 (3C), 127.76, 75.63, 71.26, 70.88, 70.71, 57.87, 53.47, 34.11, 27.57, 14.37. HRMS m/z: $[M+Na]^+$ calculated for $C_{21}H_{24}O_3$ 347.1618, found 347.1615.

Compound **46**

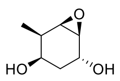

Epoxide **s67** (0.027 g, 0.082 mmol) was treated according to General procedure C. Purification by silica gel column chromatography (DCM/MeOH, 30:1) yielded contaminated compound **46**. The product was taken up in MeCN, washed three times with pentane and concentrated to afford pure compound **46** (0.0055 g, 48%).

^{1}H NMR (500 MHz, MeOD) δ 4.22 – 4.18 (m, 1H), 3.93 – 3.88 (m, 1H), 3.21 – 3.16 (m, 1H), 3.10 (dd, *J* = 3.6, 1.5 Hz, 1H), 2.28 – 2.20 (m, 1H), 1.96 – 1.88 (m, 1H), 1.40 (m, 1H), 1.10 (d, *J* = 7.1 Hz, 3H). ^{13}C NMR (126 MHz, MeOD) δ 66.17, 65.77, 58.33, 57.52, 35.21, 34.13, 11.21.

Compound **45**

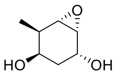

Epoxide **s68** (0.024 g, 0.070 mmol) was treated according to General procedure C. Purification by silica gel column chromatography (DCM/MeOH, 50:1 → 33:1) yielded contaminated compound **45**. The product was taken up in MeCN, washed three times with pentane and concentrated to afford pure compound **45** (0.0024 g, 24%).

^{1}H NMR (500 MHz, MeOD) δ 4.30 – 4.24 (m, 1H), 3.78 (td, *J* = 4.1, 2.0 Hz, 1H), 3.27 – 3.20 (m, 1H), 2.95 – 2.90 (m, 1H), 1.94 (m, 1H), 1.87 (dtd, *J* = 12.8, 5.9, 1.0 Hz, 1H), 1.44 (ddd, *J* = 13.1, 9.8, 1.8 Hz, 1H), 1.09 (d, *J* = 7.6 Hz, 3H). ^{13}C NMR (126 MHz, MeOD) δ 69.22, 65.30, 58.90, 56.43, 35.52, 34.98, 14.56.

Compound **s69**

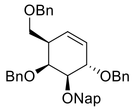

Diol **16** (0.58 g, 1.51 mmol) was dissolved in 7.5 mL of DMF, then benzyl bromide (0.43 mL, 3.63 mmol) and tetrabutylammonium iodide (0.006 g, 0.015 mmol) were added. The reaction mixture was cooled to 0 °C and sodium hydride (60% dispersion in mineral oil, 0.145 g, 3.63 mmol) was added portion wise and the mixture was allowed to warm up to room temperature and stirred overnight. Afterwards, it was re-cooled to 0 °C, quenched with water, diluted with Et_2O, washed with water, brine, dried over anhydrous $MgSO_4$ and filtered, after which the filtrate was concentrated and the product purified by silica gel column chromatography (pentane/Et_2O, 33:1 → 6.5:1) to afford compound **s69** as yellow oil (0.71 g, 84%).

^{1}H NMR (400 MHz, $CDCl_3$) δ 7.97 – 7.80 (m, 4H), 7.64 – 7.51 (m, 3H), 7.49 – 7.33 (m, 15H), 5.92 (d, *J* = 10.1 Hz, 1H), 5.61 (d, *J* = 9.8 Hz, 1H), 5.09 (d, *J* = 15.0 Hz, 1H), 5.05 – 4.93 (m, 2H), 4.92 – 4.76 (m, 3H), 4.74 – 4.51 (m, 3H), 4.35 (s, 1H), 3.92 (d, *J* = 10.3 Hz, 1H), 3.79 – 3.68 (m, 1H), 3.65 – 3.55 (m, 1H), 2.83 (d, *J* = 2.4 Hz, 1H). ^{13}C NMR (101 MHz, $CDCl_3$) δ 139.14, 138.89, 138.27, 136.28, 133.36, 132.97, 128.49, 128.40, 128.28, 128.12, 127.98, 127.96, 127.88, 127.75, 127.60, 127.56, 127.48, 126.97, 126.78, 126.20, 126.10, 125.84, 125.78, 83.14, 77.77, 75.26, 74.16, 73.36, 72.39, 72.26, 70.27, 42.03.

Compound **s70**

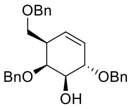

Compound **s69** (3.63 g, 6.36 mmol) was dissolved in 57.2 mL of DCM, then water (6.4 mL) was added, and the mixture was stirred vigorously. The reaction mixture was cooled to 0 °C and DDQ (1.73 g, 7.63 mmol) was added slowly and portion wise under vigorous stirring. After the last addition of DDQ, the reaction was allowed to warm up to room temperature and stirred for 1.5 hours after which TLC analysis indicated full conversion of starting material. The reaction mixture was diluted with DCM and washed with $NaHCO_3$ (aq., sat.) until the aqueous layer did not color upon extraction, then washed with brine, dried over anhydrous $MgSO_4$ and filtered, after which the filtrate was concentrated and the product purified by silica gel column chromatography (pentane/Et_2O, 10:1 → 5:1) to afford compound **s70** as yellow oil (2.17 g, 79%).

^{1}H NMR (400 MHz, $CDCl_3$) δ 7.37 – 7.24 (m, 15H), 5.87 – 5.81 (m, 1H), 5.63 – 5.58 (m, 1H), 4.80 (d, *J* = 11.8 Hz, 1H), 4.69 (d, *J* = 11.6 Hz, 1H), 4.62 (dd, *J* = 11.7, 3.8 Hz, 2H), 4.51 (d, *J* = 2.0 Hz, 2H), 4.18 (m, 1H), 4.14 – 4.10 (m, 1H), 3.96 (td, *J* = 6.2, 2.3 Hz, 1H), 3.62 (dd, *J* = 9.0, 5.2 Hz, 1H), 3.59 – 3.52 (m, 1H), 3.15 (d, *J* = 6.0 Hz, 1H), 2.74 (dq, *J* = 6.9, 2.6 Hz, 1H). ^{13}C NMR (101 MHz, $CDCl_3$) δ 138.97, 138.58, 137.87, 128.60 (2C), 128.56, 128.55 (2C), 128.47 (2C), 127.99 (2C), 127.89 (2C), 127.87 (3C), 127.67, 126.37, 78.30, 76.55, 73.44, 73.41, 72.39, 71.59, 69.01, 41.12.

Compound **s71**

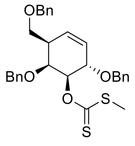

Diisopropylamine (1.4 mL, 10.0 mmol) was dissolved in 45 mL of THF, then the solution was cooled to -78°C and *n*-butyllithium solution (3.83 mL, 9.57 mmol, 2.5 M) was added dropwise and stirred for 15 minutes. Alcohol **s70** (1.96 g, 4.56 mmol) was added dropwise as a solution in 5 mL of THF and the mixture was stirred at -78 °C for another 10 minutes. Carbon disulfide (4.7 mL, 78 mmol) was added and the mixture was stirred for 1 hour at -78 °C. Next, iodomethane (2.3 mL, 36.5 mmol) was added and the reaction mixture was allowed to warm up to -10 °C over 4 hours, after which TLC indicated near full conversion of starting material. The reaction was quenched with $NaHCO_3$ (aq., sat.), diluted with Et_2O and washed with water, brine, dried over anhydrous $MgSO_4$ and filtered, after which the filtrate was concentrated and the product purified by silica gel column chromatography (pentane/Et_2O, 67:1 → 12.5:1) to yield compound **s71** as yellow oil (2.31 g, 97%).

^{1}H NMR (400 MHz, $CDCl_3$) δ 7.37 – 7.21 (m, 15H), 6.03 – 5.95 (m, 1H), 5.83 – 5.75 (m, 1H), 5.57 – 5.49 (m, 1H), 4.72 – 4.61 (m, 4H), 4.51 – 4.43 (m, 3H), 4.36 (s, 1H), 3.55 (t, *J* = 8.8 Hz, 1H), 3.51 – 3.43 (m, 1H), 2.86 (m, 1H), 2.55 (s, 3H). ^{13}C NMR (101 MHz, $CDCl_3$) δ 215.48, 138.46, 138.39, 138.14, 128.50 (2C), 128.44 (2C), 128.38 (2C), 127.95 (2C), 127.92 (2C), 127.84 (2C), 127.77, 127.70 (2C), 127.17, 127.13, 86.03, 75.41, 74.86, 74.63, 73.28, 71.91, 69.76, 41.44, 19.29. HRMS m/z: $[M+NH_4]^+$ calculated for $C_{30}H_{32}O_4S_2$ 538.2080, found 538.2080.

Compound **s72**

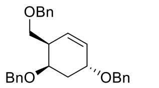

Xanthate **s71** (2.50 g, 4.80 mmol) was dissolved in 48 mL of toluene and the solution was purged with nitrogen gas flow over 10 minutes. Tributyltin hydride (3.9 mL, 14.46 mmol) was added and the solution was heated to 70 °C, followed by the addition of azobisisobutyronitrile solution in toluene (4.8 mL, 0.96 mmol, 0.2 M). The reaction mixture was heated further to 100 °C and stirred for 2 hours, after which TLC indicated full conversion of starting material. The reaction mixture was concentrated and purified over two silica gel chromatography columns (pentane/Et_2O, 1:0 → 33:1, then DCM/toluene, 13:1) to obtain compound **s72** as a yellow oil (1.27 g, 64%).

^{1}H NMR (400 MHz, $CDCl_3$) δ 7.38 – 7.24 (m, 15H), 5.96 – 5.87 (m, 1H), 5.75 – 5.68 (m, 1H), 4.60 – 4.43 (m, 6H), 4.26 – 4.16 (m, 1H), 4.08 – 3.97 (m, 1H), 3.67 (dd, *J* = 8.9, 7.5 Hz, 1H), 3.53 – 3.42 (m, 1H), 2.72 (m, 1H), 2.32 (dddd, *J* = 13.3, 6.6, 5.3, 1.0 Hz, 1H), 1.73 (ddd, *J* = 13.3, 7.9, 2.2 Hz, 1H). ^{13}C NMR (101 MHz, $CDCl_3$) δ 138.83, 138.80, 138.55, 128.86, 128.56, 128.51 (2C), 128.48 (2C), 128.44 (2C), 127.86 (2C), 127.84 (2C), 127.76 (2C), 127.68, 127.66, 127.66, 73.40, 73.38, 72.03, 71.19, 70.55, 70.51, 40.60, 31.74. HRMS m/z: $[M+Na]^+$ calculated for $C_{28}H_{30}O_3$ 437.2087, found 437.2091.

Compounds **s73** and **s74**

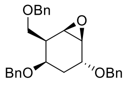
s73

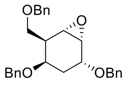
s74

Method A: Alkene **s72** (0.053 g, 0.130 mmol) was treated according to General procedure A. Purification by silica gel column chromatography (pentane/Et_2O, 20:1 → 5:1) afforded titled compounds (**s73**: 0.025 g, 45 %; **s74**: 0.009 g, 16 %).

Method B: Alkene **s72** (0.078 g, 0.190 mmol) was treated according to General procedure B. Purification by silica gel column chromatography (pentane/acetone, 30:1) afforded titled compounds (**s73**: 0.021 g, 26 %; **s74**: 0.045 g, 56 %).

s73: ^{1}H NMR (400 MHz, $CDCl_3$) δ 7.38 – 7.22 (m, 15H), 4.63 – 4.50 (m, 4H), 4.49 – 4.39 (m, 2H), 4.08 – 4.02 (m, 1H), 3.87 – 3.69 (m, 3H), 3.38 – 3.32 (m, 1H), 3.25 (dd, *J* = 3.6, 1.5 Hz, 1H), 2.67 – 2.58 (m, 1H), 1.86 (ddd, *J* = 13.6, 9.7, 4.0 Hz, 1H), 1.60 (ddd, *J* = 13.7, 5.8, 2.8 Hz, 1H). ^{13}C NMR (101 MHz, $CDCl_3$) δ 138.59, 138.45, 138.07, 128.63 (2C), 128.48 (4C), 127.98, 127.91 (2C), 127.83 (2C), 127.79 (2C), 127.73, 127.67, 73.64, 72.75, 71.94, 71.19, 70.35, 67.68, 54.26, 53.74, 37.59, 28.71. HRMS m/z: $[M+Na]^+$ calculated for $C_{28}H_{30}O_4$ 453.2036, found 453.2029. **s74**: ^{1}H NMR (400 MHz, $CDCl_3$) δ 7.43 – 7.27 (m, 13H), 7.21 – 7.17 (m, 2H), 4.75 – 4.64 (m, 2H), 4.58 – 4.49 (m, 2H), 4.45 (d, *J* = 11.5 Hz, 1H), 4.30 (d, *J* = 11.5 Hz, 1H), 4.11 (ddd, *J* = 10.6, 5.5, 2.0 Hz, 1H), 3.78 (td, *J* = 4.0, 1.7 Hz, 1H), 3.68 – 3.57 (m, 2H), 3.43 – 3.39 (m, 1H), 3.11 (dd, *J* = 4.1, 1.5 Hz, 1H), 2.28 (td, *J* = 7.8, 4.1 Hz, 1H), 2.16 (dtd, *J* = 13.5, 5.4, 1.1 Hz, 1H), 1.51 (ddd, *J* = 13.5, 10.6, 1.3 Hz, 1H). ^{13}C NMR (101 MHz, $CDCl_3$) δ 138.57, 138.31, 138.30, 128.55 (2C), 128.52 (2C), 128.49 (2C), 127.95 (2C), 127.85 (2C), 127.82 (2C), 127.78 (3C), 73.51, 73.37, 71.36, 70.90, 70.74, 68.99, 54.45, 53.43, 40.12, 27.10. HRMS m/z: $[M+Na]^+$ calculated for $C_{28}H_{30}O_4$ 453.2036, found 453.2032.

Compound **44**

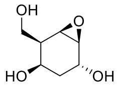

Epoxide **s73** (0.031 g, 0.073 mmol) was dissolved in 2 mL of 1,4-dioxane and 1 mL of Milli-Q water, before being purged with argon flow. Palladium hydroxide on carbon (0.010 g, 0.015 mmol, 20 wt. %) was added to the reaction mixture, which was then purged again with argon, then a constant flow of hydrogen gas was purged through the reaction solution for 6 hours. After TLC analysis indicated the full conversion of starting material and mono-/dibenzylated products, the reaction mixture was filtered through celite. The filtrate was concentrated and silica gel column chromatography (DCM/MeOH, 20:1 → 10:1) was performed to purify the product. The thus purified compound was taken up in MeCN and washed three times with pentane to remove residual grease. Compound **44** was obtained after the evaporation of MeCN and lyophilization (0.0072 mg, 62%).

^{1}H NMR (500 MHz, MeOD) δ 4.21 (t, *J* = 6.6 Hz, 1H), 4.05 (ddd, *J* = 8.3, 5.5, 2.8 Hz, 1H), 3.89 (dd, *J* = 10.7, 6.5 Hz, 1H), 3.78 (dd, *J* = 10.7, 8.0 Hz, 1H), 3.34 (t, *J* = 3.5 Hz, 1H), 3.14 (dd, *J* = 3.7, 1.1 Hz, 1H), 2.29 – 2.23 (m, 1H), 2.01 (ddd, *J* = 13.4, 8.4, 5.1 Hz, 1H), 1.42 (ddd, *J* = 13.4, 7.0, 2.4 Hz, 1H). ^{13}C NMR (126 MHz, MeOD) δ 65.21, 65.19, 61.19, 57.13, 55.26, 42.07, 36.55. HRMS m/z: $[M+Na]^+$ calculated for $C_7H_{12}O_4$ 183.0628, found 183.0629.

Compound **43**

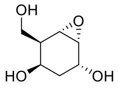

Epoxide **s74** (0.036 g, 0.085 mmol) was dissolved in 2 mL of 1,4-dioxane and 1 mL of Milli-Q water, before being purged with argon flow. Palladium hydroxide on carbon (0.012 g, 0.017 mmol, 20 wt. %) was added to the reaction mixture, which was then purged again with argon, then a constant flow of hydrogen gas was purged through the reaction solution for 6 hours. After TLC analysis indicated the full conversion of starting material and mono-/dibenzylated products, the reaction mixture was filtered through celite. The filtrate was concentrated and silica gel column chromatography (DCM/MeOH, 20:1 → 10:1) was performed to purify the product. The thus purified compound was taken up in MeCN and washed three times with pentane to remove residual grease. Compound **43** was obtained after the evaporation of MeCN and lyophilization (0.006 g, 44%).

^{1}H NMR (500 MHz, MeOD) δ 4.34 – 4.28 (m, 1H), 4.01 – 3.97 (m, 1H), 3.76 (dd, *J* = 11.0, 6.3 Hz, 1H), 3.69 (dd, *J* = 11.0, 7.9 Hz, 1H), 3.29 – 3.27 (m, 1H), 3.14 (dd, *J* = 4.1, 1.5 Hz, 1H), 1.93 – 1.86 (m, 2H), 1.42 (ddd, *J* = 13.0, 10.7, 1.6 Hz, 1H). ^{13}C NMR (126 MHz, MeOD) δ 67.91, 65.43, 62.68, 56.54, 55.63, 43.37, 34.94.

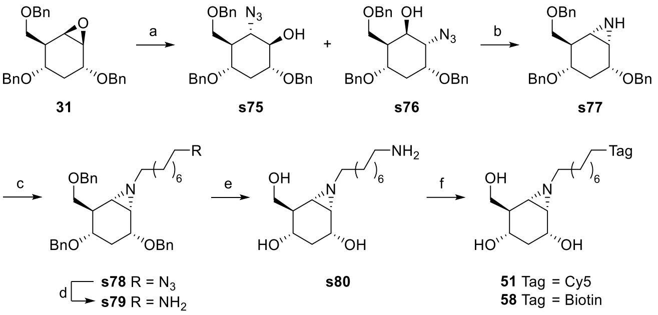

Scheme S2. Synthesis of *glucose*-configured 3-deoxy-α-aziridines. Reagents and conditions: a) NaN_3, $LiClO_4$, DMF, 95 °C, 86% in total. b) PPh_3 (beads), MeCN, 60 °C, 63%. c) 8-Azido-1-octanol, Py, Tf_2O, DCM, **s77**, DiPEA, 78%. d) PPh_3 (beads), H_2O, MeCN, 70 °C, quant. e) Li, NH_3(liq.), -70 °C → -55 °C, 82%. f) Cy5COOH, PFPOC(O)CF_3, DiPEA, DMF, **s80**, 24% for **51**, or biotin-OSu, DiPEA, 28% for **58**.

Compounds **s75** and **s76**

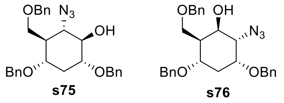

Epoxide **31** (0.370 g, 0.86 mmol) was treated according to General procedure D. Purification by silica gel column chromatography (pentane/DCM/Ac, 30:5:1) yielded partially separated titled compounds (0.350 g in total, 86%).

s75: ^{1}H NMR (500 MHz, $CDCl_3$) δ 7.41 – 7.26 (m, 15H), 4.71 (d, *J* = 11.6 Hz, 1H), 4.64 – 4.38 (m, 5H), 3.87 (dd, *J* = 9.2, 1.9 Hz, 1H), 3.65 (dd, *J* = 9.2, 2.4 Hz, 1H), 3.62 – 3.50 (m, 3H), 3.32 – 3.22 (m, 1H), 2.85 (s, 1H), 2.55 (dt, *J* = 12.1, 4.3 Hz, 1H), 1.52 (ddt, *J* = 10.7, 8.7, 2.2 Hz, 1H), 1.34 (q, *J* = 11.9 Hz, 1H). ^{13}C NMR (126 MHz, $CDCl_3$) δ 138.39, 138.25, 138.01, 128.73 (2C), 128.56 (2C), 128.50 (2C), 128.13, 127.97 (2C), 127.96 (2C), 127.93 (2C), 127.92, 127.81, 77.72, 77.39, 73.30, 71.99, 71.92, 71.70, 64.93, 62.09, 47.54, 33.75. HRMS m/z: $[M+Na]^+$ calculated for $C_{28}H_{31}N_3O_4$ 496.2207, found 496.2220. **s76**: ^{1}H NMR (500 MHz, $CDCl_3$) δ 7.43 – 7.27 (m, 15H), 4.70 – 4.61 (m, 3H), 4.54 (d, *J* = 11.8 Hz, 1H), 4.49 – 4.39 (m, 2H), 4.19 (dd, *J* = 3.4, 2.3 Hz, 1H), 4.15 (dd, *J* = 9.2, 3.2 Hz, 1H), 4.07 – 4.00 (m, 3H), 3.87 (td, *J* = 11.1, 4.4 Hz, 1H), 3.69 (dd, *J* = 9.2, 2.5 Hz, 1H), 2.43 (dtd, *J* = 11.7, 4.3, 1.5 Hz, 1H), 1.88 (dq, *J* = 11.1, 2.7 Hz, 1H), 1.85 – 1.75 (m, 1H). ^{13}C NMR (126 MHz, $CDCl_3$) δ 138.56, 138.24, 137.35, 128.66 (2C), 128.55 (2C), 128.48 (2C), 128.13, 127.86 (2C), 127.81 (2C), 127.78, 127.73, 127.59 (2C), 74.56, 73.84, 73.46, 71.21, 71.06, 71.00, 70.03, 62.11, 41.77, 32.12. HRMS m/z: $[M+Na]^+$ calculated for $C_{28}H_{31}N_3O_4$ 496.2207, found 496.2217.

Compound **s77**

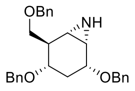

The mixture of compounds **s75** and **s76** (0.210 g, 0.443 mmol) was treated according to General procedure E. An additional amount of polymer-bound triphenylphosphine (0.8 eq, 3 mmol/g) was added and the mixture was stirred at 60 °C for additional 8 hours. Purification by silica gel column chromatography (DCM/Ac, 20:1 → 10:1) yielded compound **s77** (0.120 g, 63%).

^{1}H NMR (500 MHz, $CDCl_3$) δ 7.42 – 7.26 (m, 13H), 7.26 – 7.23 (m, 2H), 4.73 – 4.66 (m, 2H), 4.57 (d, *J* = 11.5 Hz, 1H), 4.54 – 4.42 (m, 2H), 4.29 (d, *J* = 11.5 Hz, 1H), 3.94 – 3.87 (m, 1H), 3.64 – 3.53 (m, 2H), 3.25 (ddd, *J* = 12.4, 9.6, 3.1 Hz, 1H), 2.51 – 2.44 (m, 1H), 2.31 (d, *J* = 6.0 Hz, 1H), 2.13 (ddd, *J* = 11.9, 5.3, 3.4 Hz, 1H), 2.04 (ddd, *J* = 9.2, 5.5, 3.4 Hz, 1H), 1.56 (q, *J* = 11.6 Hz, 1H). ^{13}C NMR (126 MHz, $CDCl_3$) δ 138.82, 138.52, 138.48, 128.51 (2C), 128.48 (2C), 128.46 (2C), 127.94 (2C), 127.88 (2C), 127.84 (2C), 127.75, 127.71, 127.69, 74.41, 74.00, 73.25, 70.57, 70.27, 70.06, 42.46, 32.97, 32.71, 28.67. HRMS m/z: $[M+H]^+$ calculated for $C_{28}H_{31}NO_3$ 430.2377, found 430.2373.

Compound **s78**

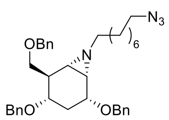

Compound **s77** (0.180 g, 0.419 mmol) was treated according to General procedure F. Purification by silica gel column chromatography (pentane/Ac, 12:1 → 8:1) yielded compound **s78** (0.190 g, 78%).

^{1}H NMR (500 MHz, $CDCl_3$) δ 7.42 – 7.20 (m, 15H), 4.68 (s, 2H), 4.57 – 4.49 (m, 2H), 4.44 (d, *J* = 12.2 Hz, 1H), 4.26 (d, *J* = 11.5 Hz, 1H), 3.76 (ddd, *J* = 11.3, 5.2, 3.1 Hz, 1H), 3.64 (dd, *J* = 9.2, 3.1 Hz, 1H), 3.55 (dd, *J* = 9.2, 5.9 Hz, 1H), 3.23 (t, *J* = 7.0 Hz, 2H), 3.14 (ddd, *J* = 12.6, 9.9, 3.1 Hz, 1H), 2.38 (dt, *J* = 11.5, 7.0 Hz, 1H), 2.10 (dq, *J* = 8.4, 5.9, 4.5 Hz, 2H), 2.01 (ddd, *J* = 9.6, 5.9, 3.1 Hz, 1H), 1.79 (dd, *J* = 6.4, 3.1 Hz, 1H), 1.64 – 1.51 (m, 6H), 1.39 – 1.25 (m, 8H). ^{13}C NMR (126 MHz, $CDCl_3$) δ 139.10, 138.67, 138.65, 128.45 (2C), 128.44 (2C), 128.42 (2C), 127.88 (2C), 127.77 (2C),

127.65 (3C), 127.63, 127.56, 74.95, 74.32, 73.13, 70.44, 70.25, 70.08, 61.15, 51.59, 42.50, 41.51, 41.26, 29.90, 29.82, 29.59, 29.17, 28.92, 27.39, 26.79. HRMS m/z: [M+H]+ calculated for $C_{36}H_{46}N_4O_3$ 583.3643, found 583.3641.

Compound **s79**

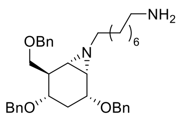

Compound **s78** (0.070 g, 0.120 mmol) was treated according to General procedure G to yield compound **s79** (0.067 g, quant.).

^{1}H NMR (400 MHz, $CDCl_3$) δ 7.43 – 7.21 (m, 15H), 4.69 (s, 2H), 4.58 – 4.51 (m, 2H), 4.44 (d, *J* = 12.2 Hz, 1H), 4.26 (d, *J* = 11.5 Hz, 1H), 3.77 (ddd, *J* = 11.3, 5.2, 3.1 Hz, 1H), 3.64 (dd, *J* = 9.2, 3.2 Hz, 1H), 3.56 (dd, *J* = 9.2, 5.9 Hz, 1H), 3.20 – 3.10 (m, 1H), 2.70 – 2.62 (m, 2H), 2.40 (dt, *J* = 11.4, 7.0 Hz, 1H), 2.11 (dt, *J* = 11.0, 7.3 Hz, 2H), 2.02 (ddd, *J* = 9.5, 5.9, 3.1 Hz, 1H), 1.85 – 1.68 (m, 3H), 1.66 – 1.51 (m, 4H), 1.46 – 1.38 (m, 2H), 1.37 – 1.25 (m, 8H). ^{13}C NMR (101 MHz, $CDCl_3$) δ 139.01, 138.58, 138.55, 128.39 (2C), 128.37 (2C), 128.35 (2C), 127.82 (2C), 127.71 (2C), 127.59 (3C), 127.56, 127.50, 74.83, 74.20, 73.04, 70.40, 70.12, 70.00, 61.15, 42.41, 42.19, 41.41, 41.20, 33.62, 29.84, 29.78, 29.67, 29.45, 27.39, 26.89.

Compound **s80**

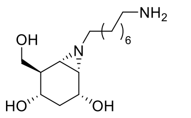

Compound **s79** (0.026 g, 0.047 mmol) was treated according to General procedure H to yield compound **s80** (0.011 g, 82 %).

^{1}H NMR (500 MHz, MeOD) δ 3.96 (ddd, *J* = 11.0, 5.6, 3.5 Hz, 1H), 3.83 (dd, *J* = 10.7, 4.3 Hz, 1H), 3.59 (dd, *J* = 10.7, 7.2 Hz, 1H), 3.25 – 3.18 (m, 1H), 2.66 – 2.61 (m, 2H), 2.33 (ddd, *J* = 11.6, 8.0, 6.6 Hz, 1H), 2.23 (ddd, *J* = 11.6, 8.2, 6.8 Hz, 1H), 1.80 – 1.73 (m, 2H), 1.70 – 1.64 (m, 2H), 1.64 – 1.55 (m, 2H), 1.54 – 1.42 (m, 3H), 1.42 – 1.31 (m, 8H). ^{13}C NMR (126 MHz, MeOD) δ 68.82, 67.94, 63.70, 62.10, 46.81, 45.39, 42.65, 42.45, 37.78, 33.48, 30.66, 30.52, 30.50, 28.43, 27.94. HRMS m/z: [M+H]+ calculated for $C_{15}H_{30}N_2O_3$ 287.2329, found 287.2328.

Compound **51**

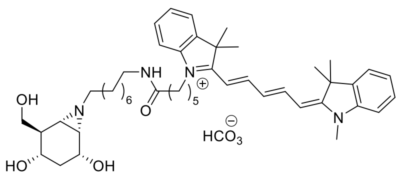

Compound **s80** (0.005 g, 0.017 mmol) was treated according to General procedure I to yield compound **51** (0.0035 g, 24%).

^{1}H NMR (850 MHz, MeOD) δ 8.29 – 8.22 (m, 2H), 7.52 – 7.48 (m, 2H), 7.45 – 7.38 (m, 2H), 7.33 – 7.24 (m, 4H), 6.63 (t, *J* = 12.4 Hz, 1H), 6.31 – 6.25 (m, 2H), 4.11 (t, *J* = 7.5 Hz, 2H), 3.96 (ddd, *J* = 11.0, 5.6, 3.5 Hz, 1H), 3.82 (dd, *J* = 10.7, 4.2 Hz, 1H), 3.63 (s, 3H), 3.58 (dd, *J* = 10.7, 7.2 Hz, 1H), 3.24 – 3.19 (m, 1H), 3.12 (t, *J* = 7.2 Hz, 2H), 2.30 (ddd, *J* = 11.7, 8.7, 6.1 Hz, 1H), 2.24 – 2.18 (m, 3H), 1.86 – 1.79 (m, 2H), 1.78 – 1.74 (m, 2H), 1.73 (s, 9H), 1.72 – 1.64 (m, 4H), 1.58 (dtd, *J* = 12.4, 8.9, 6.3 Hz, 2H), 1.52 – 1.42 (m, 5H), 1.39 – 1.26 (m, 8H). ^{13}C NMR (214 MHz, MeOD) δ 175.70, 175.36, 174.70, 155.54, 144.25, 143.58, 142.65, 142.51, 129.77, 129.75, 126.63, 126.28, 126.25, 123.43, 123.30, 112.05, 111.85, 104.36, 104.30, 68.73, 67.91, 67.90, 63.66, 62.07, 50.56, 50.50, 46.78, 45.39, 44.76, 42.64, 40.40, 37.78, 36.69, 31.48, 30.68, 30.49, 30.39, 30.34, 28.41, 28.20, 27.99, 27.94, 27.80, 27.36, 26.57. HRMS m/z: [M]+ calculated for $C_{47}H_{67}N_4O_4$ 751.5157, found 751.5162.

Compound **58**

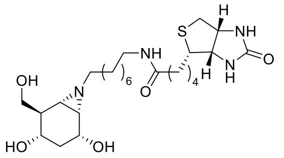

Compound **s80** (0.0045 g, 0.016 mmol) was treated according to General procedure J to yield compound **58** (0.0023 g, 28%).

^{1}H NMR (600 MHz, MeOD) δ 4.49 (ddd, *J* = 7.8, 5.0, 0.9 Hz, 1H), 4.30 (dd, *J* = 7.9, 4.5 Hz, 1H), 3.97 (ddd, *J* = 11.0, 5.5, 3.4 Hz, 1H), 3.83 (dd, *J* = 10.7, 4.2 Hz, 1H), 3.59 (dd, *J* = 10.7, 7.2 Hz, 1H), 3.25 – 3.11 (m, 4H), 2.93 (dd, *J* = 12.8, 5.0 Hz, 1H), 2.71 (d, *J* = 12.7 Hz, 1H), 2.32 (ddd, *J* = 11.6, 8.3, 6.4 Hz, 1H), 2.27 – 2.17 (m, 3H), 1.80 – 1.55 (m, 10H), 1.53 – 1.40 (m, 5H), 1.39 – 1.28 (m, 8H). ^{13}C NMR (151 MHz, MeOD) δ 175.97, 166.13, 68.75, 67.93, 63.66, 63.39, 62.09, 61.62, 57.04, 46.81, 45.40, 42.65, 41.06, 40.37, 37.78, 36.83, 30.66, 30.50, 30.43, 30.37, 29.80, 29.52, 28.42, 27.96, 26.97. HRMS m/z: [M+H]+ calculated for $C_{25}H_{44}N_4O_5S$ 513.3105, found 513.3108.

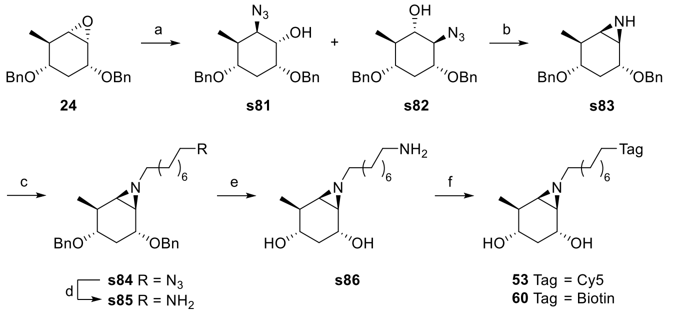

Scheme S3. Synthesis of *glucose*-configured 3,6-dideoxy-β-aziridines. Reagents and conditions: a) NaN_3, $LiClO_4$, DMF, 95 °C, 99% in total. b) PPh_3 (beads), MeCN, 60 °C. c) 8-Azido-1-octanol, Py, Tf_2O, DCM, **s83**, DiPEA, 77% over 2 steps. d) PPh_3 (beads), H_2O, MeCN, 70 °C, quant. e) Li, NH_3(liq.), -70 °C → -55 °C, quant. f) Cy5COOH, PFPOC(O)CF_3, DiPEA, DMF, **s86**, 28% for **53**, or biotin-OSu, DiPEA, 31% for **60**.

Compounds **s81** and **s82**

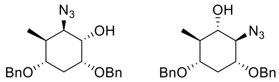

s81 s82

Epoxide **24** (0.120 g, 0.370 mmol) was treated according to General procedure D. Purification by silica gel column chromatography (pentane/EtOAc, 35:1 → 30:1) and (pentane/Ac, 30:1 → 20:1) yielded partially separated titled compounds (0.135 g in total, 99%).

s81: ^{1}H NMR (500 MHz, $CDCl_3$) δ 7.48 – 7.29 (m, 10H), 4.69 – 4.55 (m, 3H), 4.47 (d, *J* = 11.5 Hz, 1H), 4.07 – 4.02 (m, 1H), 3.82 (t, *J* = 3.7 Hz, 1H), 3.68 (ddd, *J* = 11.6, 4.6, 2.9 Hz, 1H), 3.26 (td, *J* = 10.4, 4.1 Hz, 1H), 2.76 (d, *J* = 2.0 Hz, 1H), 2.30 – 2.16 (m, 2H), 1.75 (q, *J* = 11.5 Hz, 1H), 1.17 (d, *J* = 6.8 Hz, 3H). ^{13}C NMR (126 MHz, $CDCl_3$) δ 138.64, 137.88, 128.62 (2C), 128.43 (2C), 128.06, 127.86 (2C), 127.72 (2C), 127.65, 76.36, 74.42, 71.12, 70.94, 68.32, 66.48, 35.69, 30.62, 14.06. HRMS m/z: $[M+Na]^+$ calculated for $C_{21}H_{25}N_3O_3$ 390.1788, found 390.1797. **s82**: ^{1}H NMR (400 MHz, $CDCl_3$) δ 7.42 – 7.27 (m, 10H), 4.71 – 4.59 (m, 3H), 4.43 (d, *J* = 11.4 Hz, 1H), 3.40 – 3.26 (m, 2H), 3.00 – 2.84 (m, 2H), 2.57 (dt, *J* = 12.1, 4.1 Hz, 1H), 2.32 (d, *J* = 3.0 Hz, 1H), 1.71 – 1.60 (m, 1H), 1.49 – 1.39 (m, 1H), 1.15 (d, *J* = 6.3 Hz, 3H). ^{13}C NMR (101 MHz, $CDCl_3$) δ 138.21, 137.79, 128.65 (2C), 128.60 (2C), 128.07, 128.04 (2C), 127.96 (3C), 76.98, 76.42, 73.89, 71.86, 71.52, 71.41, 43.40, 34.85, 14.42. HRMS m/z: $[M+Na]^+$ calculated for $C_{21}H_{25}N_3O_3$ 390.1788, found 390.1794.

Compound **s84**

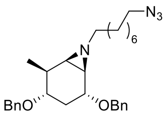

The mixture of compounds **s81** and **s82** (0.125 g, 0.340 mmol) was treated according to General procedure E. The reaction mixture was stirred additional 4 hours at 70 °C. Filtration of the reaction mixture resulted in 0.115 g of crystalline substance that was co-evaporated with toluene before being used directly in the next step. The thus obtained material was treated according to General procedure F. Purification by silica gel column chromatography (pentane/EtOAc, 50:1 → 15:1) yielded compound **s84** (0.125 g, 77% over 2 steps).

^{1}H NMR (500 MHz, $CDCl_3$) δ 7.42 – 7.24 (m, 10H), 4.71 – 4.62 (m, 2H), 4.59 (d, *J* = 11.3 Hz, 1H), 4.38 (d, *J* = 11.3 Hz, 1H), 3.73 (dd, *J* = 10.8, 5.9 Hz, 1H), 3.26 (t, *J* = 6.9 Hz, 2H), 3.00 (ddd, *J* = 12.1, 9.5, 2.9 Hz, 1H), 2.48 (ddd, *J* = 11.5, 9.1, 6.3 Hz, 1H), 2.33 (ddd, *J* = 11.8, 6.0, 2.9 Hz, 1H), 1.95 (ddd, *J* = 11.5, 9.1, 5.4 Hz, 1H), 1.88 – 1.80 (m, 1H), 1.71 – 1.64 (m, 2H), 1.64 – 1.57 (m, 2H), 1.57 – 1.46 (m, 2H), 1.42 – 1.27 (m, 10H), 1.22 (d, *J* = 6.5 Hz, 3H). ^{13}C NMR (126 MHz, $CDCl_3$) δ 138.80, 138.36, 128.49 (2C), 128.37 (2C), 127.80 (2C), 127.79 (2C), 127.72, 127.56, 77.45, 75.36, 71.57, 71.03, 61.44, 51.48, 46.96, 43.47, 36.72, 35.04, 29.75, 29.47, 29.13, 28.86, 27.31, 26.71, 15.70. HRMS m/z: $[M+H]^+$ calculated for $C_{29}H_{40}N_4O_2$ 477.3224, found 477.3220.

Compound **s85**

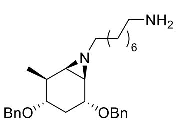

Compound **s84** (0.065 g, 0.136 mmol) was treated according to General procedure G to yield compound **s85** (0.062 g, quant.).

^{1}H NMR (400 MHz, $CDCl_3$) δ 7.40 – 7.22 (m, 10H), 4.68 – 4.53 (m, 3H), 4.36 (d, *J* = 11.3 Hz, 1H), 3.70 (dd, *J* = 10.9, 5.9 Hz, 1H), 2.98 (ddd, *J* = 12.1, 9.5, 2.9 Hz, 1H), 2.70 – 2.63 (m, 2H), 2.46 (ddd, *J* = 11.5, 9.1, 6.3 Hz, 1H), 2.30 (ddt, *J* = 12.0, 6.0, 3.0 Hz, 1H), 1.97 – 1.86 (m, 1H), 1.86 – 1.77 (m, 1H), 1.71 – 1.58 (m, 4H), 1.57 – 1.38 (m, 4H), 1.36 – 1.23 (m, 9H), 1.19 (d, *J* = 6.5 Hz, 3H). ^{13}C NMR (101 MHz, $CDCl_3$) δ 138.77, 138.33, 128.48 (2C), 128.36 (2C), 127.79 (4C), 127.71, 127.55, 77.45, 75.35, 71.56, 71.02, 61.48, 46.94, 43.44, 42.18, 36.70, 35.03, 33.64, 29.77, 29.60, 29.46, 27.38, 26.88, 15.68. HRMS m/z: $[M+H]^+$ calculated for $C_{29}H_{42}N_2O_2$ 451.3319, found 451.3316.

Compound **s86**

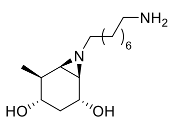

Compound **s85** (0.055 g, 0.122 mmol) was treated according to General procedure H to yield compound **s86** (0.033 g, quant.).

^{1}H NMR (400 MHz, MeOD) δ 3.86 (dd, *J* = 10.5, 5.9 Hz, 1H), 3.11 (ddd, *J* = 11.8, 9.0, 2.8 Hz, 1H), 2.67 – 2.60 (m, 1H), 2.45 (ddd, *J* = 11.8, 9.3, 6.4 Hz, 1H), 2.06 – 1.92 (m, 2H), 1.78 (dd, *J* = 6.3, 3.7 Hz, 1H), 1.67 – 1.42 (m, 6H), 1.42 – 1.19 (m, 10H), 1.17 (d, *J* = 6.6 Hz, 3H). ^{13}C NMR (101 MHz, MeOD) δ 69.72, 68.52, 62.33, 48.14, 47.33, 42.44, 42.08, 39.14, 33.47, 30.64, 30.61, 30.53, 28.34, 27.95, 15.77.

Compound **53**

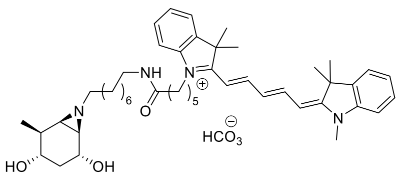

Compound **s86** (0.0067 g, 0.025 mmol) was treated according to General procedure I to yield compound **53** (0.0055 g, 28%).

^{1}H NMR (600 MHz, MeOD) δ 8.25 (ddd, *J* = 14.1, 12.4, 2.0 Hz, 2H), 7.50 (ddd, *J* = 7.4, 1.2, 0.6 Hz, 2H), 7.44 – 7.38 (m, 2H), 7.32 – 7.24 (m, 4H), 6.63 (t, *J* = 12.4 Hz, 1H), 6.28 (dd, *J* = 13.7, 6.4 Hz, 2H), 4.10 (t, *J* = 7.4 Hz, 2H), 3.85 (dd, *J* = 10.5, 5.9 Hz, 1H), 3.63 (s, 3H), 3.14 – 3.08 (m, 3H), 2.41 (ddd, *J* = 11.6, 9.4, 6.2 Hz, 1H), 2.20 (t, *J* = 7.3 Hz, 2H), 2.03 – 1.93 (m, 2H), 1.86 – 1.80 (m, 2H), 1.77 – 1.66 (m, 15H), 1.65 – 1.58 (m, 2H), 1.58 – 1.41 (m, 7H), 1.38 – 1.19 (m, 10H), 1.15 (d, *J* = 6.6 Hz, 3H). ^{13}C NMR (151 MHz, MeOD) δ 175.69, 175.38, 174.69, 155.54, 155.52, 144.25, 143.57, 142.65, 142.51, 129.77, 129.75, 126.62, 126.29, 126.24, 123.43, 123.29, 112.04, 111.84, 104.36, 104.29, 69.73, 68.53, 62.30, 50.55, 50.51, 48.10, 47.31, 44.77, 42.07, 40.38, 39.12, 36.69, 31.49, 30.63, 30.60, 30.39, 30.32, 28.32, 28.19, 27.97, 27.95, 27.80, 27.37, 26.56, 15.80. HRMS m/z: [M]$^{+}$ calculated for $C_{47}H_{67}N_4O_3$ 735.5208, found 735.5210.

Compound **60**

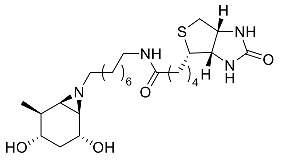

Compound **s86** (0.0038 g, 0.014 mmol) was treated according to General procedure J to yield compound **60** (0.0022 g, 31%).

^{1}H NMR (600 MHz, MeOD) δ 4.49 (ddd, *J* = 7.9, 5.0, 0.9 Hz, 1H), 4.30 (dd, *J* = 7.9, 4.4 Hz, 1H), 3.86 (dd, *J* = 10.5, 5.9 Hz, 1H), 3.23 – 3.08 (m, 4H), 2.93 (dd, *J* = 12.8, 5.0 Hz, 1H), 2.71 (d, *J* = 12.7 Hz, 1H), 2.44 (ddd, *J* = 11.6, 9.4, 6.2 Hz, 1H), 2.19 (td, *J* = 7.3, 1.2 Hz, 2H), 2.03 (ddd, *J* = 11.7, 9.4, 5.5 Hz, 1H), 1.99 – 1.93 (m, 1H), 1.78 (dd, *J* = 6.3, 3.7 Hz, 1H), 1.76 – 1.40 (m, 12H), 1.39 – 1.28 (m, 8H), 1.24 (td, *J* = 11.8, 10.5 Hz, 1H), 1.16 (d, *J* = 6.6 Hz, 3H). ^{13}C NMR (151 MHz, MeOD) δ 175.97, 166.12, 69.74, 68.53, 63.39, 62.33, 61.62, 57.03, 48.13, 47.32, 42.06, 41.05, 40.36, 39.14, 36.83, 30.61, 30.60, 30.42, 30.35, 29.79, 29.52, 28.33, 27.95, 26.96, 15.79. HRMS m/z: [M+H]$^{+}$ calculated for $C_{25}H_{44}N_4O_4S$ 497.3156, found 497.3154.

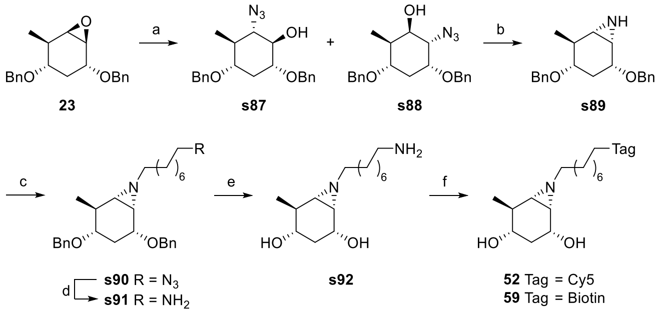

Scheme S4. Synthesis of *glucose*-configured 3,6-dideoxy-α-aziridines. Reagents and conditions: a) NaN_3, $LiClO_4$, DMF, 95 °C, quant. b) PPh_3 (beads), MeCN, 60 °C. c) 8-Azido-1-octanol, Py, Tf_2O, DCM, **s89**, DiPEA, 37% over 2 steps. d) PPh_3 (beads), H_2O, MeCN, 70 °C, quant. e) Li, NH_3(liq.), -70 °C → -55 °C, 40%. f) Cy5COOH, PFPOC(O)CF_3, DiPEA, DMF, **s92**, 24% for **52**, or biotin-OSu, DiPEA, 60% for **59**.

Compounds **s87** and **s88**

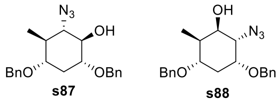

s87 **s88**

Epoxide **23** (0.200 g, 0.616 mmol) was treated according to General procedure D. Purification by silica gel column chromatography (pentane/EtOAc, 25:1) yielded partially separated titled compounds (0.227 g in total, quant.).

s87: ^{1}H NMR (500 MHz, $CDCl_3$) δ 7.44 – 7.29 (m, 10H), 4.79 – 4.41 (m, 4H), 3.55 (td, *J* = 9.3, 2.1 Hz, 1H), 3.27 (ddd, *J* = 11.8, 9.0, 4.3 Hz, 1H), 3.06 – 2.95 (m, 2H), 2.89 (dd, *J* = 11.3, 9.6 Hz, 1H), 2.54 (dt, *J* = 12.2, 4.2 Hz, 1H), 1.56 (ddq, *J* = 11.1, 10.2, 6.4 Hz, 1H), 1.35 (q, *J* = 11.8 Hz, 1H), 1.19 (d, *J* = 6.4 Hz, 3H). ^{13}C NMR (126 MHz, $CDCl_3$) δ 138.12, 137.99, 128.69 (2C), 128.55 (2C), 128.08, 127.92 (3C), 127.89 (2C), 77.61, 77.53, 77.47, 71.61, 71.43, 67.77, 41.81, 33.39, 15.36. HRMS m/z: [M+Na]$^{+}$ calculated for $C_{21}H_{25}N_3O_3$ 390.1788, found 390.1799. **s88**: ^{1}H NMR (500 MHz, $CDCl_3$)

δ 7.45 – 7.28 (m, 10H), 4.68 – 4.62 (m, 3H), 4.45 (d, *J* = 11.4 Hz, 1H), 3.93 (ddt, *J* = 16.8, 6.9, 3.3 Hz, 2H), 3.78 (s, 1H), 3.34 (td, *J* = 10.8, 4.3 Hz, 1H), 2.39 – 2.30 (m, 1H), 2.05 (bs, 1H), 1.88 (ddt, *J* = 13.6, 9.6, 4.8 Hz, 1H), 1.79 (q, *J* = 11.6 Hz, 1H), 1.10 (d, *J* = 6.9 Hz, 3H). ^{13}C NMR (126 MHz, $CDCl_3$) δ 138.60, 138.18, 128.54 (2C), 128.46 (2C), 127.83 (2C), 127.80, 127.71, 127.58 (2C), 76.47, 74.52, 73.00, 71.07, 70.98, 63.12, 36.54, 31.73, 13.45. HRMS m/z: $[M+Na]^+$ calculated for $C_{21}H_{25}N_3O_3$ 390.1788, found 390.1792.

Compound **s90**

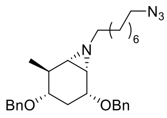

The mixture of compounds **s87** and **s88** (0.220 g, 0.60 mmol) was treated according to General procedure E. Purification by silica gel column chromatography (pentane/Ac, 15:1 → 5:1) yielded 0.160 g of a material that was co-evaporated with toluene before being used directly in the next step. The thus obtained material was treated according to General procedure F. Purification by silica gel column chromatography (pentane/Ac, 25:1) yielded compound **s90** (0.105 g, 37% over 2 steps).

^{1}H NMR (500 MHz, $CDCl_3$) δ 7.45 – 7.26 (m, 10H), 4.70 (s, 2H), 4.61 (d, *J* = 11.6 Hz, 1H), 4.37 (d, *J* = 11.6 Hz, 1H), 3.76 (ddd, *J* = 11.2, 5.2, 3.1 Hz, 1H), 3.25 (t, *J* = 7.0 Hz, 2H), 2.78 (ddd, *J* = 12.3, 9.6, 2.9 Hz, 1H), 2.36 (dt, *J* = 11.4, 6.9 Hz, 1H), 2.18 – 2.06 (m, 2H), 1.88 (dq, *J* = 9.5, 7.1 Hz, 1H), 1.76 (dd, *J* = 6.4, 3.1 Hz, 1H), 1.65 – 1.52 (m, 5H), 1.42 – 1.27 (m, 8H), 1.22 (d, *J* = 6.3 Hz, 1H), 1.14 (d, *J* = 7.1 Hz, 3H). ^{13}C NMR (126 MHz, $CDCl_3$) δ 139.05, 138.76, 128.36 (2C), 128.33 (2C), 127.78 (2C), 127.55 (2C), 127.51, 127.47, 80.86, 74.57, 70.27, 69.97, 61.14, 51.49, 44.32, 41.59, 36.38, 29.90, 29.78, 29.51, 29.10, 28.85, 27.29, 26.71, 18.39.

Compound **s91**

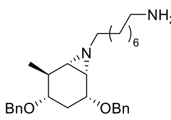

Compound **s90** (0.100 g, 0.210 mmol) was treated according to General procedure G to yield compound **s91** (0.095 g, quant.).

^{1}H NMR (400 MHz, $CDCl_3$) δ 7.44 – 7.22 (m, 10H), 4.68 (s, 2H), 4.59 (d, *J* = 11.6 Hz, 1H), 4.34 (d, *J* = 11.6 Hz, 1H), 3.74 (ddd, *J* = 11.2, 5.2, 3.1 Hz, 1H), 2.76 (ddd, *J* = 12.3, 9.6, 2.9 Hz, 1H), 2.69 – 2.60 (m, 2H), 2.35 (dt, *J* = 11.4, 7.0 Hz, 1H), 2.14 – 2.02 (m, 2H), 1.91 – 1.76 (m, 3H), 1.74 (dd, *J* = 6.3, 3.1 Hz, 1H), 1.62 – 1.50 (m, 3H), 1.45 – 1.25 (m, 10H), 1.20 (d, *J* = 6.3 Hz, 1H), 1.12 (d, *J* = 7.1 Hz, 3H). ^{13}C NMR (101 MHz, $CDCl_3$) δ 138.94, 138.65, 128.31 (2C), 128.28 (2C), 127.73 (2C), 127.51 (2C), 127.46, 127.42, 80.74, 74.43, 70.19, 69.89, 61.14, 44.27, 42.09, 41.48, 36.30, 33.53, 29.82, 29.75, 29.59, 29.39, 27.30, 26.82, 18.33. HRMS m/z: $[M+H]^+$ calculated for $C_{29}H_{42}N_2O_2$ 451.3319, found 451.3317.

Compound **s92**

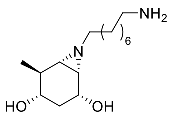

Compound **s91** (0.042 g, 0.093 mmol) was treated according to General procedure H to yield pure compound **s92** (0.010 g, 40 %) as well as impure fractions (0.015 g).

^{1}H NMR (500 MHz, $CDCl_3$) δ 3.98 (ddd, *J* = 11.3, 5.8, 3.6 Hz, 1H), 2.95 (ddd, *J* = 12.2, 9.1, 3.3 Hz, 1H), 2.70 – 2.62 (m, 2H), 2.31 (dt, *J* = 11.4, 7.0 Hz, 1H), 2.19 (dt, *J* = 11.6, 7.4 Hz, 1H), 1.79 – 1.72 (m, 2H), 1.62 – 1.29 (m, 15H), 1.14 (d, *J* = 7.7 Hz, 3H). ^{13}C NMR (126 MHz, $CDCl_3$) δ 73.98, 68.10, 62.01, 46.18, 45.50, 42.25, 38.93, 37.92, 32.92, 30.64, 30.58, 30.50, 28.39, 27.88, 18.41. HRMS m/z: $[M+H]^+$ calculated for $C_{15}H_{30}N_2O_2$ 271.2380, found 271.2378.

Compound **52**

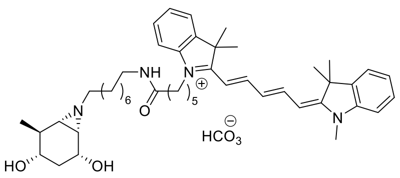

Compound **s92** (0.0049 g, 0.018 mmol) was treated according to General procedure I to yield compound **52** (0.0034 g, 24%).

^{1}H NMR (600 MHz, MeOD) δ 8.30 – 8.20 (m, 2H), 7.52 – 7.47 (m, 2H), 7.45 – 7.39 (m, 2H), 7.33 – 7.24 (m, 4H), 6.63 (t, *J* = 12.4 Hz, 1H), 6.32 – 6.25 (m, 2H), 4.11 (t, *J* = 7.4 Hz, 2H), 3.98 (ddd, *J* = 10.7, 5.7, 3.5 Hz, 1H), 3.63 (s, 3H), 3.12 (t, *J* = 7.2 Hz, 2H), 2.94 (ddd, *J* = 12.1, 9.2, 3.1 Hz, 1H), 2.29 (dt, *J* = 11.6, 7.2 Hz, 1H), 2.21 – 2.13 (m, 3H), 1.86 – 1.79 (m, 2H), 1.79 – 1.65 (m, 15H), 1.60 – 1.52 (m, 3H), 1.50 – 1.41 (m, 5H), 1.37 – 1.28 (m, 10H), 1.12 (d, *J* = 7.1 Hz, 3H). ^{13}C NMR (151 MHz, MeOD) δ 175.69, 175.38, 174.70, 155.54, 144.25, 143.57, 142.65, 142.51, 129.77, 129.75, 126.61, 126.28, 126.25, 123.43, 123.29, 112.05, 111.84, 104.35, 104.29, 73.97, 68.09, 62.00, 50.56, 50.51, 46.17, 45.49, 44.77, 40.40, 38.93, 37.94, 36.69, 31.49, 30.68, 30.58, 30.40, 30.36, 28.39, 28.20, 27.98, 27.94, 27.80, 27.36, 26.56, 18.43. HRMS m/z: $[M]^+$ calculated for $C_{47}H_{67}N_4O_3$ 735.5208, found 735.5220.

Compound **59**

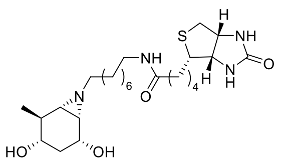

Compound **s92** (0.0046 g, 0.017 mmol) was treated according to General procedure J to yield compound **59** (0.0051 g, 60%).
^{1}H NMR (600 MHz, MeOD) δ 4.49 (ddd, *J* = 7.8, 5.0, 0.9 Hz, 1H), 4.30 (dd, *J* = 7.9, 4.5 Hz, 1H), 3.98 (ddd, *J* = 10.8, 5.7, 3.5 Hz, 1H), 3.23 – 3.11 (m, 3H), 2.99 – 2.90 (m, 2H), 2.71 (d, *J* = 12.7 Hz, 1H), 2.31 (dt, *J* = 11.6, 7.2 Hz, 1H), 2.23 – 2.15 (m, 3H), 1.80 – 1.54 (m, 9H), 1.54 – 1.40 (m, 5H), 1.40 – 1.30 (m, 9H), 1.14 (d, *J* = 7.1 Hz, 3H). ^{13}C NMR (151 MHz, MeOD) δ 175.96, 166.12, 74.01, 68.12, 63.39, 62.02, 61.63, 57.03, 46.20, 45.51, 41.05, 40.38, 38.94, 37.93, 36.83, 30.64, 30.58, 30.43, 30.38, 29.78, 29.52, 28.39, 27.96, 26.96, 18.41. HRMS m/z: [M+H]$^+$ calculated for $C_{25}H_{44}N_4O_4S$ 497.3156, found 497.3153.

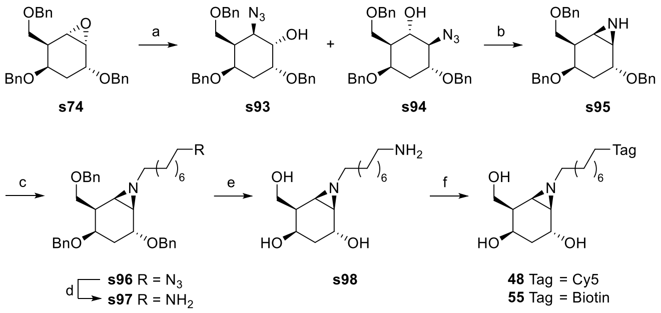

Scheme S5. Synthesis of *galactose*-configured 3-deoxy-β-aziridines. Reagents and conditions: a) NaN_3, $LiClO_4$, DMF, 95 °C, 85% in total. b) PPh_3 (beads), MeCN, 60 °C, 47%. c) 8-Azido-1-octanol, Py, Tf_2O, DCM, **s95**, DiPEA, 60%. d) PPh_3 (beads), H_2O, MeCN, 70 °C, 98%. e) Li, NH_3(liq.), -70 °C → -55 °C, 59%. f) Cy5COOH, PFPOC(O)CF_3, DiPEA, DMF, **s98**, 13% for **48**, or biotin-OSu, DiPEA, 25% for **55**.

Compounds **s93** and **s94**

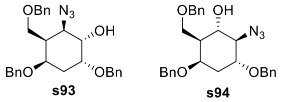

Epoxide **s74** (0.048 g, 0.111 mmol) was treated according to General procedure D. After TLC analysis indicated the presence of the starting material, additional amounts of sodium azide and lithium perchlorate were added and the mixture was stirred for 5 hours at 105 °C. Purification by silica gel column chromatography (pentane/Ac, 25:1 → 20:1) yielded a mixture of the titled compounds (0.045 g in total, 85%). NMR analysis of the mixture was made. Some signals were absent in ^{13}C APT NMR made at 293 K, but could be seen as cross-peaks in HSQC spectrum and were revealed in ^{13}C NMR made at 326 K.
s93 + **s94**: ^{1}H NMR (500 MHz, $CDCl_3$, 326 K) δ 7.41 – 7.27 (m, 27H), 7.23 – 7.19 (m, 2H), 4.67 (d, *J* = 11.6 Hz, 1H), 4.60 – 4.45 (m, 9H), 4.42 (d, *J* = 11.7 Hz, 1H), 4.30 (d, *J* = 11.7 Hz, 1H), 4.14 (dd, *J* = 6.4, 3.1 Hz, 1H), 3.96 – 3.74 (m, 6H), 3.74 – 3.61 (m, 5H), 3.36 (t, *J* = 9.6 Hz, 1H), 3.03 (s, 1H), 2.44 – 2.32 (m, 3H), 2.05 (ddd, *J* = 13.8, 7.1, 3.7 Hz, 1H), 1.97 – 1.87 (m, 2H), 1.39 – 1.34 (m, 2H). ^{13}C NMR (126 MHz, $CDCl_3$, 326 K) δ 138.85, 138.60, 138.55, 138.36, 138.25, 138.16, 128.68, 128.58, 128.57, 128.51, 128.48, 128.46, 128.20, 128.07, 127.94, 127.88, 127.82, 127.77, 127.71, 127.66, 127.65, 127.63, 75.71, 74.41, 73.68, 73.56, 73.53, 73.46, 72.44, 72.31, 71.58, 71.50, 71.44, 71.31, 70.43, 70.37, 67.35, 62.55, 46.95, 40.42, 33.23, 29.42. HRMS m/z: [M+Na]$^+$ calculated for $C_{28}H_{31}N_3O_4$ 496.2207, found 496.2213.

Compound **s95**

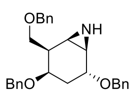

The mixture of compounds **s93** and **s94** (0.135 g, 0.285 mmol) was treated according to General procedure E. Purification by silica gel column chromatography (pentane/Ac + 1% TEA, 10:1 → 5:1) yielded compound **s95** (0.057 g, 47%). Some signals were absent in ^{13}C NMR made at 293 K, but could be seen as cross-peaks in HSQC spectrum.
^{1}H NMR (500 MHz, $CDCl_3$) δ 7.40 – 7.27 (m, 13H), 7.25 – 7.20 (m, 2H), 4.75 – 4.65 (m, 1H), 4.64 – 4.52 (m, 3H), 4.46 (d, *J* = 11.6 Hz, 1H), 4.42 – 4.34 (m, 1H), 3.99 – 3.88 (m, 2H), 3.83 – 3.71 (m, 2H), 2.51 (s, 1H), 2.36 – 2.20 (m, 2H), 1.52 – 1.40 (m, 1H), 1.32 – 1.28 (m, 2H). ^{13}C NMR (126 MHz, $CDCl_3$) δ 138.55, 138.33, 138.25, 128.53, 128.45, 128.44, 127.82, 127.79, 127.78, 127.73, 127.67, 127.66, 73.51, 72.22, 71.75, 71.35, 69.66, 34.24, 32.50.

Compound **s96**

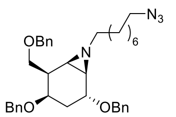

Compound **s95** (0.057 g, 0.133 mmol) was treated according to General procedure F. Purification by silica gel column chromatography (pentane/Ac, 25:1) yielded compound **s96** (0.046 g, 60%).
^{1}H NMR (500 MHz, $CDCl_3$) δ 7.40 – 7.26 (m, 15H), 4.63 – 4.48 (m, 5H), 4.43 (d, *J* = 12.0 Hz, 1H), 4.06 – 4.01 (m, 1H), 3.84 – 3.73 (m, 3H), 3.25 (t, *J* = 7.0 Hz, 2H), 2.72 – 2.63 (m, 1H), 2.32 (dt, *J* = 11.5, 7.5 Hz, 1H), 2.16 (dt, *J* = 11.5, 7.1 Hz, 1H), 1.79 (t, *J* = 6.1 Hz, 1H), 1.76 – 1.69 (m, 2H), 1.67 – 1.51 (m, 5H), 1.37 – 1.24 (m, 8H). ^{13}C

NMR (126 MHz, $CDCl_3$) δ 138.87, 138.76, 138.60, 128.52 (2C), 128.41 (4C), 127.80 (2C), 127.79 (4C), 127.72, 127.57, 127.52, 74.90, 73.50, 71.32, 71.07, 70.74, 68.73, 61.53, 51.56, 42.42, 40.73, 36.01, 29.49, 29.44, 29.23, 28.92, 28.75, 27.43, 26.78. HRMS m/z: $[M+H]^+$ calculated for $C_{36}H_{46}N_4O_3$ 583.3643, found 583.3642.

Compound **s97**

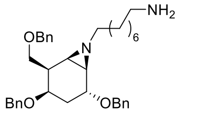

Compound **s96** (0.045 g, 0.077 mmol) was treated according to General procedure G to yield compound **s97** (0.042 g, 98%).

^{1}H NMR (500 MHz, $CDCl_3$) δ 7.38 – 7.25 (m, 15H), 4.62 – 4.46 (m, 5H), 4.42 (d, *J* = 11.9 Hz, 1H), 4.04 – 3.99 (m, 1H), 3.82 – 3.73 (m, 3H), 2.70 – 2.62 (m, 3H), 2.30 (dt, *J* = 11.5, 7.5 Hz, 1H), 2.16 (dt, *J* = 11.5, 7.0 Hz, 1H), 1.81 – 1.66 (m, 5H), 1.62 (dt, *J* = 13.4, 3.4 Hz, 1H), 1.59 – 1.48 (m, 2H), 1.41 (dt, *J* = 13.1, 6.6 Hz, 2H), 1.31 – 1.23 (m, 10H). ^{13}C NMR (126 MHz, $CDCl_3$) δ 138.85, 138.75, 138.59, 128.50 (2C), 128.39 (4C), 127.81 (2C), 127.79 (2C), 127.77 (2C), 127.70, 127.55, 127.51, 74.90, 73.50, 71.31, 71.05, 70.72, 68.71, 61.56, 42.40, 42.23, 40.71, 36.00, 33.67, 29.63, 29.57, 29.48, 28.75, 27.49, 26.94.

Compound **s98**

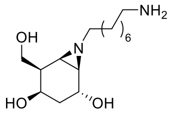

Compound **s97** (0.042 g, 0.075 mmol) was treated according to General procedure H to yield pure compound **s98** (0.0128 g, 59%) as well as impure fractions (0.0066 g).

^{1}H NMR (500 MHz, MeOD) δ 4.15 (dd, *J* = 9.3, 6.9 Hz, 1H), 3.91 – 3.87 (m, 1H), 3.82 (dd, *J* = 10.6, 7.1 Hz, 1H), 3.74 (dd, *J* = 10.6, 7.4 Hz, 1H), 2.65 – 2.59 (m, 2H), 2.43 (dt, *J* = 11.7, 7.1 Hz, 1H), 2.26 (dddd, *J* = 13.1, 7.0, 5.0, 0.9 Hz, 1H), 2.18 (dt, *J* = 11.7, 6.7 Hz, 1H), 2.03 (dt, *J* = 6.2, 2.0 Hz, 1H), 1.91 (tdd, *J* = 7.2, 3.4, 2.6 Hz, 1H), 1.80 (d, *J* = 6.1 Hz, 1H), 1.57 – 1.31 (m, 12H), 1.23 (ddd, *J* = 13.3, 9.4, 2.0 Hz, 1H). ^{13}C NMR (126 MHz, MeOD) δ 67.34, 65.85, 63.07, 61.01, 45.71, 42.76, 42.53, 41.70, 39.71, 33.71, 30.56, 30.52 (2C), 28.37, 27.94.

Compound **48**

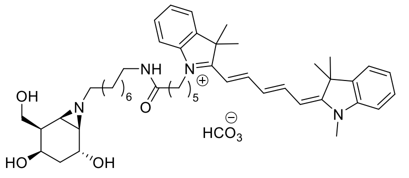

Compound **s98** (0.0065 g, 0.023 mmol) was treated according to General procedure I to yield compound **48** (0.0024 g, 13%).

^{1}H NMR (600 MHz, MeOD) δ 8.31 – 8.20 (m, 2H), 7.52 – 7.46 (m, 2H), 7.45 – 7.37 (m, 2H), 7.34 – 7.23 (m, 4H), 6.63 (t, *J* = 12.4 Hz, 1H), 6.32 – 6.24 (m, 2H), 4.17 – 4.07 (m, 3H), 3.91 – 3.86 (m, 1H), 3.80 (dd, *J* = 10.6, 7.1 Hz, 1H), 3.73 (dd, *J* = 10.6, 7.3 Hz, 1H), 3.63 (s, 3H), 3.12 (t, *J* = 7.2 Hz, 2H), 2.41 (dt, *J* = 11.7, 7.1 Hz, 1H), 2.25 (dddd, *J* = 13.1, 6.9, 5.0, 0.9 Hz, 1H), 2.22 – 2.12 (m, 3H), 2.01 (dt, *J* = 6.1, 1.9 Hz, 1H), 1.93 – 1.90 (m, 1H), 1.87 – 1.77 (m, 3H), 1.73 (s, 11H), 1.71 – 1.66 (m, 2H), 1.56 – 1.42 (m, 6H), 1.41 – 1.29 (m, 8H), 1.22 (ddd, *J* = 13.3, 9.4, 2.0 Hz, 1H). ^{13}C NMR (151 MHz, MeOD) δ 175.70, 175.37, 174.70, 155.53, 144.26, 143.58, 142.65, 142.51, 129.77, 129.75, 126.62, 126.28, 126.25, 123.43, 123.29, 112.05, 111.84, 104.36, 104.30, 67.34, 65.85, 63.09, 60.96, 50.56, 50.51, 45.70, 44.77, 42.76, 41.69, 40.38, 39.72, 36.70, 31.48, 30.54, 30.51, 30.37, 30.31, 28.36, 28.20, 27.94, 27.80, 27.37, 26.56. HRMS m/z: $[M]^+$ calculated for $C_{47}H_{67}N_4O_4$ 751.5157, found 751.5165.

Compound **55**

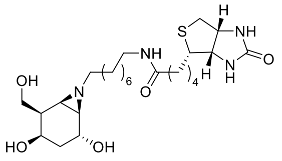

Compound **s98** (0.004 g, 0.014 mmol) was treated according to General procedure J to yield compound **55** (0.0018 g, 25%).

^{1}H NMR (850 MHz, MeOD) δ 4.49 (ddd, *J* = 7.9, 5.0, 1.0 Hz, 1H), 4.30 (dd, *J* = 7.9, 4.5 Hz, 1H), 4.15 (dd, *J* = 9.4, 7.0 Hz, 1H), 3.89 (ddt, *J* = 5.3, 3.6, 1.8 Hz, 1H), 3.81 (dd, *J* = 10.6, 7.1 Hz, 1H), 3.75 (dd, *J* = 10.5, 7.4 Hz, 1H), 3.21 (ddd, *J* = 9.0, 5.8, 4.5 Hz, 1H), 3.19 – 3.13 (m, 2H), 2.93 (dd, *J* = 12.8, 5.0 Hz, 1H), 2.71 (d, *J* = 12.7 Hz, 1H), 2.43 (dt, *J* = 11.7, 7.2 Hz, 1H), 2.26 (dddd, *J* = 13.1, 7.0, 5.1, 0.9 Hz, 1H), 2.22 – 2.16 (m, 3H), 2.03 (dt, *J* = 6.2, 1.9 Hz, 1H), 1.93 – 1.90 (m, 1H), 1.81 (d, *J* = 6.1 Hz, 1H), 1.77 – 1.57 (m, 5H), 1.57 – 1.47 (m, 4H), 1.47 – 1.39 (m, 3H), 1.39 – 1.31 (m, 6H), 1.23 (ddd, *J* = 13.3, 9.4, 2.0 Hz, 1H). ^{13}C NMR (214 MHz, MeOD) δ 175.98, 166.13, 67.35, 65.87, 63.39, 63.07, 61.62, 60.99, 57.05, 45.69, 42.76, 41.68, 41.05, 40.35, 39.69, 36.83, 30.56, 30.50, 30.40, 30.33, 29.80, 29.52, 28.36, 27.92, 26.97. HRMS m/z: $[M+H]^+$ calculated for $C_{25}H_{44}N_4O_5S$ 513.3105, found 513.3106.

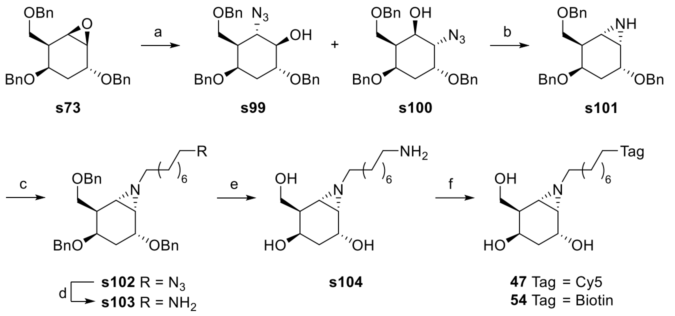

Scheme S6. Synthesis of *galactose*-configured 3-deoxy-α-aziridines. Reagents and conditions: a) NaN_3, $LiClO_4$, DMF, 95 °C, 93% in total. b) PPh_3 (beads), MeCN, 60 °C, 50%. c) 8-Azido-1-octanol, Py, Tf_2O, DCM, **s101**, DiPEA, 56%. d) PPh_3 (beads), H_2O, MeCN, 70 °C, 96%. e) Li, NH_3(liq.), -70 °C → -55 °C, 70%. f) Cy5COOH, PFPOC(O)CF_3, DiPEA, DMF, **s104**, 22% for **47**, or biotin-OSu, DiPEA, 52% for **54**.

Compounds **s99** and **s100**

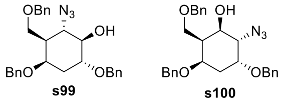

Epoxide **s73** (0.130 g, 0.302 mmol) was treated according to General procedure D. Purification by silica gel column chromatography (pentane/Ac, 30:1 → 20:1) yielded partially separated titled compounds (0.133 g in total, 93%).

s99: ^{1}H NMR (500 MHz, $CDCl_3$) δ 7.41 – 7.29 (m, 13H), 7.27 – 7.24 (m, 2H), 4.63 (d, *J* = 11.7 Hz, 1H), 4.59 – 4.52 (m, 2H), 4.51 – 4.45 (m, 2H), 4.38 (d, *J* = 11.6 Hz, 1H), 4.07 – 4.00 (m, 1H), 3.75 – 3.67 (m, 3H), 3.60 (td, *J* = 9.2, 1.9 Hz, 1H), 3.49 (dd, *J* = 11.8, 9.4 Hz, 1H), 2.93 (d, *J* = 2.2 Hz, 1H), 2.40 (dt, *J* = 13.7, 4.0 Hz, 1H), 1.80 (dddd, *J* = 11.7, 9.1, 5.0, 2.6 Hz, 1H), 1.23 (ddd, *J* = 13.7, 11.6, 2.1 Hz, 1H). ^{13}C NMR (126 MHz, $CDCl_3$) δ 138.53, 138.31, 138.19, 128.66 (2C), 128.50 (2C), 128.45 (2C), 128.06 (2C), 128.05, 127.83 (2C), 127.76, 127.75, 127.68 (2C), 78.45, 77.14, 73.42, 72.43, 71.95, 71.66, 68.20, 62.59, 45.31, 31.42. HRMS m/z: $[M+Na]^+$ calculated for $C_{28}H_{31}N_3O_4$ 496.2207, found 496.2215. **s100**: ^{1}H NMR (500 MHz, $CDCl_3$) δ 7.43 – 7.27 (m, 13H), 7.19 – 7.14 (m, 2H), 4.68 (d, *J* = 11.9 Hz, 1H), 4.60 – 4.52 (m, 2H), 4.51 – 4.45 (m, 2H), 4.31 (d, *J* = 11.4 Hz, 1H), 4.19 – 4.11 (m, 2H), 4.11 – 4.06 (m, 1H), 3.99 – 3.91 (m, 1H), 3.76 (t, *J* = 9.0 Hz, 1H), 3.69 – 3.59 (m, 2H), 2.27 (d, *J* = 13.6 Hz, 1H), 2.10 (s, 1H), 1.79 (ddd, *J* = 13.7, 11.6, 2.3 Hz, 1H). ^{13}C NMR (126 MHz, $CDCl_3$) δ 138.30, 138.11, 137.46, 128.64 (2C), 128.60 (2C), 128.51 (2C), 128.15, 127.92, 127.89 (2C), 127.85 (2C), 127.78 (3C), 76.18, 73.42, 71.99, 71.85, 71.49, 71.05, 68.18, 63.71, 39.12, 29.51. HRMS m/z: $[M+Na]^+$ calculated for $C_{28}H_{31}N_3O_4$ 496.2207, found 496.2217.

Compound **s101**

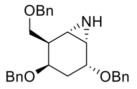

The mixture of compounds **s99** and **s100** (0.120 g, 0.254 mmol) was treated according to General procedure E. Purification by silica gel column chromatography (pentane/EtOAc + 1% TEA, 5:1 → 2:1) yielded compound **s101** (0.054 g, 50%).

^{1}H NMR (300 MHz, $CDCl_3$) δ 7.45 – 7.27 (m, 13H), 7.25 – 7.17 (m, 2H), 4.74 – 4.61 (m, 2H), 4.61 – 4.41 (m, 3H), 4.31 (d, *J* = 11.6 Hz, 1H), 4.12 (ddd, *J* = 10.5, 5.7, 3.6 Hz, 1H), 3.74 (dt, *J* = 5.4, 2.7 Hz, 1H), 3.70 – 3.55 (m, 2H), 2.55 (dd, *J* = 6.3, 3.6 Hz, 1H), 2.22 – 2.08 (m, 3H), 1.43 – 1.35 (m, 1H). ^{13}C NMR (75 MHz, $CDCl_3$) δ 138.97, 138.56, 138.47, 128.46 (2C), 128.44 (2C), 128.41 (2C), 127.89 (2C), 127.81 (2C), 127.76 (2C), 127.67 (2C), 127.62, 73.99, 73.26, 71.35, 70.75, 70.44, 70.31, 40.84, 32.32, 32.29, 27.33.

Compound **s102**

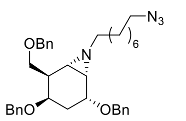

Compound **s101** (0.053 g, 0.123 mmol) was treated according to General procedure F. Purification by silica gel column chromatography (pentane/Ac, 12:1) yielded compound **s102** (0.040 g, 56%).

^{1}H NMR (500 MHz, $CDCl_3$) δ 7.43 – 7.27 (m, 13H), 7.23 – 7.19 (m, 2H), 4.75 – 4.63 (m, 2H), 4.59 – 4.50 (m, 2H), 4.46 (d, *J* = 11.7 Hz, 1H), 4.29 (d, *J* = 11.7 Hz, 1H), 4.01 (ddd, *J* = 10.6, 5.7, 3.7 Hz, 1H), 3.75 – 3.70 (m, 1H), 3.68 – 3.57 (m, 2H), 3.24 (t, *J* = 7.0 Hz, 2H), 2.41 (ddd, *J* = 11.5, 8.0, 6.5 Hz, 1H), 2.18 – 2.07 (m, 3H), 1.91 – 1.84 (m, 1H), 1.65 – 1.52 (m, 4H), 1.46 (dt, *J* = 7.7, 1.2 Hz, 1H), 1.44 – 1.39 (m, 1H), 1.38 – 1.26 (m, 8H). ^{13}C NMR (126 MHz, $CDCl_3$) δ 139.22, 138.67, 138.65, 128.44 (2C), 128.40 (4C), 127.81 (2C), 127.73 (2C), 127.64 (3C), 127.60, 127.52, 74.42, 73.15, 71.30, 70.65, 70.54, 70.34, 61.51, 51.57, 40.99, 40.94, 40.74, 29.78, 29.58, 29.14, 28.91, 28.37, 27.40, 26.78. HRMS m/z: $[M+H]^+$ calculated for $C_{36}H_{46}N_4O_3$ 583.3643, found 583.3642.

Compound **s103**

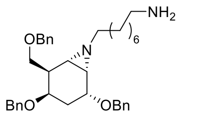

Compound **s102** (0.038 g, 0.065 mmol) was treated according to General procedure G to yield compound **s103** (0.035 g, 96%).

^{1}H NMR (500 MHz, $CDCl_3$) δ 7.42 – 7.26 (m, 13H), 7.22 – 7.18 (m, 2H), 4.73 – 4.61 (m, 2H), 4.59 – 4.49 (m, 2H), 4.44 (d, *J* = 11.7 Hz, 1H), 4.28 (d, *J* = 11.7 Hz, 1H), 4.00 (ddd, *J* = 10.7, 5.6, 3.6 Hz, 1H), 3.74 – 3.68 (m, 1H), 3.67 – 3.56 (m, 2H), 2.69 – 2.63 (m, 2H), 2.40 (ddd, *J* = 11.5, 8.1, 6.4 Hz, 1H), 2.16 – 2.06 (m, 3H), 1.89 – 1.70 (m, 3H), 1.63 – 1.54 (m, 2H), 1.47 – 1.38 (m, 4H), 1.36 – 1.23 (m, 8H). ^{13}C NMR (126 MHz, $CDCl_3$) δ 139.20, 138.66, 138.63, 128.42 (2C), 128.39 (2C), 128.39 (2C), 127.80 (2C), 127.73 (2C), 127.63 (3C), 127.58, 127.50, 74.39, 73.13, 71.28, 70.64, 70.50, 70.32, 61.55, 42.24, 40.96, 40.91, 40.71, 33.68, 29.81, 29.70, 29.48, 28.37, 27.46, 26.94. HRMS m/z: $[M+H]^+$ calculated for $C_{36}H_{48}N_2O_3$ 557.3738, found 557.3741.

Compound **s104**

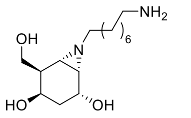

Compound **s103** (0.035 g, 0.063 mmol) was treated according to General procedure H to yield pure compound **s104** (0.0126 g, 70%) as well as impure fractions (0.005 g).

^{1}H NMR (500 MHz, MeOD) δ 4.24 (ddd, *J* = 10.4, 6.0, 3.9 Hz, 1H), 3.97 – 3.91 (m, 1H), 3.73 (dd, *J* = 10.9, 6.6 Hz, 1H), 3.65 (dd, *J* = 10.9, 8.0 Hz, 1H), 2.68 – 2.61 (m, 2H), 2.35 (dt, *J* = 11.7, 7.1 Hz, 1H), 2.25 (dt, *J* = 11.7, 7.3 Hz, 1H), 1.91 – 1.82 (m, 2H), 1.75 (dddd, *J* = 7.9, 6.6, 3.8, 1.2 Hz, 1H), 1.66 – 1.55 (m, 3H), 1.53 – 1.43 (m, 2H), 1.40 – 1.28 (m, 9H). ^{13}C NMR (126 MHz, MeOD) δ 68.43, 64.70, 63.74, 62.24, 44.70, 43.62, 42.40, 42.36, 35.99, 33.34, 30.68, 30.52, 30.50, 28.43, 27.93. HRMS m/z: $[M+H]^+$ calculated for $C_{15}H_{30}N_2O_3$ 287.2329, found 287.2329.

Compound **47**

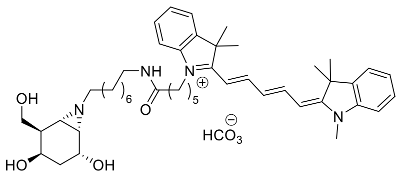

Compound **s104** (0.0042 g, 0.014 mmol) was treated according to General procedure I to yield compound **47** (0.0026 g, 22%).

^{1}H NMR (600 MHz, MeOD) δ 8.30 – 8.21 (m, 2H), 7.52 – 7.47 (m, 2H), 7.44 – 7.39 (m, 2H), 7.33 – 7.24 (m, 4H), 6.63 (t, *J* = 12.4 Hz, 1H), 6.31 – 6.24 (m, 2H), 4.23 (ddd, *J* = 10.4, 6.1, 4.0 Hz, 1H), 4.11 (t, *J* = 7.4 Hz, 2H), 3.95 – 3.92 (m, 1H), 3.73 (dd, *J* = 10.9, 6.6 Hz, 1H), 3.67 – 3.61 (m, 4H), 3.12 (t, *J* = 7.2 Hz, 2H), 2.32 (ddd, *J* = 11.7, 8.3, 6.5 Hz, 1H), 2.27 – 2.16 (m, 3H), 1.89 – 1.80 (m, 4H), 1.76 – 1.71 (m, 13H), 1.71 – 1.66 (m, 2H), 1.62 – 1.54 (m, 3H), 1.49 – 1.42 (m, 4H), 1.36 – 1.28 (m, 9H). ^{13}C NMR (151 MHz, MeOD) δ 175.30, 174.98, 174.30, 155.15, 143.86, 143.18, 142.25, 142.12, 129.38, 129.36, 126.22, 125.89, 125.86, 123.04, 122.90, 111.66, 111.45, 103.96, 103.90, 68.01, 64.29, 63.36, 61.82, 50.16, 50.12, 44.37, 44.30, 43.23, 41.96, 40.01, 36.30, 35.59, 31.09, 30.30, 30.12, 30.01, 29.94, 28.03, 27.81, 27.60, 27.55, 27.41, 26.97, 26.17. HRMS m/z: $[M]^+$ calculated for $C_{47}H_{67}N_4O_4$ 751.5157, found 751.5163.

Compound **54**

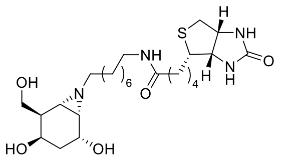

Compound **s104** (0.0039 g, 0.014 mmol) was treated according to General procedure J to yield compound **54** (0.0036 g, 52%).

^{1}H NMR (600 MHz, MeOD) δ 4.49 (ddd, *J* = 7.9, 5.1, 1.0 Hz, 1H), 4.30 (dd, *J* = 7.9, 4.5 Hz, 1H), 4.24 (ddd, *J* = 10.4, 6.1, 4.1 Hz, 1H), 3.94 (ddd, *J* = 5.1, 3.4, 1.4 Hz, 1H), 3.73 (dd, *J* = 10.9, 6.6 Hz, 1H), 3.65 (dd, *J* = 10.9, 7.9 Hz, 1H), 3.23 – 3.11 (m, 3H), 2.93 (dd, *J* = 12.8, 5.0 Hz, 1H), 2.71 (d, *J* = 12.7 Hz, 1H), 2.34 (ddd, *J* = 11.7, 8.4, 6.5 Hz, 1H), 2.26 (ddd, *J* = 11.7, 8.5, 6.7 Hz, 1H), 2.19 (td, *J* = 7.3, 1.1 Hz, 2H), 1.90 – 1.83 (m, 2H), 1.77 – 1.55 (m, 8H), 1.53 – 1.40 (m, 4H), 1.40 – 1.28 (m, 9H). ^{13}C NMR (151 MHz, MeOD) δ 175.96, 166.13, 68.41, 64.69, 63.74, 63.39, 62.21, 61.62, 57.04, 44.71, 43.62, 42.37, 41.06, 40.37, 36.83, 35.97, 30.68, 30.52, 30.43, 30.36, 29.80, 29.52, 28.43, 27.97, 26.97. HRMS m/z: $[M+H]^+$ calculated for $C_{25}H_{44}N_4O_5S$ 513.3105, found 513.3109.

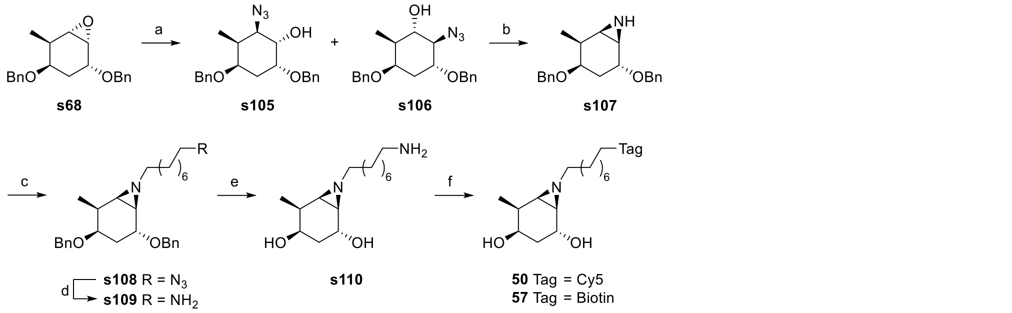

Scheme S7. Synthesis of *galactose*-configured 3,6-dideoxy-β-aziridines. Reagents and conditions: a) NaN_3, $LiClO_4$, DMF, 95 °C, 69% in total. b) PPh_3 (beads), MeCN, 60 °C, 74%. c) 8-Azido-1-octanol, Py, Tf_2O, DCM, **s107**, DiPEA, 71%. d) PPh_3 (beads), H_2O, MeCN, 70 °C, quant. e) Li, NH_3(liq.), -70 °C → -55 °C, 73%. f) Cy5COOH, PFPOC(O)CF_3, DiPEA, DMF, **s110**, 25% for **50**, or biotin-OSu, DiPEA, 15% for **57**.

Compounds **s105** and **s106**

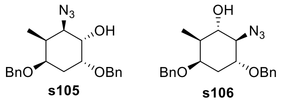

Epoxide **s68** (0.230 g, 0.71 mmol) was treated according to General procedure D. After TLC analysis indicated the presence of the starting material, additional amounts of sodium azide and lithium perchlorate were added and the mixture was stirred for 5 hours at 95 °C. Purification by silica gel column chromatography (DCM/EtOAc, 60:1) yielded partially separated titled compounds (0.180 g in total, 69%) as well as re-isolated staring material (0.036 g, 0.111 mmol, 16%).

s105: ^{1}H NMR (500 MHz, $CDCl_3$) δ 7.39 – 7.24 (m, 10H), 4.60 (d, *J* = 11.4 Hz, 1H), 4.54 (d, *J* = 11.8 Hz, 1H), 4.47 – 4.39 (m, 2H), 3.87 (dt, *J* = 4.3, 2.9 Hz, 1H), 3.79 (dt, *J* = 11.2, 4.3 Hz, 1H), 3.75 – 3.65 (m, 2H), 2.57 – 2.47 (m, 1H), 2.39 (d, *J* = 8.4 Hz, 1H), 2.14 (dt, *J* = 14.7, 4.3 Hz, 1H), 1.67 (ddd, *J* = 14.1, 11.2, 2.6 Hz, 1H), 0.98 (d, *J* = 7.2 Hz, 3H). ^{13}C NMR (126 MHz, $CDCl_3$) δ 138.45, 137.82, 128.73 (2C), 128.59 (2C), 128.20, 128.02 (2C), 127.84, 127.72 (2C), 75.94, 73.31, 71.62, 70.70, 70.65, 64.25, 35.50, 28.37, 7.00. HRMS m/z: $[M+Na]^+$ calculated for $C_{21}H_{25}N_3O_3$ 390.1788, found 390.1795. **s106**: ^{1}H NMR (500 MHz, $CDCl_3$) δ 7.45 – 7.27 (m, 10H), 4.67 (d, *J* = 11.5 Hz, 1H), 4.59 (d, *J* = 11.5 Hz, 1H), 4.47 (d, *J* = 11.8 Hz, 1H), 4.35 (d, *J* = 11.8 Hz, 1H), 3.71 (ddd, *J* = 11.6, 9.5, 4.5 Hz, 1H), 3.59 (q, *J* = 2.8 Hz, 1H), 3.43 (t, *J* = 10.1 Hz, 1H), 3.35 (t, *J* = 9.5 Hz, 1H), 2.56 (s, 1H), 2.40 (ddd, *J* = 13.8, 4.5, 3.5 Hz, 1H), 1.62 (dtd, *J* = 13.4, 6.7, 2.8 Hz, 1H), 1.36 (ddd, *J* = 13.7, 11.6, 2.2 Hz, 1H), 1.16 (d, *J* = 6.7 Hz, 3H). ^{13}C NMR (126 MHz, $CDCl_3$) δ 138.54, 138.04, 128.53 (2C), 128.42 (2C), 128.19 (2C), 127.93, 127.71, 127.60 (2C), 77.03, 76.15, 72.68, 72.28, 72.20, 71.42, 41.51, 33.18, 14.04. HRMS m/z: $[M+Na]^+$ calculated for $C_{21}H_{25}N_3O_3$ 390.1788, found 390.1785.

Compound **s107**

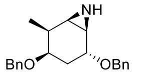

The mixture of compounds **s105** and **s106** (0.115 g, 0.313 mmol) was treated according to General procedure E. Purification by silica gel column chromatography (pentane/EtOAc + 1% TEA, 5:1 → 3:1) yielded compound **s107** (0.075 g, 74%). Some signals were absent in ^{13}C APT NMR made at 293 K, but could be seen as cross-peaks in HSQC spectrum and were revealed in ^{13}C APT NMR made at 323 K.

^{1}H NMR (300 MHz, $CDCl_3$, 323 K) δ 7.39 – 7.20 (m, 10H), 4.69 – 4.54 (m, 2H), 4.48 – 4.34 (m, 2H), 3.92 (dd, *J* = 7.6, 4.8 Hz, 1H), 3.67 (ddd, *J* = 7.5, 5.2, 2.3 Hz, 1H), 2.36 – 2.22 (m, 2H), 2.21 – 2.03 (m, 2H), 1.55 – 1.41 (m, 1H), 1.16 (d, *J* = 7.0 Hz, 3H). ^{13}C NMR (75 MHz, $CDCl_3$, 323 K) δ 138.70, 138.67, 128.50 (2C), 128.43 (2C), 127.77 (2C), 127.69 (2C), 127.63 (2C), 74.61, 73.45, 71.47, 71.39, 35.86, 34.91, 32.12, 13.83.

Compound **s108**

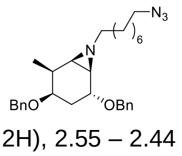

Compound **s107** (0.080 g, 0.247 mmol) was treated according to General procedure F. Purification by silica gel column chromatography (pentane/EtOAc, 20:1 → 15:1) yielded compound **s108** (0.084 g, 71%).

^{1}H NMR (500 MHz, $CDCl_3$) δ 7.40 – 7.26 (m, 10H), 4.63 (d, *J* = 11.7 Hz, 1H), 4.54 (d, *J* = 11.9 Hz, 2H), 4.43 (d, *J* = 12.0 Hz, 1H), 4.06 (dt, *J* = 4.6, 2.1 Hz, 1H), 3.75 (ddd, *J* = 10.7, 6.6, 4.2 Hz, 1H), 3.26 (t, *J* = 7.0 Hz, 2H), 2.55 – 2.44 (m, 2H), 1.93 (ddd, *J* = 11.5, 8.7, 5.5 Hz, 1H), 1.75 – 1.66 (m, 3H), 1.64 – 1.47 (m, 5H), 1.42 – 1.27 (m, 8H), 1.08 (d, *J* = 6.9 Hz, 3H). ^{13}C NMR (126 MHz, $CDCl_3$) δ 139.00, 138.64, 128.44 (2C), 128.34 (2C), 127.74 (2C), 127.61 (3C), 127.43, 75.46, 71.88, 71.19, 70.21, 61.39, 51.49, 43.48, 42.43, 29.67, 29.47, 29.21, 29.14, 28.86, 27.30, 27.03, 26.70, 10.98.

Compound **s109**

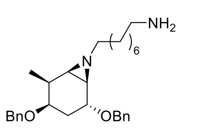

Compound **s108** (0.080 g, 0.168 mmol) was treated according to General procedure G to yield compound **s109** (0.076 g, quant.).

^{1}H NMR (500 MHz, $CDCl_3$) δ 7.36 – 7.23 (m, 10H), 4.60 (d, *J* = 11.7 Hz, 1H), 4.51 (d, *J* = 11.8 Hz, 2H), 4.40 (d, *J* = 12.0 Hz, 1H), 4.04 (dt, *J* = 4.4, 2.0 Hz, 1H), 3.73 (ddd, *J* = 10.7, 6.6, 4.2 Hz, 1H), 2.67 (t, *J* = 7.1 Hz, 2H), 2.52 – 2.42 (m, 2H), 1.89 (ddd, *J* = 11.5, 8.8, 5.4 Hz, 1H), 1.80 – 1.62 (m, 5H), 1.59 (t, *J* = 6.3 Hz, 1H), 1.56 – 1.39 (m, 4H), 1.36 – 1.24 (m, 8H), 1.05 (d, *J* = 6.9 Hz, 3H). ^{13}C NMR (126 MHz, $CDCl_3$) δ 139.03, 138.67, 128.50 (2C), 128.40 (2C), 127.80 (2C), 127.67 (3C), 127.49, 75.52, 71.92, 71.25, 70.26, 61.50, 43.52, 42.46, 42.24, 33.68, 29.76, 29.68, 29.54, 29.22, 27.43, 27.07, 26.93, 11.02.

Compound **s110**

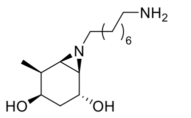

Compound **s109** (0.040 g, 0.089 mmol) was treated according to General procedure H to yield compound **s110** (0.0175 g, 73%) containing an unidentified impurity.

^{1}H NMR (500 MHz, MeOD) δ 4.12 (dd, *J* = 9.2, 6.8 Hz, 1H), 3.65 (ddt, *J* = 5.3, 3.5, 1.7 Hz, 1H), 2.66 – 2.58 (m, 1H), 2.46 (dt, *J* = 11.7, 7.1 Hz, 1H), 2.22 (dddd, *J* = 13.1, 6.7, 5.3, 0.9 Hz, 1H), 2.17 – 2.09 (m, 1H), 1.94 – 1.87 (m, 1H), 1.83 (ddd, *J* = 6.1, 2.9, 1.4 Hz, 1H), 1.76 (d, *J* = 6.0 Hz, 1H), 1.58 – 1.31 (m, 12H), 1.25 (ddd, *J* = 13.2, 9.2, 2.0 Hz, 1H), 1.17 (d, *J* = 7.0 Hz, 3H). ^{13}C NMR (126 MHz, MeOD) δ 70.66, 65.64, 61.17, 46.69, 46.40, 42.57, 39.87, 33.85, 33.74, 30.72, 30.57, 28.36, 27.98, 15.76.

Compound **50**

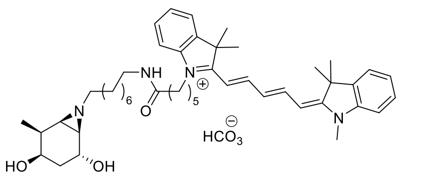

Compound **s110** (0.0042 g, 0.015 mmol) was treated according to General procedure I to yield compound **50** (0.003 g, 25%).

^{1}H NMR (600 MHz, MeOD) δ 8.29 – 8.19 (m, 2H), 7.52 – 7.47 (m, 2H), 7.45 – 7.38 (m, 2H), 7.33 – 7.23 (m, 4H), 6.63 (t, *J* = 12.4 Hz, 1H), 6.32 – 6.24 (m, 2H), 4.14 – 4.07 (m, 3H), 3.67 – 3.59 (m, 4H), 3.12 (t, *J* = 7.2 Hz, 2H), 2.42 (dt, *J* = 11.8, 7.2 Hz, 1H), 2.25 – 2.16 (m, 3H), 2.11 (dt, *J* = 11.7, 6.6 Hz, 1H), 1.89 – 1.86 (m, 1H), 1.86 – 1.78 (m, 3H), 1.78 – 1.64 (m, 15H), 1.56 – 1.43 (m, 6H), 1.40 – 1.29 (m, 8H), 1.24 (ddd, *J* = 13.2, 9.2, 2.0 Hz, 1H), 1.15 (d, *J* = 7.0 Hz, 3H). ^{13}C NMR (151 MHz, MeOD) δ 175.70, 175.37, 174.70, 155.53, 144.25, 143.57, 142.65, 142.51, 129.77, 129.75, 126.62, 126.28, 126.25, 123.43, 123.29, 112.05, 111.84, 104.35, 104.30, 70.67, 65.65, 61.12, 50.56, 50.51, 46.69, 46.39, 44.77, 40.39, 39.88, 36.69, 33.72, 31.48, 30.69, 30.54, 30.38, 30.34, 28.35, 28.20, 27.96, 27.94, 27.80, 27.36, 26.55, 15.78. HRMS m/z: $[M]^+$ calculated for $C_{47}H_{67}N_4O_3$ 735.5208, found 735.5216.

Compound **57**

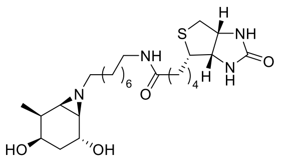

Compound **s110** (0.004 g, 0.015 mmol) was treated according to General procedure J to yield compound **57** (0.0011 g, 15%).

^{1}H NMR (850 MHz, MeOD) δ 4.49 (ddd, *J* = 7.9, 5.0, 1.0 Hz, 1H), 4.30 (dd, *J* = 7.9, 4.5 Hz, 1H), 4.12 (dd, *J* = 9.2, 6.8 Hz, 1H), 3.66 – 3.63 (m, 1H), 3.20 (ddd, *J* = 9.0, 5.8, 4.5 Hz, 1H), 3.16 (td, *J* = 7.0, 5.0 Hz, 2H), 2.93 (dd, *J* = 12.7, 5.0 Hz, 1H), 2.71 (d, *J* = 12.7 Hz, 1H), 2.45 (dt, *J* = 11.7, 7.2 Hz, 1H), 2.23 (dddd, *J* = 13.1, 6.8, 5.2, 0.9 Hz, 1H), 2.19 (td, *J* = 7.4, 2.0 Hz, 2H), 2.14 (ddd, *J* = 11.7, 7.3, 6.0 Hz, 1H), 1.93 – 1.89 (m, 1H), 1.83 (ddd, *J* = 6.2, 2.8, 1.4 Hz, 1H), 1.76 (d, *J* = 6.0 Hz, 1H), 1.75 – 1.71 (m, 1H), 1.71 – 1.65 (m, 1H), 1.65 – 1.57 (m, 2H), 1.57 – 1.47 (m, 4H), 1.47 – 1.40 (m, 3H), 1.40 – 1.32 (m, 5H), 1.25 (ddd, *J* = 13.2, 9.2, 2.0 Hz, 1H), 1.17 (d, *J* = 6.9 Hz, 2H). ^{13}C NMR (151 MHz, MeOD) δ 175.97, 166.12, 70.69, 65.67, 63.40, 61.63, 61.17, 57.03, 46.70, 46.41, 41.05, 40.36, 39.88, 36.83, 33.75, 30.71, 30.52, 30.40, 30.34, 29.79, 29.52, 28.35, 27.93, 26.96, 15.77. HRMS m/z: $[M+H]^+$ calculated for $C_{25}H_{44}N_4O_4S$ 497.3156, found 497.3156.

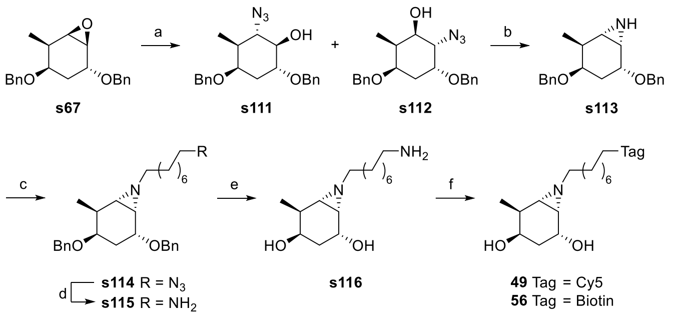

Scheme S8. Synthesis of *g*alactose-configured 3,6-dideoxy-α-aziridines. Reagents and conditions: a) NaN_3, $LiClO_4$, DMF, 95 °C, 99% in total. b) PPh_3 (beads), MeCN, 60 °C, 23%. c) 8-Azido-1-octanol, Py, Tf_2O, DCM, **s113**, DiPEA, 35%. d) PPh_3 (beads), H_2O, MeCN, 70 °C, 93%. e) Li, NH_3(liq.), -70 °C → -55 °C, 97%. f) Cy5COOH, PFPOC(O)CF_3, DiPEA, DMF, **s116**, 6% for **49**, or biotin-OSu, DiPEA, 24% for **56**.

Compounds **s111** and **s112**

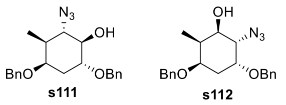

Epoxide **s67** (0.300 g, 0.925 mmol) was treated according to General procedure D. Purification by silica gel column chromatography (pentane/DCM/Ac, 40:8:1) yielded partially separated titled compounds (0.336 g in total, 99%). Some signals were broad in ^{13}C NMR of **s112** made at 293 K, but could be seen as cross-peaks in HSQC spectrum and were partially revealed in ^{13}C NMR made at 332 K.

s111: ^1H NMR (500 MHz, $CDCl_3$) δ 7.41 – 7.27 (m, 10H), 4.65 – 4.54 (m, 2H), 4.51 (d, *J* = 11.8 Hz, 1H), 4.37 (d, *J* = 11.7 Hz, 1H), 3.73 – 3.66 (m, 1H), 3.60 – 3.56 (m, 1H), 3.53 (t, *J* = 9.3 Hz, 1H), 3.42 (dd, *J* = 11.3, 9.5 Hz, 1H), 2.96 (bs, 1H), 2.41 – 2.34 (m, 1H), 1.54 (dqd, *J* = 11.2, 6.7, 2.7 Hz, 1H), 1.25 (ddd, *J* = 13.8, 11.7, 2.2 Hz, 1H), 1.17 (d, *J* = 6.7 Hz, 3H). ^{13}C NMR (126 MHz, $CDCl_3$) δ 138.44, 138.28, 128.62 (2C), 128.45 (2C), 128.04 (2C), 127.98, 127.75, 127.63 (2C), 78.17, 77.11, 77.06, 72.01, 71.37, 66.75, 40.19, 31.76, 14.97. **s112**: ^1H NMR (500 MHz, $CDCl_3$, 332 K) δ 7.38 – 7.23 (m, 8H), 7.23 – 7.18 (m, 2H), 4.62 (d, *J* = 12.0 Hz, 1H), 4.56 – 4.44 (m, 2H), 4.34 (d, *J* = 11.5 Hz, 1H), 4.13 – 4.00 (m, 2H), 3.75 – 3.66 (m, 2H), 3.36 (bs, 1H), 2.21 – 2.11 (m, 1H), 1.99 – 1.88 (m, 1H), 1.78 (ddd, *J* = 13.8, 11.1, 2.5 Hz, 1H), 1.11 (d, *J* = 7.2 Hz, 3H). ^{13}C NMR (126 MHz, $CDCl_3$, 332 K) δ 138.40, 137.80, 128.60 (2C), 128.52 (2C), 128.05, 127.79 (3C), 127.73 (2C), 79.88, 74.29, 72.07, 71.55, 71.13, 64.31, 33.59, 29.94, 14.07. HRMS m/z: $[M+Na]^+$ calculated for $C_{21}H_{25}N_3O_3$ 390.1788, found 390.1781.

Compound **s113**

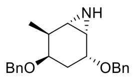

The mixture of compounds **s111** and **s112** (0.325 g, 0.885 mmol) was treated according to General procedure E. Purification by silica gel column chromatography (DCM/MeOH, 70:1) yielded compound **s113** (0.065 g, 23%). ^1H NMR (500 MHz, $CDCl_3$) δ 7.43 – 7.26 (m, 10H), 4.73 – 4.61 (m, 2H), 4.49 (d, *J* = 11.7 Hz, 1H), 4.36 (d, *J* = 11.8 Hz, 1H), 4.10 (ddd, *J* = 9.4, 5.8, 4.0 Hz, 1H), 3.54 (ddd, *J* = 6.3, 4.4, 1.8 Hz, 1H), 2.51 (dd, *J* = 6.1, 4.0 Hz, 1H), 2.10 – 2.00 (m, 3H), 1.47 (ddd, *J* = 13.6, 9.0, 1.8 Hz, 1H), 1.12 (d, *J* = 7.3 Hz, 3H). ^{13}C NMR (126 MHz, $CDCl_3$) δ 138.94, 138.75, 128.50 (2C), 128.44 (2C), 128.39 (2C), 127.89 (2C), 127.77 (2C), 127.61 (2C), 75.90, 71.27, 70.69, 70.38, 36.46, 34.63, 32.31, 27.98, 15.69.

Compound **s114**

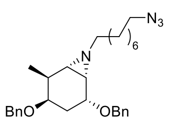

Compound **s113** (0.050 g, 0.155 mmol) was treated according to General procedure F. Purification by silica gel column chromatography (pentane/EtOAc + 1% TEA, 5:1) yielded compound **s114** (0.026 g, 35%).

^1H NMR (500 MHz, $CDCl_3$) δ 7.42 – 7.24 (m, 10H), 4.73 – 4.61 (m, 2H), 4.48 (d, *J* = 11.8 Hz, 1H), 4.33 (d, *J* = 11.8 Hz, 1H), 3.99 (ddd, *J* = 9.7, 5.8, 3.9 Hz, 1H), 3.52 – 3.48 (m, 1H), 3.24 (t, *J* = 7.0 Hz, 2H), 2.40 – 2.32 (m, 1H), 2.15 – 1.93 (m, 3H), 1.83 (dd, *J* = 6.5, 3.9 Hz, 1H), 1.58 (dtd, *J* = 12.7, 7.1, 6.5, 3.7 Hz, 4H), 1.48 – 1.41 (m, 1H), 1.38 – 1.23 (m, 9H), 1.10 (d, *J* = 7.4 Hz, 3H). ^{13}C NMR (126 MHz, $CDCl_3$) δ 139.25, 138.91, 128.42 (2C), 128.41 (2C), 127.82 (2C), 127.75 (2C), 127.60, 127.53, 76.79, 71.25, 70.67, 70.32, 61.56, 51.60, 45.00, 41.03, 34.76, 29.90, 29.61, 29.19, 28.95, 28.83, 27.41, 26.80, 15.94. HRMS m/z: $[M+H]^+$ calculated for $C_{29}H_{40}N_4O_2$ 477.3224, found 477.3223.

Compound **s115**

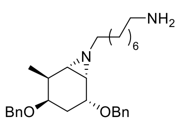

Compound **s114** (0.026 g, 0.054 mmol) was treated according to General procedure G to yield compound **s115** (0.023 g, 93%).

^1H NMR (500 MHz, $CDCl_3$) δ 7.41 – 7.37 (m, 2H), 7.37 – 7.24 (m, 8H), 4.72 – 4.60 (m, 2H), 4.47 (d, *J* = 11.8 Hz, 1H), 4.33 (d, *J* = 11.8 Hz, 1H), 3.98 (ddd, *J* = 9.7, 5.9, 3.9 Hz, 1H), 3.50 (t, *J* = 4.7 Hz, 1H), 2.70 – 2.64 (m, 2H), 2.51 – 2.26 (m, 3H), 2.13 – 1.91 (m, 3H), 1.82 (dd, *J* = 6.5, 3.9 Hz, 1H), 1.62 – 1.53 (m, 2H), 1.49 – 1.39 (m, 3H),

1.35 – 1.23 (m, 9H), 1.09 (d, *J* = 7.4 Hz, 3H). ^{13}C NMR (126 MHz, $CDCl_3$) δ 139.26, 138.92, 128.42 (2C), 128.40 (2C), 127.81 (2C), 127.76 (2C), 127.59, 127.52, 76.79, 71.26, 70.66, 70.30, 61.63, 44.99, 42.14, 40.99, 34.78, 33.43, 29.94, 29.73, 29.51, 28.85, 27.48, 26.95, 15.91.

Compound **s116**

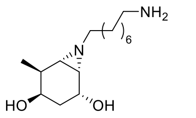

Compound **s115** (0.012 g, 0.027 mmol) was treated according to General procedure H to yield compound **s116** (0.007 g, 97%).

^{1}H NMR (400 MHz, MeOD) δ 4.21 (ddd, *J* = 10.1, 6.1, 4.2 Hz, 1H), 3.71 (t, *J* = 4.6 Hz, 1H), 2.85 – 2.76 (m, 2H), 2.27 (ddt, *J* = 35.6, 11.6, 7.4 Hz, 2H), 1.91 – 1.72 (m, 3H), 1.65 – 1.53 (m, 4H), 1.42 – 1.29 (m, 12H), 1.09 (d, *J* = 7.4 Hz, 3H). ^{13}C NMR (126 MHz, MeOD) δ 70.25, 64.54, 62.15, 46.31, 44.65, 41.20, 36.04, 35.73, 30.59, 30.51, 30.24, 29.92, 28.33, 27.54, 16.24.

Compound **49**

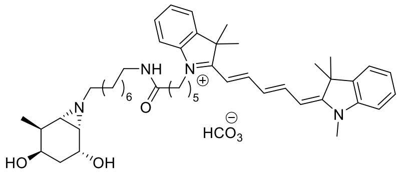

Compound **s116** (0.002 g, 0.0074 mmol) was treated according to General procedure I to yield compound **49** (0.00035 g, 6%).

^{1}H NMR (850 MHz, MeOD) δ 8.28 – 8.23 (m, 2H), 7.51 – 7.48 (m, 2H), 7.44 – 7.39 (m, 2H), 7.32 – 7.24 (m, 4H), 6.63 (t, *J* = 12.3 Hz, 1H), 6.30 – 6.26 (m, 2H), 4.20 (ddd, *J* = 10.1, 6.1, 4.1 Hz, 1H), 4.11 (t, *J* = 7.5 Hz, 2H), 3.70 (d, *J* = 5.3 Hz, 1H), 3.63 (s, 3H), 3.12 (t, *J* = 7.2 Hz, 2H), 2.31 – 2.28 (m, 1H), 2.21 – 2.17 (m, 3H), 1.86 – 1.79 (m, 4H), 1.76 (ddd, *J* = 7.5, 4.4, 2.1 Hz, 1H), 1.73 (s, 12H), 1.71 – 1.67 (m, 2H), 1.58 – 1.54 (m, 2H), 1.49 – 1.43 (m, 4H), 1.38 (dt, *J* = 6.6, 1.2 Hz, 1H), 1.36 – 1.27 (m, 8H), 1.07 (d, *J* = 7.5 Hz, 3H). ^{13}C NMR (214 MHz, MeOD) δ 175.69, 175.37, 174.70, 155.55, 144.25, 143.58, 142.65, 142.51, 129.77, 129.75, 126.61, 126.28, 126.25, 123.43, 123.30, 112.05, 111.84, 104.35, 104.29, 70.25, 64.53, 62.16, 50.56, 50.51, 46.30, 44.76, 44.63, 40.40, 36.69, 36.03, 35.72, 31.47, 30.70, 30.62, 30.40, 30.37, 28.41, 28.20, 27.99, 27.93, 27.79, 27.36, 26.56, 16.26. HRMS m/z: $[M]^+$ calculated for $C_{47}H_{67}N_4O_3$ 735.5208, found 735.5219.

Compound **56**

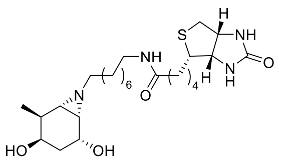

Compound **s116** (0.0025 g, 0.009 mmol) was treated according to General procedure J to yield compound **56** (0.0011 g, 24%).

^{1}H NMR (850 MHz, MeOD) δ 4.49 (ddd, *J* = 7.9, 5.0, 1.0 Hz, 1H), 4.30 (dd, *J* = 7.9, 4.5 Hz, 1H), 4.21 (ddd, *J* = 10.1, 6.0, 4.2 Hz, 1H), 3.71 (t, *J* = 4.9 Hz, 1H), 3.20 (ddd, *J* = 9.1, 5.8, 4.5 Hz, 1H), 3.19 – 3.12 (m, 2H), 2.93 (dd, *J* = 12.7, 5.0 Hz, 1H), 2.71 (d, *J* = 12.7 Hz, 1H), 2.32 (dt, *J* = 11.6, 7.3 Hz, 1H), 2.24 – 2.17 (m, 3H), 1.87 – 1.81 (m, 2H), 1.80 – 1.55 (m, 8H), 1.53 – 1.47 (m, 2H), 1.47 – 1.39 (m, 3H), 1.39 – 1.28 (m, 8H), 1.09 (d, *J* = 7.5 Hz, 3H). ^{13}C NMR (214 MHz, MeOD) δ 175.97, 166.12, 70.25, 64.57, 63.40, 62.18, 61.63, 57.03, 46.37, 44.63, 41.05, 40.38, 36.83, 36.04, 35.73, 30.66, 30.63, 30.42, 30.37, 29.79, 29.52, 28.41, 27.96, 26.96, 16.19. HRMS m/z: $[M+H]^+$ calculated for $C_{25}H_{44}N_4O_4S$ 497.3156, found 497.3154.

Biochemical experiments

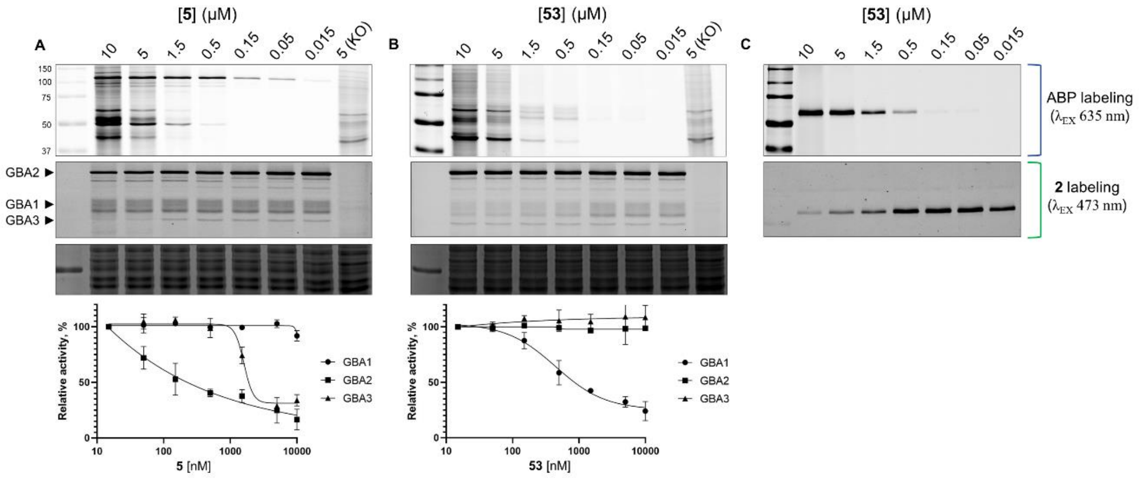

Figure S1. cABPP experiments with **2** (300 nM) in GBA2/GBA3 overexpressing HEK293T cell lysates with (A) ABP **5**; (B) ABP **53**; (C) ABP **53** in rhGBA1. KO: lysates of GBA1/GBA2 KO HEK293T cells were incubated with the specified ABP concentration. Lower panels: Coomassie brilliant blue (CBB) loading controls.

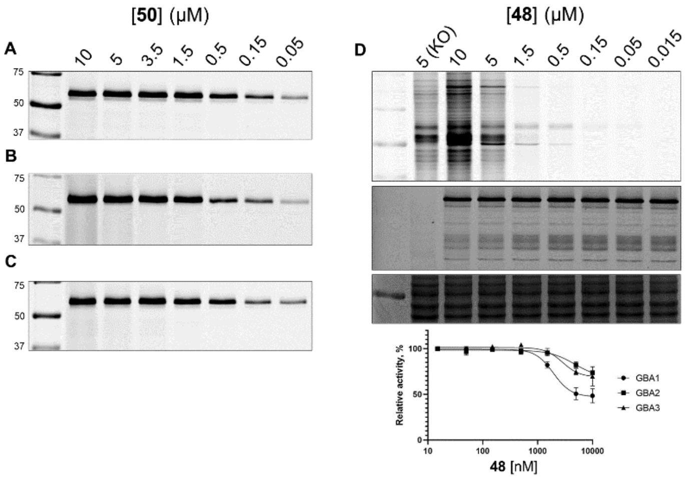

Figure S2. ABP labeling with **50** in rhGBA1 at (A) pH 5.2, supplemented with 0.1% (v/v) Triton X-100, and 0.2% (w/v) sodium taurocholate; (B) pH 7.0; (C) pH 6.0; (D) ABP labeling with **48** in lysates of GBA2/GBA3 overexpressing HEK293T cells followed by incubation with **2** (300 nM). KO: lysates of GBA1/GBA2 KO HEK293T cells were incubated with the specified ABP concentration. Lower panel: Coomassie brilliant blue (CBB) loading control.

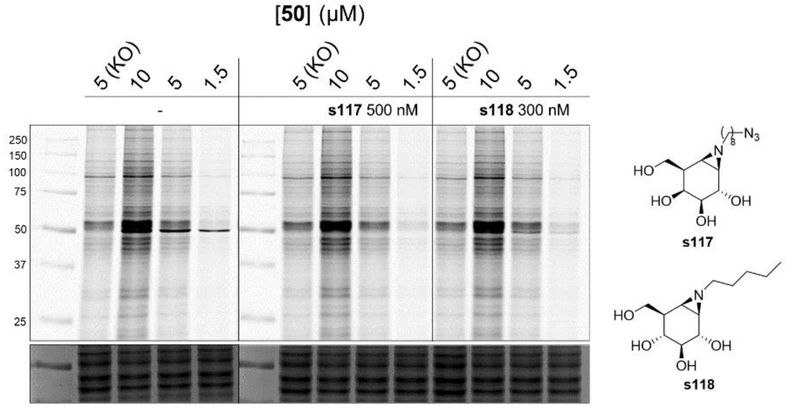

Figure S3. cABPP of **50** with galactosidase inhibitor (**s117**) and glucosidase inhibitor (**s118**). Lysates of GBA2/GBA3 overexpressing HEK293T cells were pre-incubated for 30 minutes with vehicle, **s117** (500 nM) or **s118** (300 nM) before incubation with a range of **50** concentrations. KO: lysates of GBA1/GBA2 KO HEK293T cells were incubated with the specified ABP concentration. Lower panels: Coomassie brilliant blue (CBB) loading controls.

Cell culture and generation of cells genetically modified in β-glucosidase expression

Cell culturing was performed exactly as described previously.[6] HEK293T cells with both overexpressed human GBA2 and human GBA3 were generated exactly as described previously.[6] GBA1/GBA2 knockout HEK293T cells were generated, analyzed for lack of GBA1/GBA2 expression, and used for transfection with either GBA2 or GBA3 constructs exactly as described previously.[6,16]

IC_{50} determination using a fluorogenic substrate assay

All assays were performed in 96-well plates (Greiner, black, flat-bottomed) at 37 °C. For GBA1, 3.5 ng of rhGBA1 (Imiglucerase, Sanofi Genzyme) was dissolved in 12.5 µL McIlvaine buffer (150 mM citric acid-Na_2HPO_4, pH 5.2), supplemented with 0.1% (v/v) Triton X-100, and 0.2% (w/v) sodium taurocholate, 0.1% (w/v) bovine serum albumin (BSA). Lysates of GBA1/GBA2 KO HEK293T cells overexpressing GBA2 or GBA3 were prepared by sonication on ice with Polytron PT 1300D sonicator (Kinematica) in potassium phosphate buffer (25 mM K_2HPO_4/KH_2PO_4, pH 6.5, supplemented with 0.1% (v/v) Triton X-100 and protease inhibitor cocktail (EDTA-free, Roche)). The lysates were diluted to 12.5 µL in McIlvaine buffer (150 mM, pH 5.8 for GBA2 or pH 6.0 for GBA3) supplemented with 0.1% (w/v) bovine serum albumin (BSA). The enzymes were incubated with 12.5 µL solution of inhibitors in McIlvaine buffer (150 mM, pH appropriate for each enzyme, final concentration 1% DMSO) at 37 °C for 30 minutes (β-configured aziridines and ABP **3**) or 3 hours (epoxides and α-configured aziridines). This was followed by incubation of the samples with 100 µL of fluorogenic substrate mixtures for 30 minutes. The substrate mixtures diluted in McIlvaine buffer (supplemented with 0.1% (w/v) BSA) at optimal pH for each enzyme were as follows: GBA1, 3.75 mM 4-MU-β-D-glucopyranoside (Glycosynth) at pH 5.2, supplemented with 0.2% (w/v) sodium taurocholate, 0.1% (v/v) Triton X-100, 0.1% (w/v) bovine serum albumin (BSA); GBA2, 4-MU-β-D-glucopyranoside (3.75 mM) at pH 5.8; GBA3, 4-MU-β-D-glucopyranoside (3.75 mM) at pH 6.0. After stopping the enzyme reaction with 200 µL 1 M NaOH-glycine (pH 10.3), 4-methylumbelliferone fluorescence was measured with a fluorimeter LS55 (Perkin Elmer) with λ_{EX} 366 nm and λ_{EM} 445 nm. Enzyme activities were determined by subtraction of background (measured for incubations without enzyme), normalized with enzyme activity of the control sample (vehicle incubation) and curve-fitted to inhibitor concentrations using GraphPad Prism 9.0 software by the [inhibitor] vs. response - variable slope (four parameters) method to determine IC_{50} values. Displayed values represent mean values from triplicate or duplicate experiments (n = 3 biological replicates) and error ranges indicate standard deviation (SD).

ABPP assays

Cell lysates were prepared by sonication as described above and 20 µg total protein per experiment was used. For assays with recombinant proteins, 10 ng of rhGBA1 or rhGBA3 (purchased from Bio-Techne R&D Systems, catalog#: 5969-GH-010) per experiment was used. The enzymes were diluted in McIlvaine buffer (150 mM, pH 6.0, 20 µL total volume), exposed to a solution of an ABP in McIlvaine buffer (150 mM, pH 6.0, 5 µL, final concentration 1% DMSO) and incubated at 37 °C for 30 minutes. For competitive ABPP, after incubation with the inhibitor at 37 °C for 30 minutes (or 3 hours – Figure 4B), the samples were incubated with vehicle or **2** (300 nM) in McIlvaine buffer (150 mM, pH 6.0, 5 µL, final concentration 1% DMSO) at 37 °C for 30 minutes. After ABP incubation, proteins were denatured by boiling the samples with 5× Laemmli buffer (50% (v/v) 1 M Tris-HCl, pH 6.8, 50% (v/v) 100% glycerol, 10% (w/v) DTT, 10% (w/v) SDS, 0.01% (w/v) bromophenol blue) for 5 minutes at 98 °C and separated on 10% (w/v) SDS-PAGE gels. Wet gel slabs were scanned on fluorescence using the Typhoon FLA 9500 (GE Healthcare) at λ_{EX} 635 nm and $\lambda_{EM} \geq 665$ nm for Cy5 fluorescent ABPs and λ_{EX} 473 nm and $\lambda_{EM} \geq 510$ nm for competition assays with BODIPY fluorescent ABP **2**. ABP-emitted fluorescence was quantified using ImageQuant software (GE Healthcare), normalized and curve-fitted using GraphPad Prism 9.0 software. Displayed values represent mean values from triplicate or duplicate experiments and error bars indicate standard deviation (SD). After fluorescence scanning, SDS-PAGE gels were stained for total protein loading with Coomassie G250 and scanned on a ChemiDoc MP imager (Bio-Rad).

IC$_{50}$ values determination graphs

Y axis – relative fluorescence intensity, X axis – inhibitor concentration [nM].

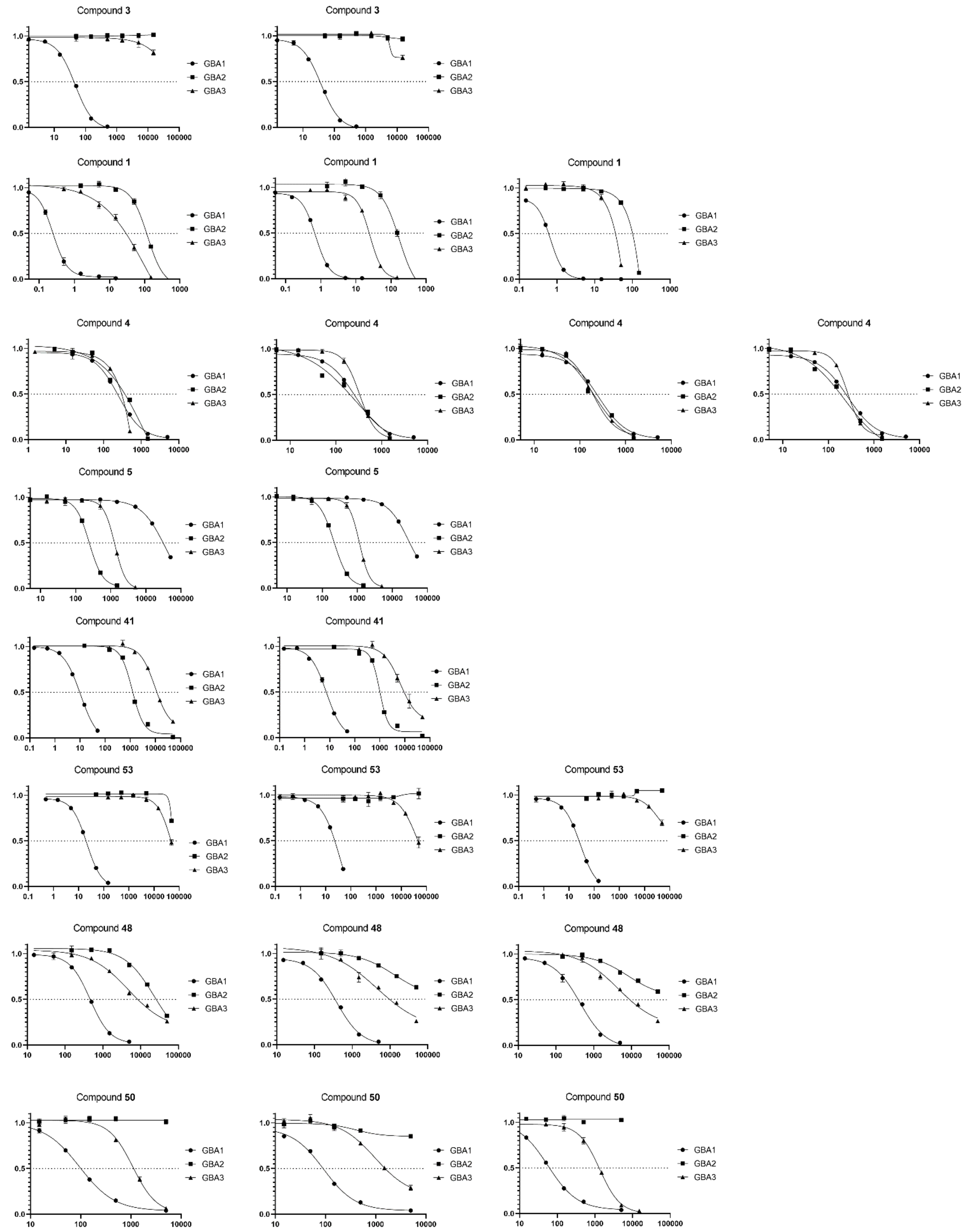

NMR spectra

Compound **7** (α-anomer) ^{1}H NMR spectrum

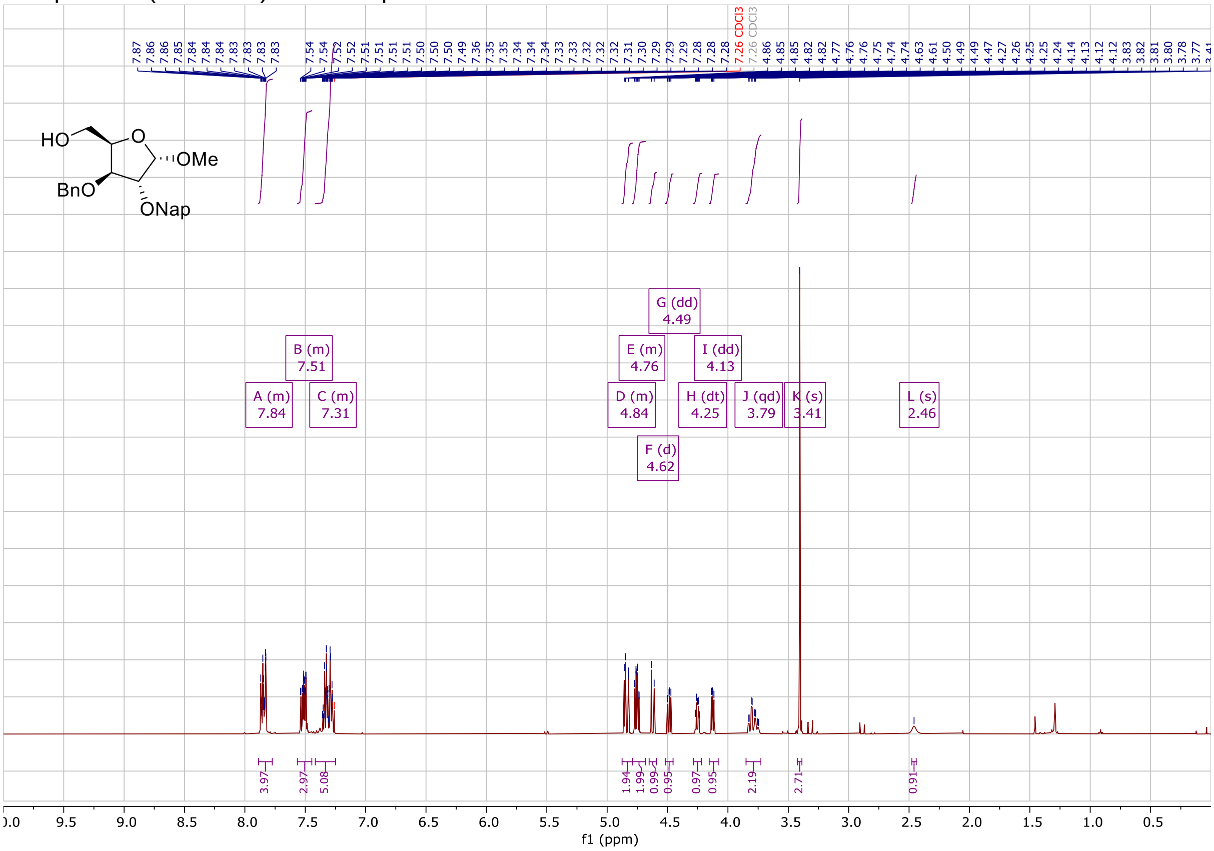

Compound **7** (α-anomer) ^{13}C NMR APT spectrum

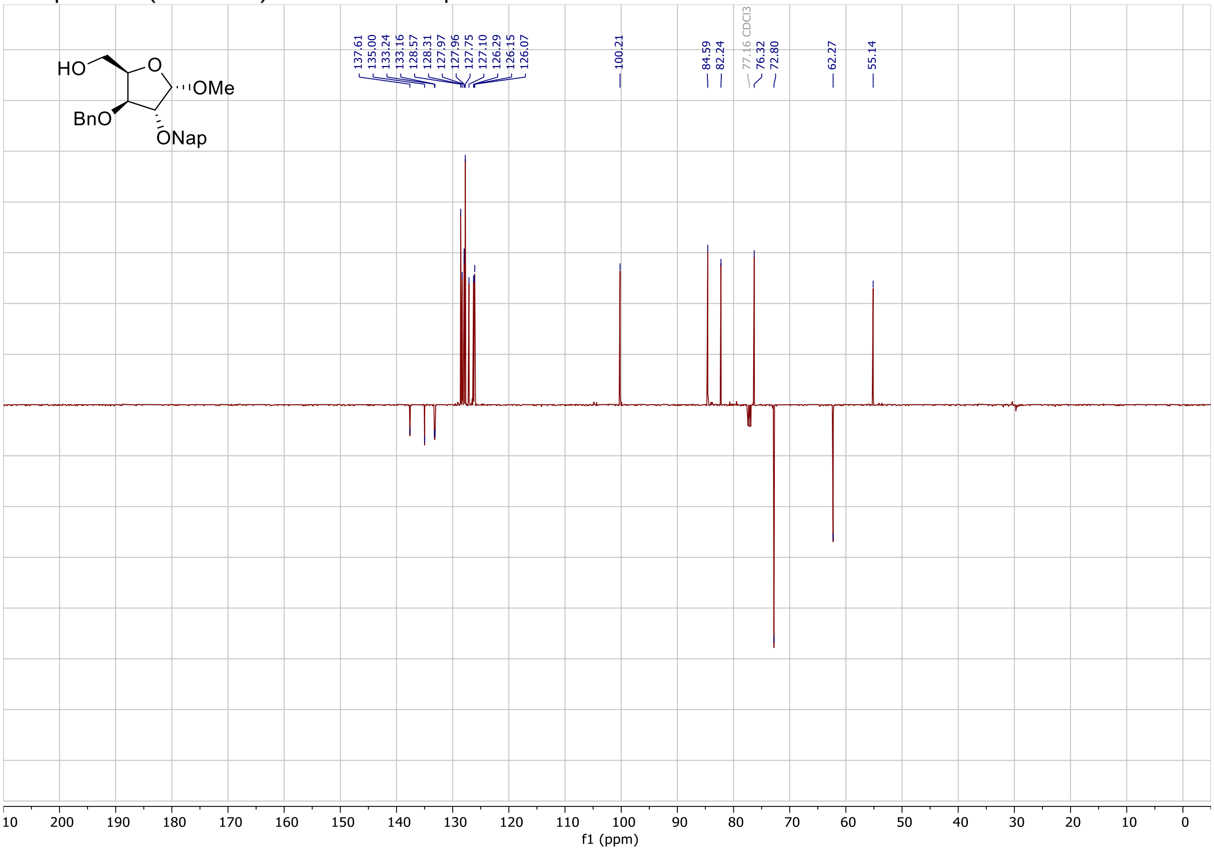

Compound **7** (β-anomer) ^{1}H NMR spectrum

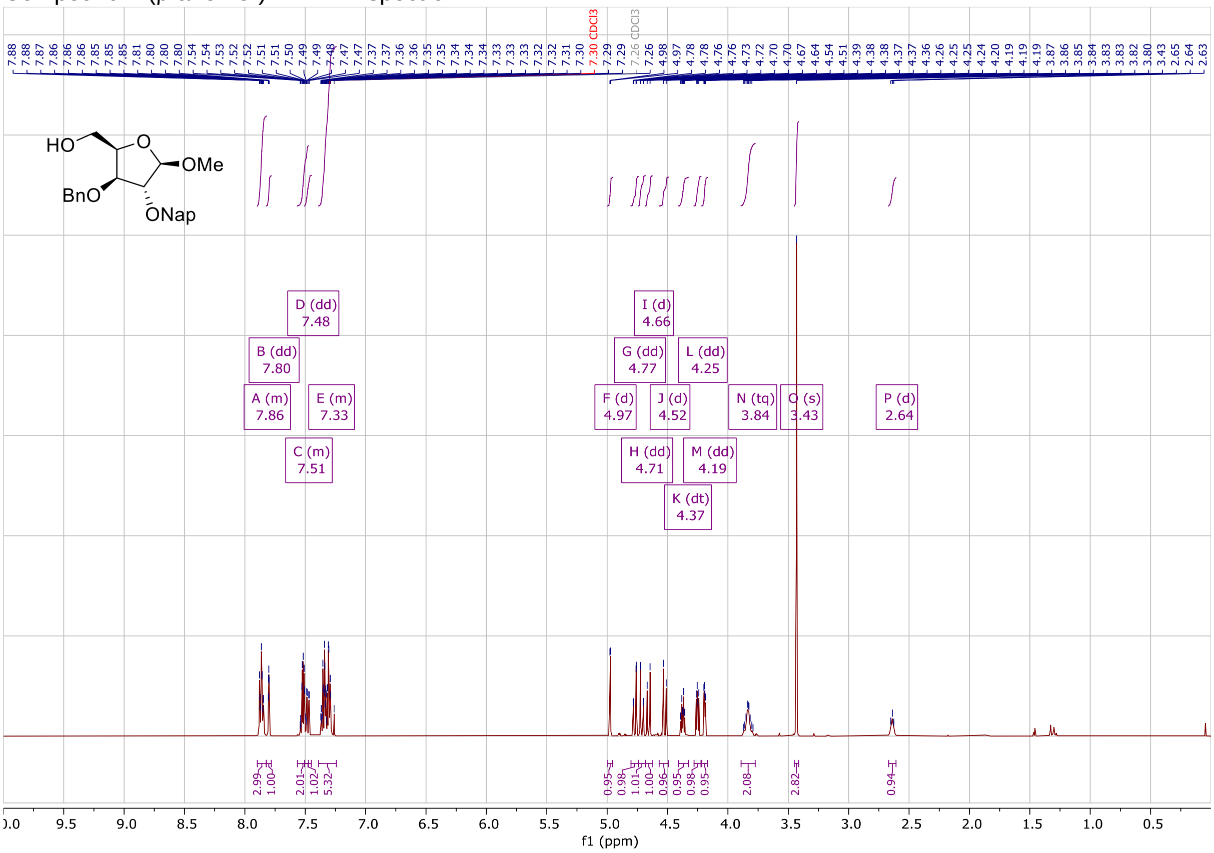

Compound **7** (β-anomer) ^{13}C NMR APT spectrum

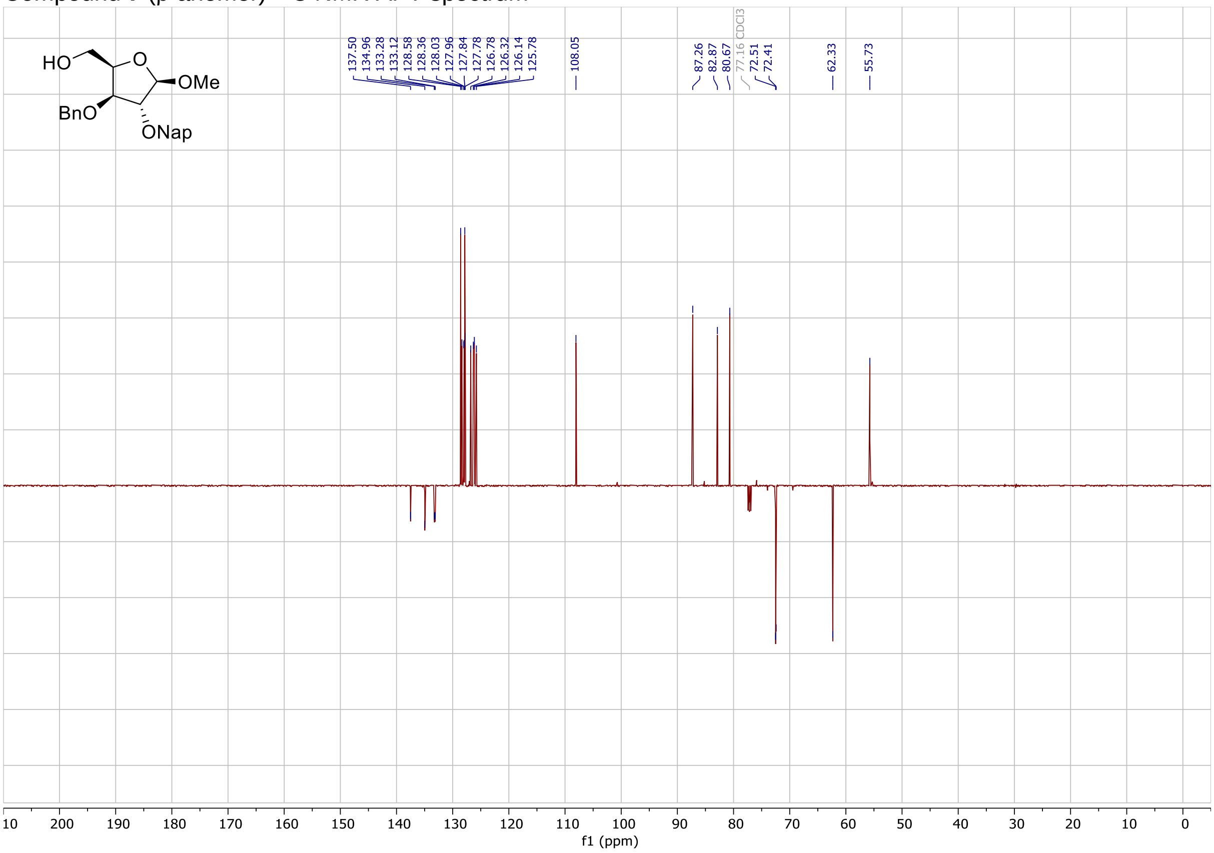

Compound **8** (α-anomer) ^{1}H NMR spectrum

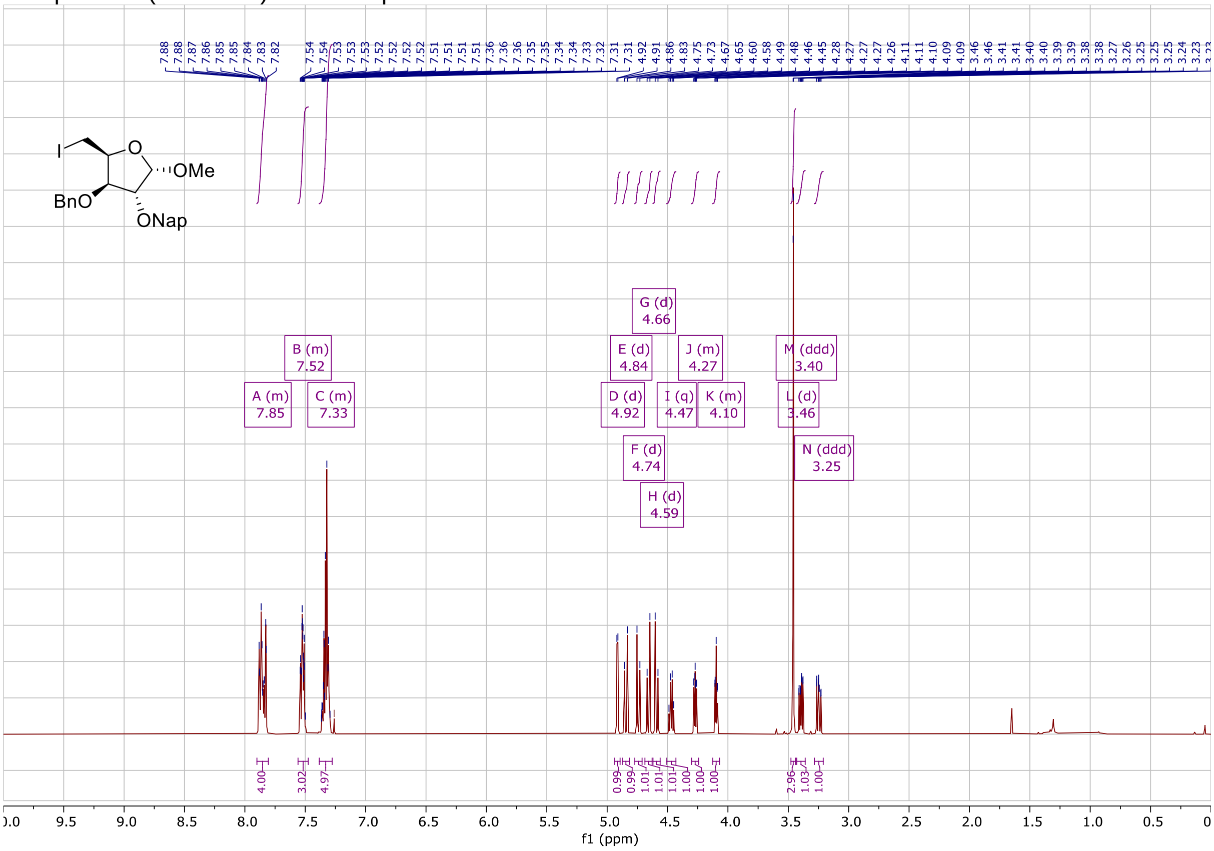

Compound **8** (α-anomer) ^{13}C NMR APT spectrum

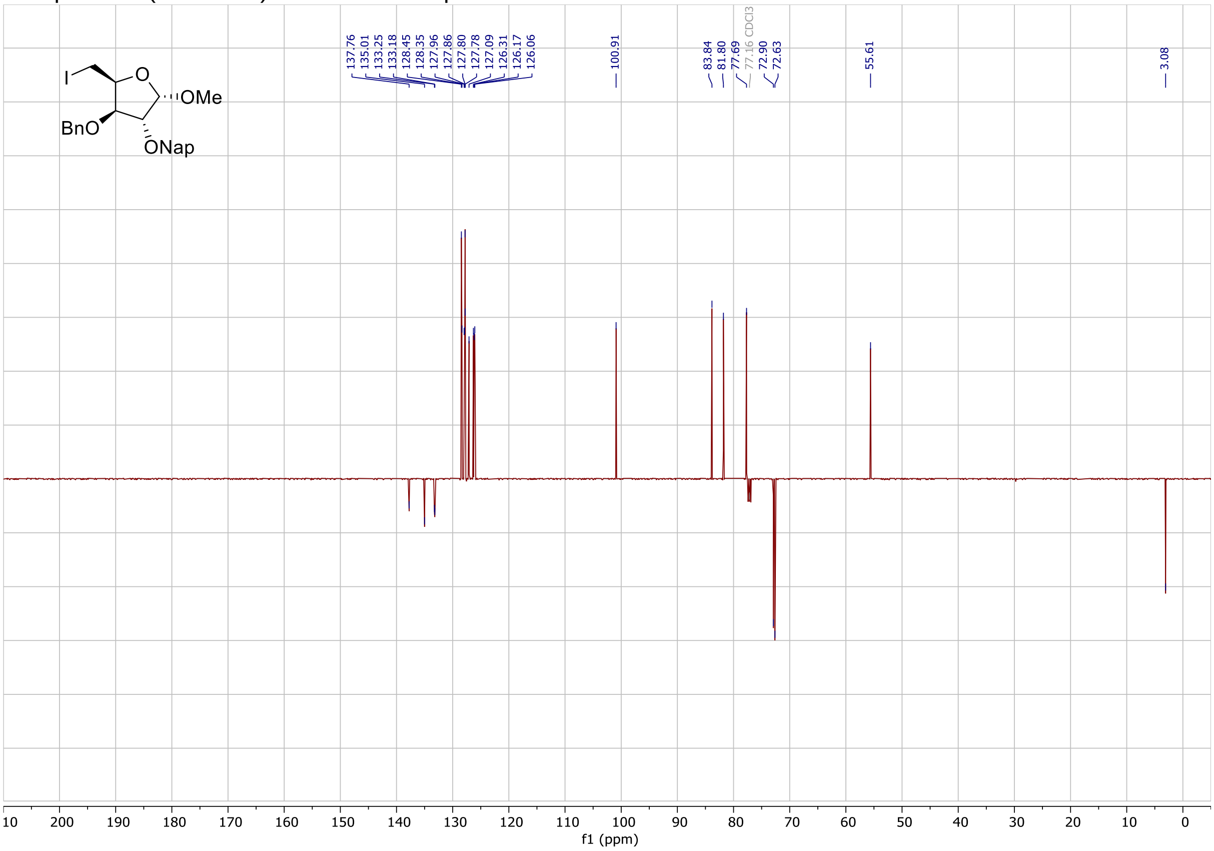

Compound **8** (β-anomer) ^{1}H NMR spectrum

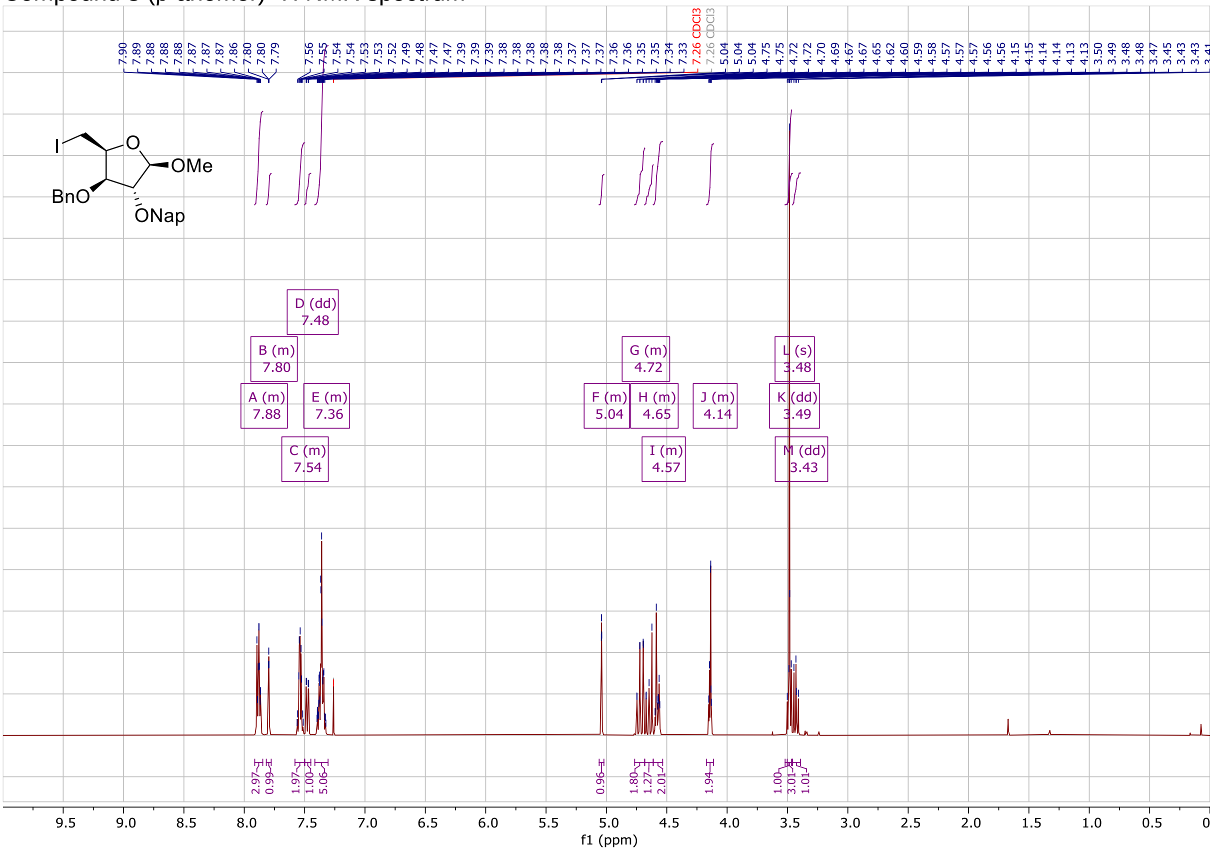

Compound **8** (β-anomer) ^{13}C NMR APT spectrum

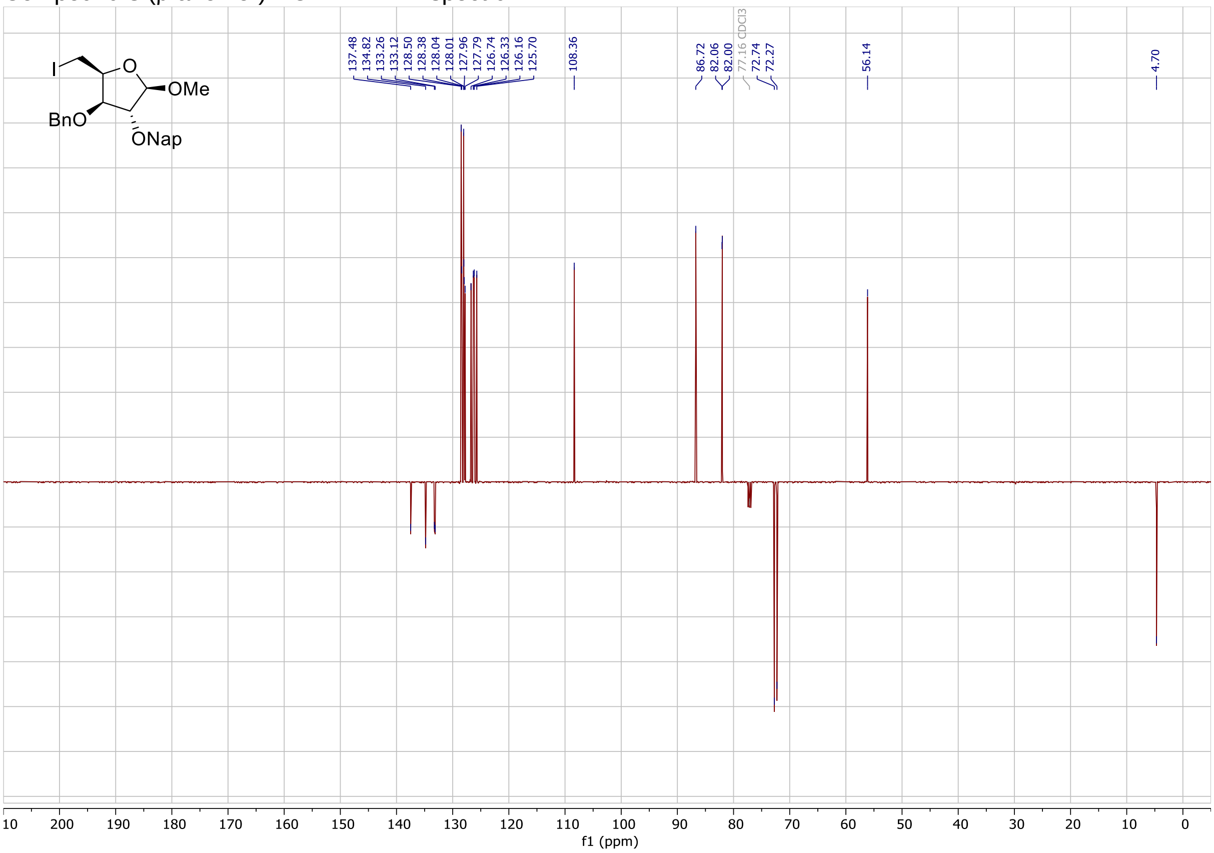

Compound **9** ^{1}H NMR spectrum

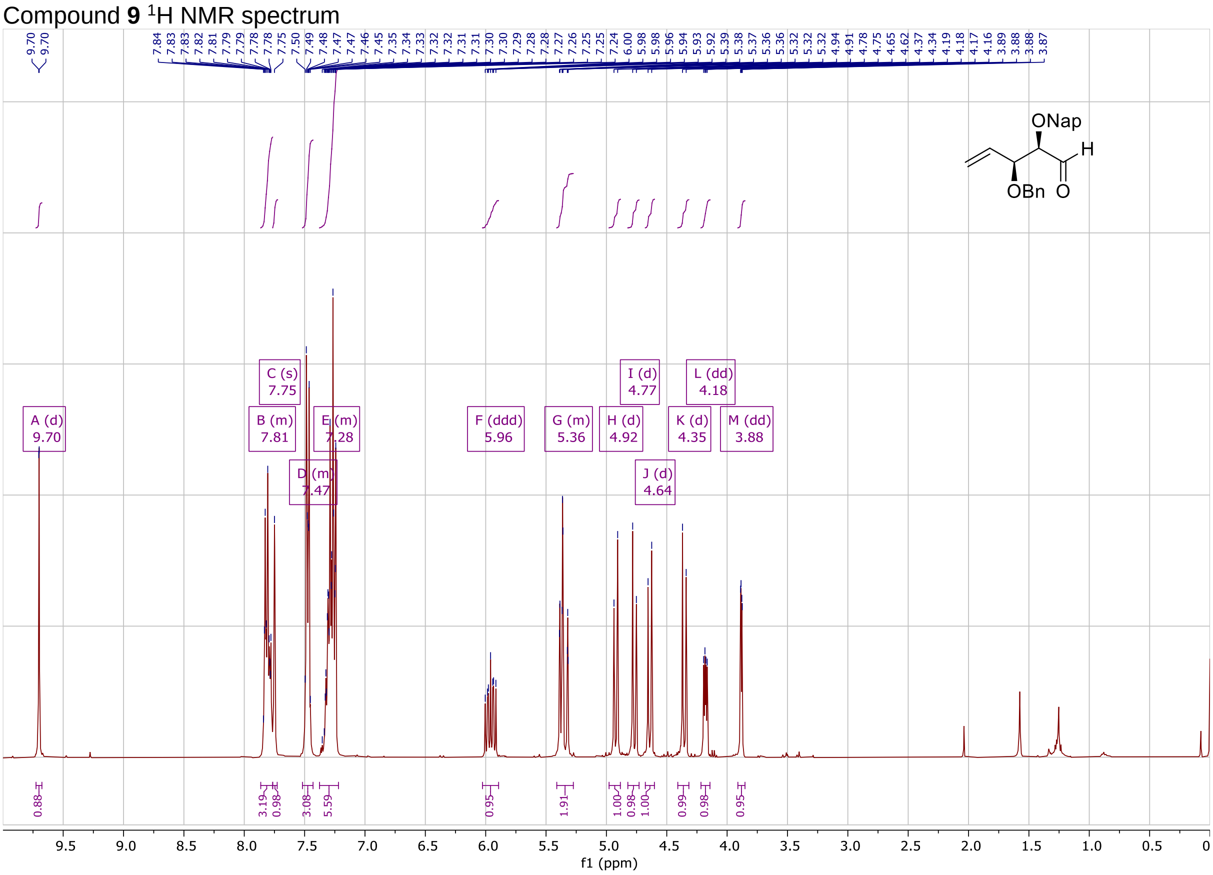

Compound **9** ^{13}C NMR APT spectrum

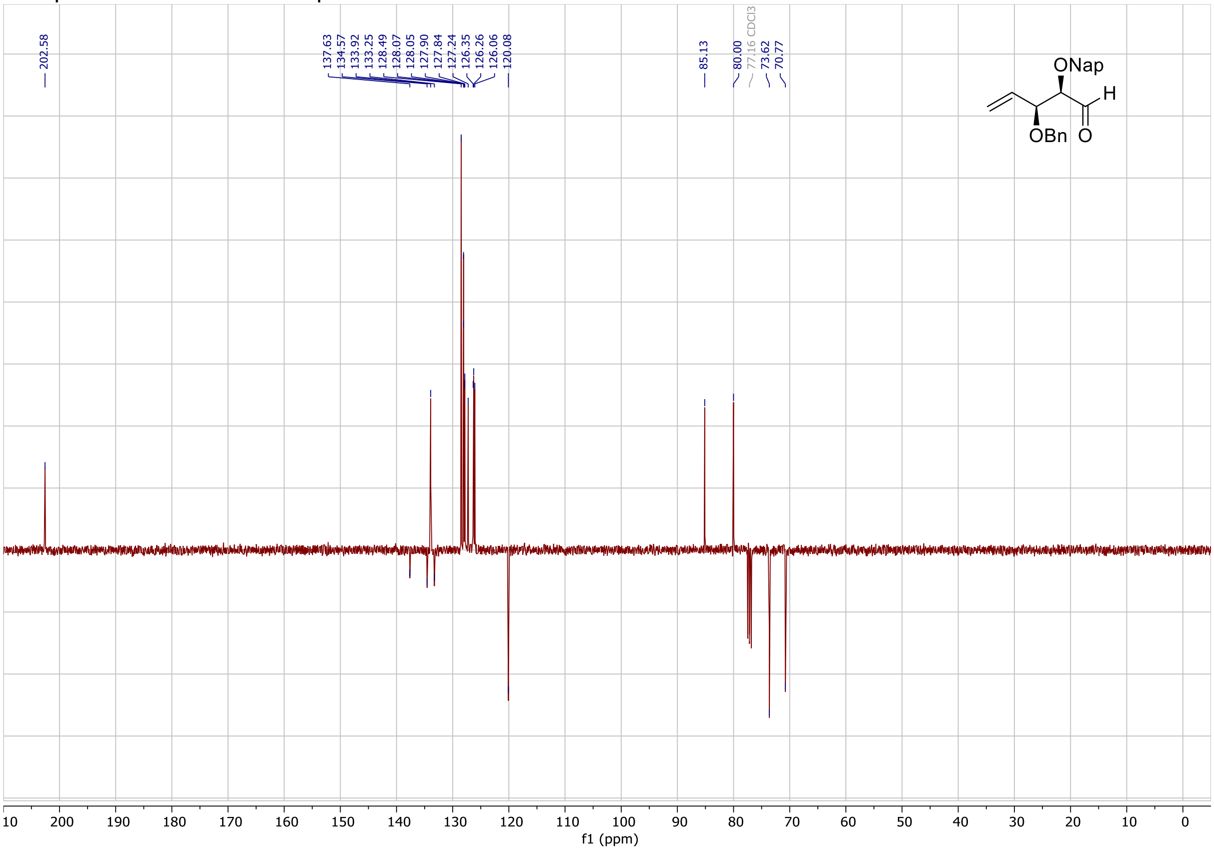

Compound **10** ^{1}H NMR spectrum

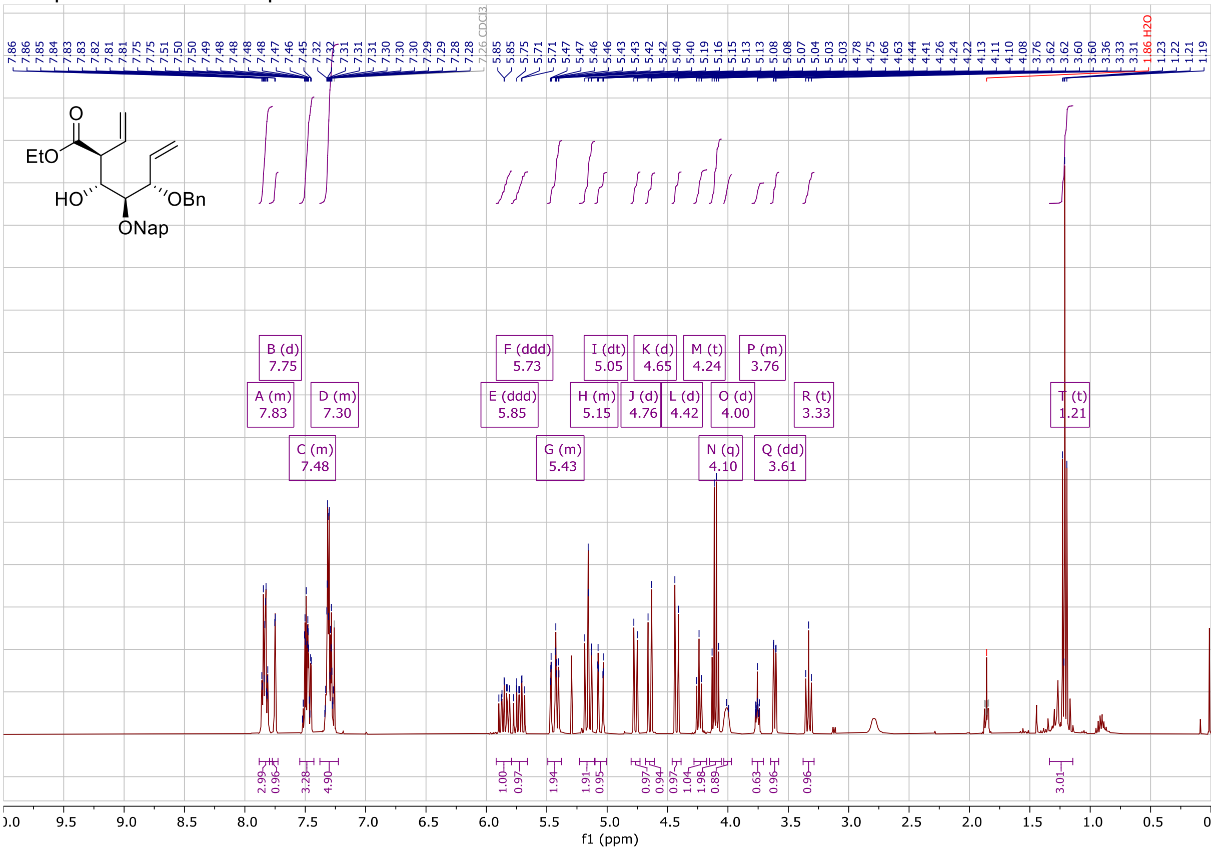

Compound **10** ^{13}C NMR APT spectrum

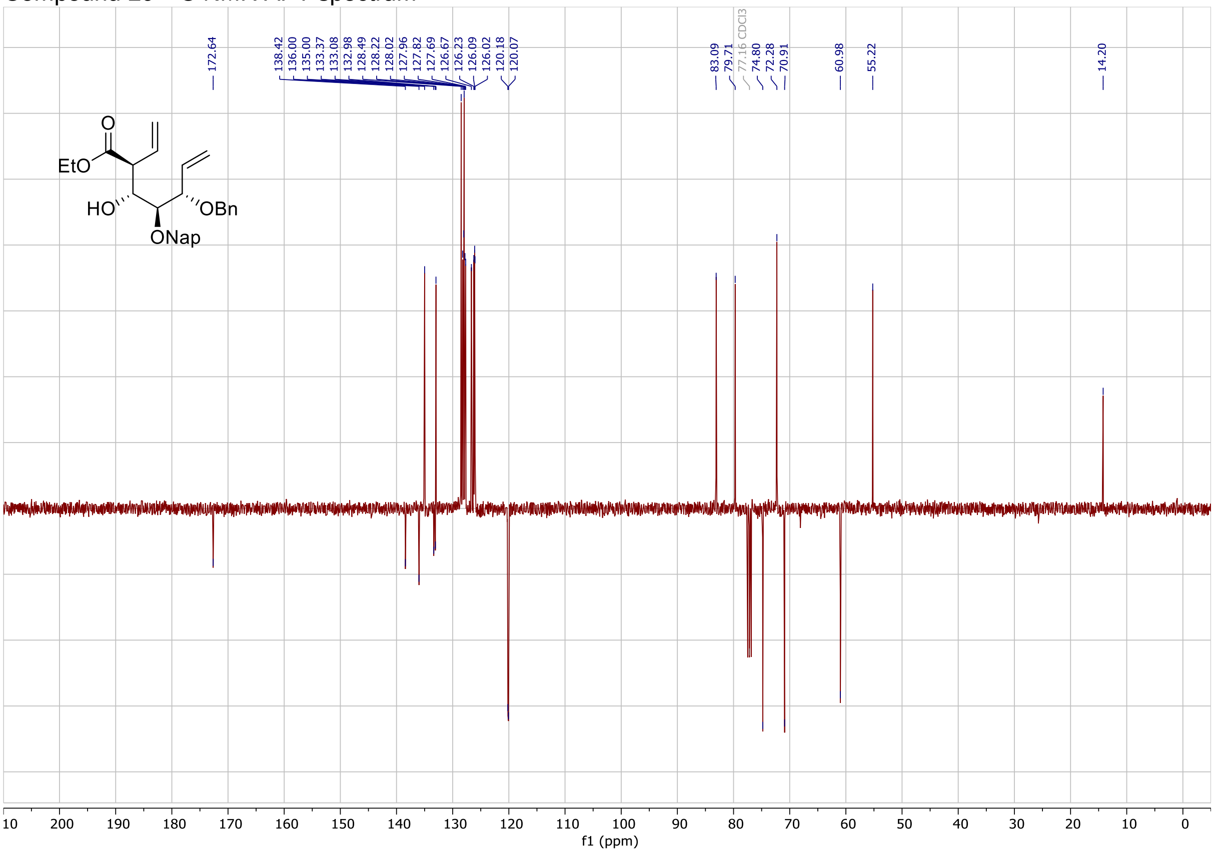

Compound **11** ^{1}H NMR spectrum

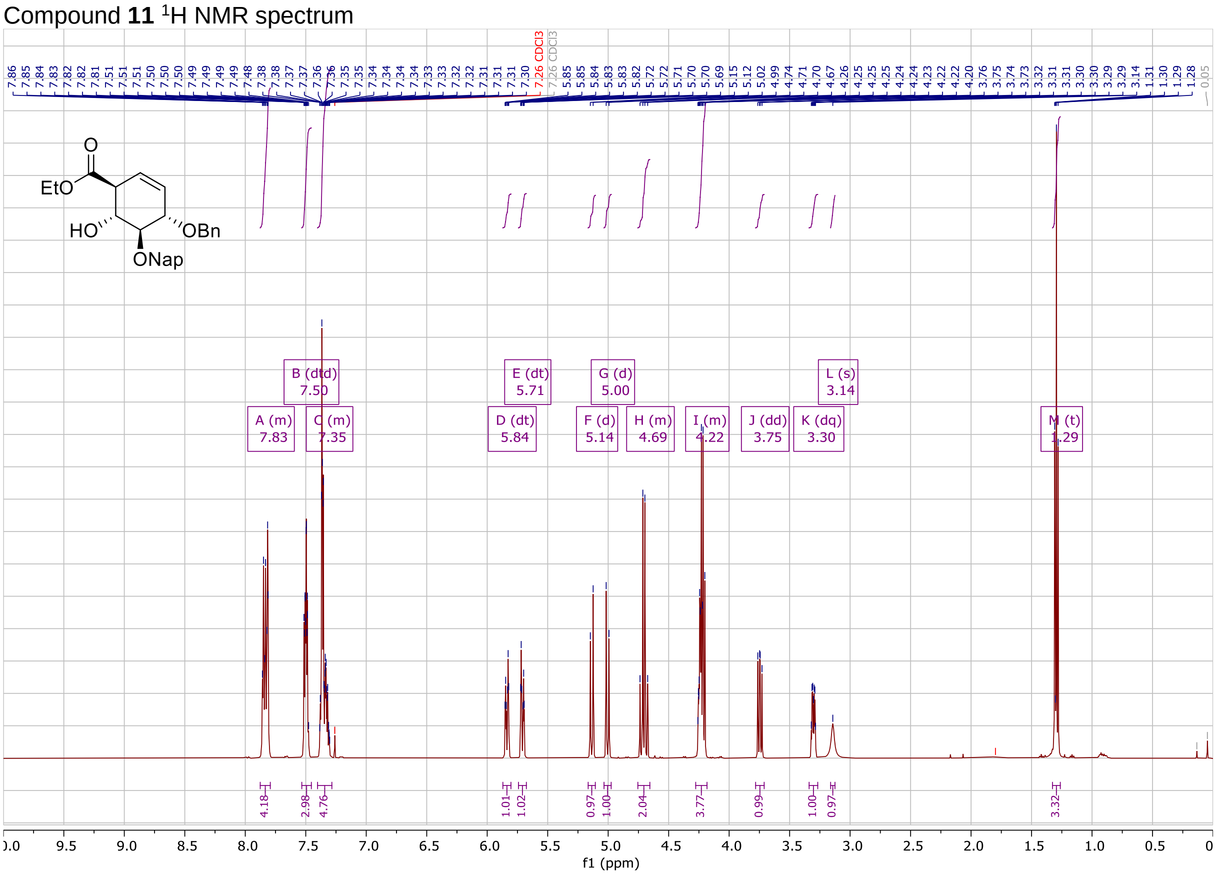

Compound **11** ^{13}C NMR APT spectrum

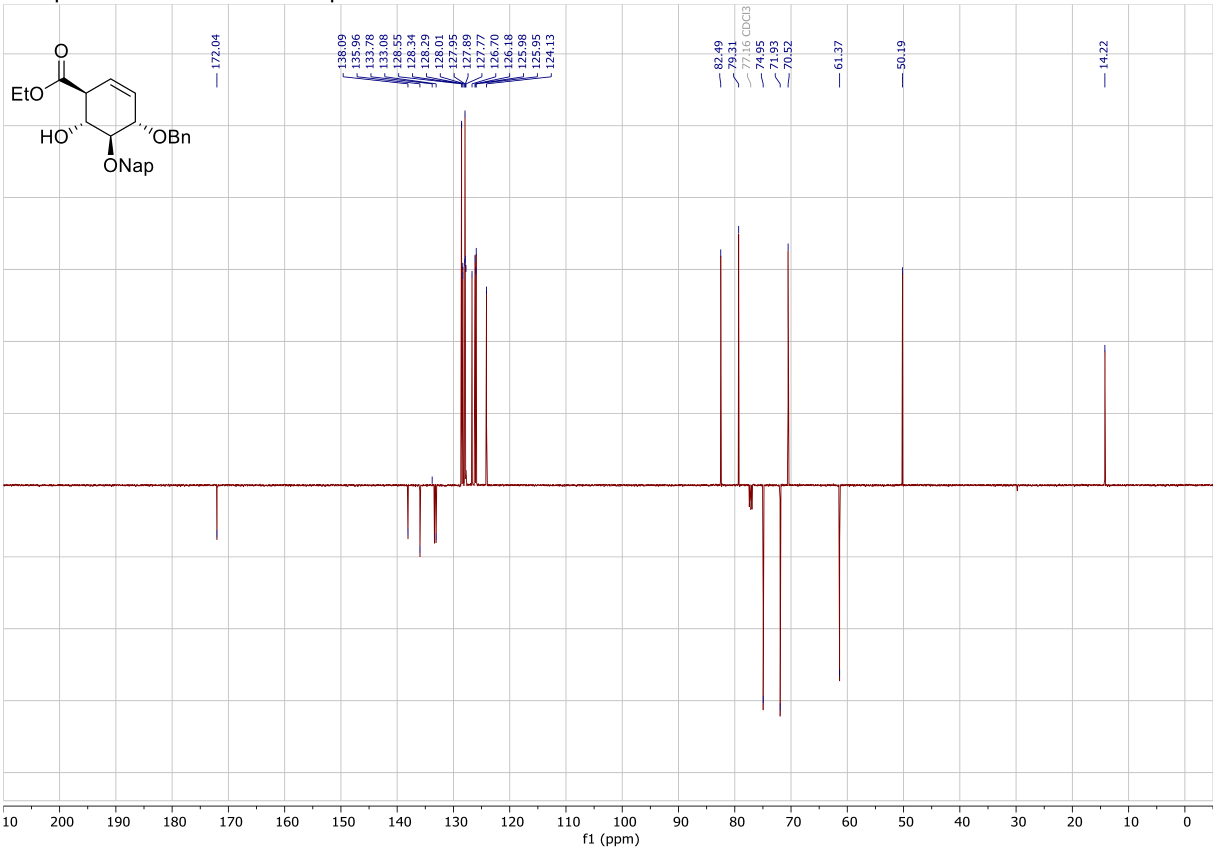

Compound **11** ^1H-^1H COSY spectrum

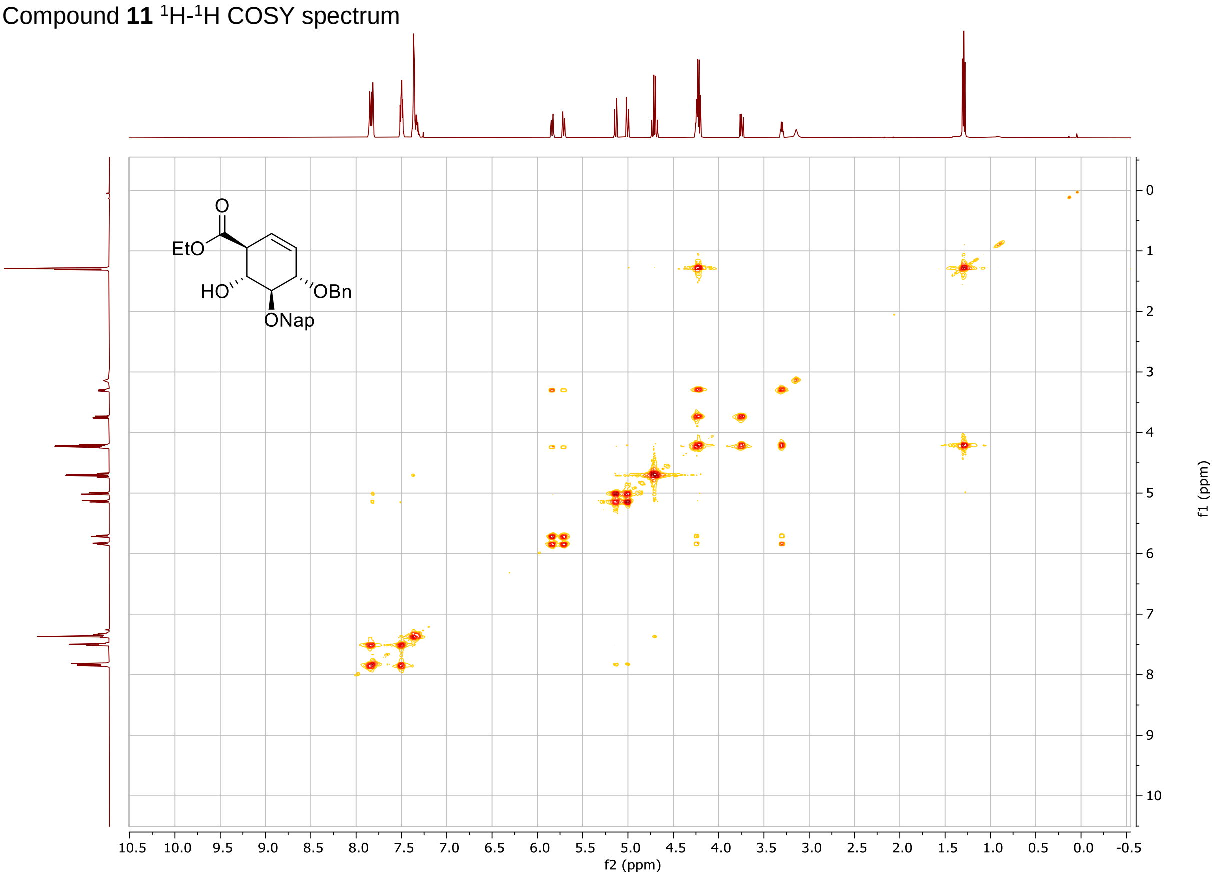

Compound **11** ^1H-^{13}C HSQC spectrum

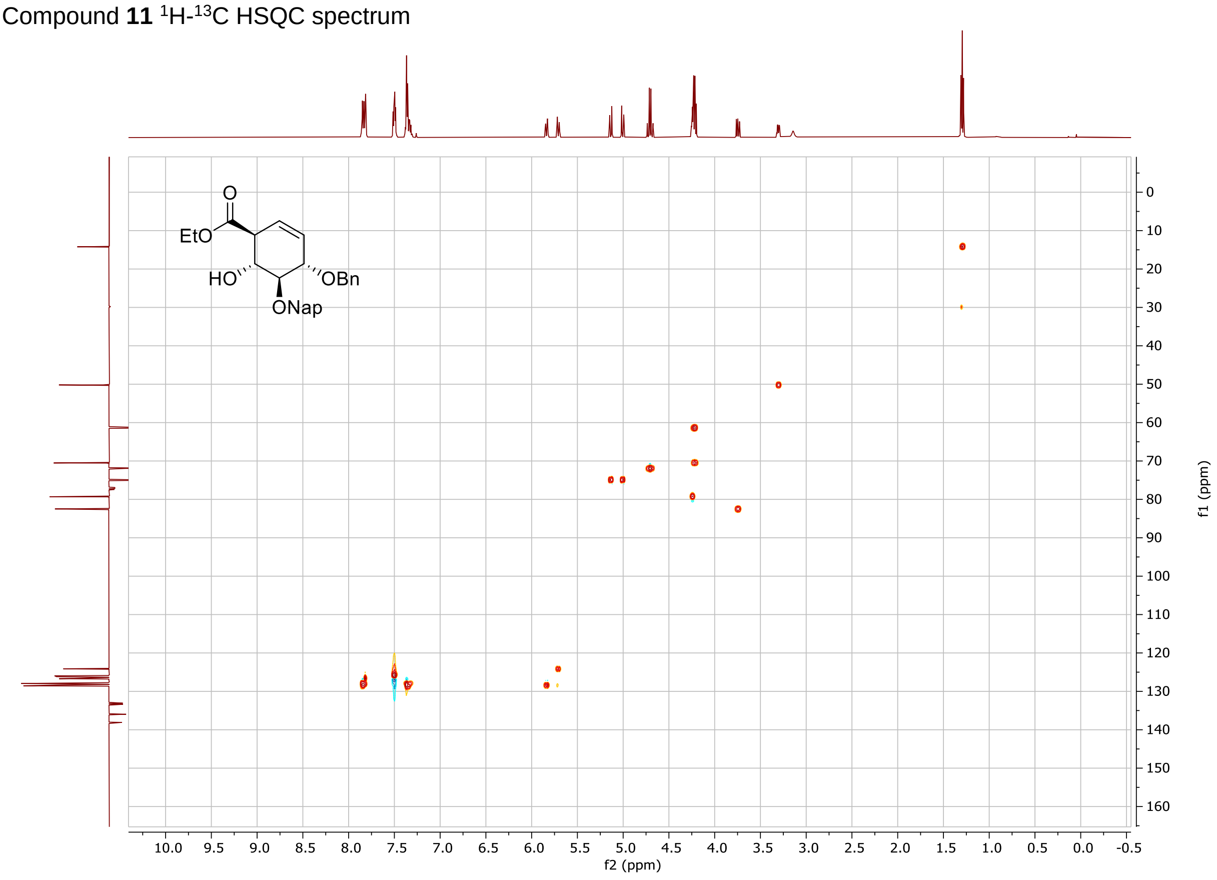

Compound **11** ^{1}H-^{1}H NOESY spectrum

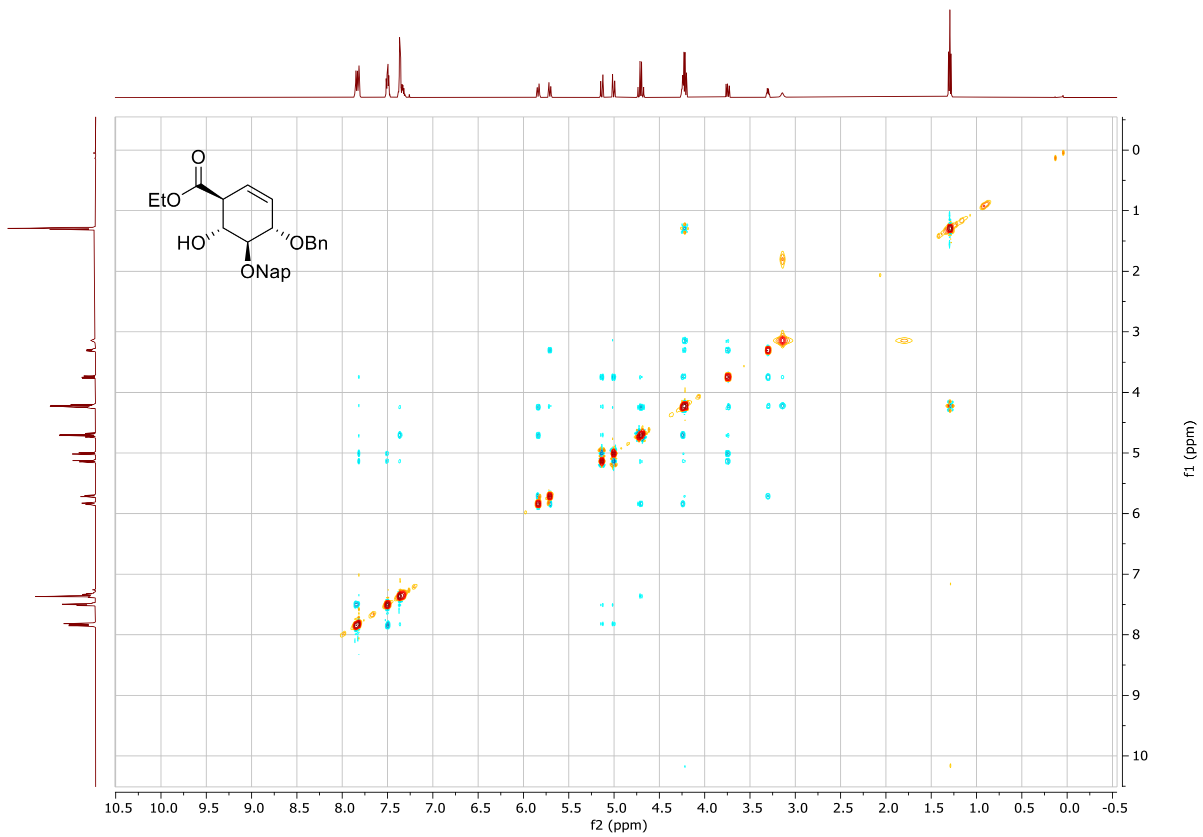

Compound **11** ^{1}H-^{13}C HMBC spectrum

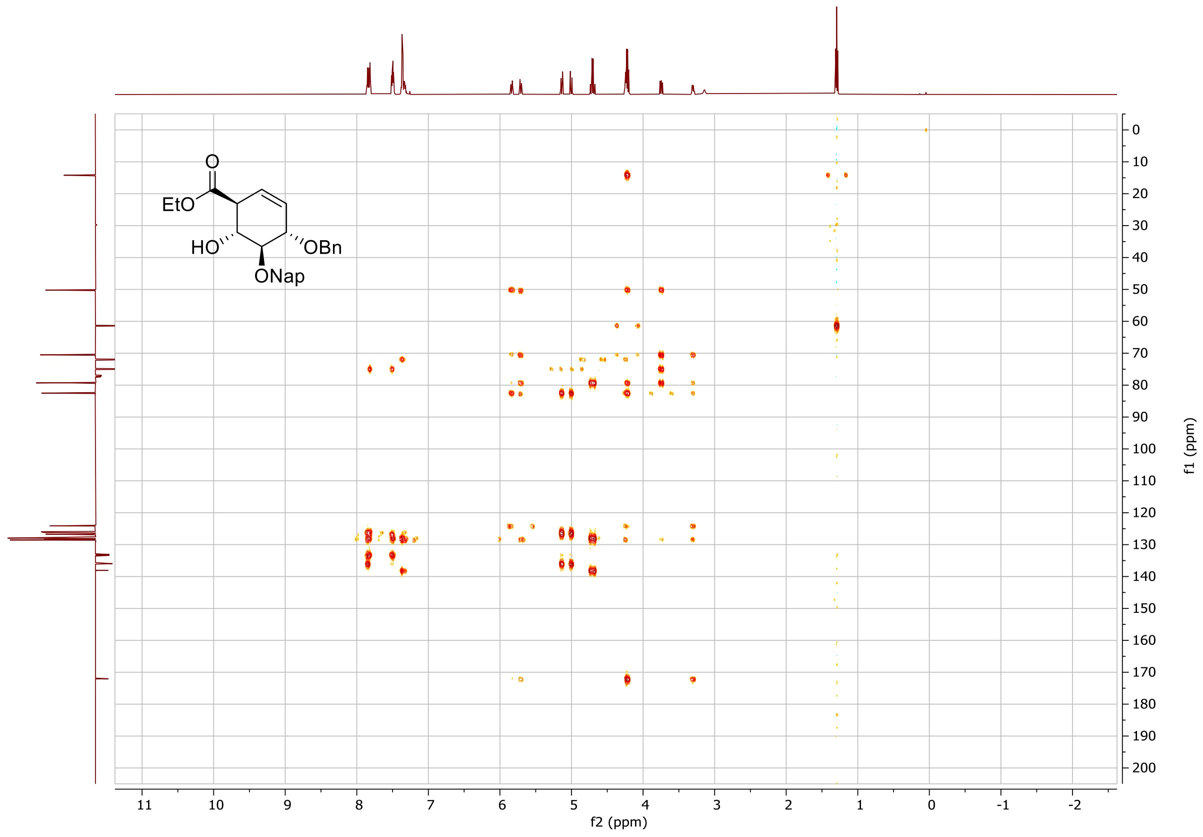

Compound **12** ^{1}H NMR spectrum

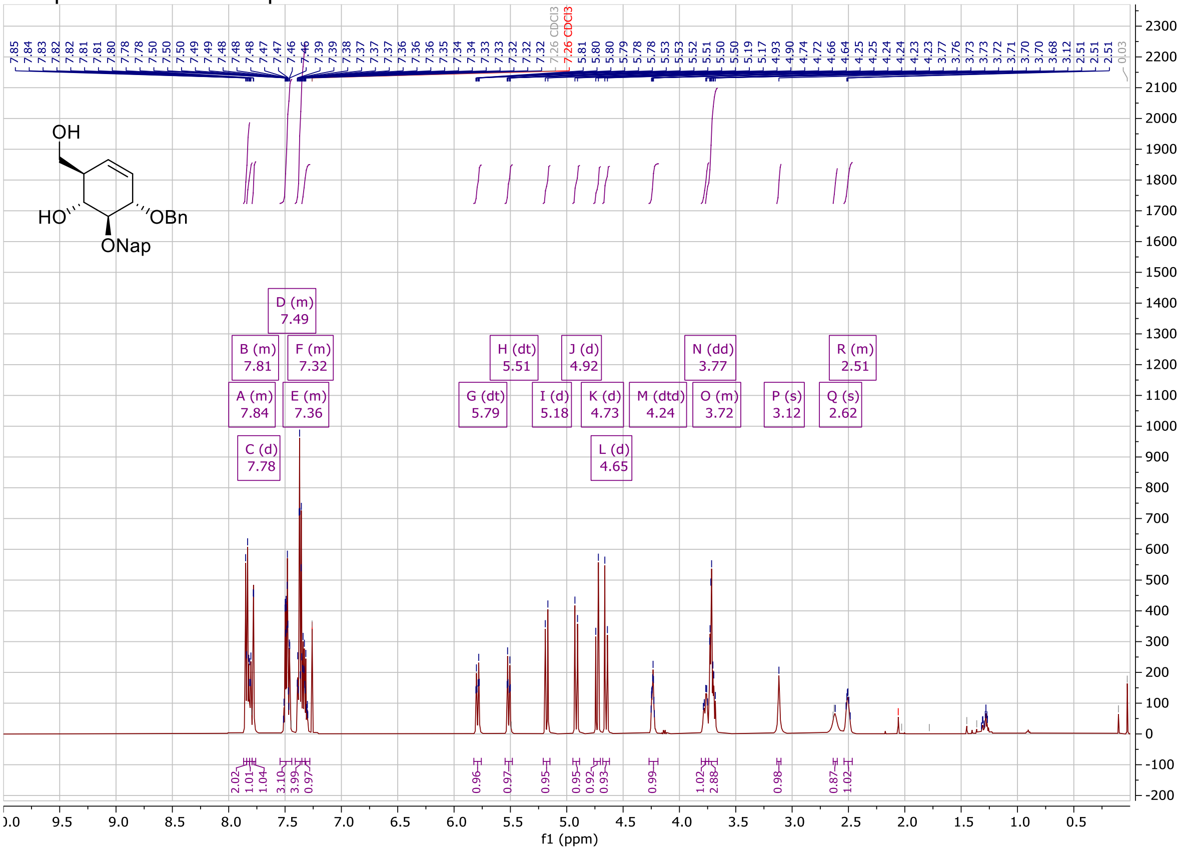

Compound **12** ^{13}C NMR APT spectrum

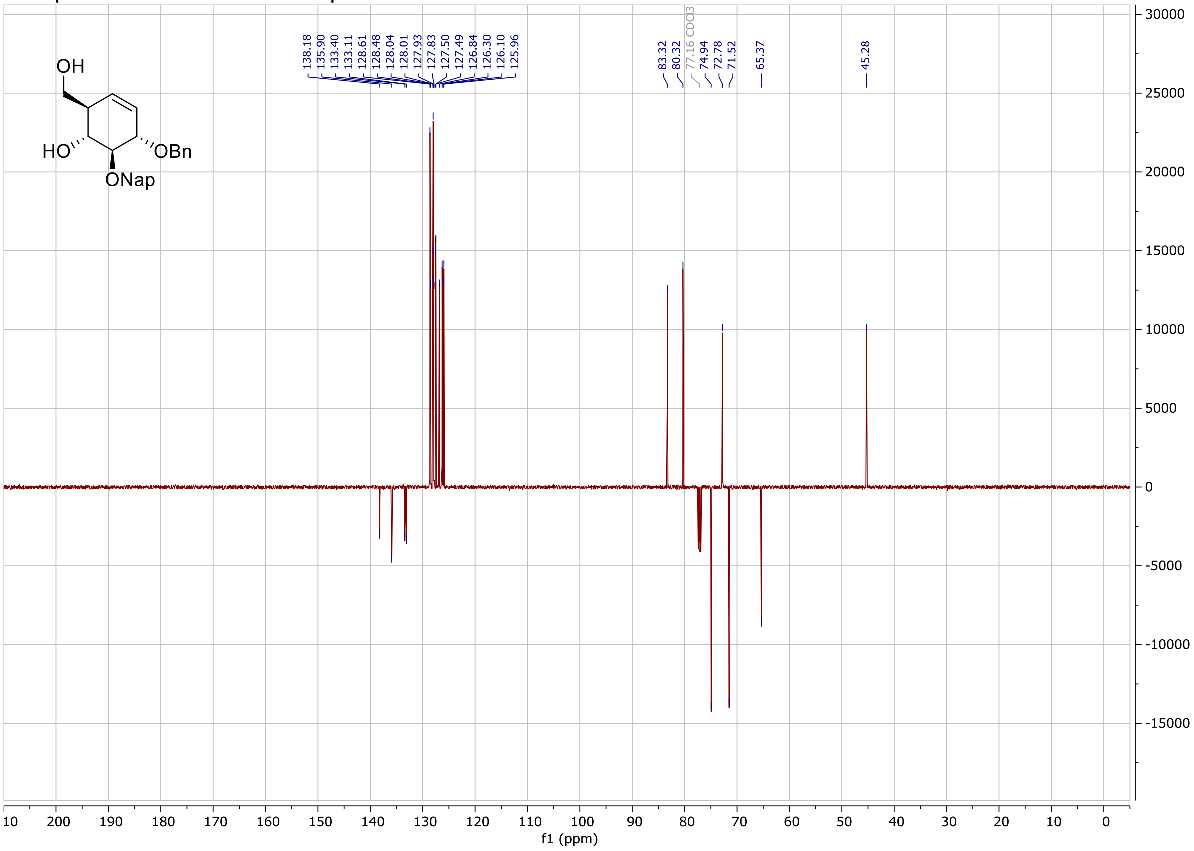

Compound **12** ^{1}H-^{1}H COSY spectrum

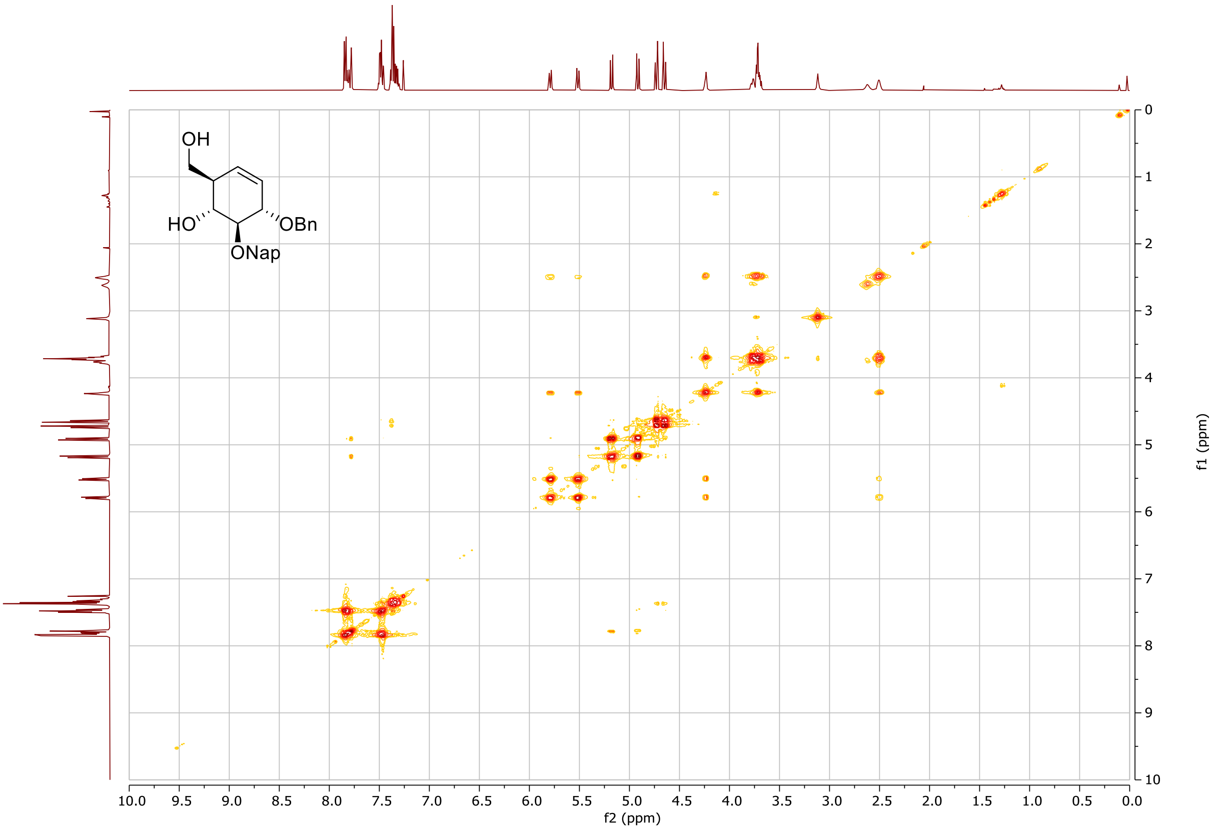

Compound **12** ^{1}H-^{13}C HSQC spectrum

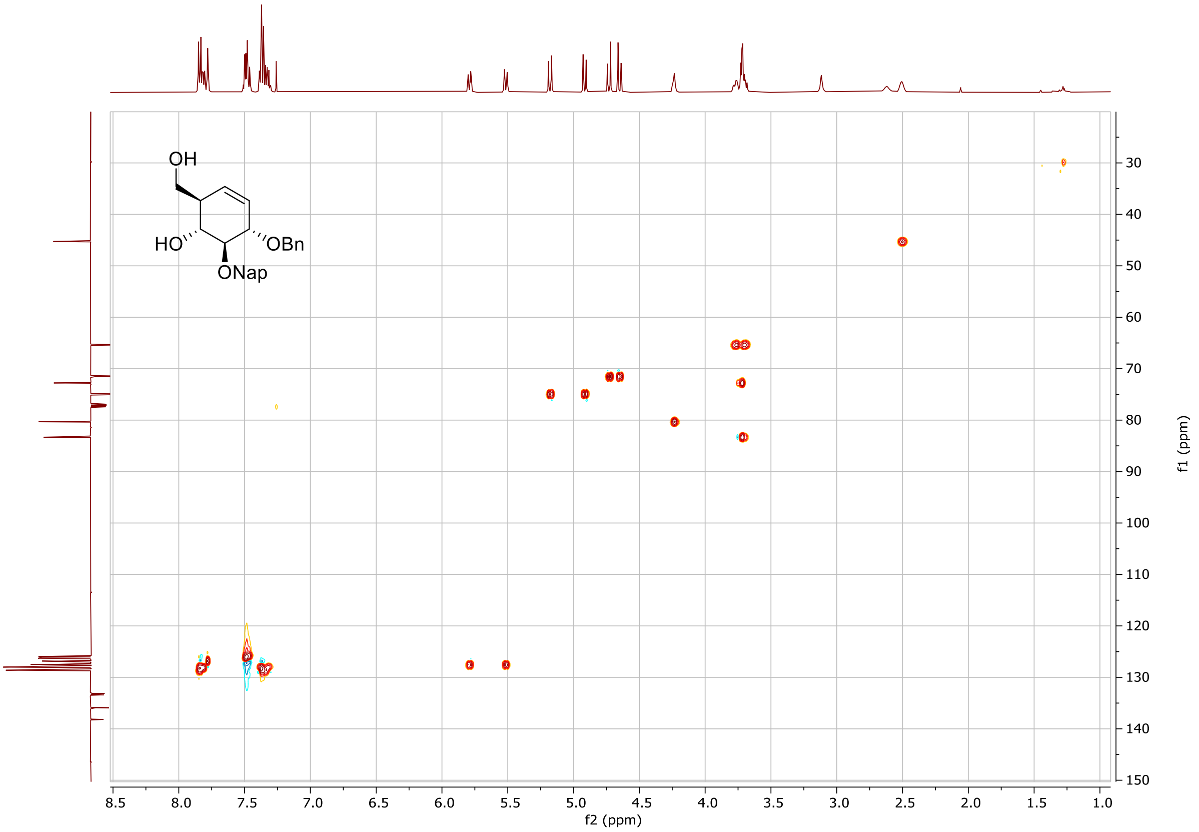

Compound **12** ^{1}H-^{1}H NOESY spectrum

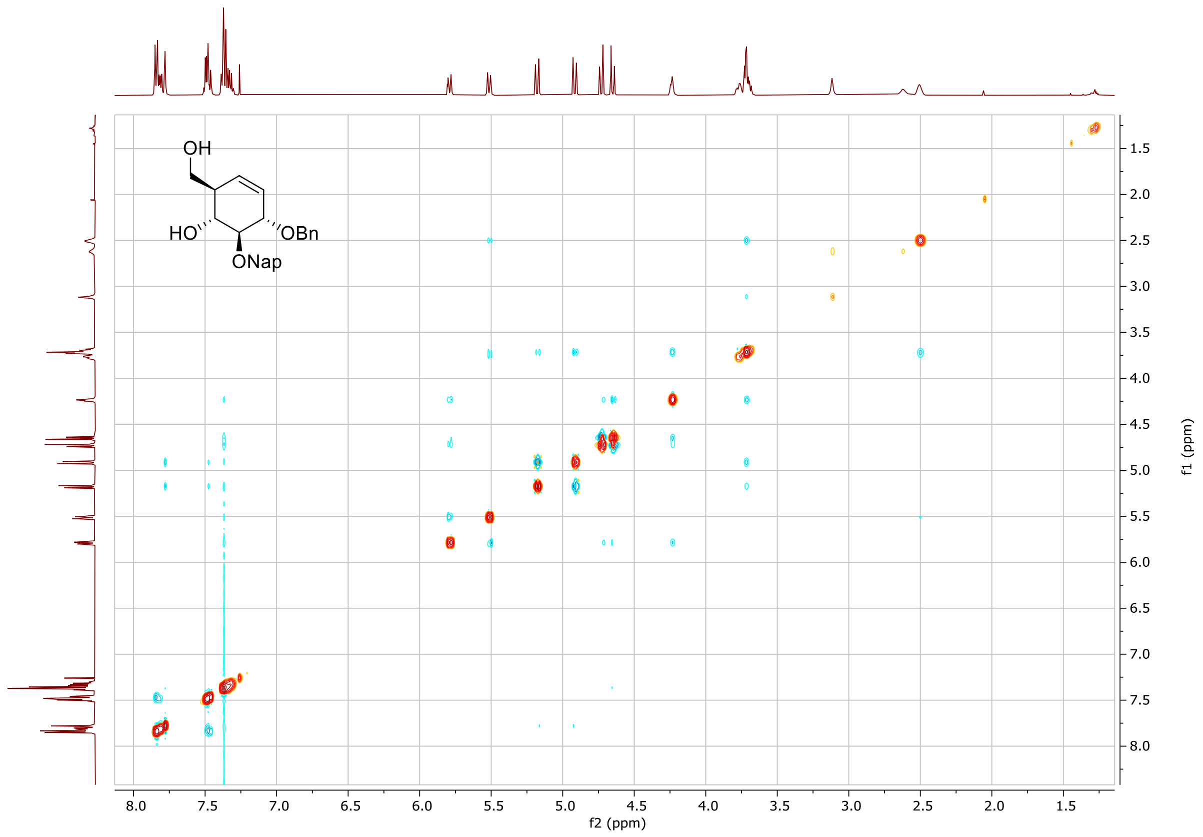

Compound **12** ^{1}H-^{13}C HMBC spectrum

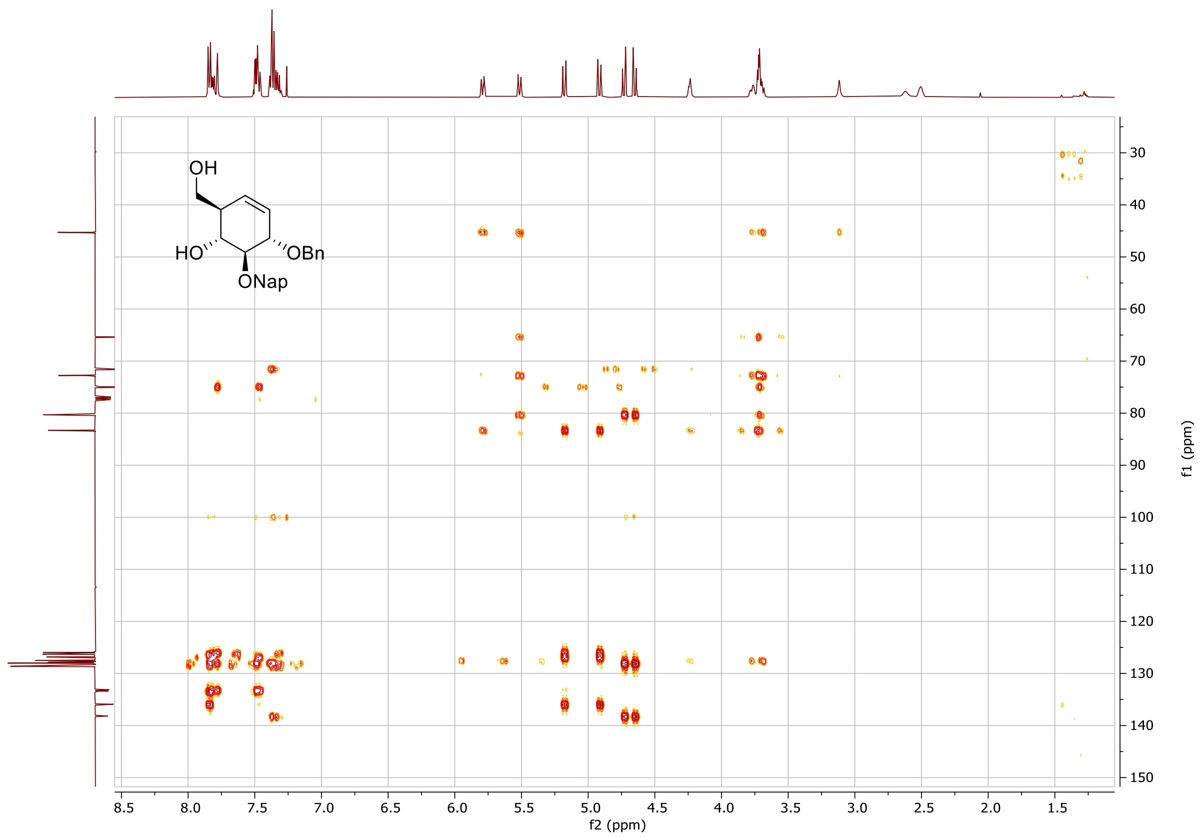

Compound **14** ^{1}H NMR spectrum

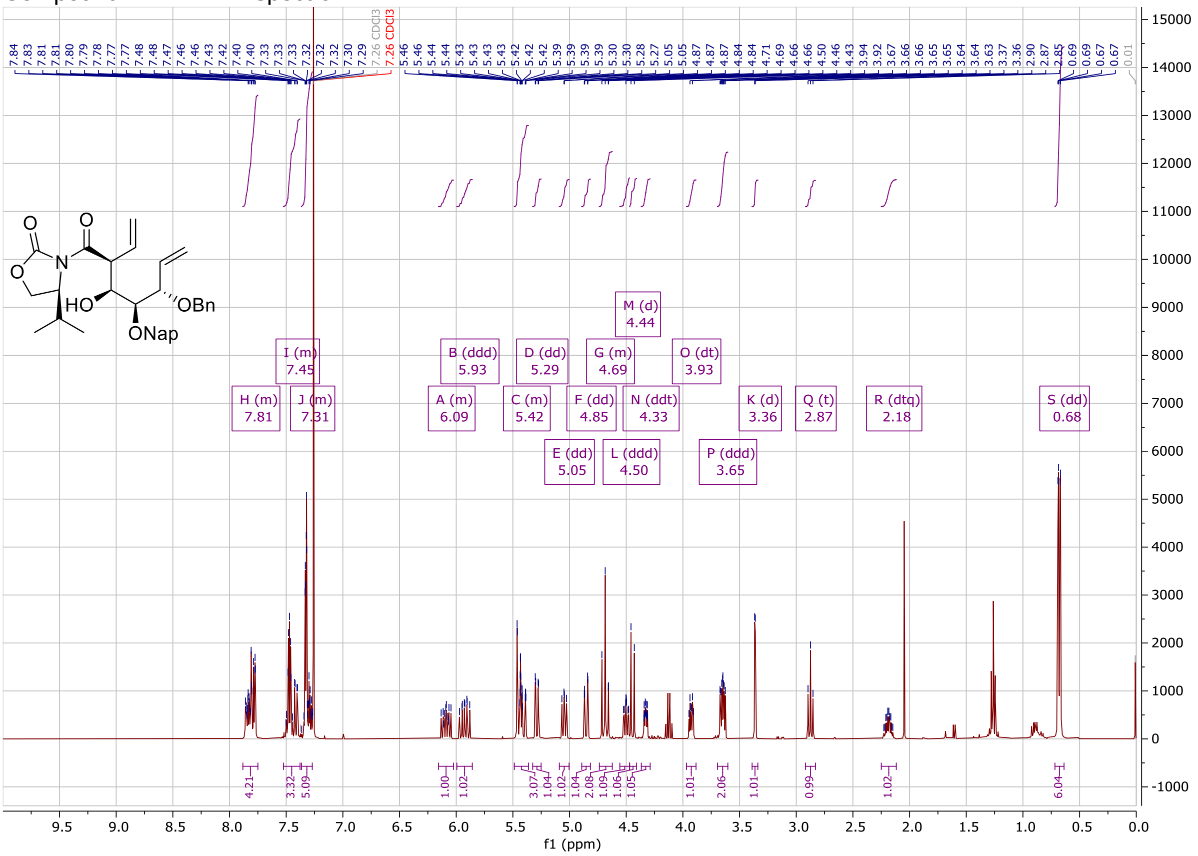

Compound **14** ^{13}C NMR APT spectrum

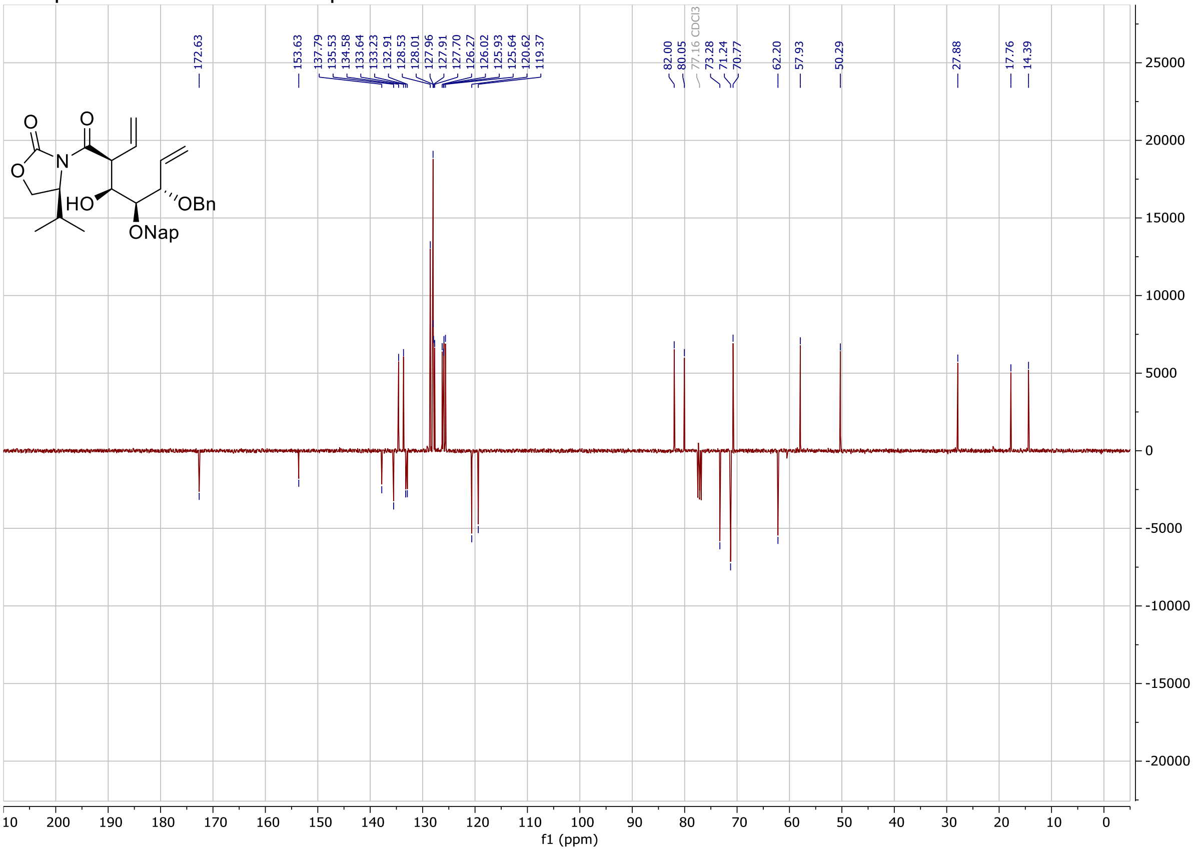

Compound **15** ^{1}H NMR spectrum

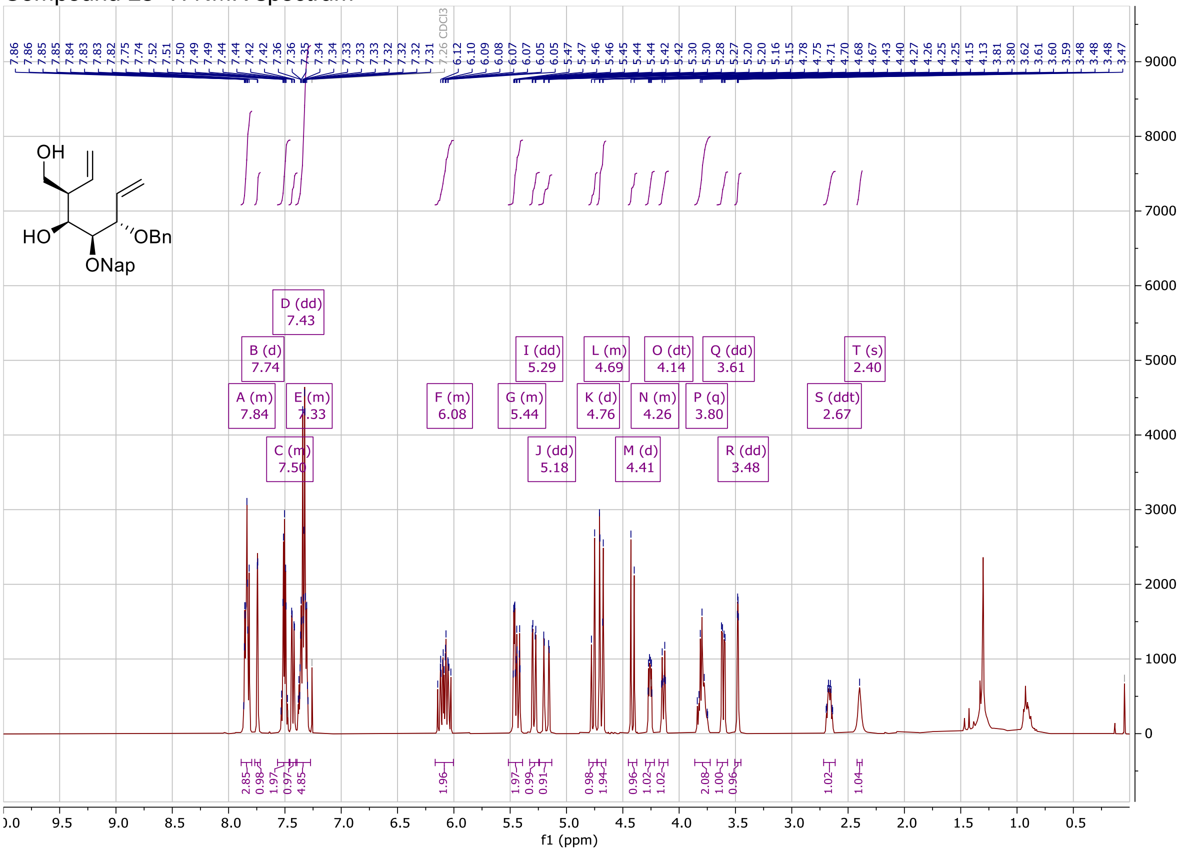

Compound **15** ^{13}C NMR APT spectrum

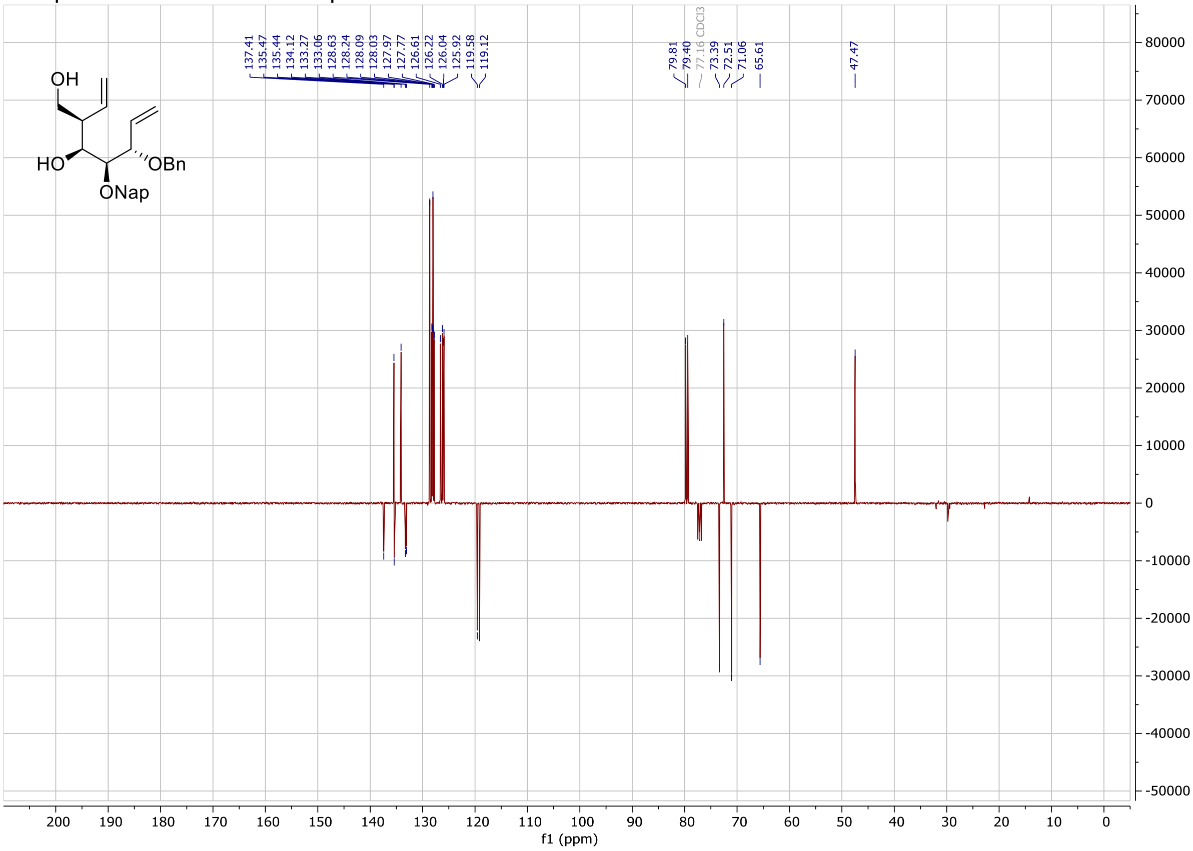

Compound **16** ^{1}H NMR spectrum

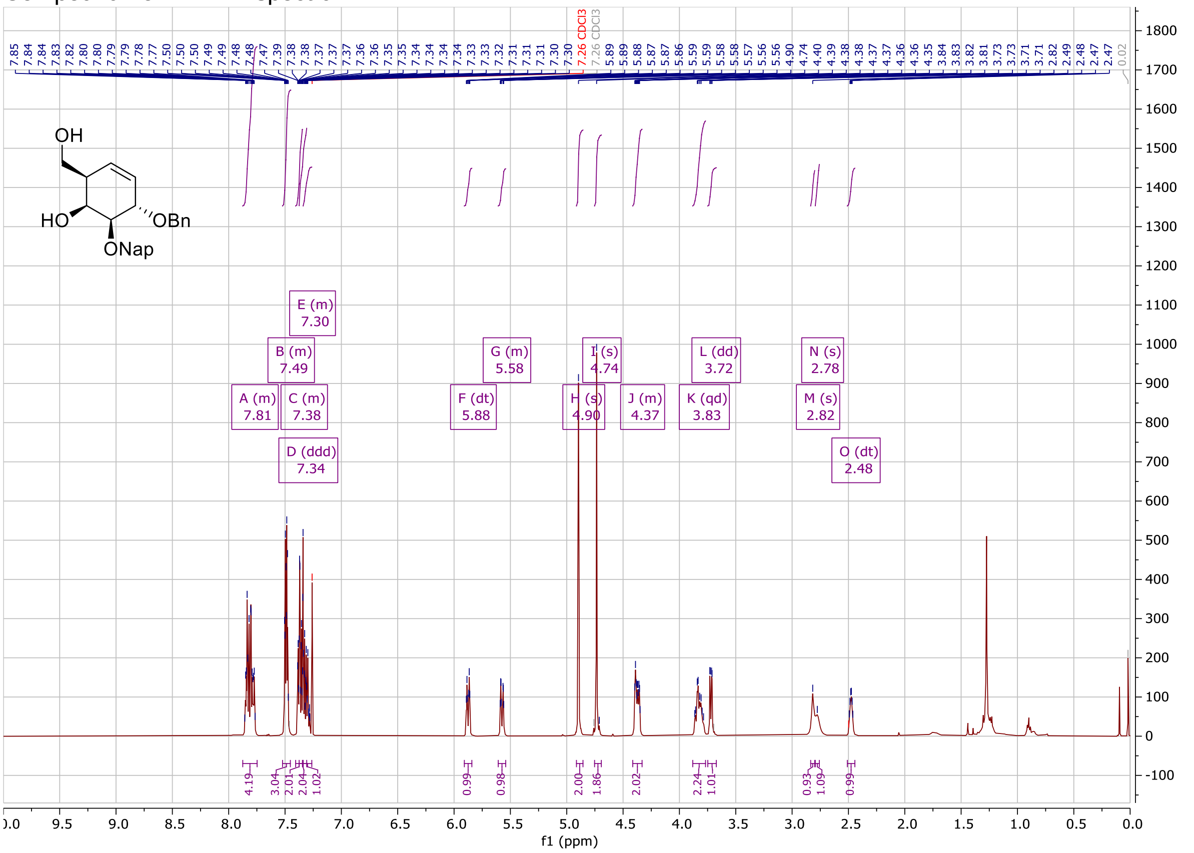

Compound **16** ^{13}C NMR APT spectrum

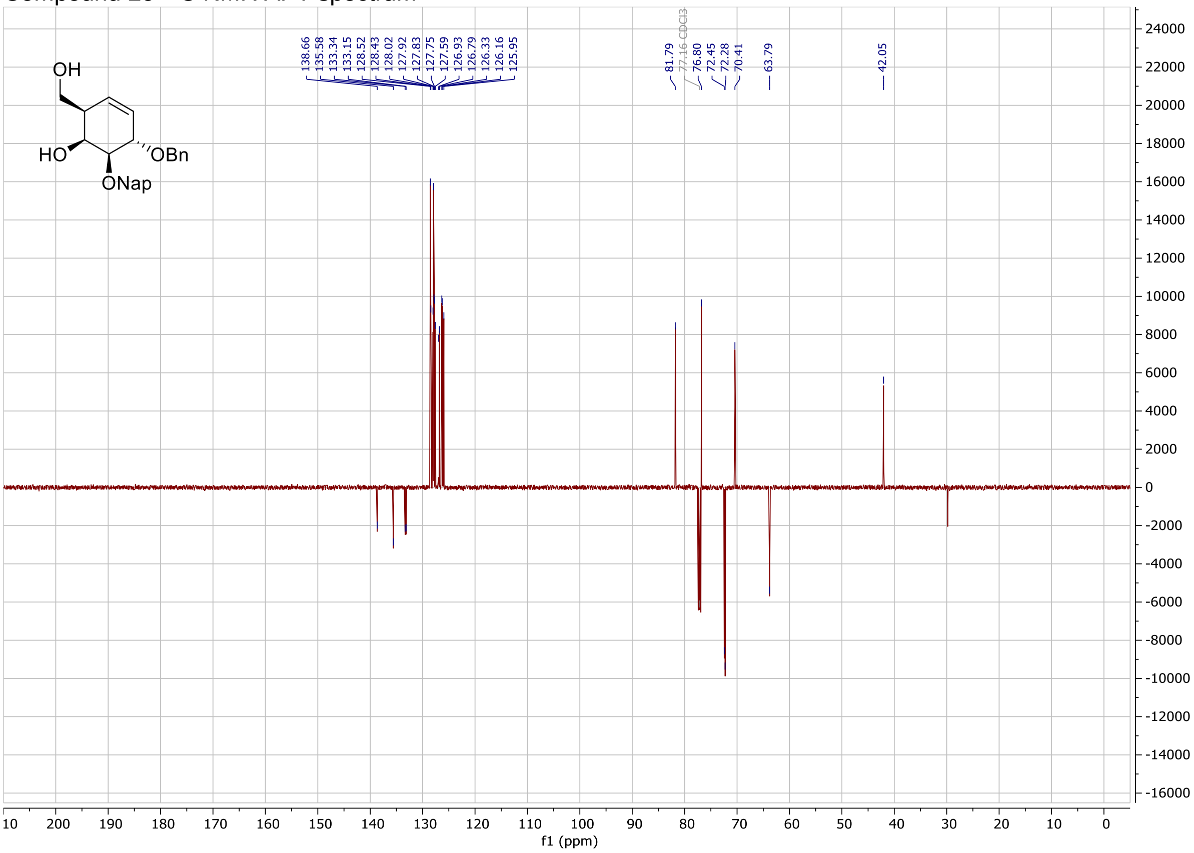

Compound **16** ^{1}H-^{1}H COSY spectrum

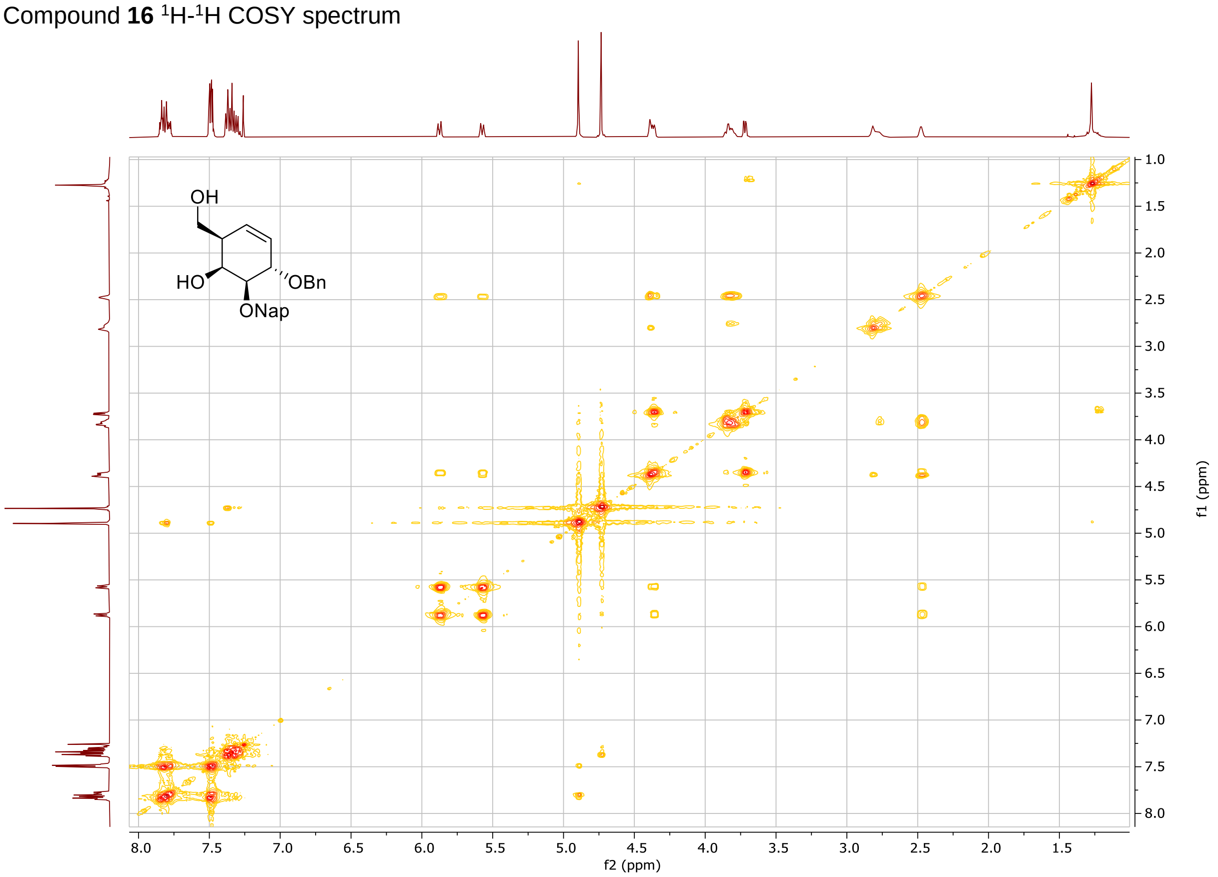

Compound **16** ^{1}H-^{13}C HSQC spectrum

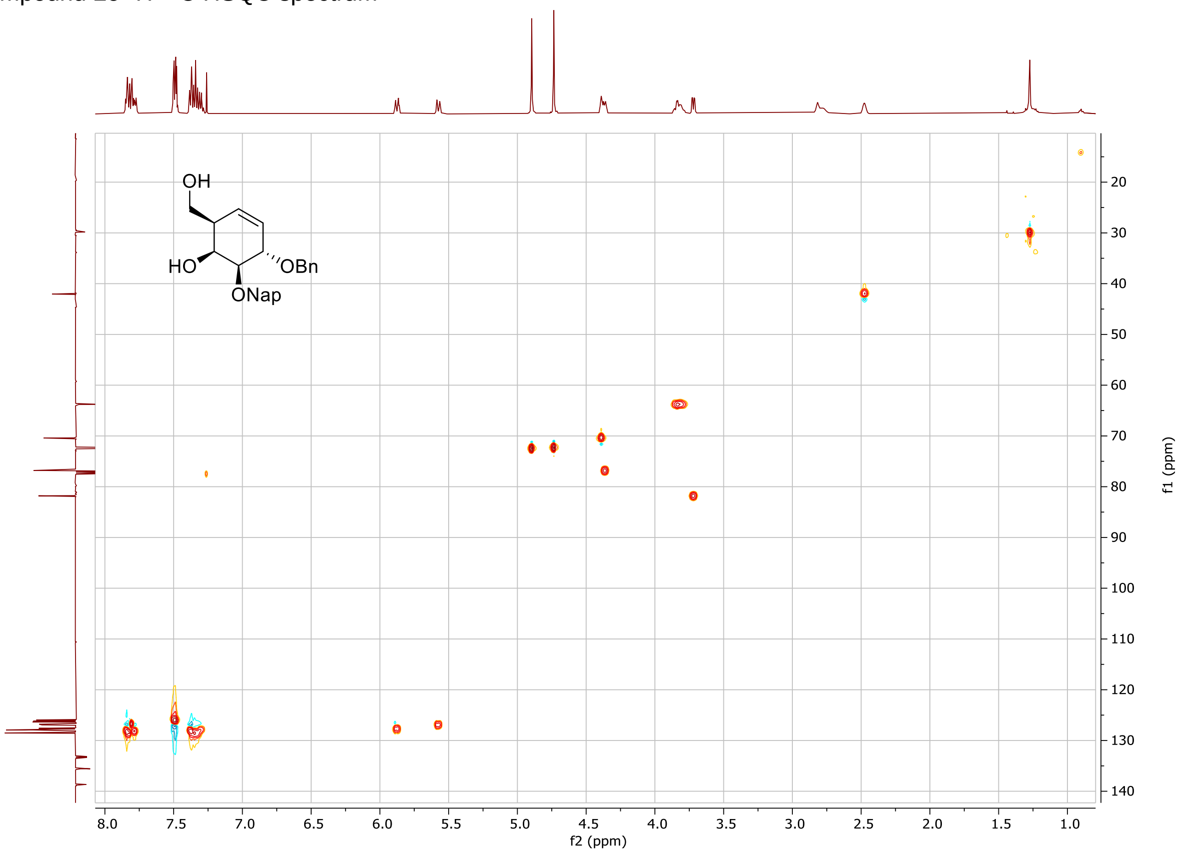

Compound **16** ^{1}H-^{1}H NOESY spectrum

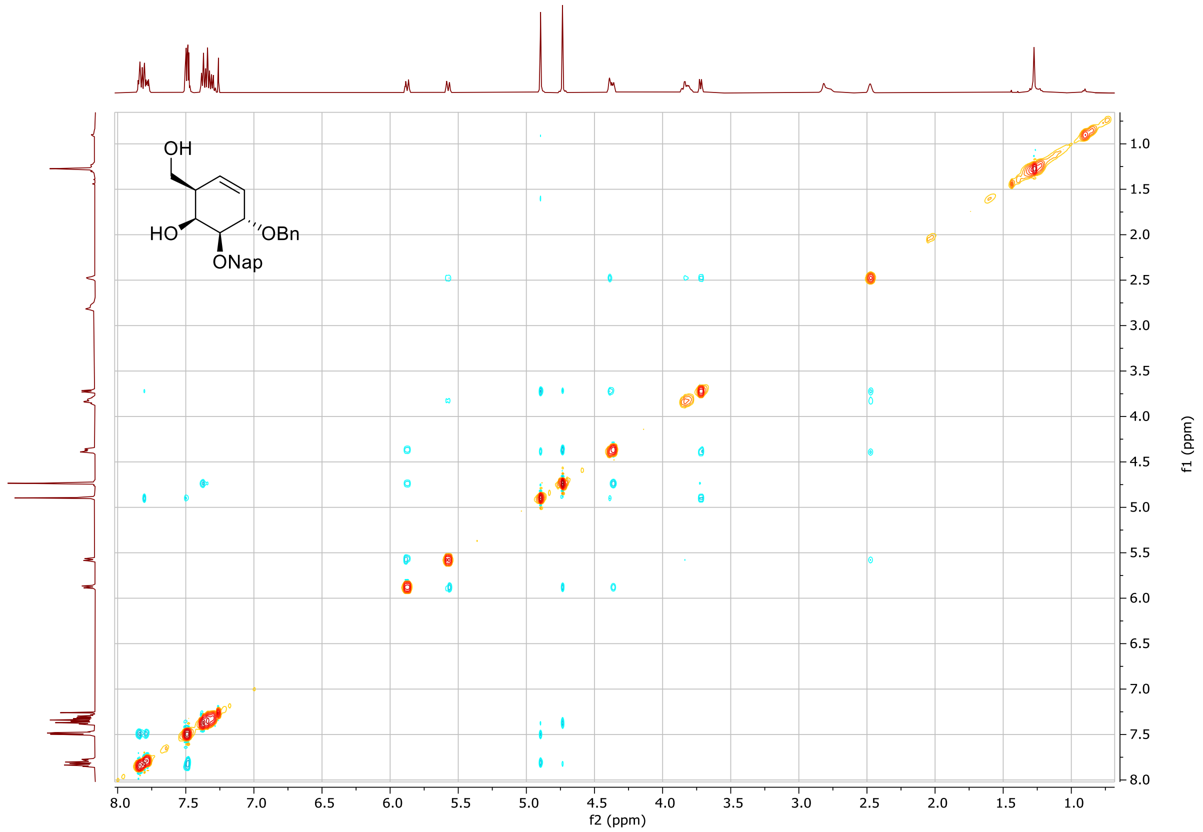

Compound **17** ^{1}H NMR spectrum

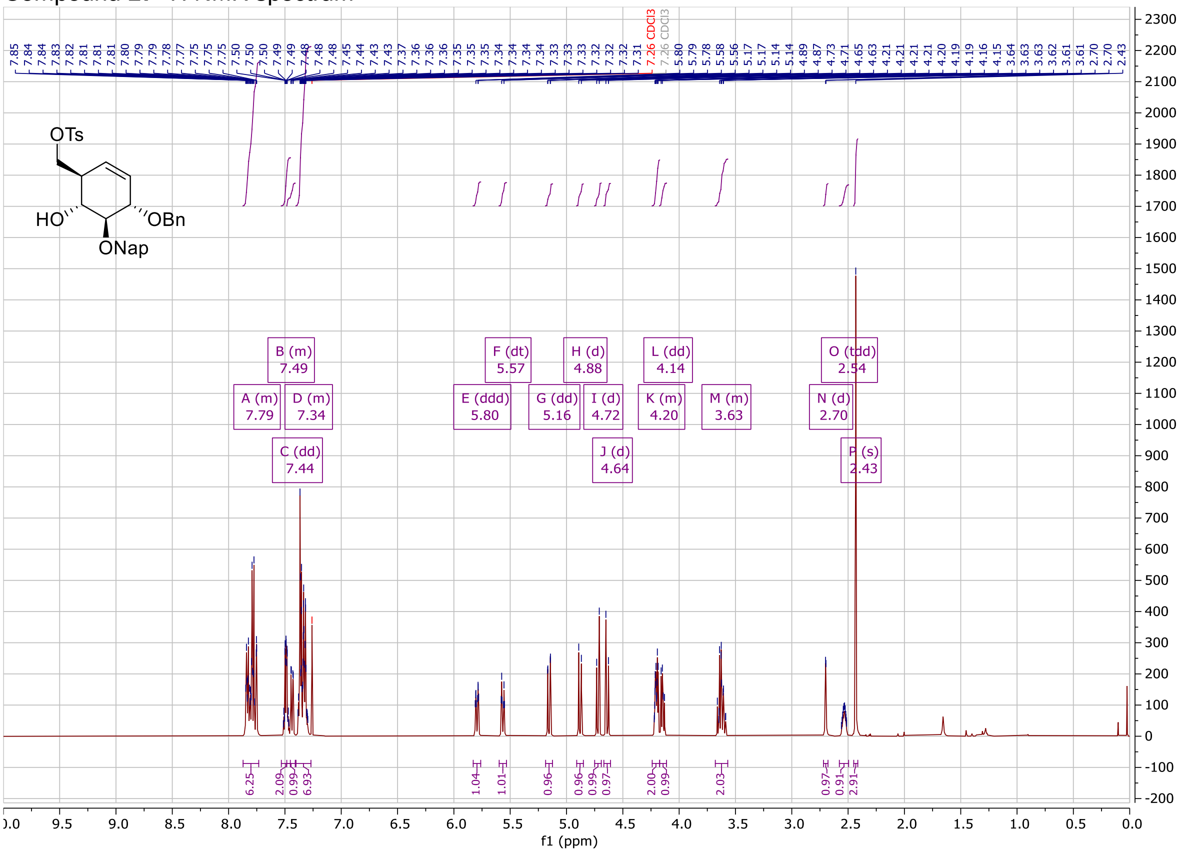

Compound **17** ^{13}C NMR APT spectrum

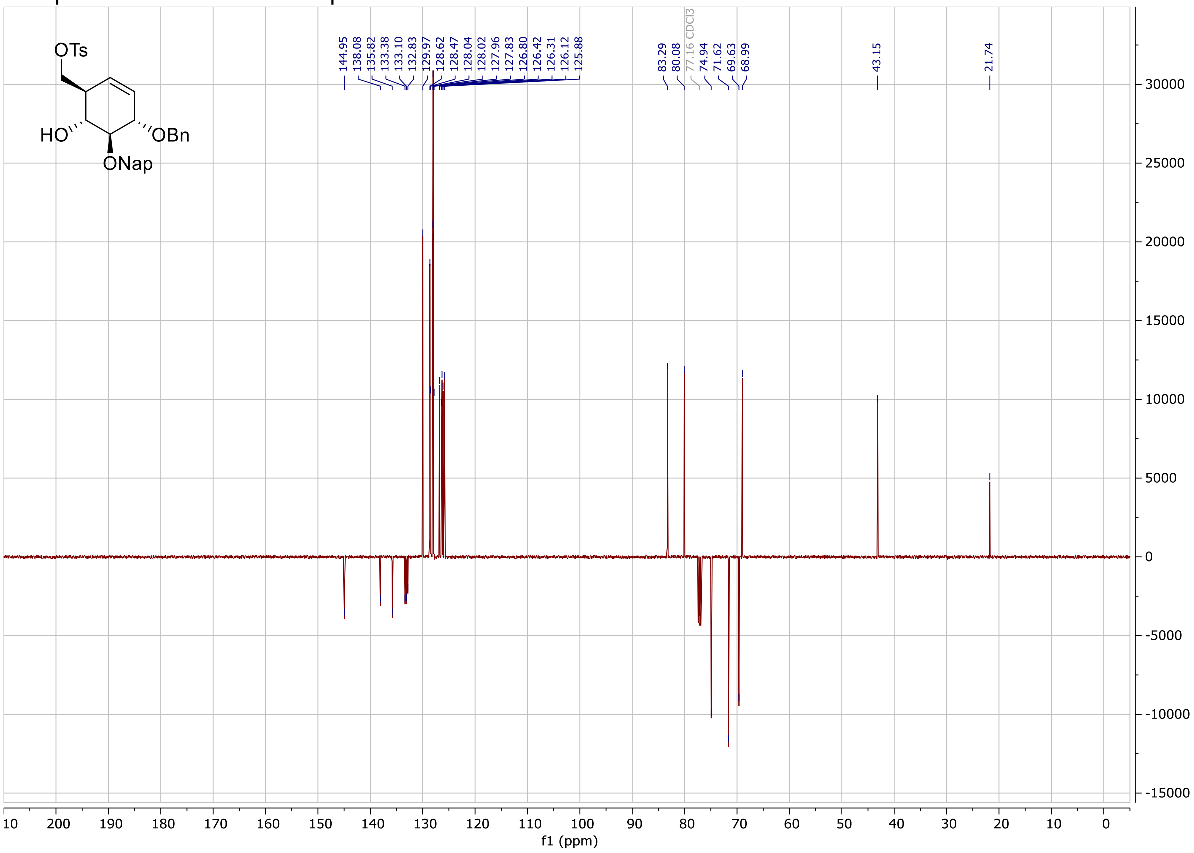

Compound **17** ^{1}H-^{1}H COSY spectrum

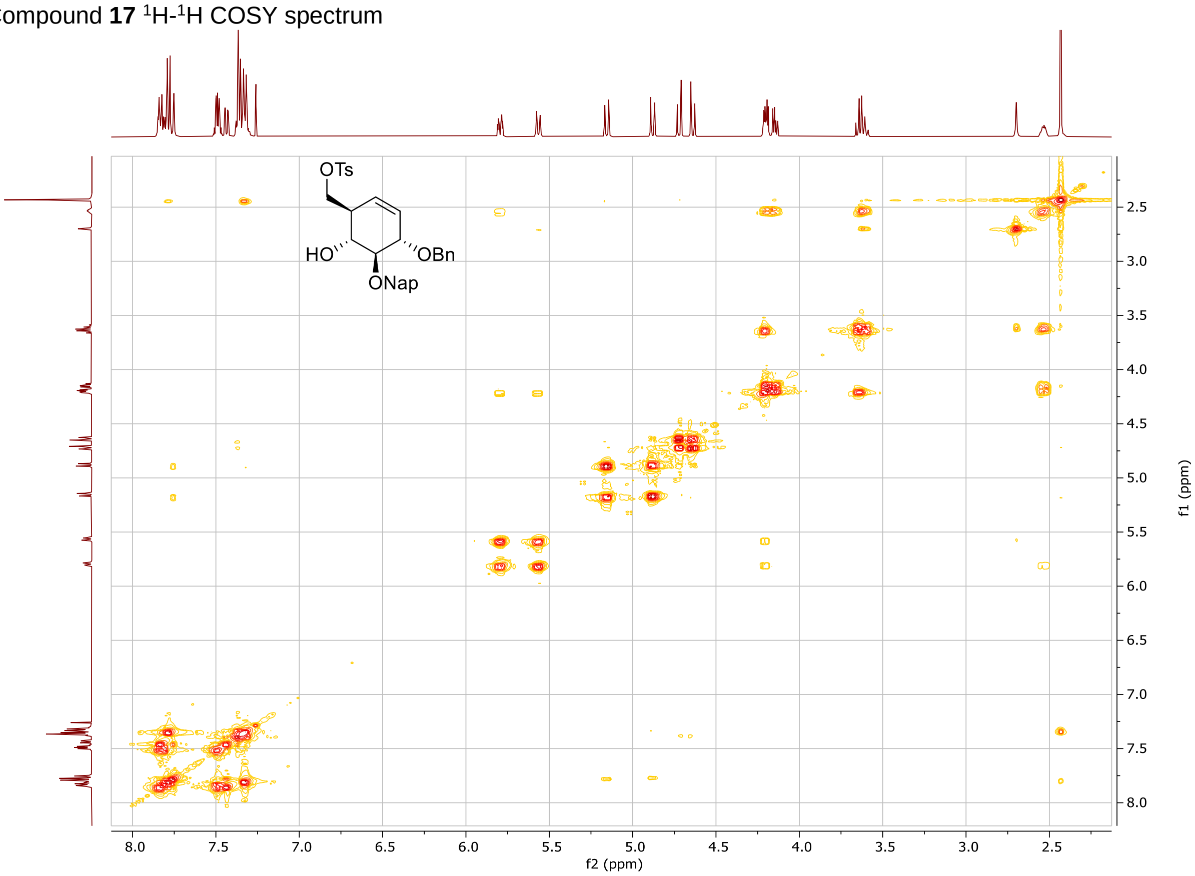

Compound **17** ^{1}H-^{13}C HSQC spectrum

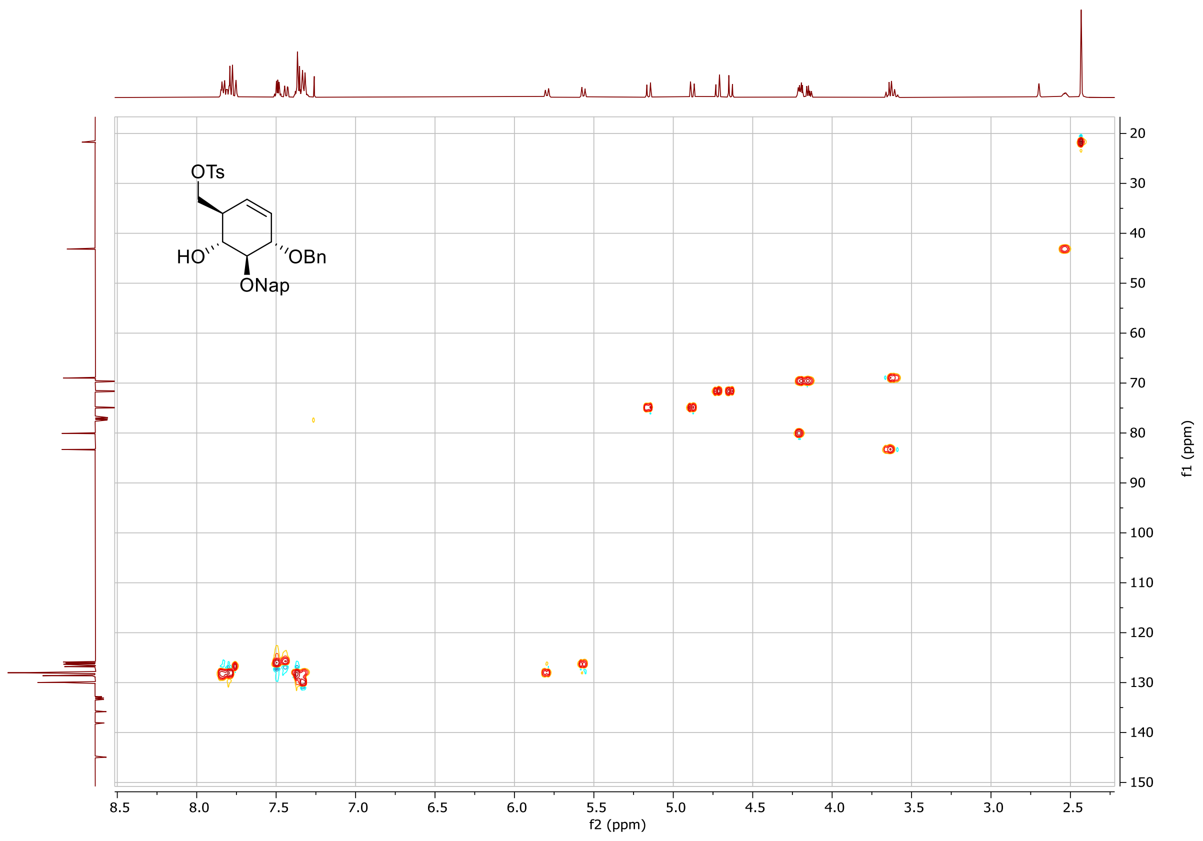

Compound **17** ^{1}H-^{1}H NOESY spectrum

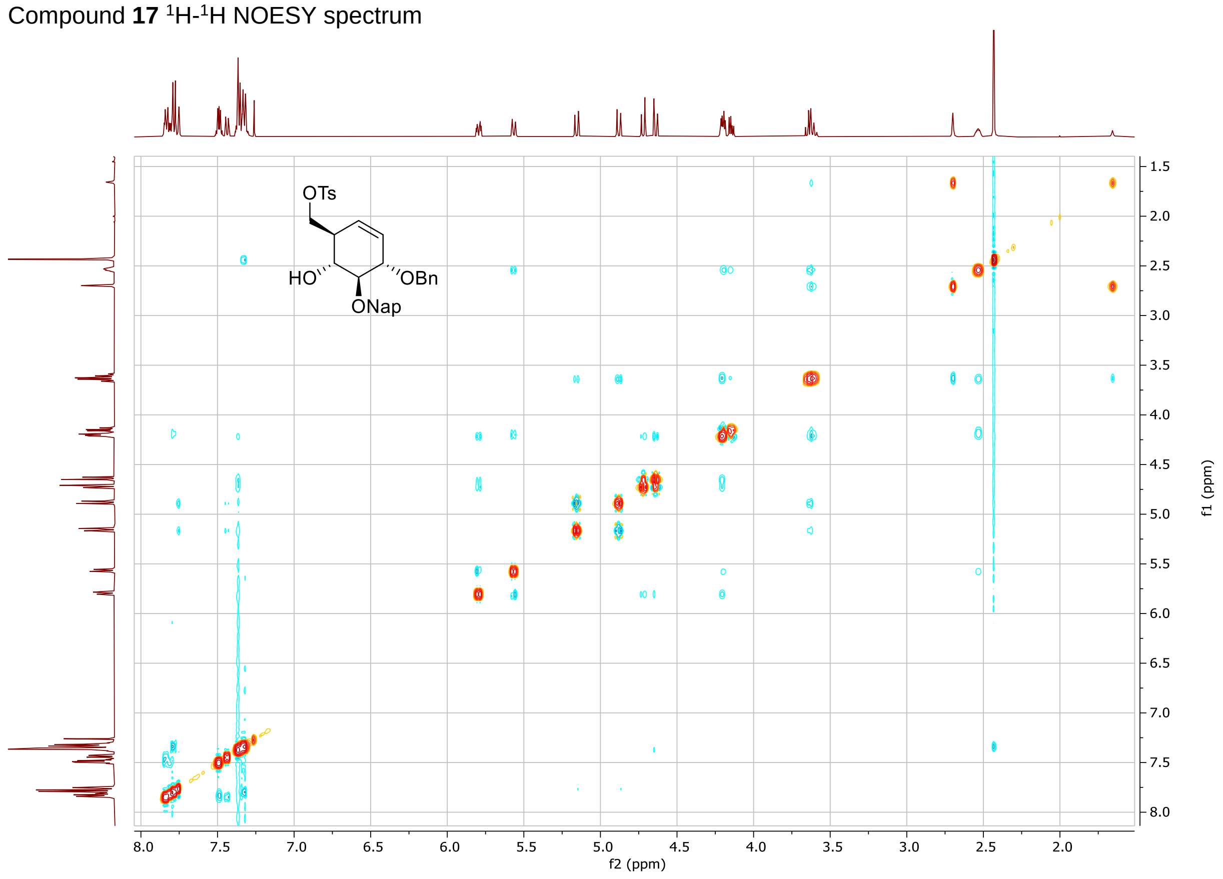

Compound **17** ^{1}H-^{13}C HMBC spectrum

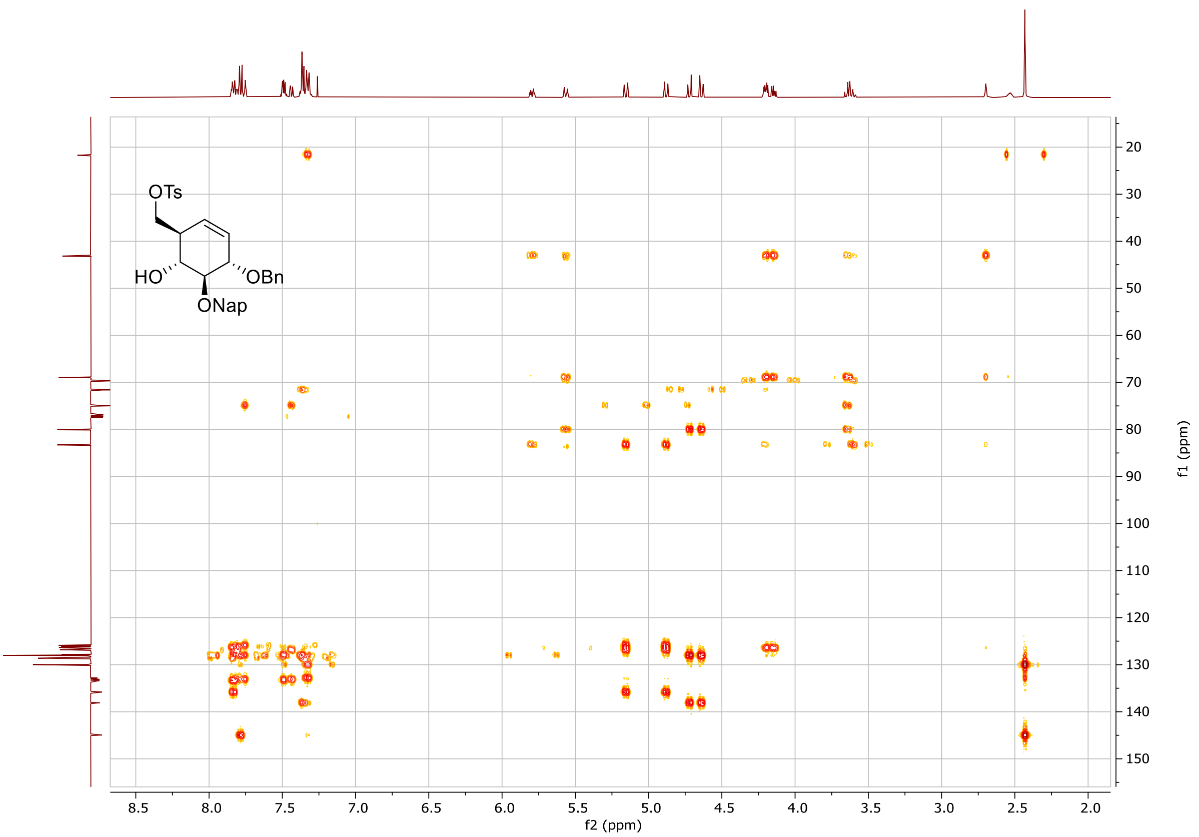

Compound **18** ^{1}H NMR spectrum

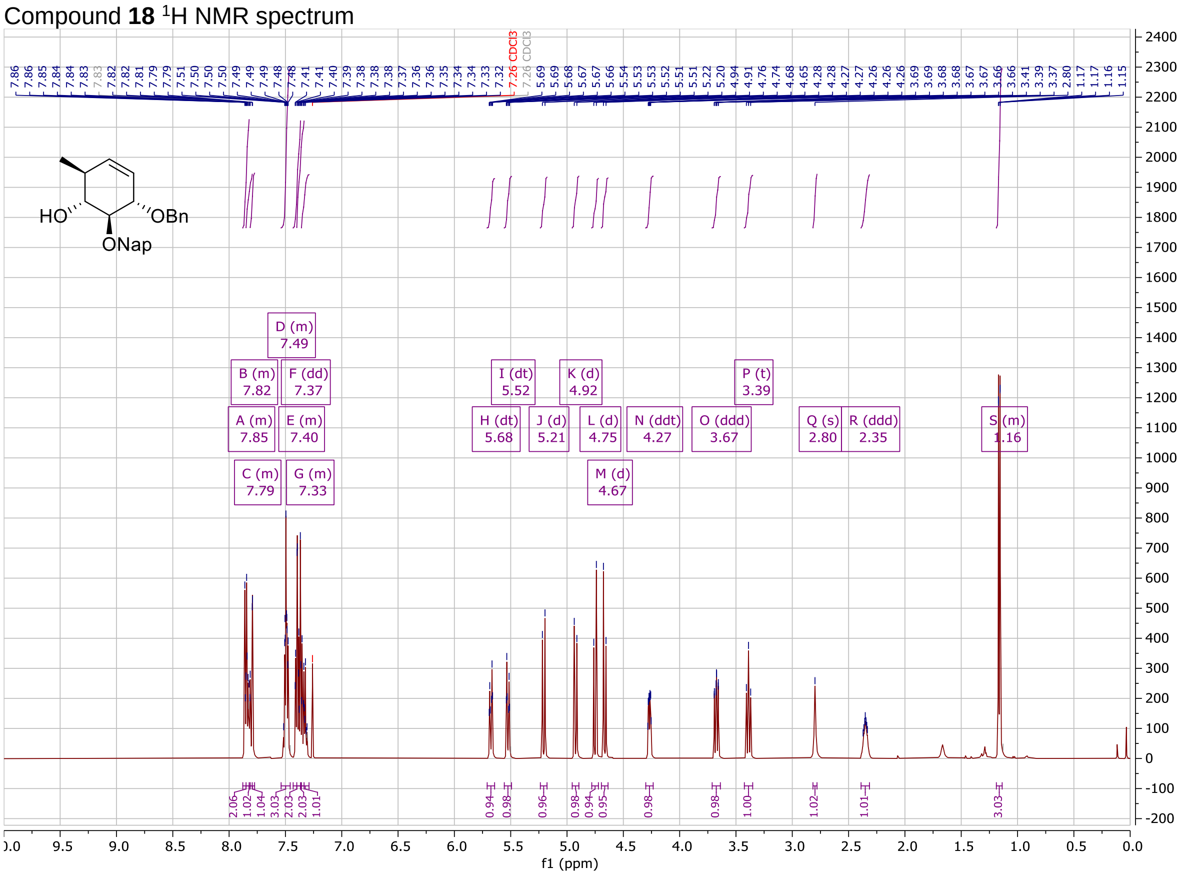

Compound **18** ^{13}C NMR APT spectrum

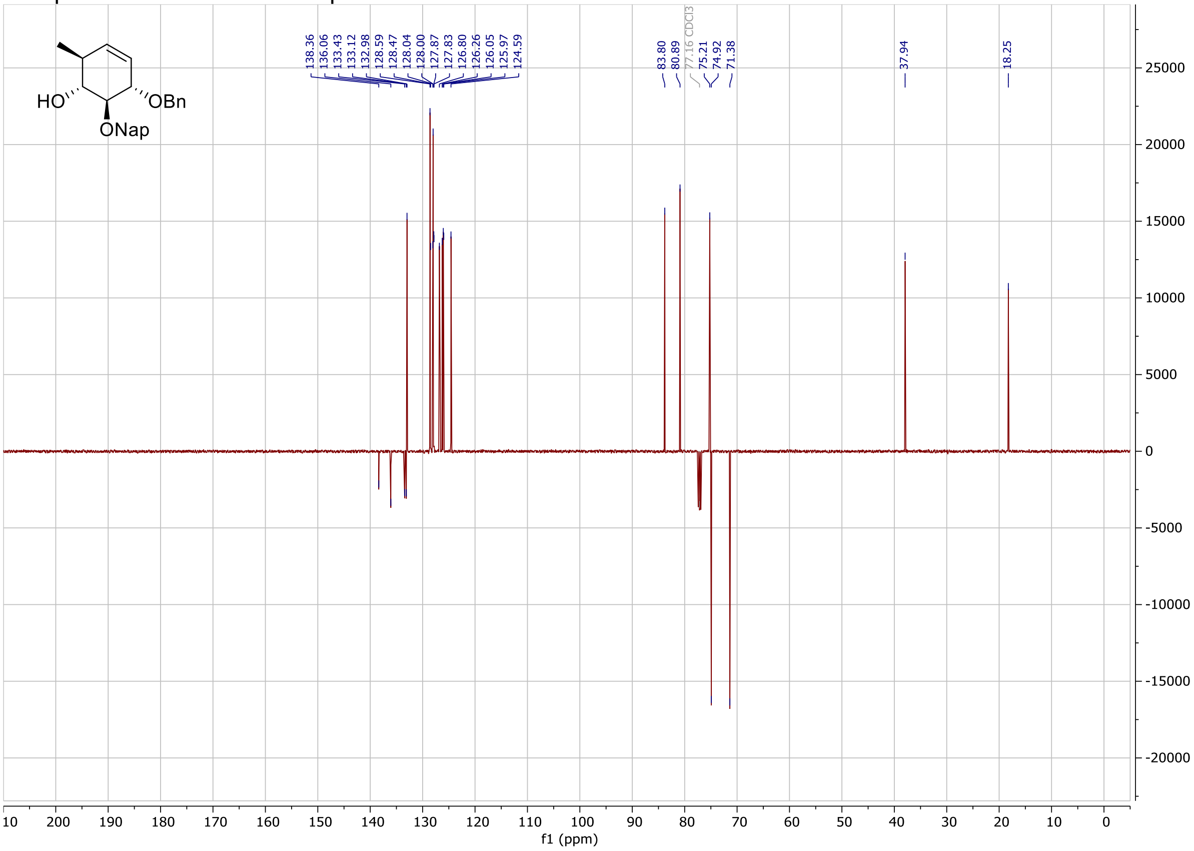

Compound **18** ^{1}H-^{1}H COSY spectrum

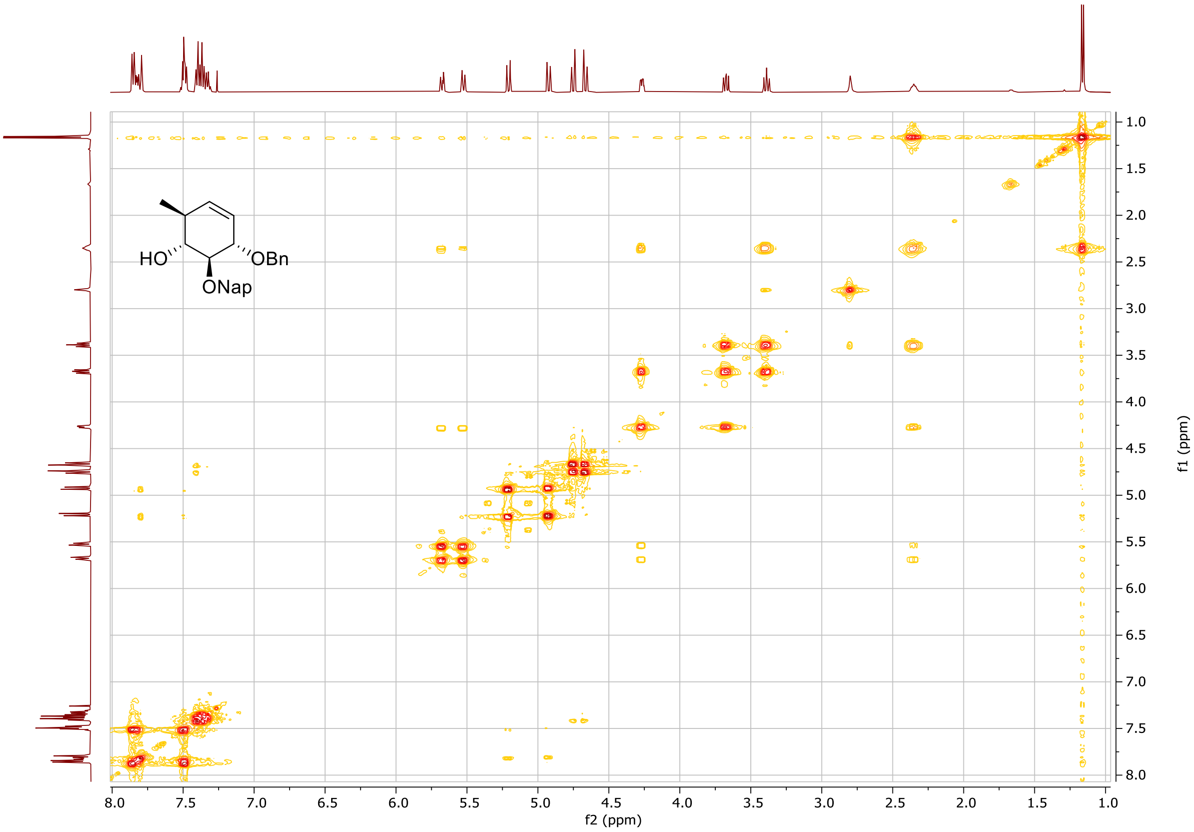

Compound **18** ^{1}H-^{13}C HSQC spectrum

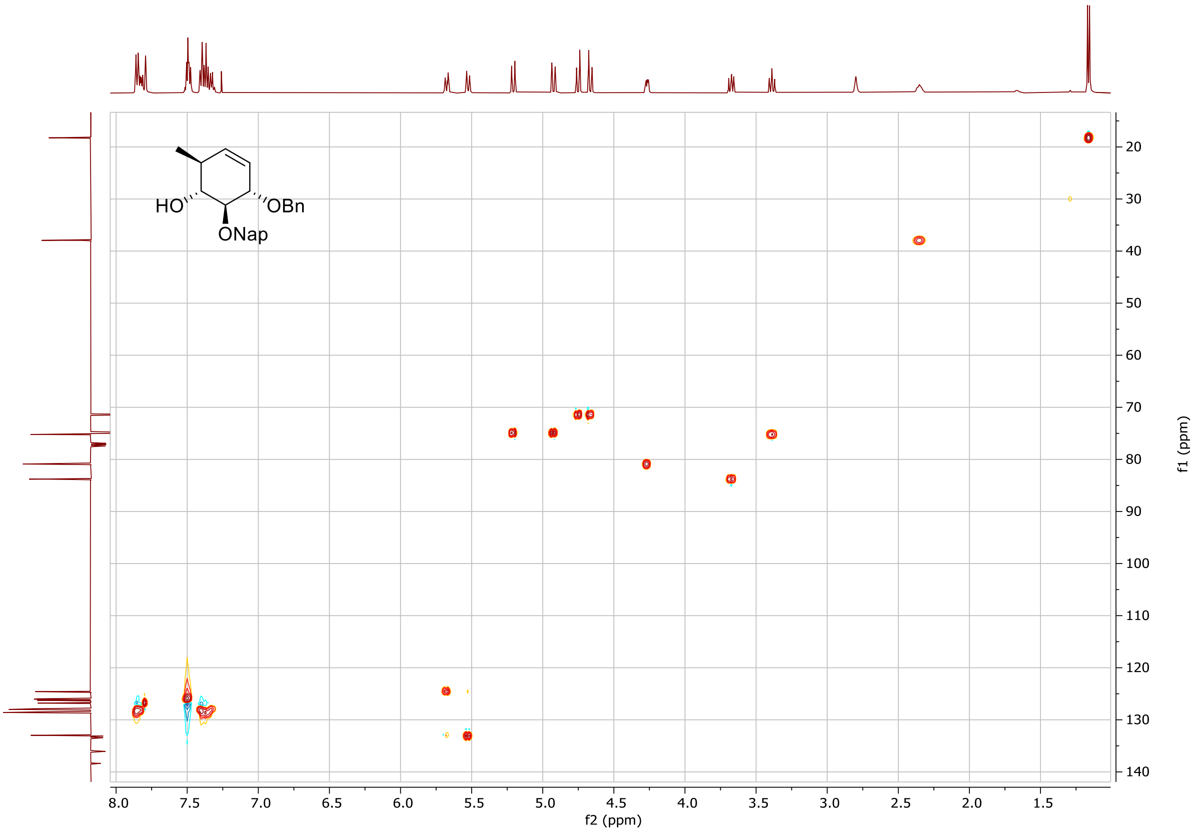

Compound **18** ^{1}H-^{1}H NOESY spectrum

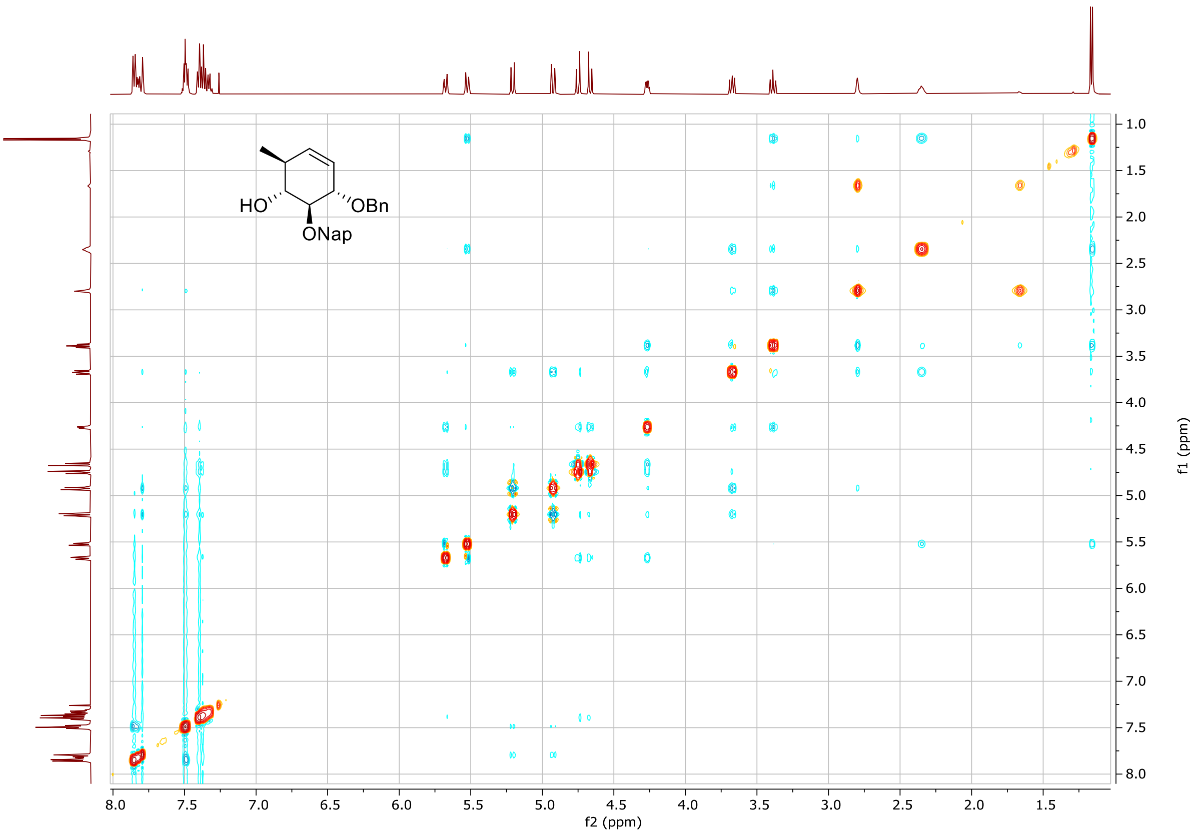

Compound **19** ^{1}H NMR spectrum

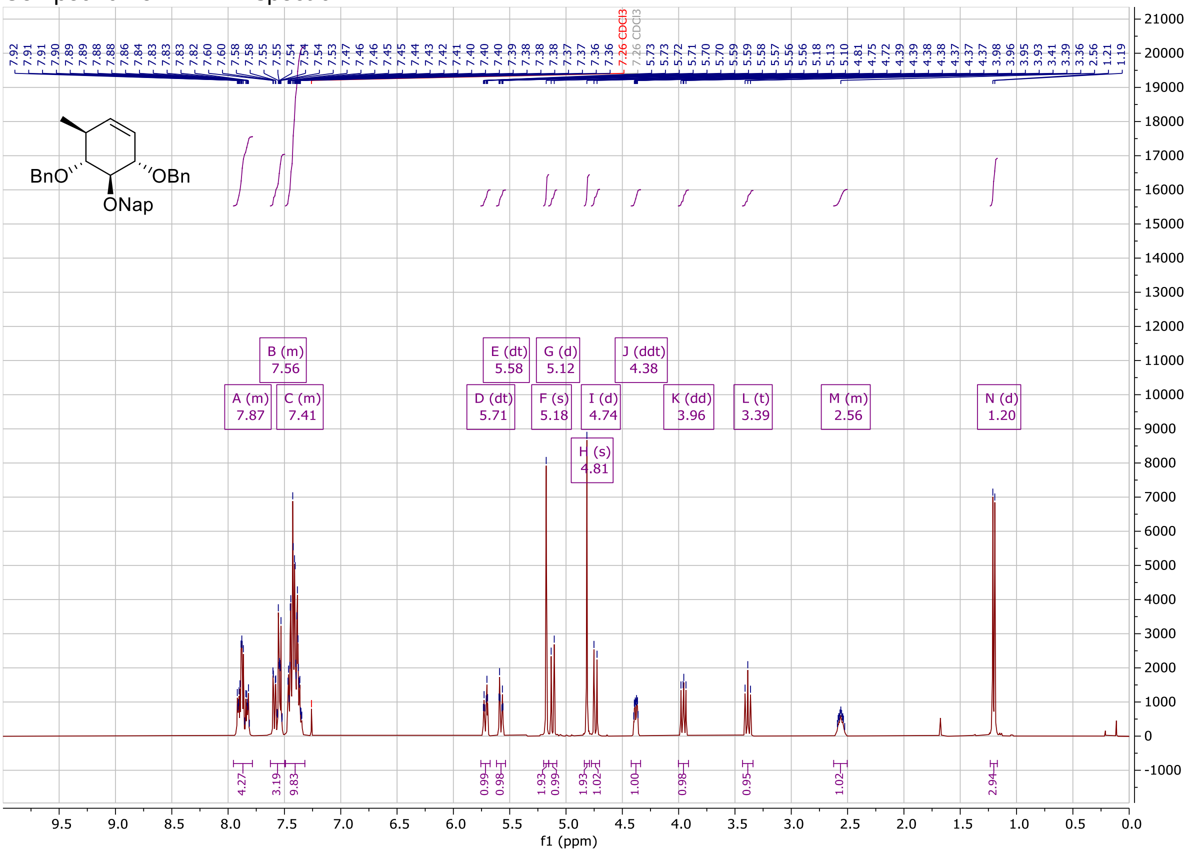

Compound **19** ^{13}C NMR APT spectrum

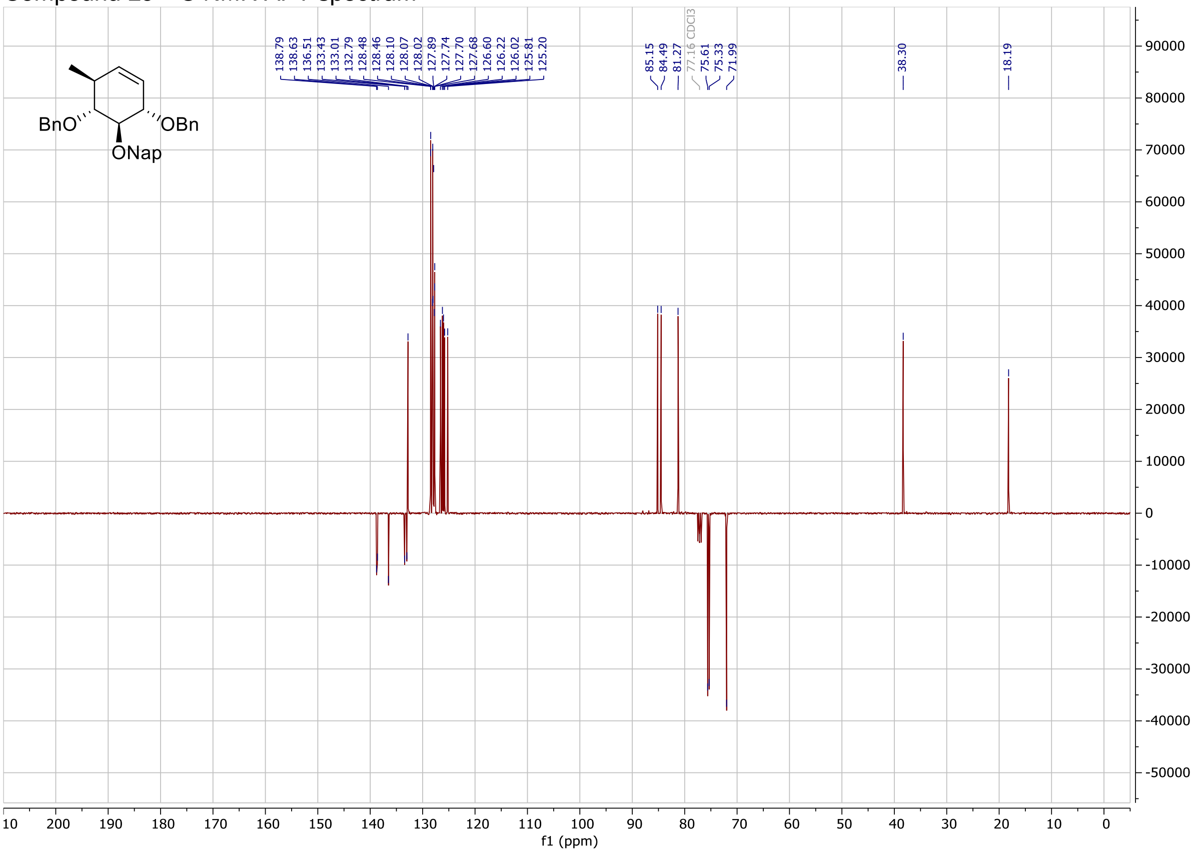

Compound **19** ^{1}H-^{1}H COSY spectrum

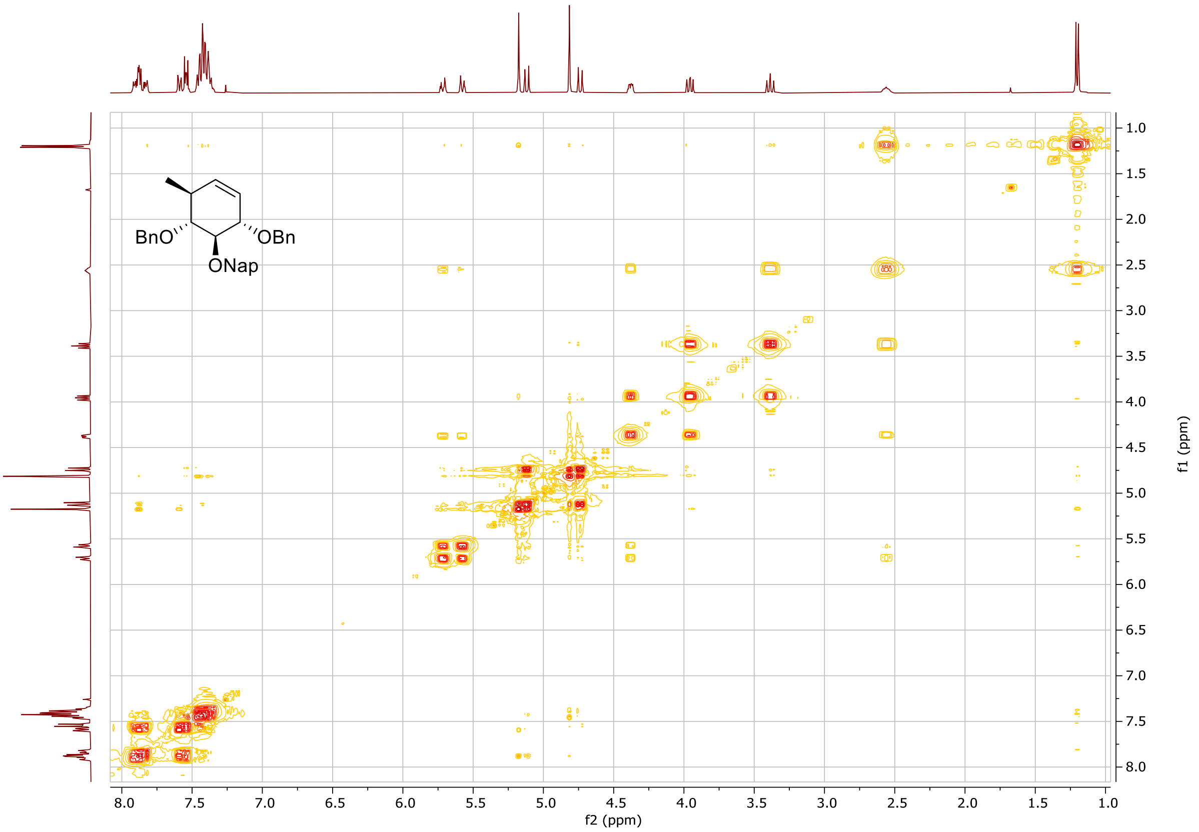

Compound **19** ^{1}H-^{13}C HSQC spectrum

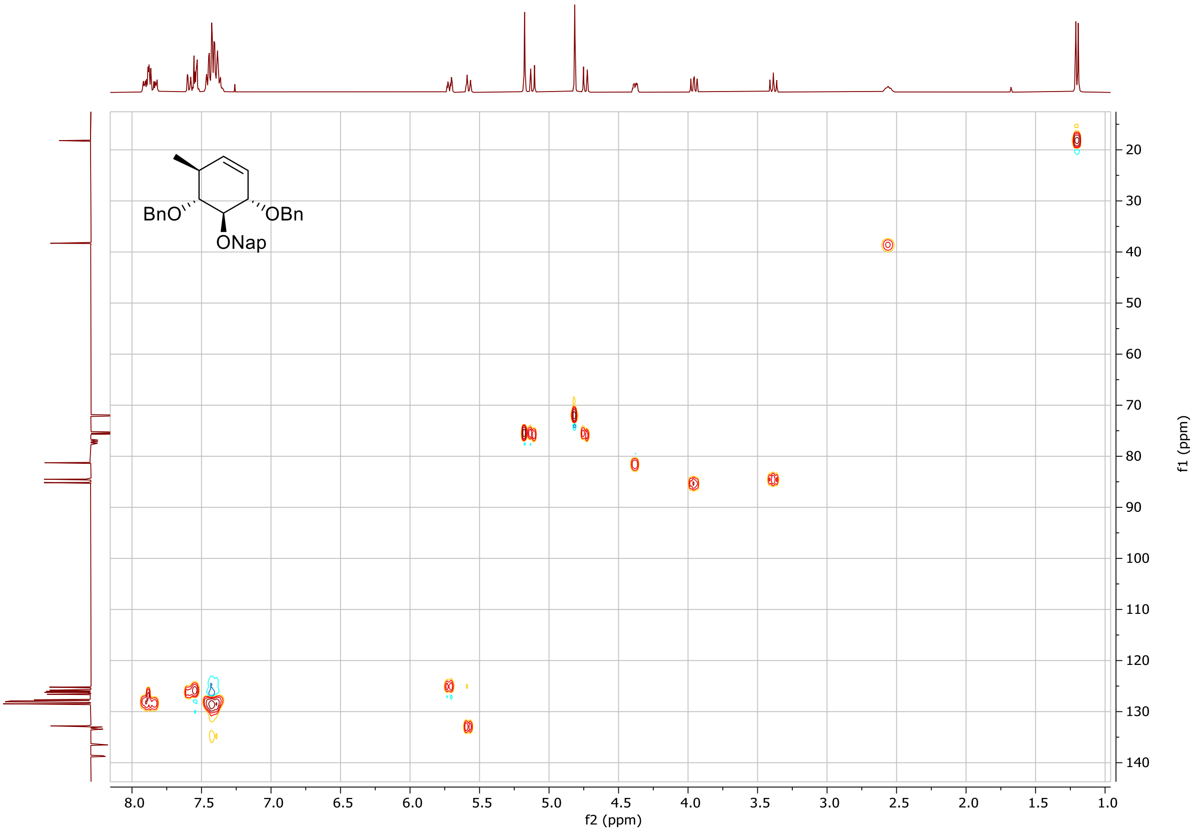

Compound **20** ^{1}H NMR spectrum

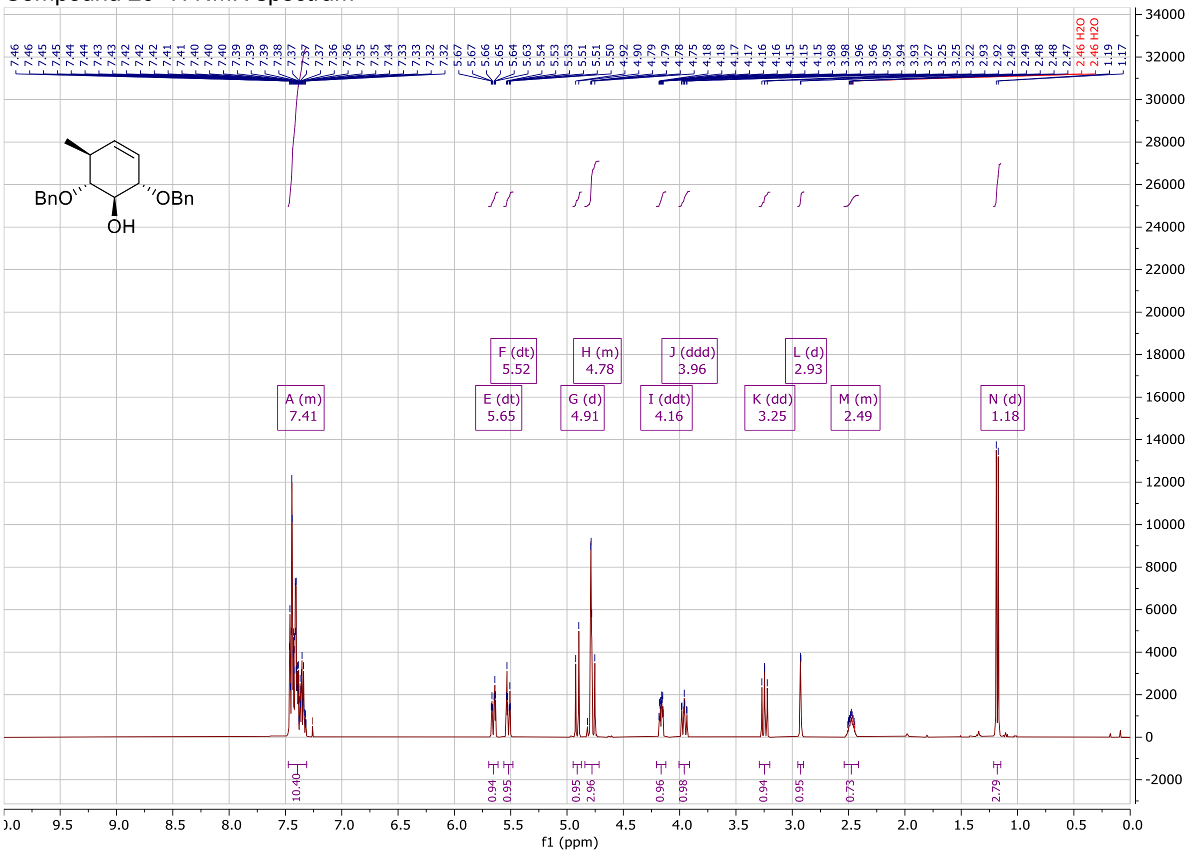

Compound **20** ^{13}C NMR APT spectrum

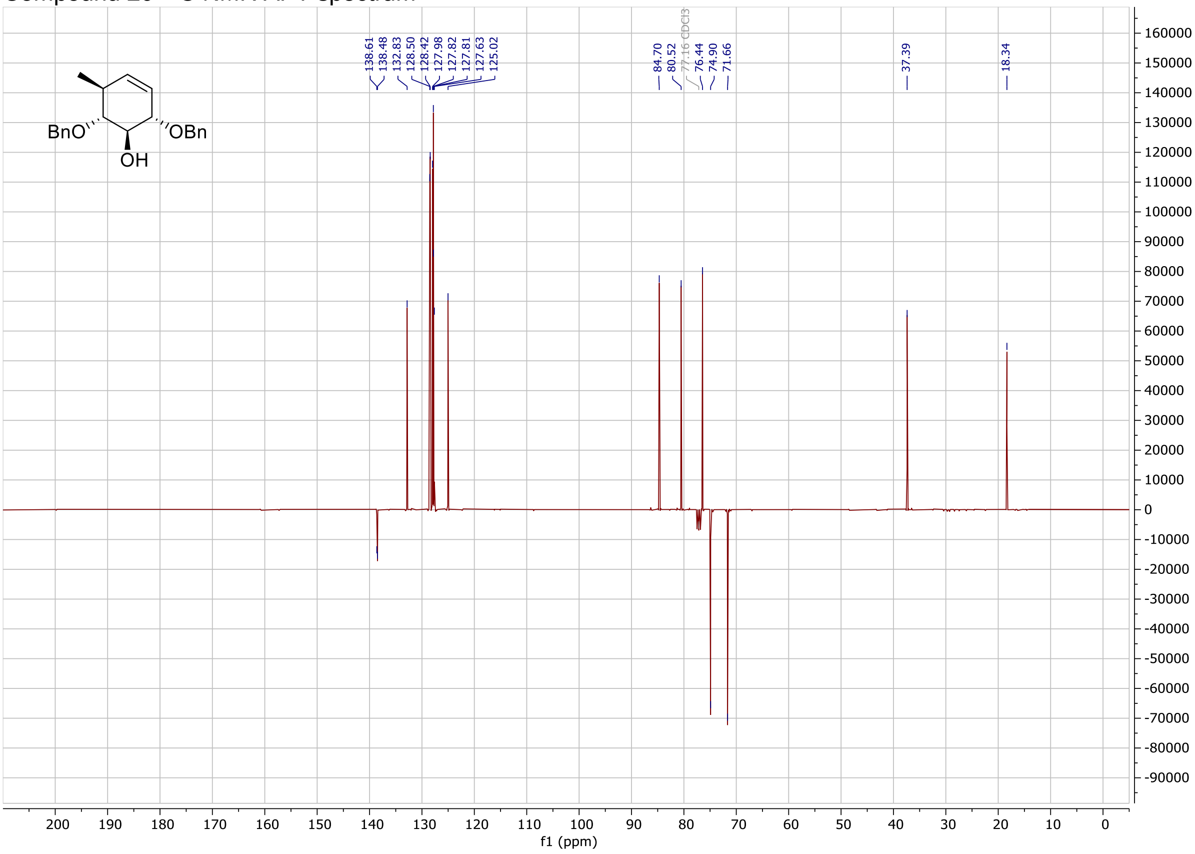

Compound **20** ^{1}H-^{1}H COSY spectrum

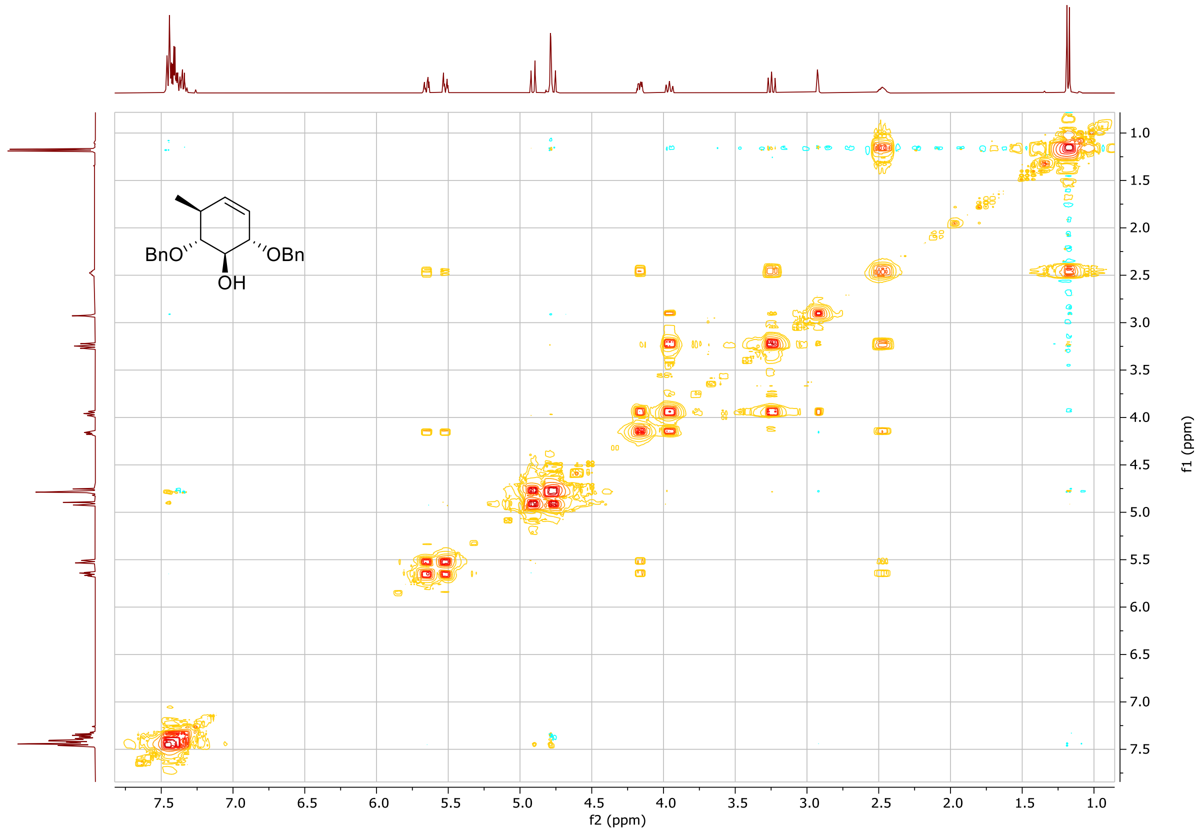

Compound **20** ^{1}H-^{13}C HSQC spectrum

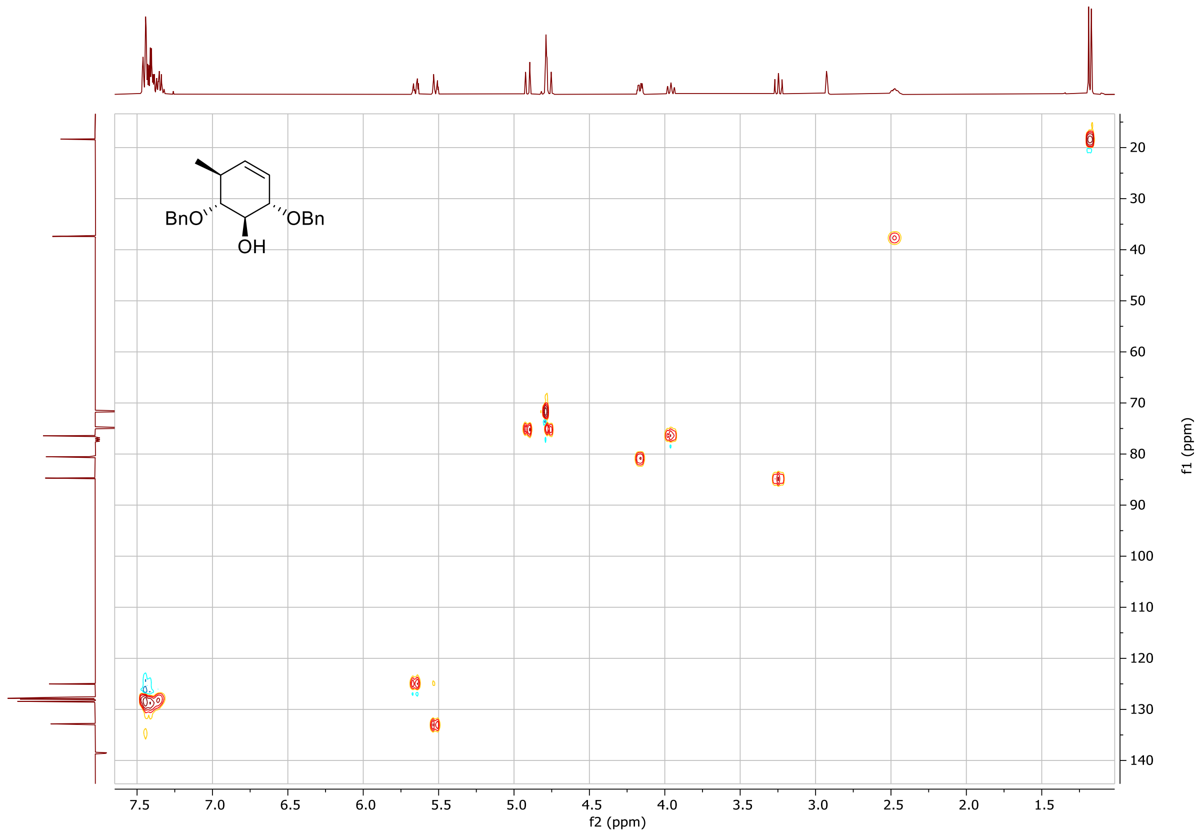

Compound **21** ^{1}H NMR spectrum

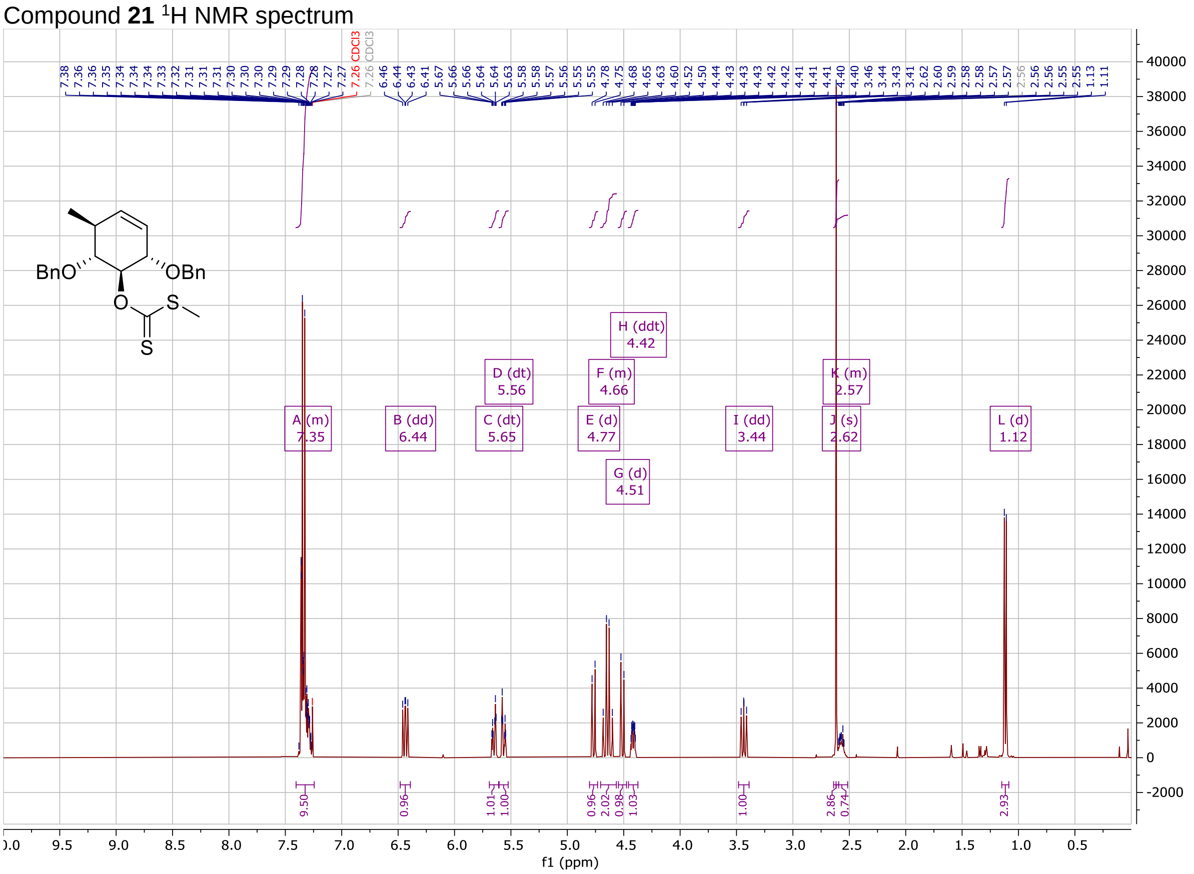

Compound **21** ^{13}C NMR APT spectrum

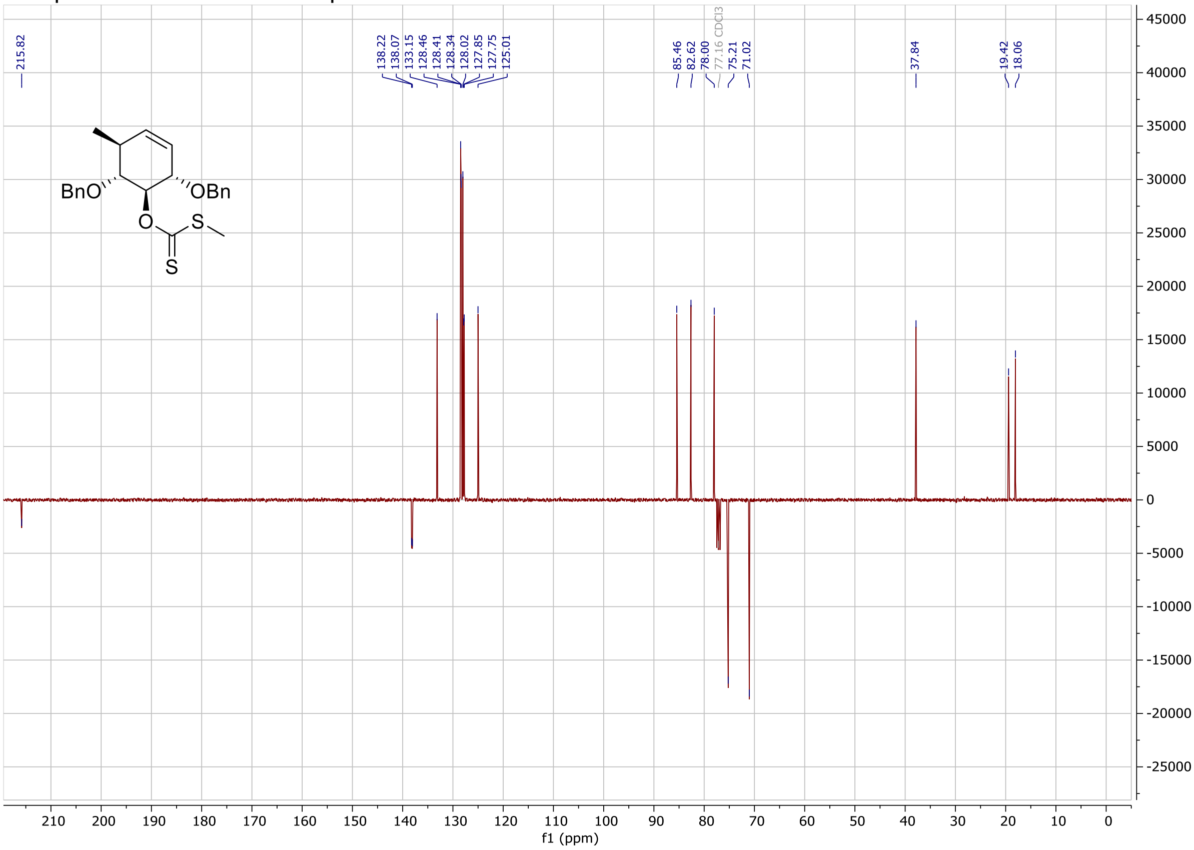

Compound **21** ^{1}H-^{1}H COSY spectrum

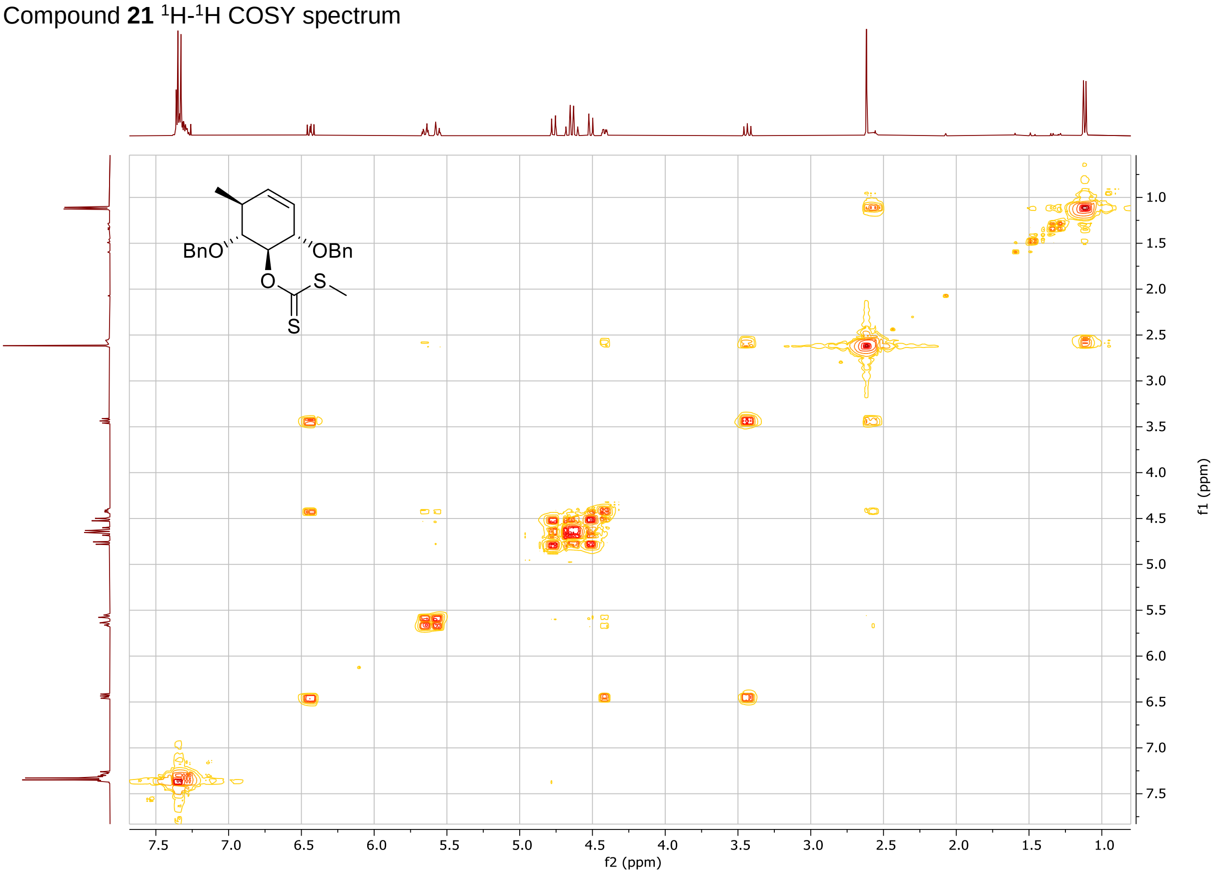

Compound **21** ^{1}H-^{13}C HSQC spectrum

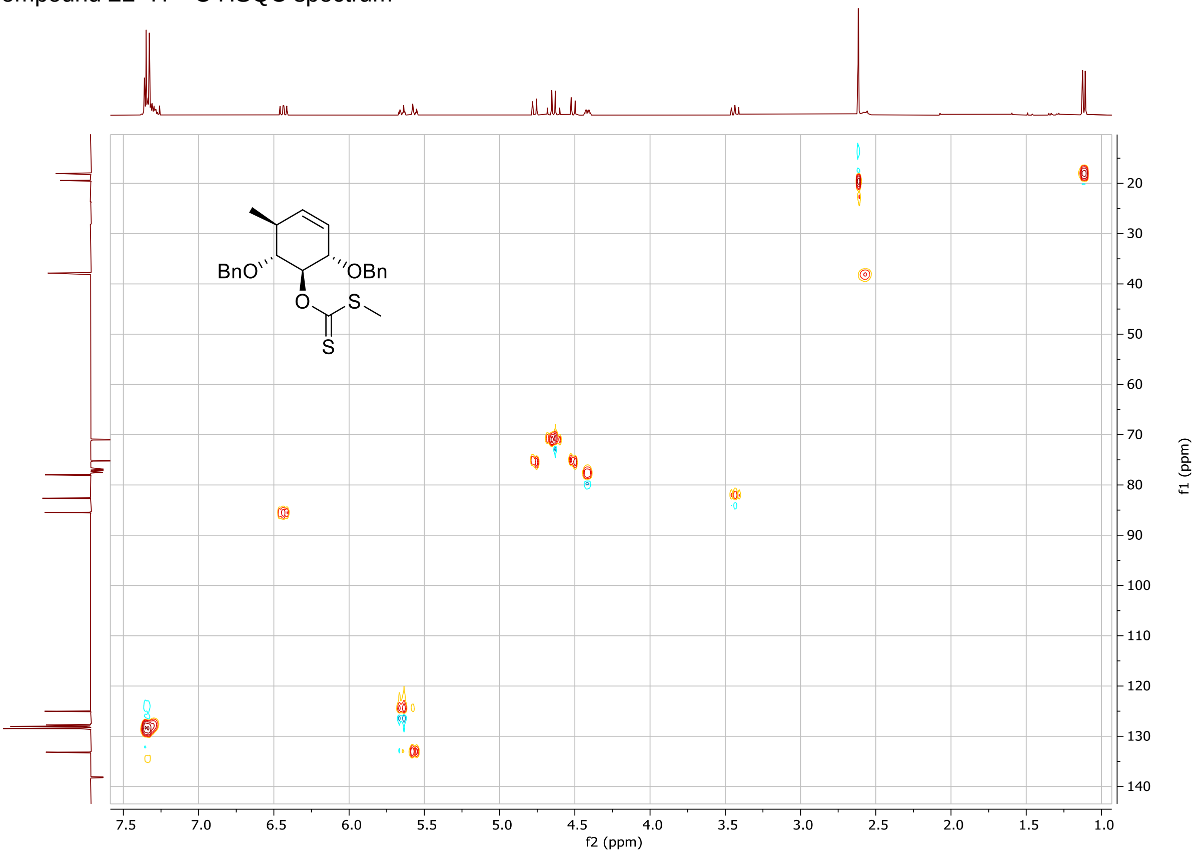

Compound **22** ^{1}H NMR spectrum

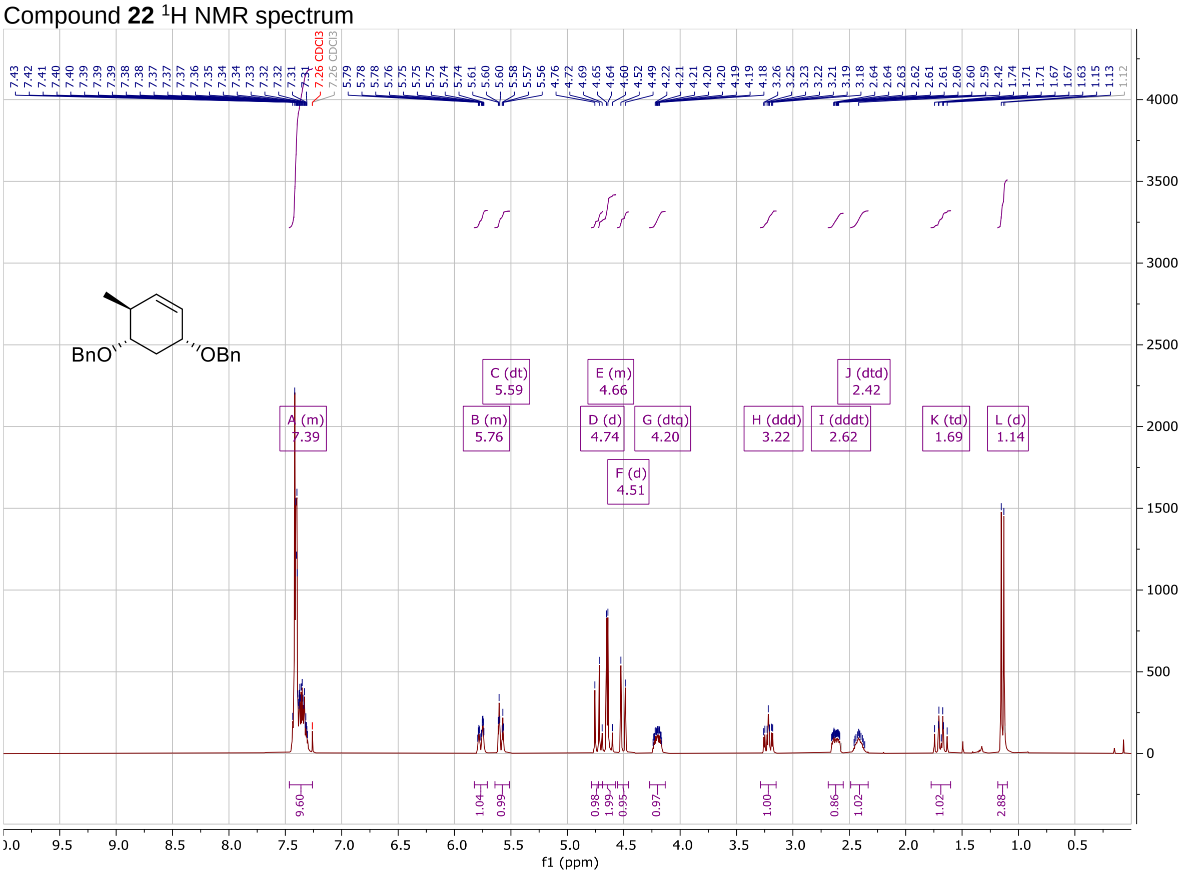

Compound **22** ^{13}C NMR APT spectrum

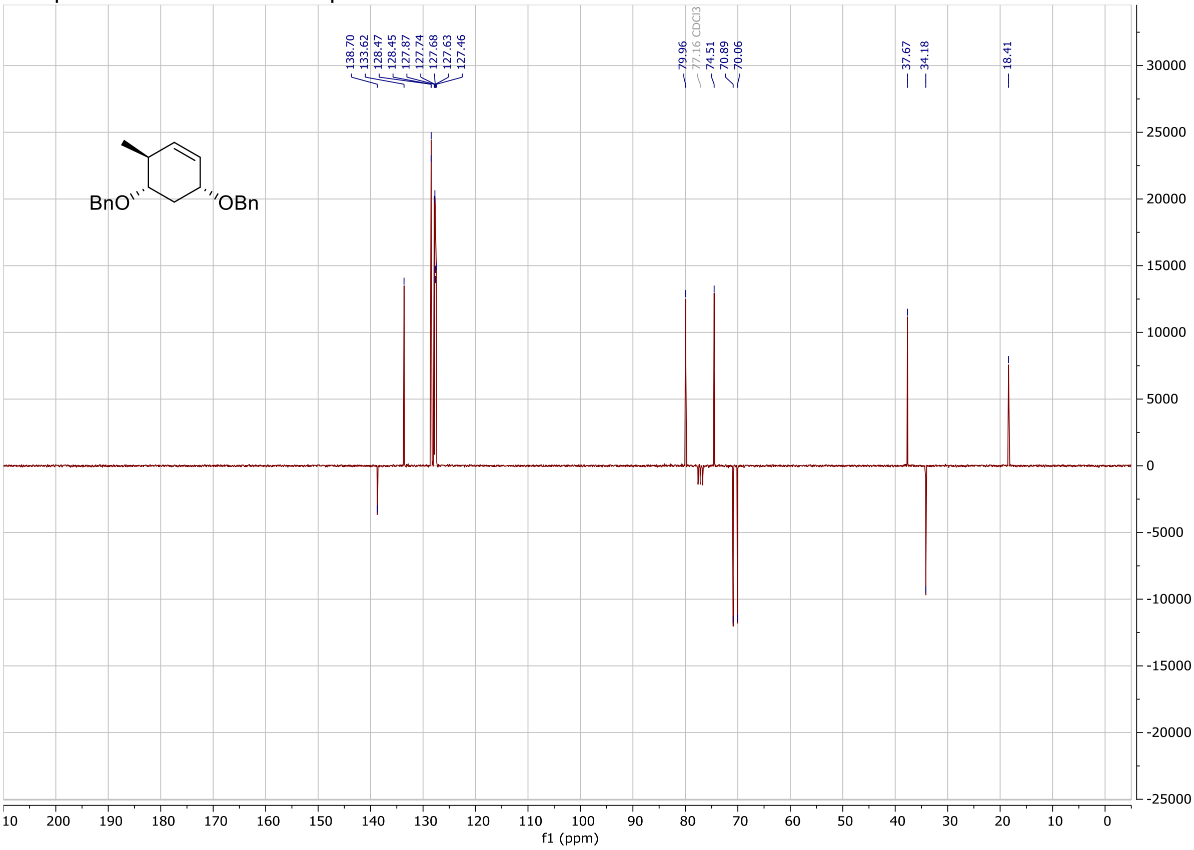

Compound **23** ^{1}H NMR spectrum

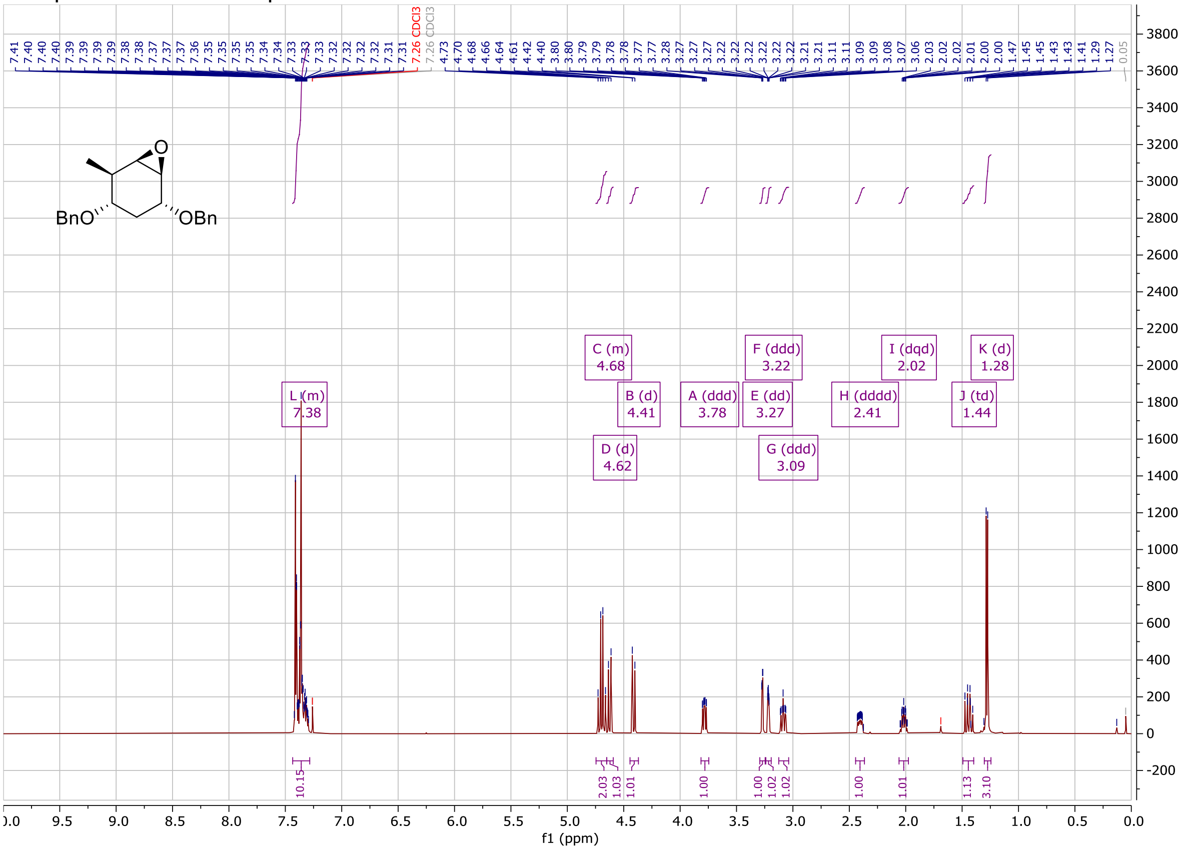

Compound **23** ^{13}C NMR APT spectrum

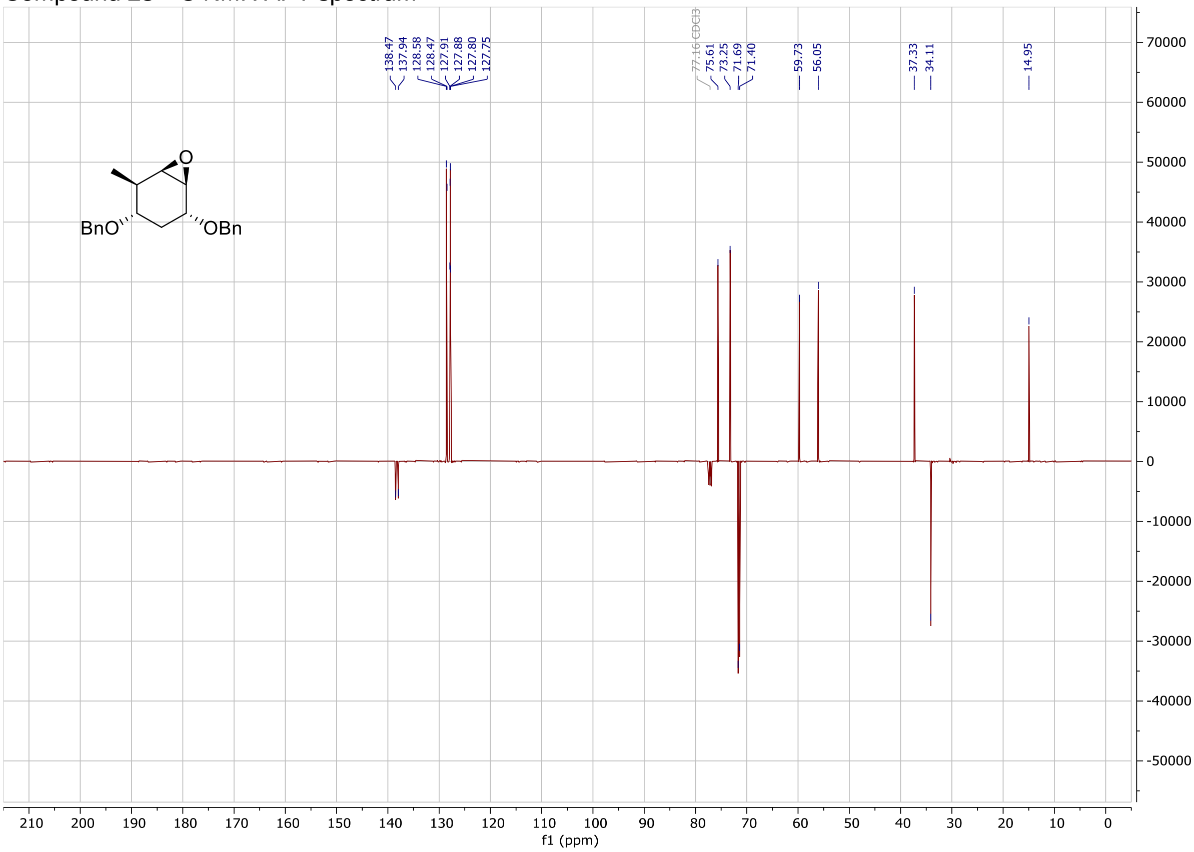

Compound **23** ^{1}H-^{1}H COSY spectrum

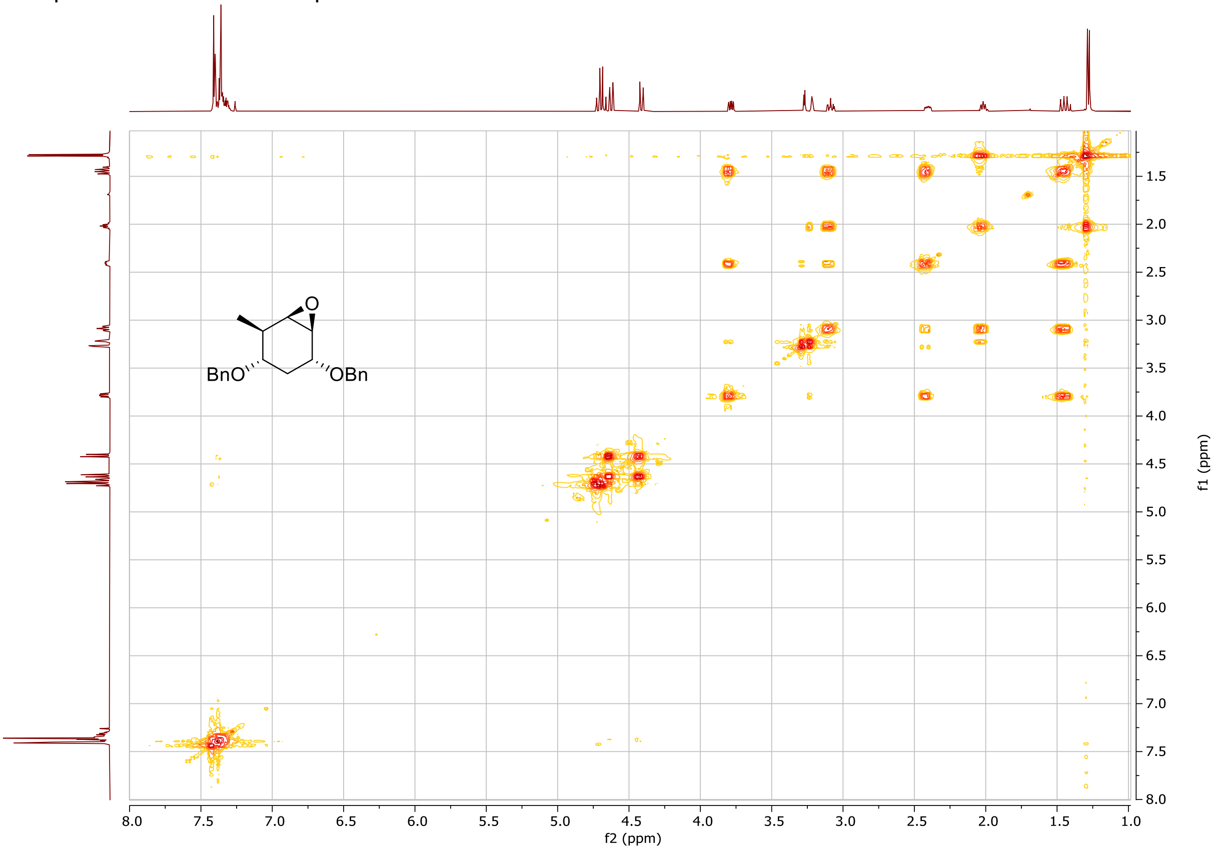

Compound **23** ^{1}H-^{13}C HSQC spectrum

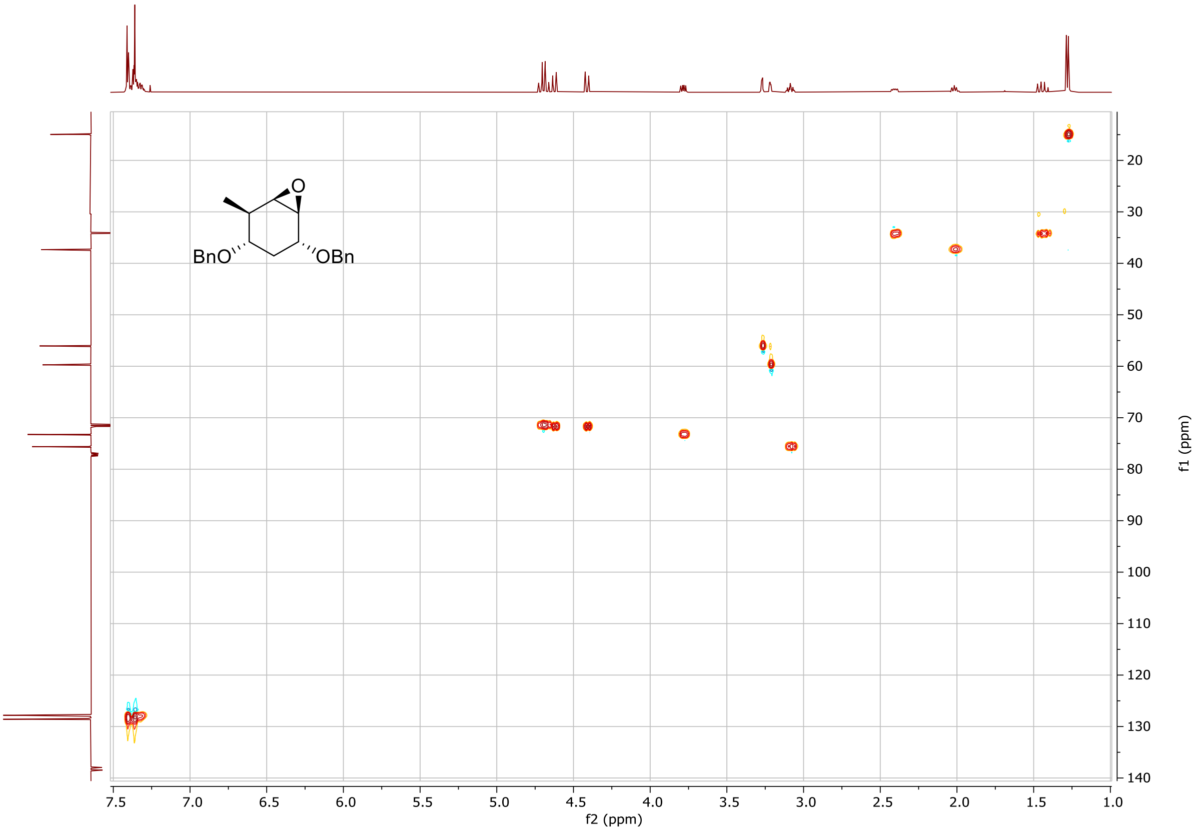

Compound **23** ^{1}H-^{1}H NOESY spectrum

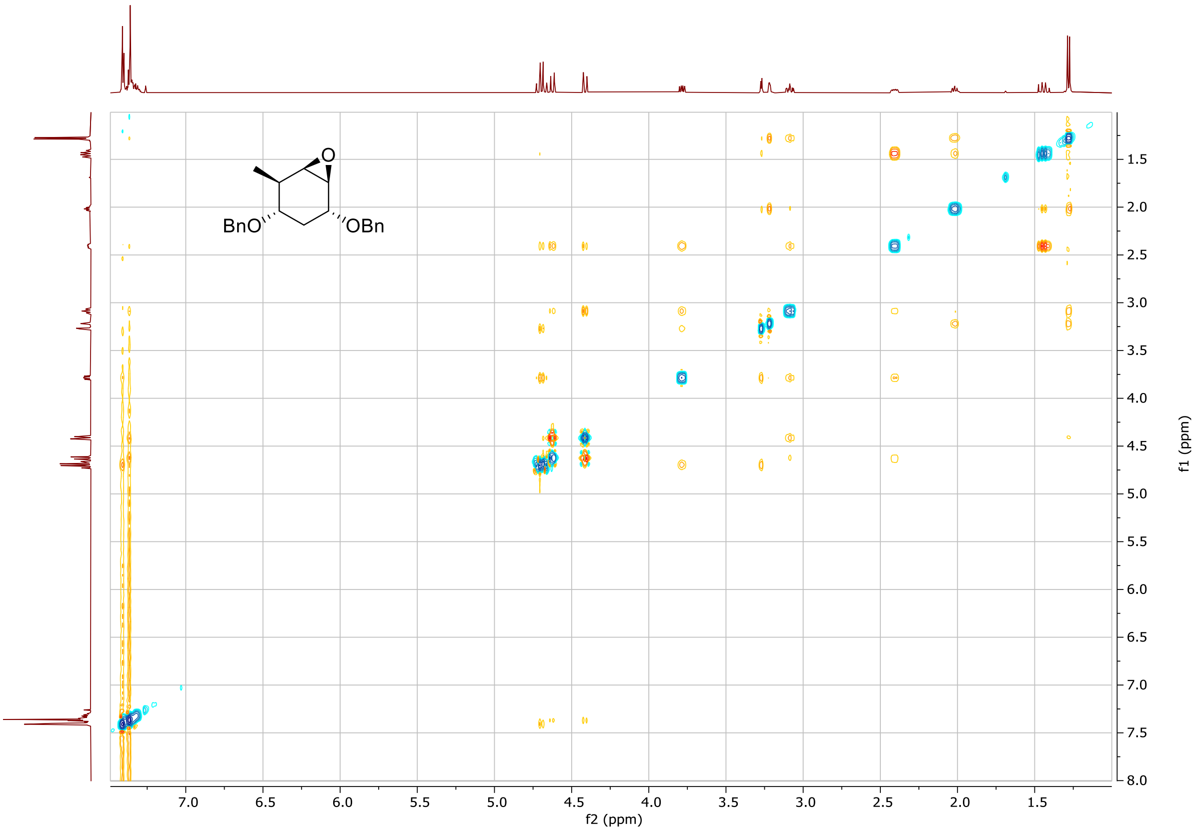

Compound **24** ^{1}H NMR spectrum

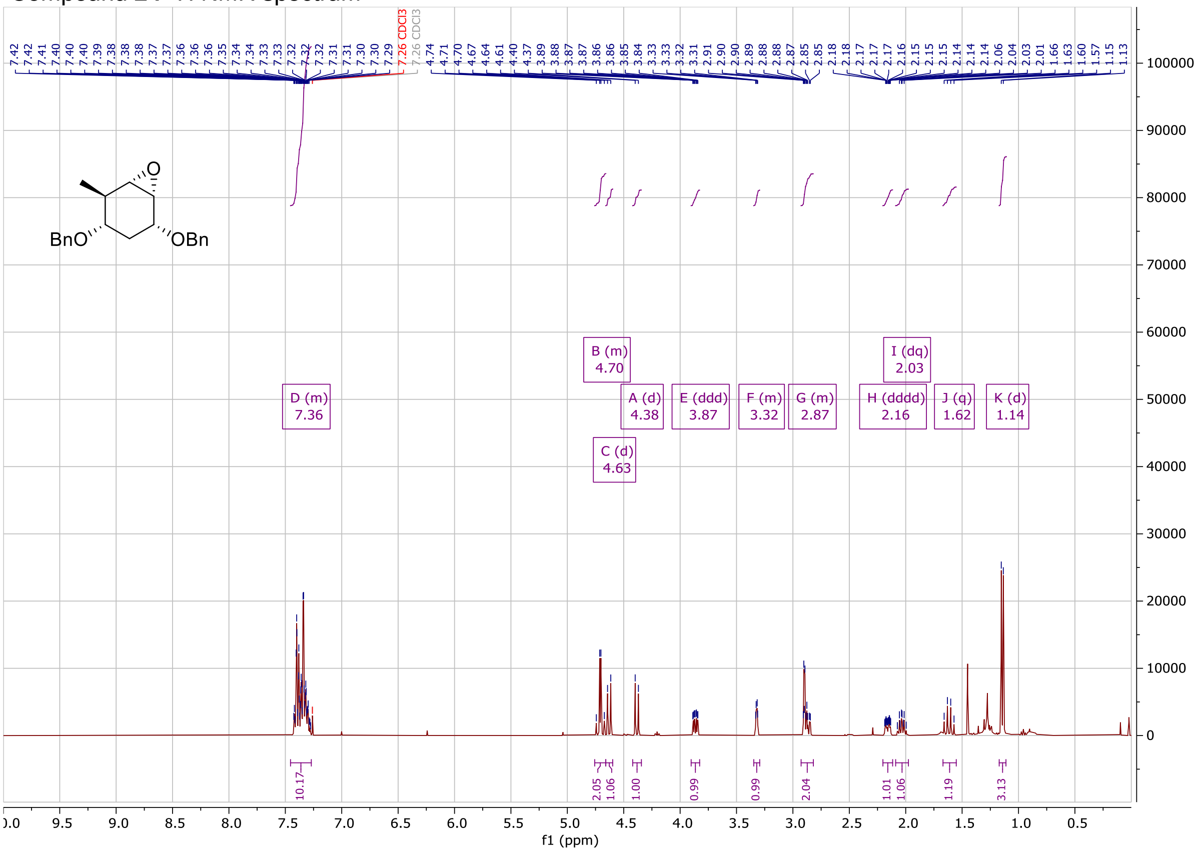

Compound **24** ^{13}C NMR APT spectrum

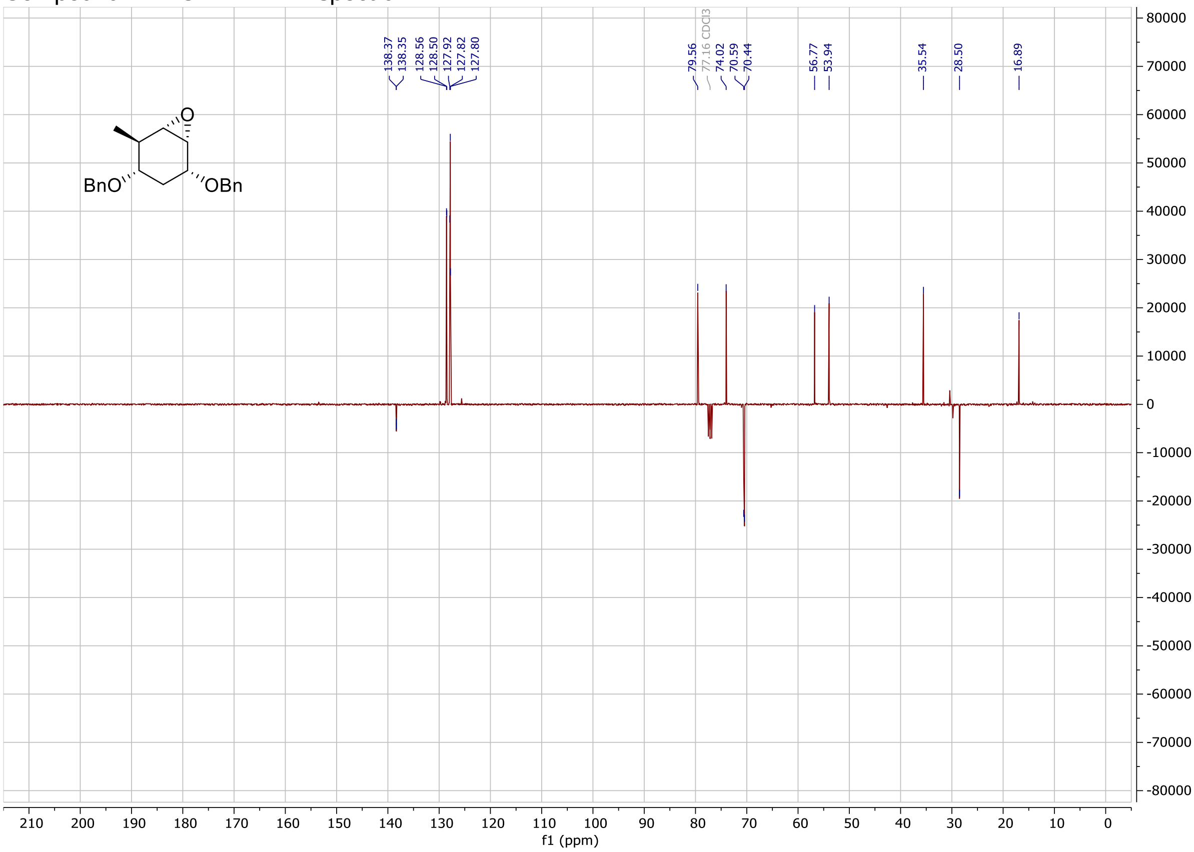

Compound **24** ^{1}H-^{1}H COSY spectrum

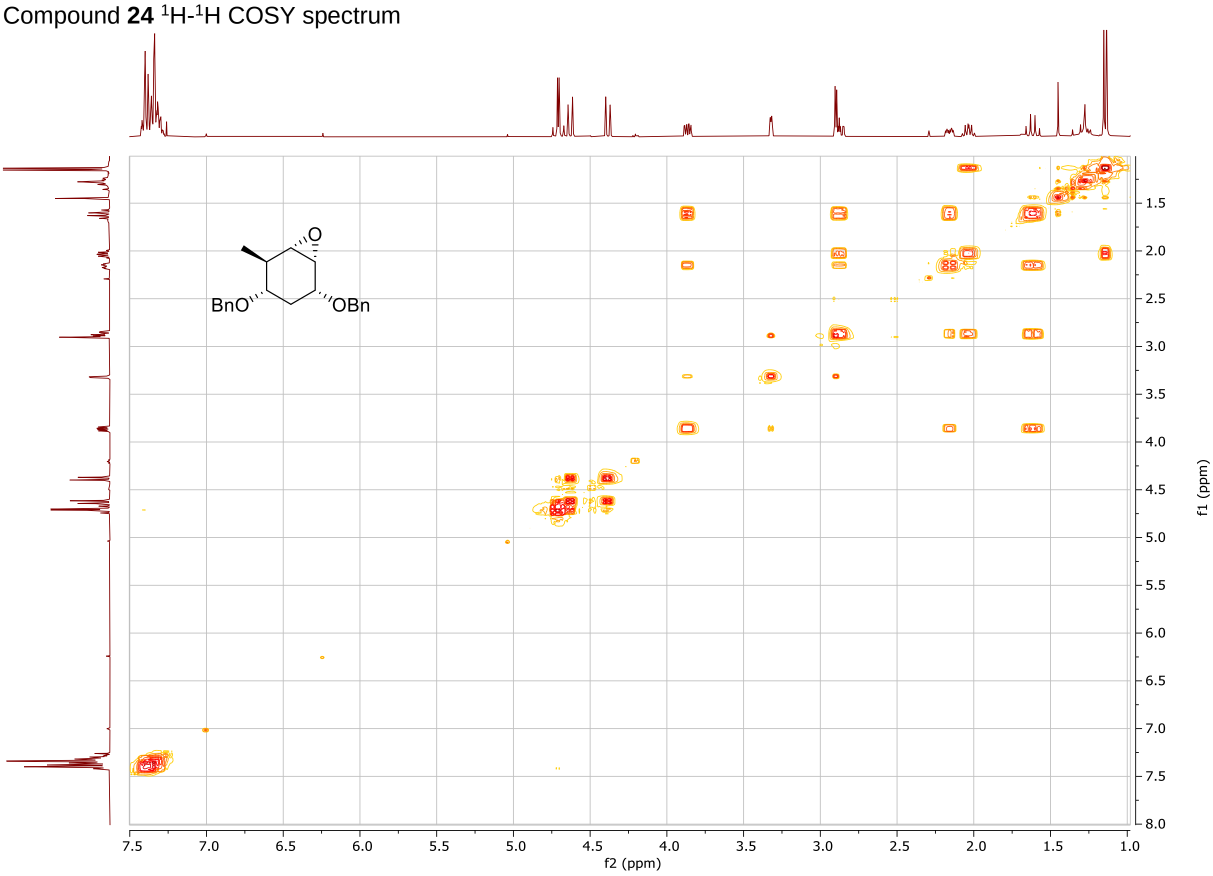

Compound **24** ^{1}H-^{13}C HSQC spectrum

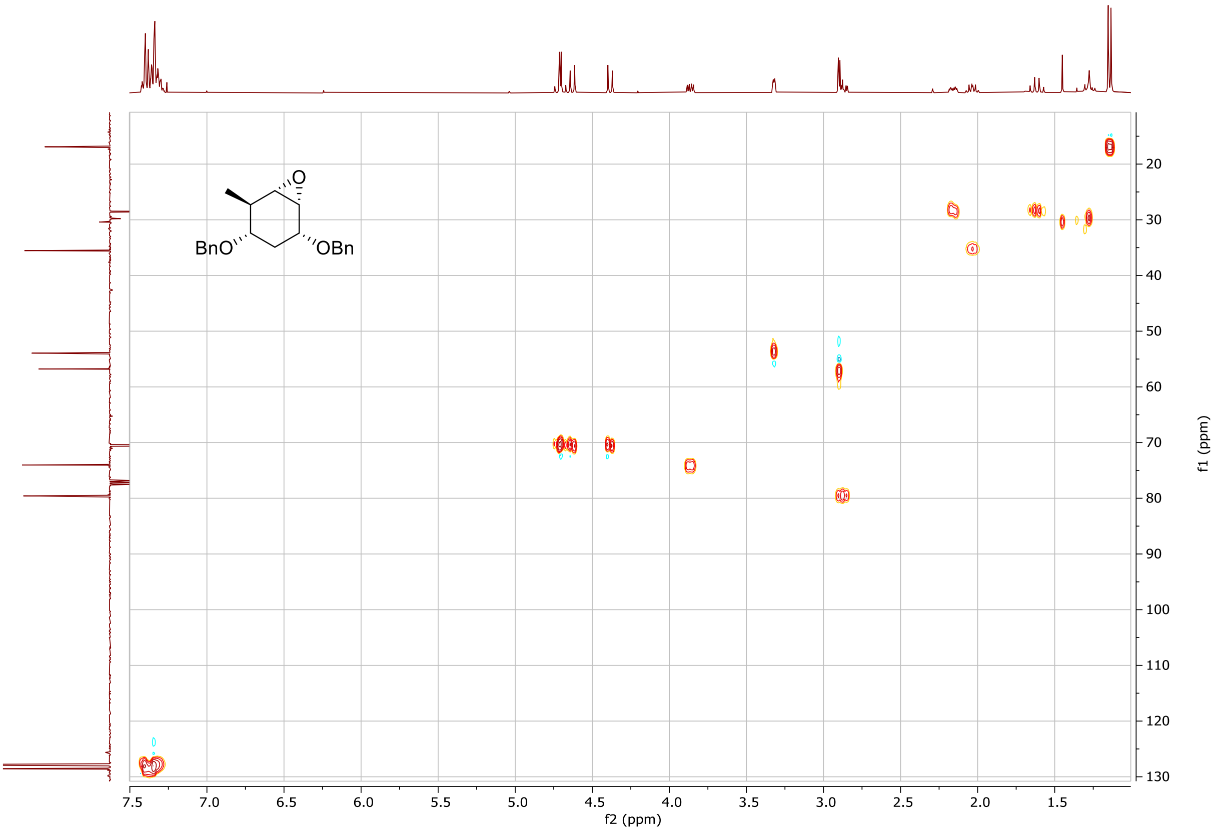

Compound **25** ^{1}H NMR spectrum

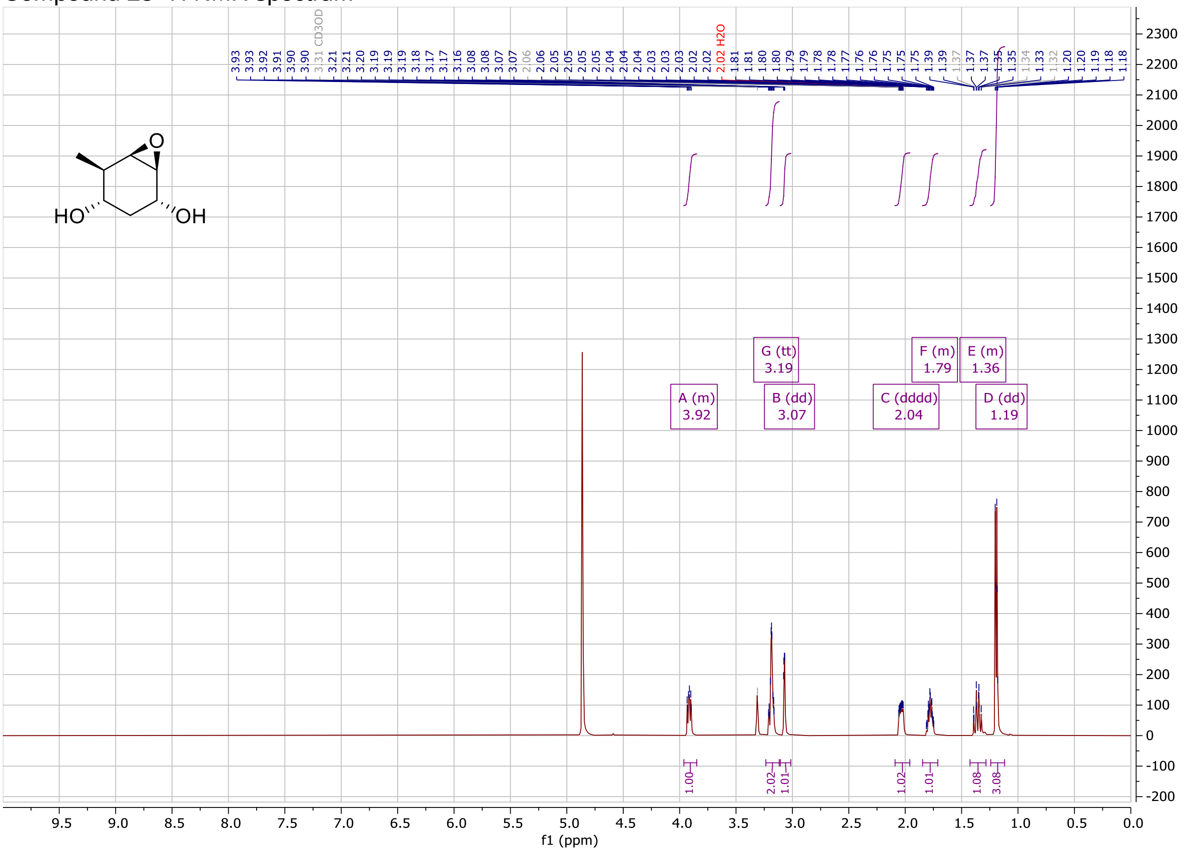

Compound **25** ^{13}C NMR APT spectrum

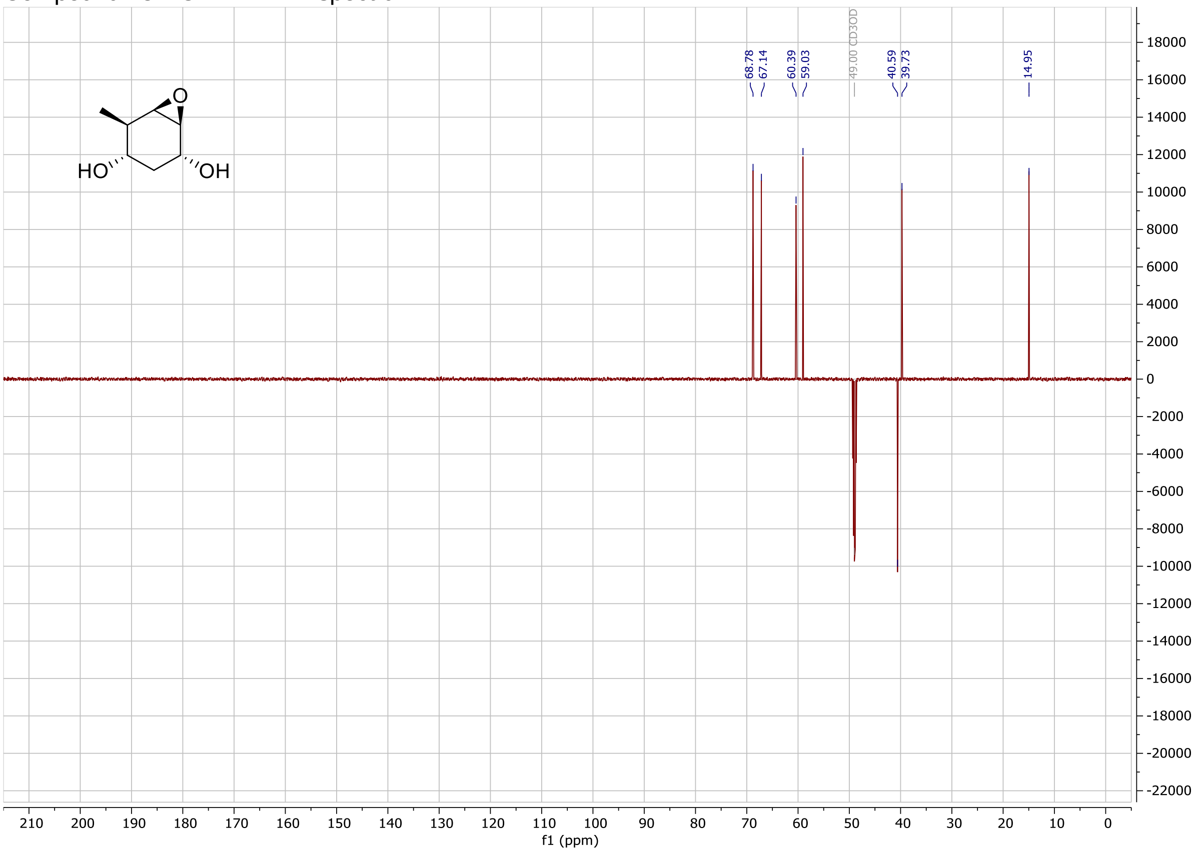

Compound **25** ^{1}H-^{1}H COSY spectrum

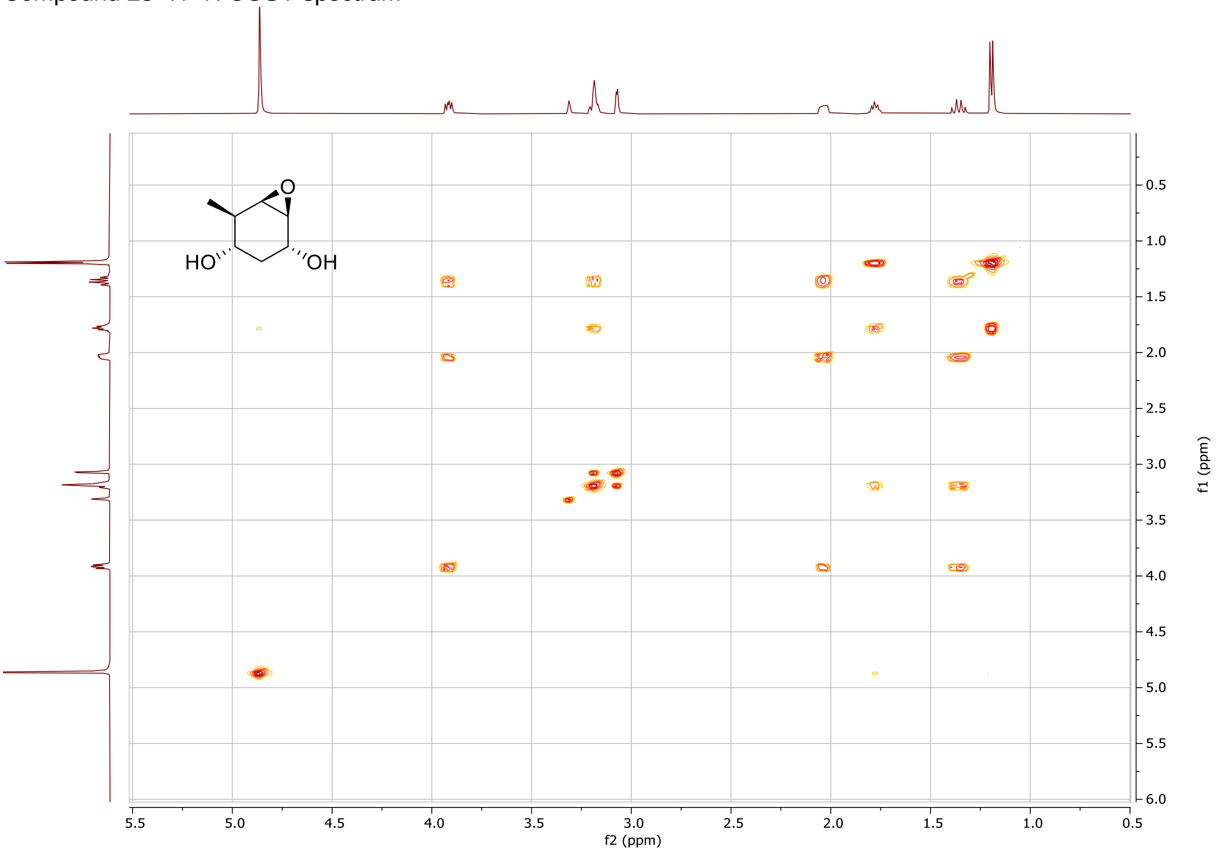

Compound **25** ^{1}H-^{13}C HSQC spectrum

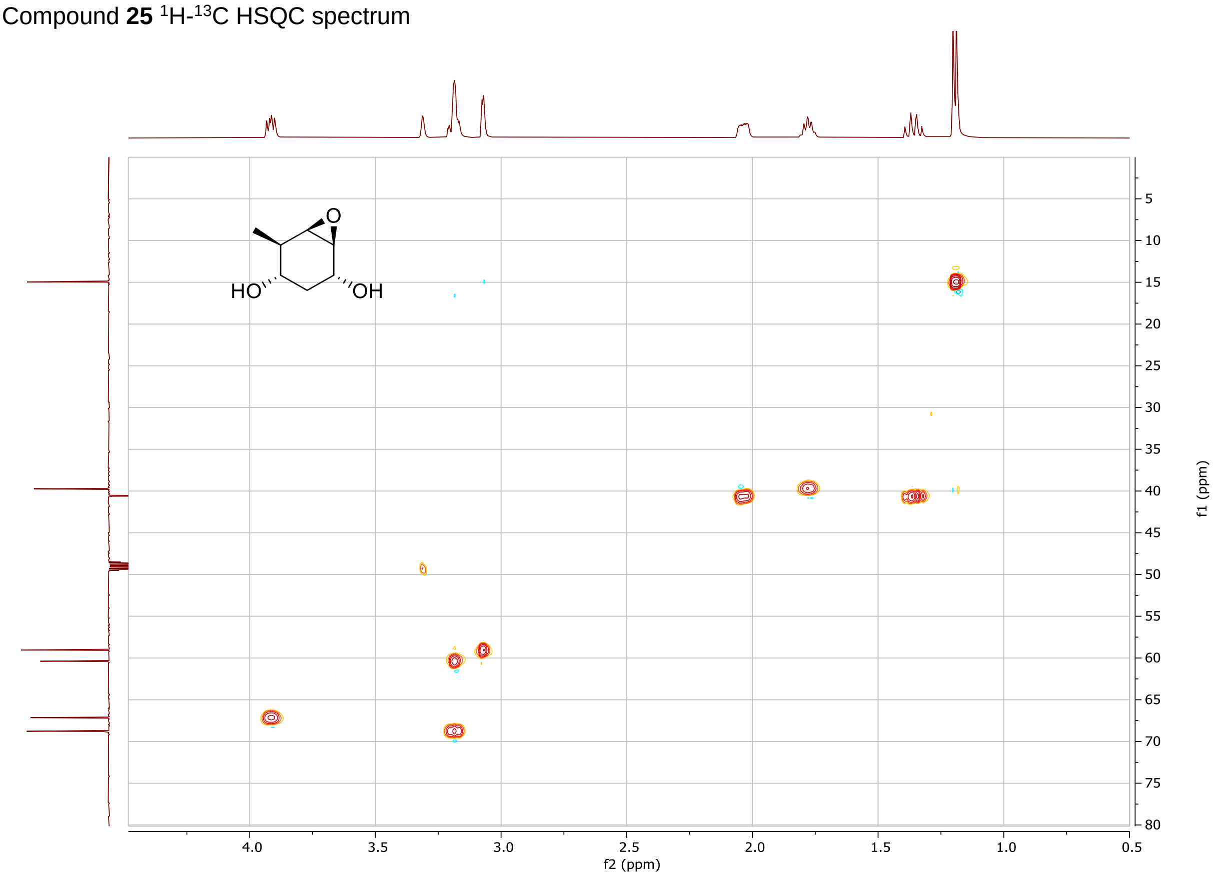

Compound **26** ^{1}H NMR spectrum

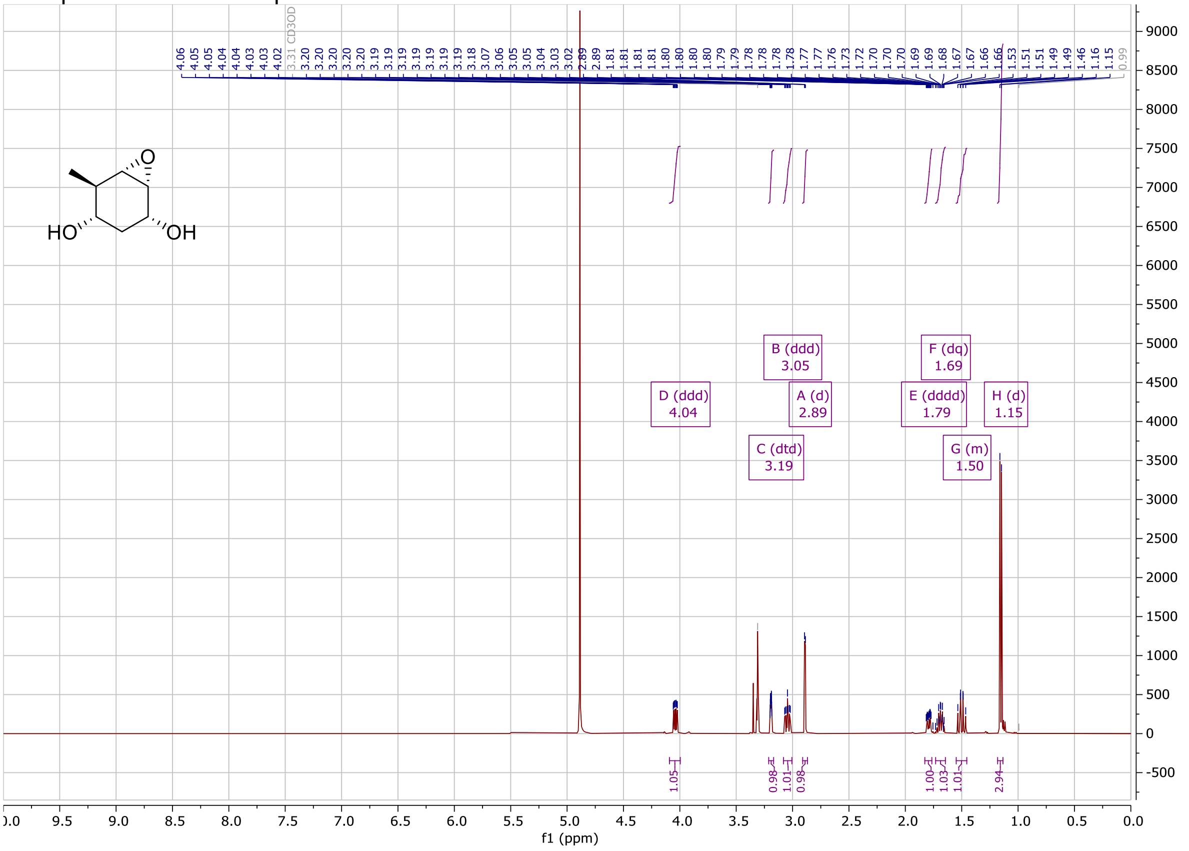

Compound **26** ^{13}C NMR APT spectrum

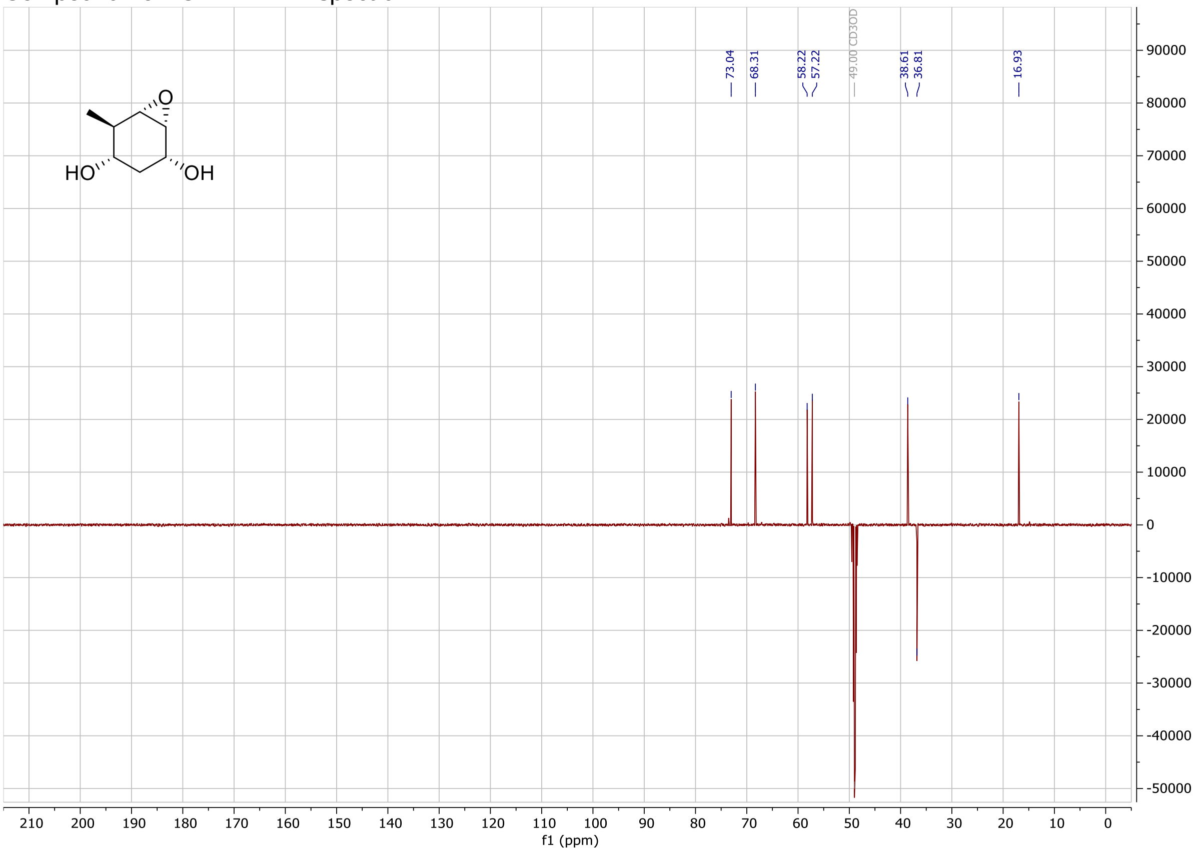

Compound **26** ^{1}H-^{1}H COSY spectrum

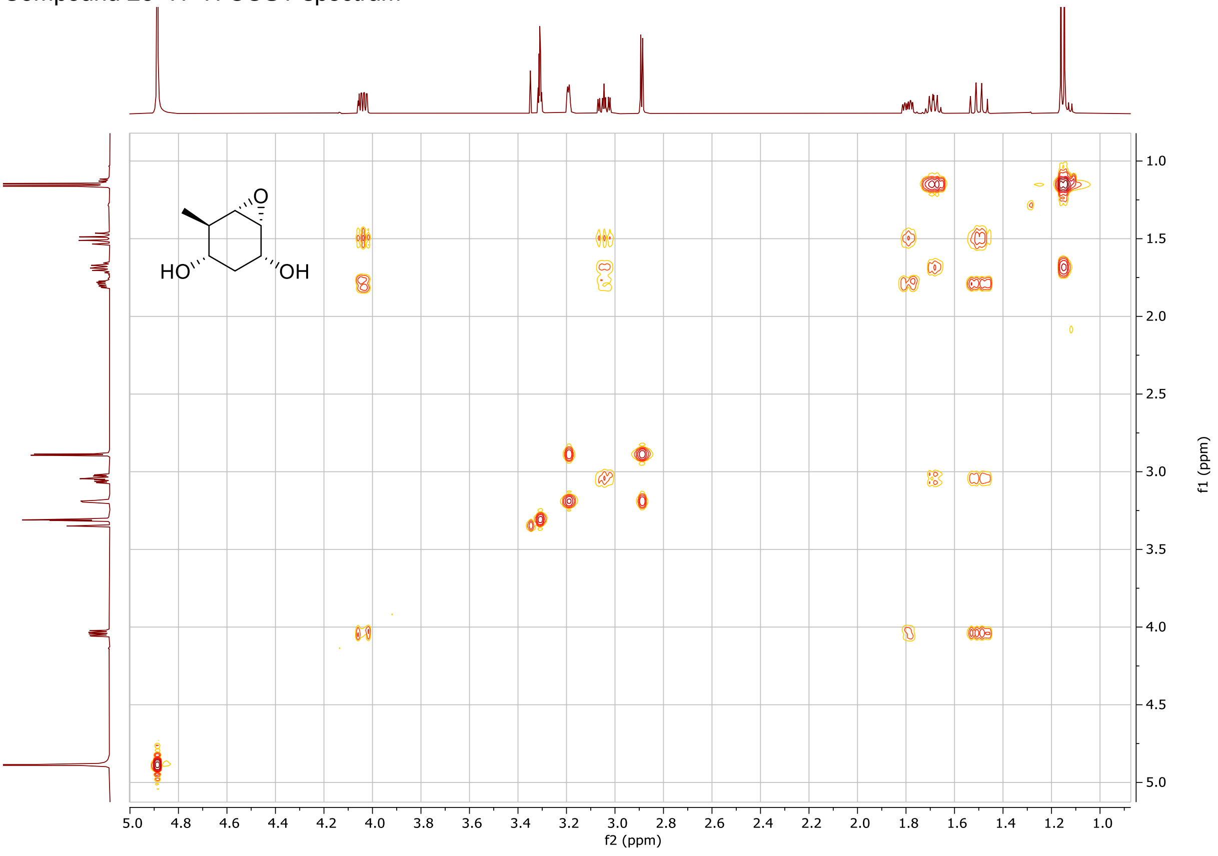

Compound **26** ^{1}H-^{13}C HSQC spectrum

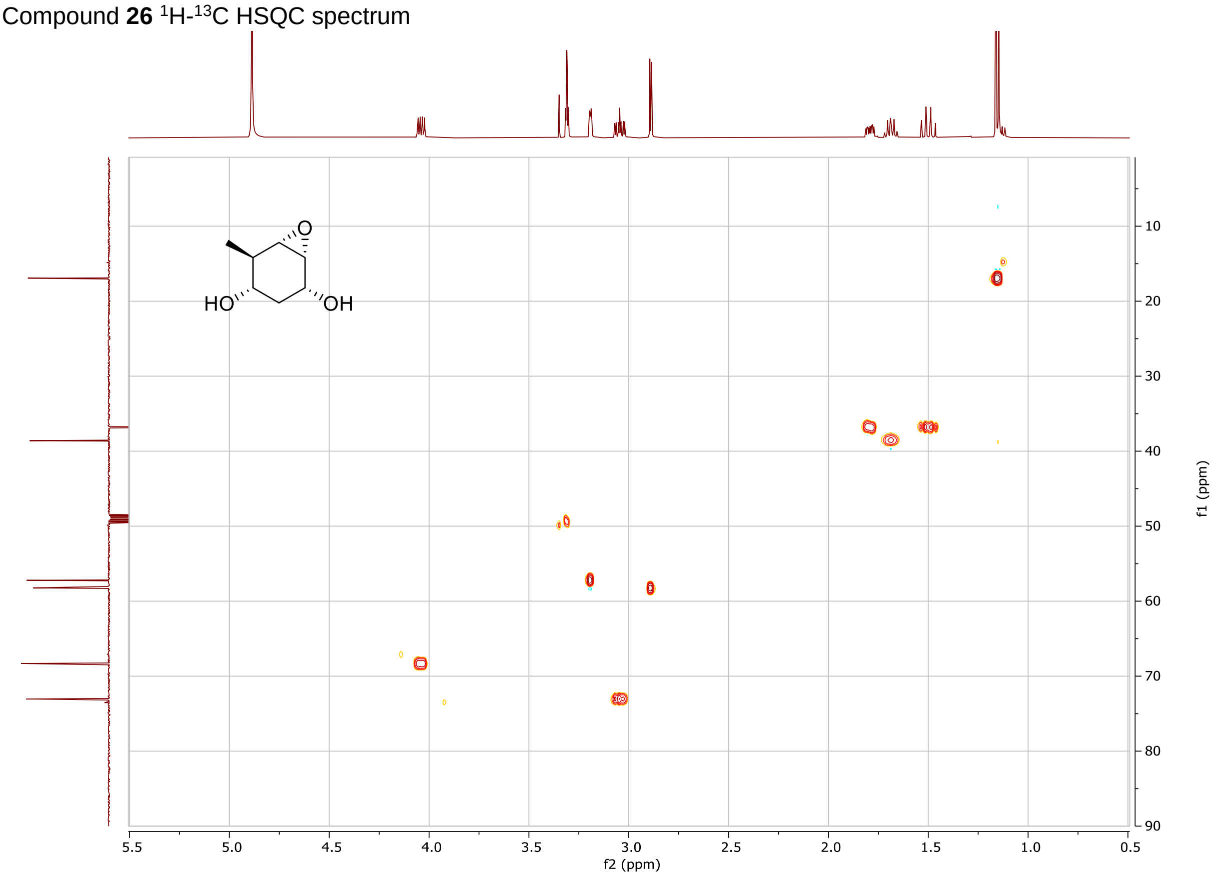

Compound **27** ^{1}H NMR spectrum

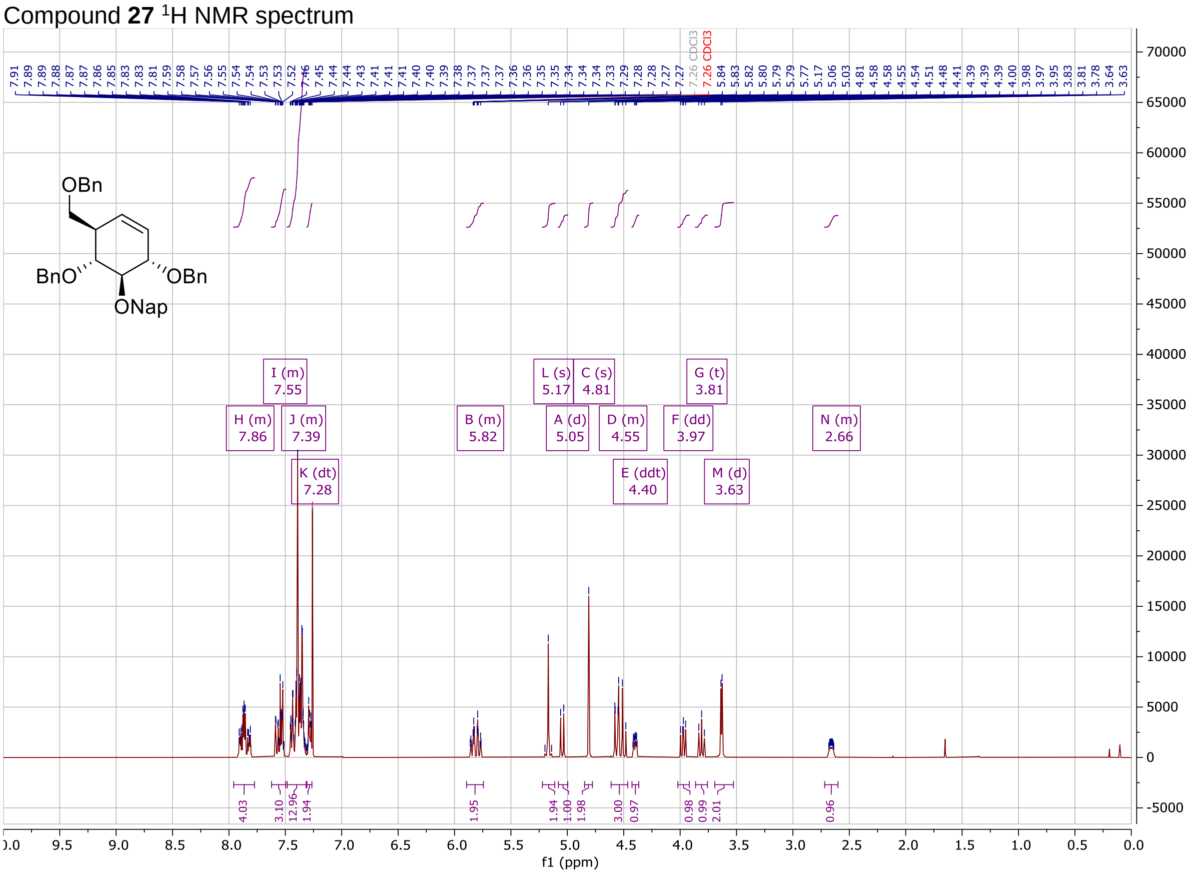

Compound **27** ^{13}C NMR APT spectrum

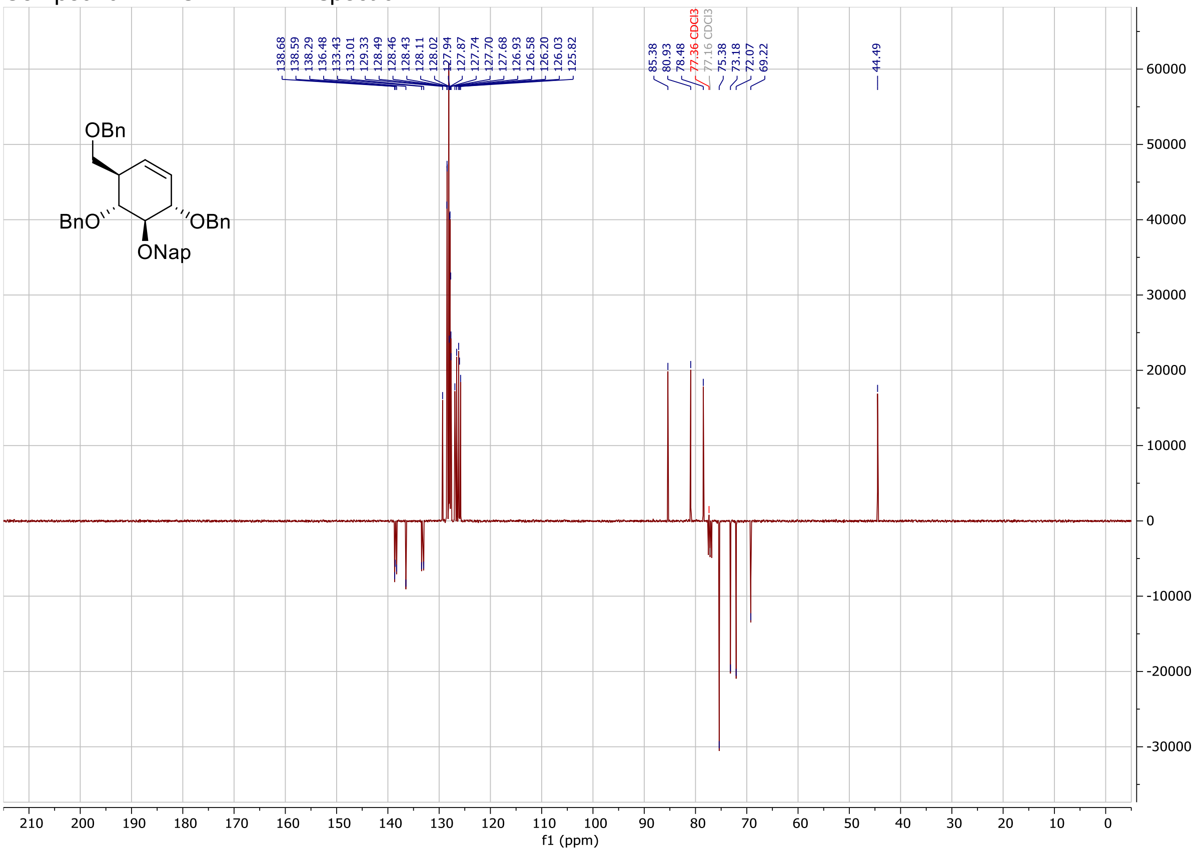

Compound **27** ^{1}H-^{1}H COSY spectrum

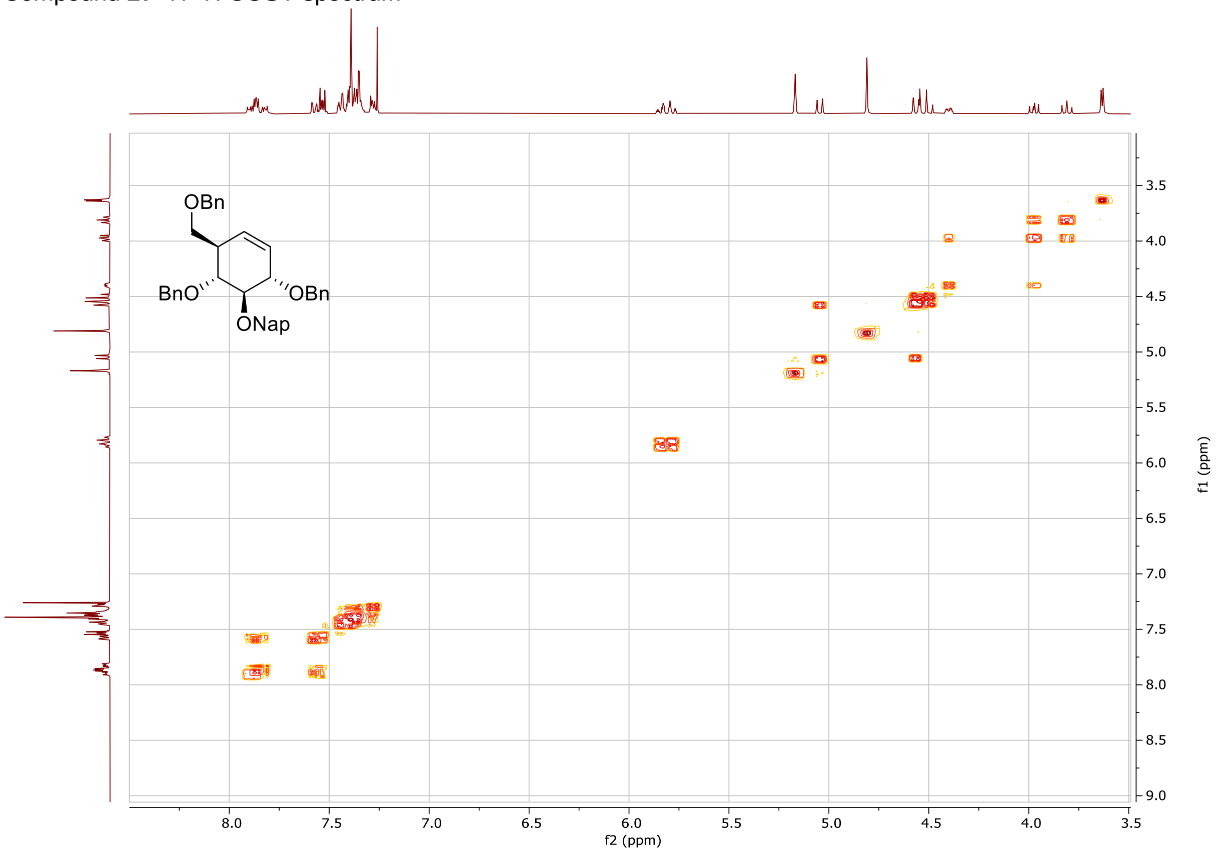

Compound **27** ^{1}H-^{13}C HSQC spectrum

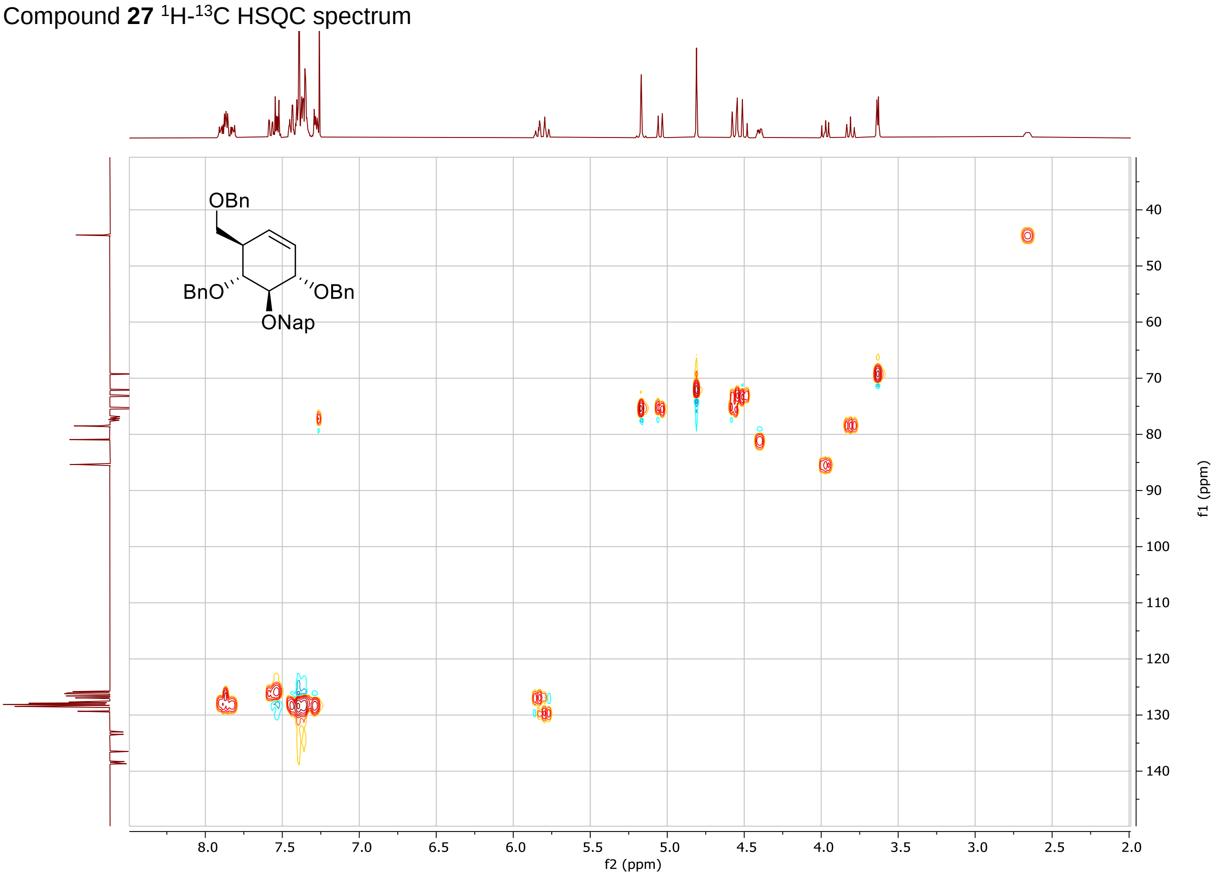

Compound **28** ^{1}H NMR spectrum

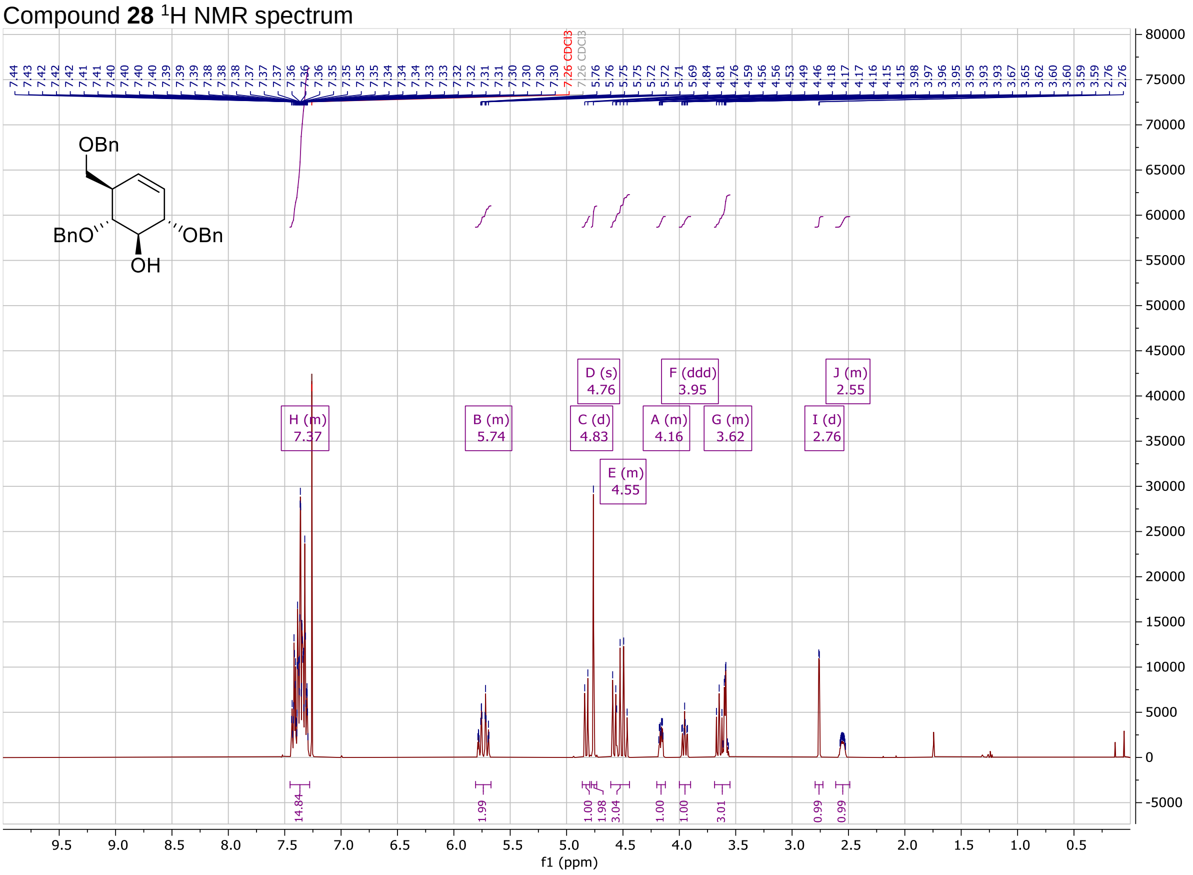

Compound **28** ^{13}C NMR APT spectrum

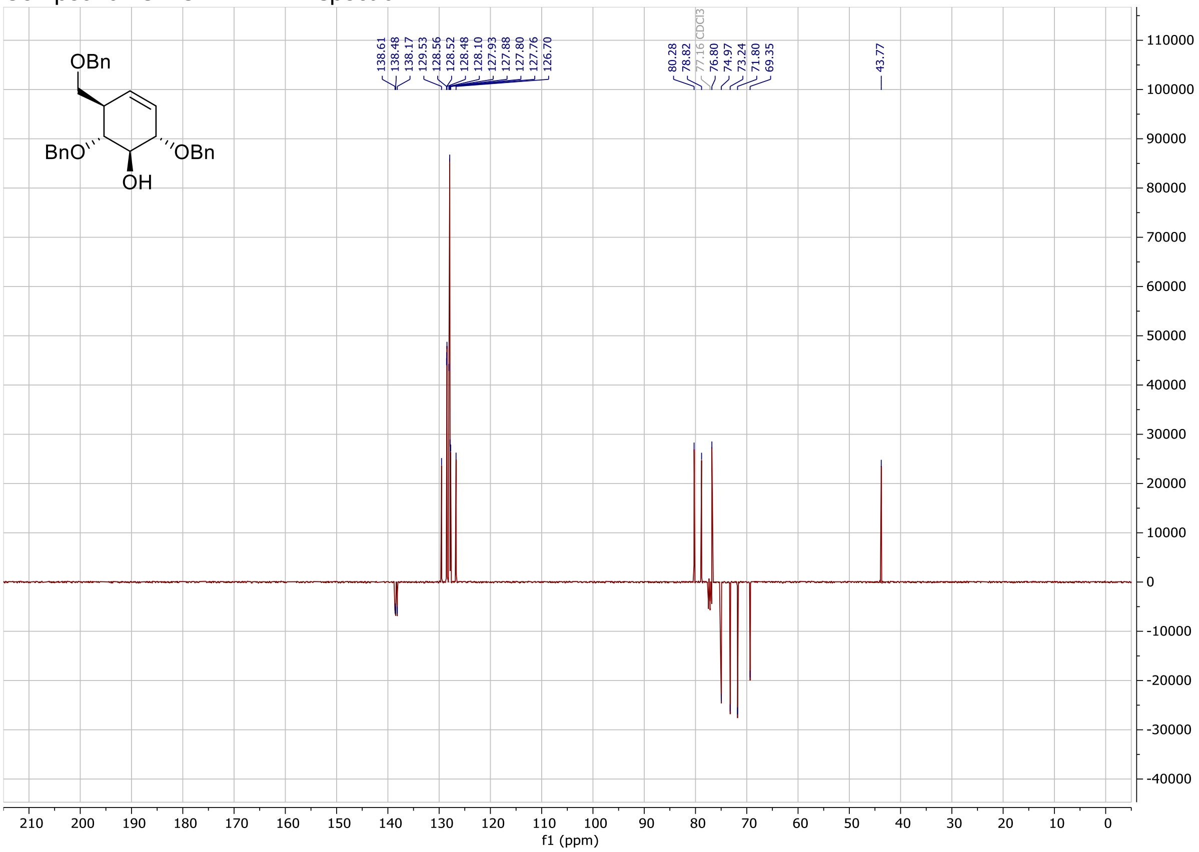

Compound **28** ^{1}H-^{1}H COSY spectrum

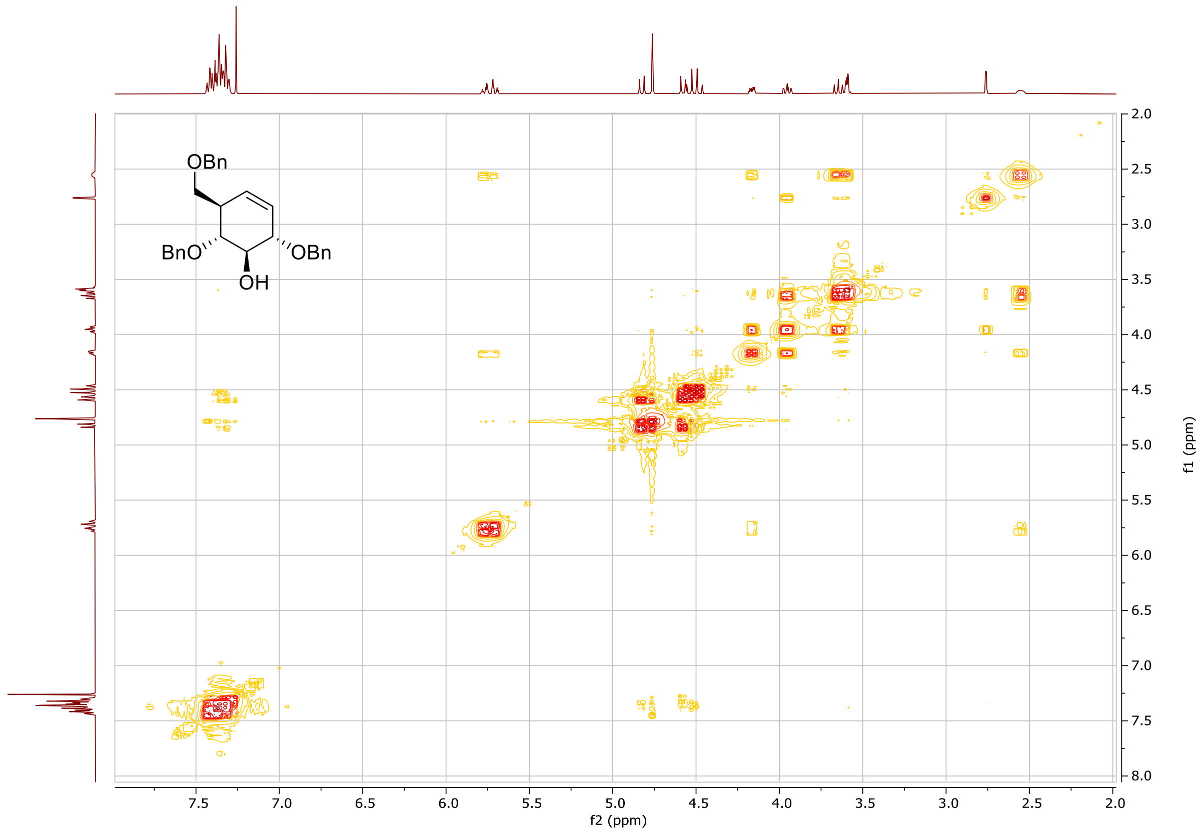

Compound **28** ^{1}H-^{13}C HSQC spectrum

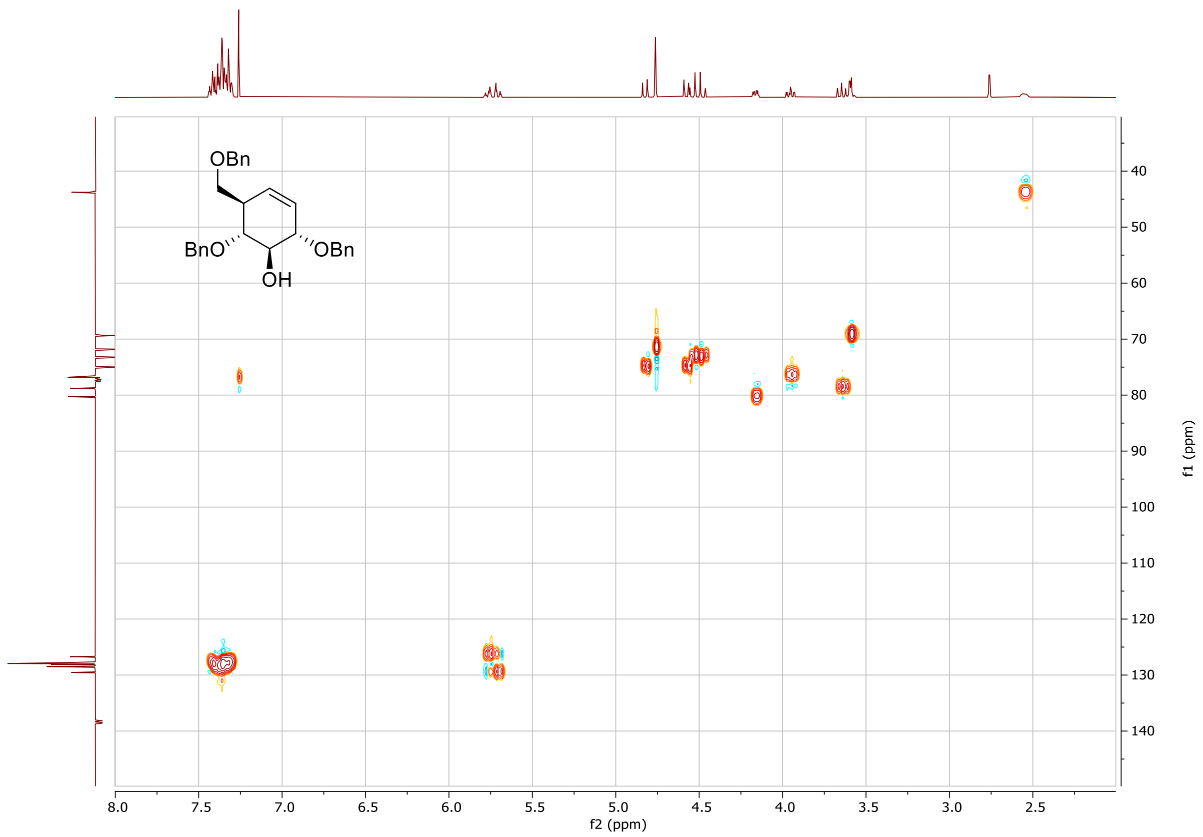

Compound **29** ^{1}H NMR spectrum

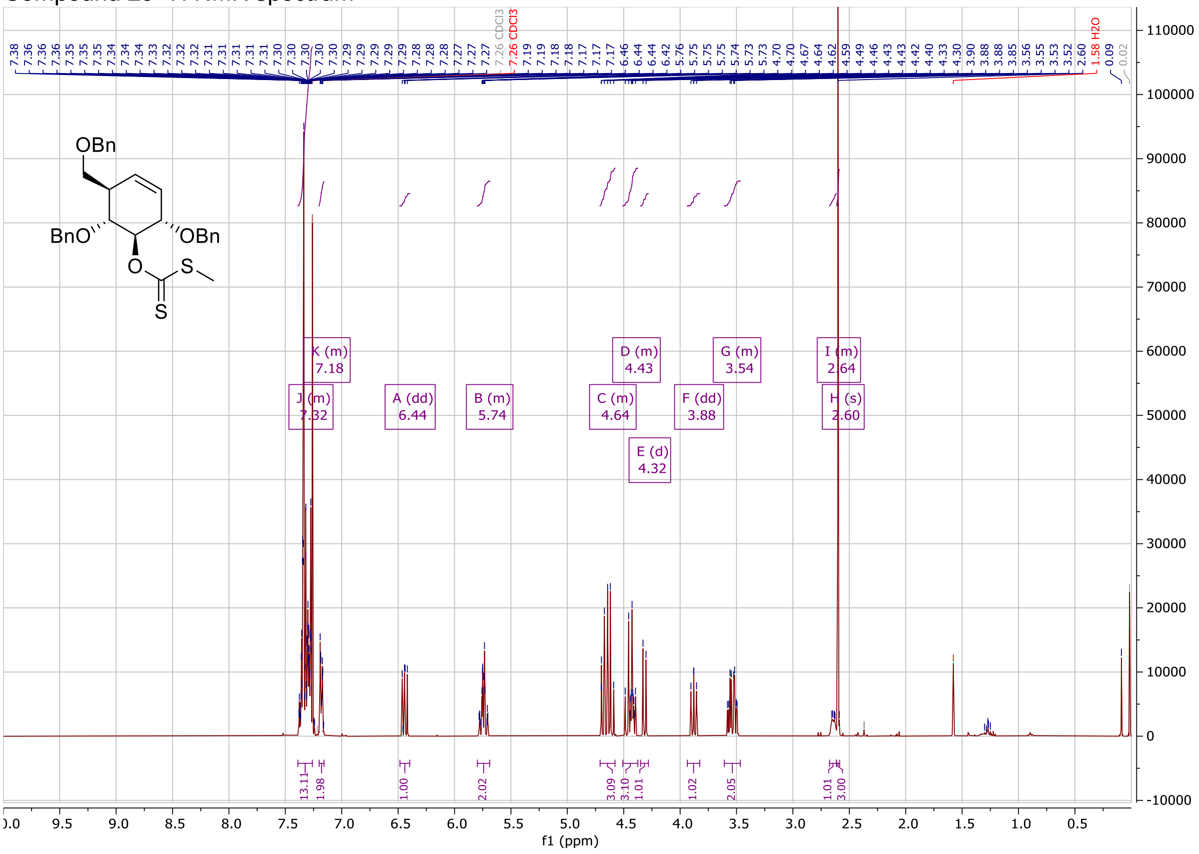

Compound **29** ^{13}C NMR APT spectrum

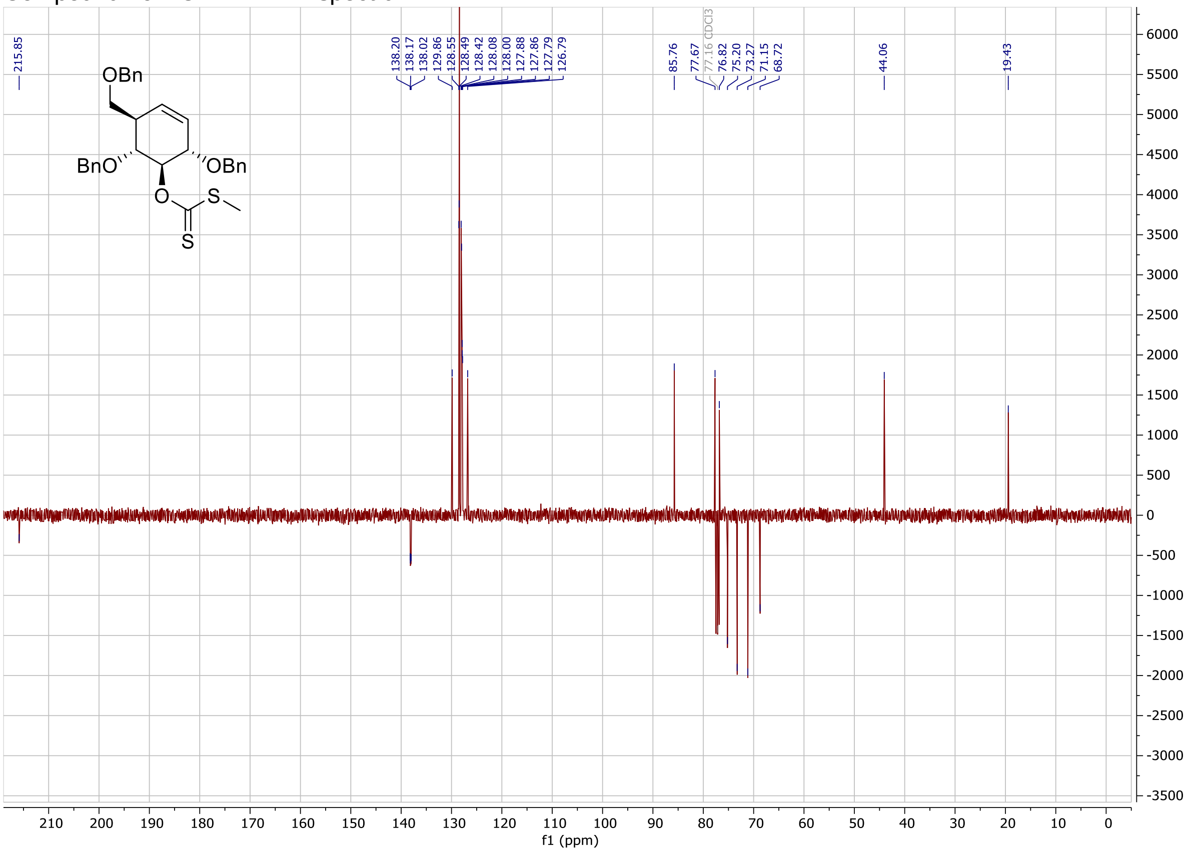

Compound **29** ^{1}H-^{1}H COSY spectrum

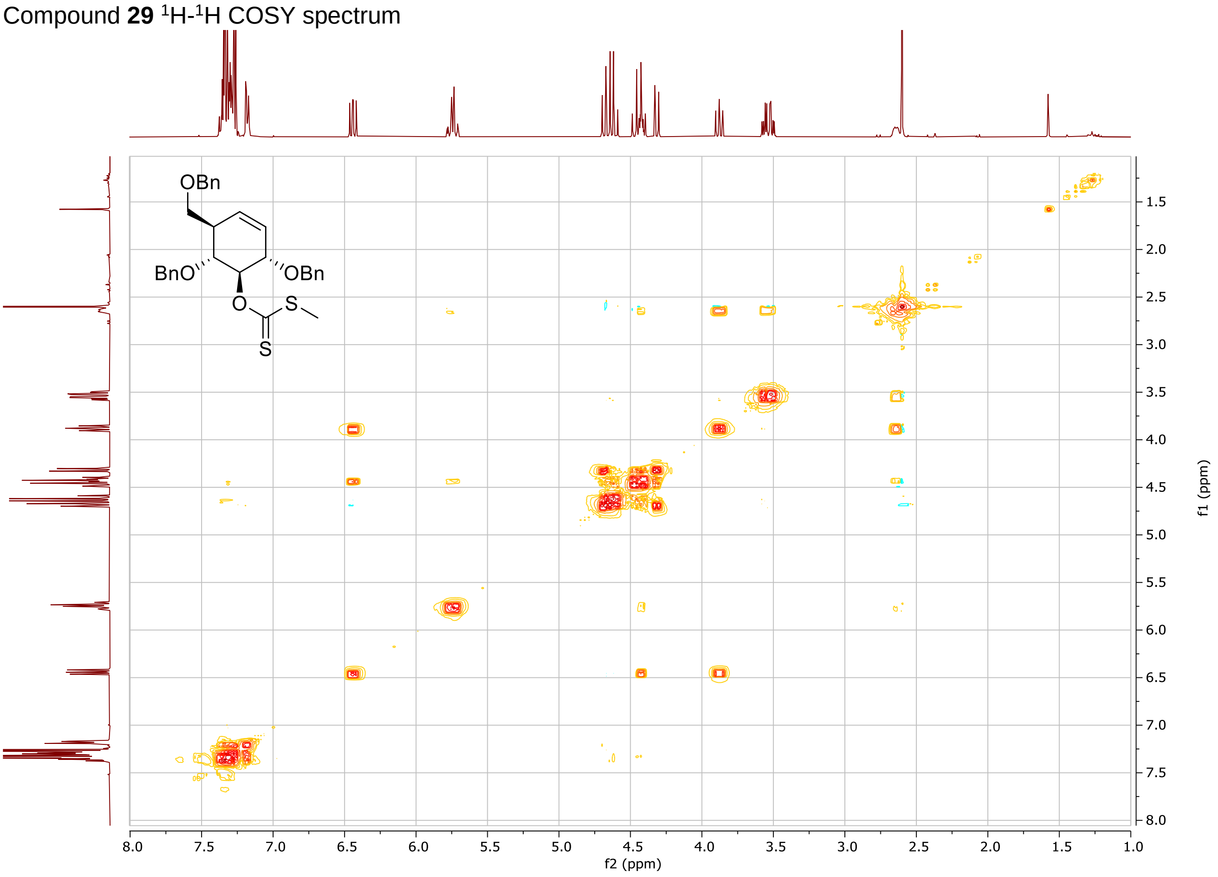

Compound **29** ^{1}H-^{13}C HSQC spectrum

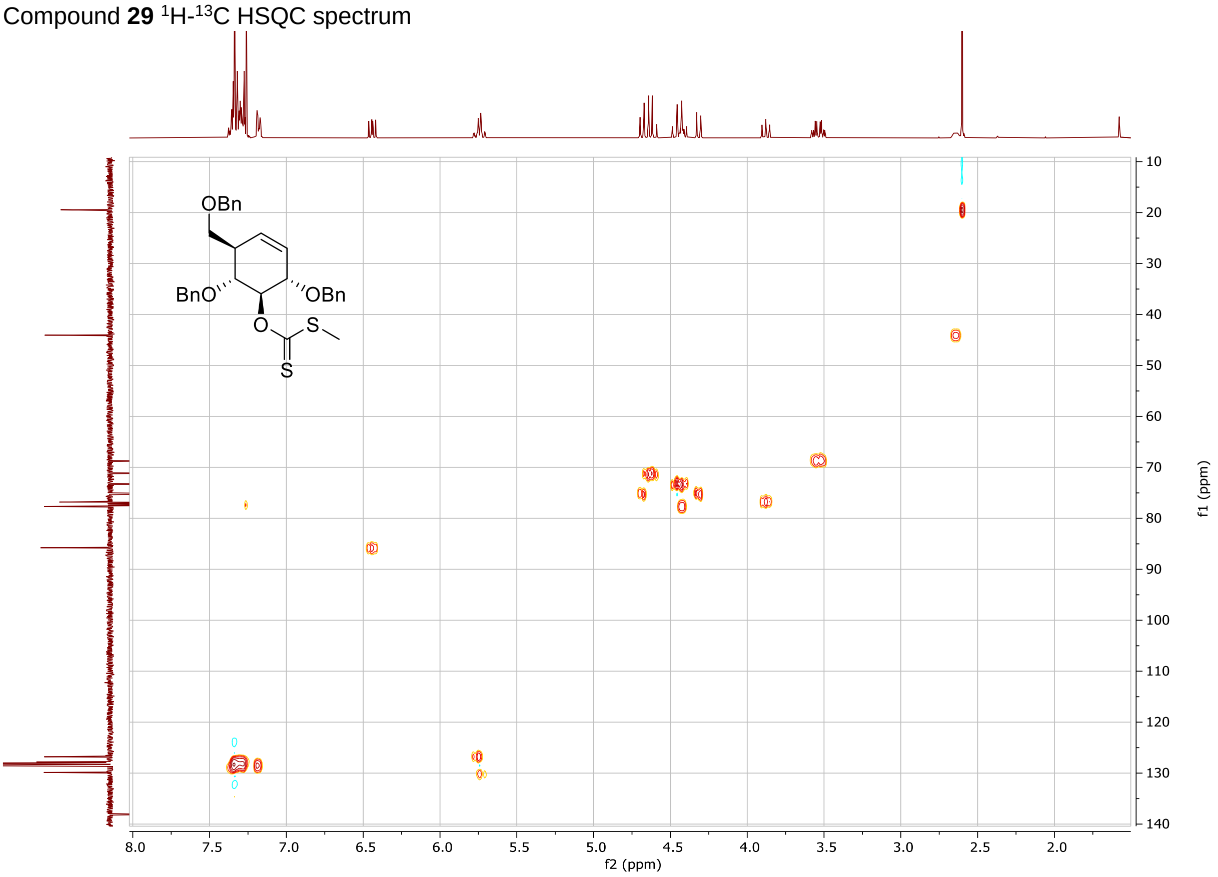

Compound **30** ^{1}H NMR spectrum

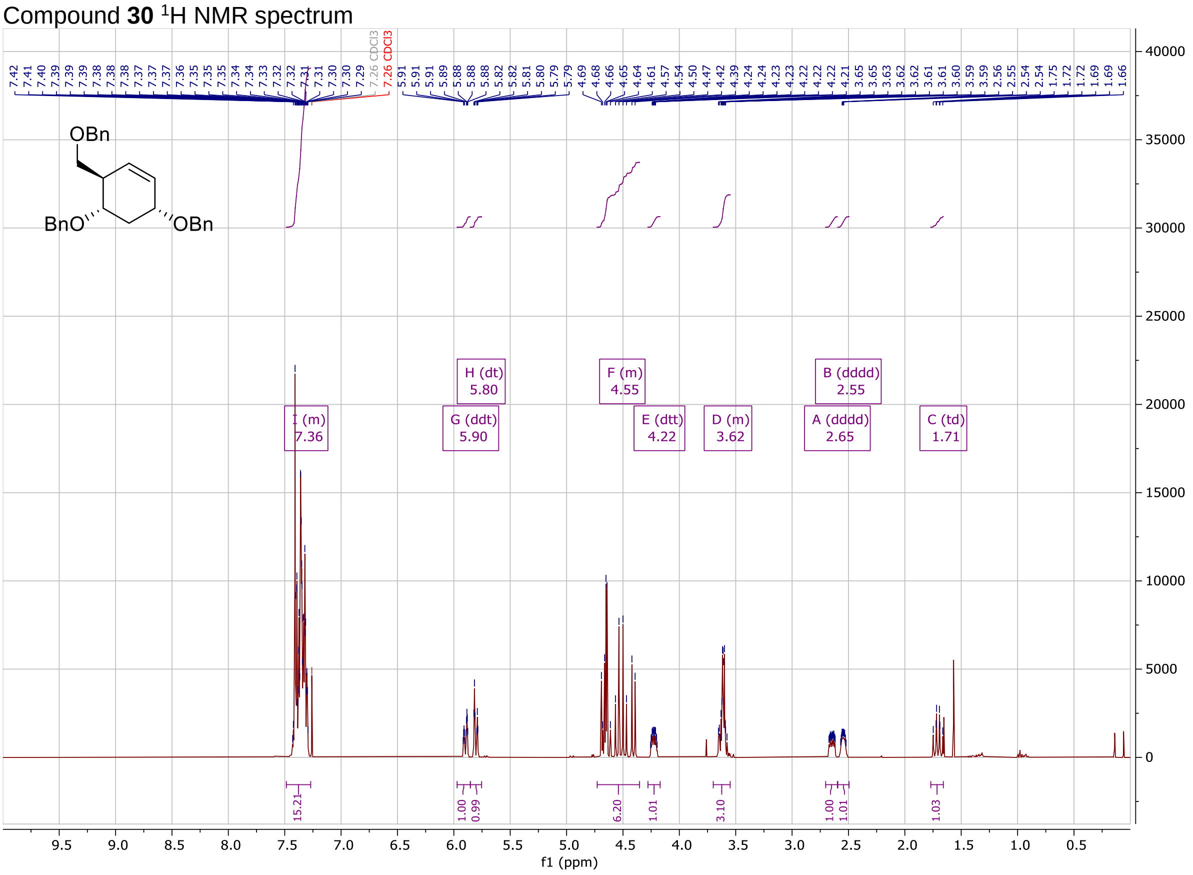

Compound **30** ^{13}C NMR APT spectrum

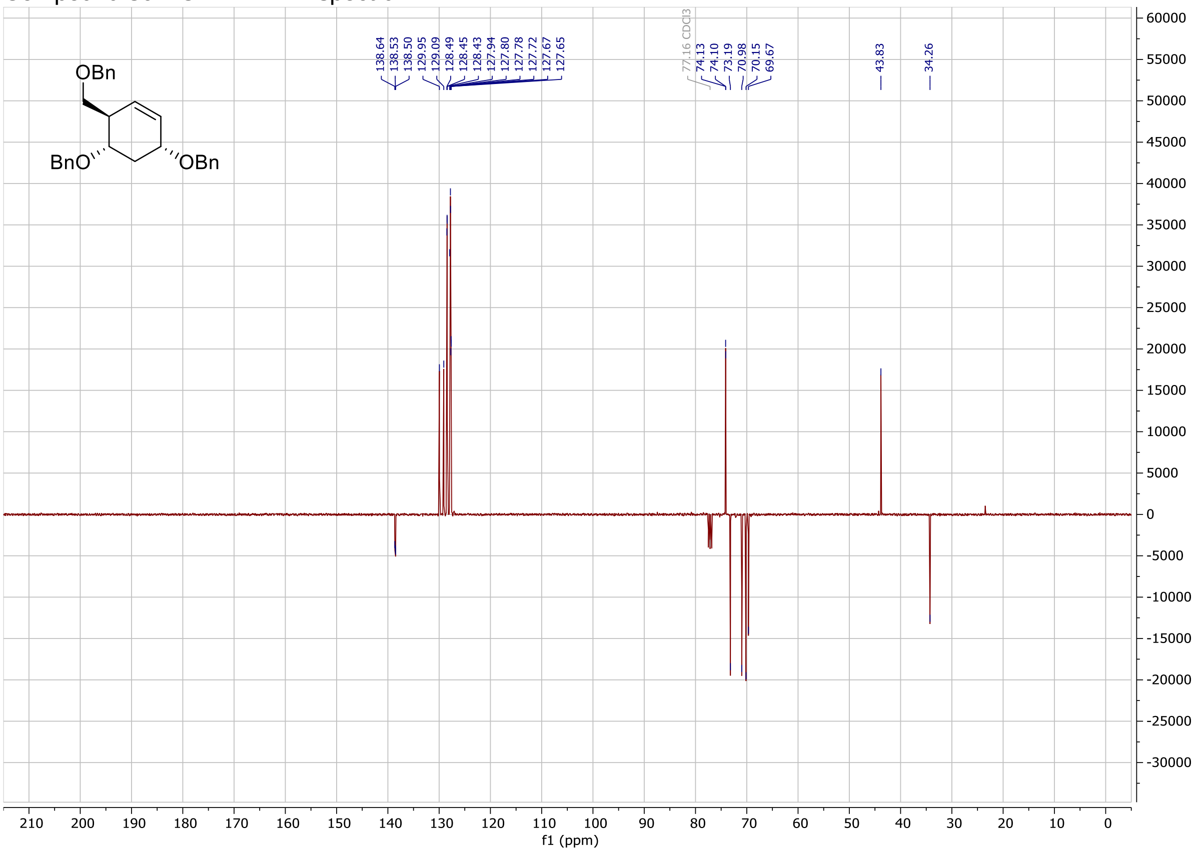

Compound **30** ^{1}H-^{1}H COSY spectrum

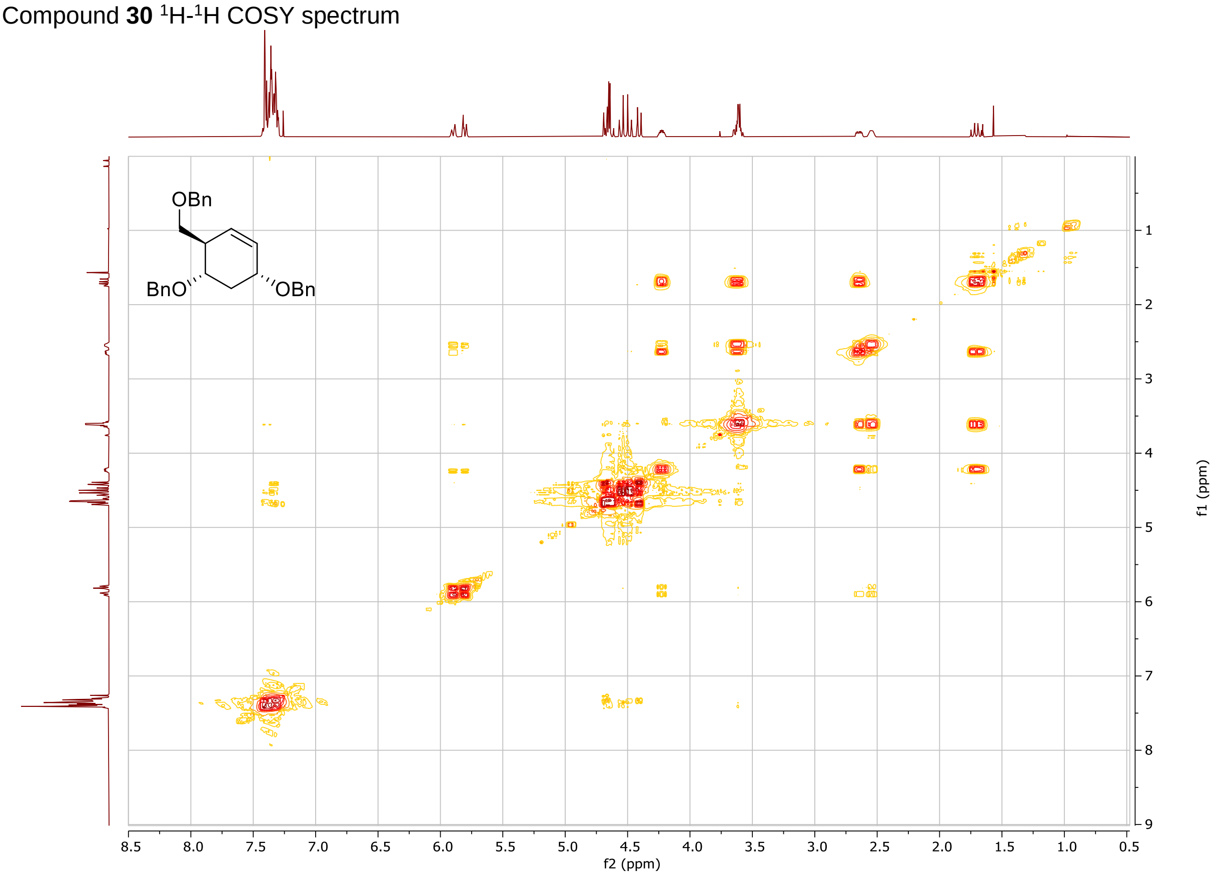

Compound **30** ^{1}H-^{13}C HSQC spectrum

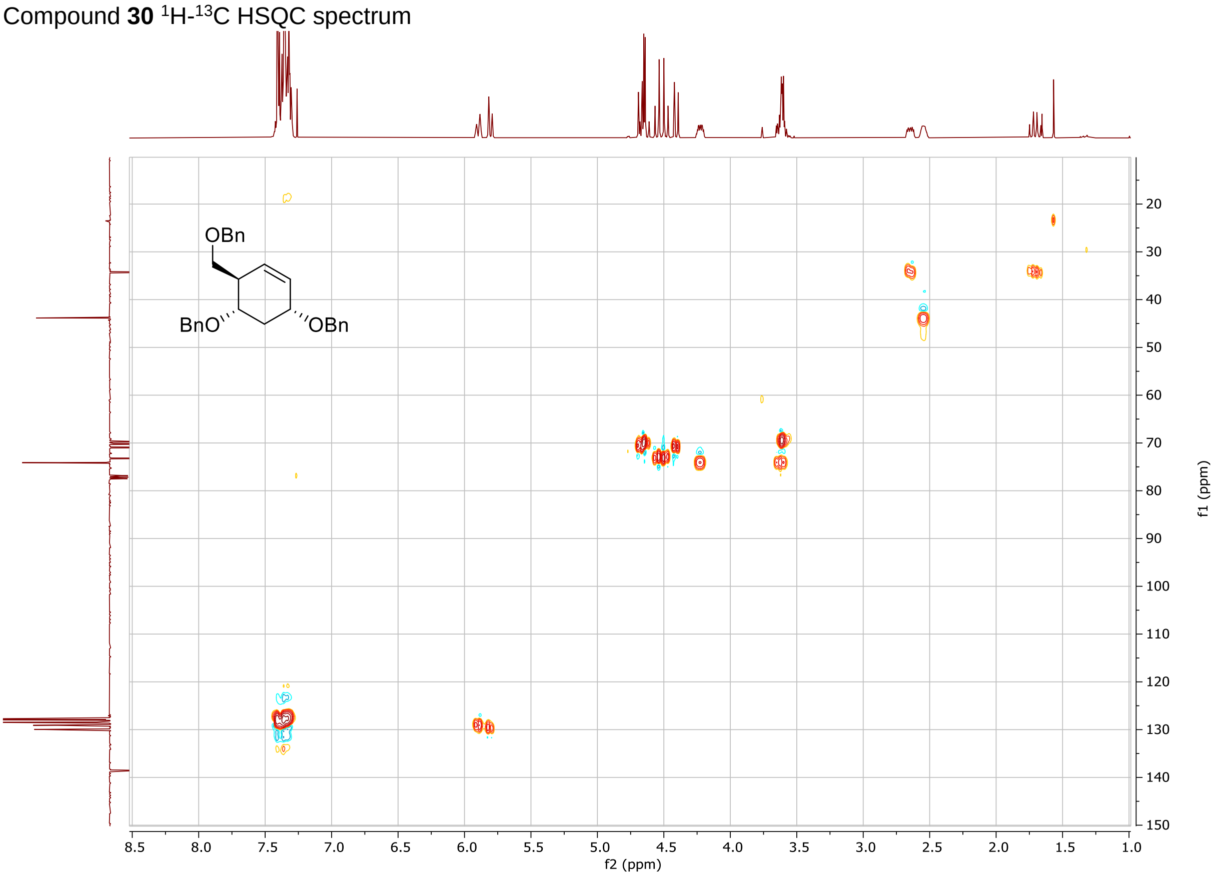

Compound **30** ^{1}H-^{13}C HMBC spectrum

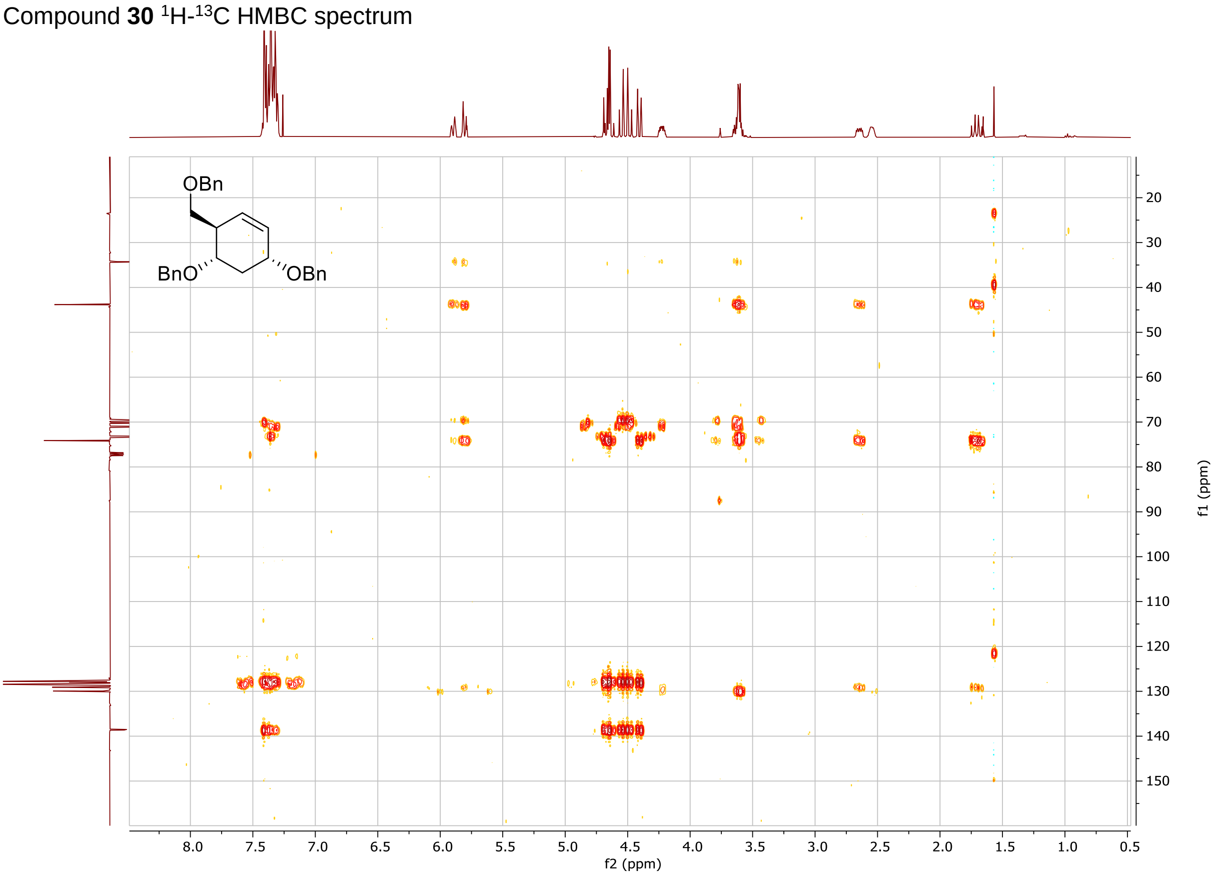

Compound **30** ^{1}H-^{1}H NOESY spectrum

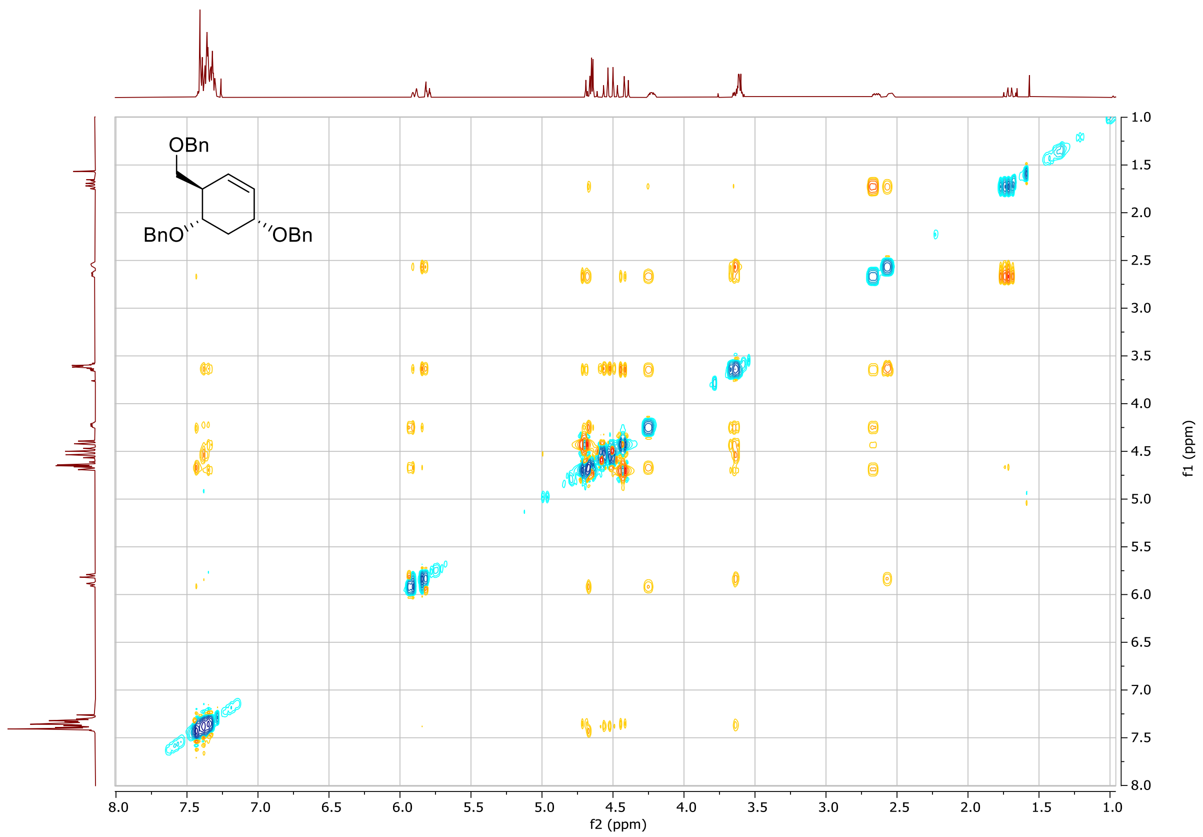

Compound **31** ^{1}H NMR spectrum

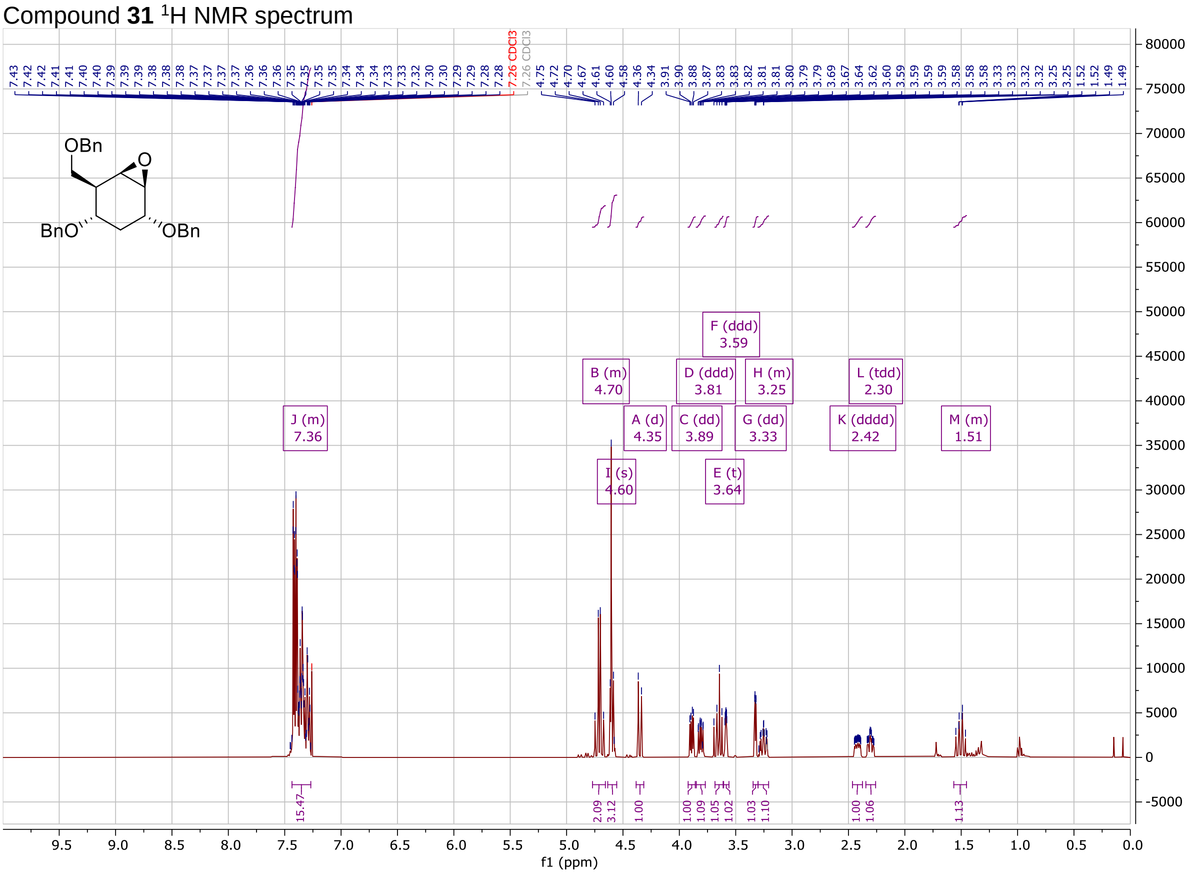

Compound **31** ^{13}C NMR APT spectrum

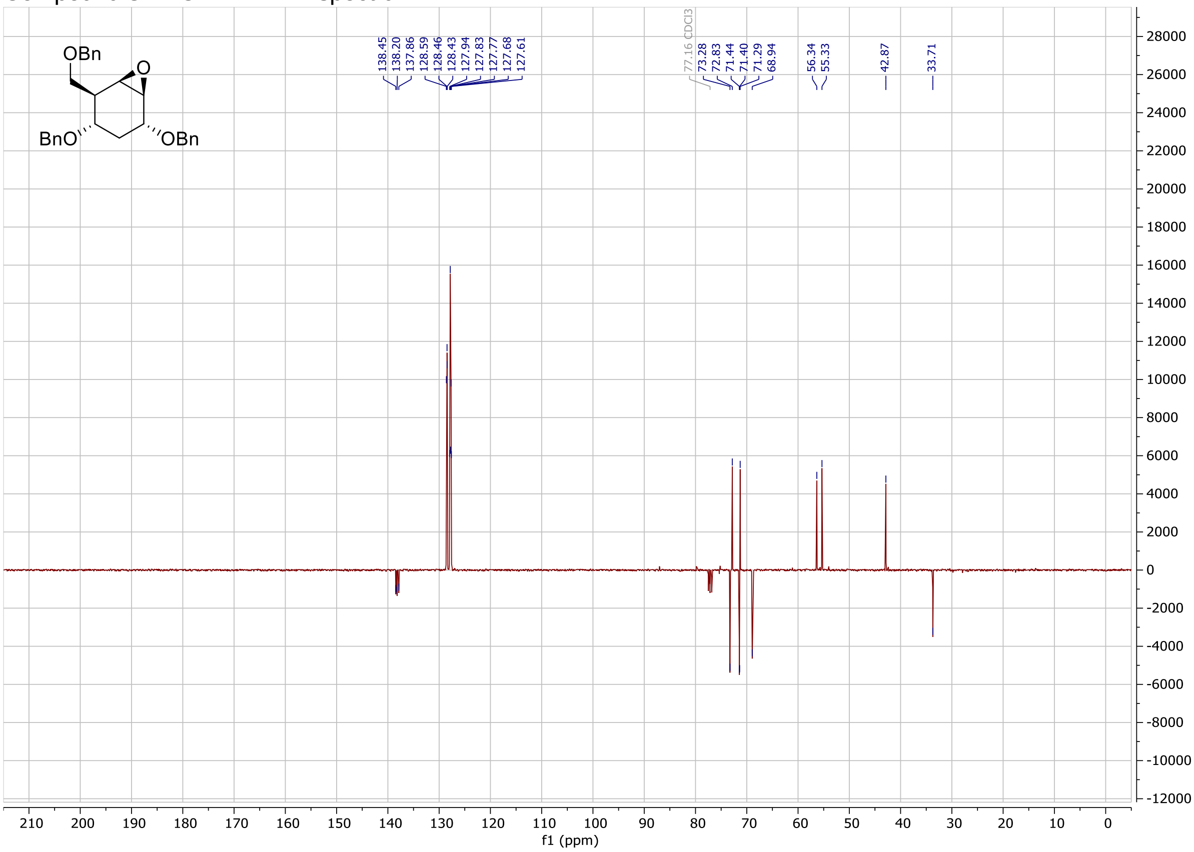

Compound **31** ^{1}H-^{1}H COSY spectrum

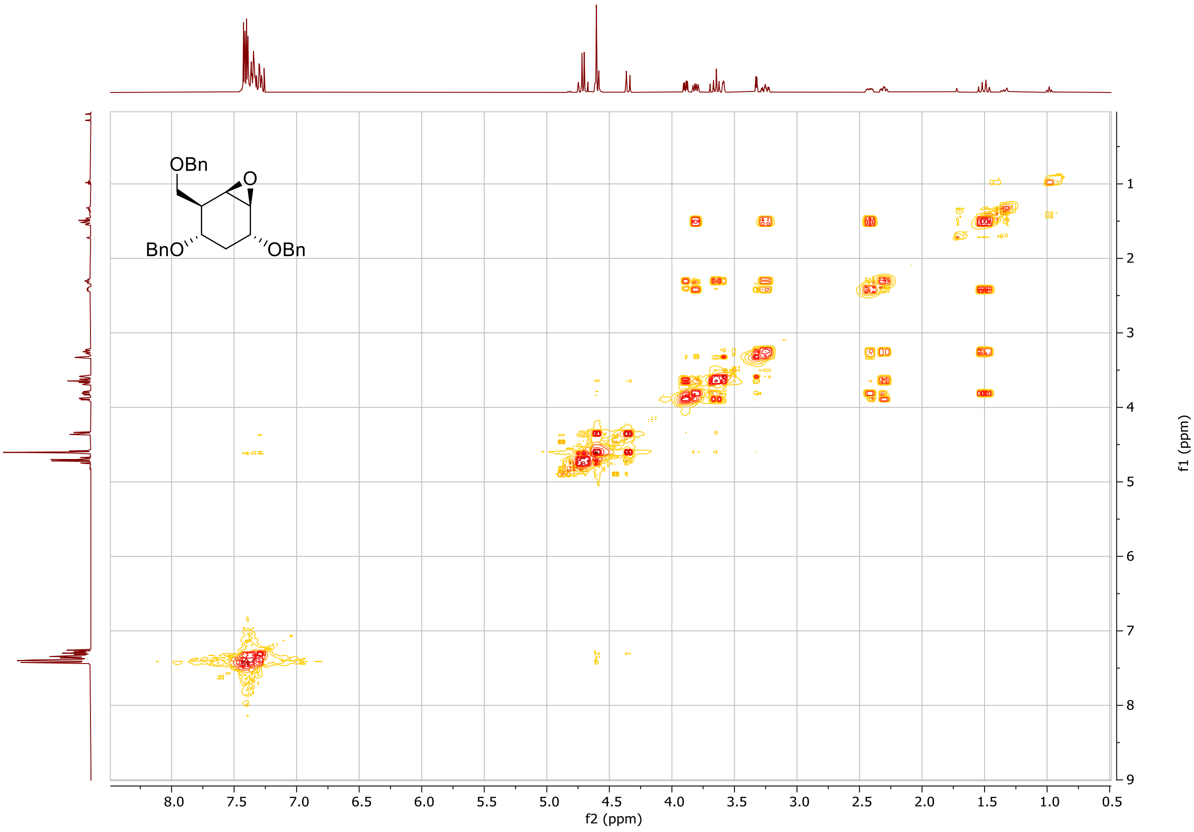

Compound **31** ^{1}H-^{13}C HSQC spectrum

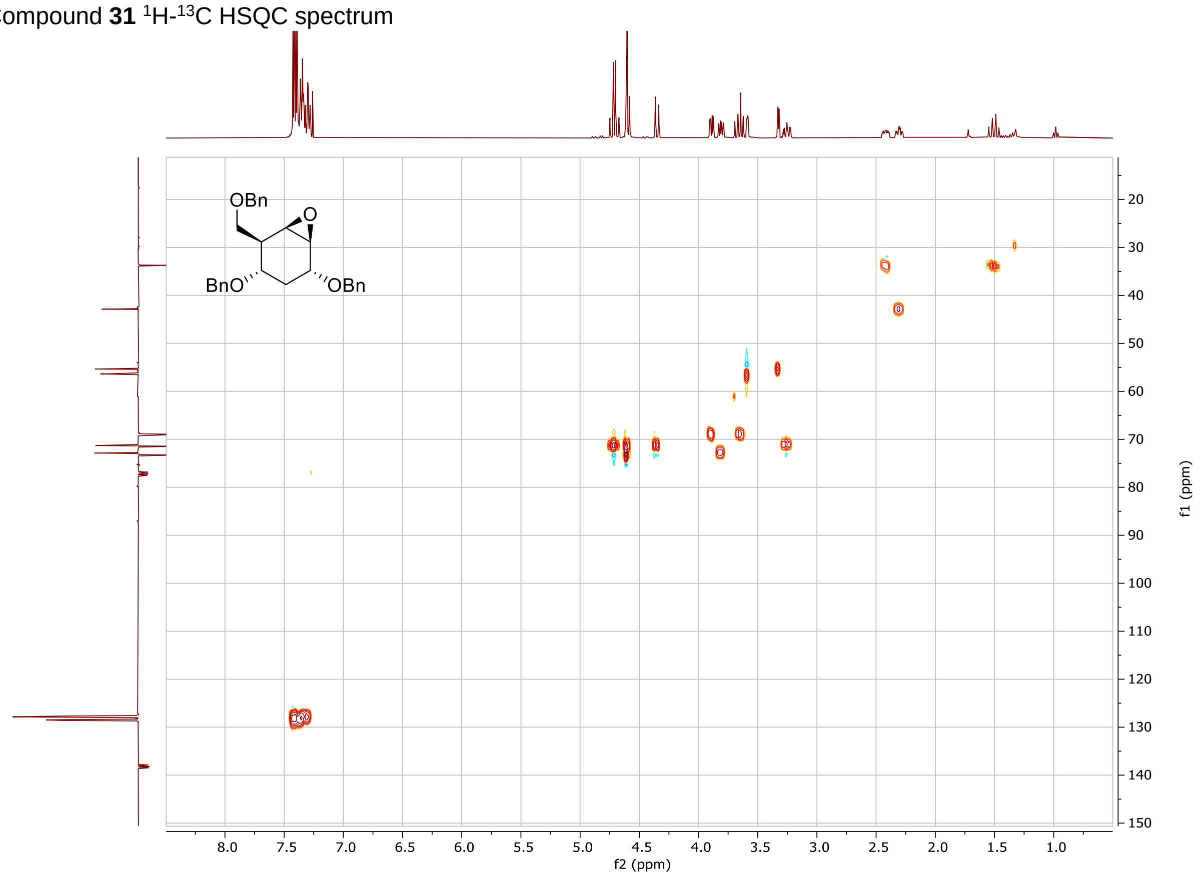

Compound **31** ^{1}H-^{1}H NOESY spectrum

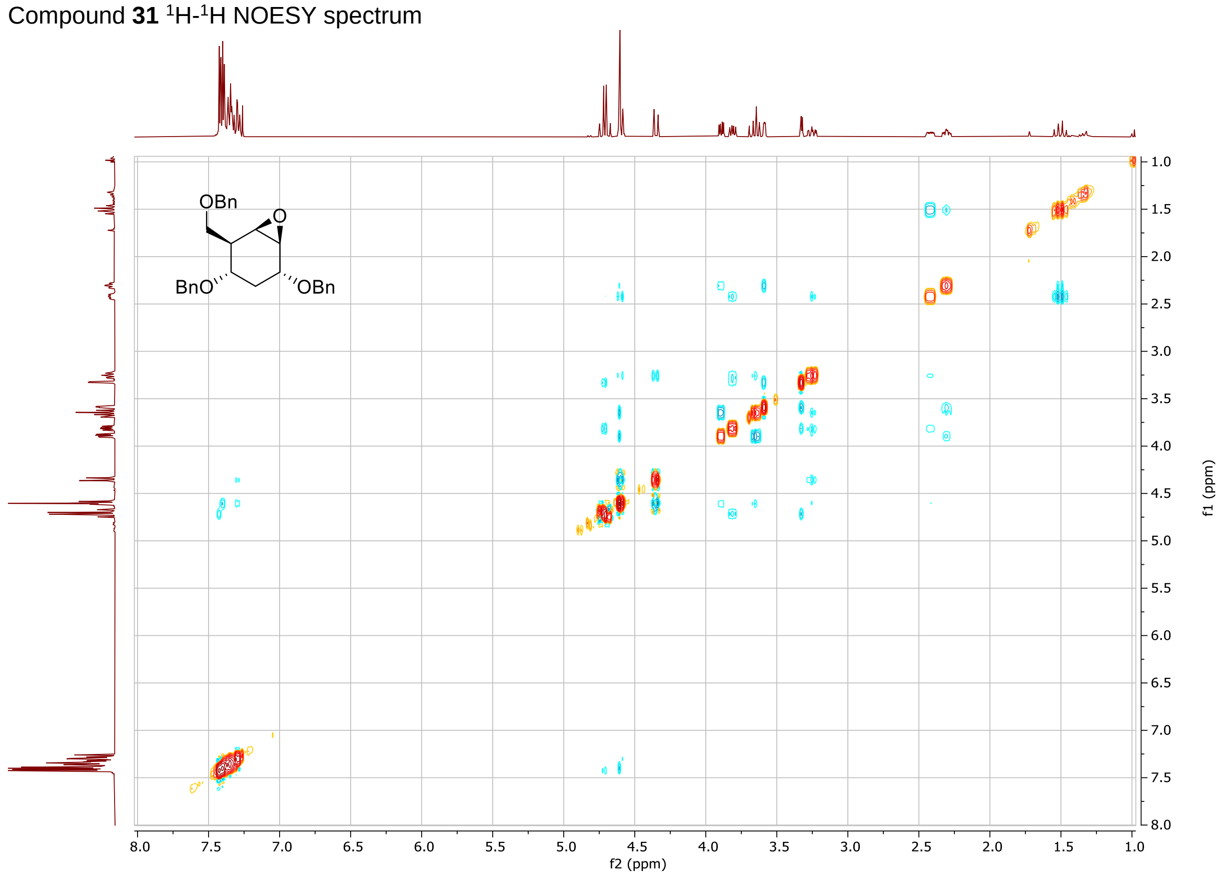

Compound **31** ^{1}H-^{13}C HMBC spectrum

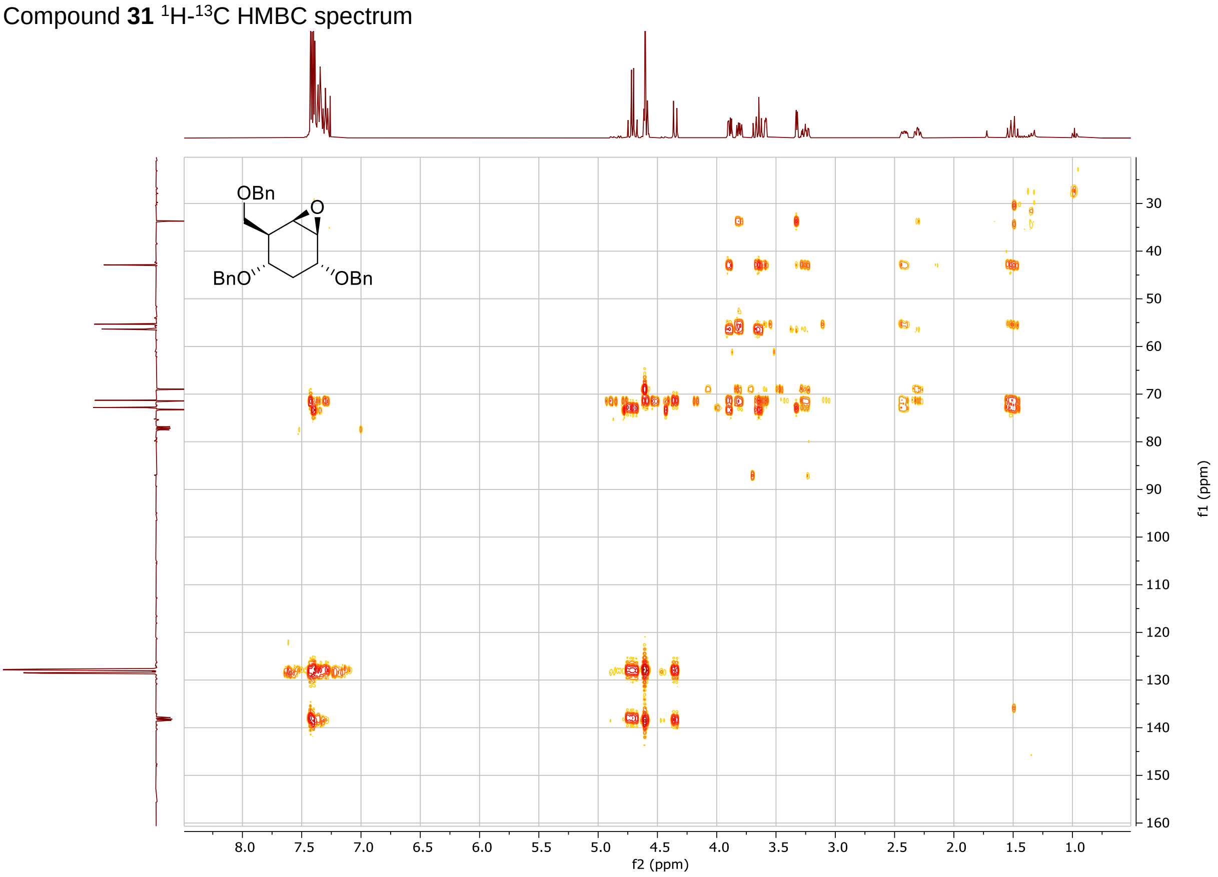

Compound **32** ^{1}H NMR spectrum

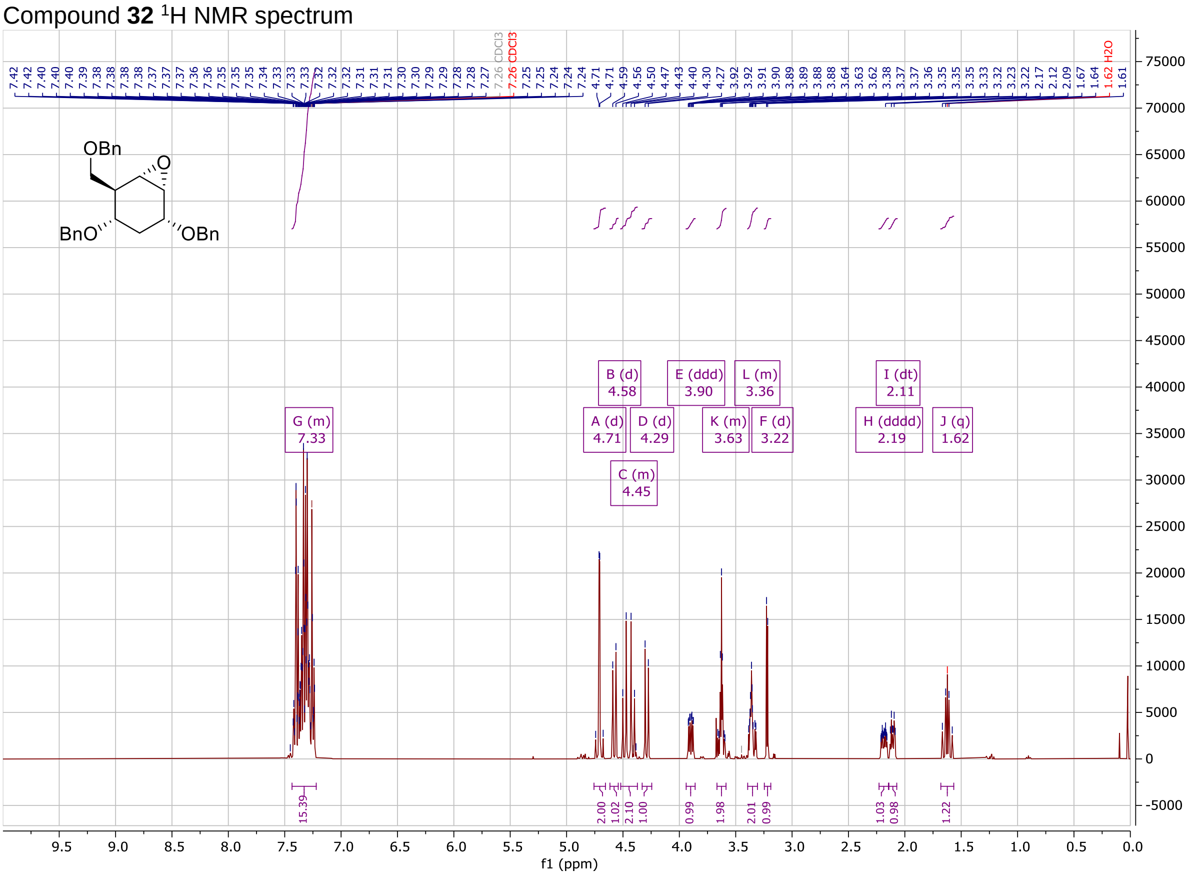

Compound **32** ^{13}C NMR APT spectrum

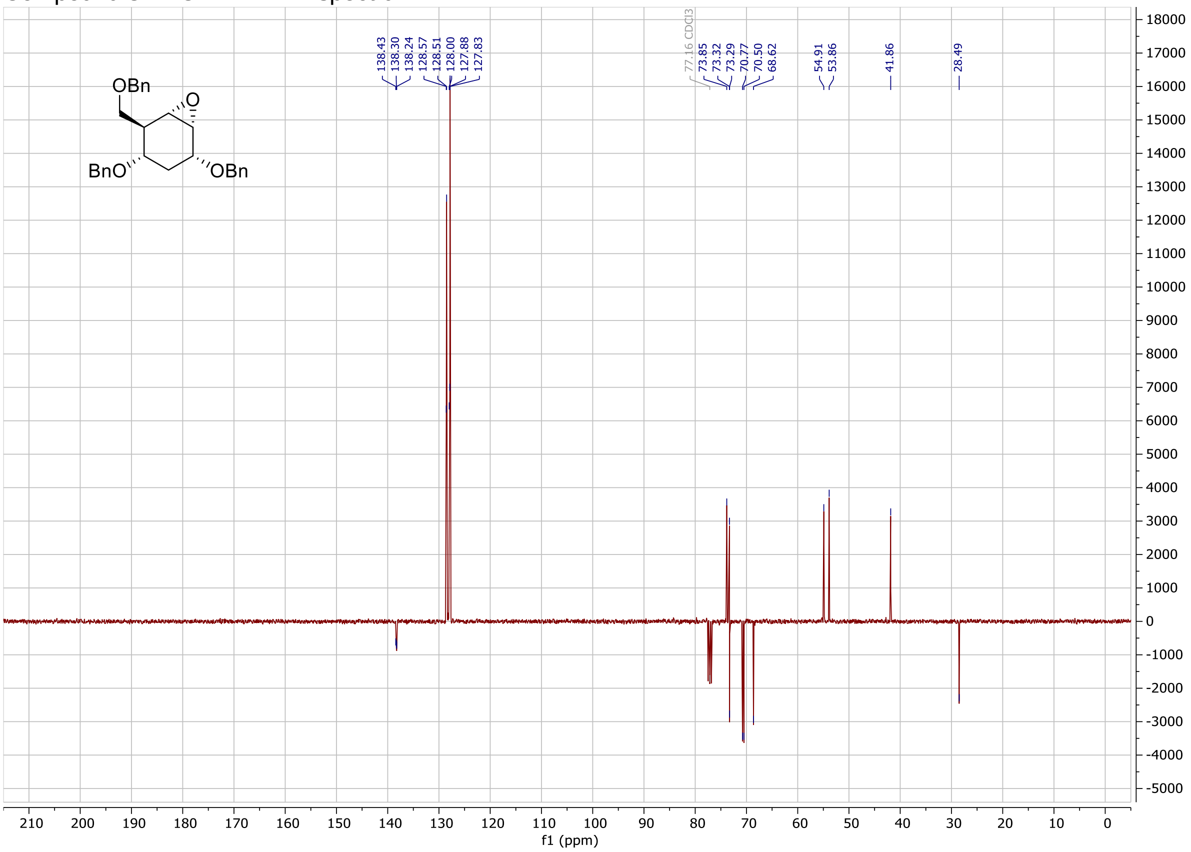

Compound **33** ^{1}H NMR spectrum

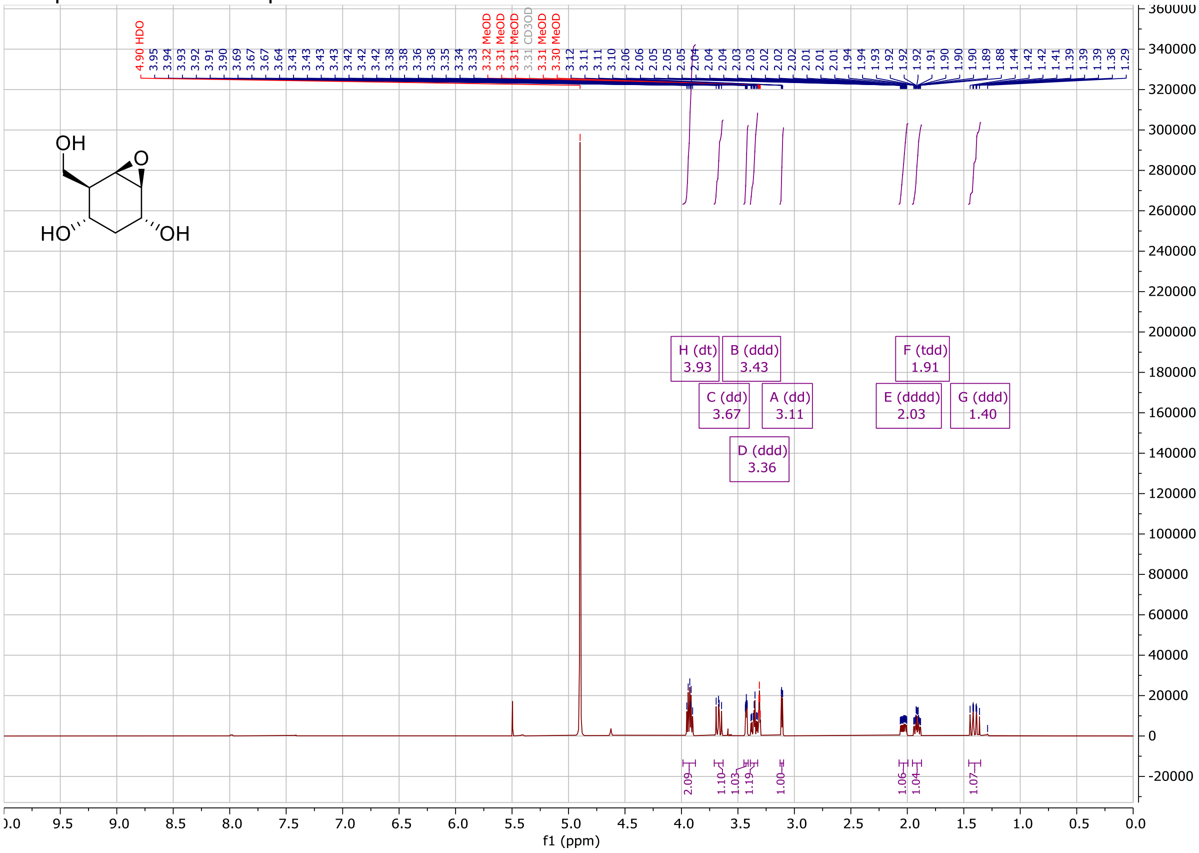

Compound **33** ^{13}C NMR APT spectrum

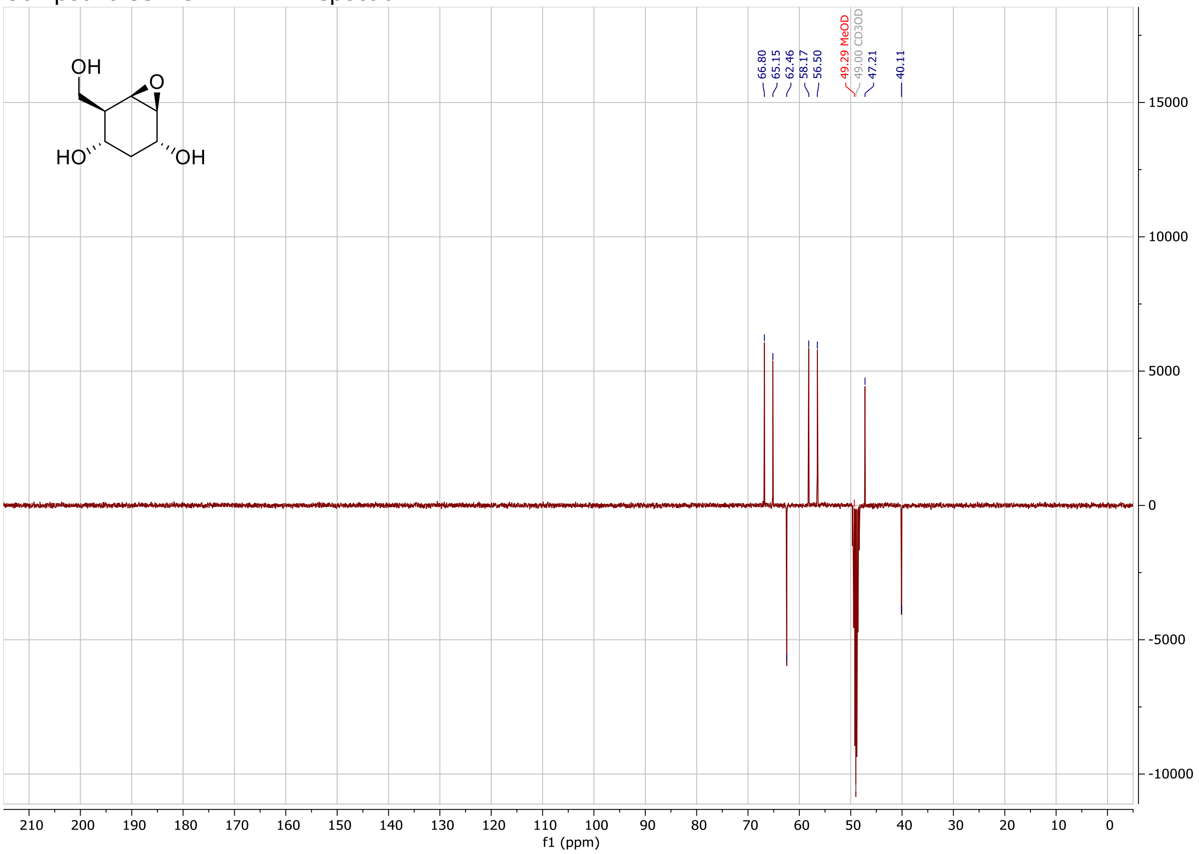

Compound **33** ^{1}H-^{1}H COSY spectrum

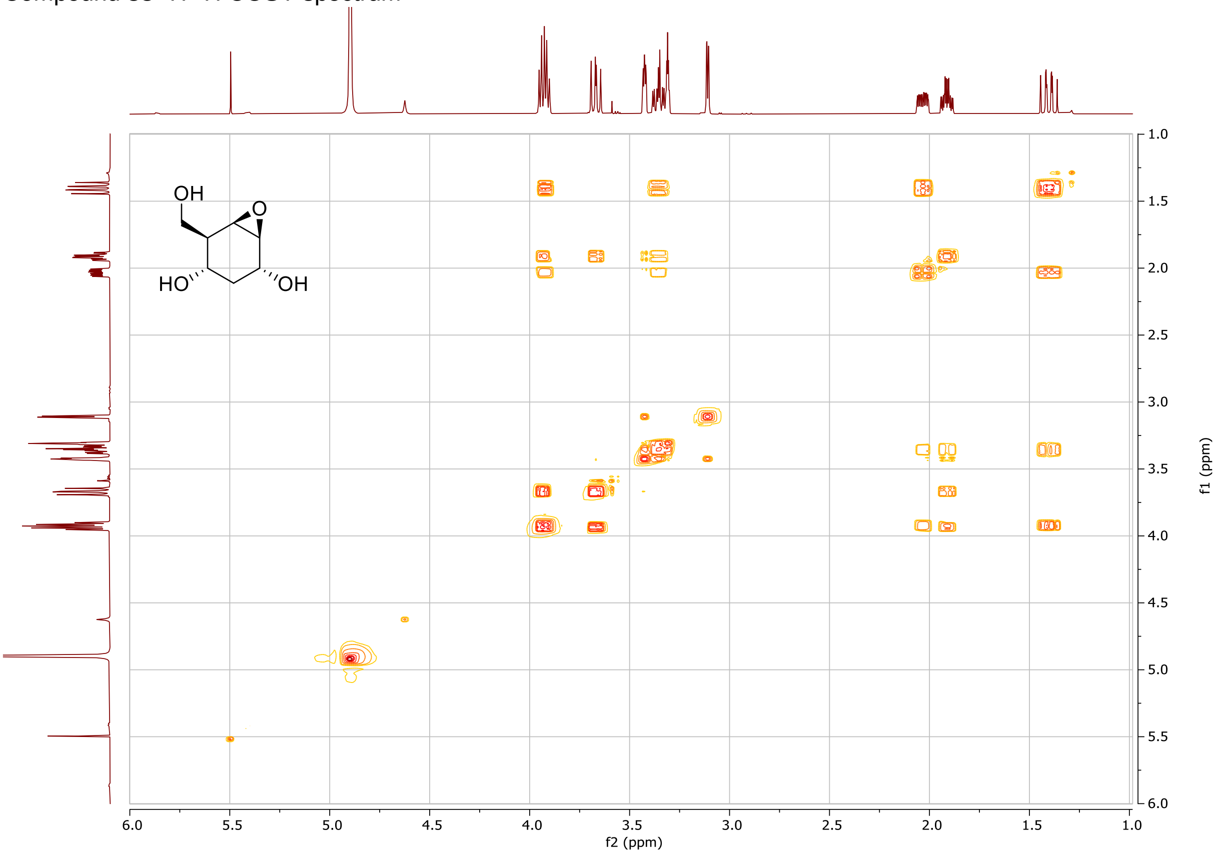

Compound **33** ^{1}H-^{13}C HSQC spectrum

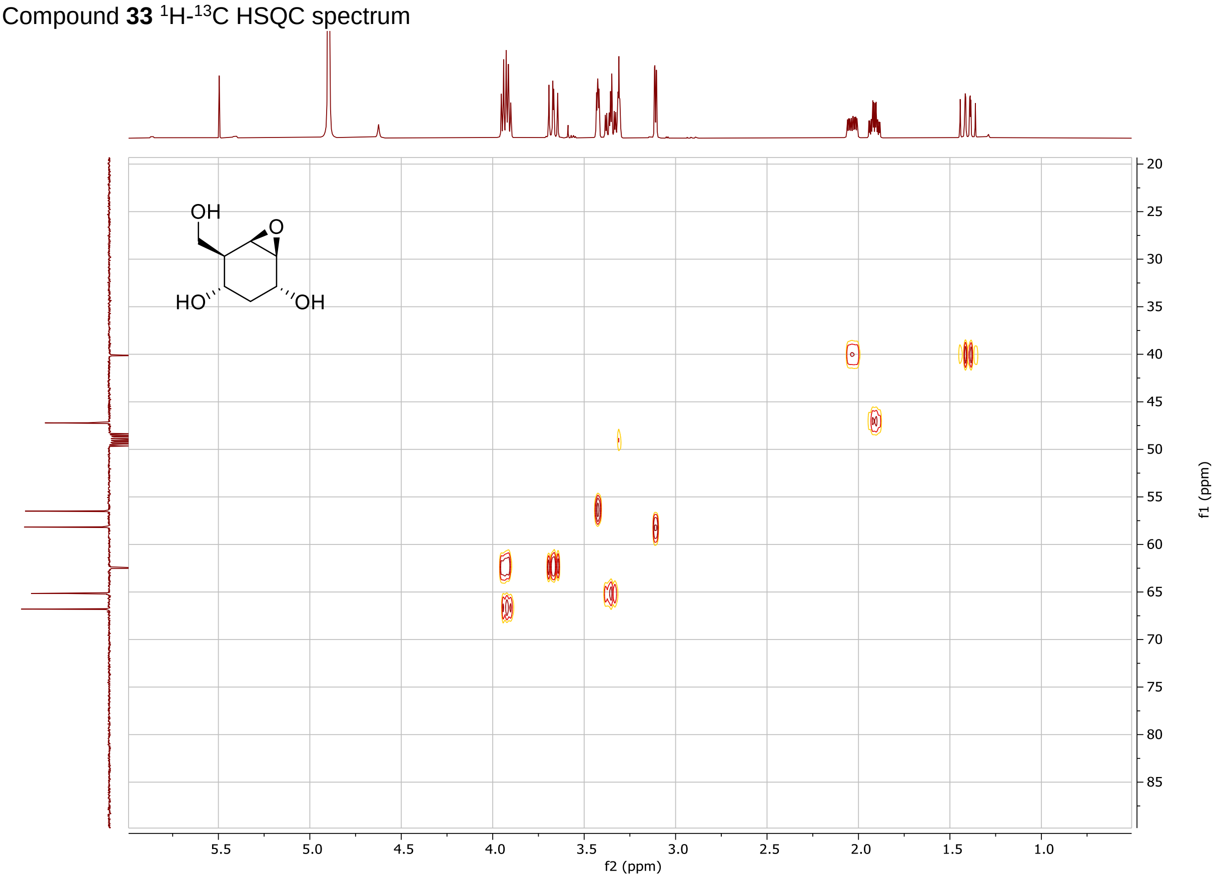

Compound **34** ^{1}H NMR spectrum

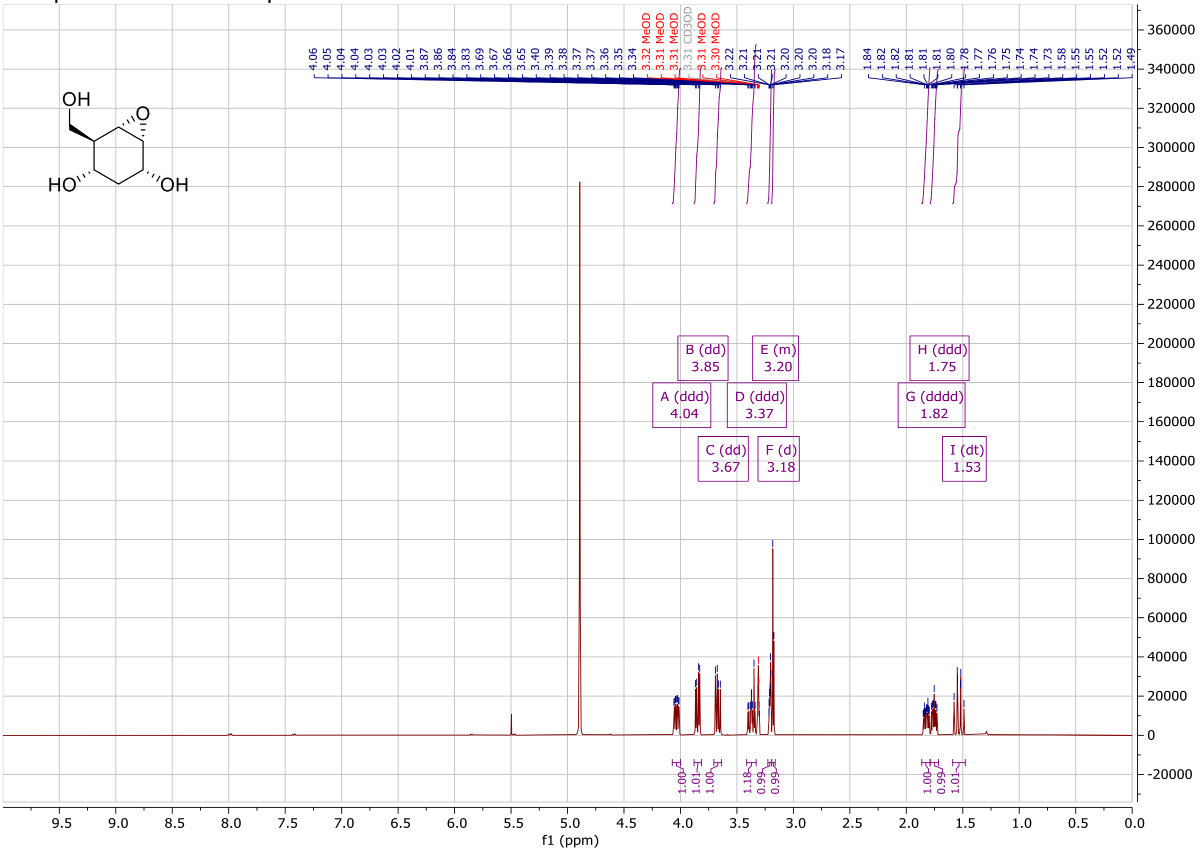

Compound **34** ^{13}C NMR APT spectrum

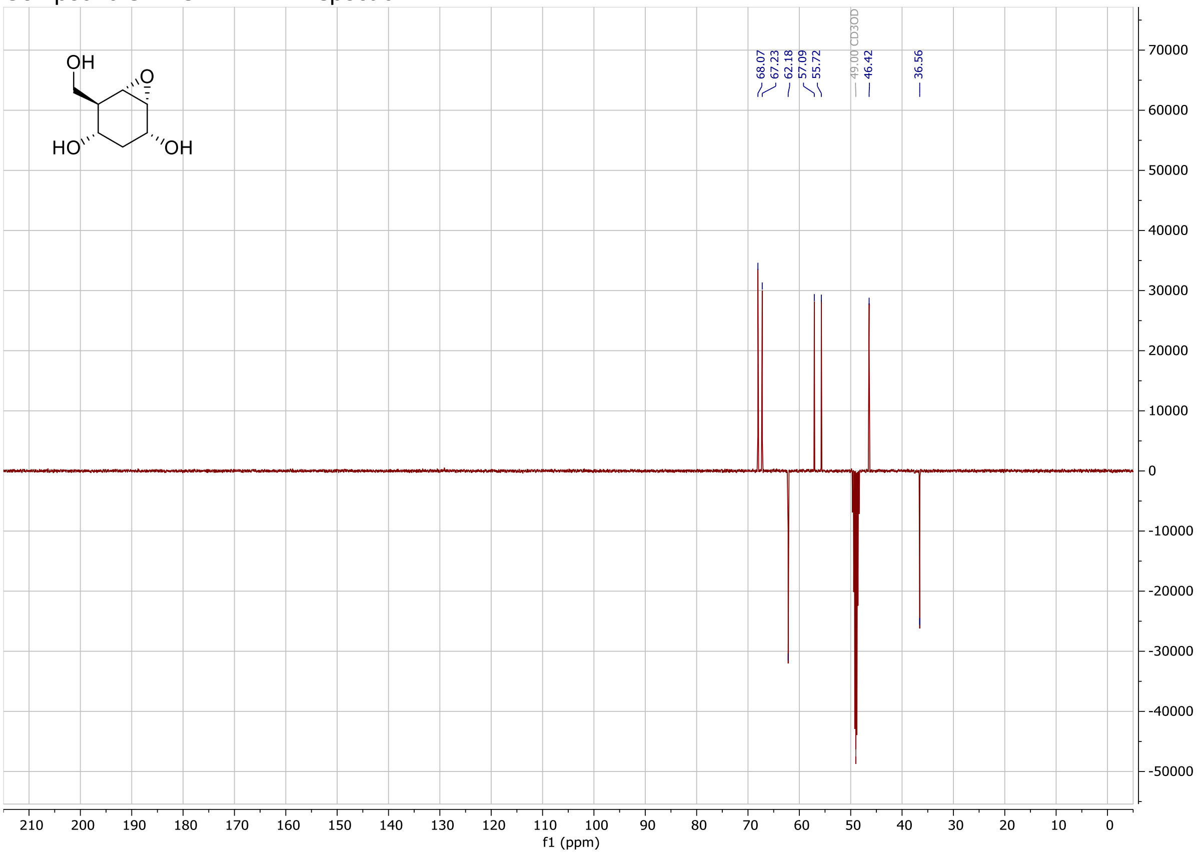

Compound **34** ^{1}H-^{1}H COSY spectrum

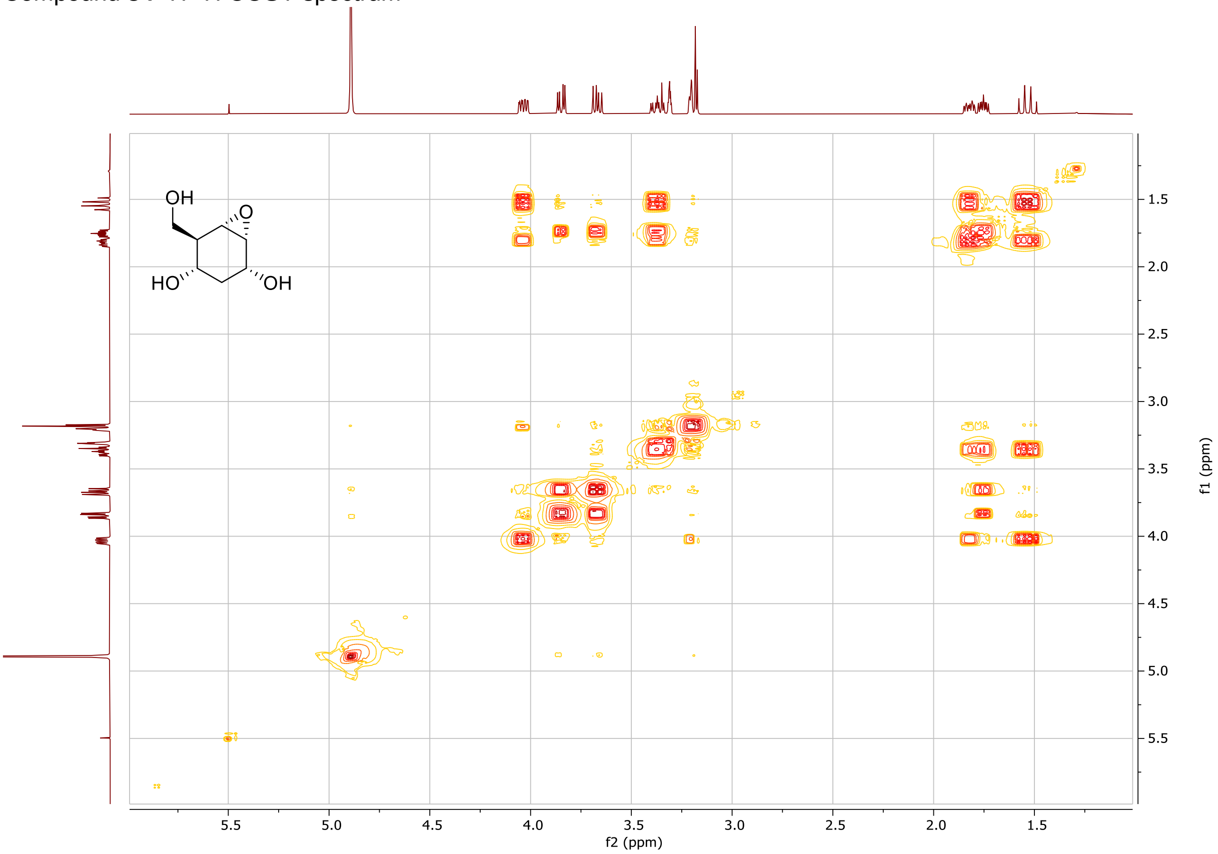

Compound **34** ^{1}H-^{13}C HSQC spectrum

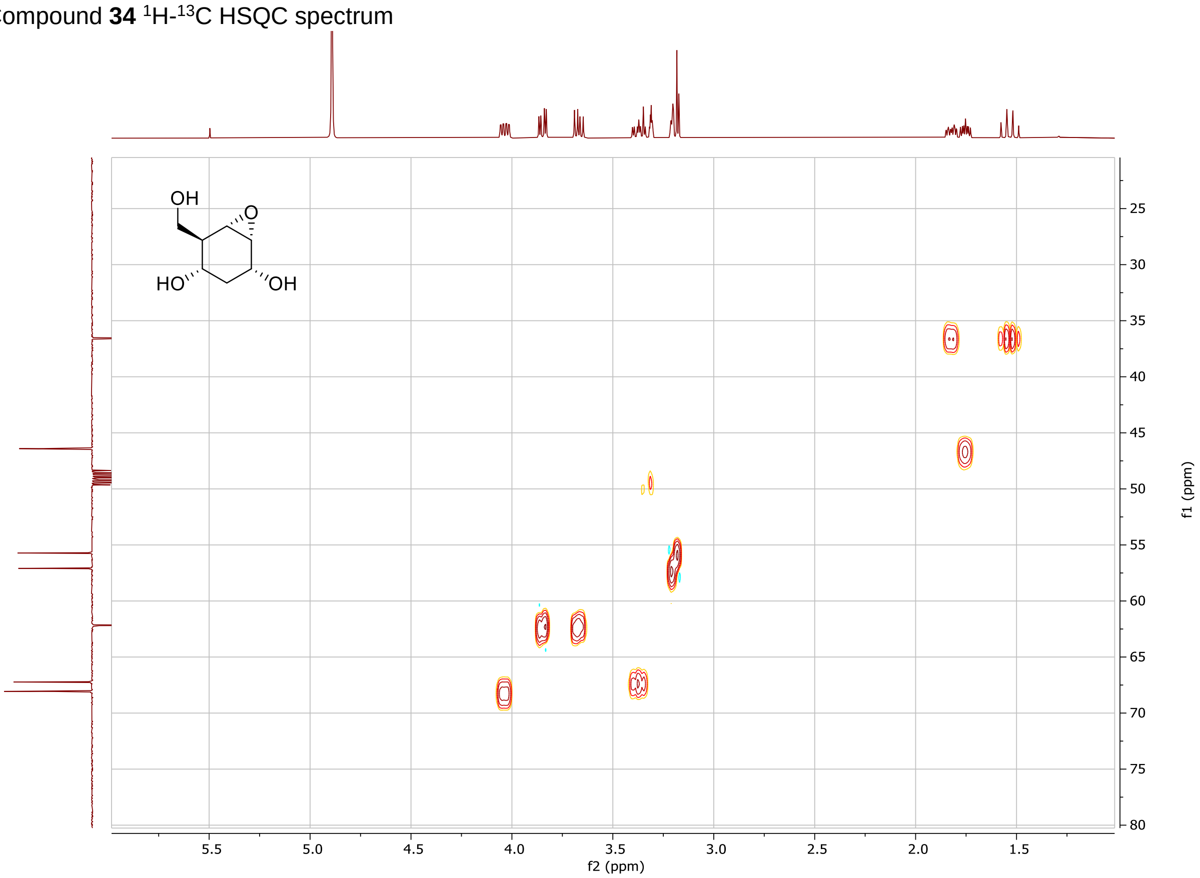

Compound **35** ^{1}H NMR spectrum

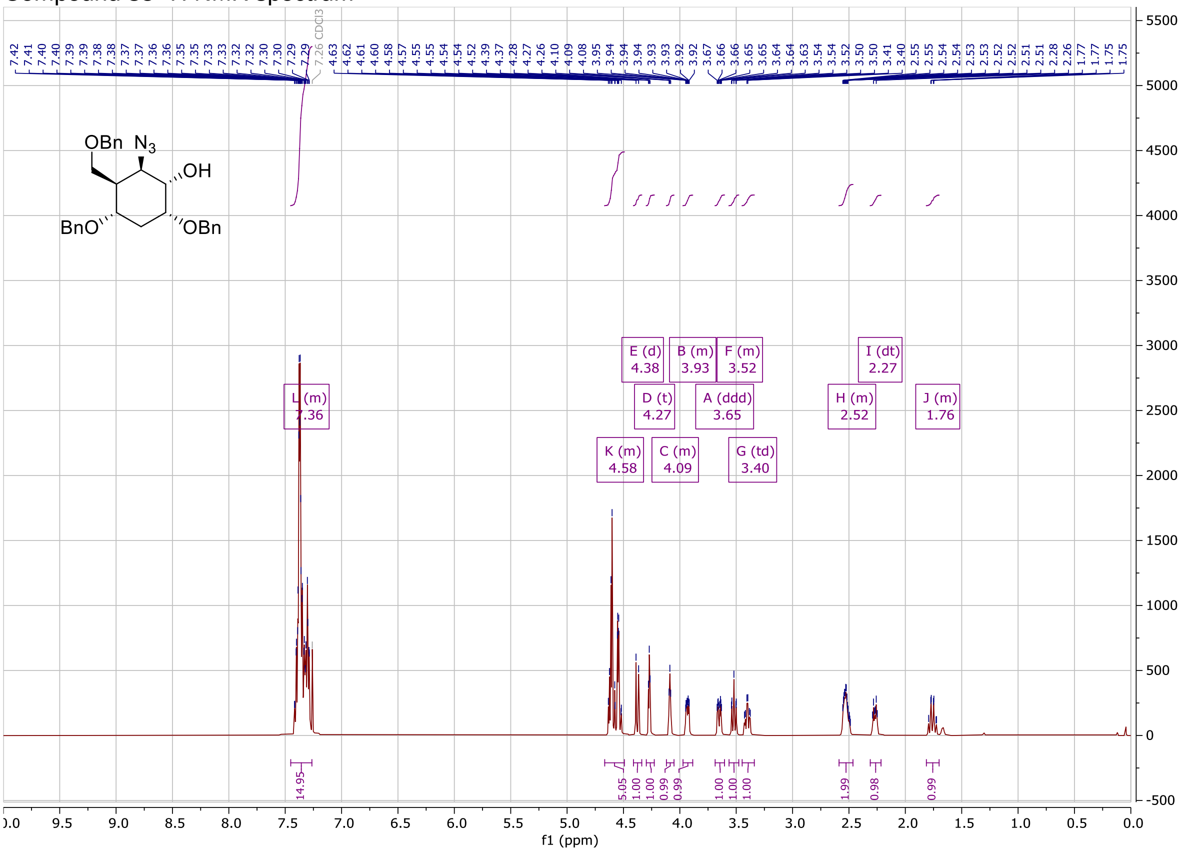

Compound **35** ^{13}C NMR APT spectrum

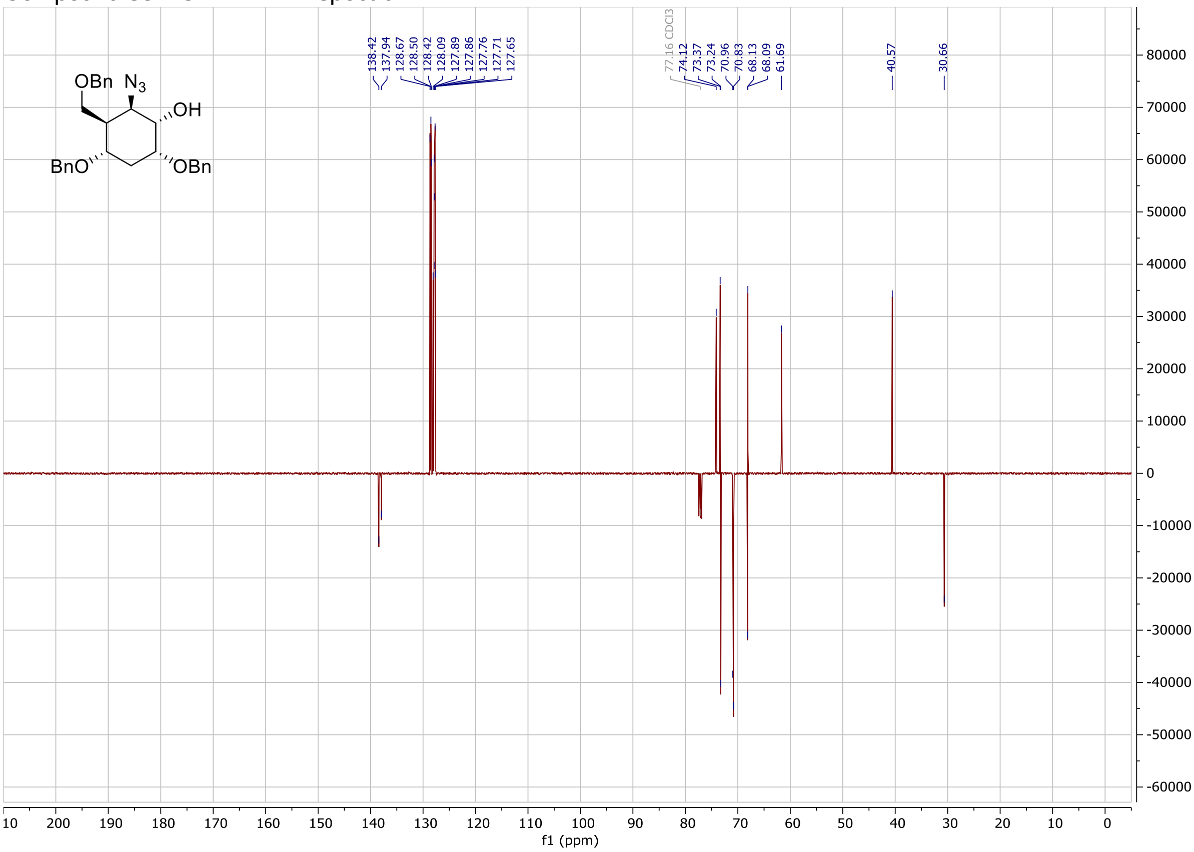

Compound **35** ^{1}H-^{1}H COSY spectrum

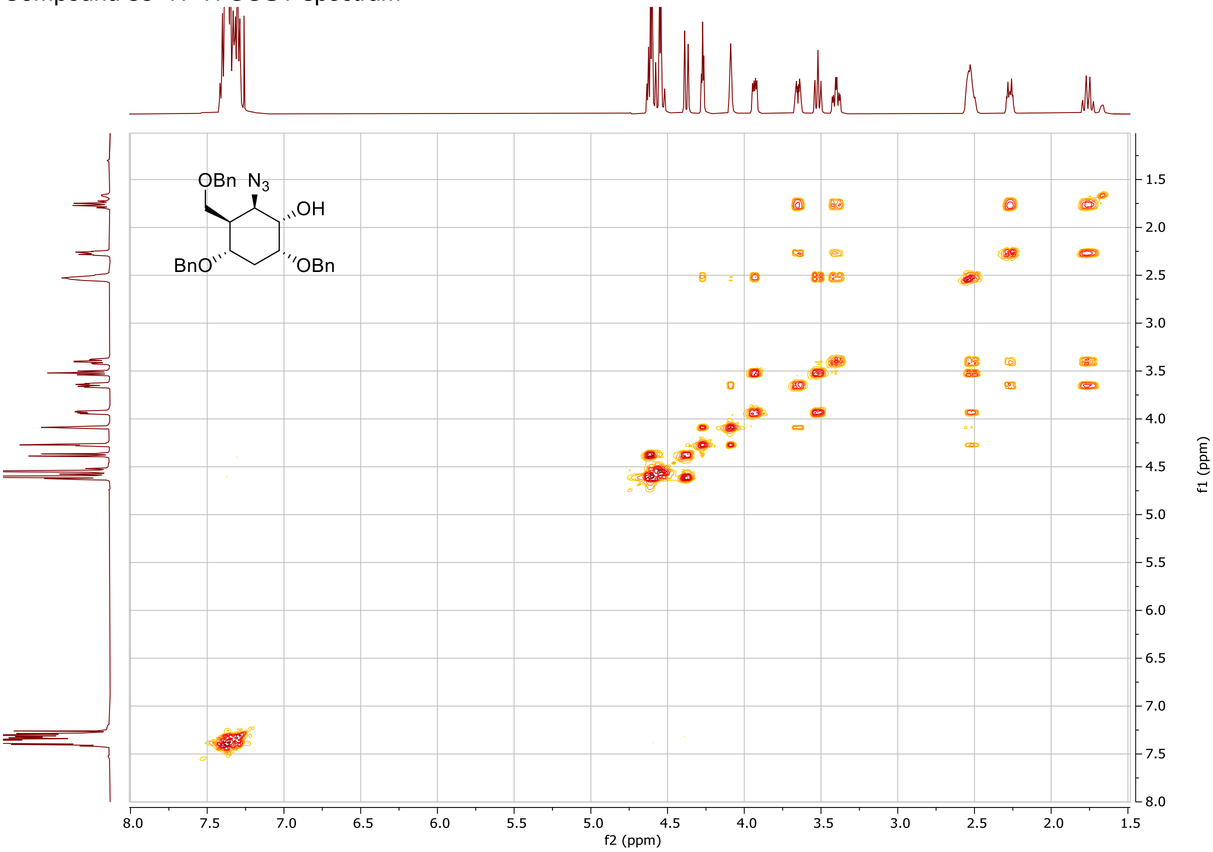

Compound **35** ^{1}H-^{13}C HSQC spectrum

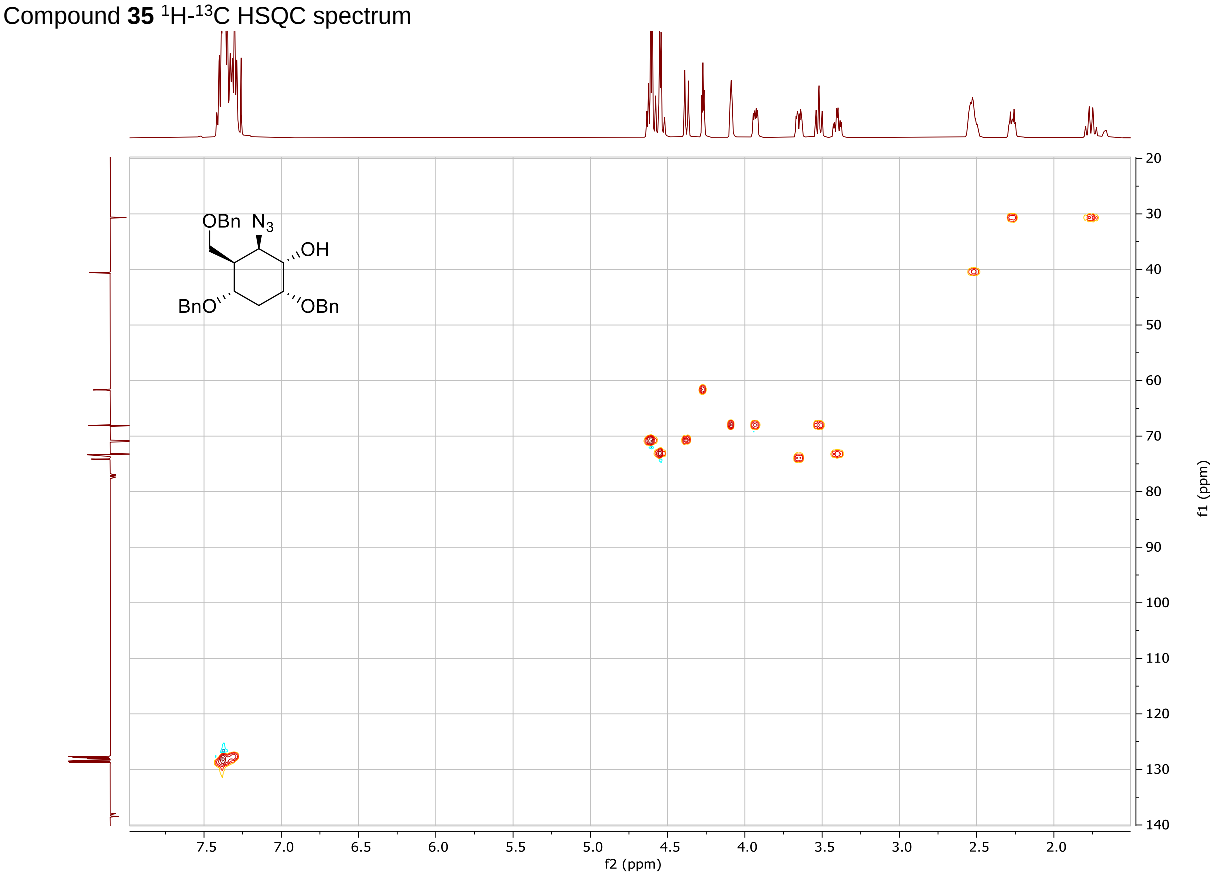

Compound **35** ^{1}H-^{1}H NOESY spectrum

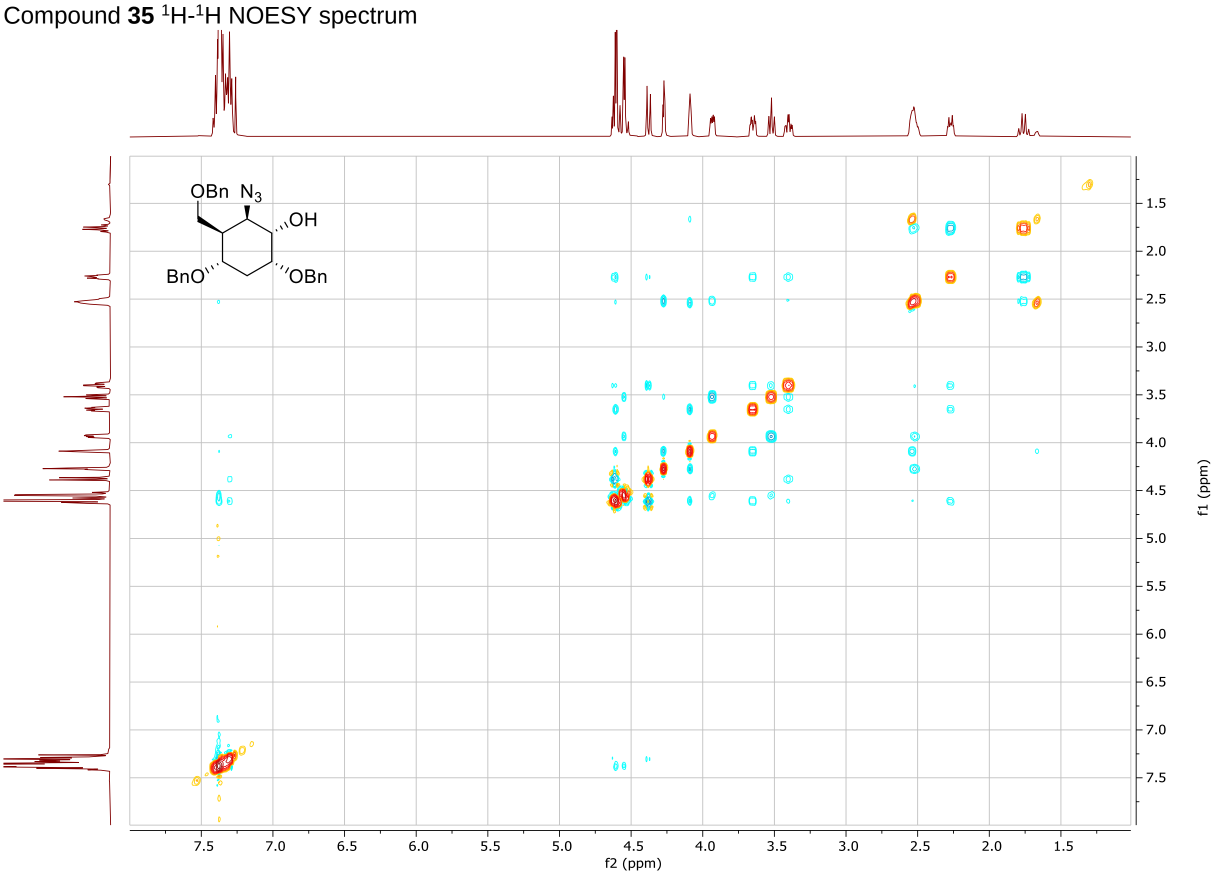

Compound **35** ^{1}H-^{13}C HMBC spectrum

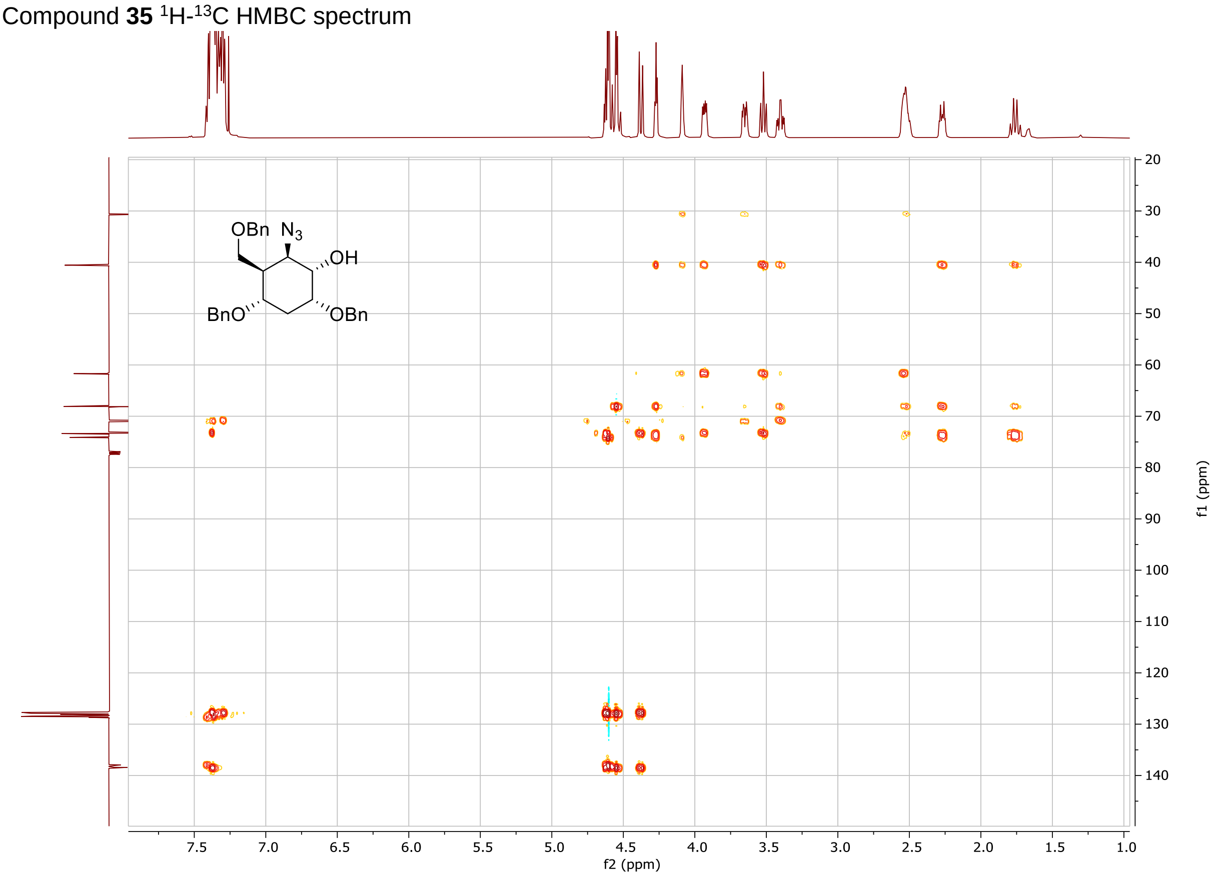

Compound **36** ^{1}H NMR spectrum

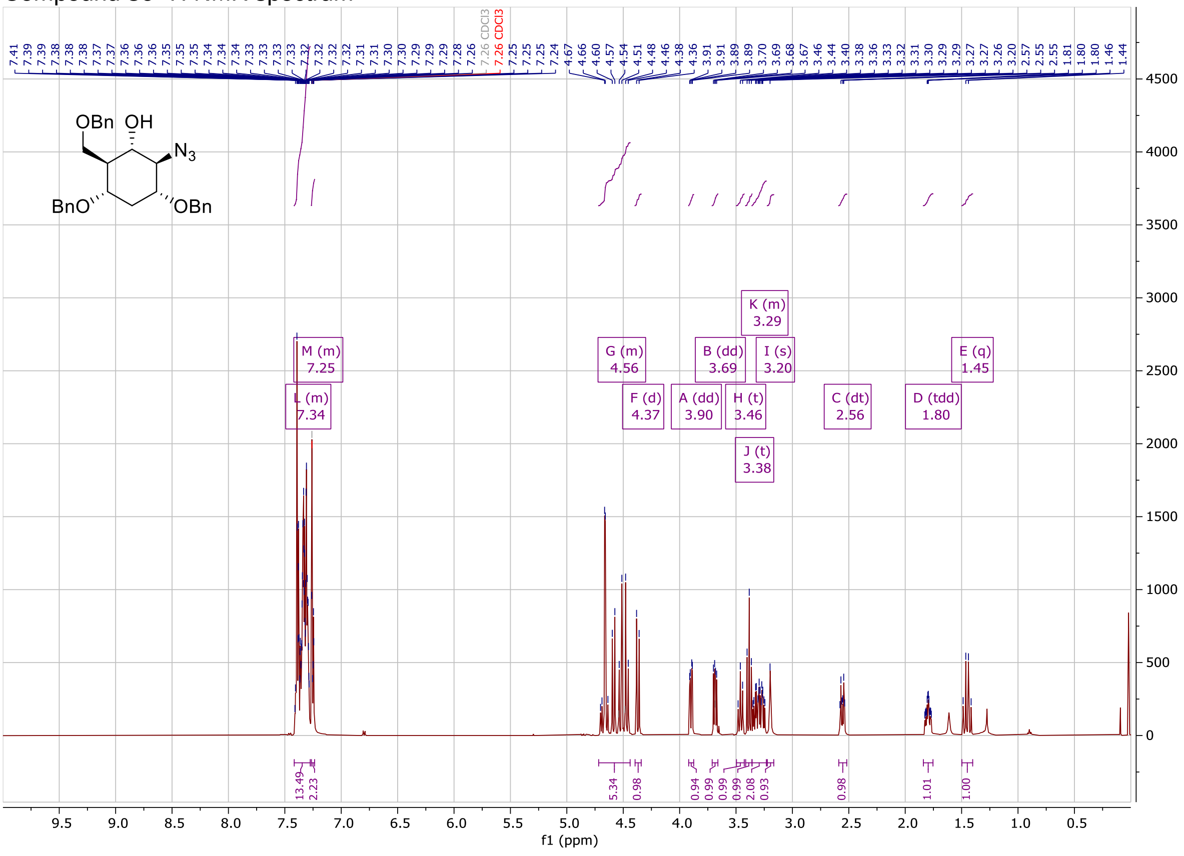

Compound **36** ^{13}C NMR APT spectrum

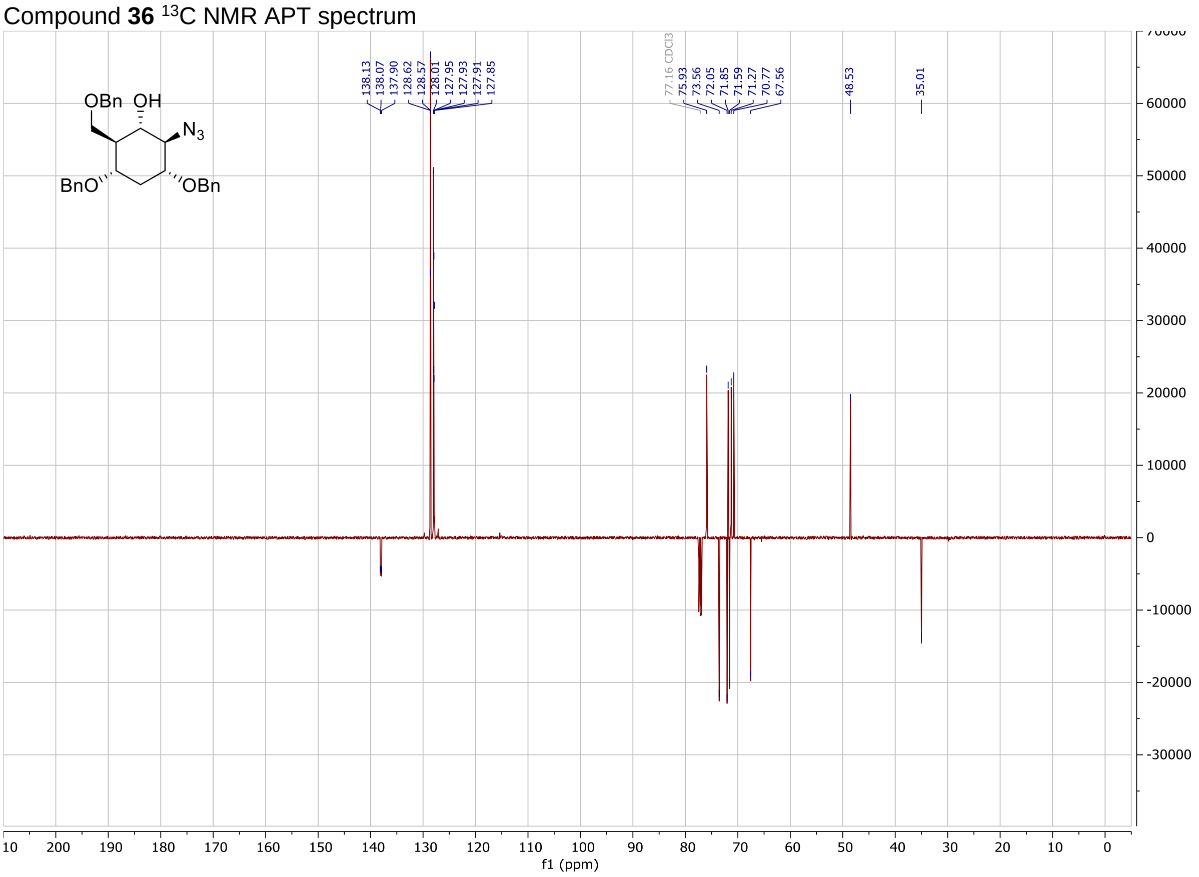

Compound **36** ^{1}H-^{1}H COSY spectrum

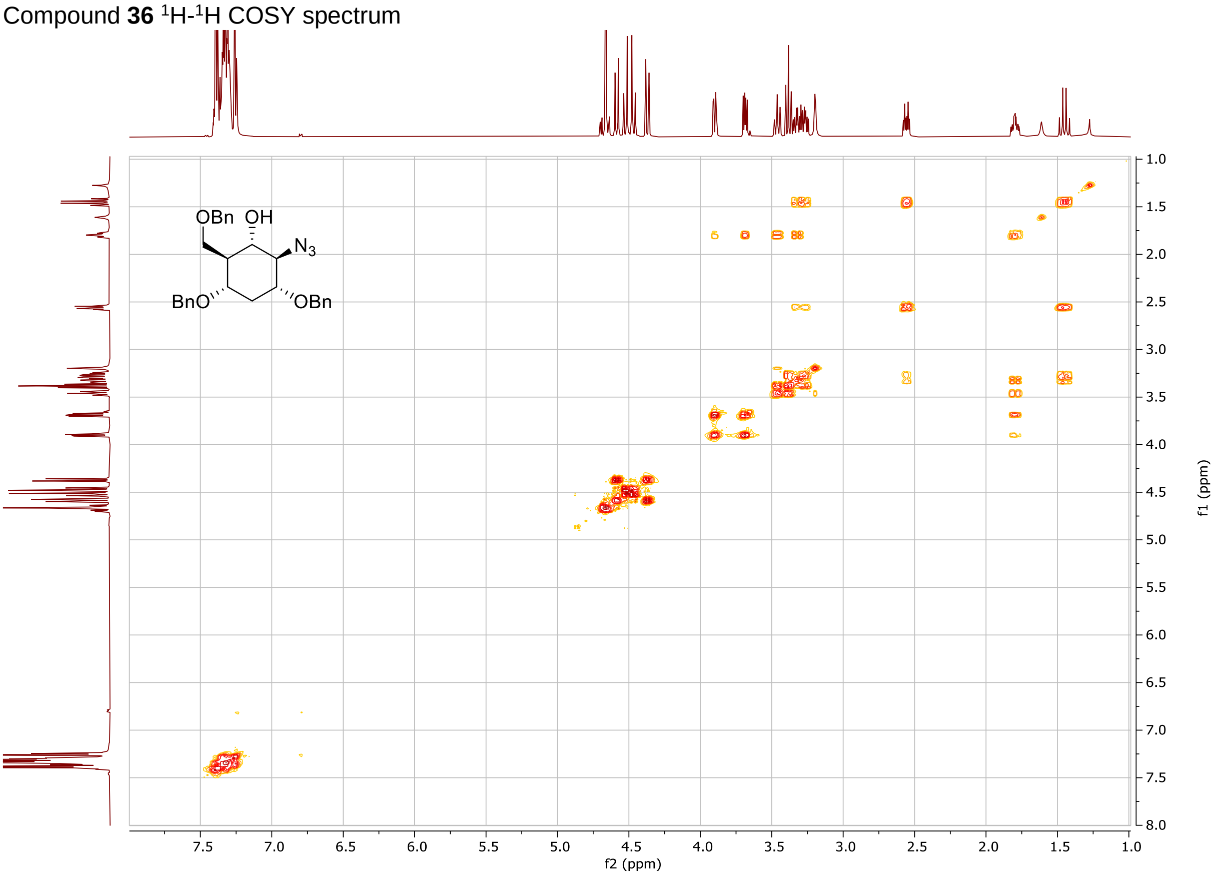

Compound **36** ^{1}H-^{13}C HSQC spectrum

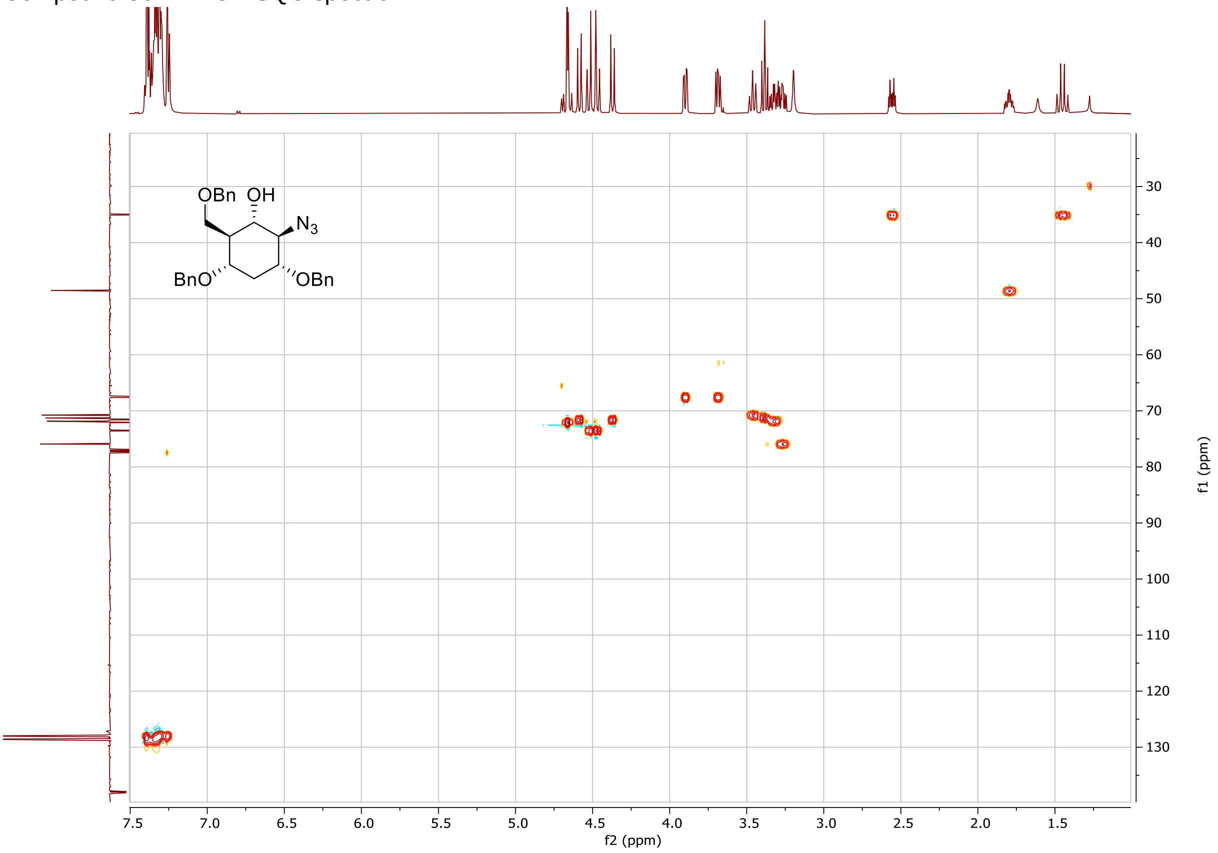

Compound **36** ^{1}H-^{1}H NOESY spectrum

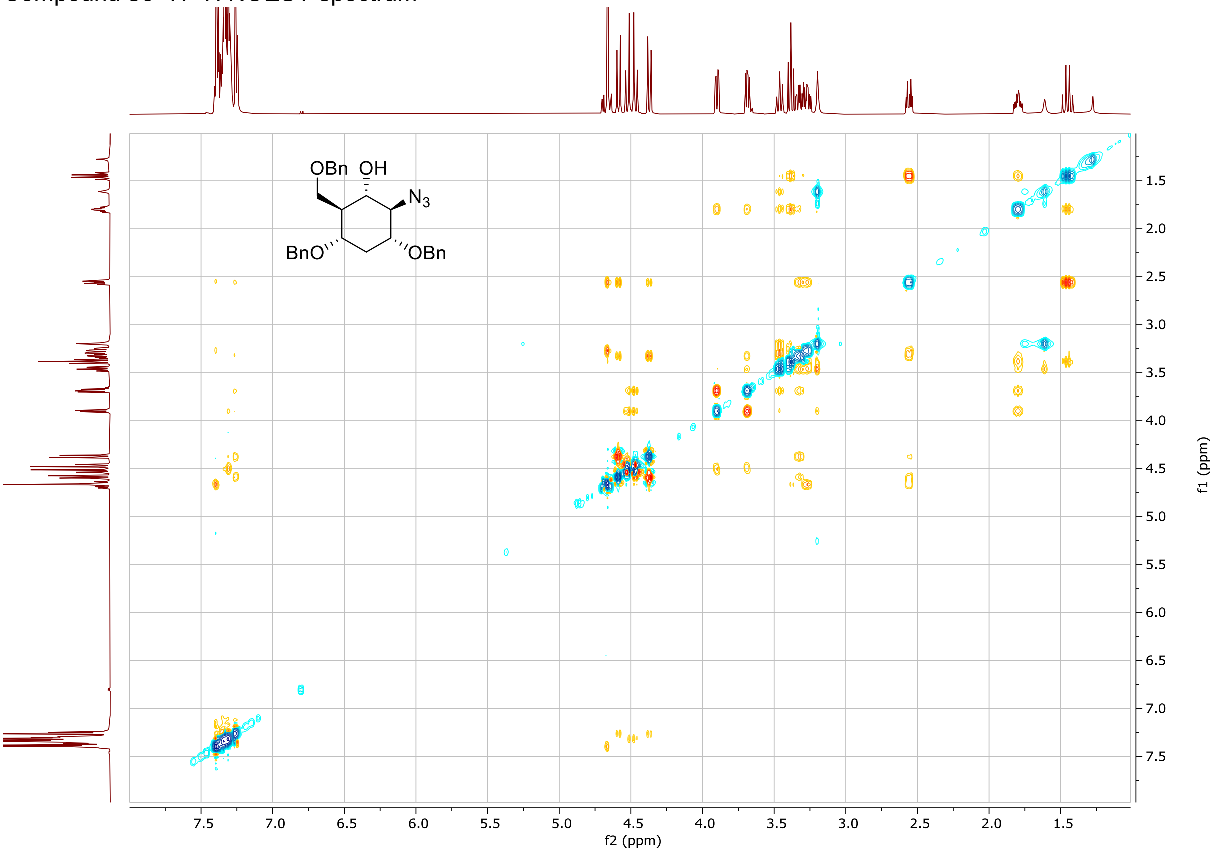

Compound **36** ^{1}H-^{13}C HMBC spectrum

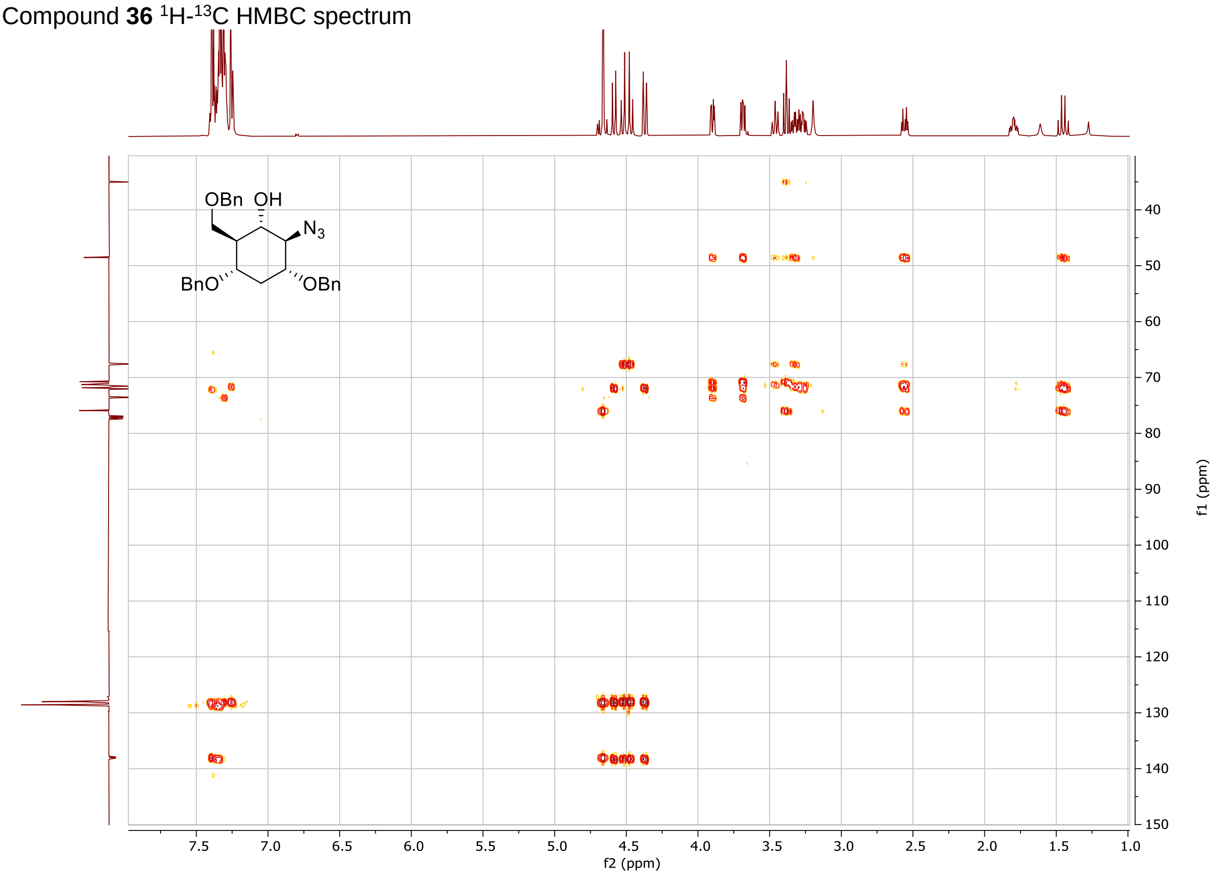

Compound **37** ^{1}H NMR spectrum

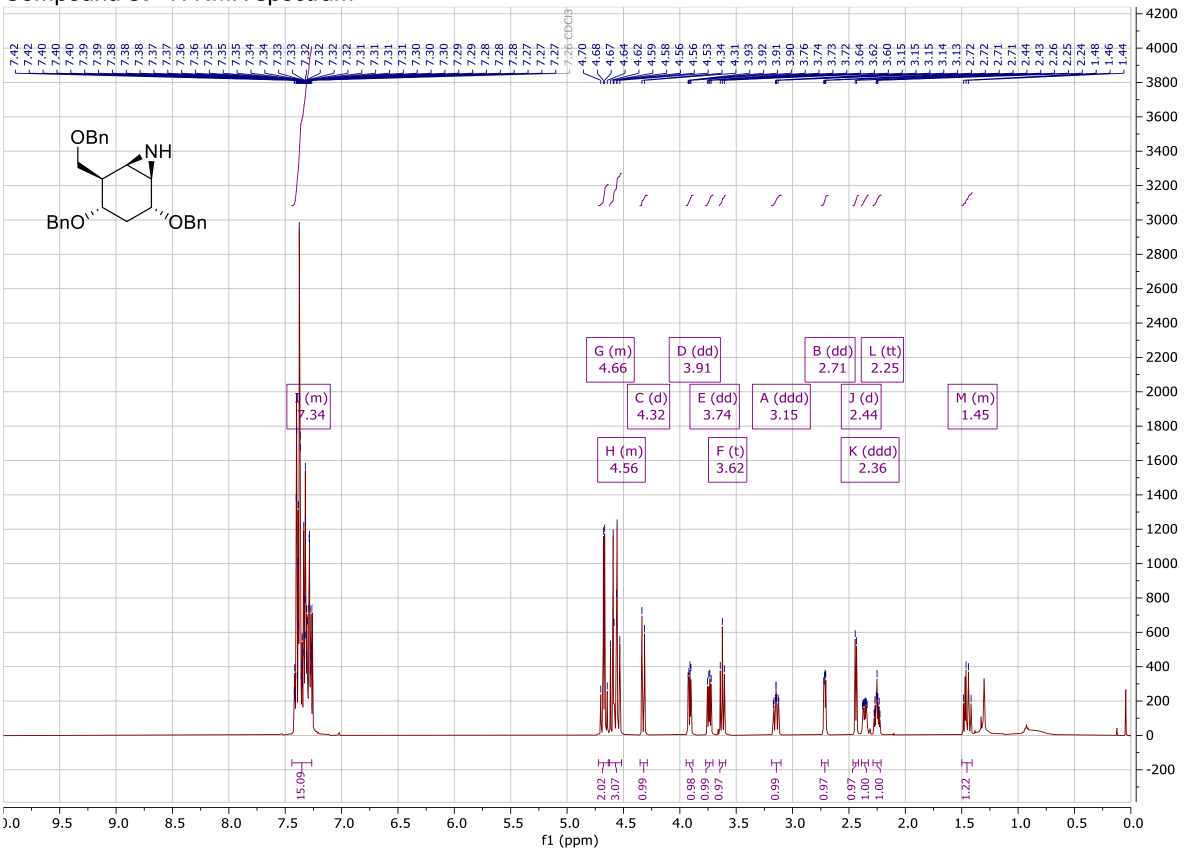

Compound **37** ^{13}C NMR APT spectrum

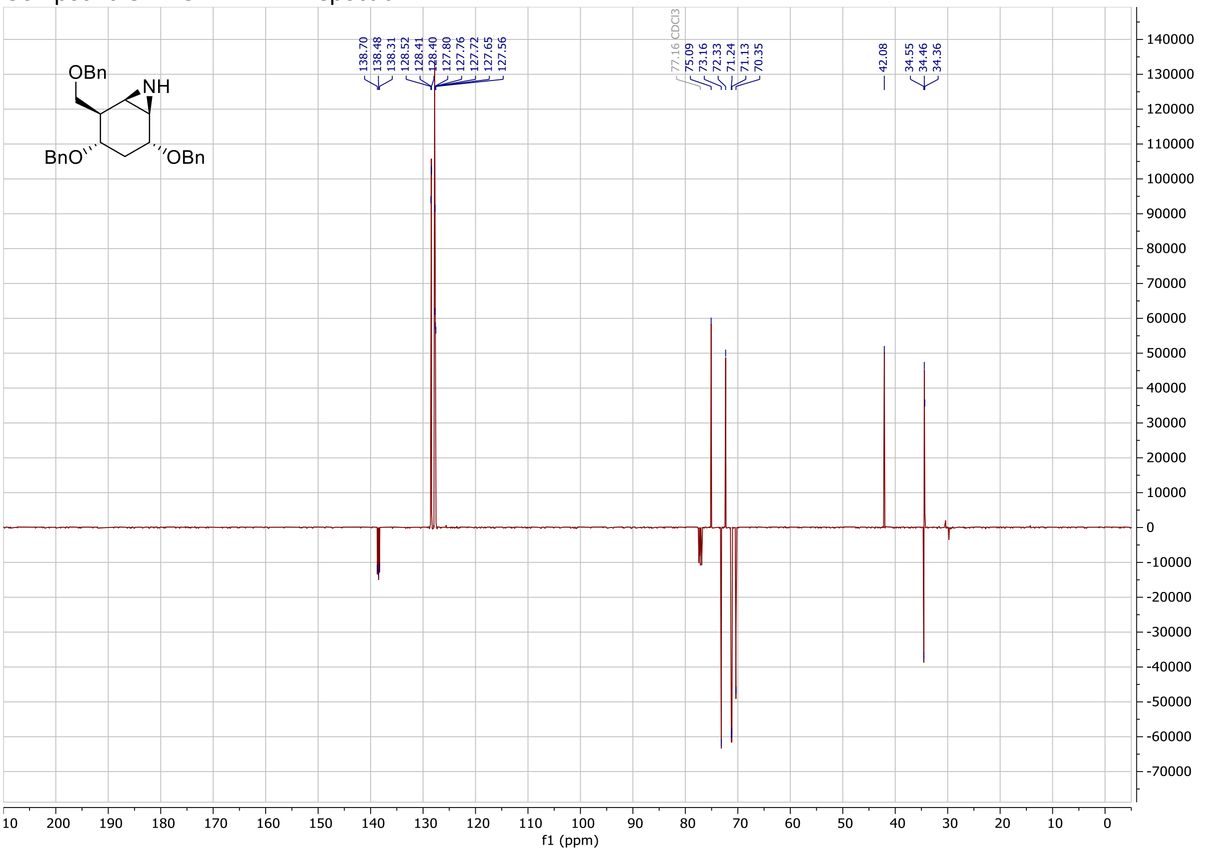

Compound **37** ^{1}H-^{1}H COSY spectrum

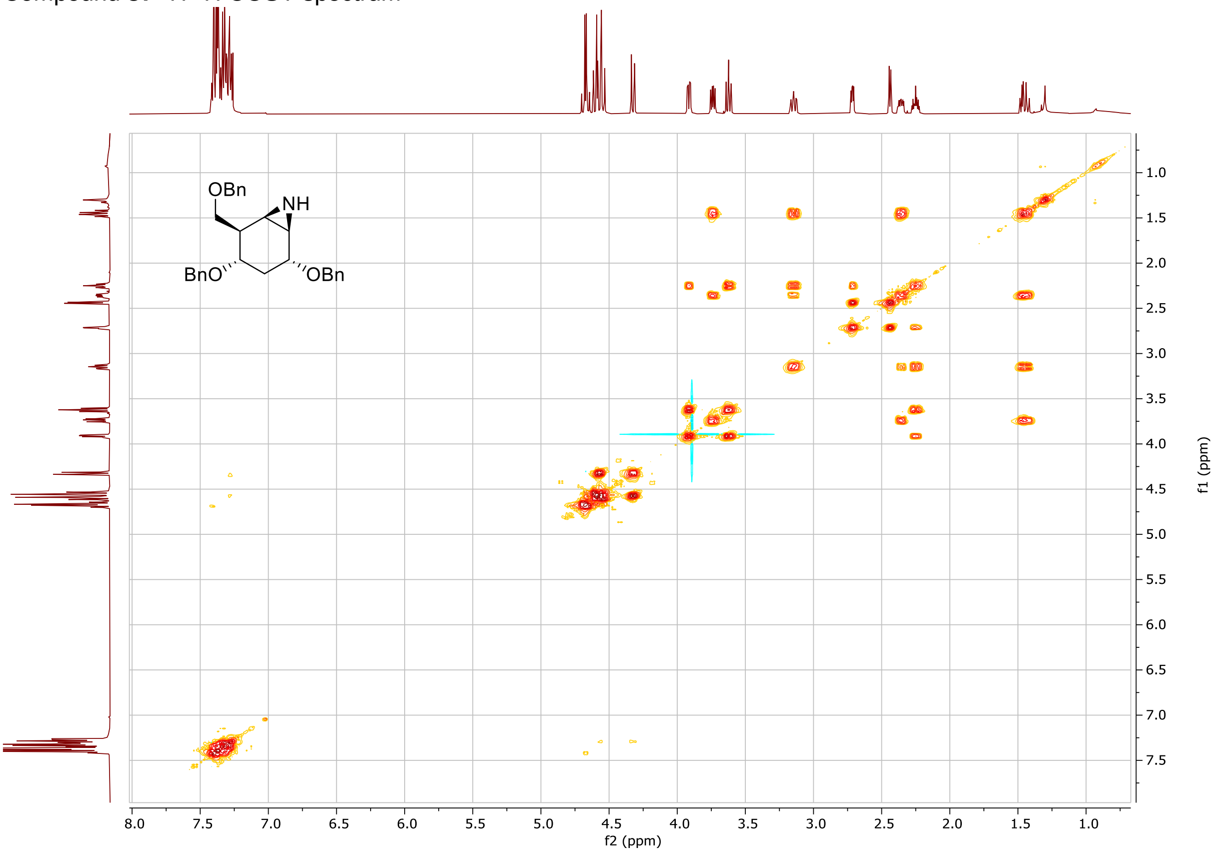

Compound **37** ^{1}H-^{13}C HSQC spectrum

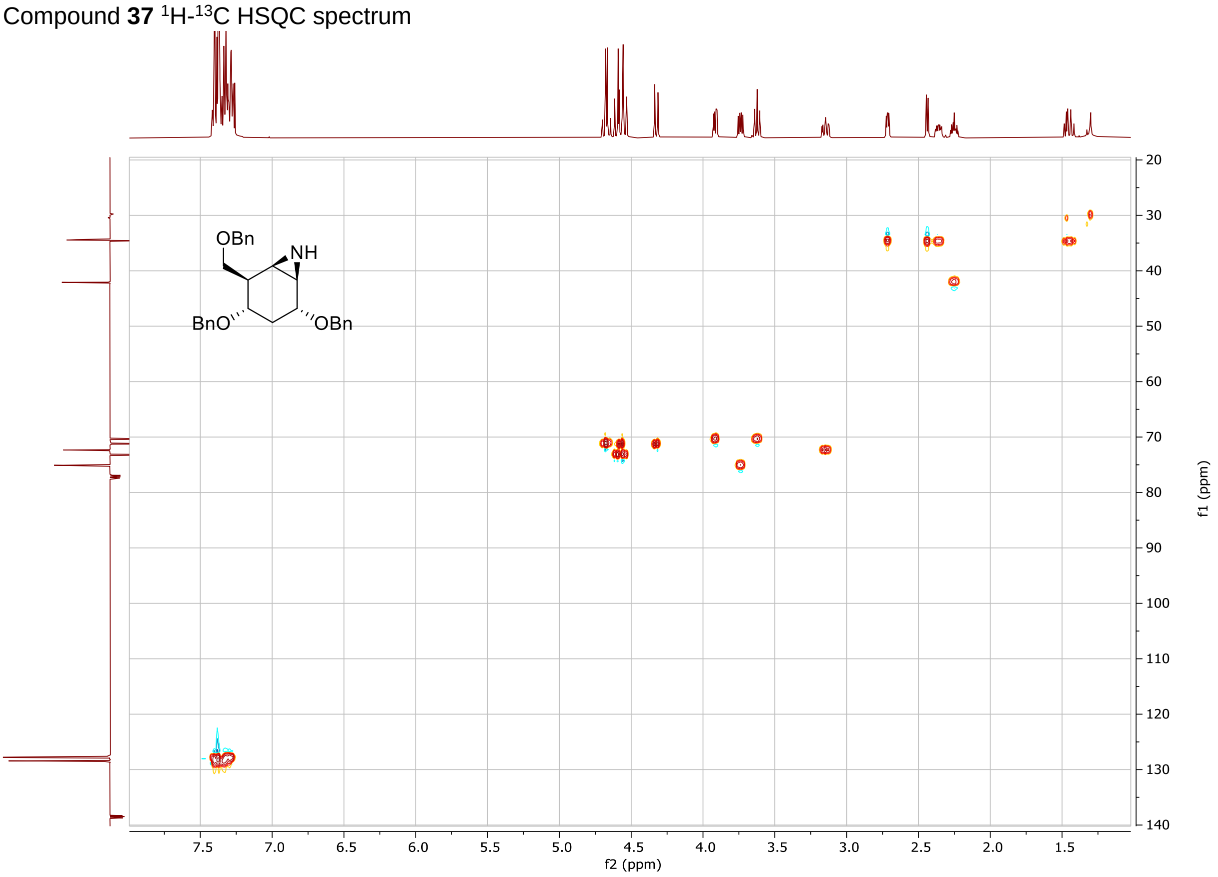

Compound **37** ^{1}H-^{1}H NOESY spectrum

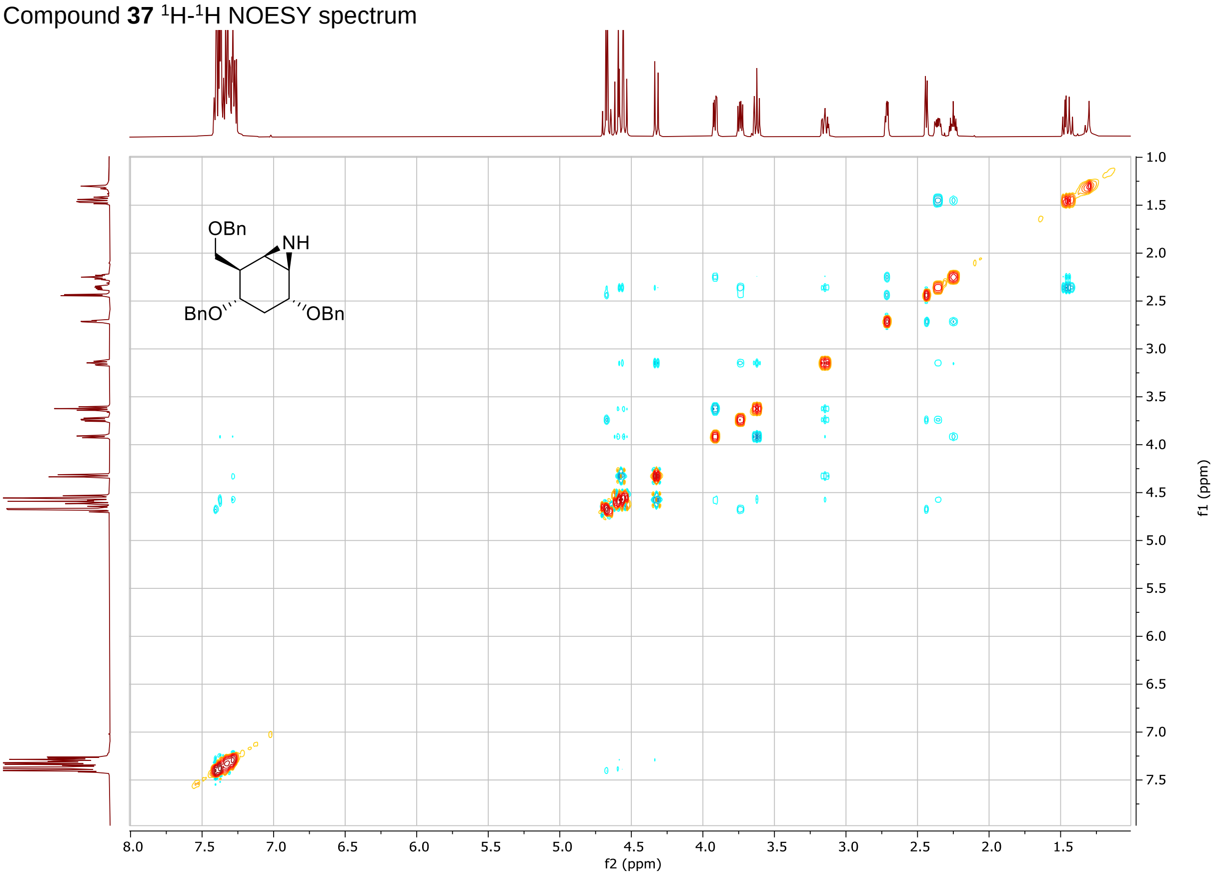

Compound **37** ^{1}H-^{13}C HMBC spectrum

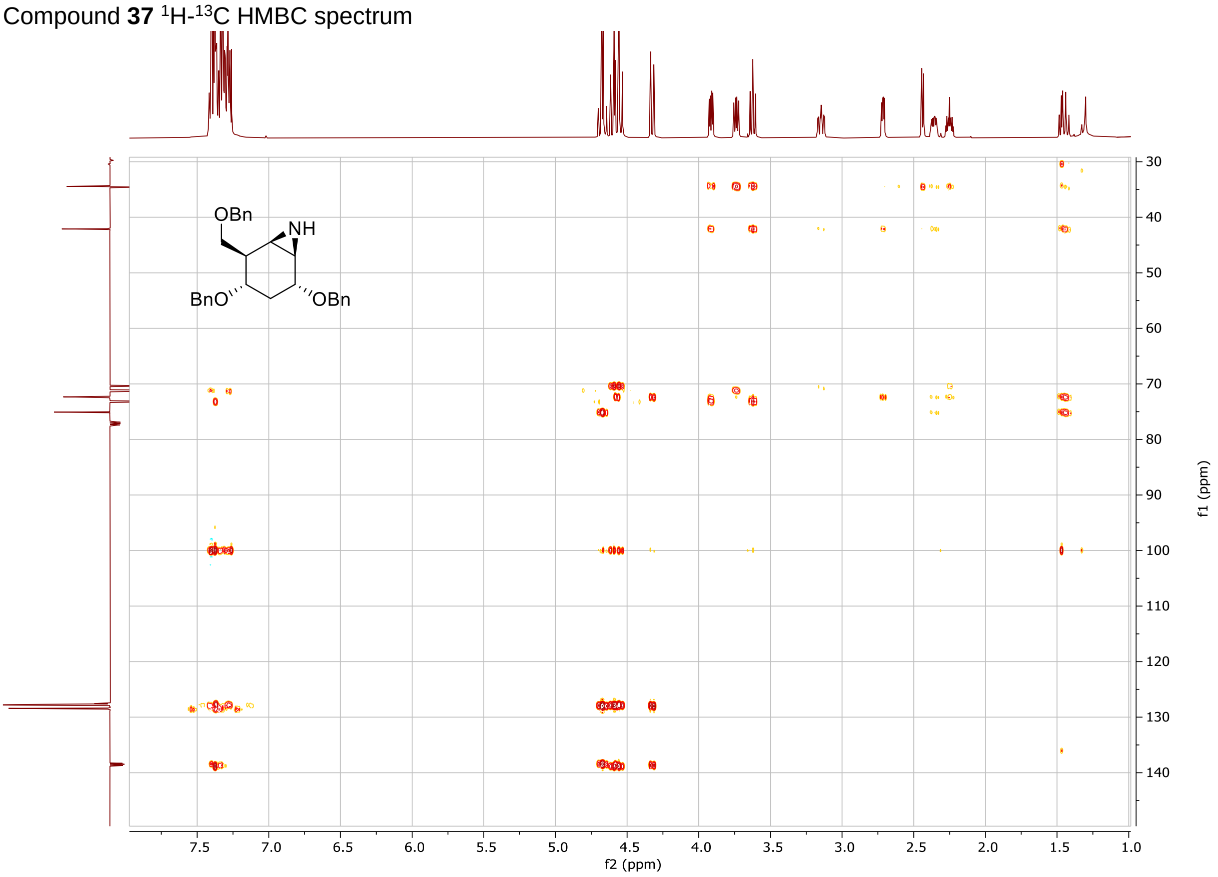

Compound **38** ^{1}H NMR spectrum

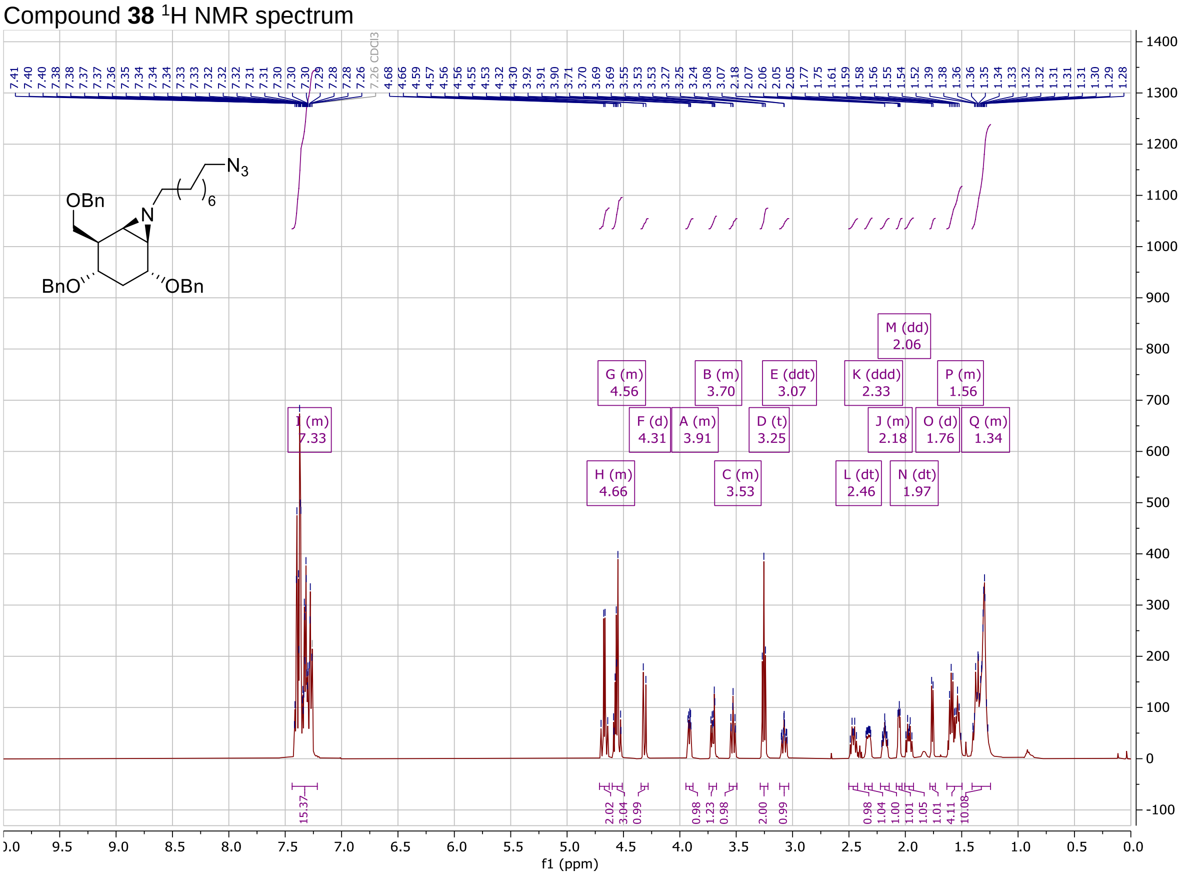

Compound **38** ^{13}C NMR APT spectrum

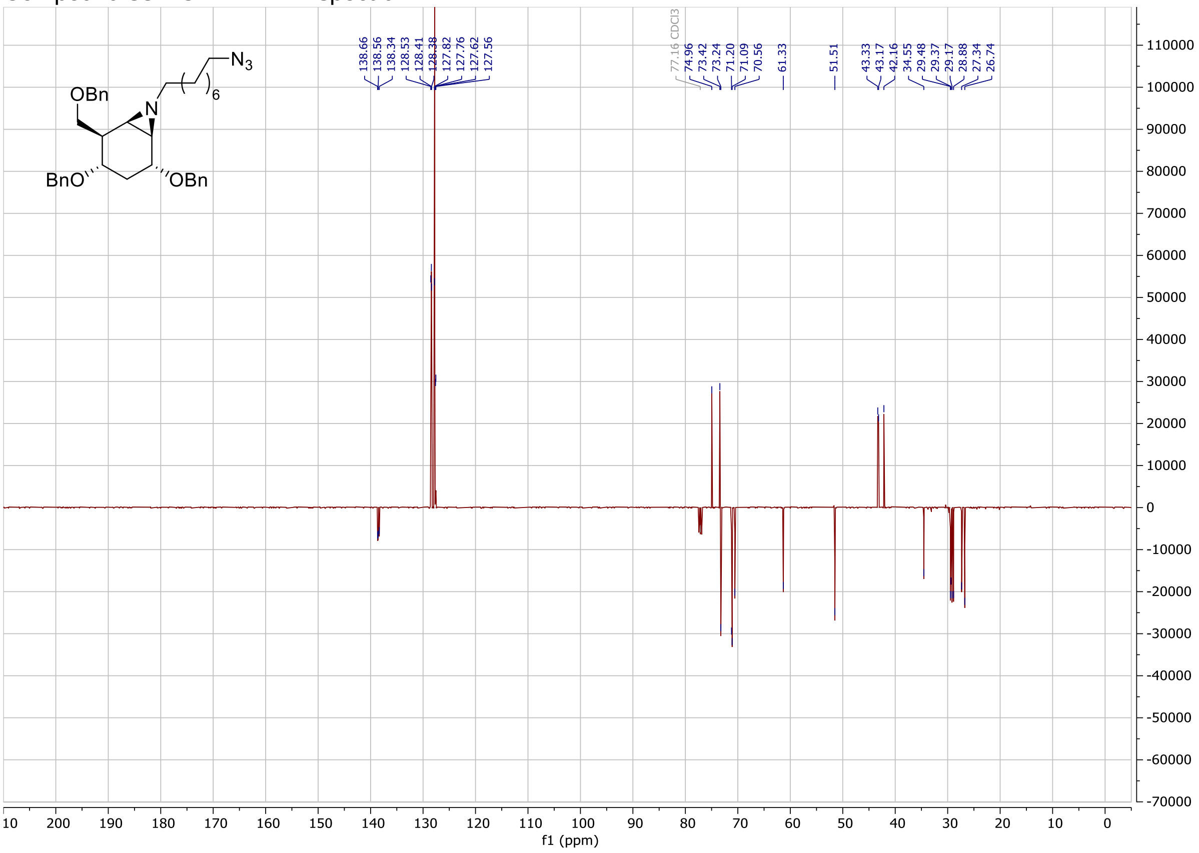

Compound **38** ^{1}H-^{1}H COSY spectrum

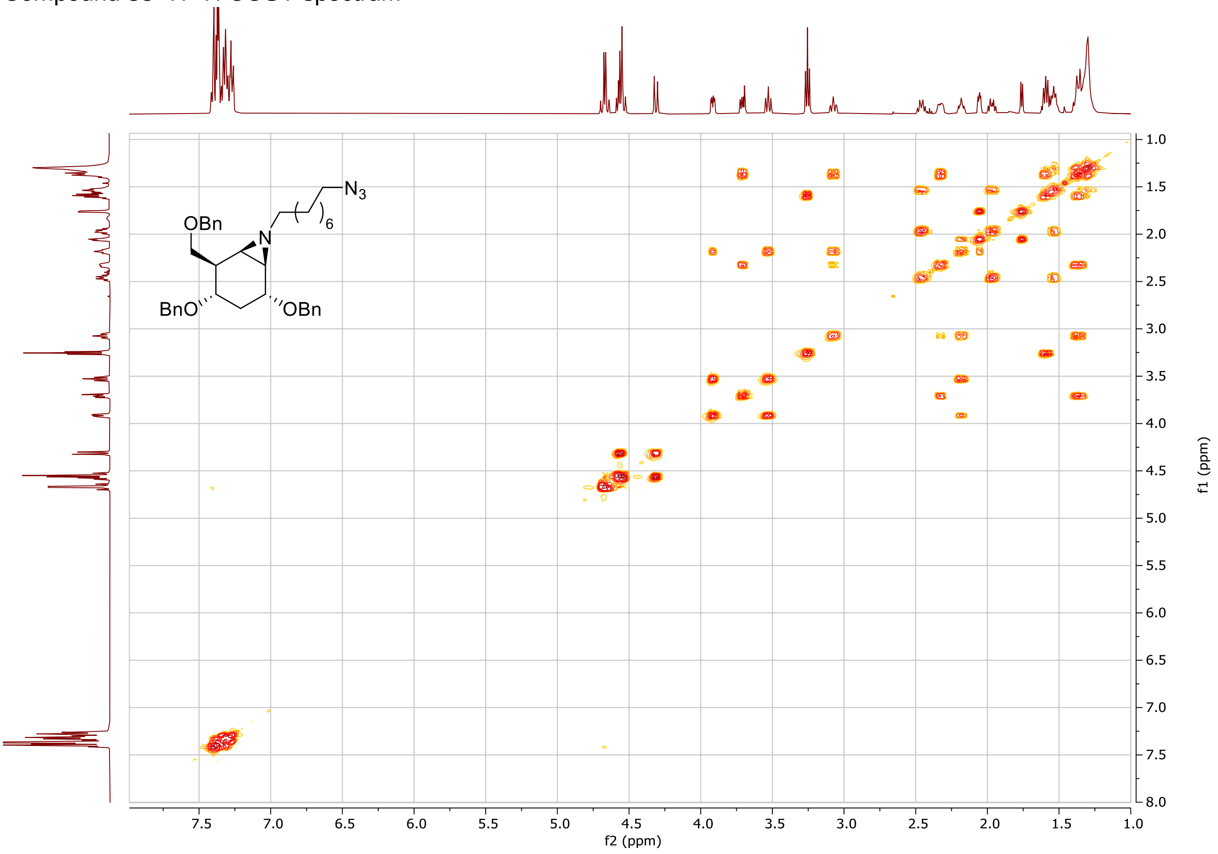

Compound **38** ^{1}H-^{13}C HSQC spectrum

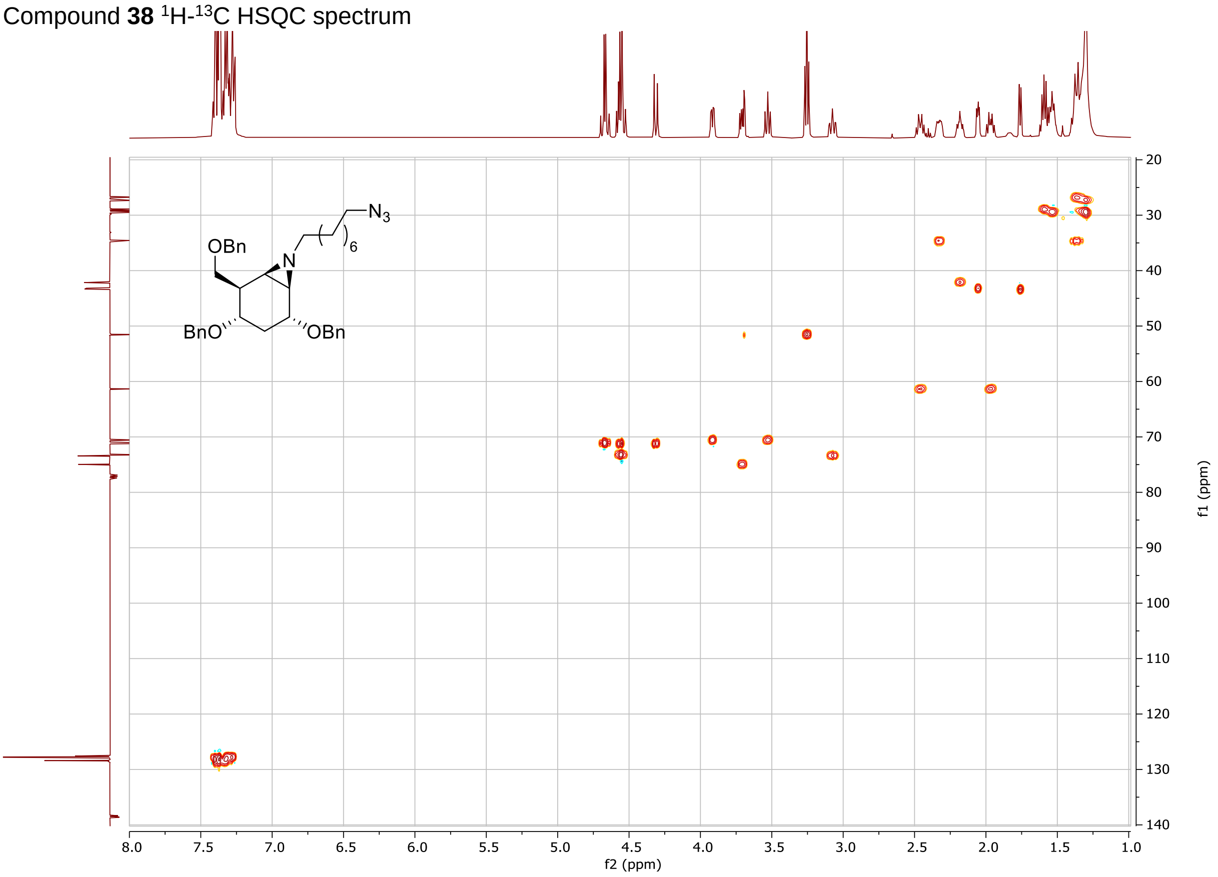

Compound **38** ^{1}H-^{1}H NOESY spectrum

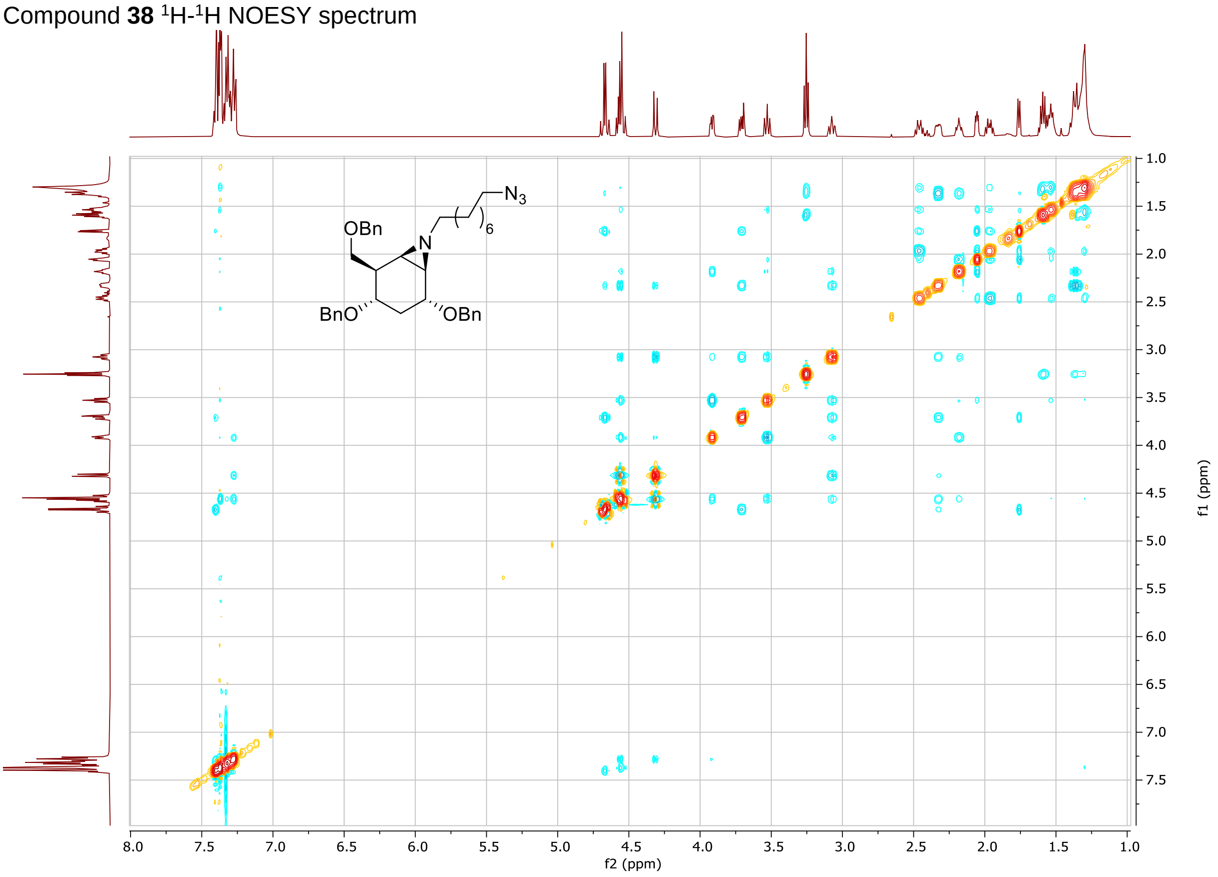

Compound **39** ^{1}H NMR spectrum

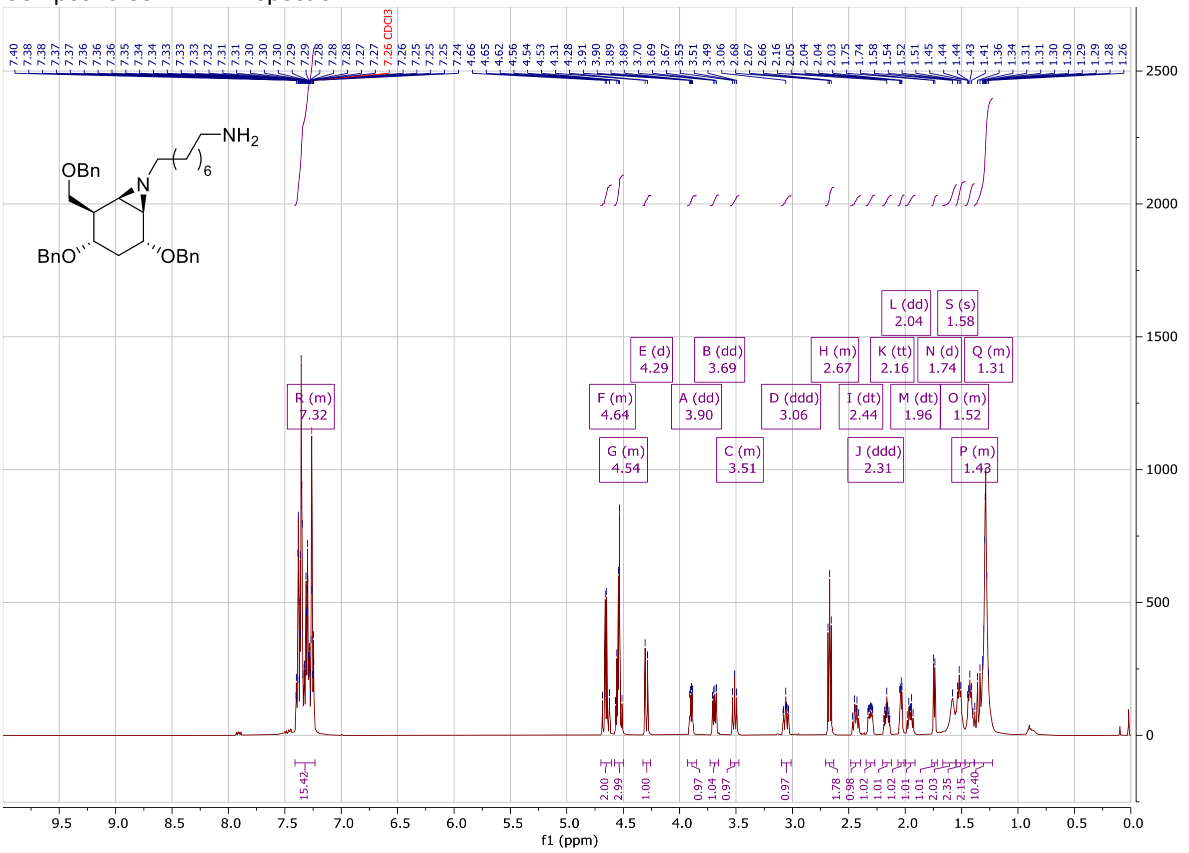

Compound **39** ^{13}C NMR APT spectrum

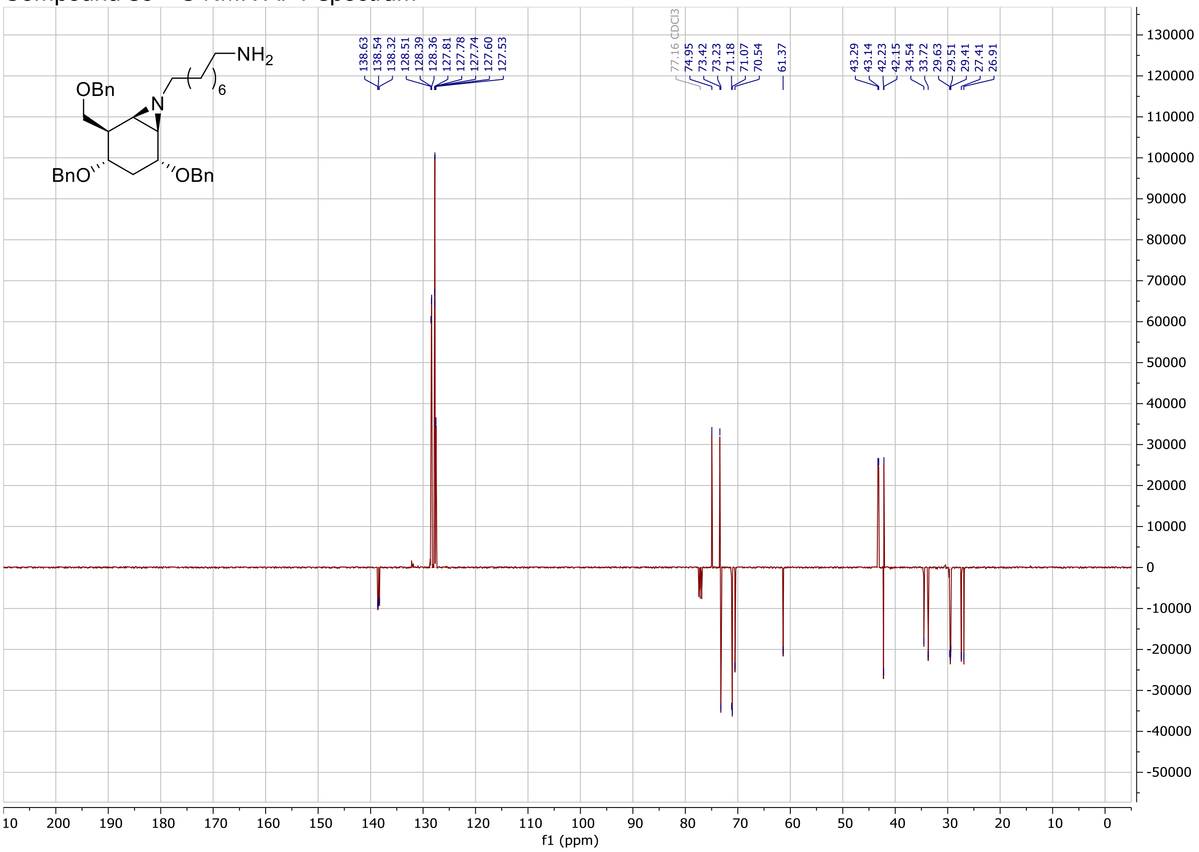

Compound **39** ^{1}H-^{1}H COSY spectrum

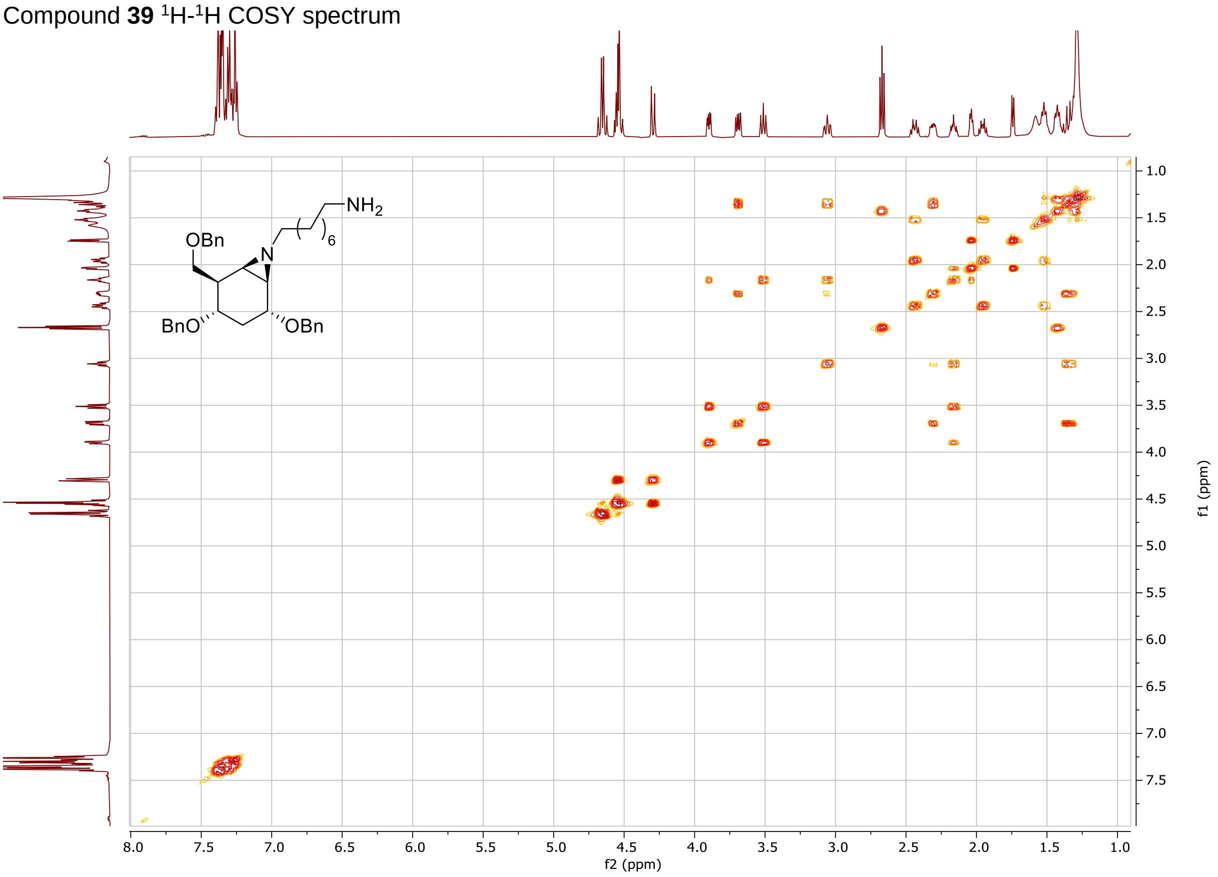

Compound **39** ^{1}H-^{13}C HSQC spectrum

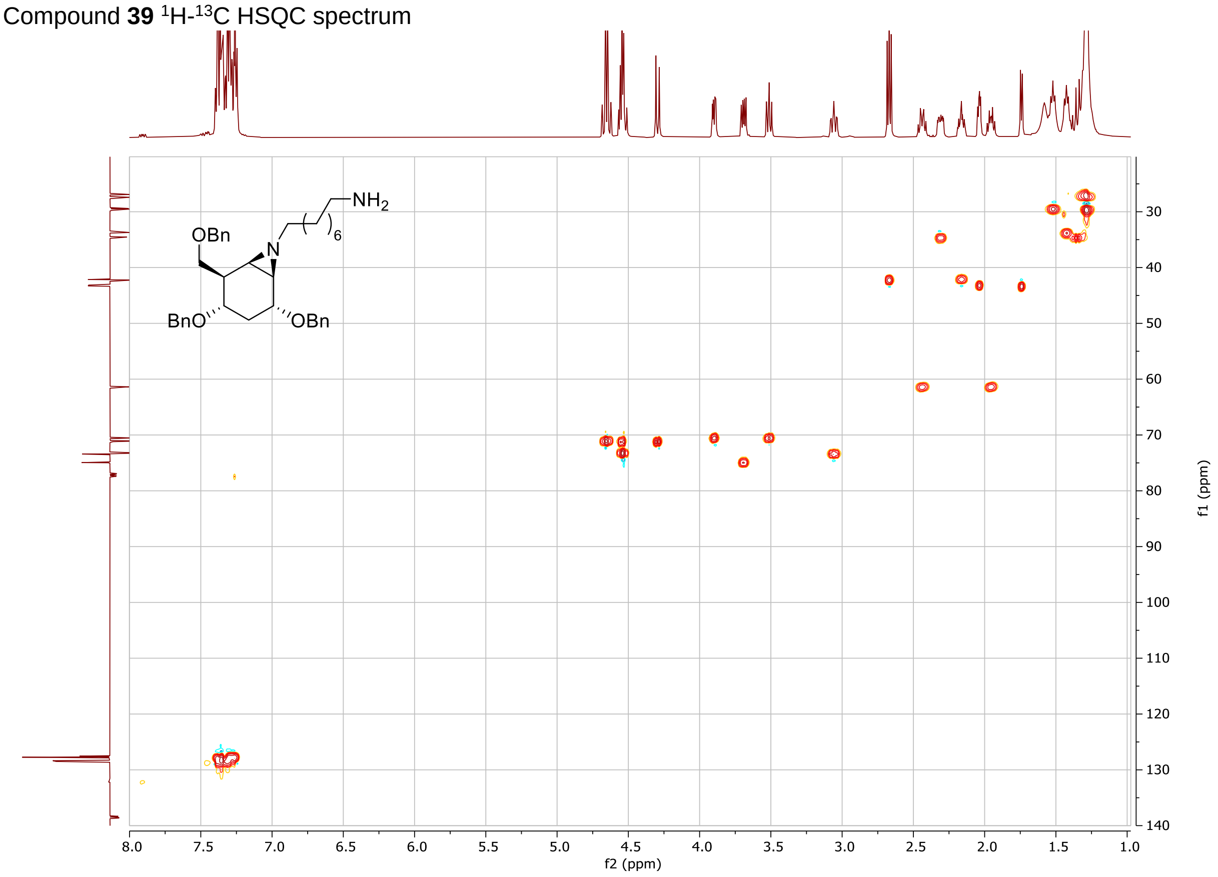

Compound **39** ^{1}H-^{1}H NOESY spectrum

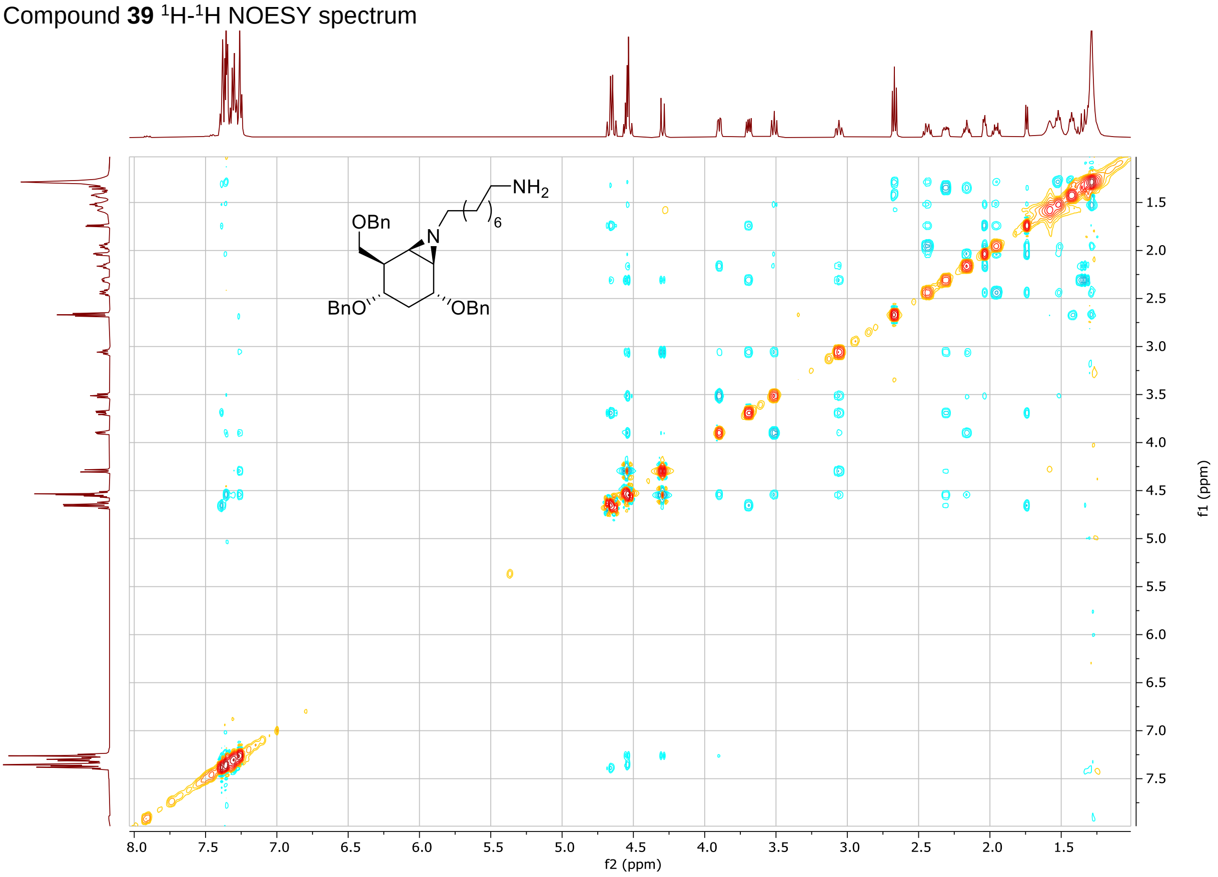

Compound **40** ^{1}H NMR spectrum

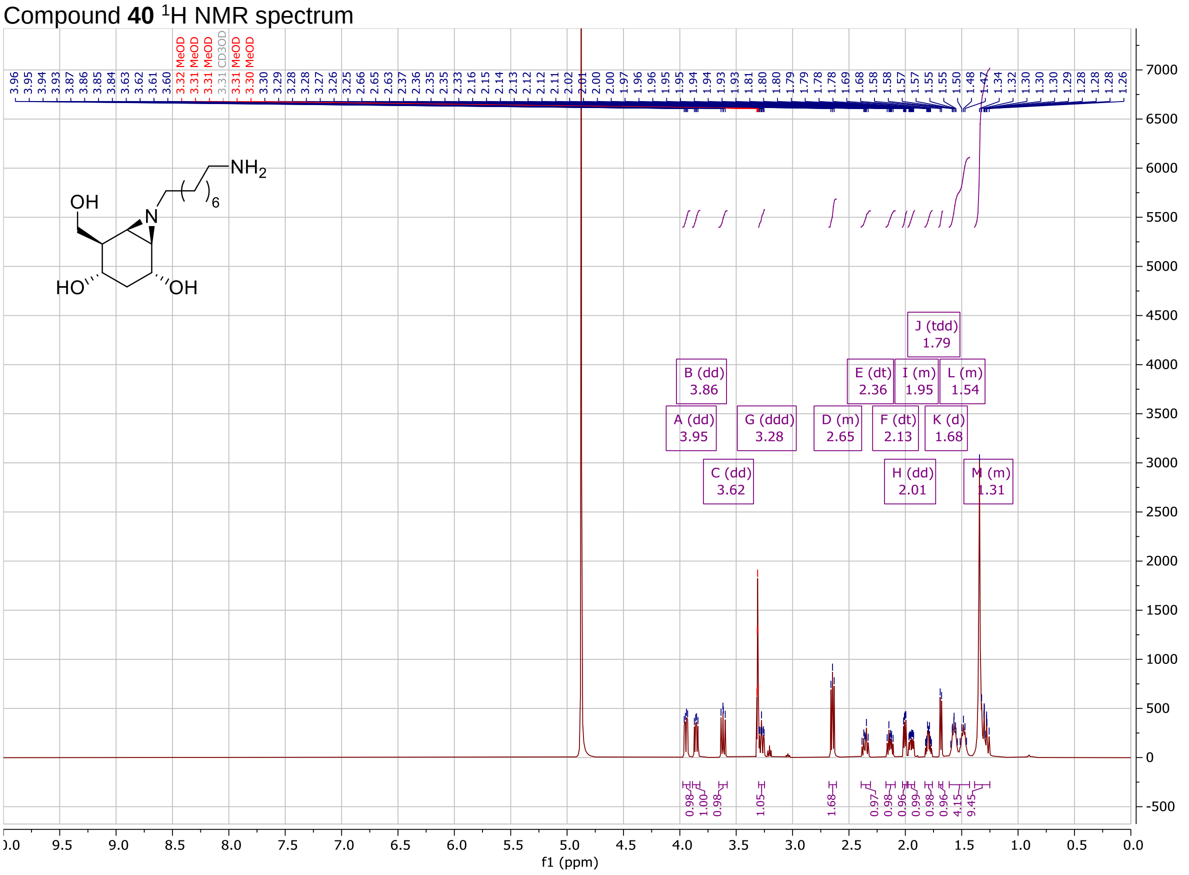

Compound **40** ^{13}C NMR APT spectrum

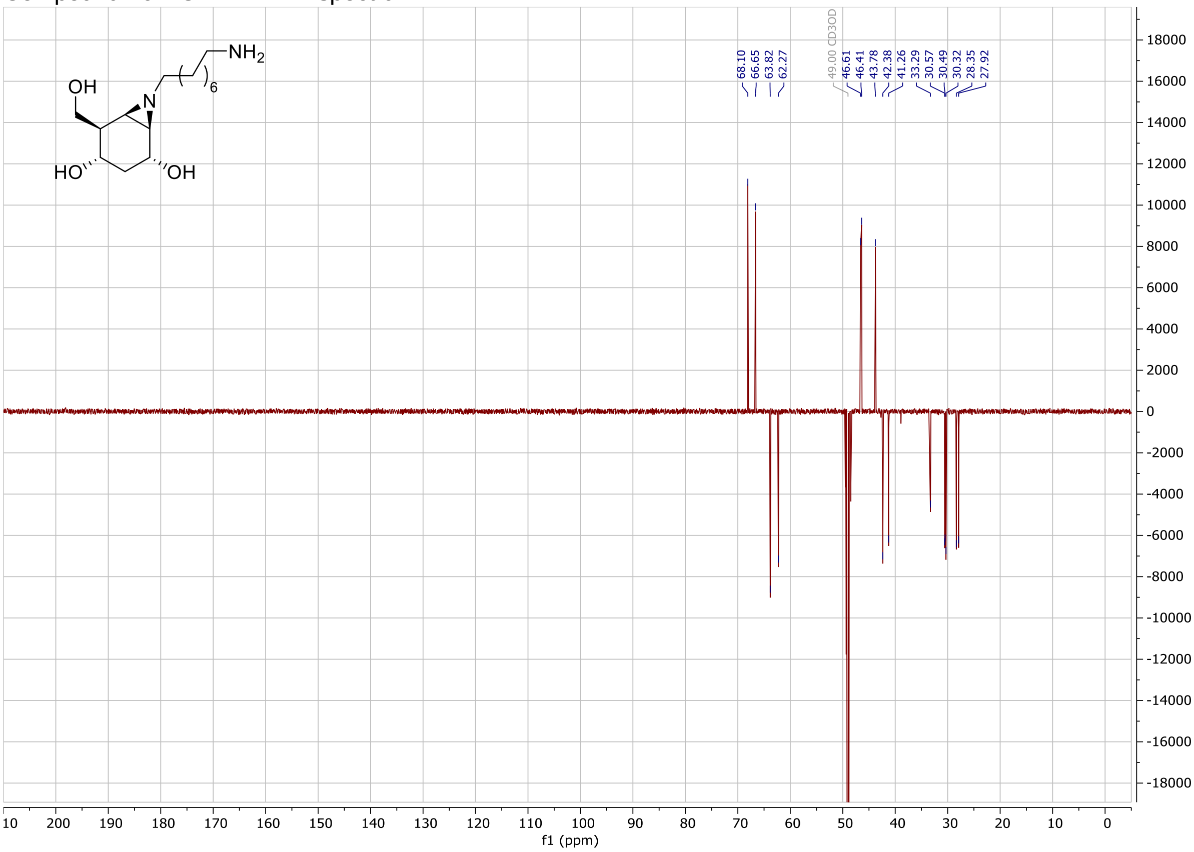

Compound **40** ^{1}H-^{1}H COSY spectrum

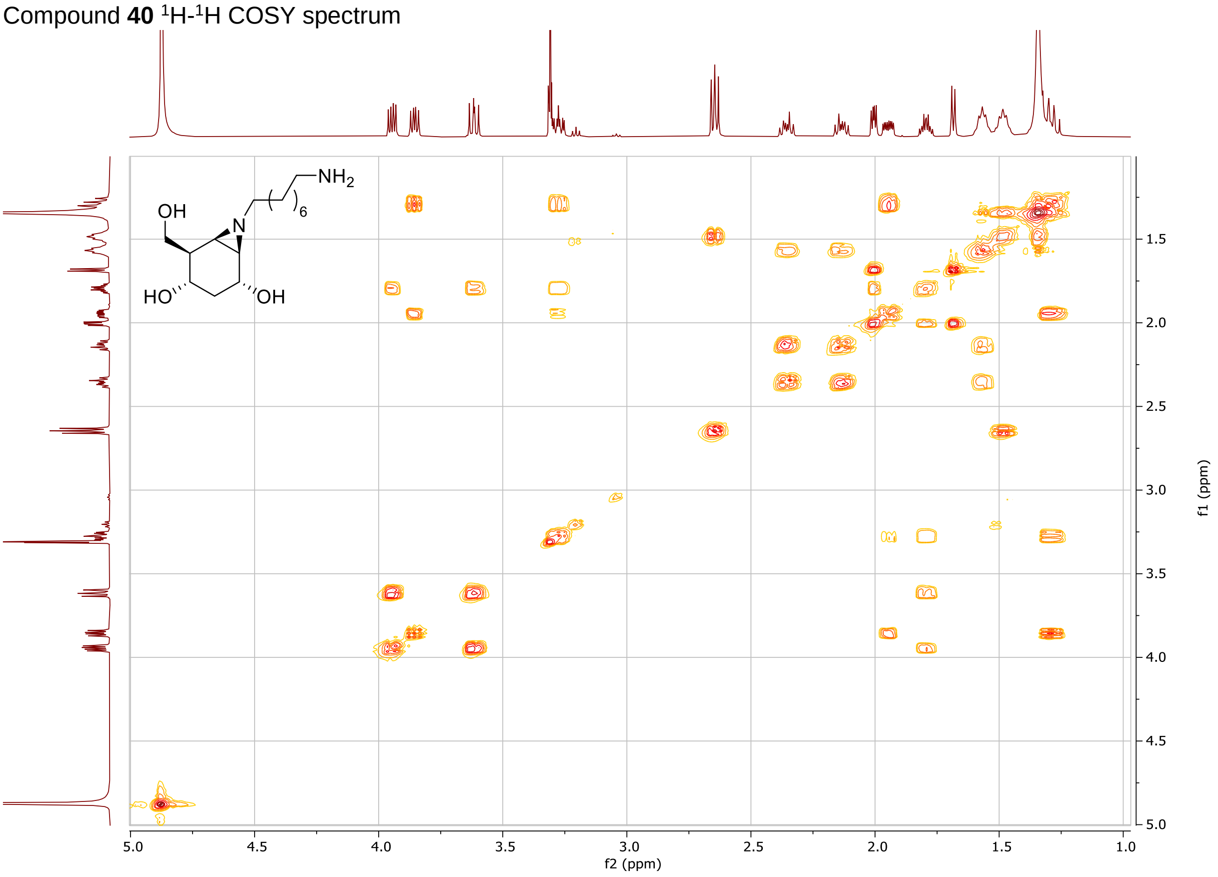

Compound **40** ^{1}H-^{13}C HSQC spectrum

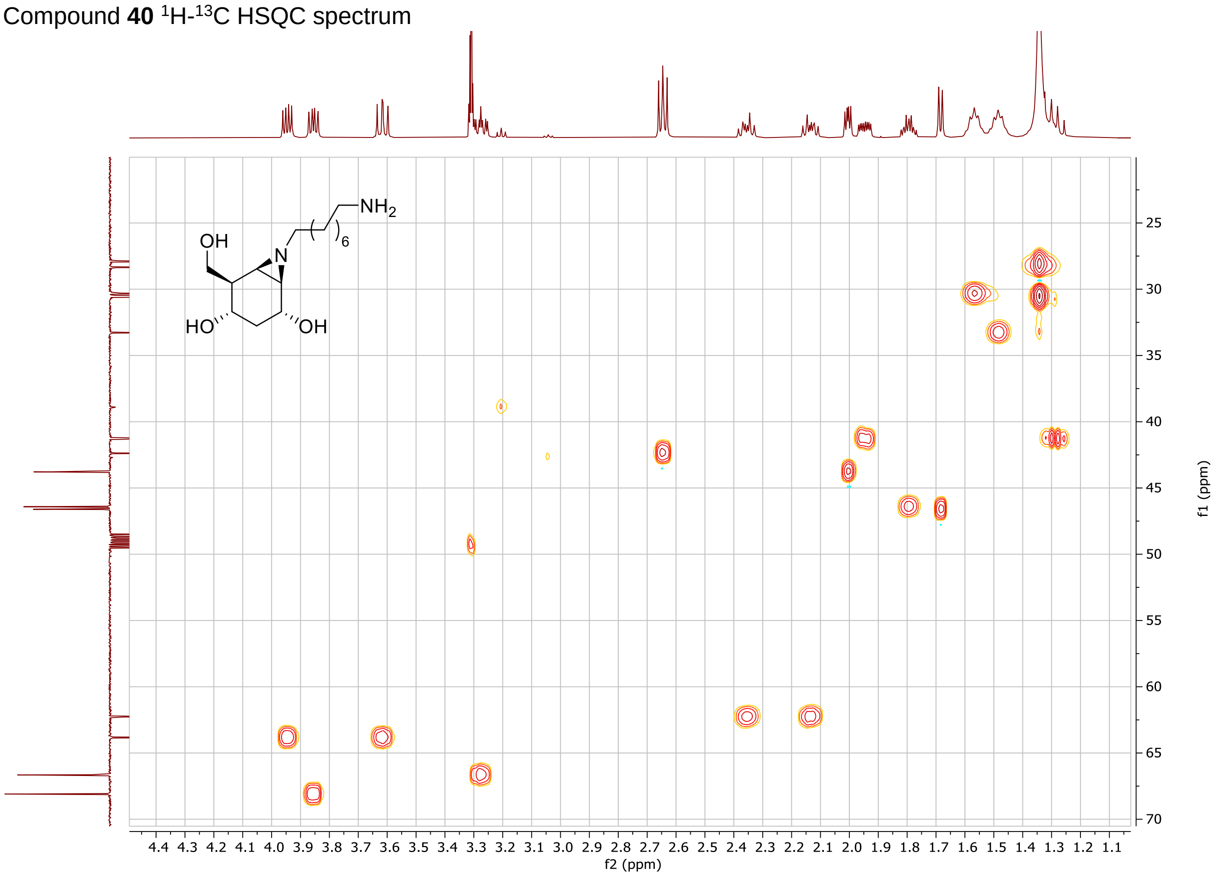

Compound **41** ^{1}H NMR spectrum

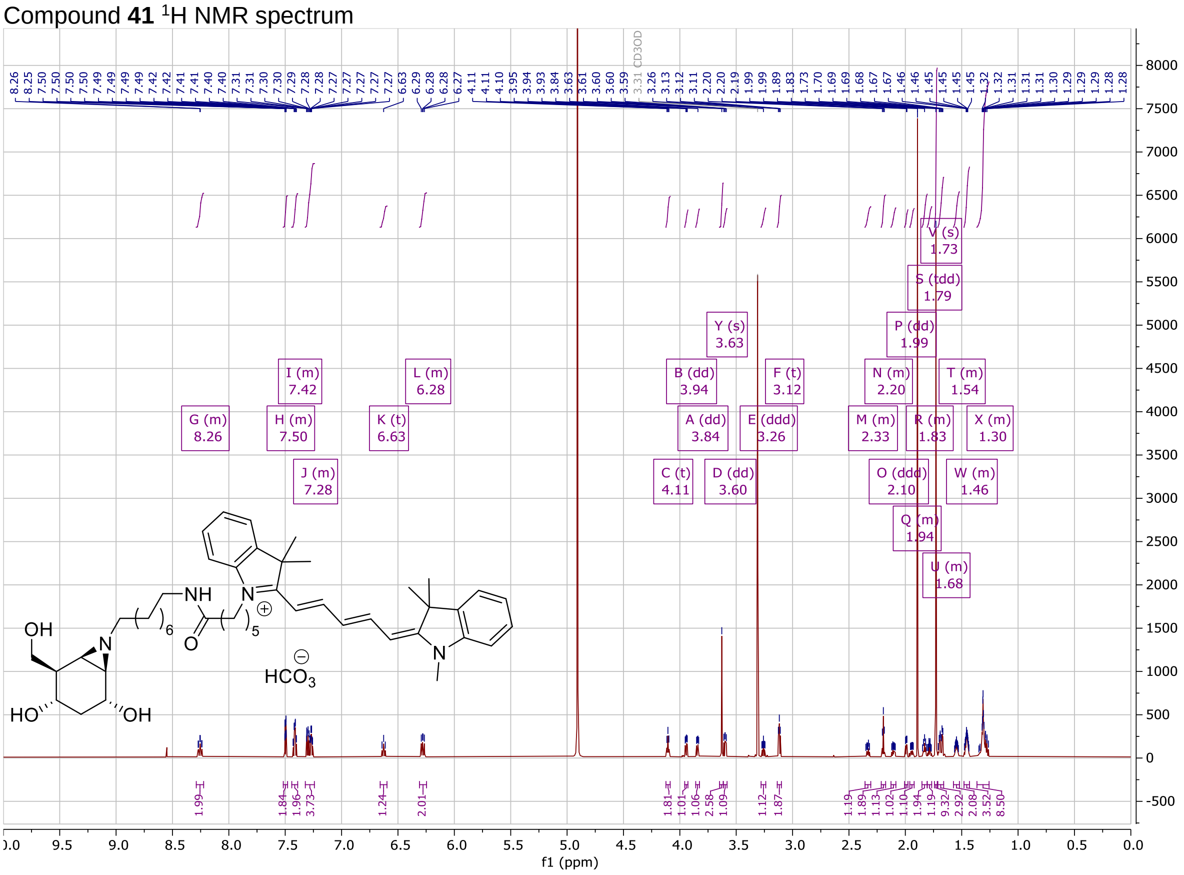

Compound **41** ^{13}C NMR APT spectrum

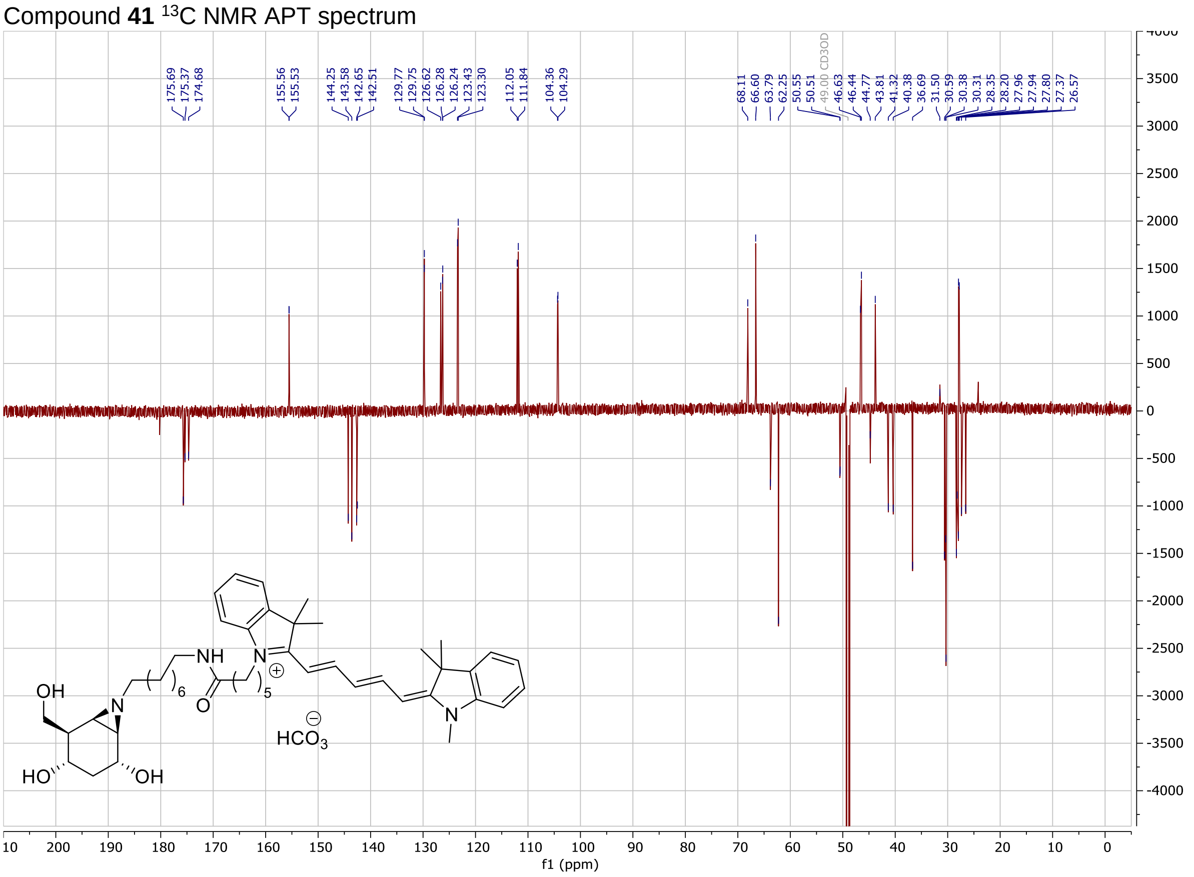

Compound **41** ^{1}H-^{1}H COSY spectrum

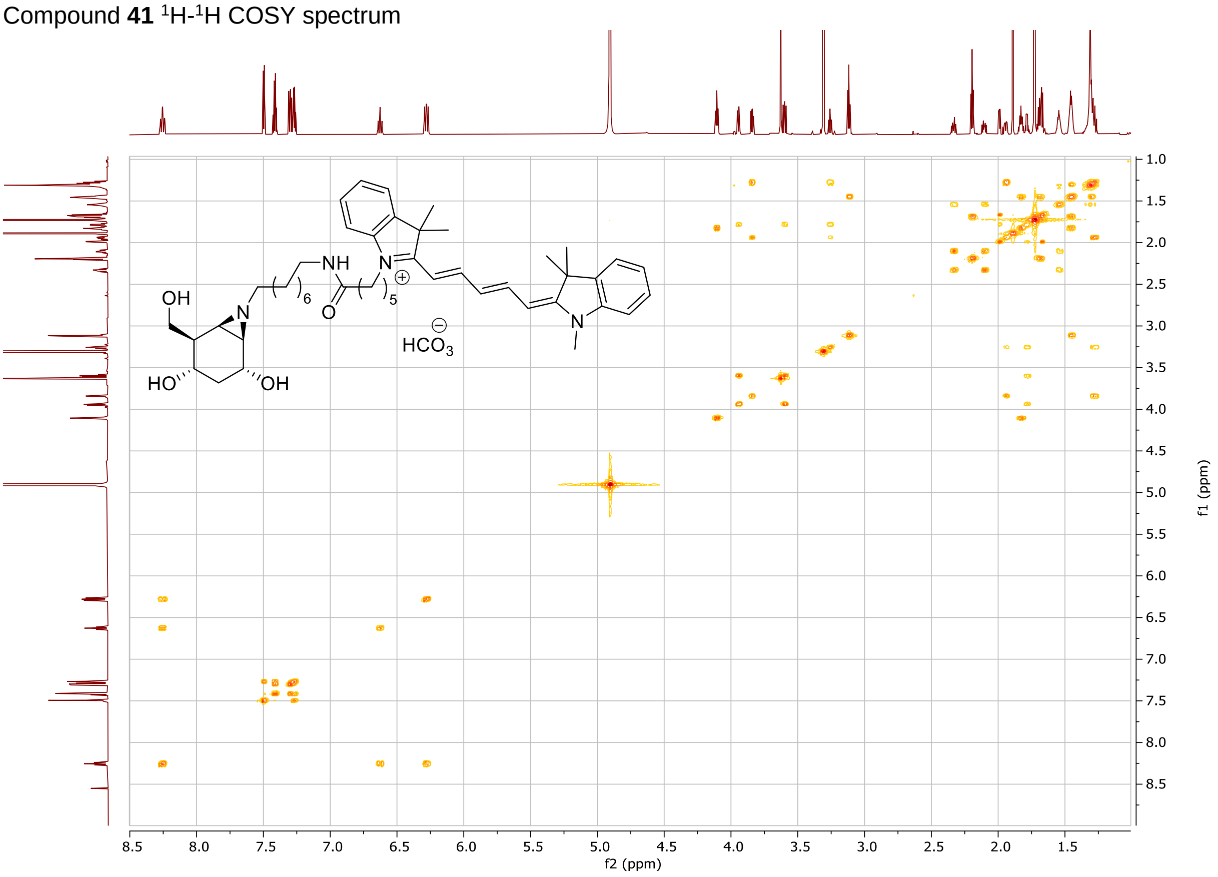

Compound **41** ^{1}H-^{13}C HSQC spectrum

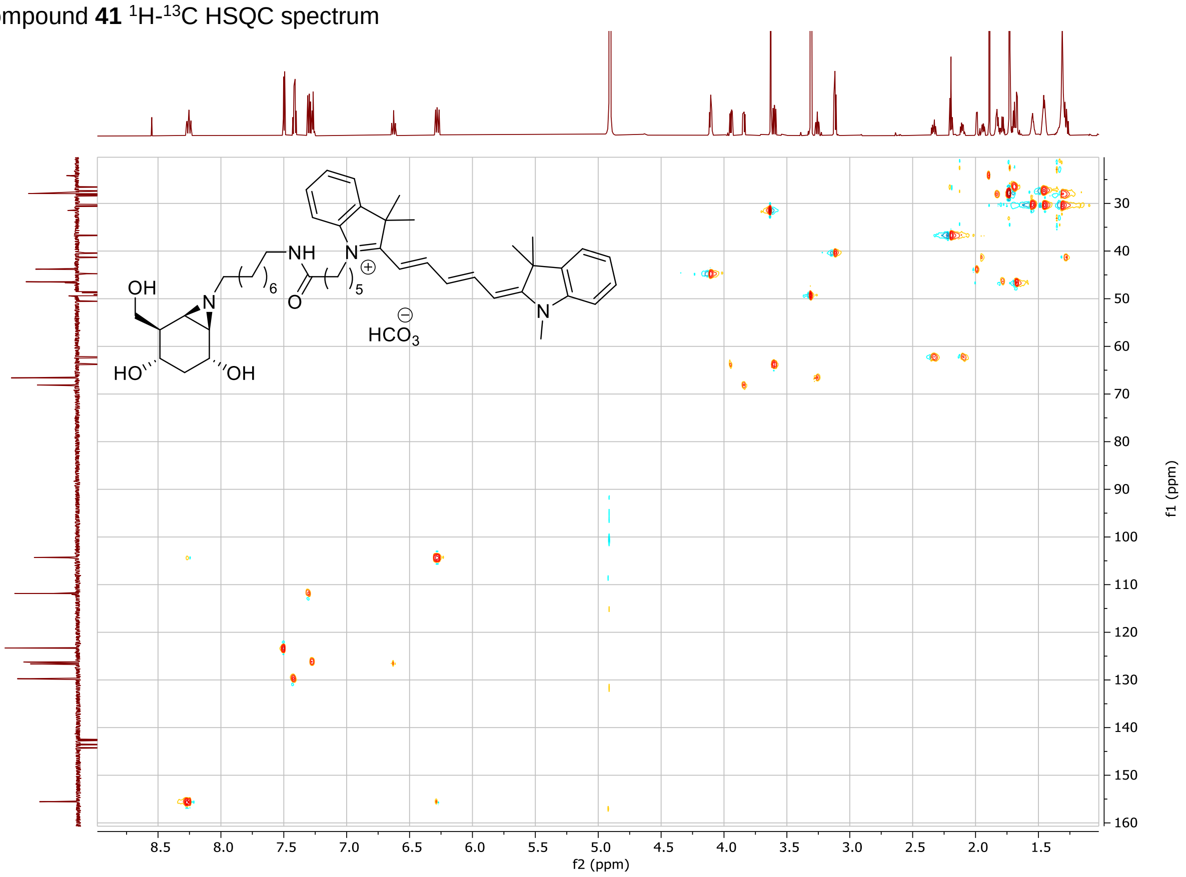

Compound **42** ^{1}H NMR spectrum

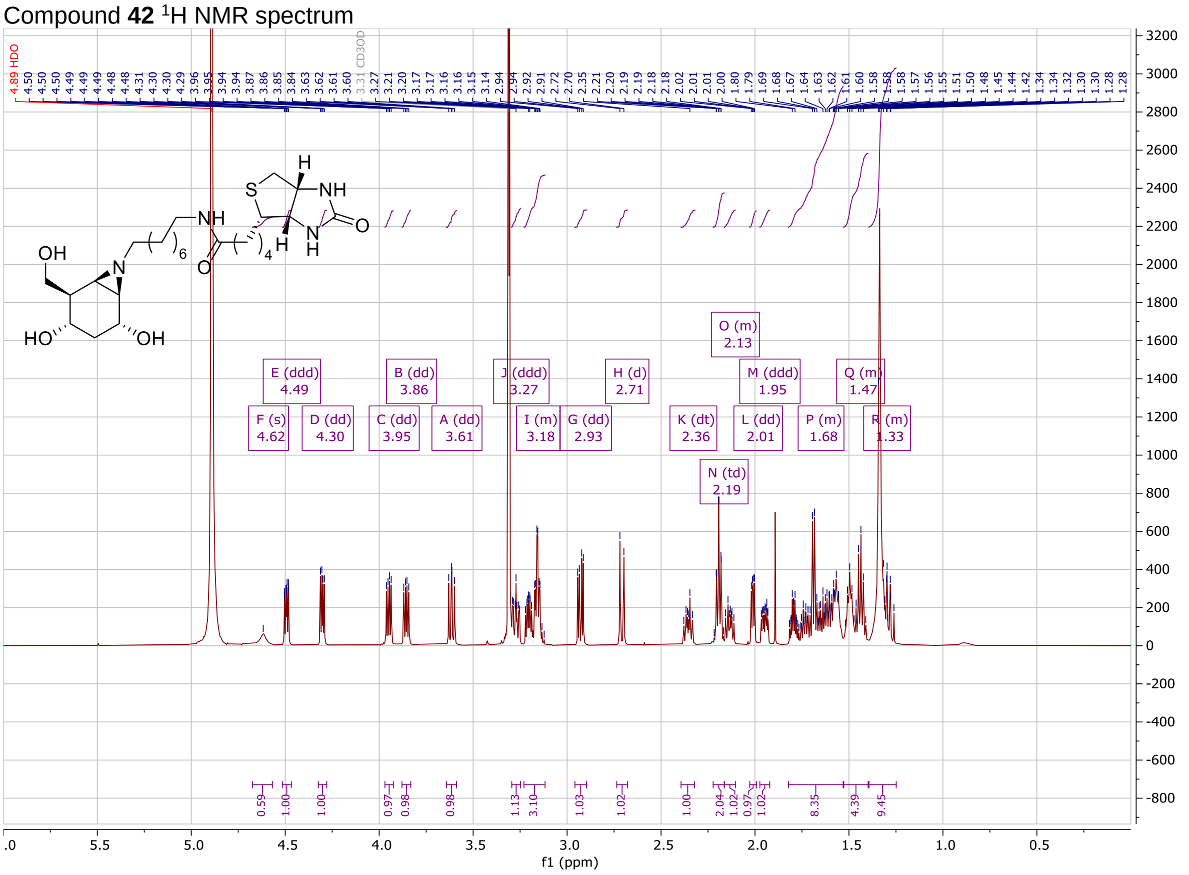

Compound **42** ^{13}C NMR APT spectrum

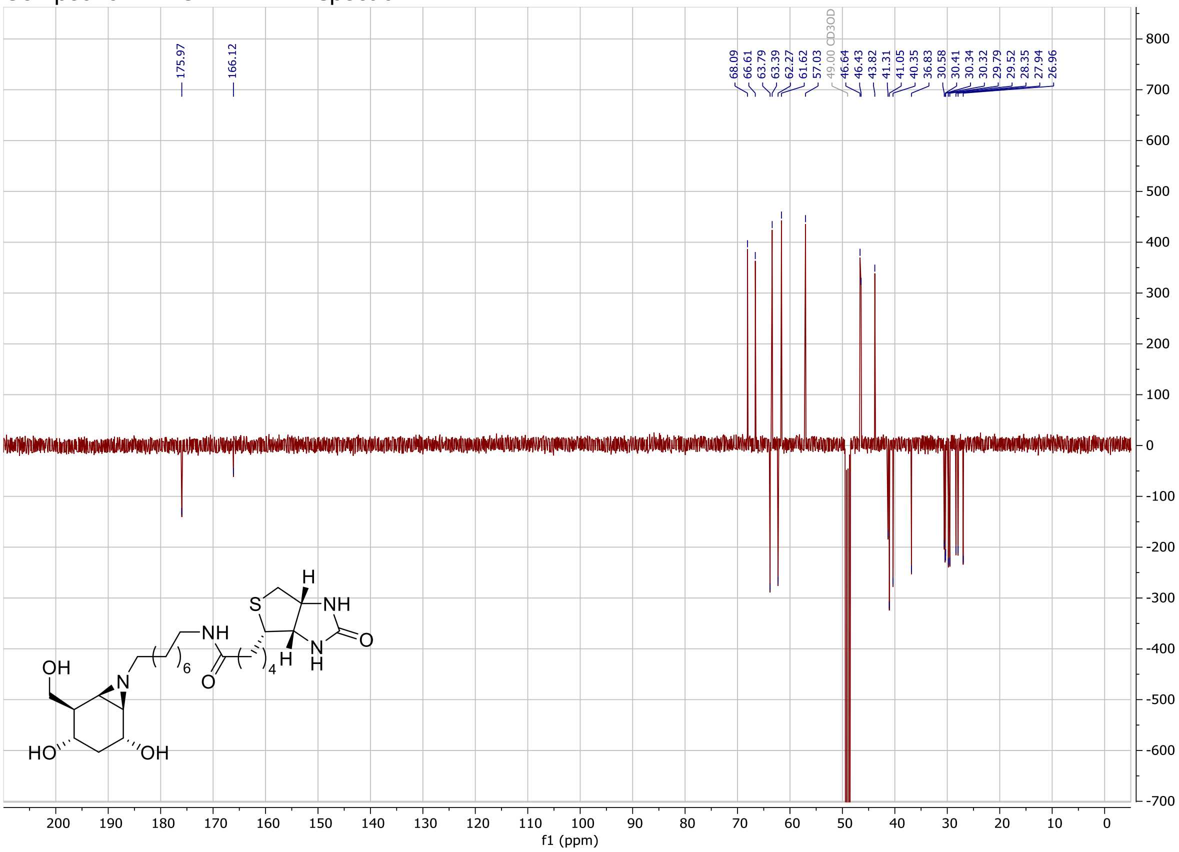

Compound **42** ^{1}H-^{1}H COSY spectrum

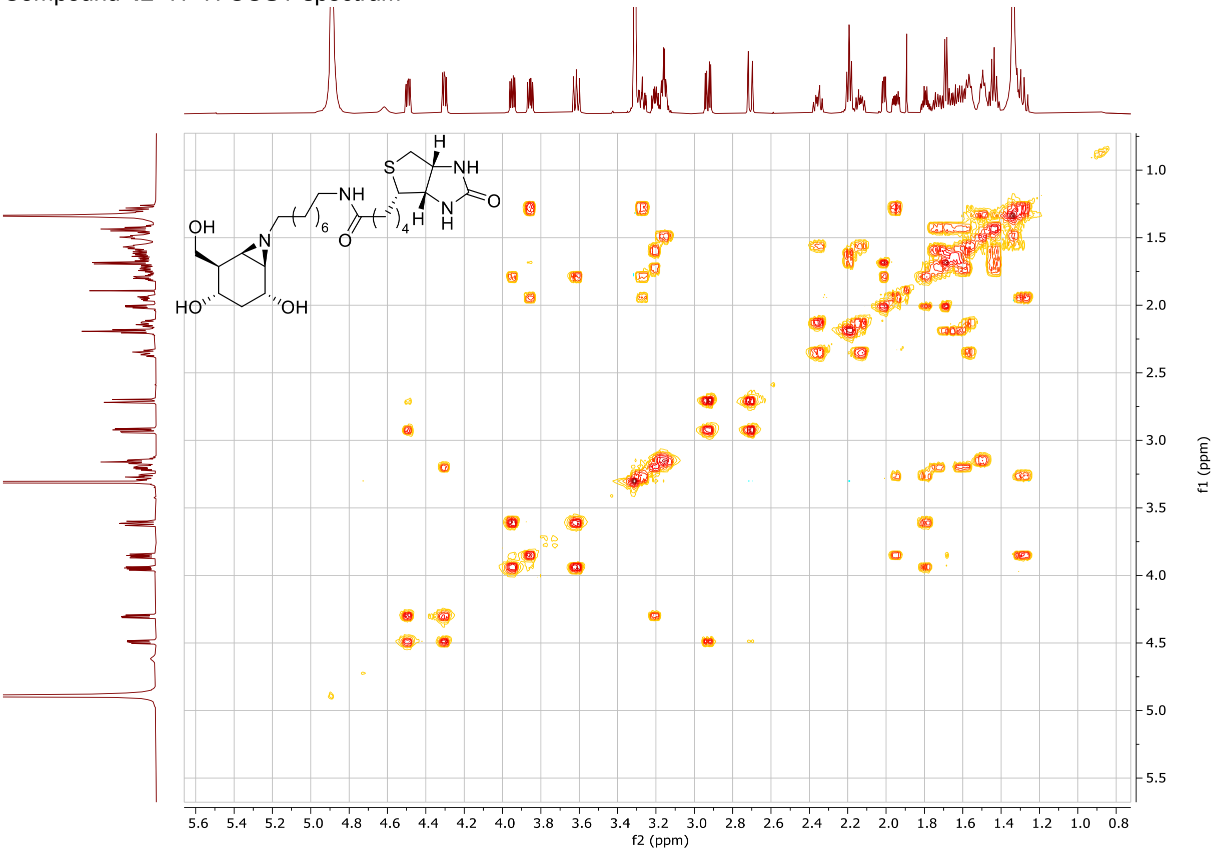

Compound **42** ^{1}H-^{13}C HSQC spectrum

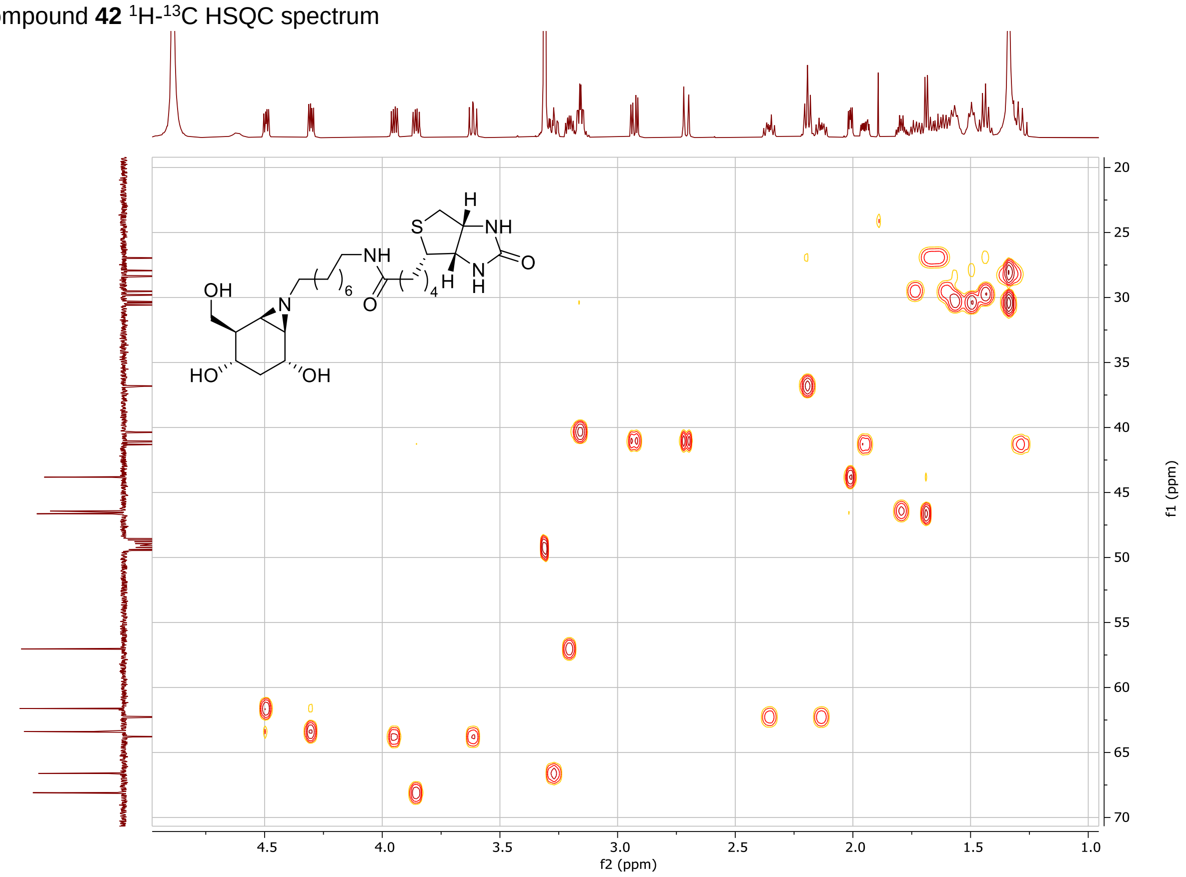

Compound **s61** ^{1}H NMR spectrum

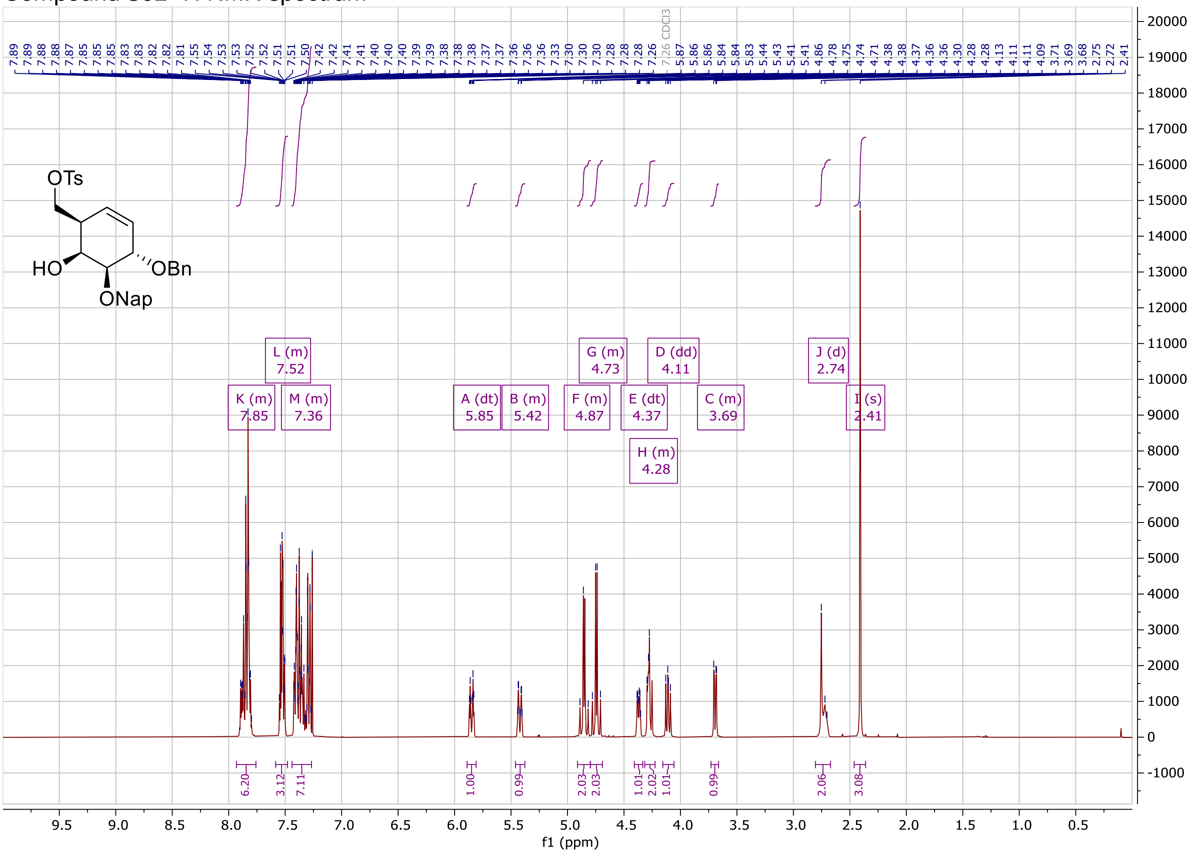

Compound **s61** ^{13}C NMR APT spectrum

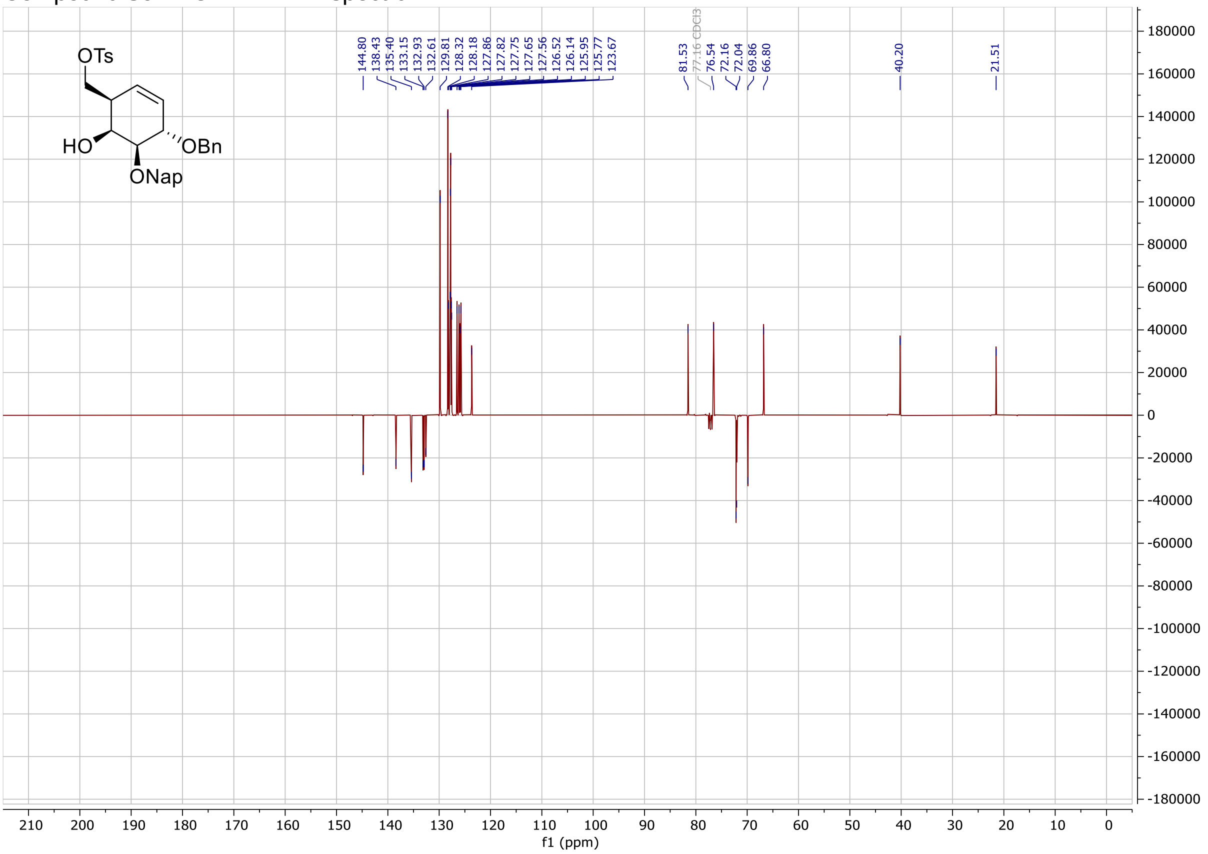

Compound **s62** ^{1}H NMR spectrum

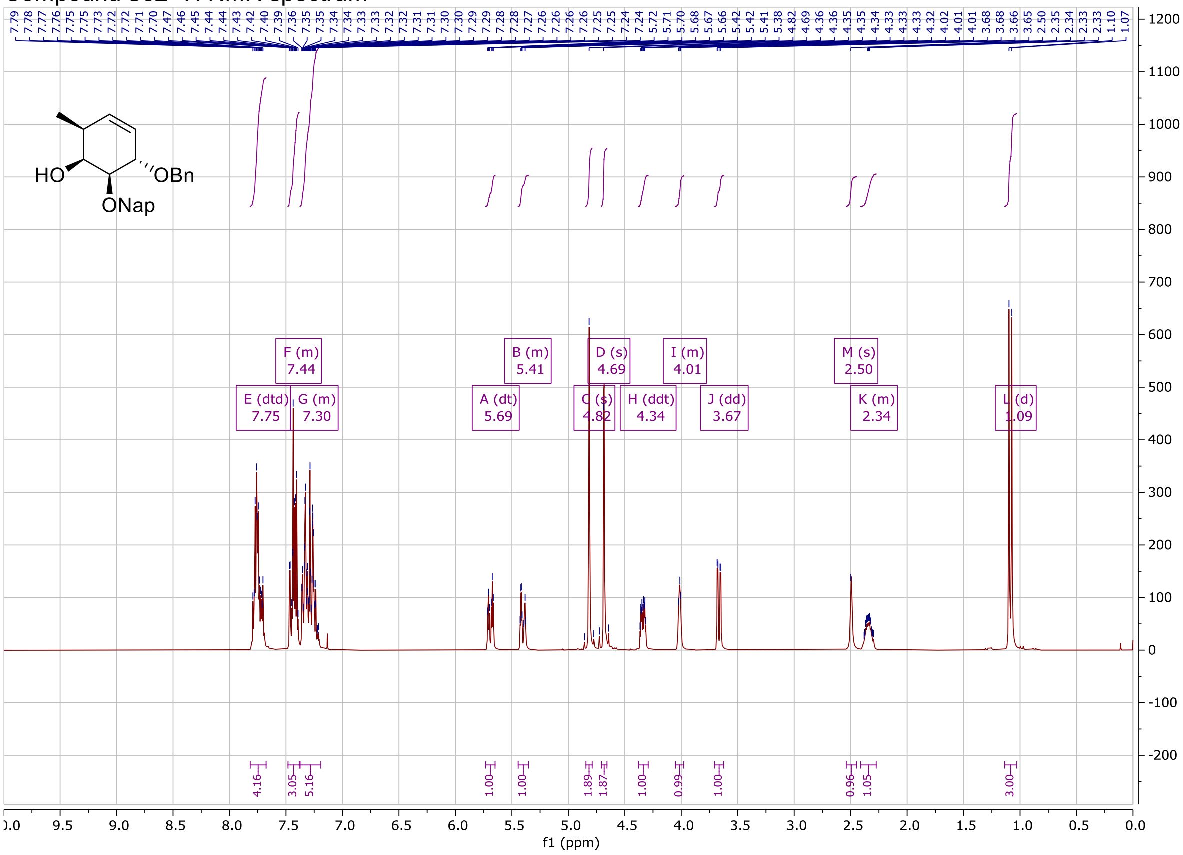

Compound **s62** ^{13}C NMR APT spectrum

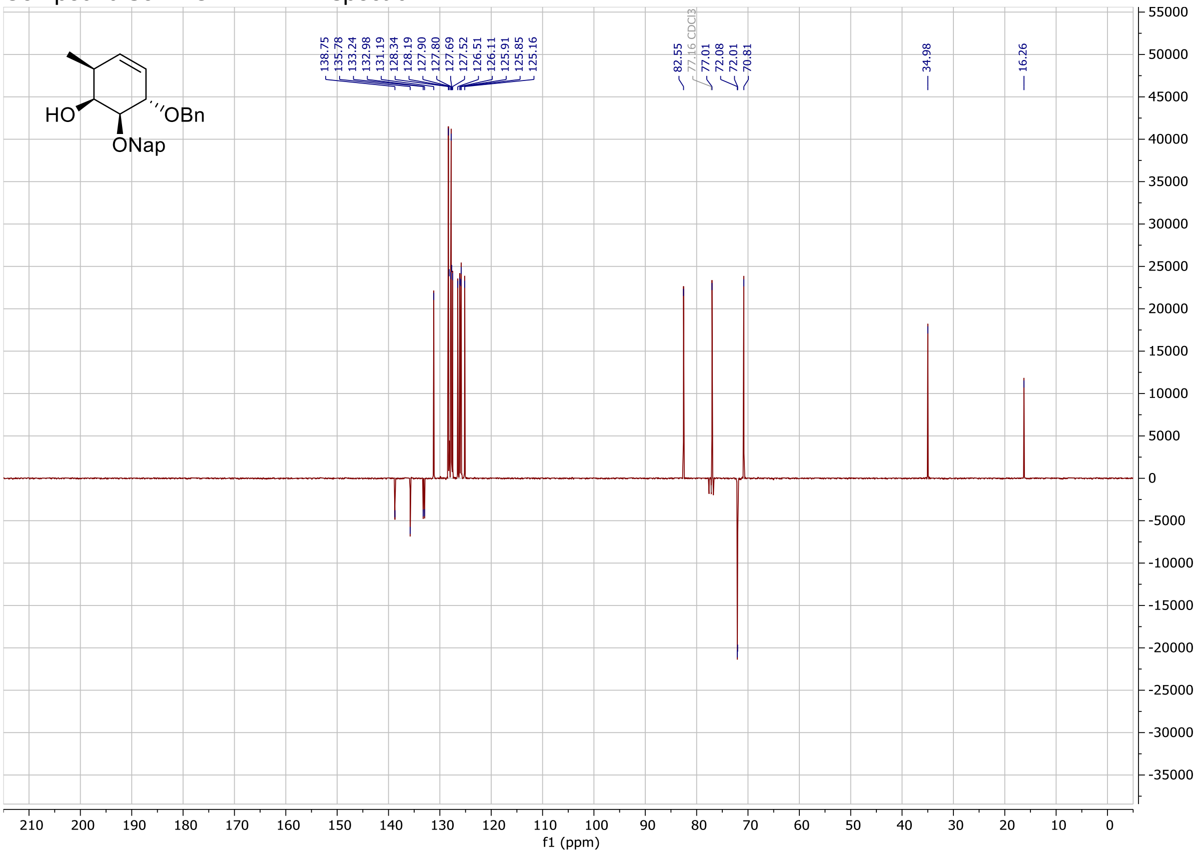

Compound **s63** ^{1}H NMR spectrum

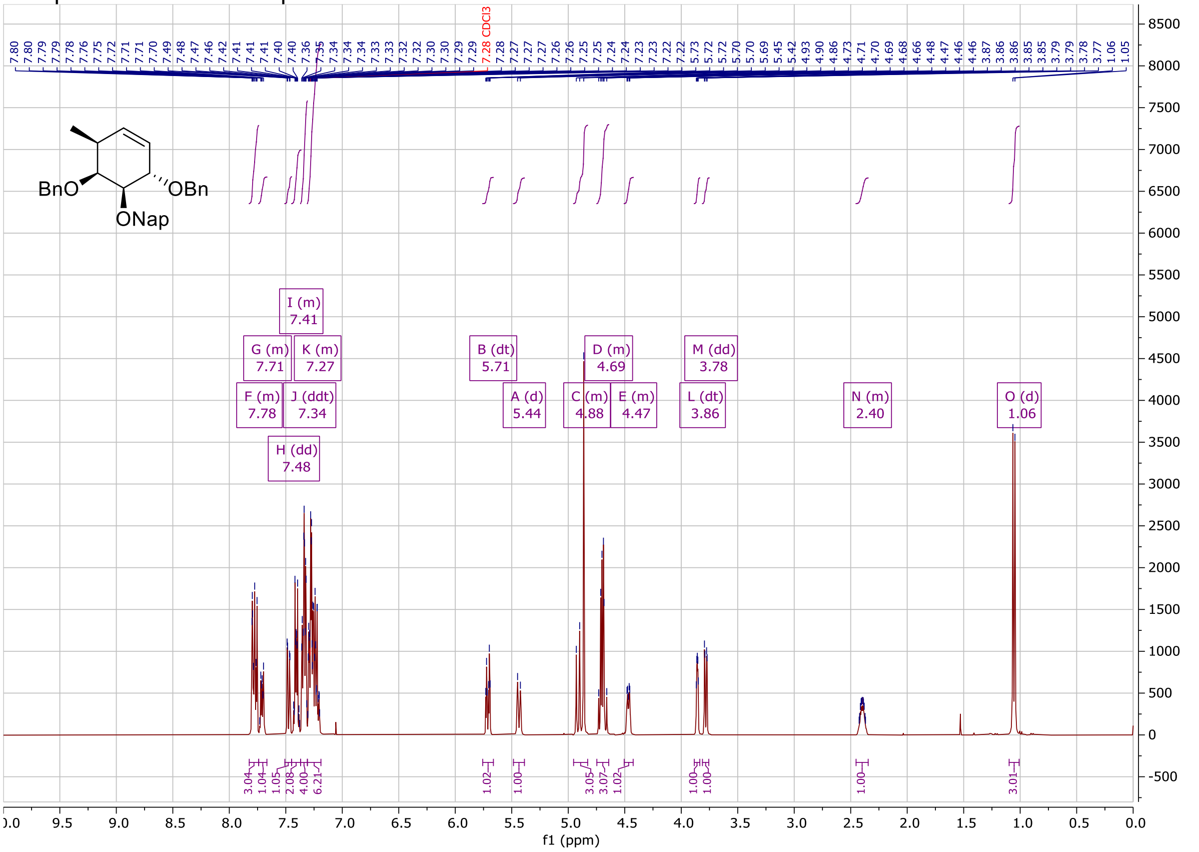

Compound **s63** ^{13}C NMR APT spectrum

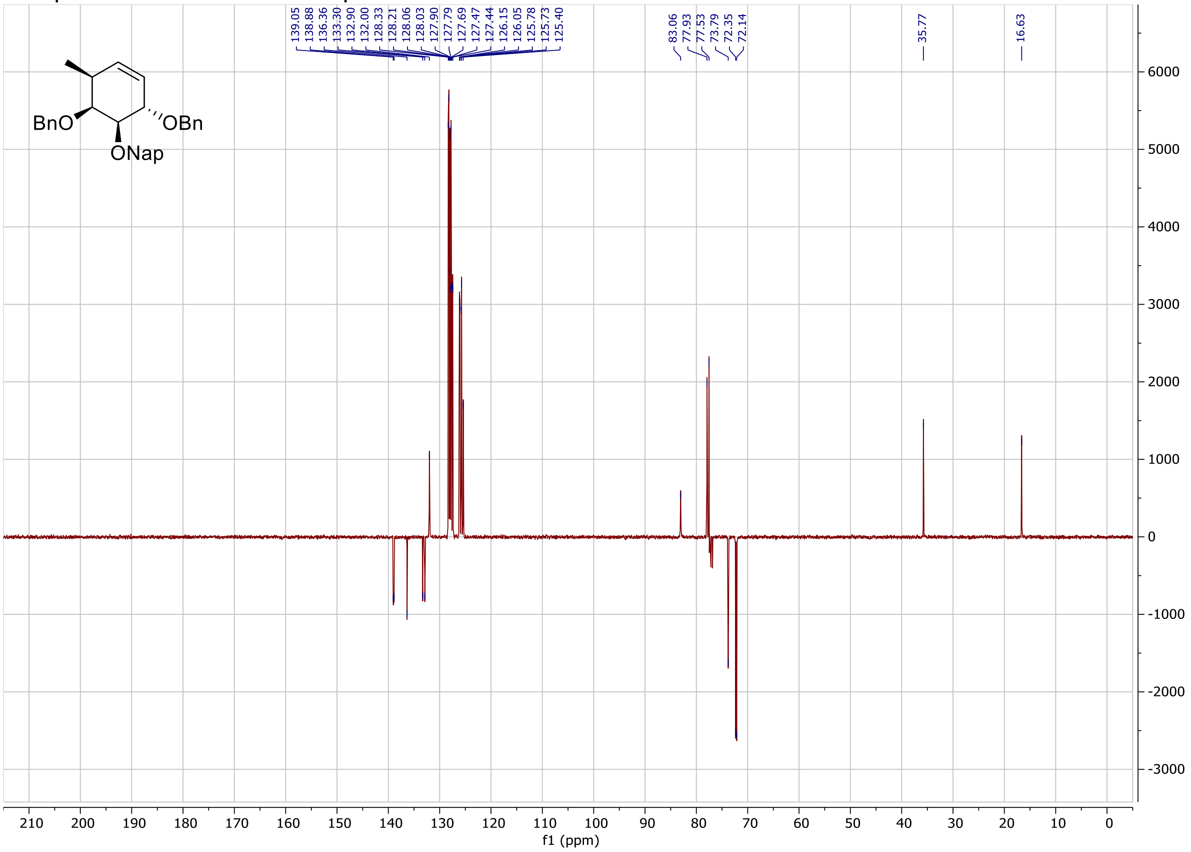

Compound **s64** ^{1}H NMR spectrum

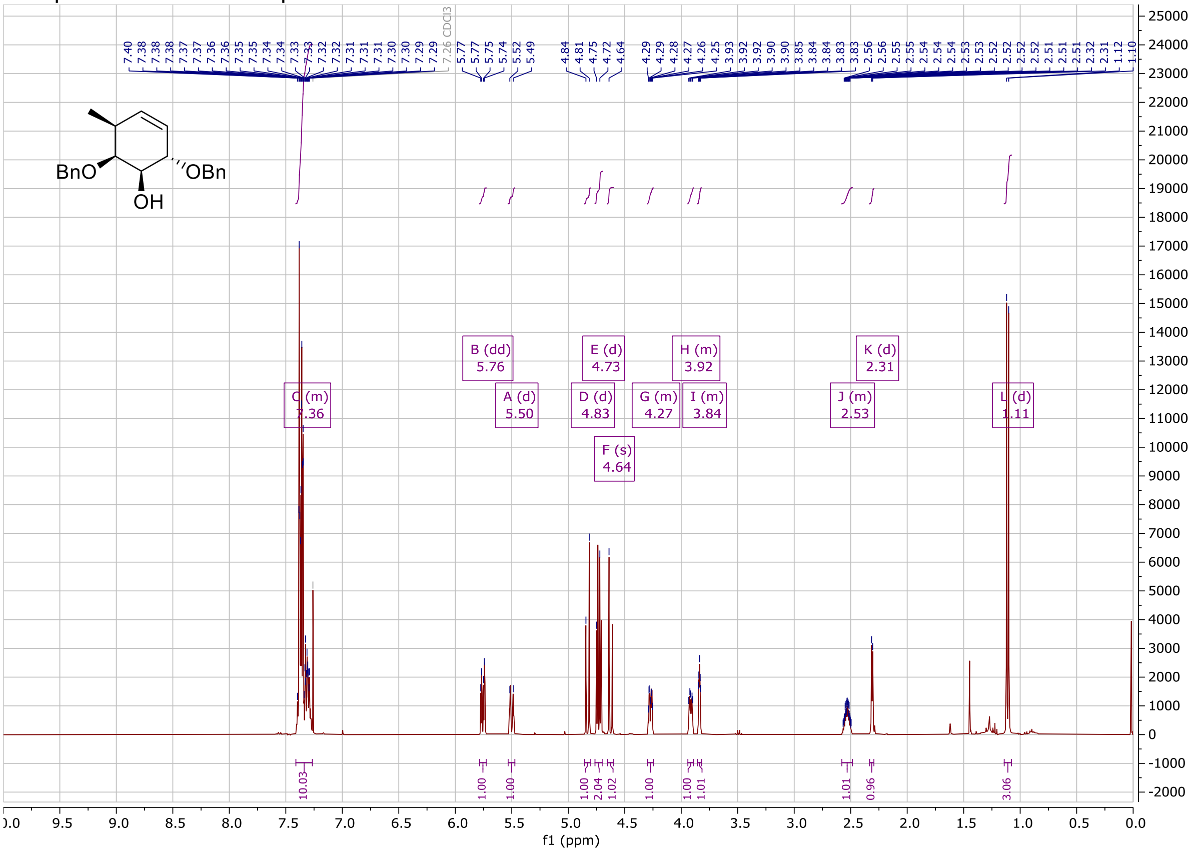

Compound **s64** ^{13}C NMR APT spectrum

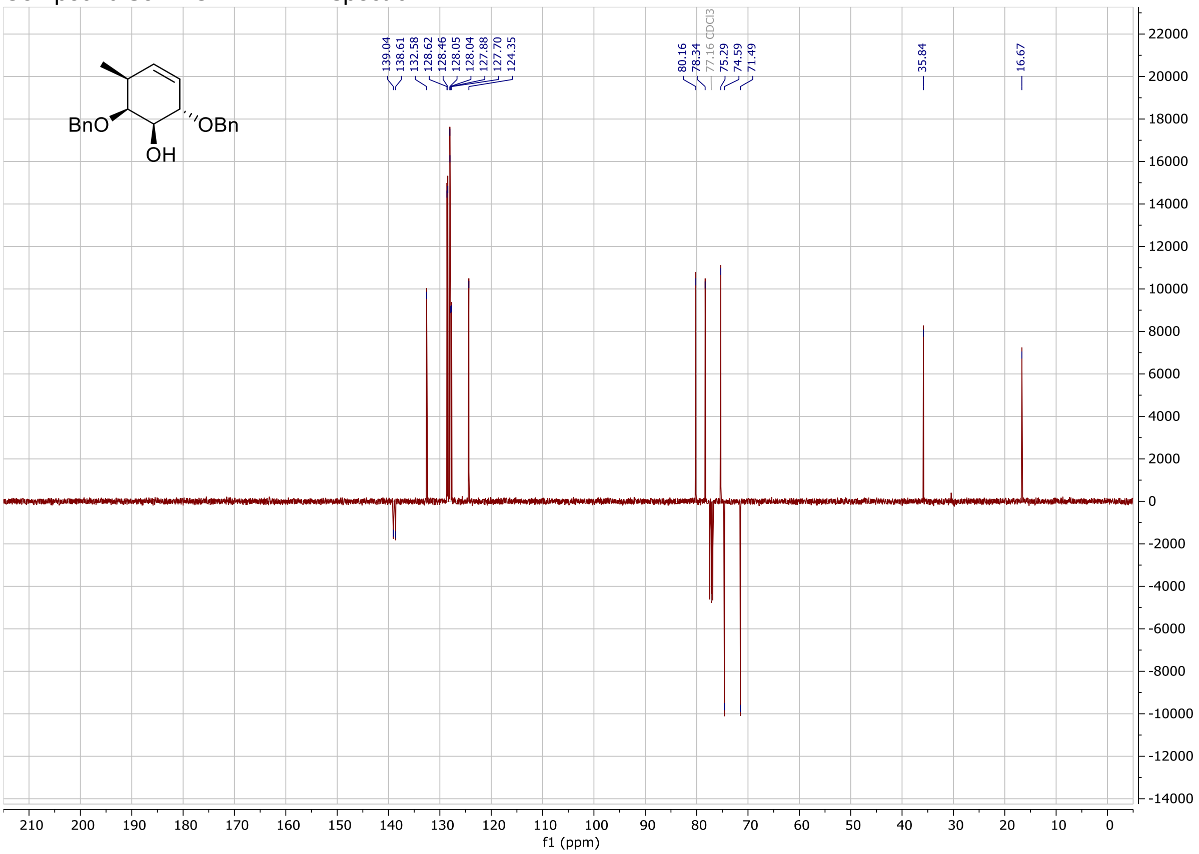

Compound **s65** ^{1}H NMR spectrum

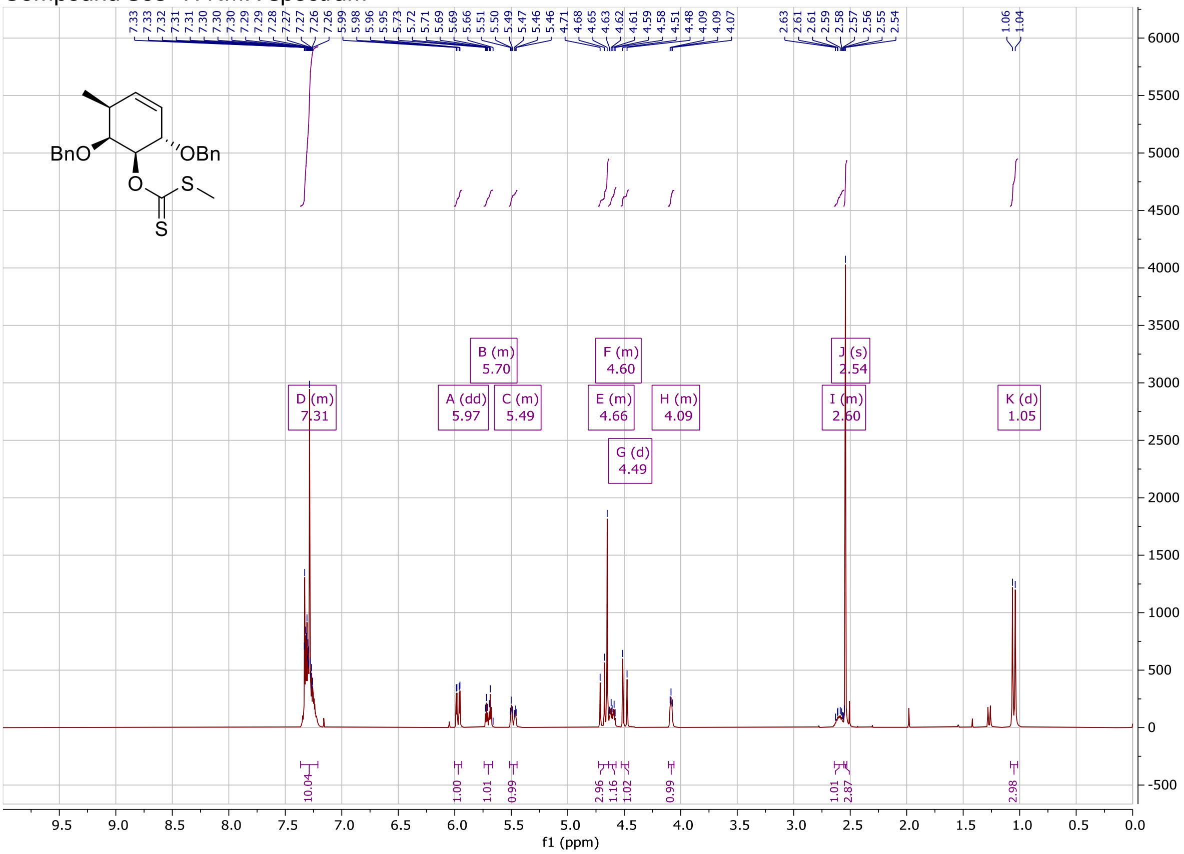

Compound **s65** ^{13}C NMR APT spectrum

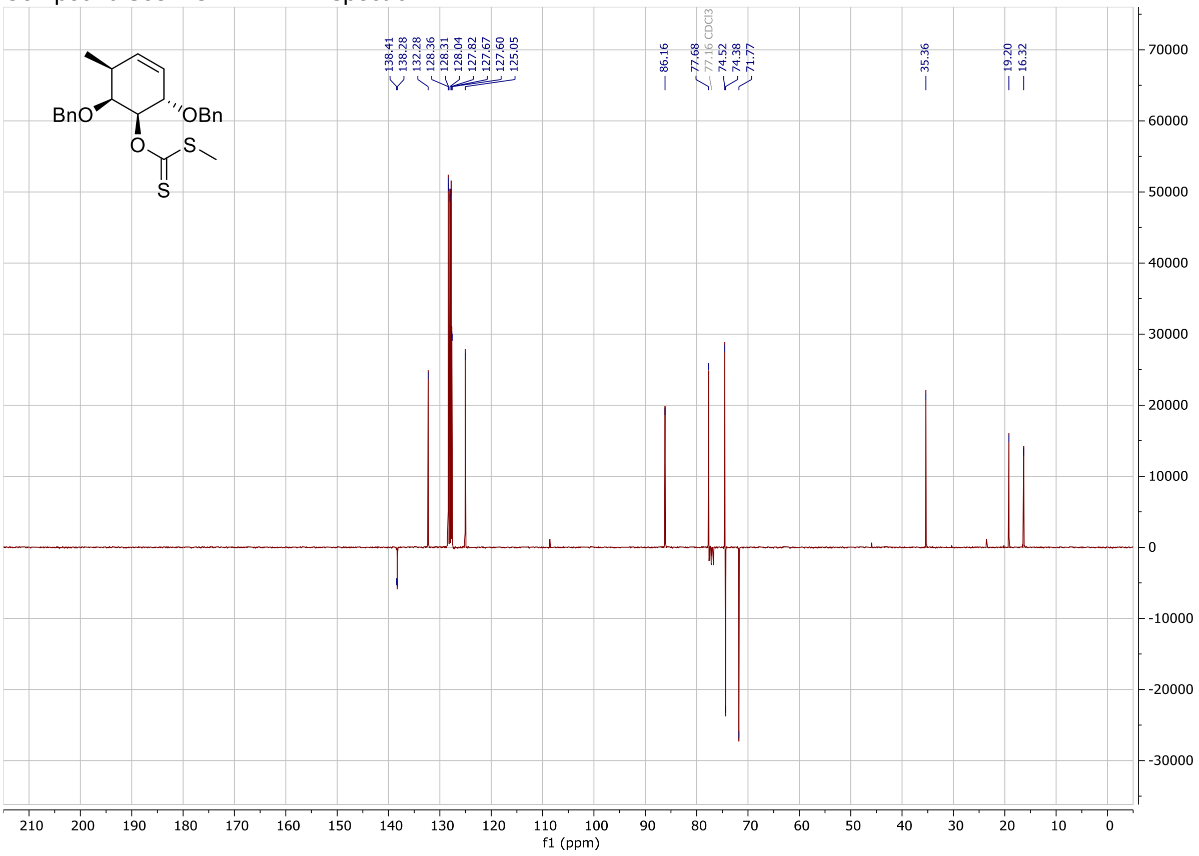

Compound **s66** ^{1}H NMR spectrum

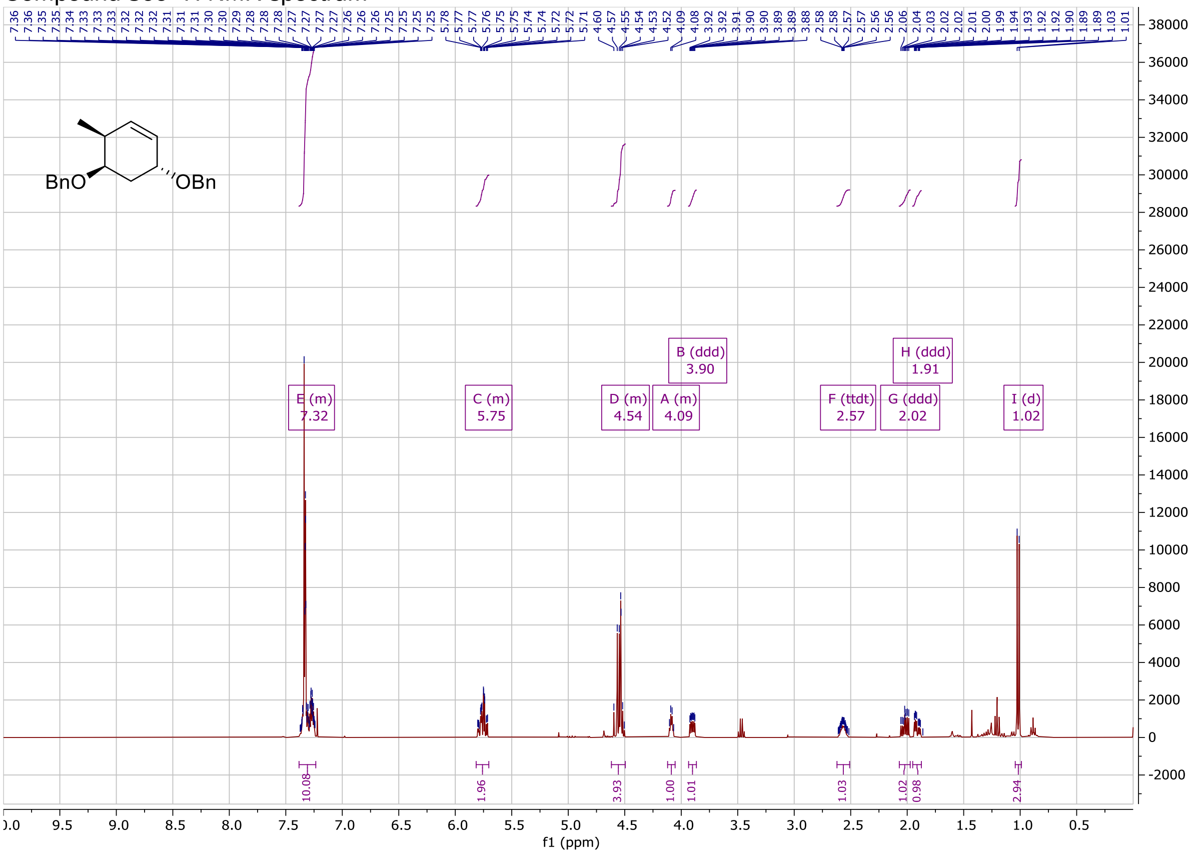

Compound **s66** ^{13}C NMR APT spectrum

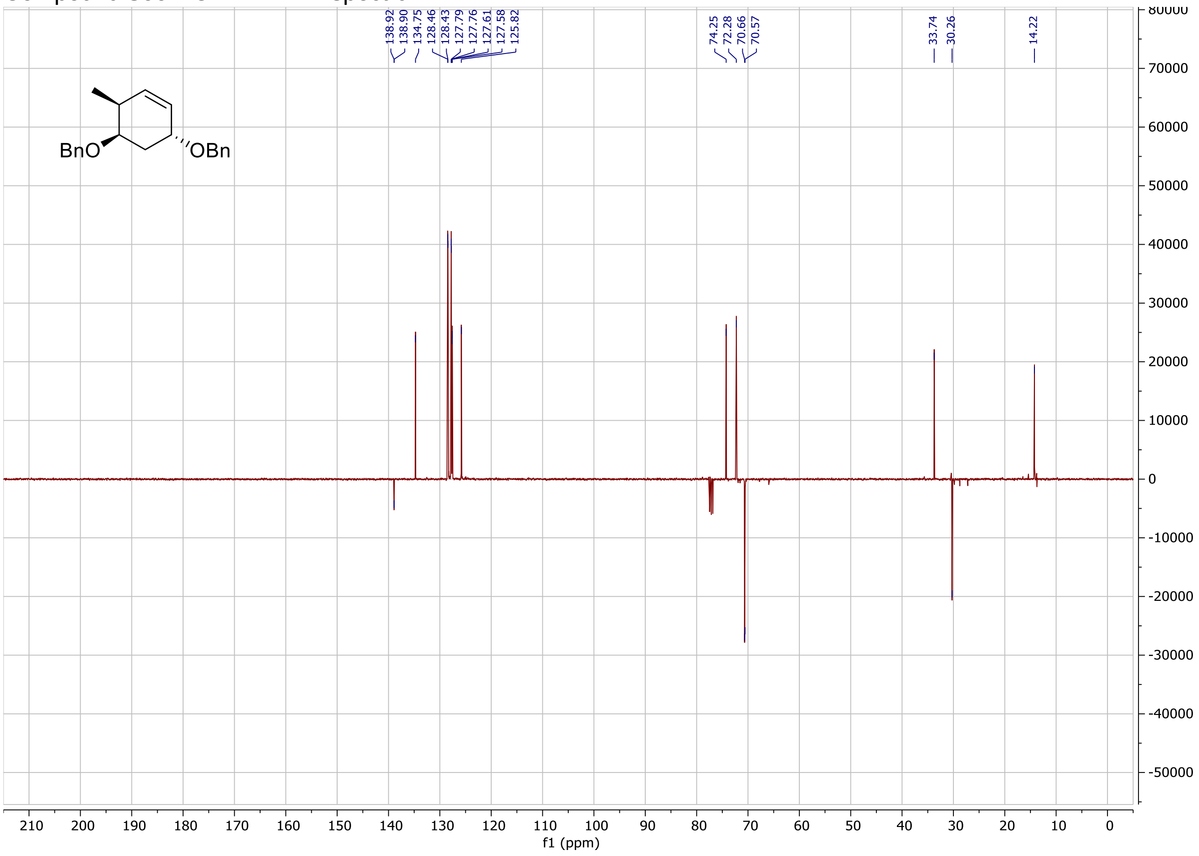

Compound **s67** ^{1}H NMR spectrum

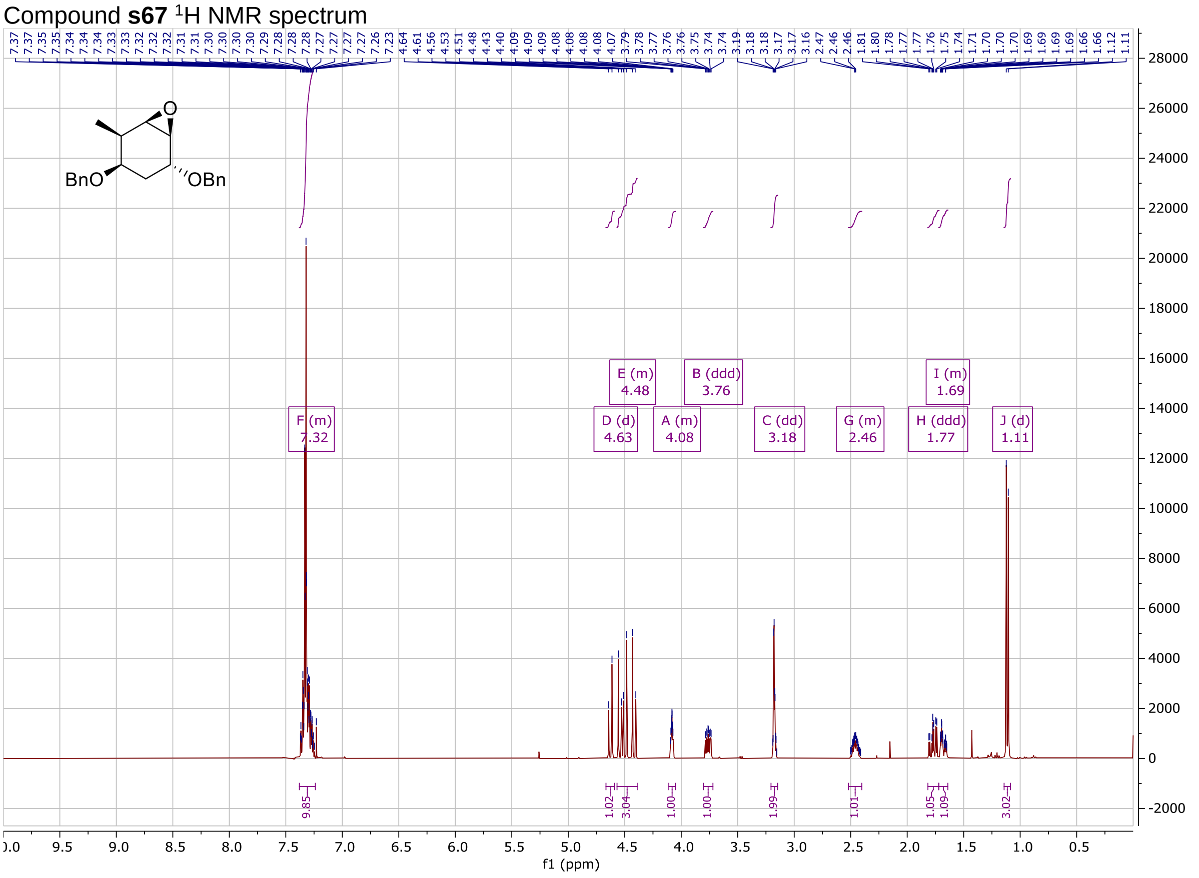

Compound **s67** ^{13}C NMR APT spectrum

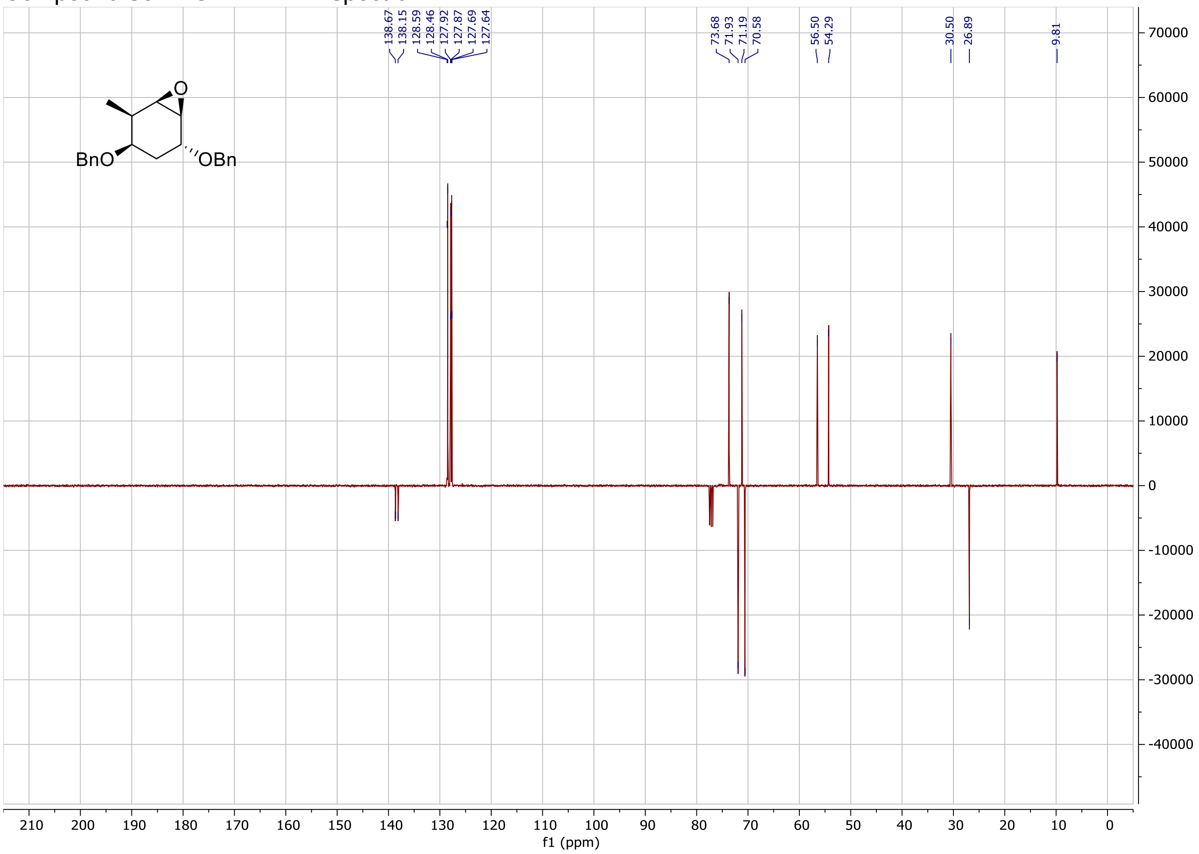

Compound **s68** ^{1}H NMR spectrum

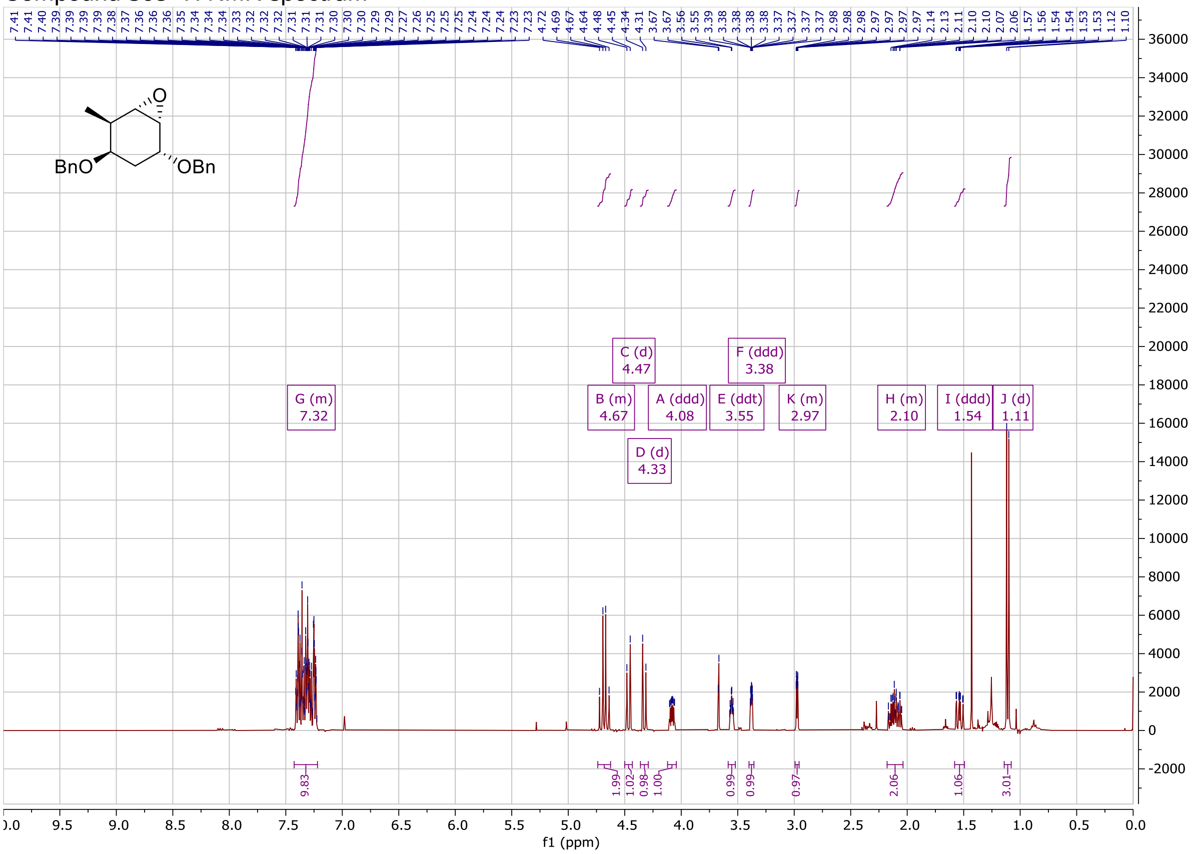

Compound **s68** ^{13}C NMR APT spectrum

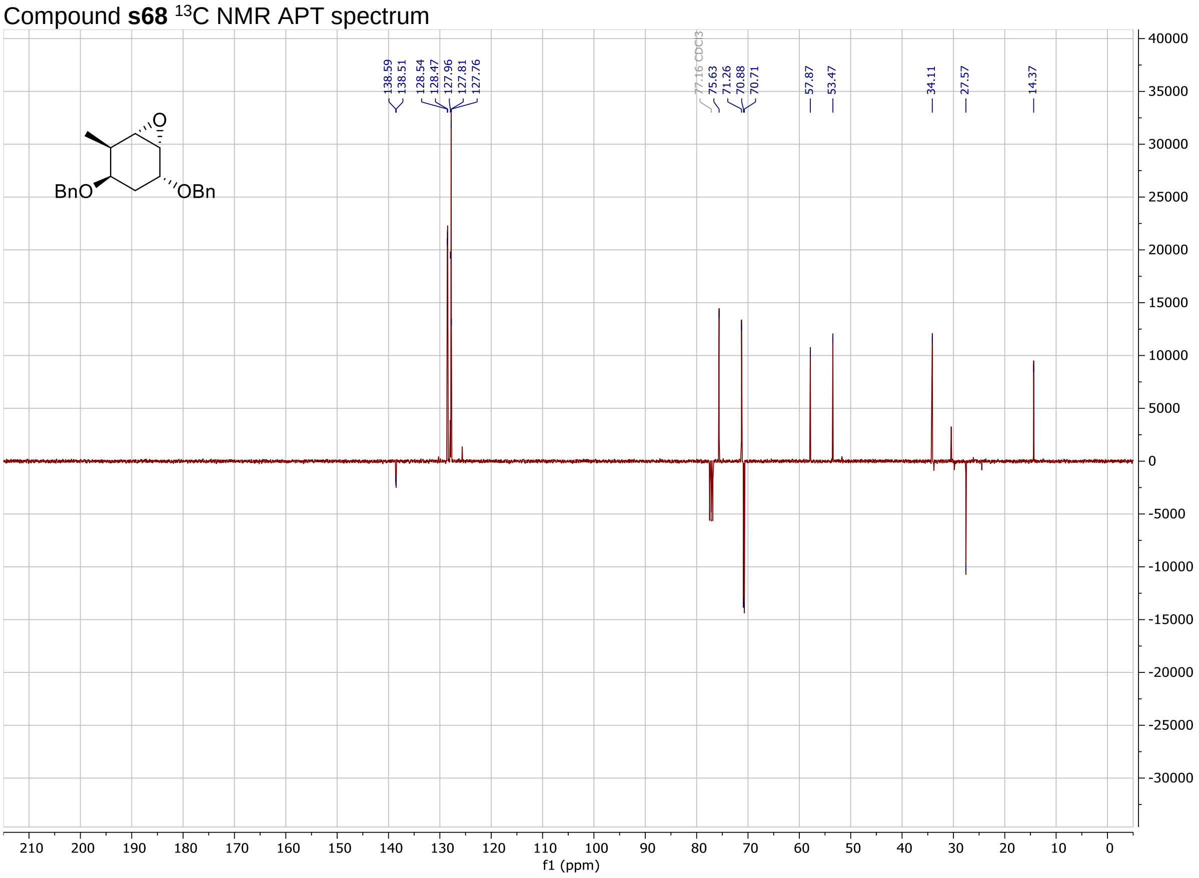

Compound **46** ^{1}H NMR spectrum

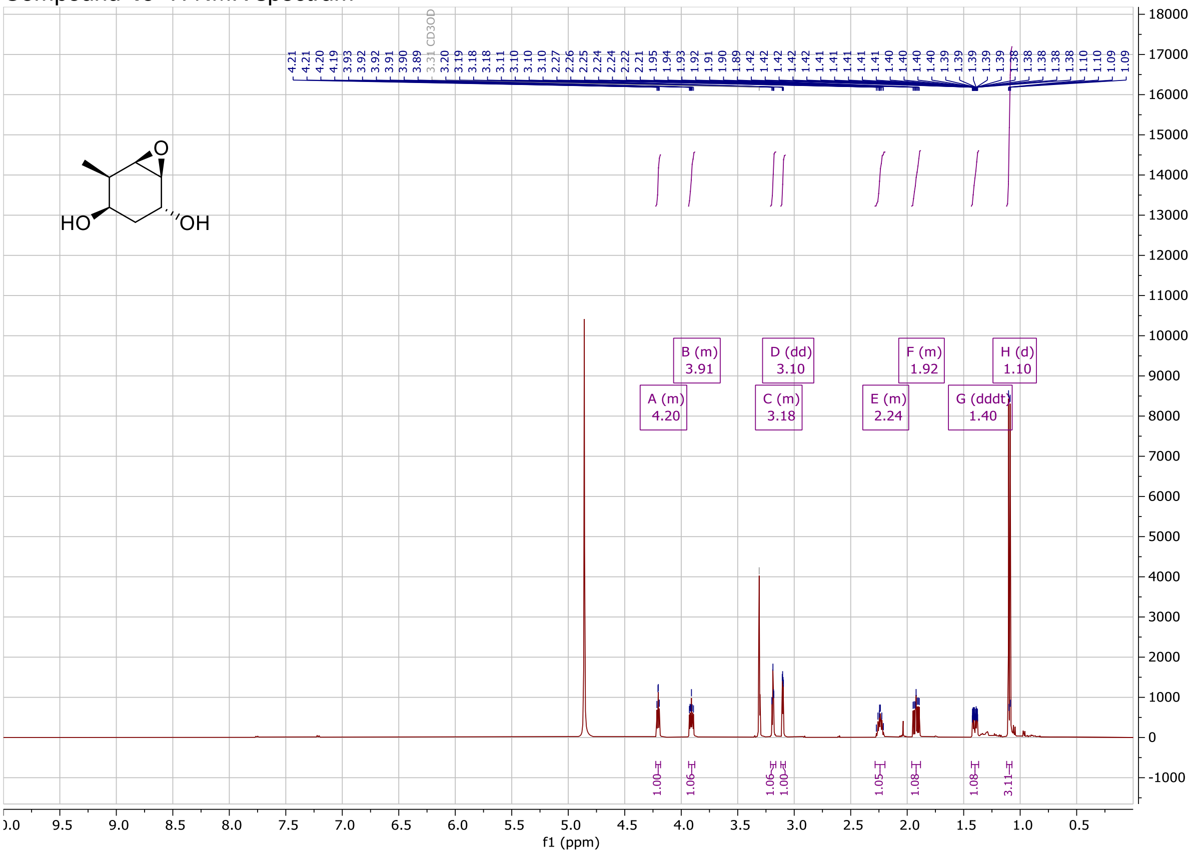

Compound **46** ^{13}C NMR APT spectrum

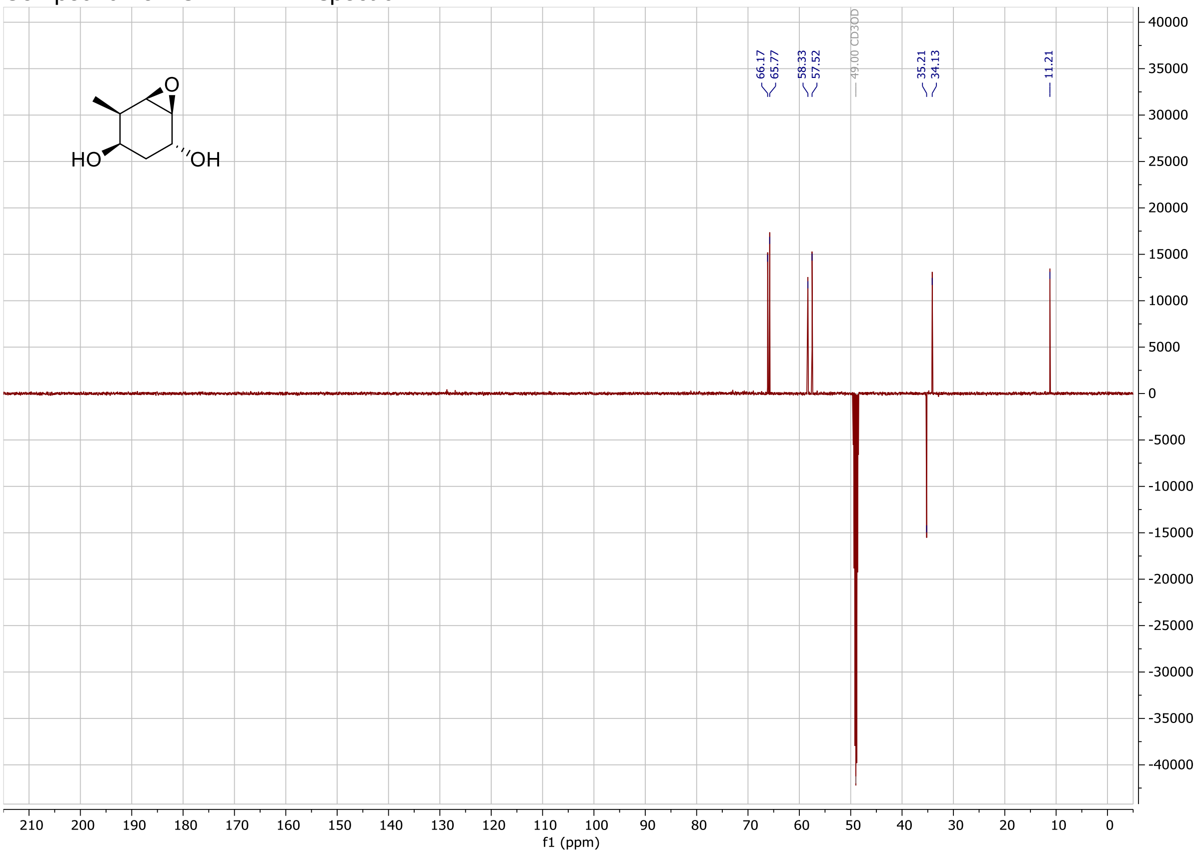

Compound **45** ^{1}H NMR spectrum

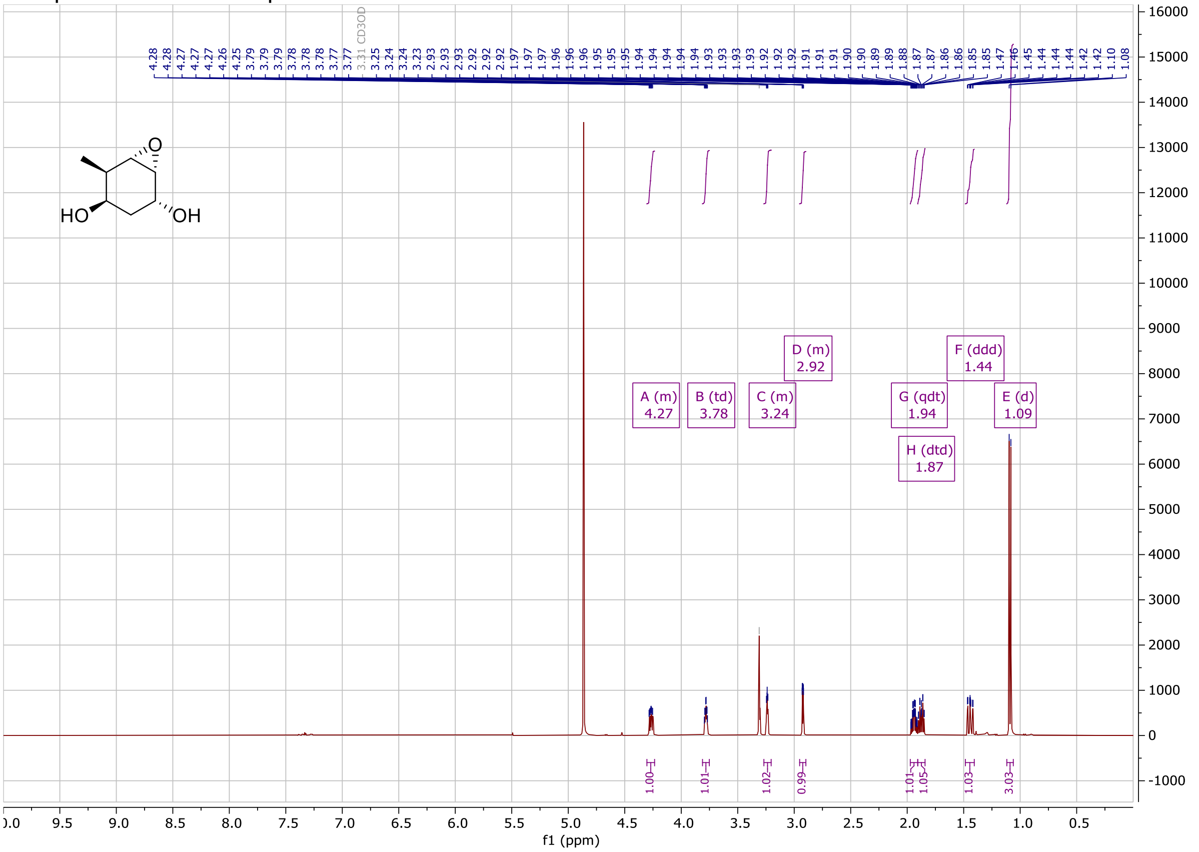

Compound **45** ^{13}C NMR APT spectrum

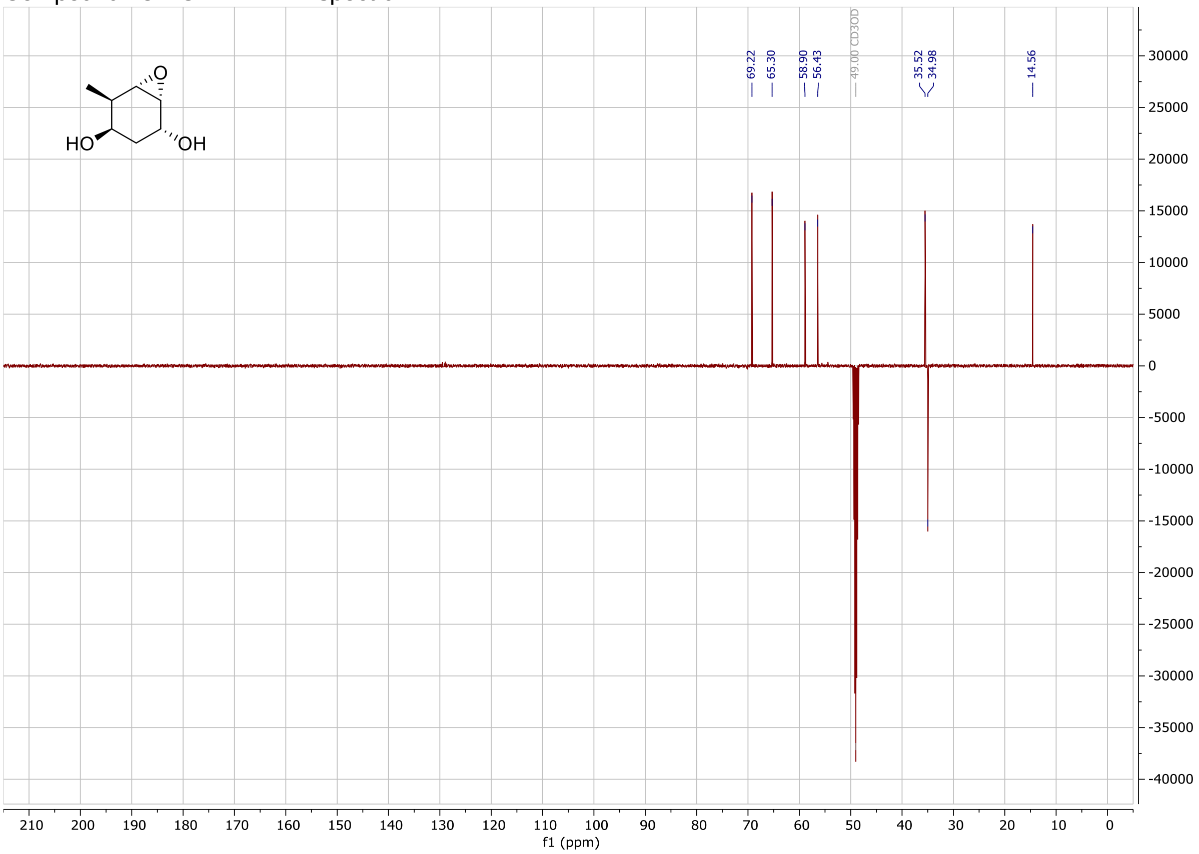

Compound **s69** ^{1}H NMR spectrum

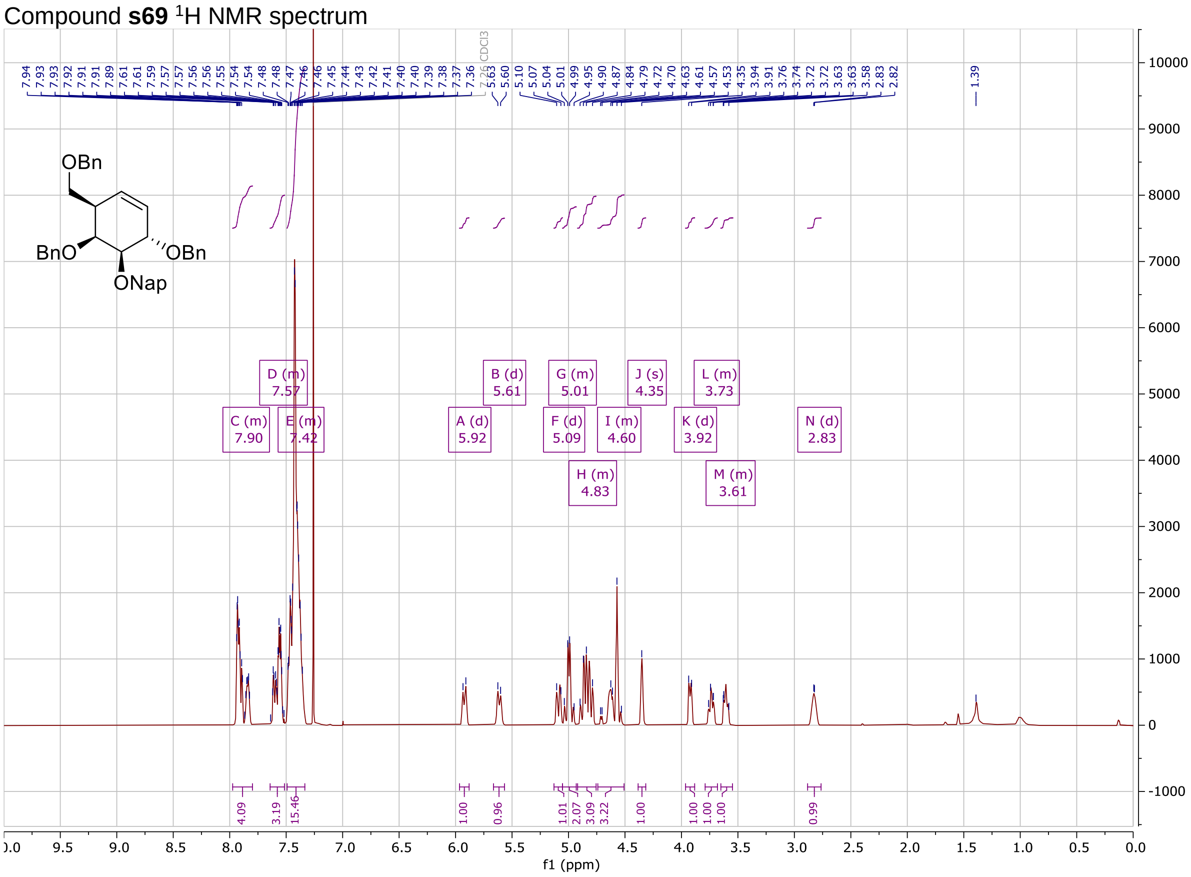

Compound **s69** ^{13}C NMR APT spectrum

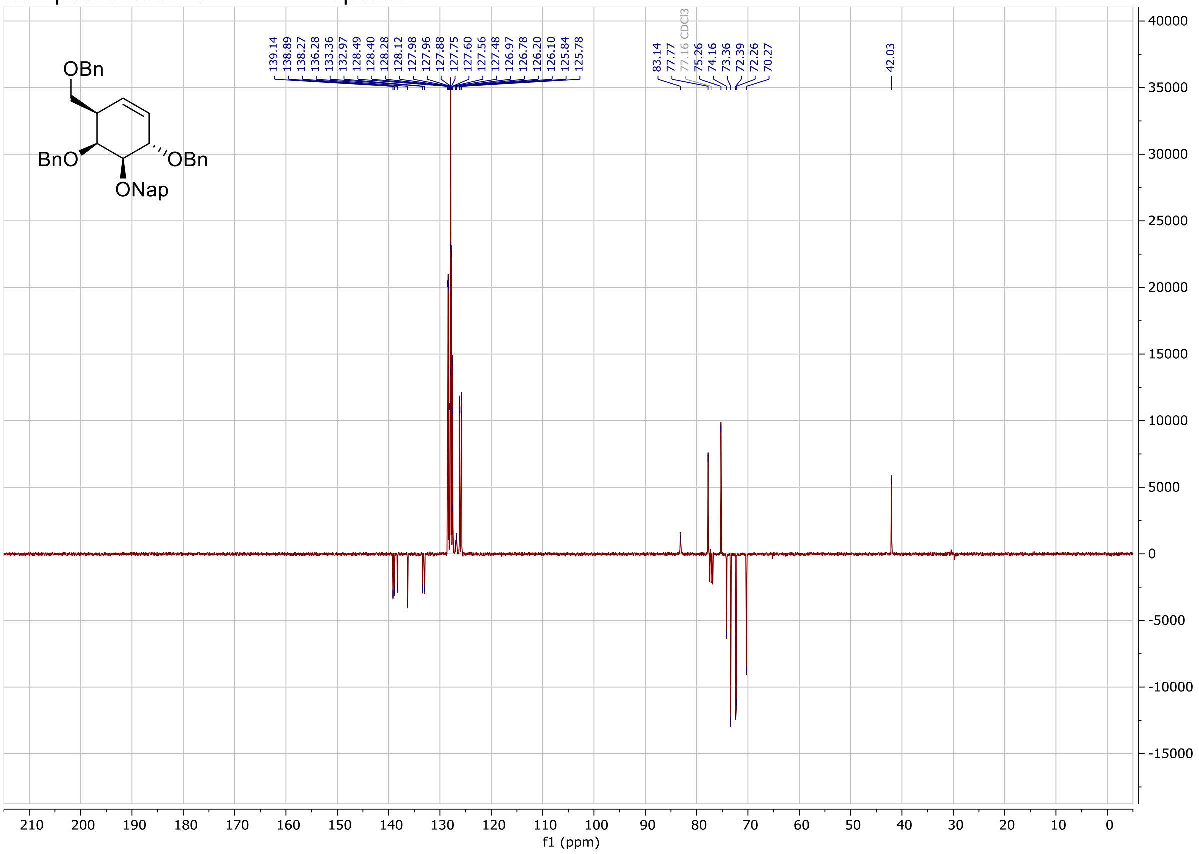

Compound **s69** ^{1}H-^{1}H COSY spectrum

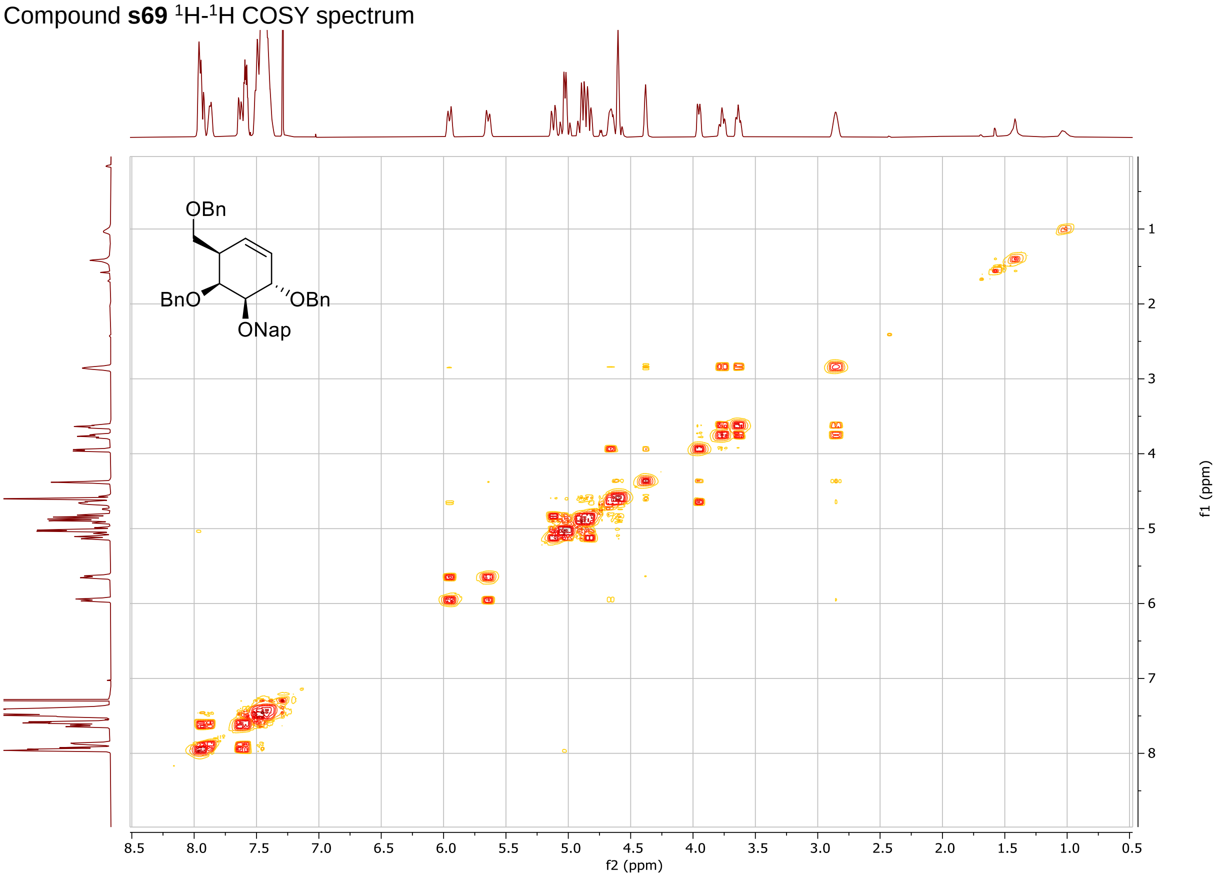

Compound **s69** ^{1}H-^{13}C HSQC spectrum

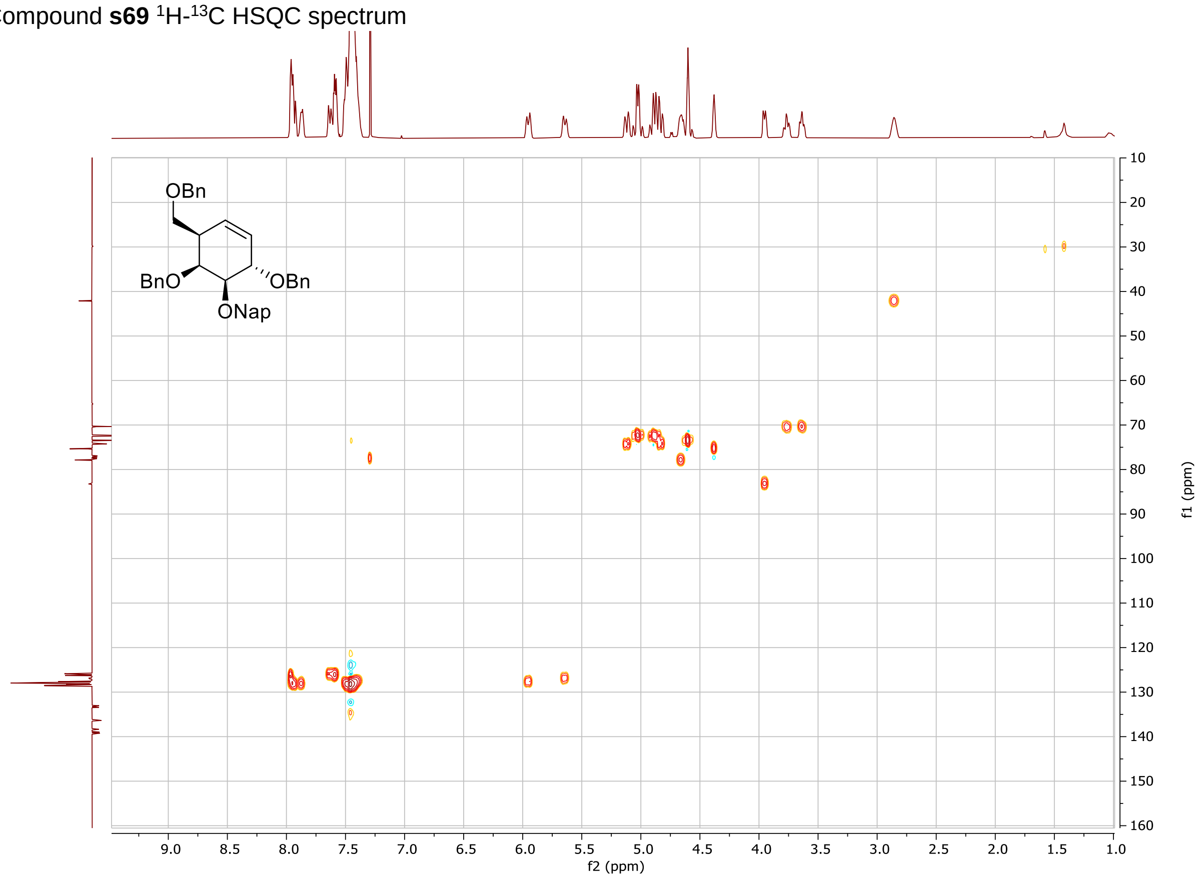

Compound **s70** ^{1}H NMR spectrum

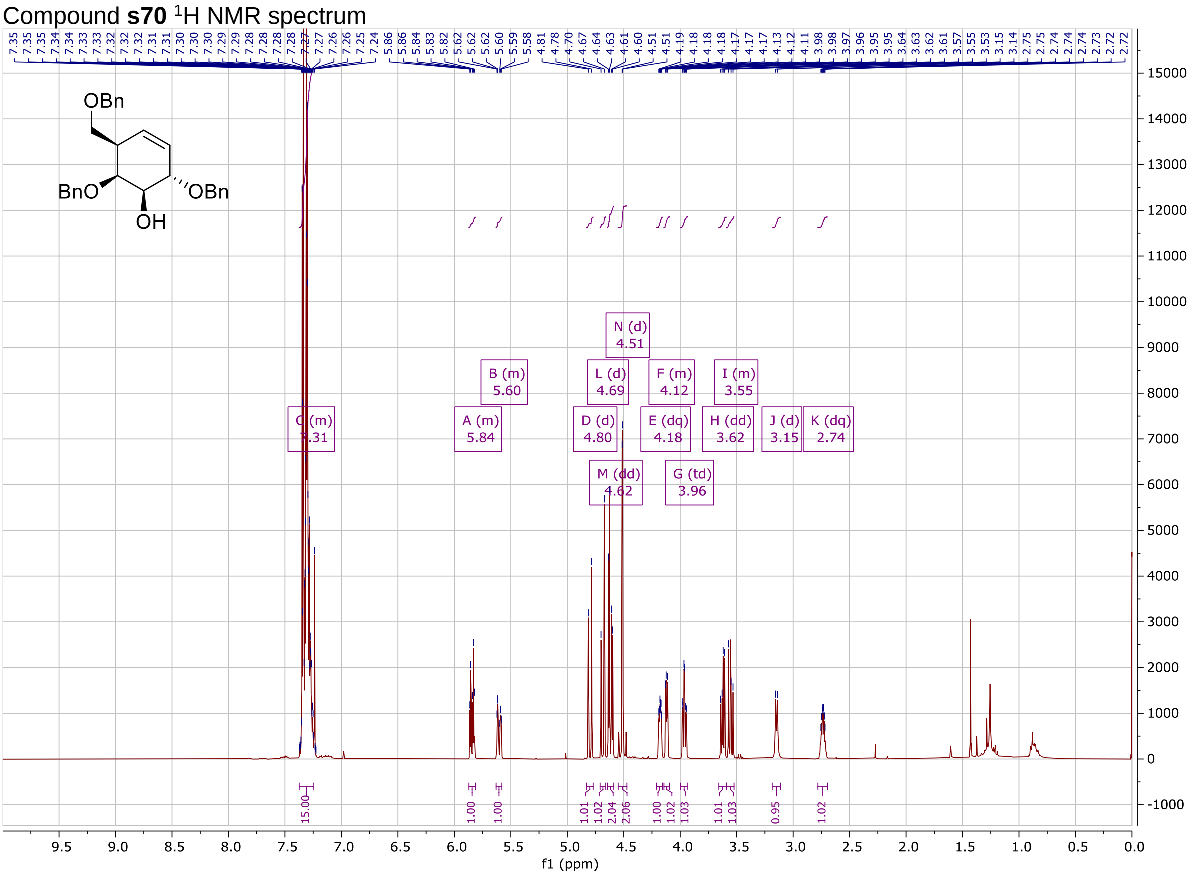

Compound **s70** ^{13}C NMR APT spectrum

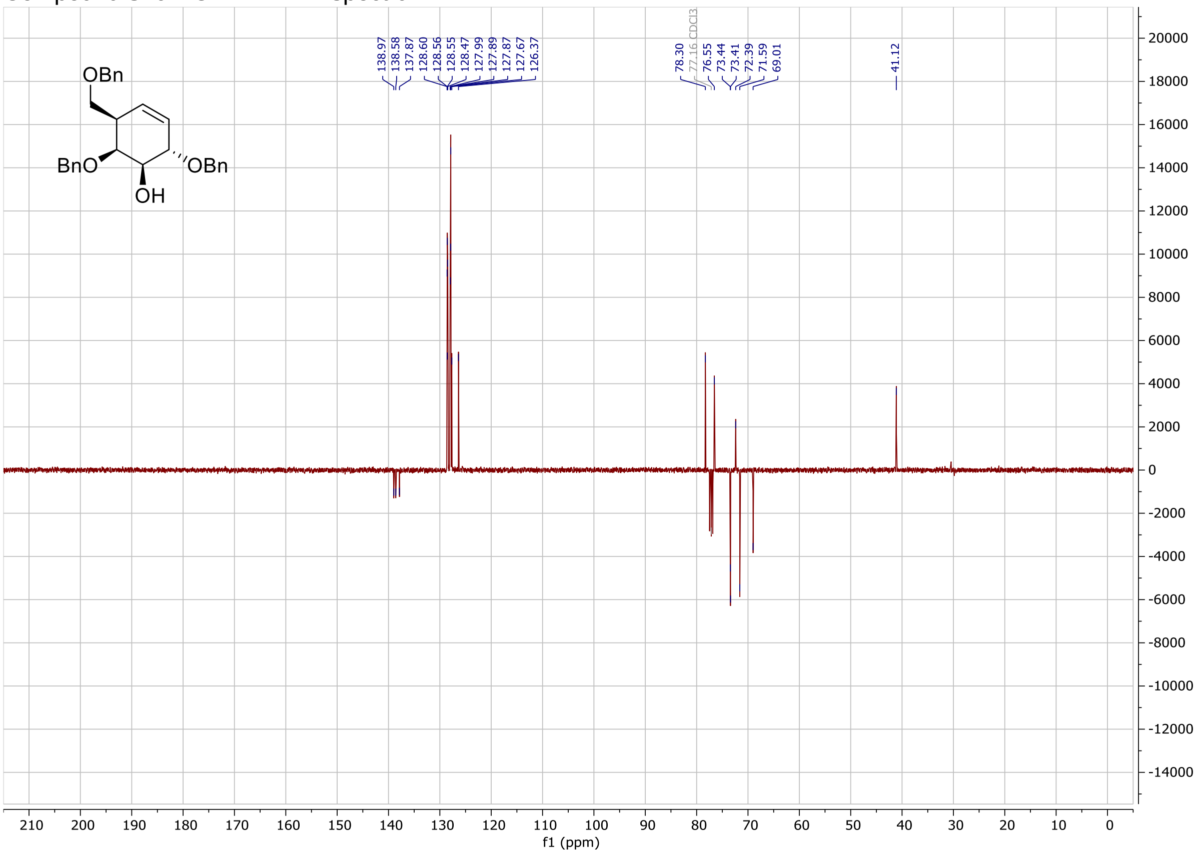

Compound **s70** ^{1}H-^{1}H COSY spectrum

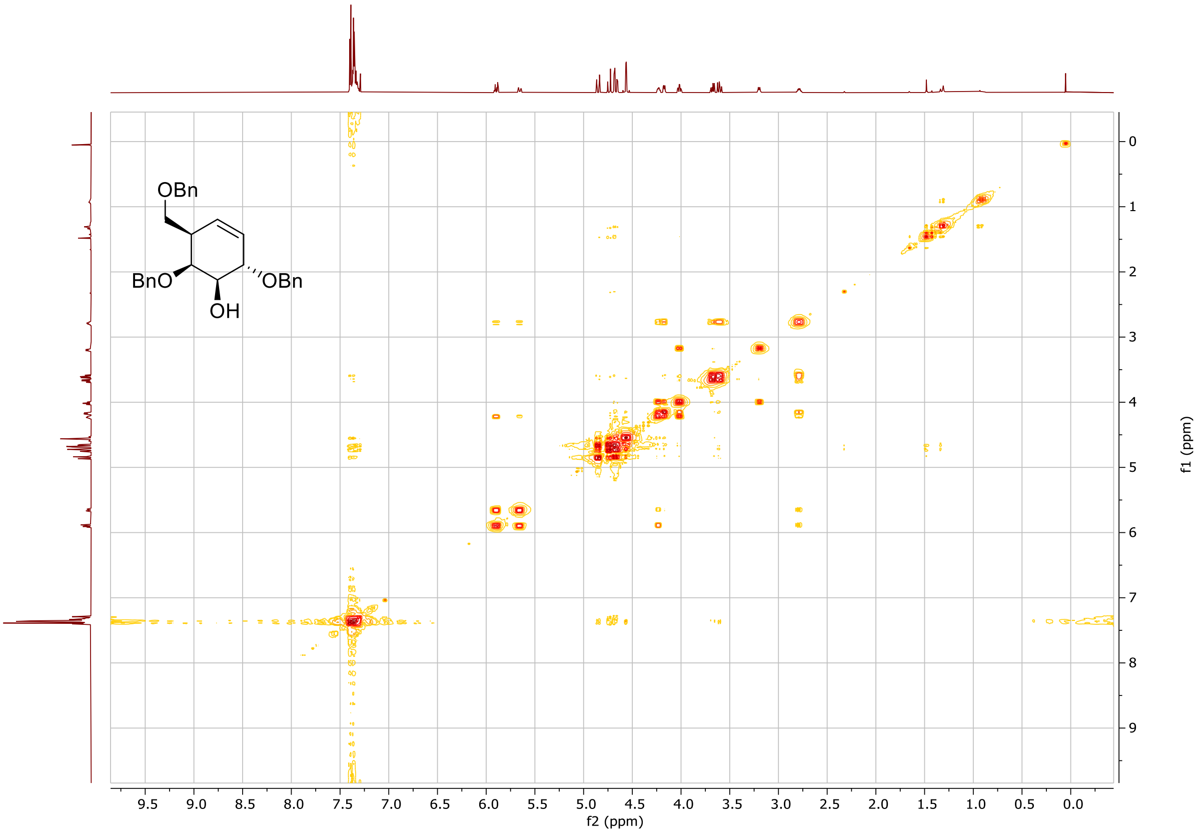

Compound **s70** ^{1}H-^{13}C HSQC spectrum

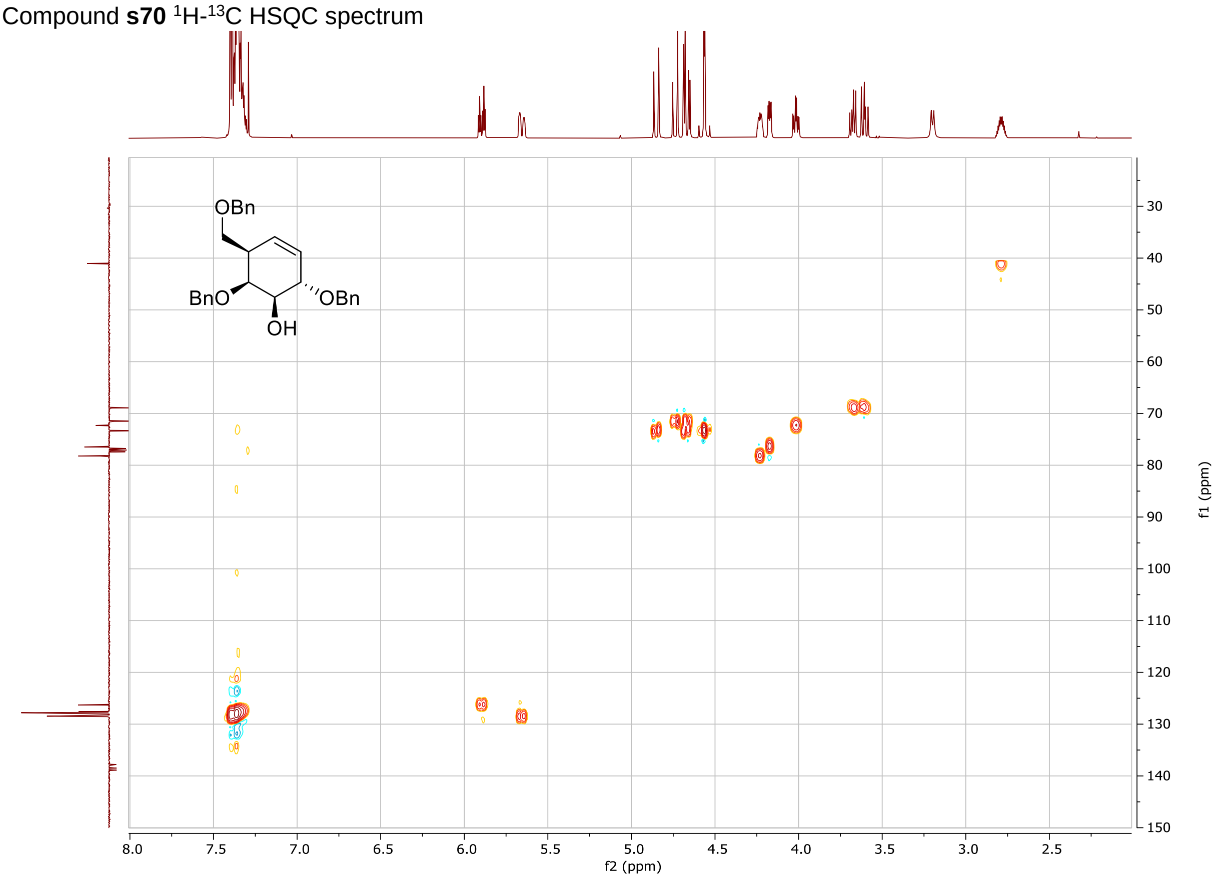

Compound **s71** ^{1}H NMR spectrum

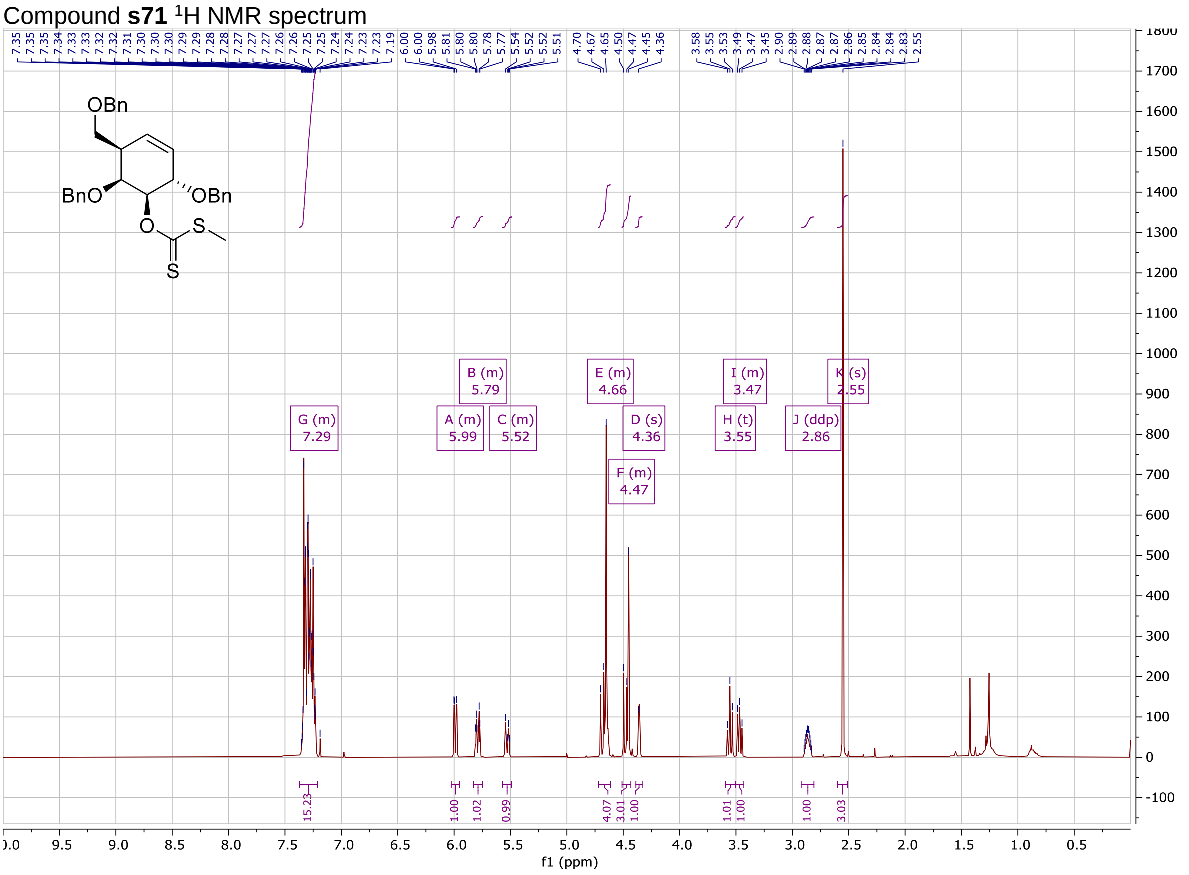

Compound **s71** ^{13}C NMR APT spectrum

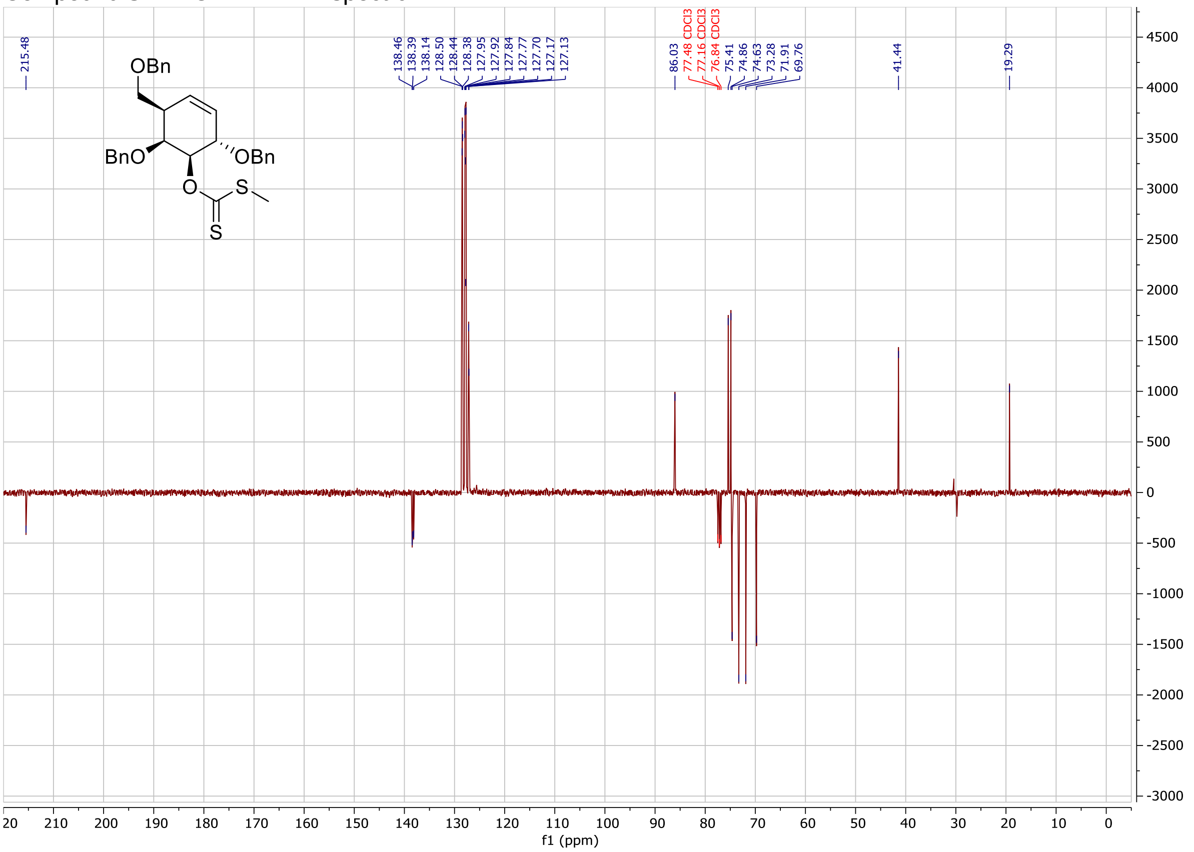

Compound **s72** ^{1}H NMR spectrum

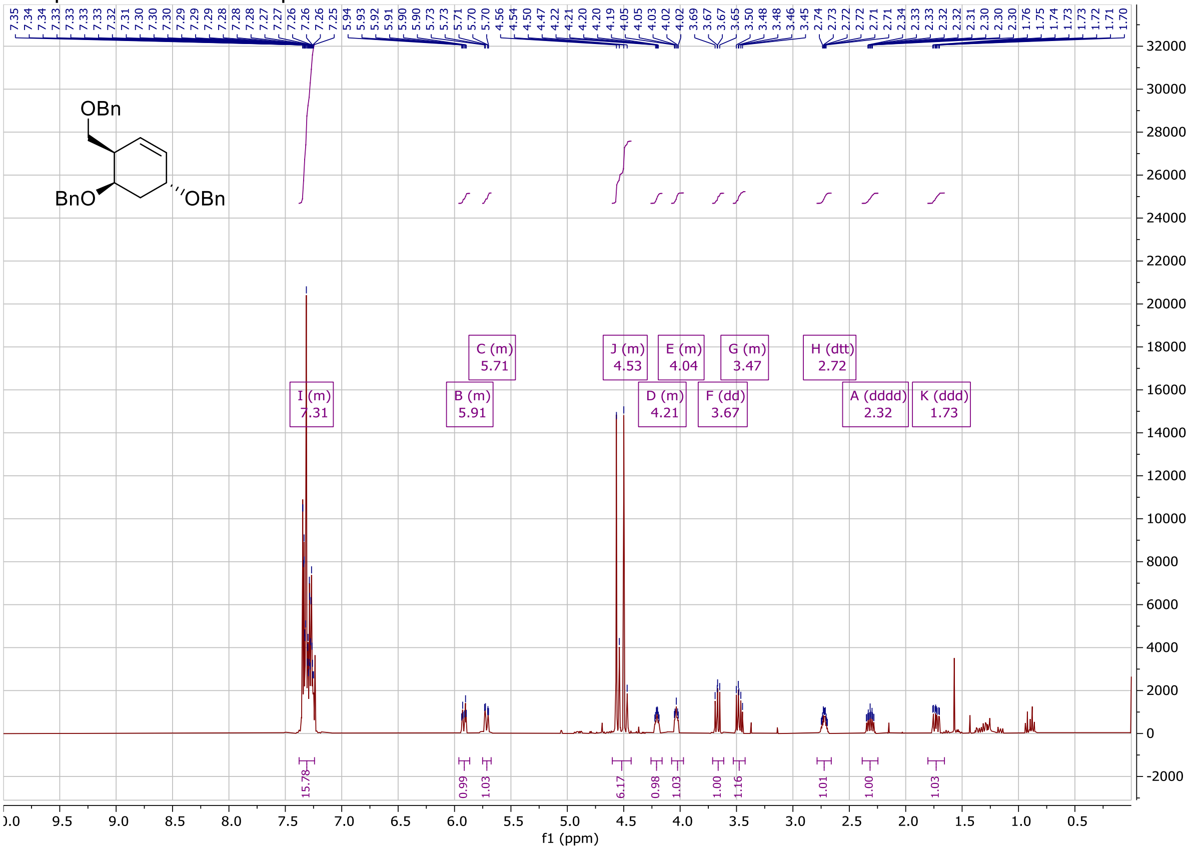

Compound **s72** ^{13}C NMR APT spectrum

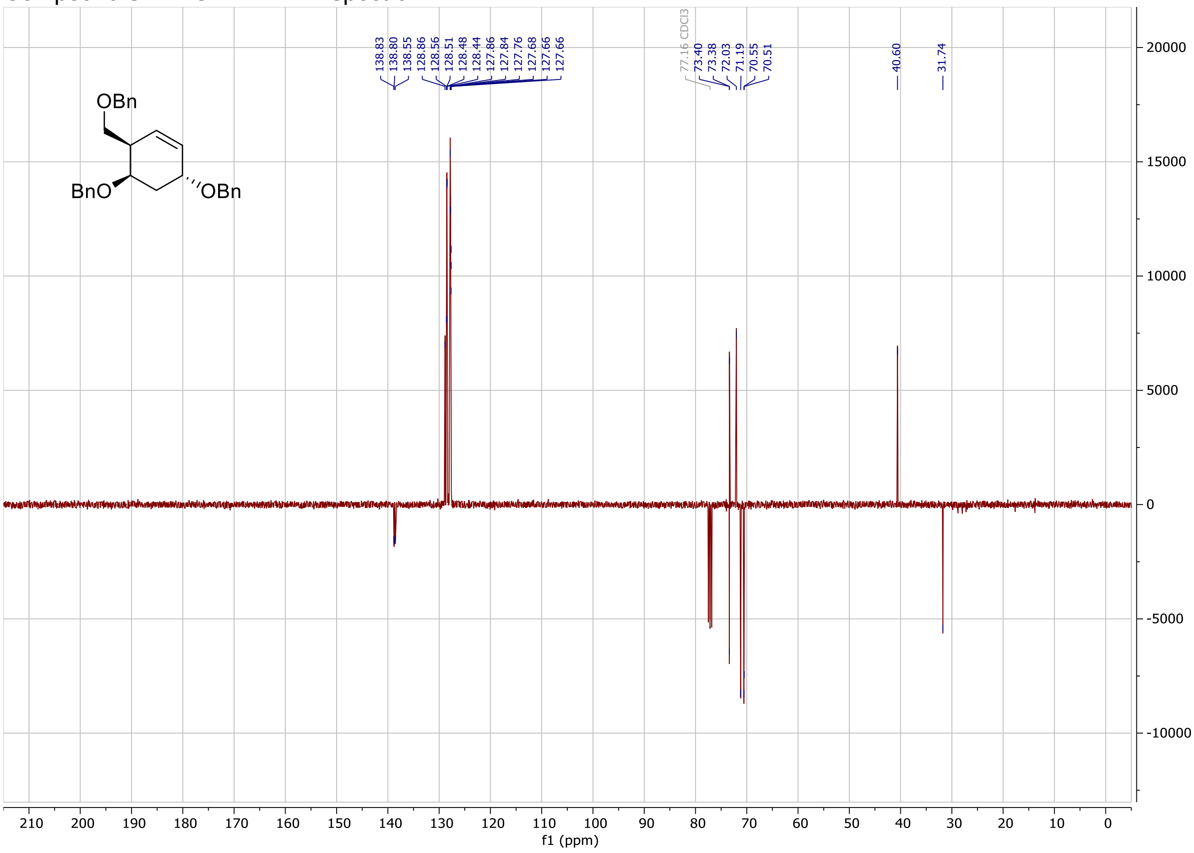

Compound **s73** ^{1}H NMR spectrum

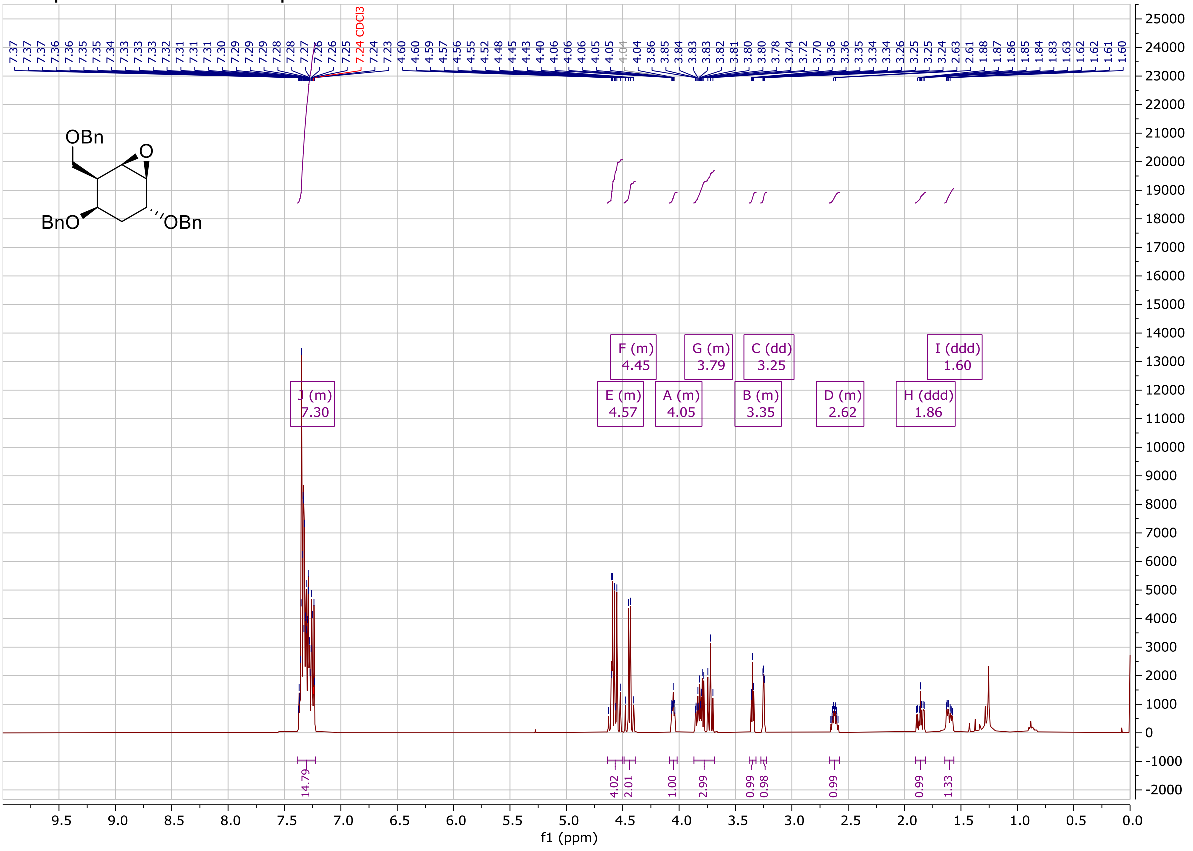

Compound **s73** ^{13}C NMR APT spectrum

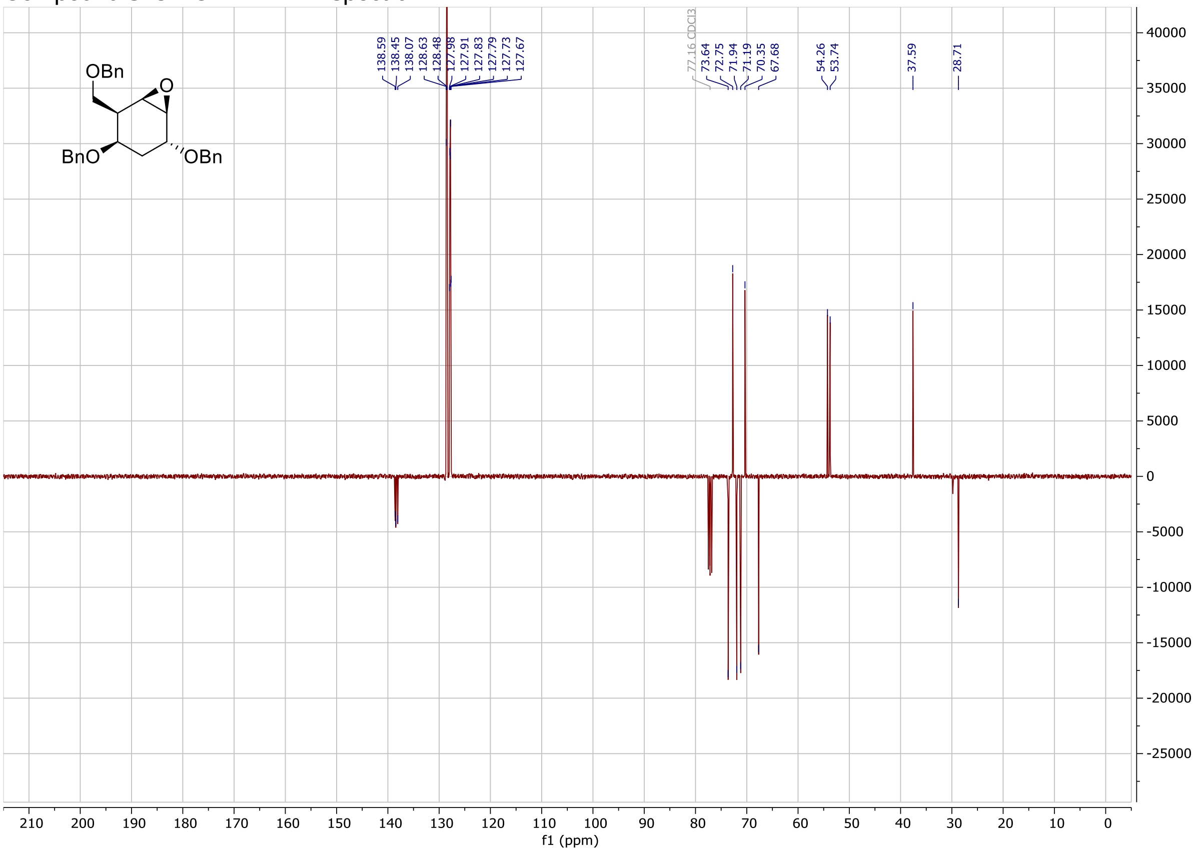

Compound **s73** ^{1}H-^{1}H COSY spectrum

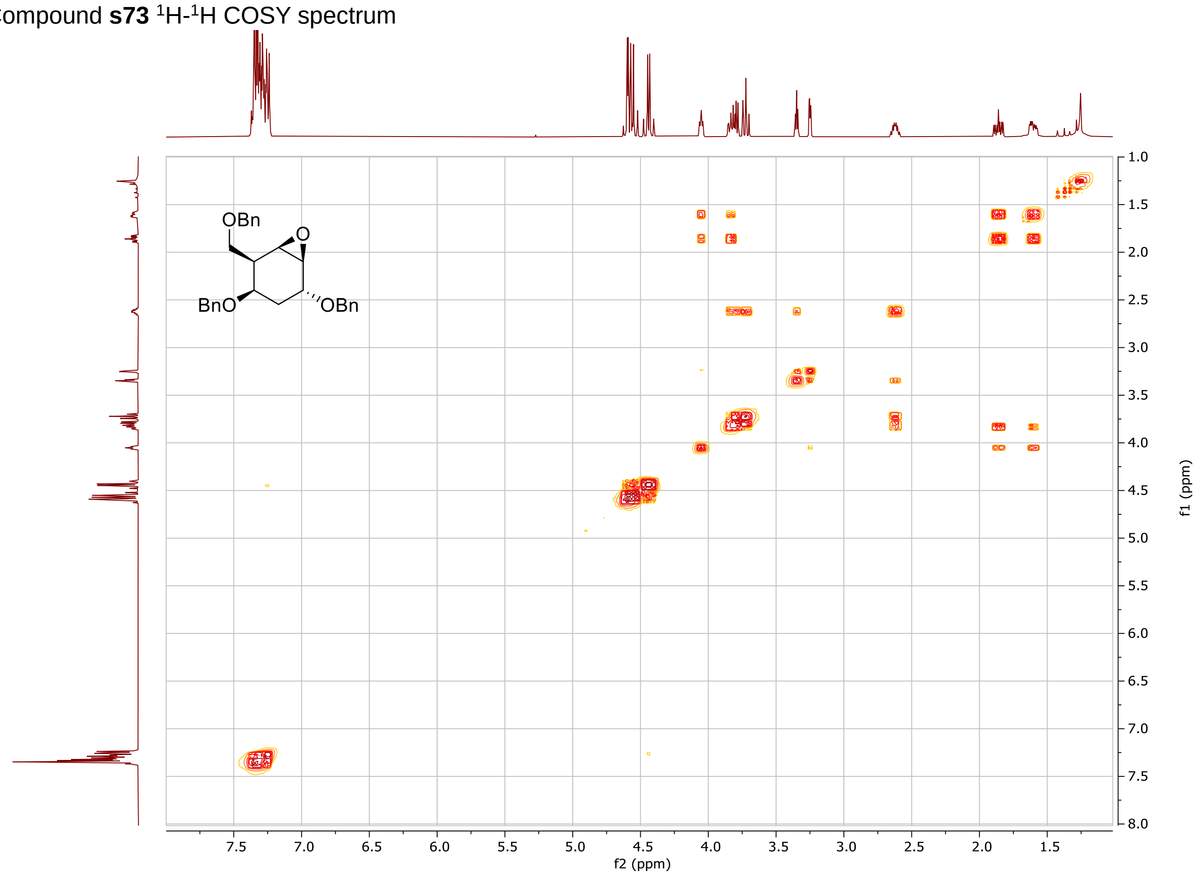

Compound **s73** ^{1}H-^{13}C HSQC spectrum

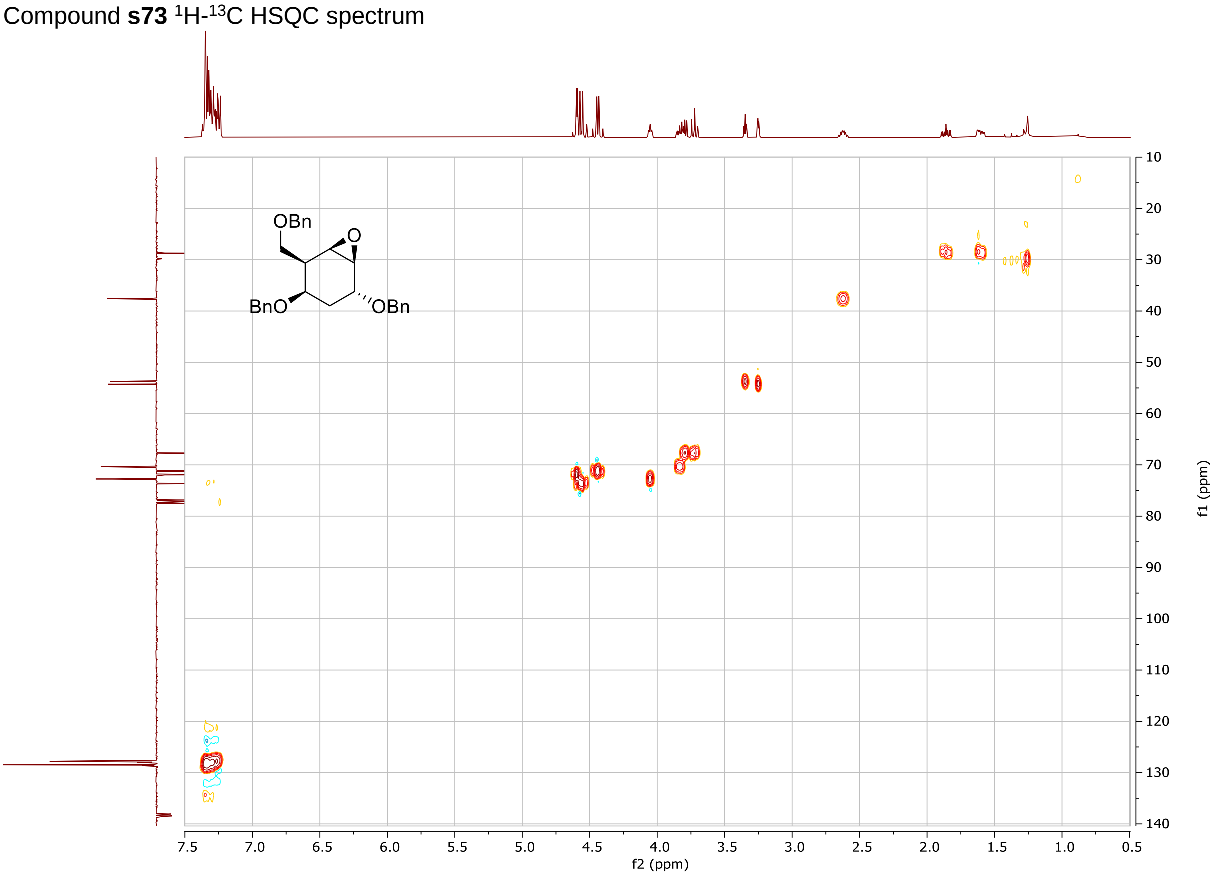

Compound **s74** ^{1}H NMR spectrum

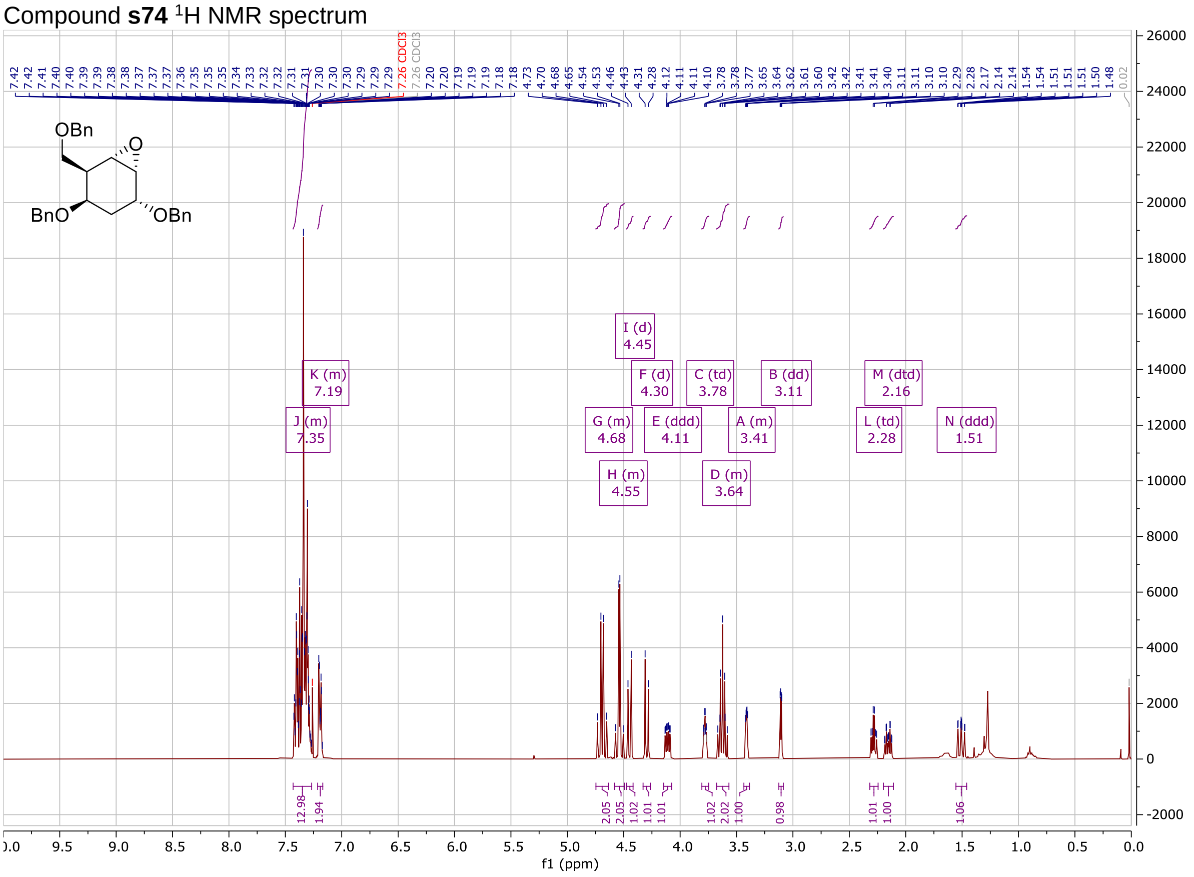

Compound **s74** ^{13}C NMR APT spectrum

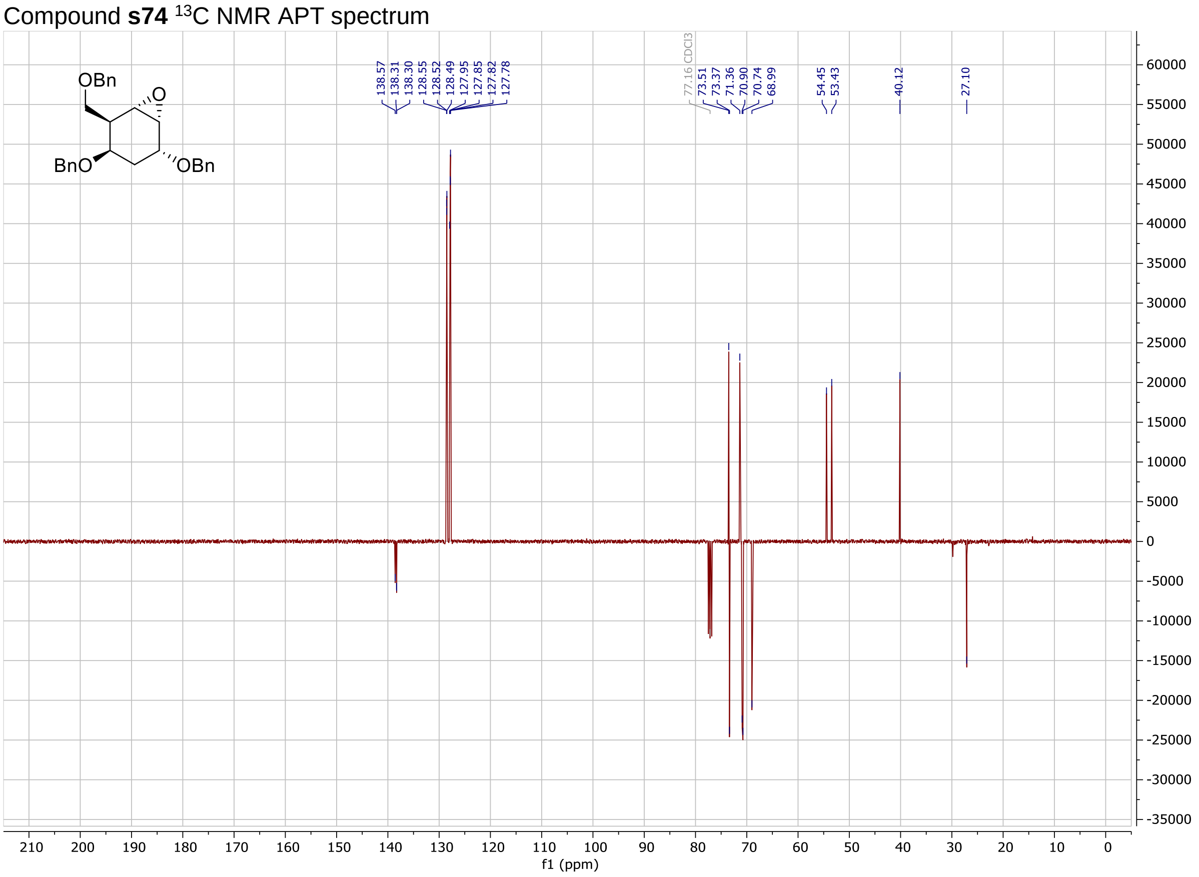

Compound **s74** ^{1}H-^{1}H COSY spectrum

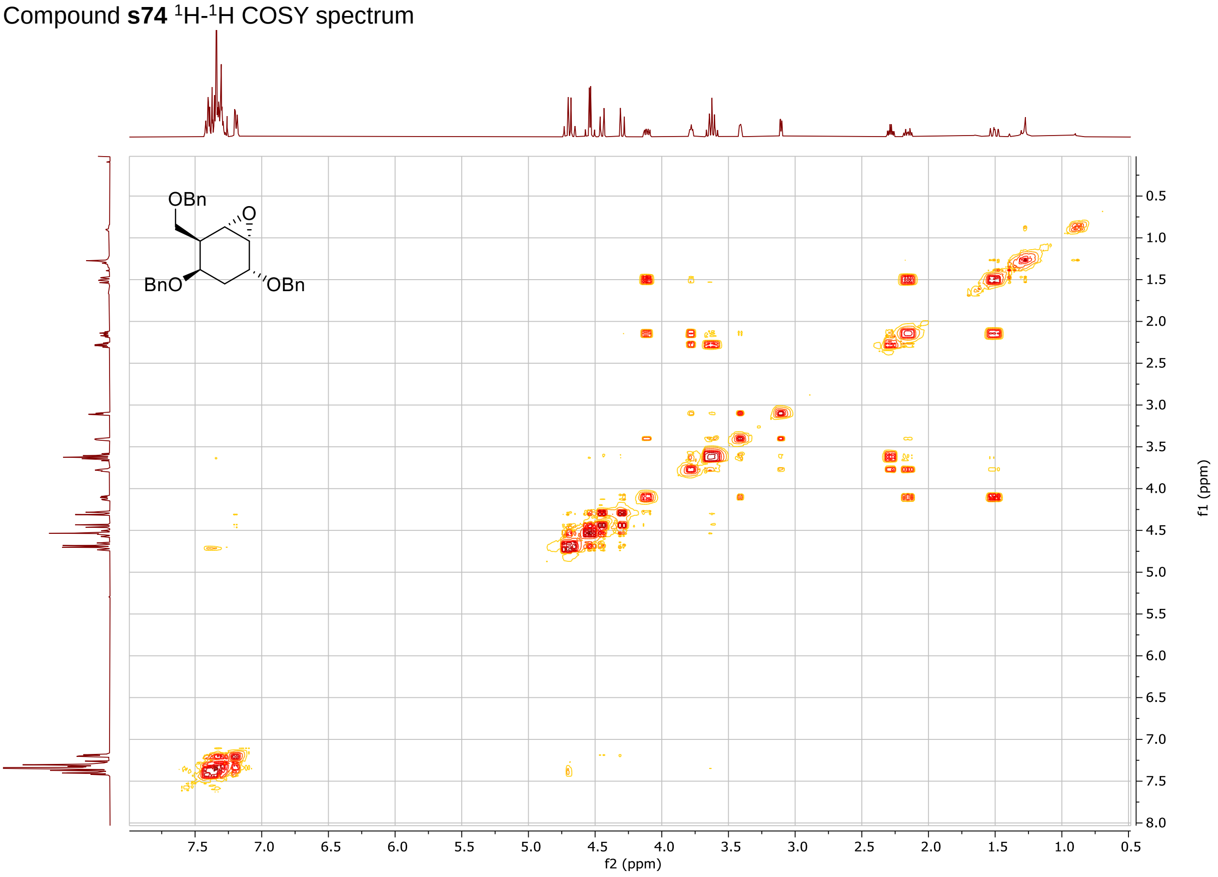

Compound **s74** ^{1}H-^{13}C HSQC spectrum

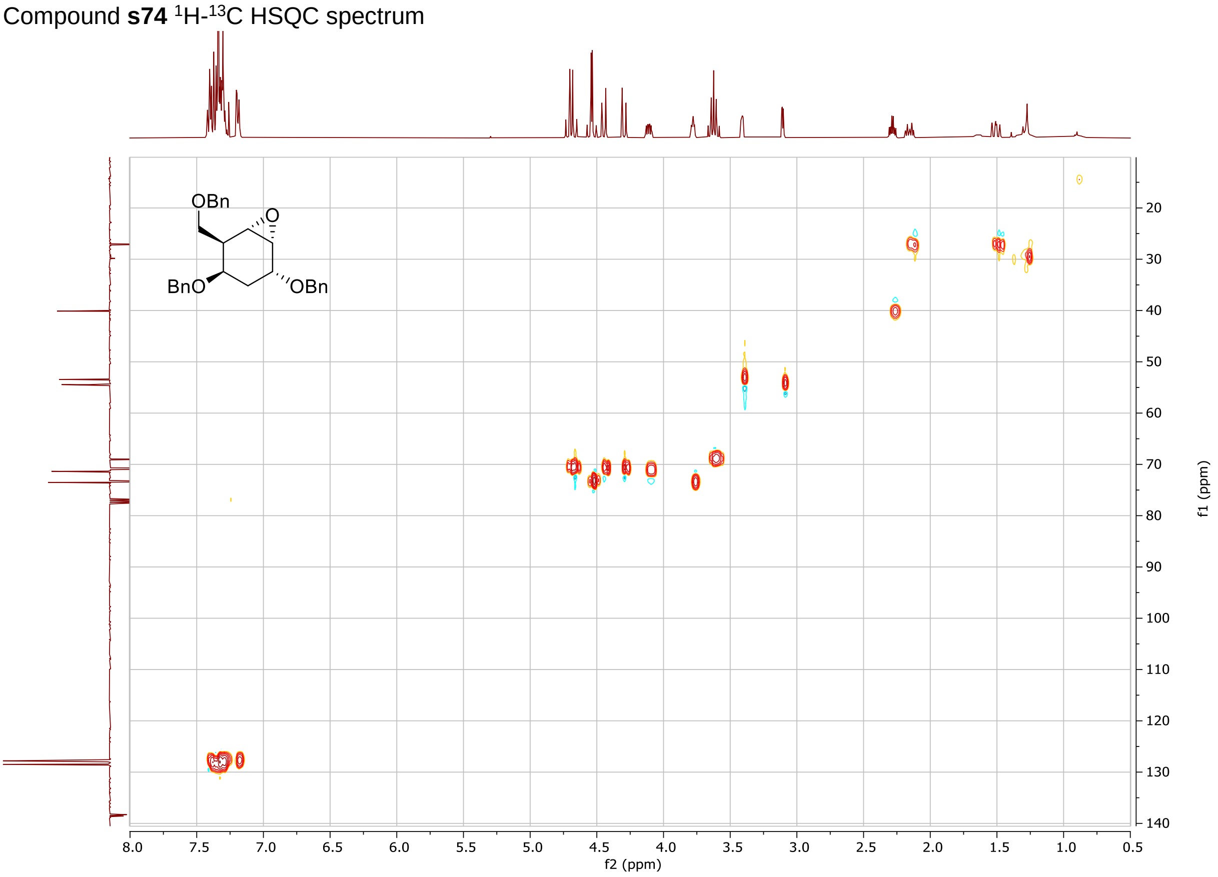

Compound **44** ^{1}H NMR spectrum

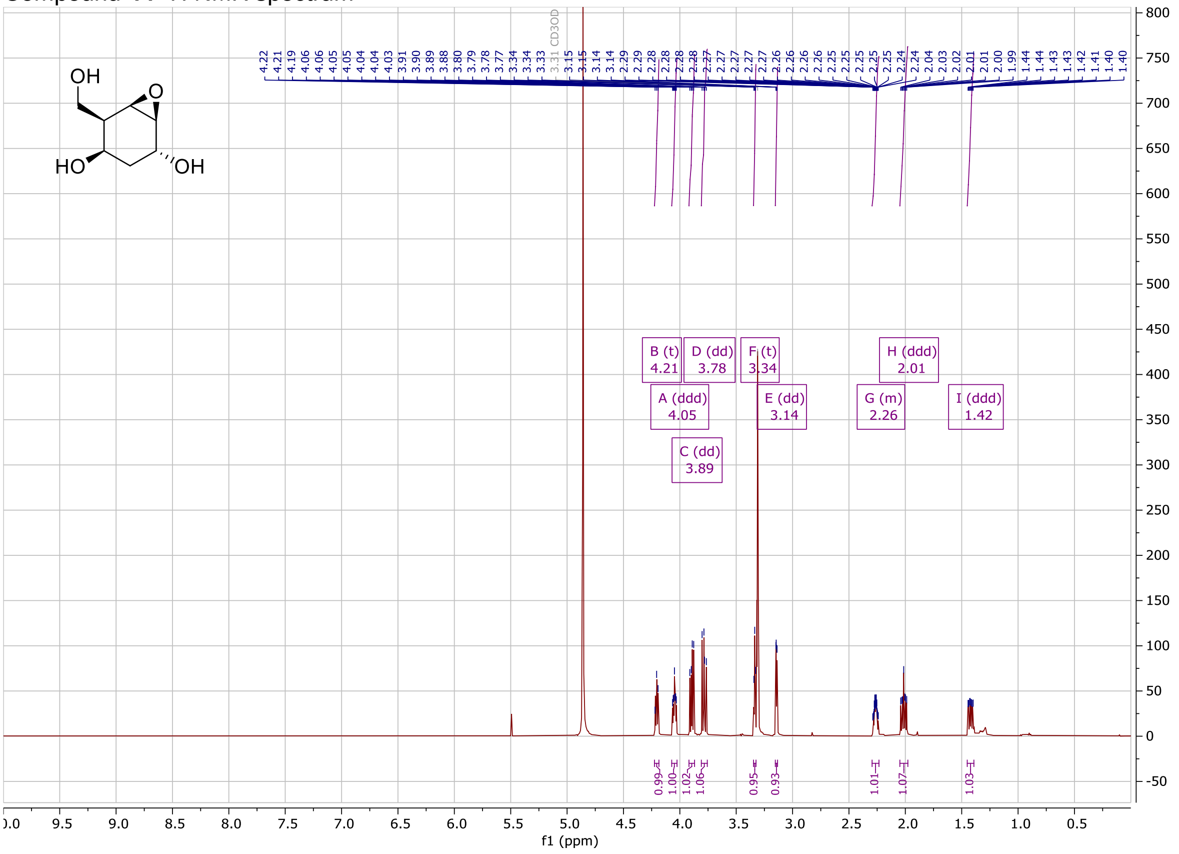

Compound **44** ^{13}C NMR APT spectrum

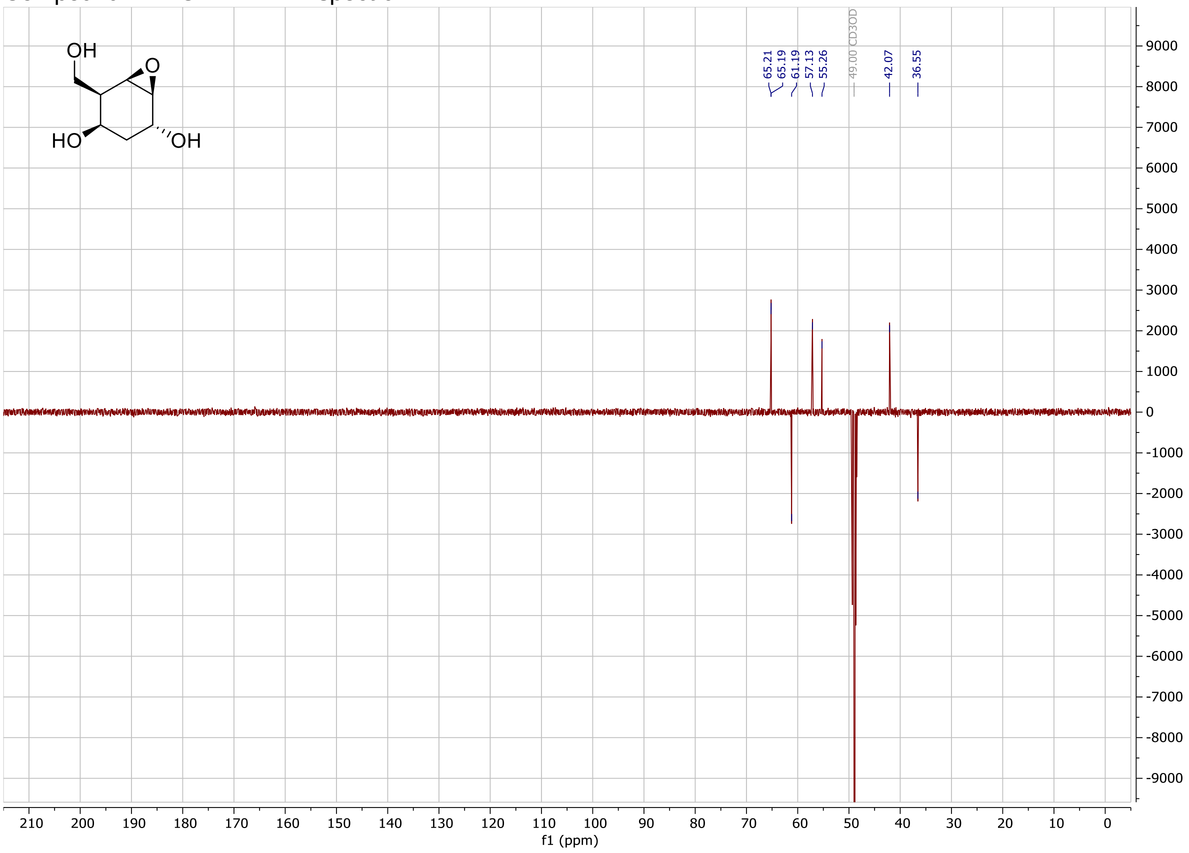

Compound **43** ^{1}H NMR spectrum

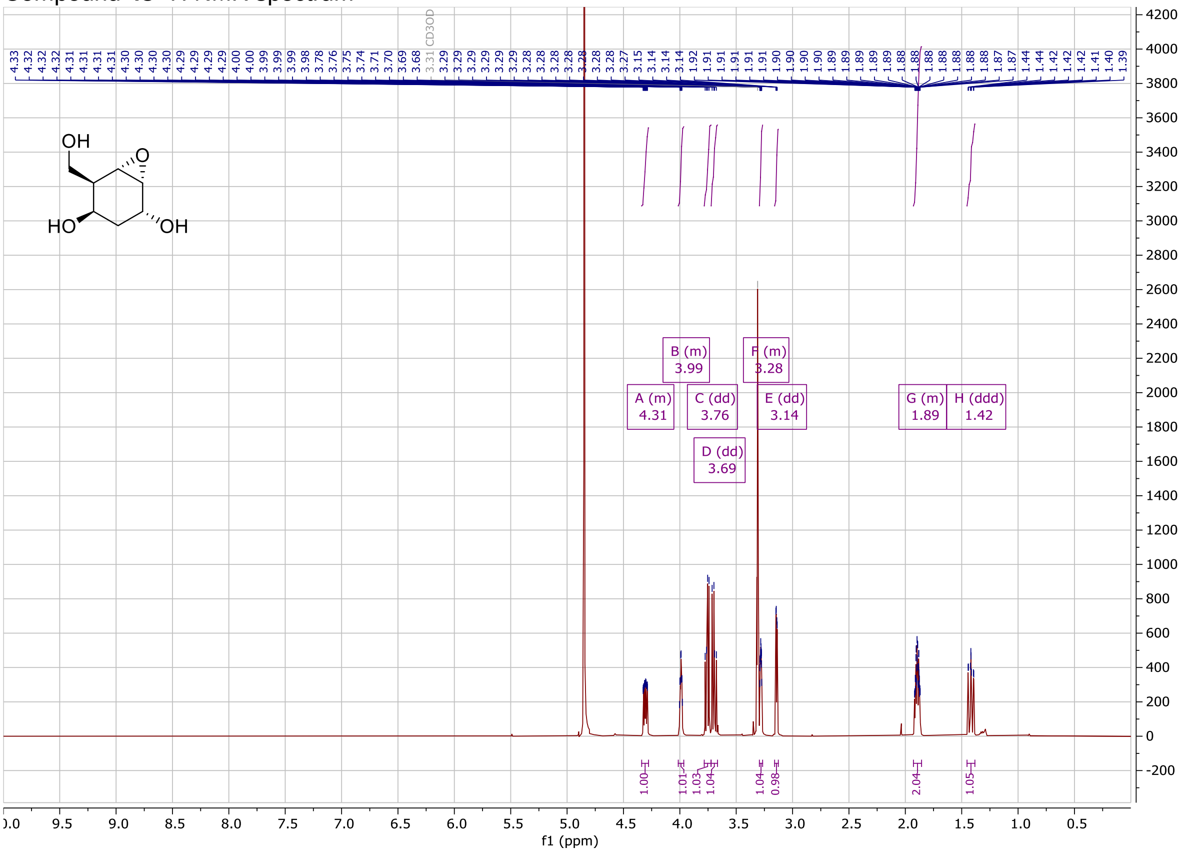

Compound **43** ^{13}C NMR APT spectrum

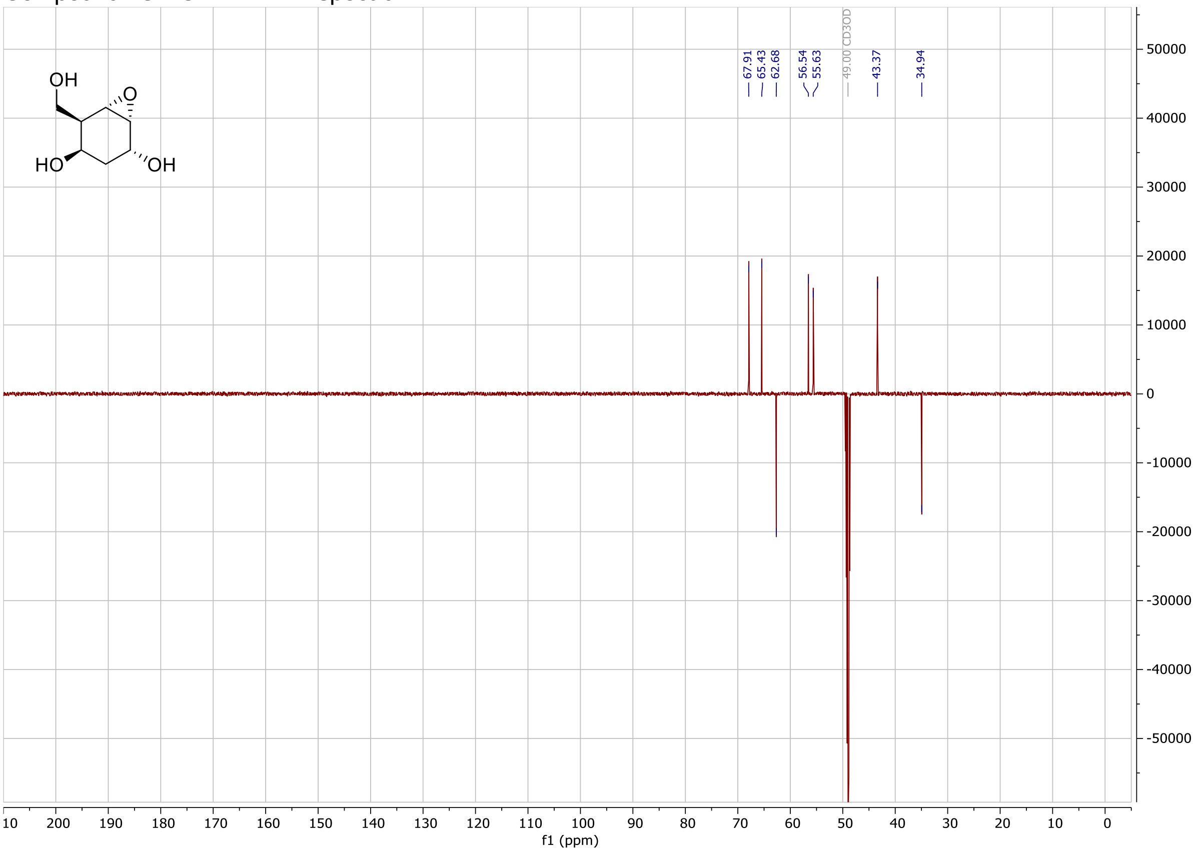

Compound **s75** ^{1}H NMR spectrum

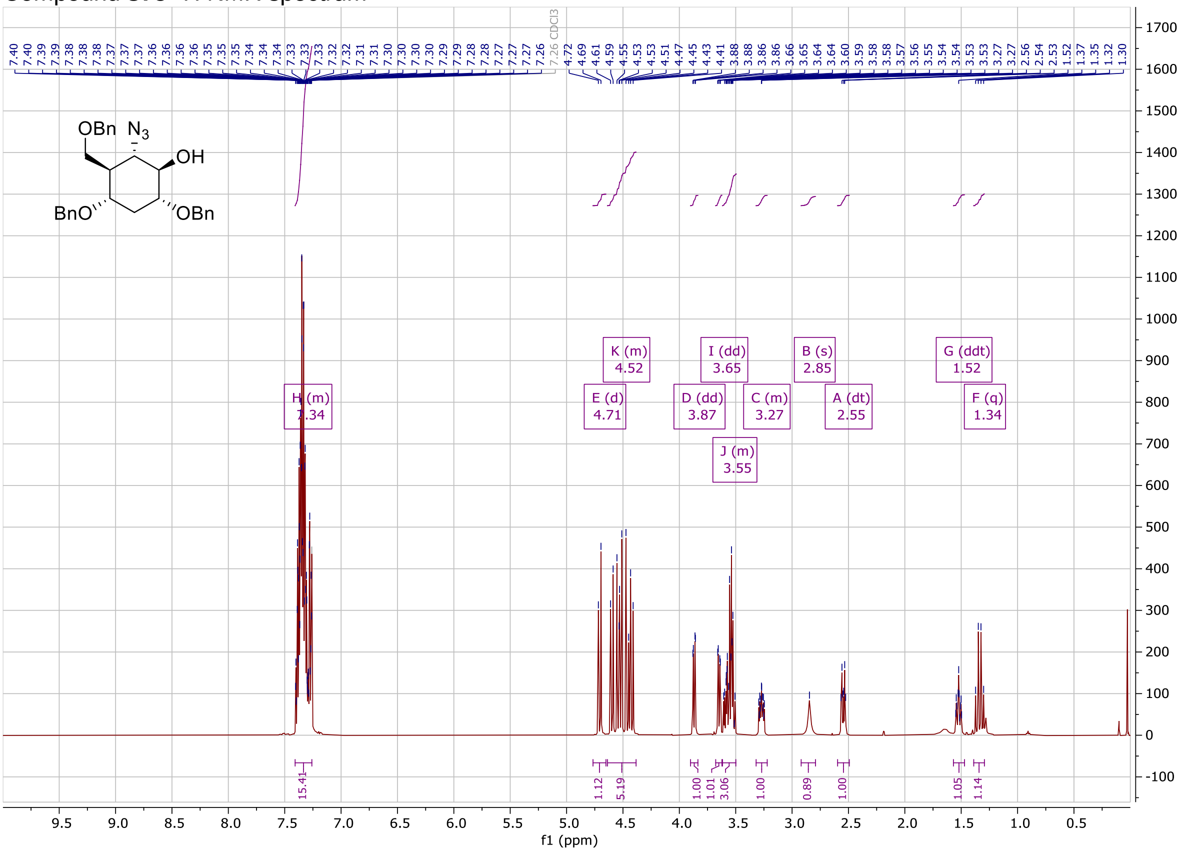

Compound **s75** ^{13}C NMR APT spectrum

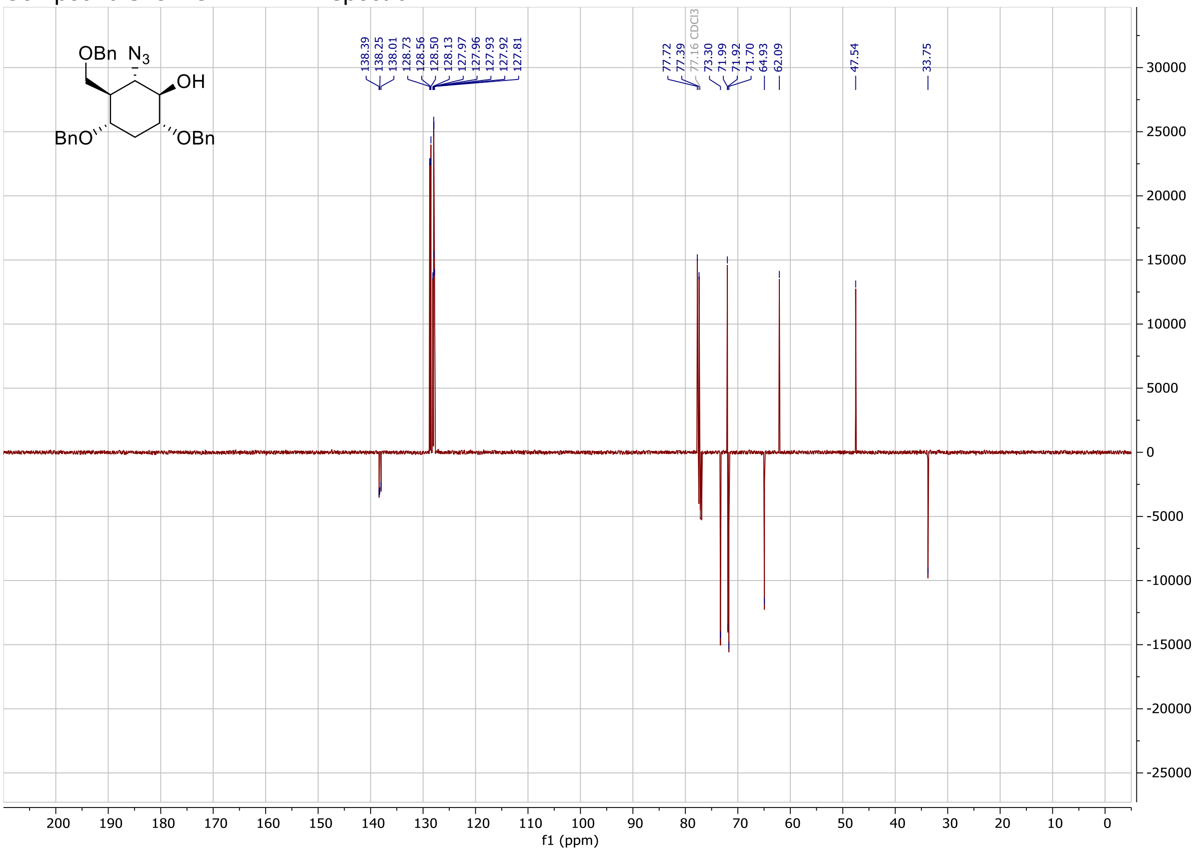

Compound **s75** ^{1}H-^{1}H COSY spectrum

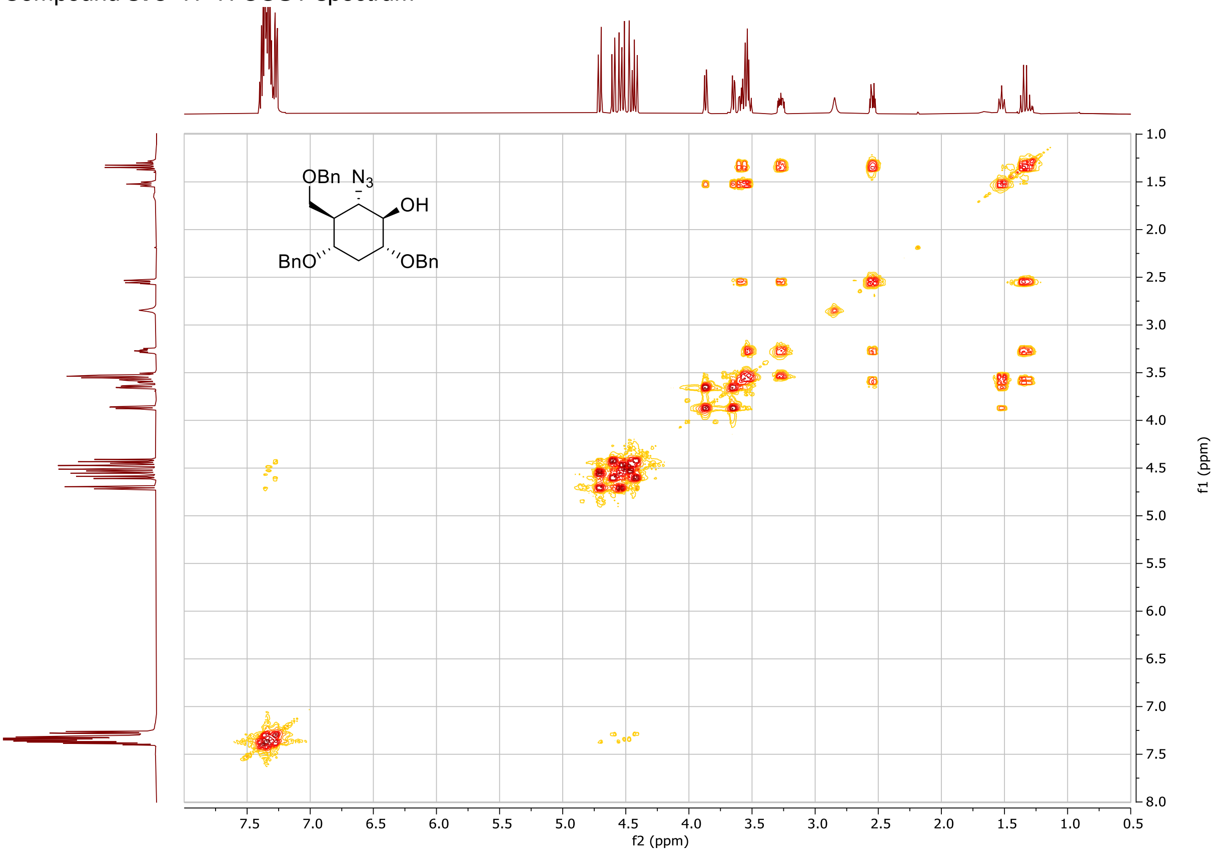

Compound **s75** ^{1}H-^{13}C HSQC spectrum

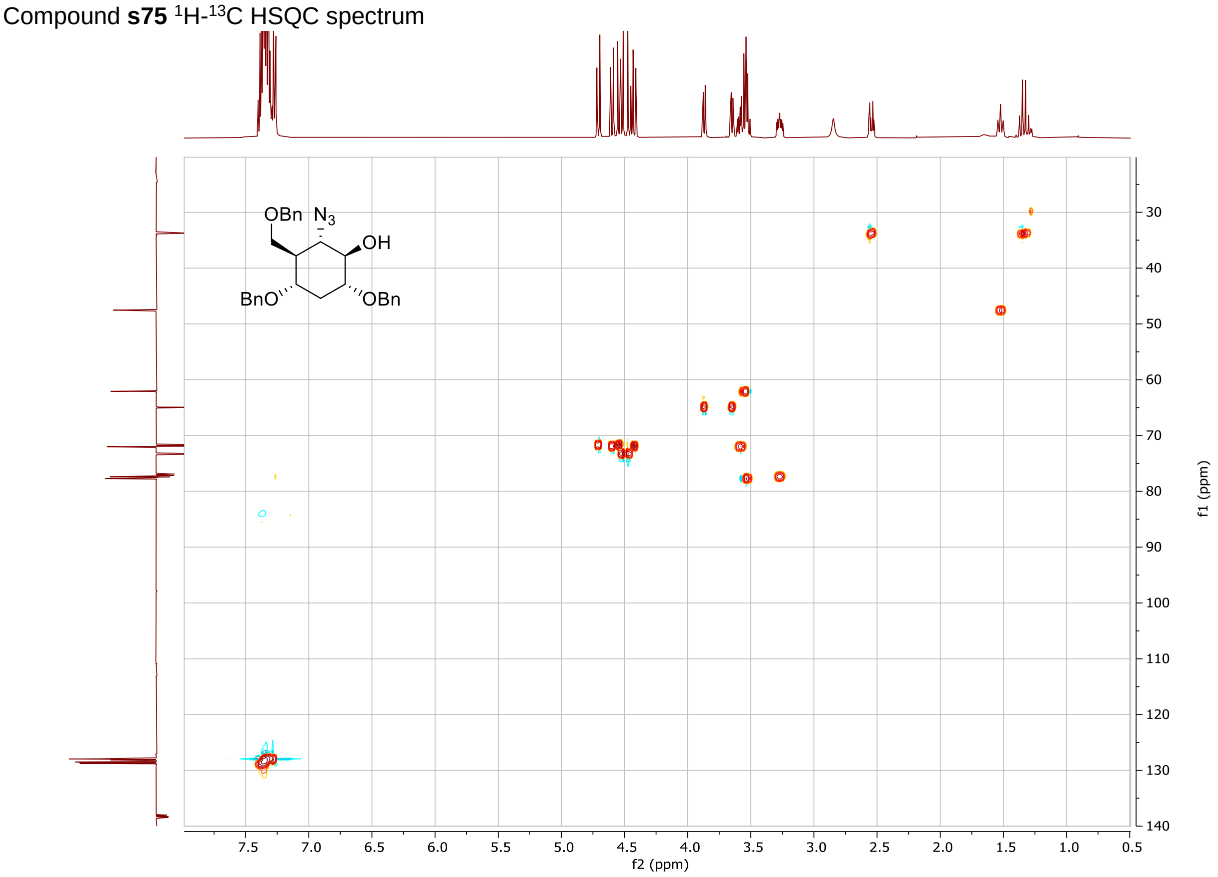

Compound **s75** ^{1}H-^{1}H NOESY spectrum

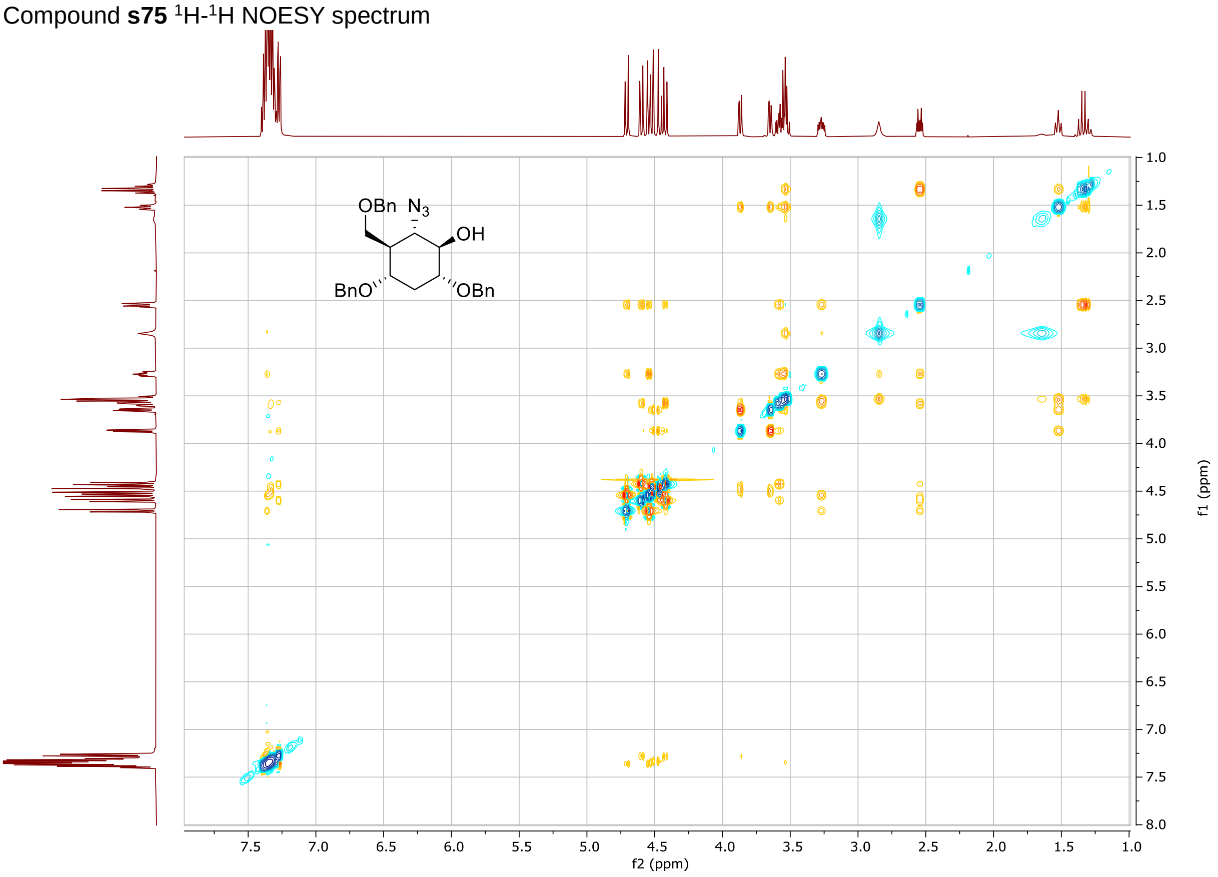

Compound **s75** ^{1}H-^{13}C HMBC spectrum

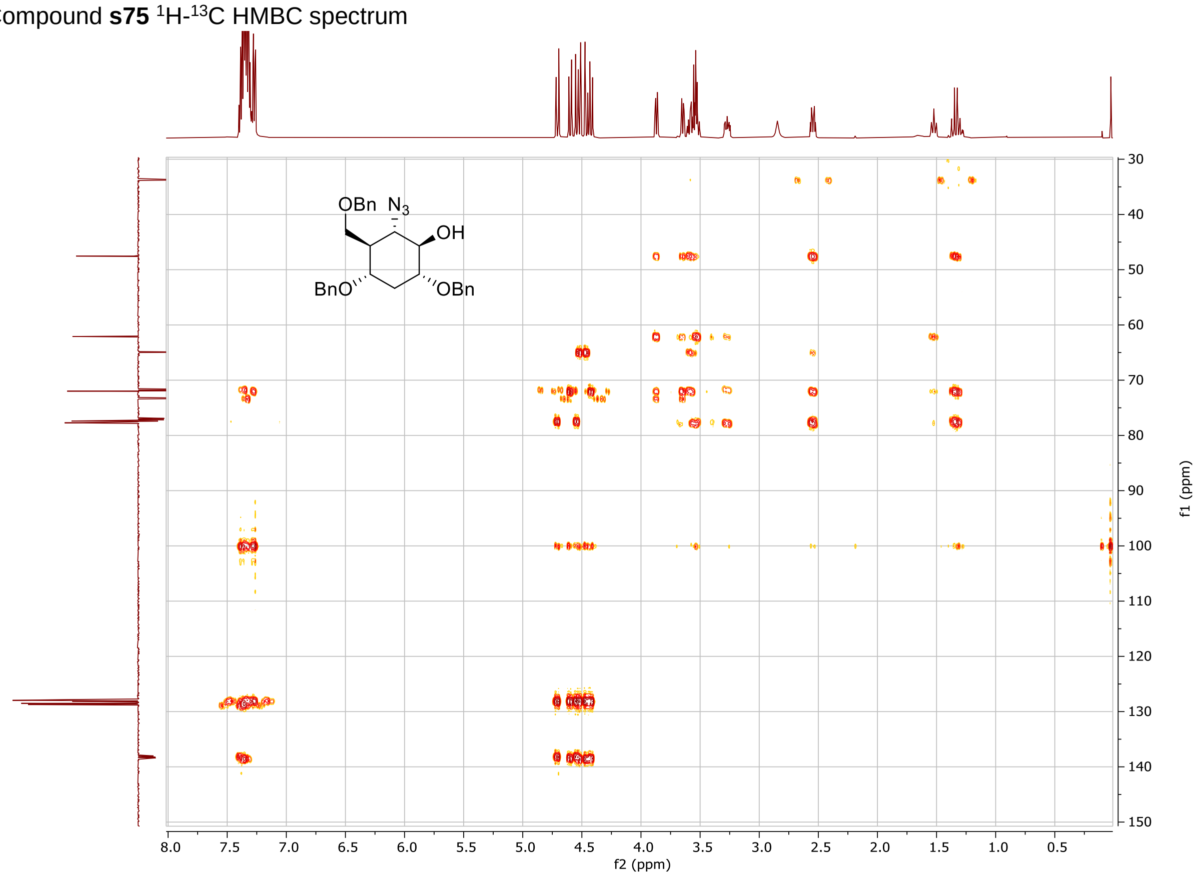

Compound **s76** ^{1}H NMR spectrum

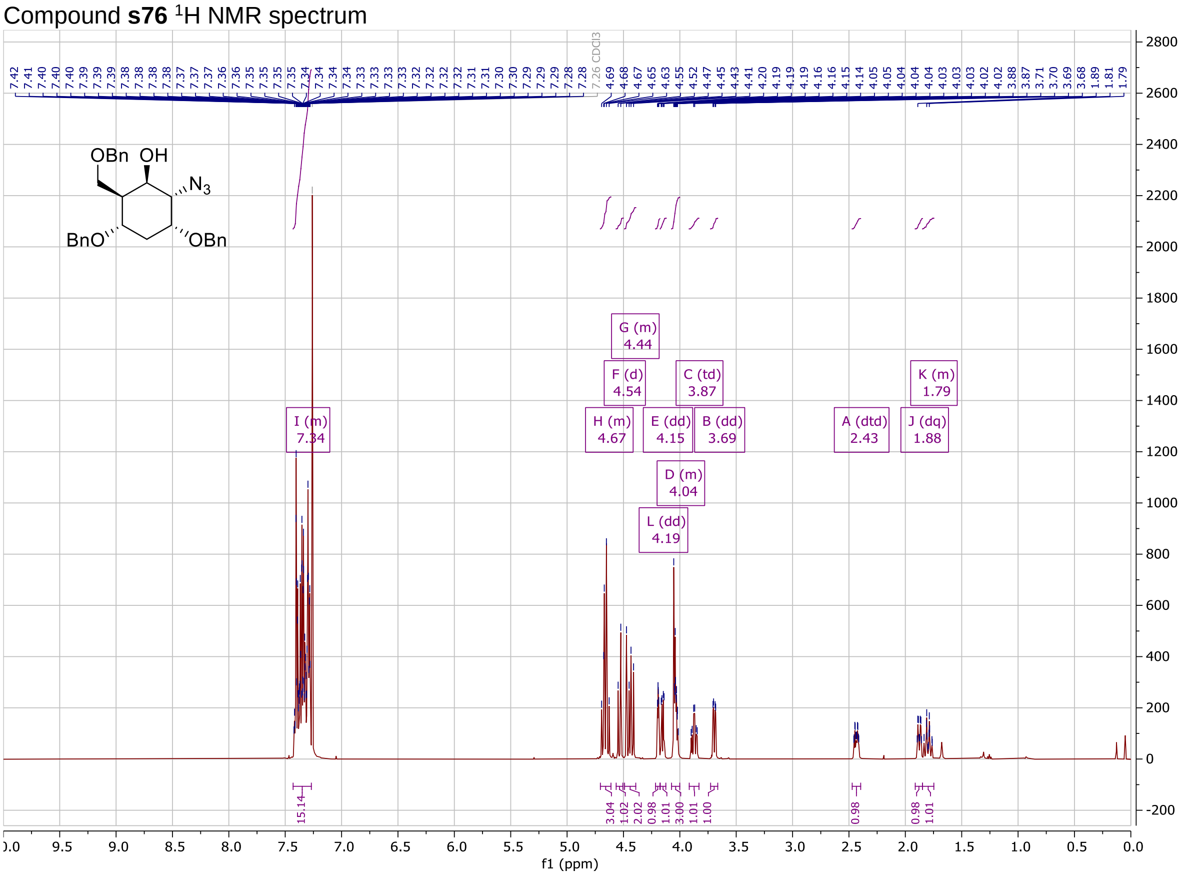

Compound **s76** ^{13}C NMR APT spectrum

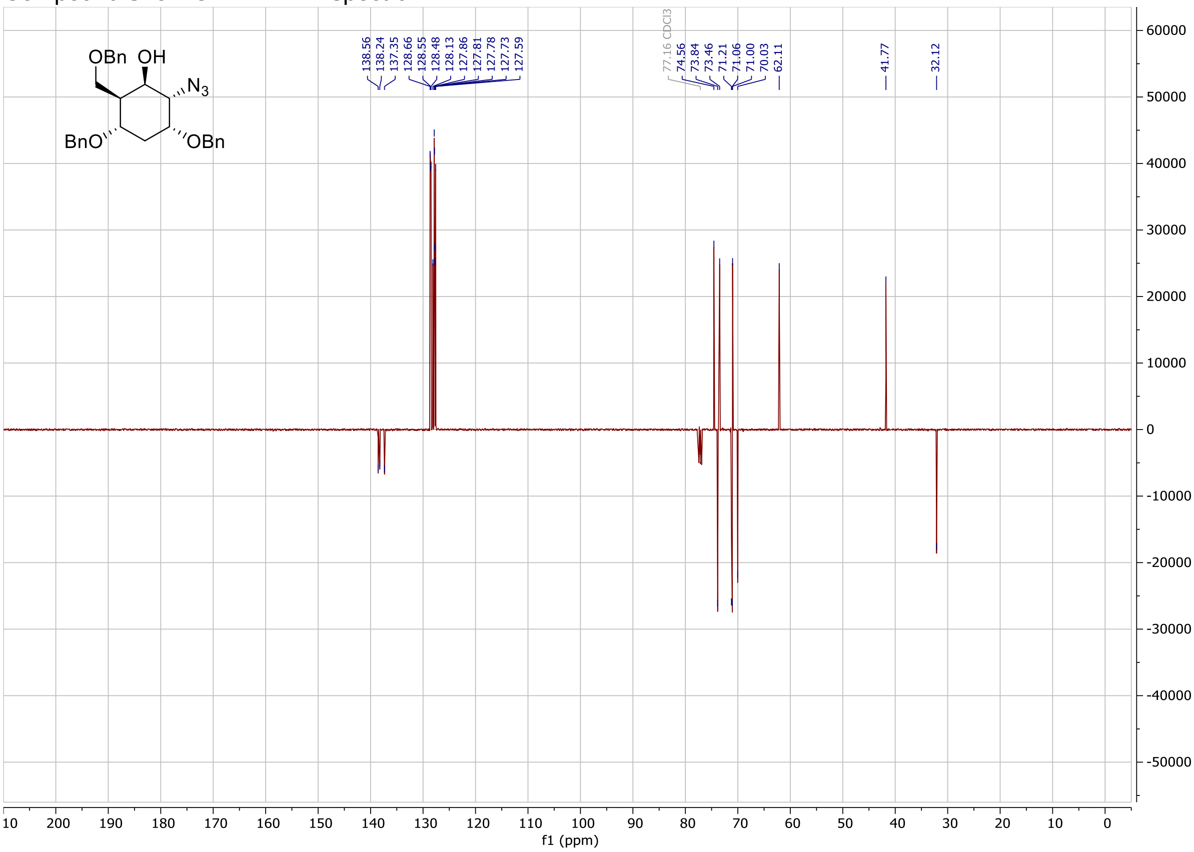

Compound **s76** ^{1}H-^{1}H COSY spectrum

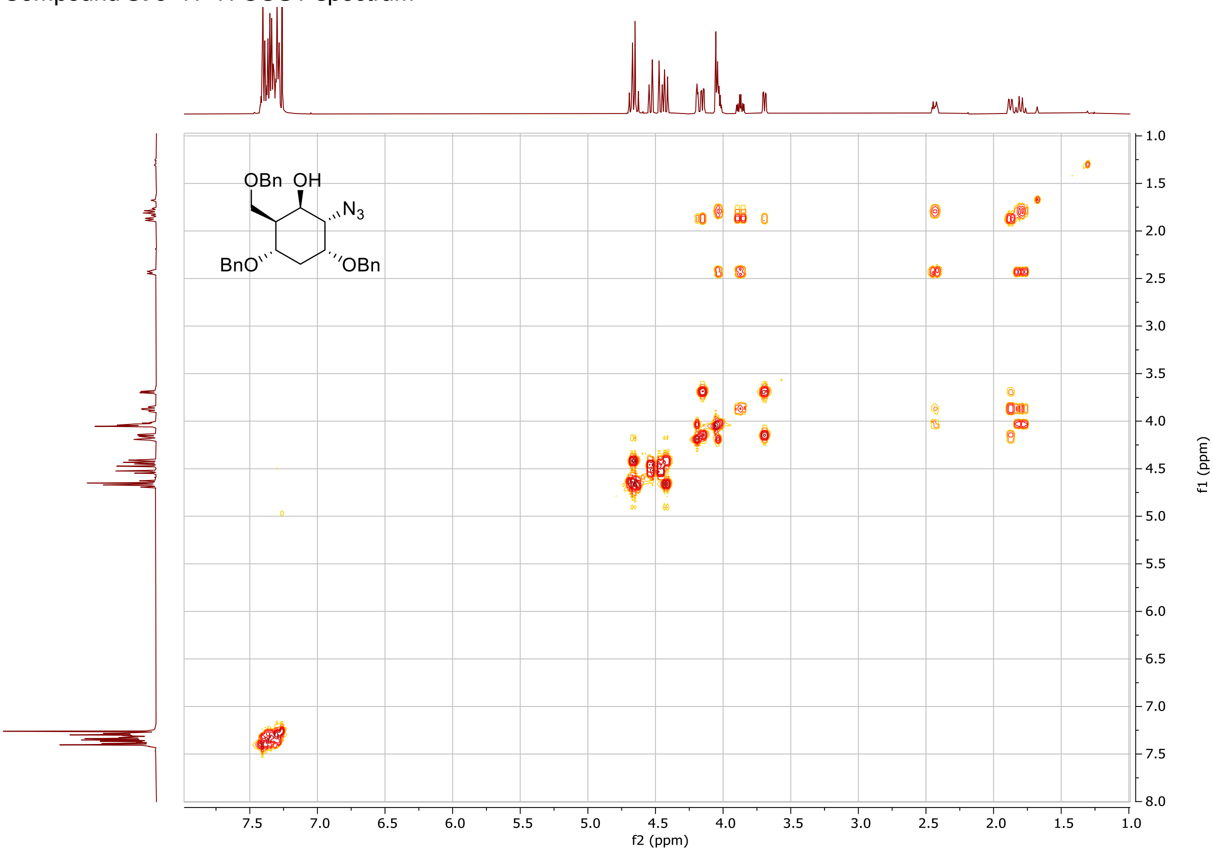

Compound **s76** ^{1}H-^{13}C HSQC spectrum

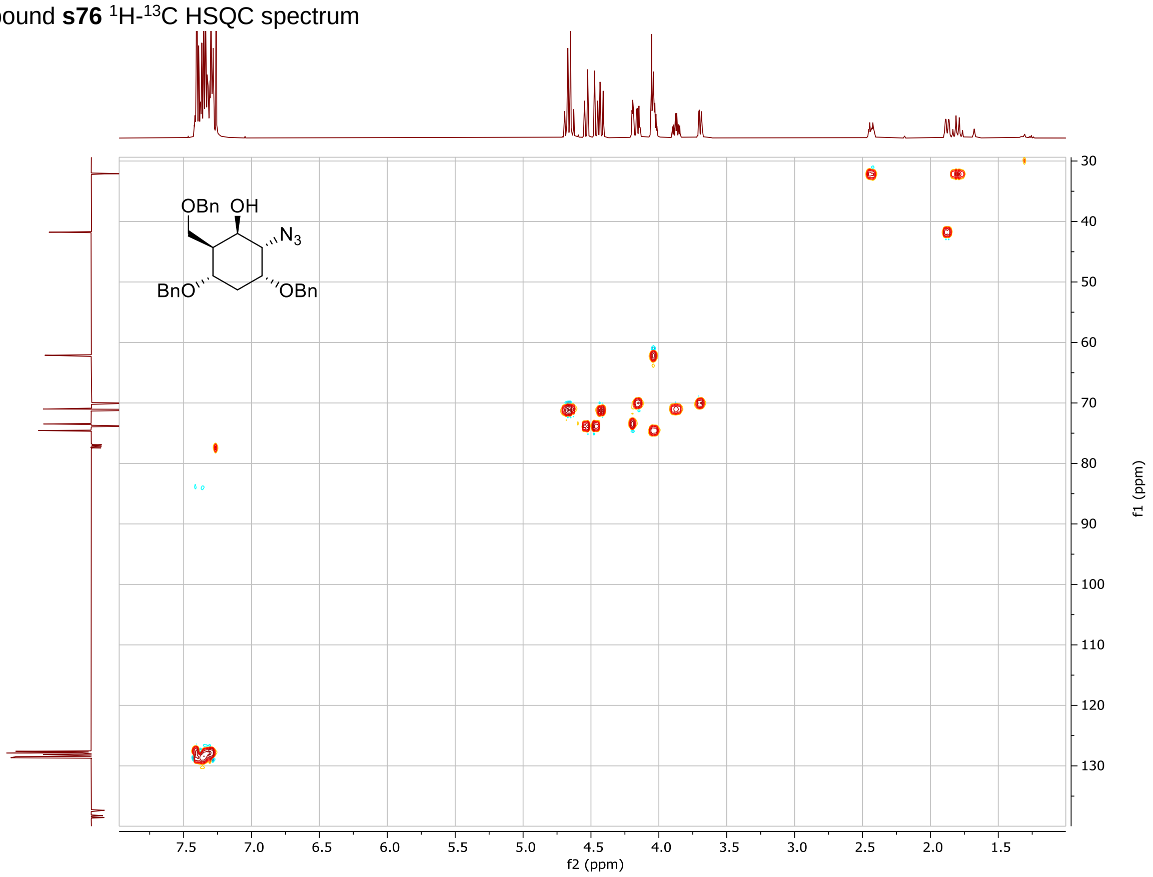

Compound **s76** ^{1}H-^{1}H NOESY spectrum

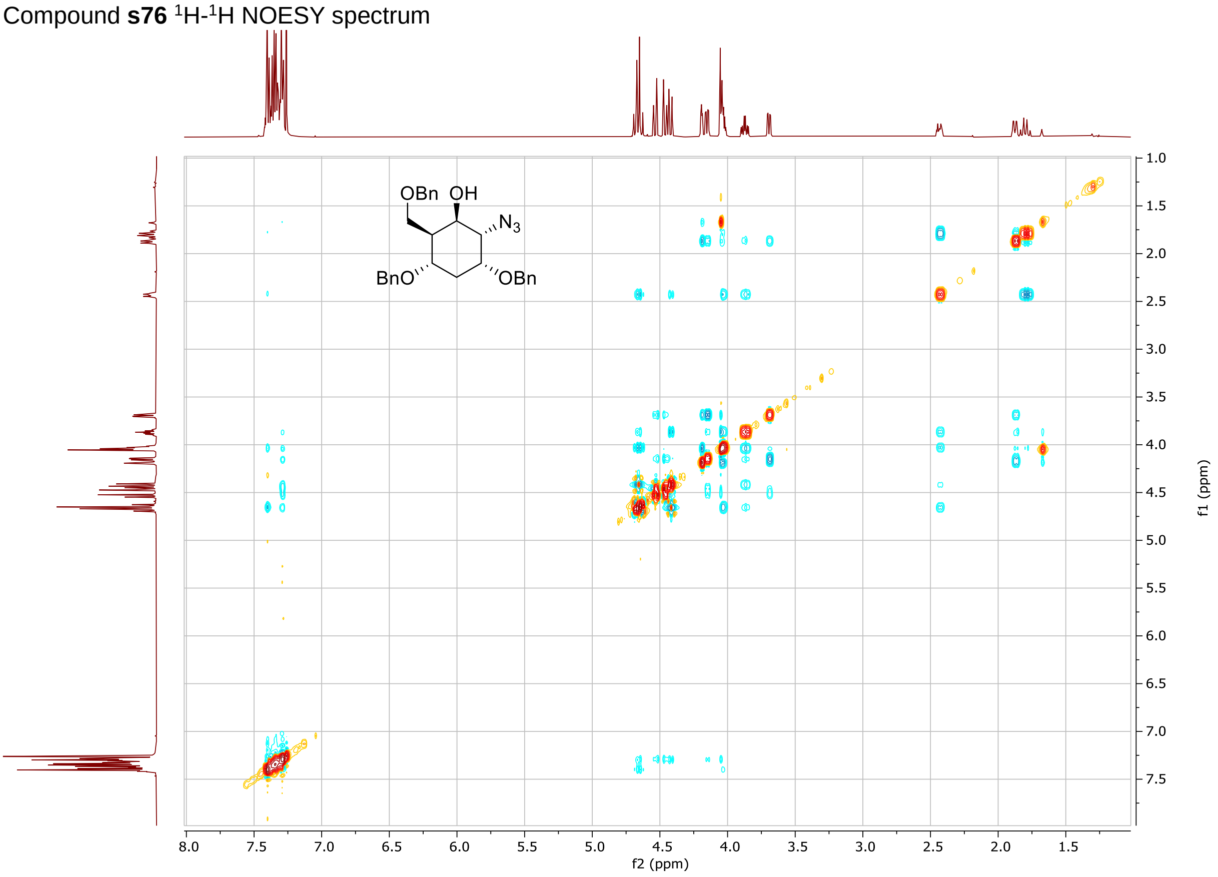

Compound **s76** ^{1}H-^{13}C HMBC spectrum

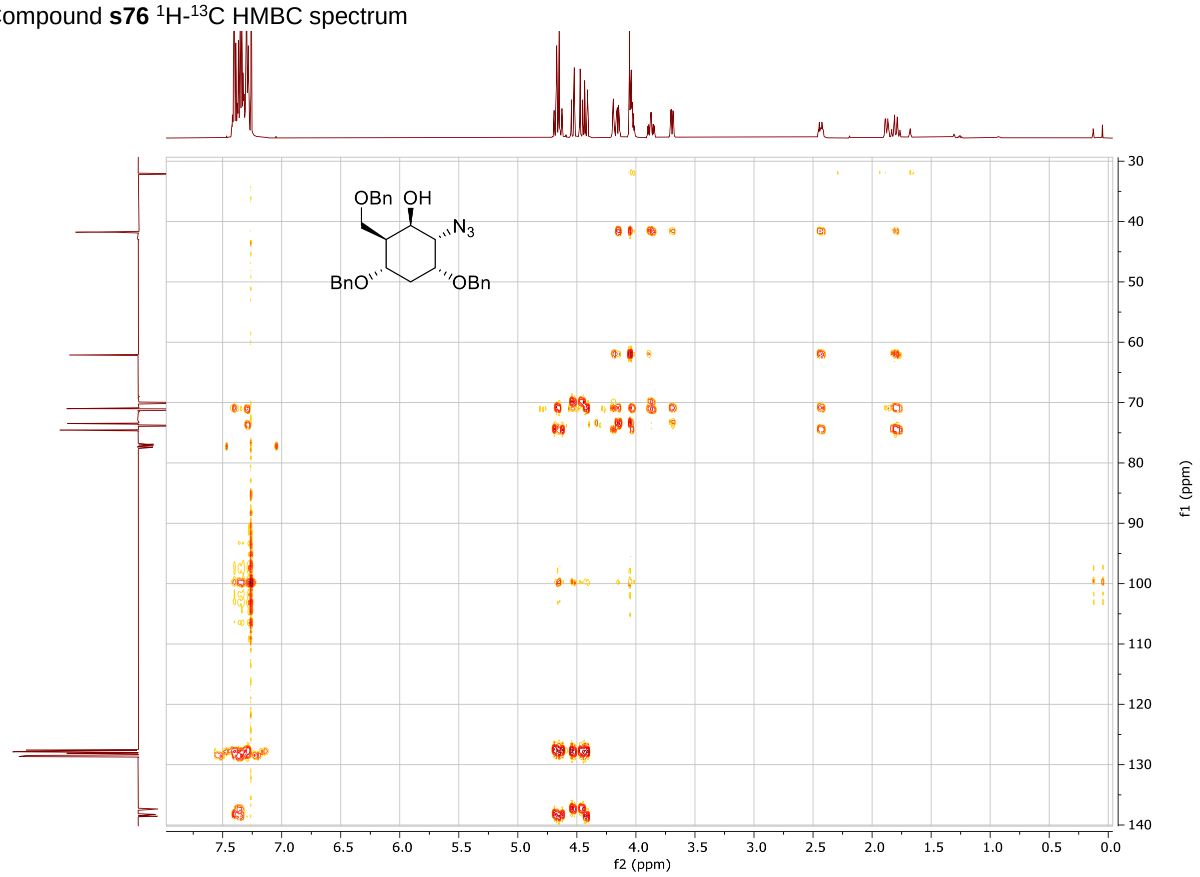

Compound **s77** ^{1}H NMR spectrum

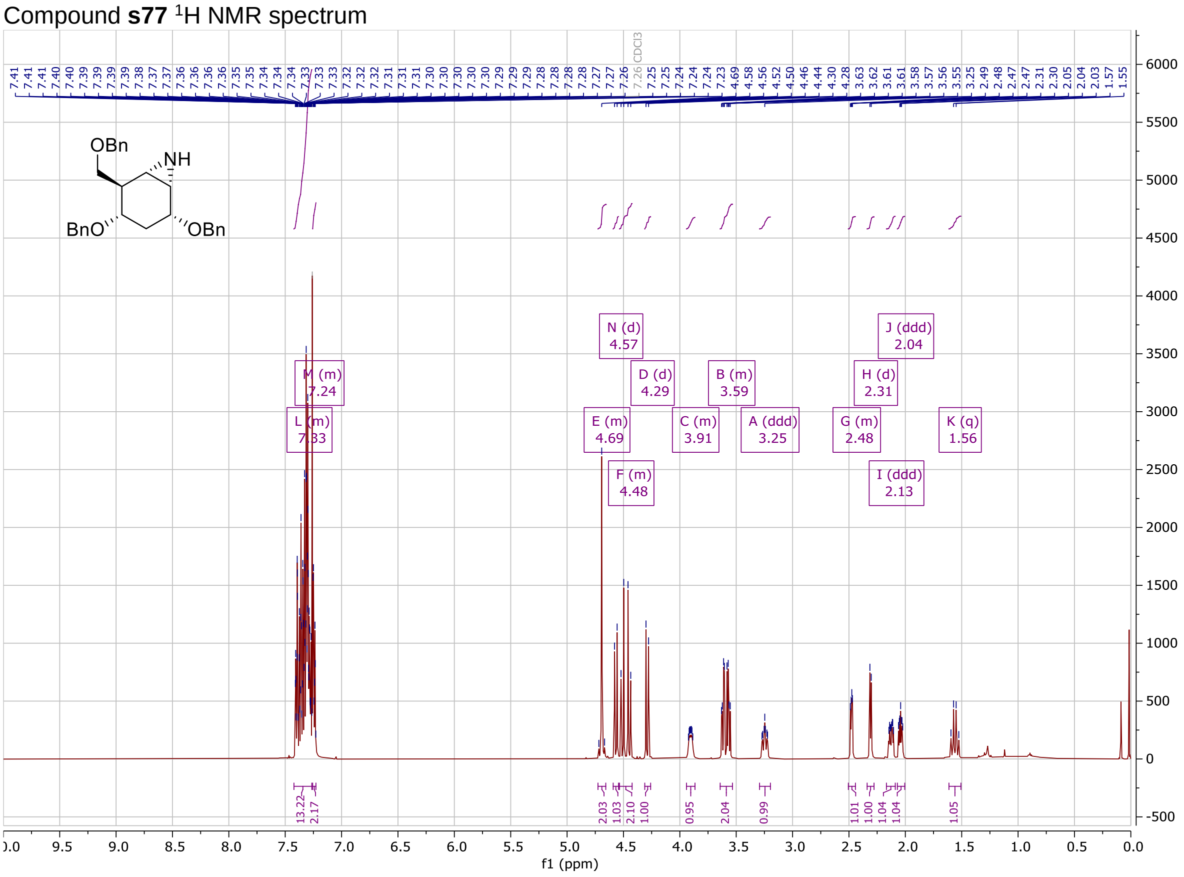

Compound **s77** ^{13}C NMR APT spectrum

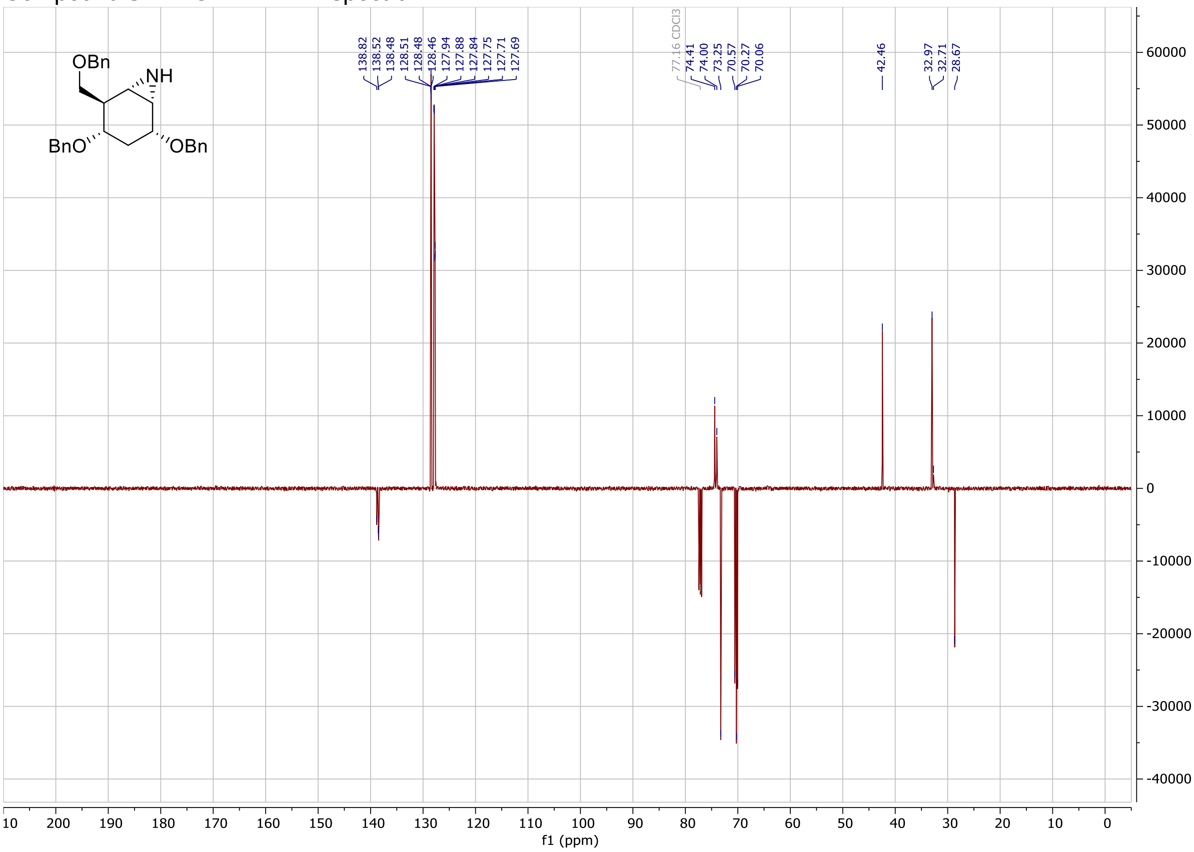

Compound **s77** ^{1}H-^{1}H COSY spectrum

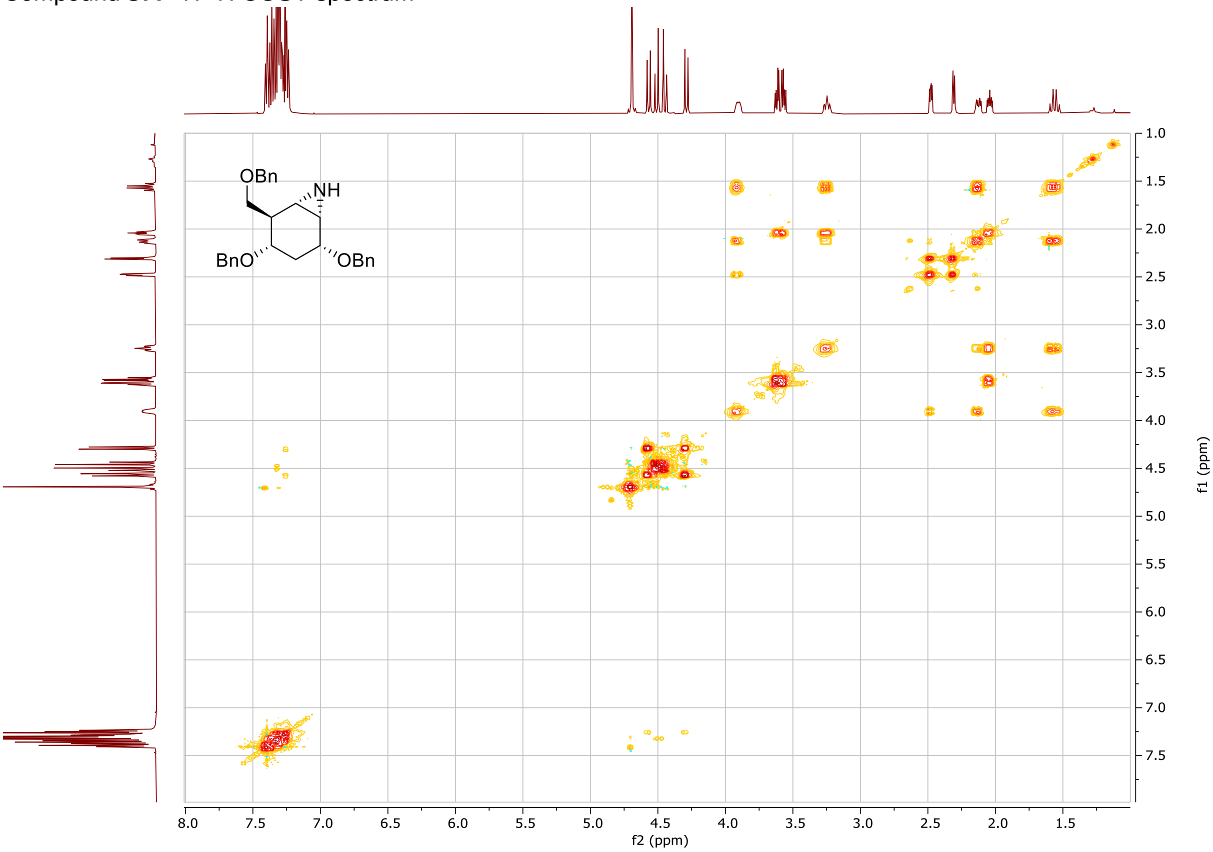

Compound **s77** ^{1}H-^{13}C HSQC spectrum

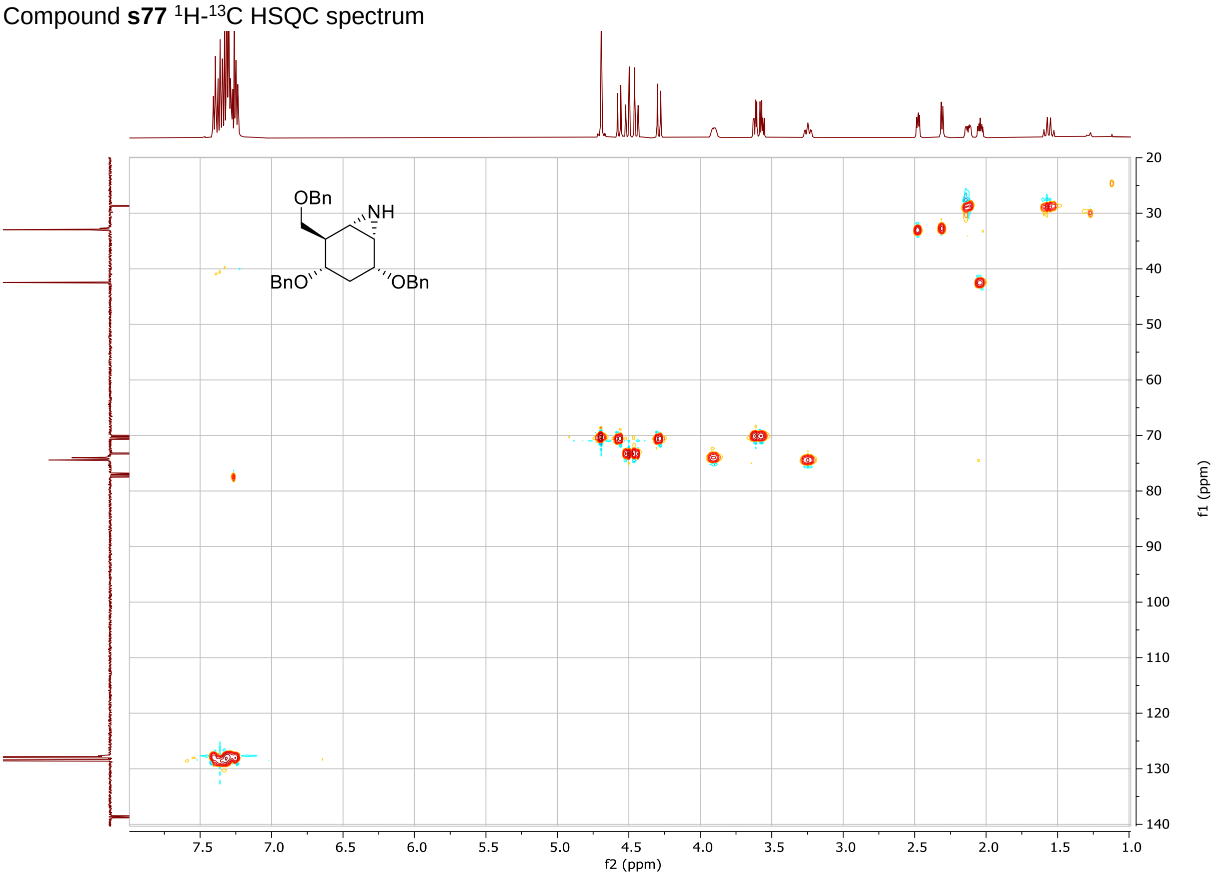

Compound **s77** ^{1}H-^{1}H NOESY spectrum

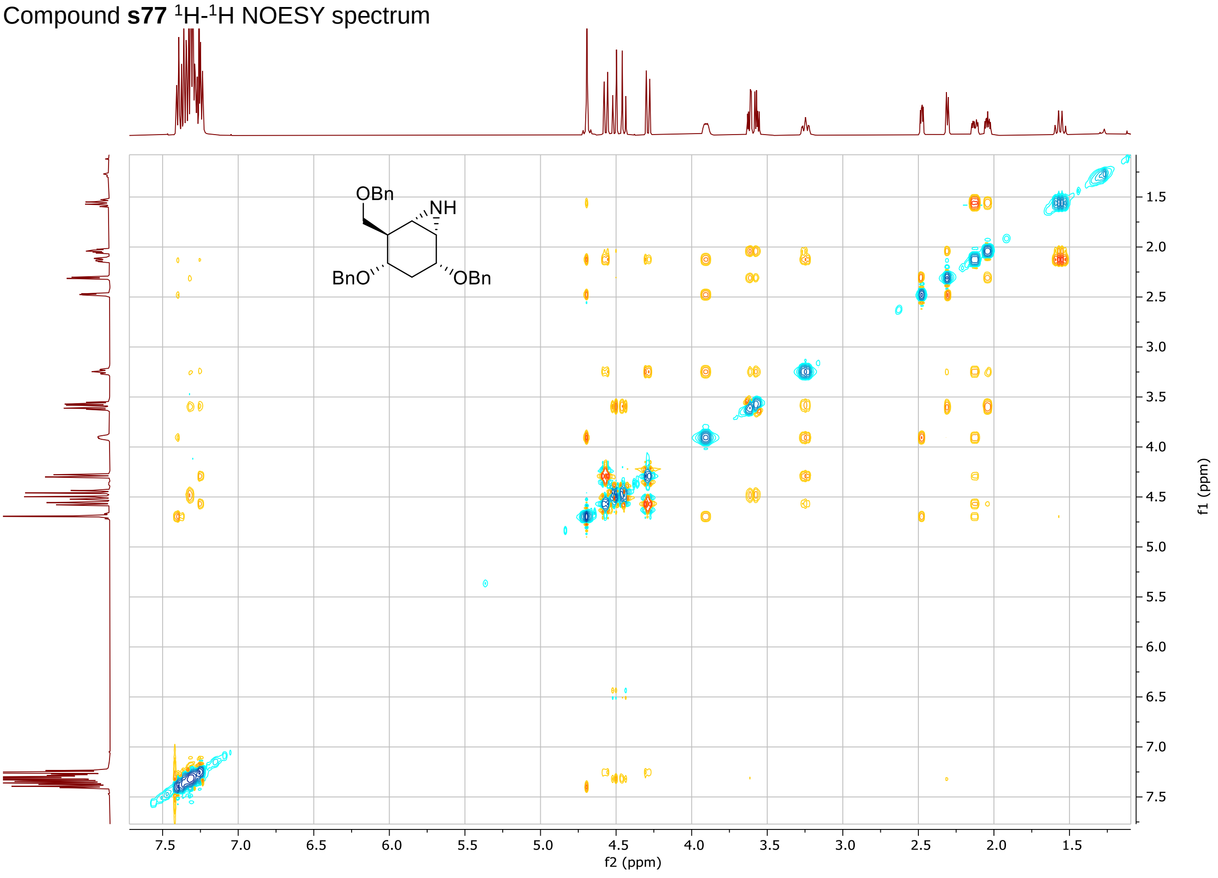

Compound **s77** ^{1}H-^{13}C HMBC spectrum

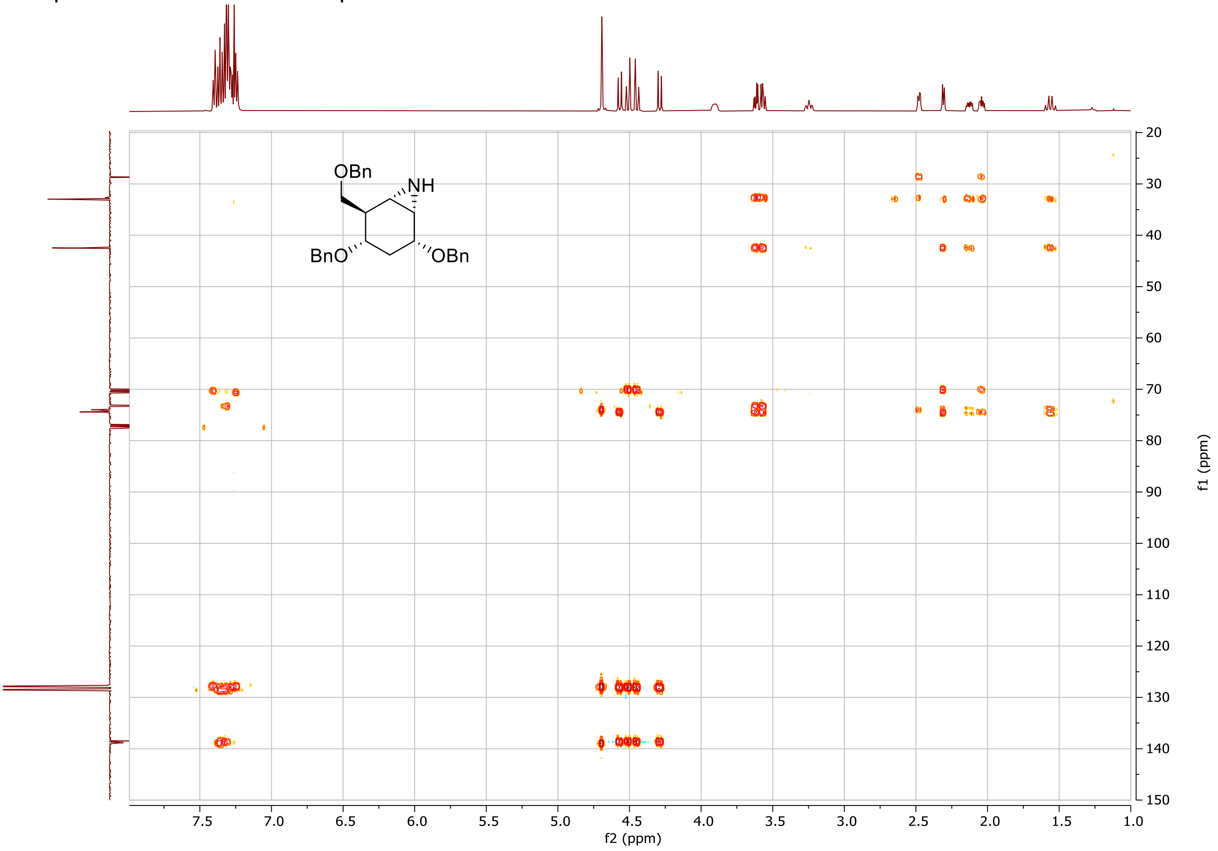

Compound **s78** ^{1}H NMR spectrum

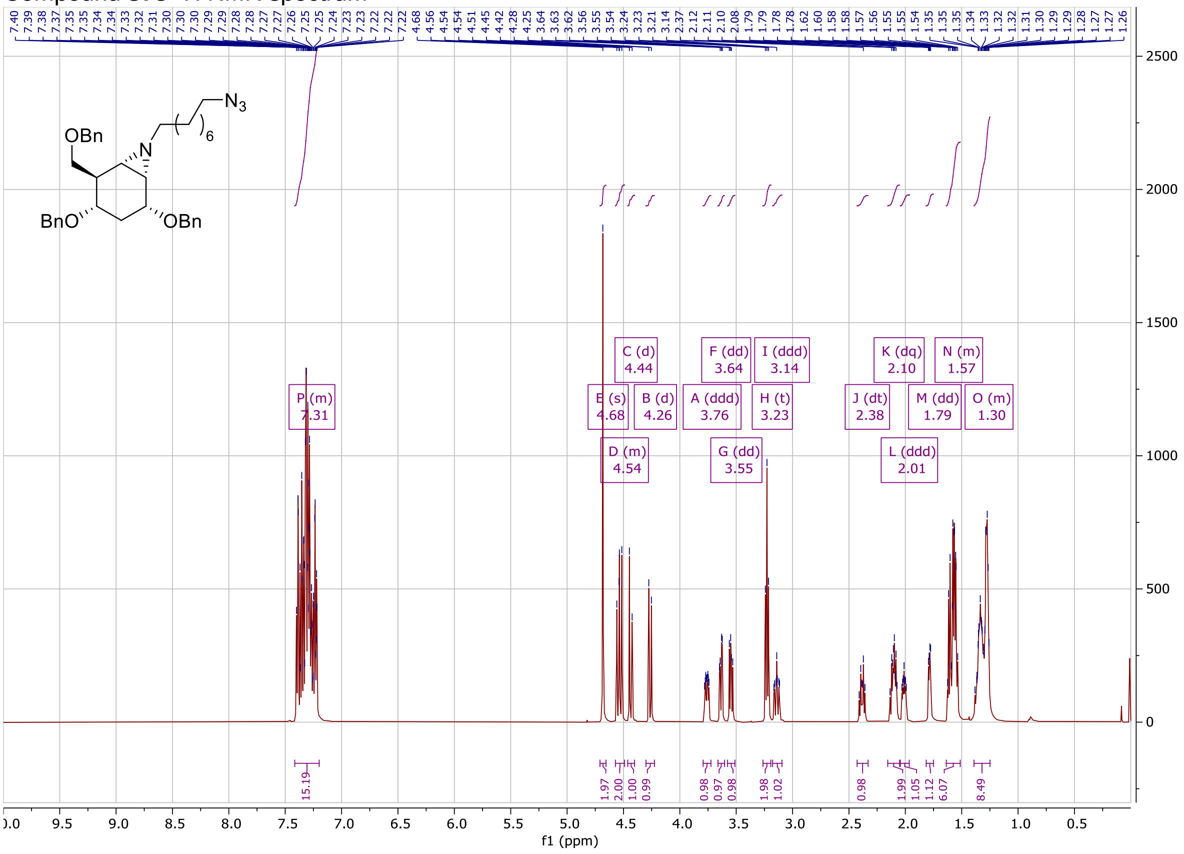

Compound **s78** ^{13}C NMR APT spectrum

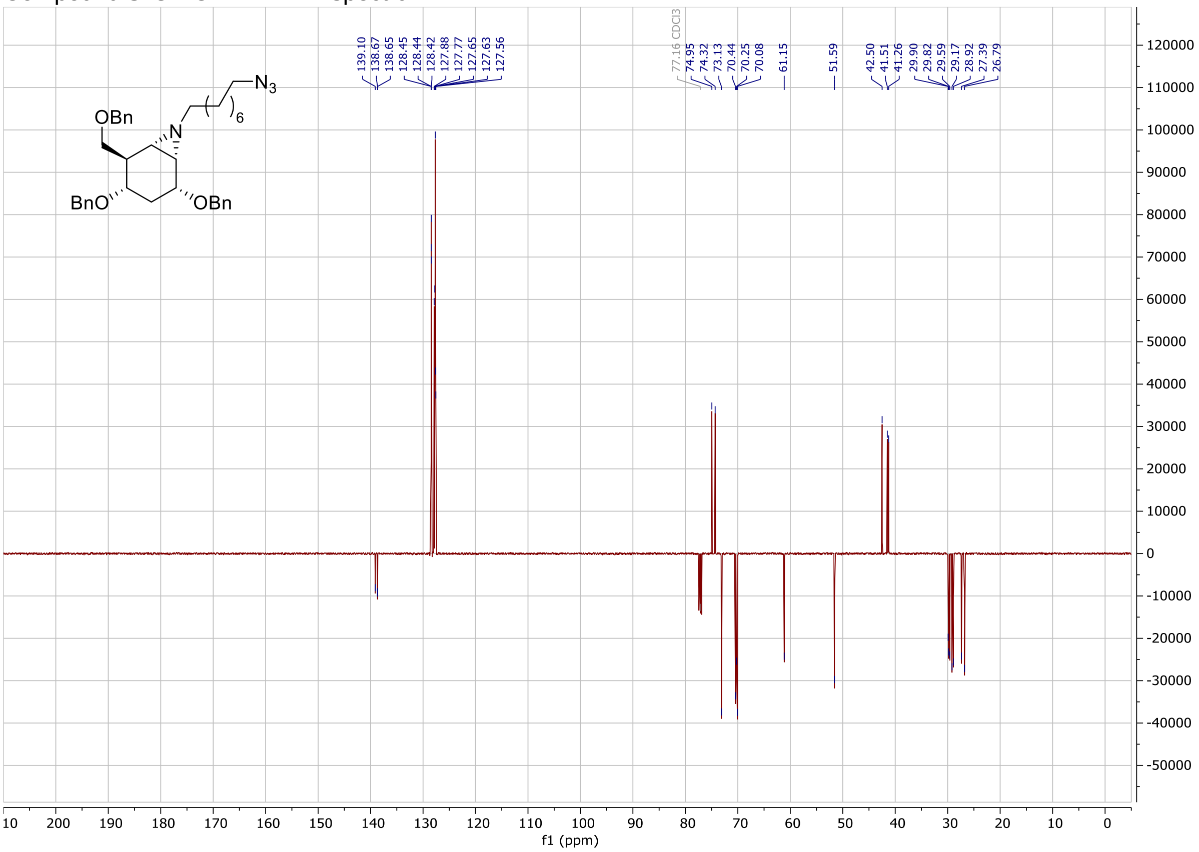

Compound **s78** ^{1}H-^{1}H COSY spectrum

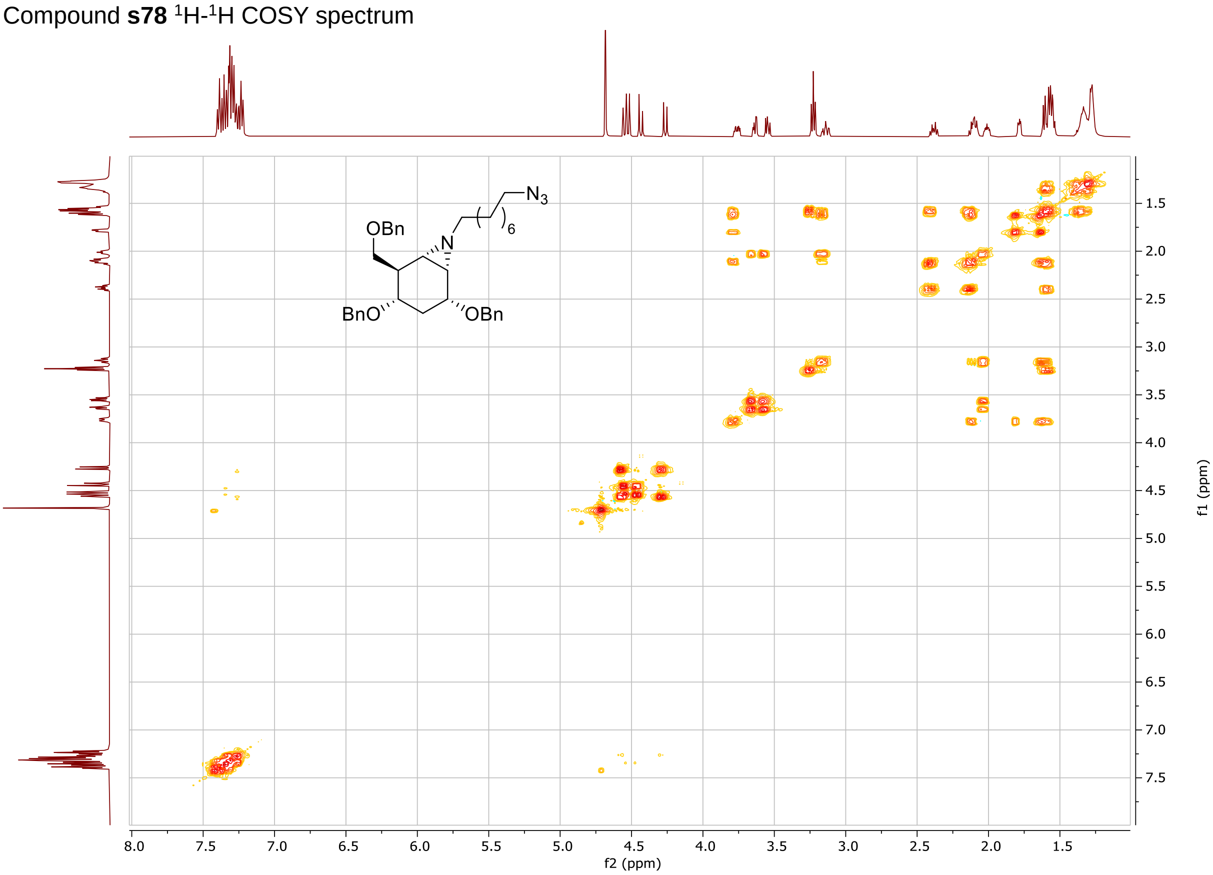

Compound **s78** ^{1}H-^{13}C HSQC spectrum

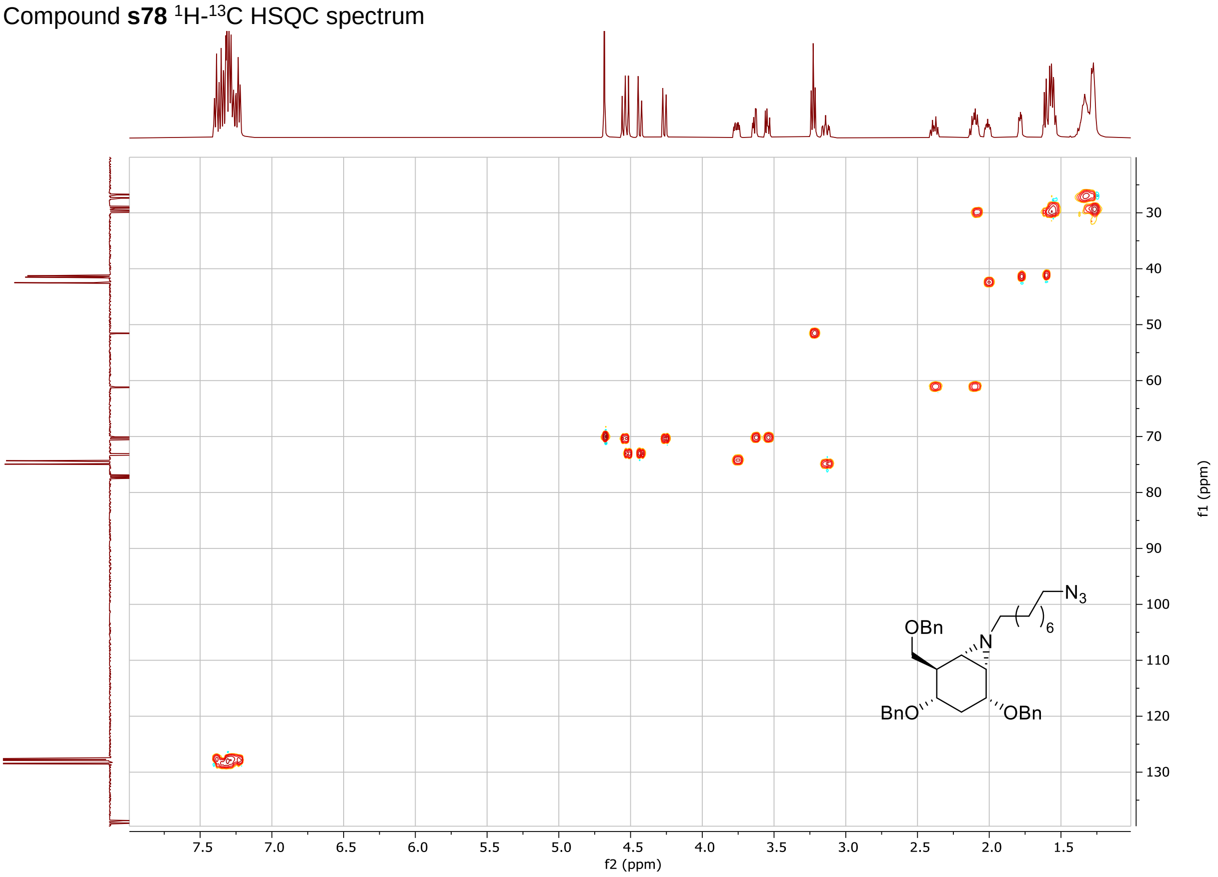

Compound **s78** ^{1}H-^{1}H NOESY spectrum

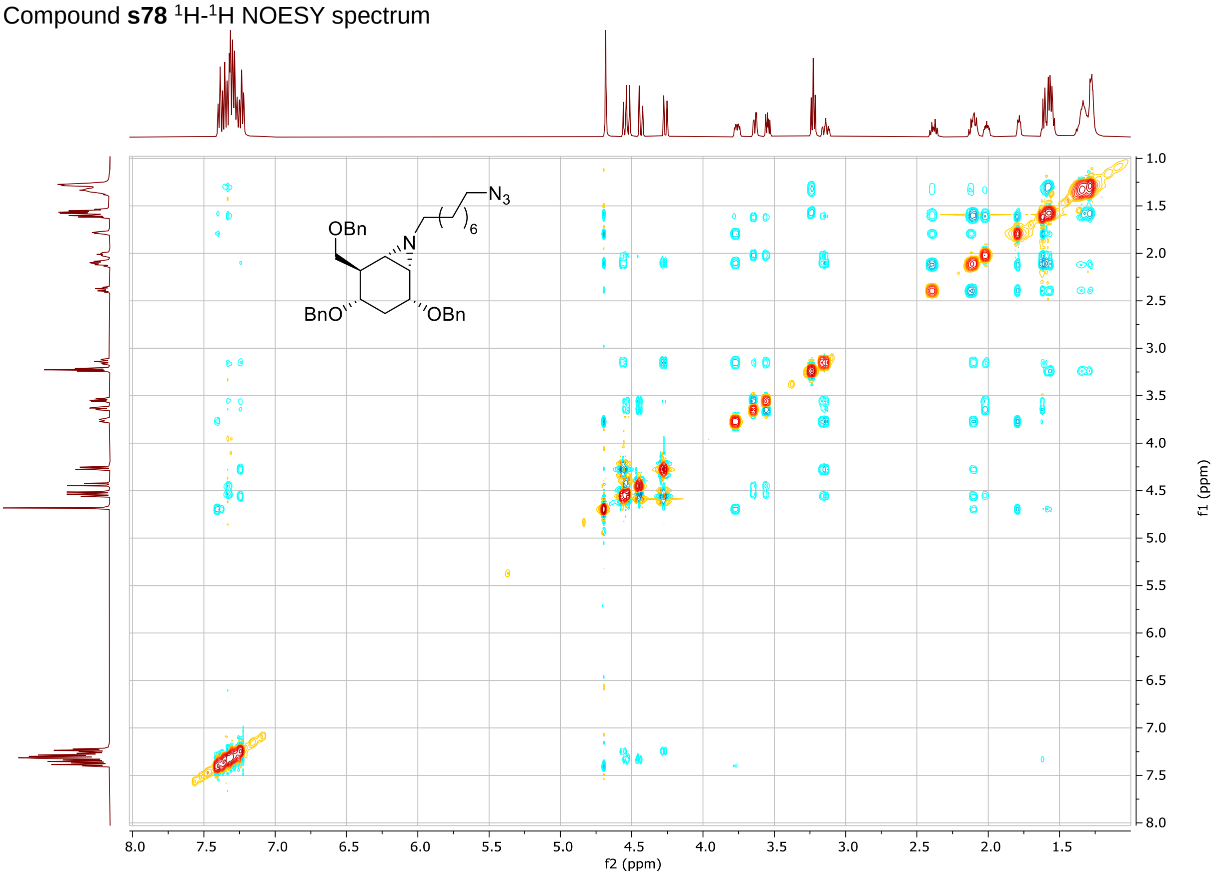

Compound **s78** ^{1}H-^{13}C HMBC spectrum

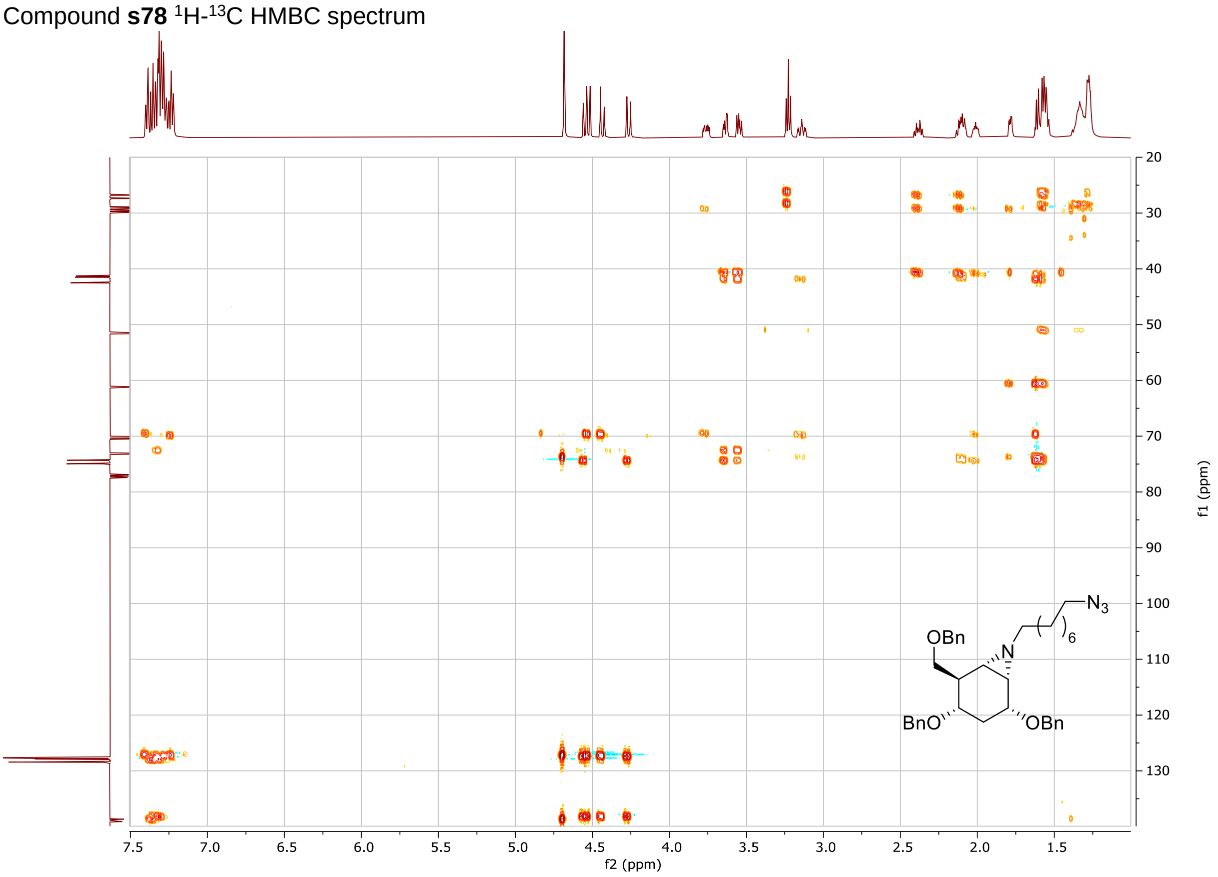

Compound **s79** ^{1}H NMR spectrum

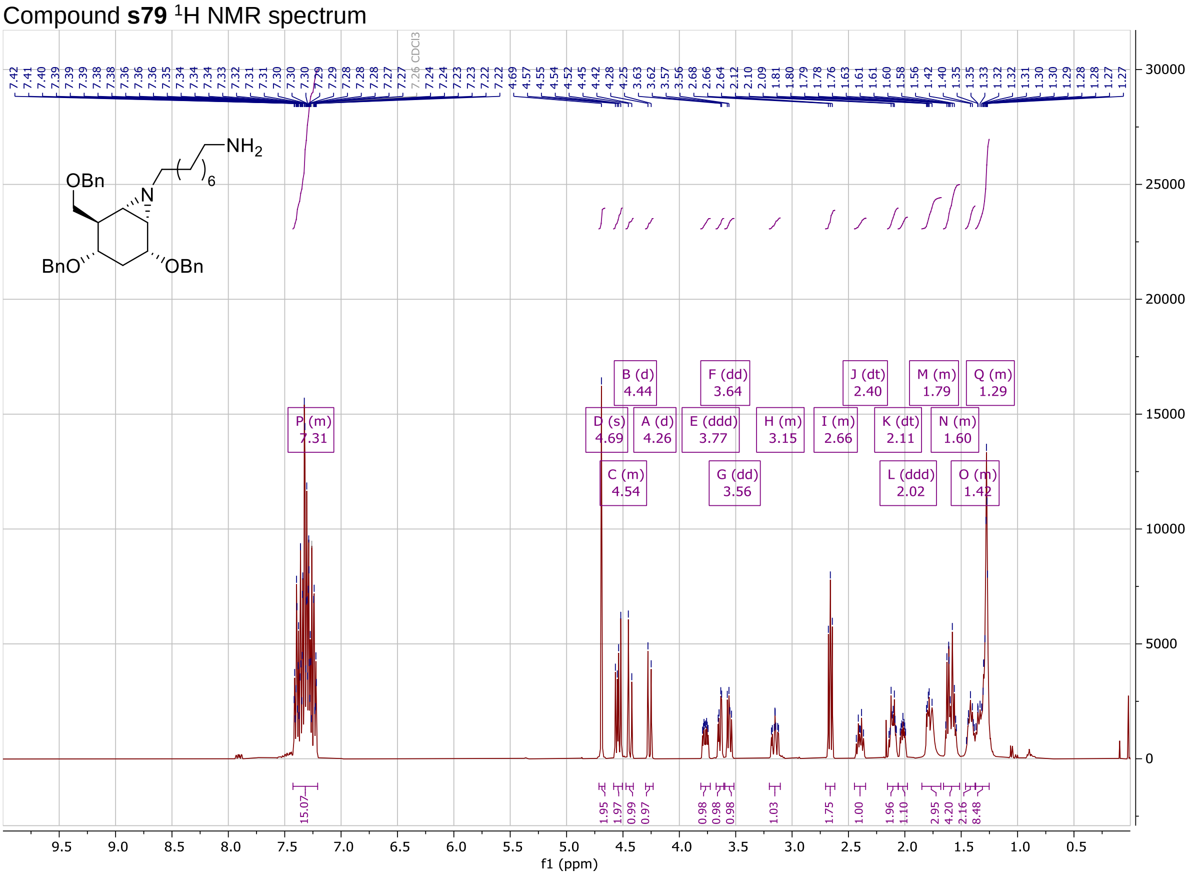

Compound **s79** ^{13}C NMR APT spectrum

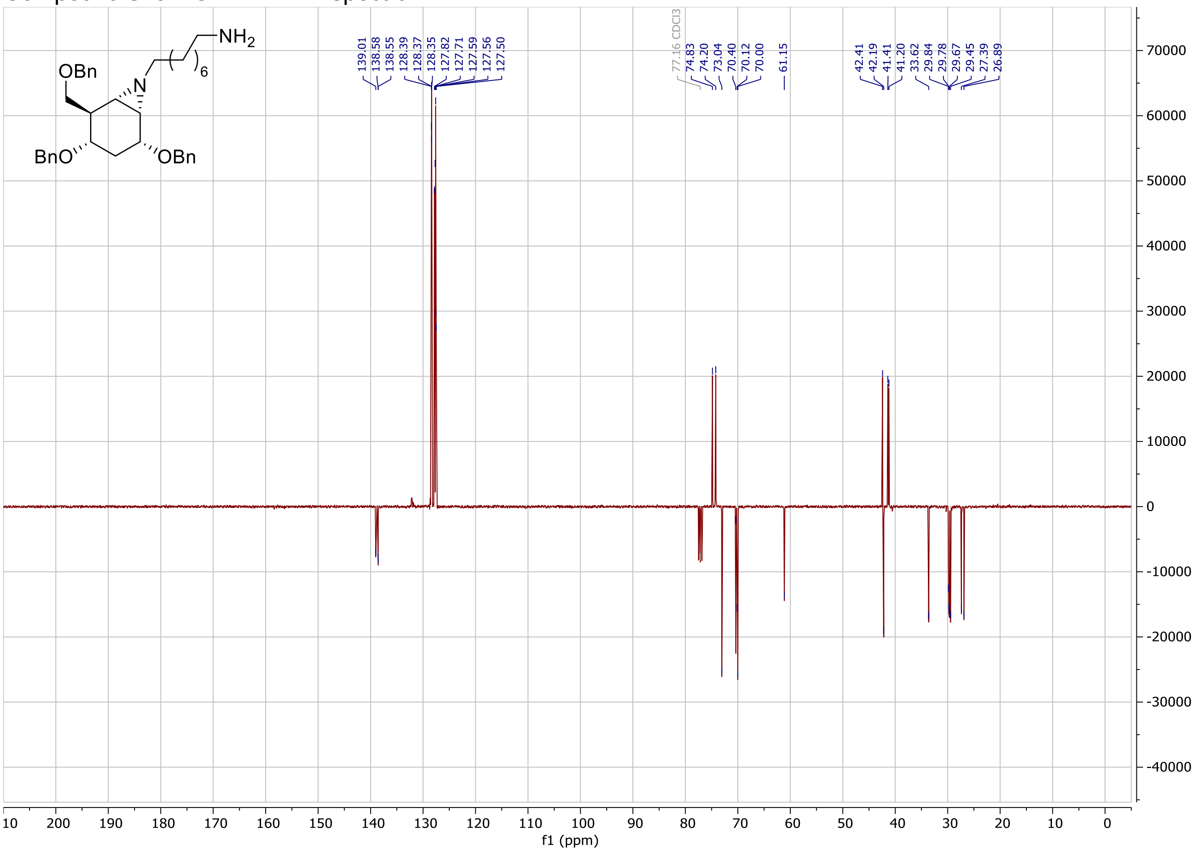

Compound **s79** ^{1}H-^{1}H COSY spectrum

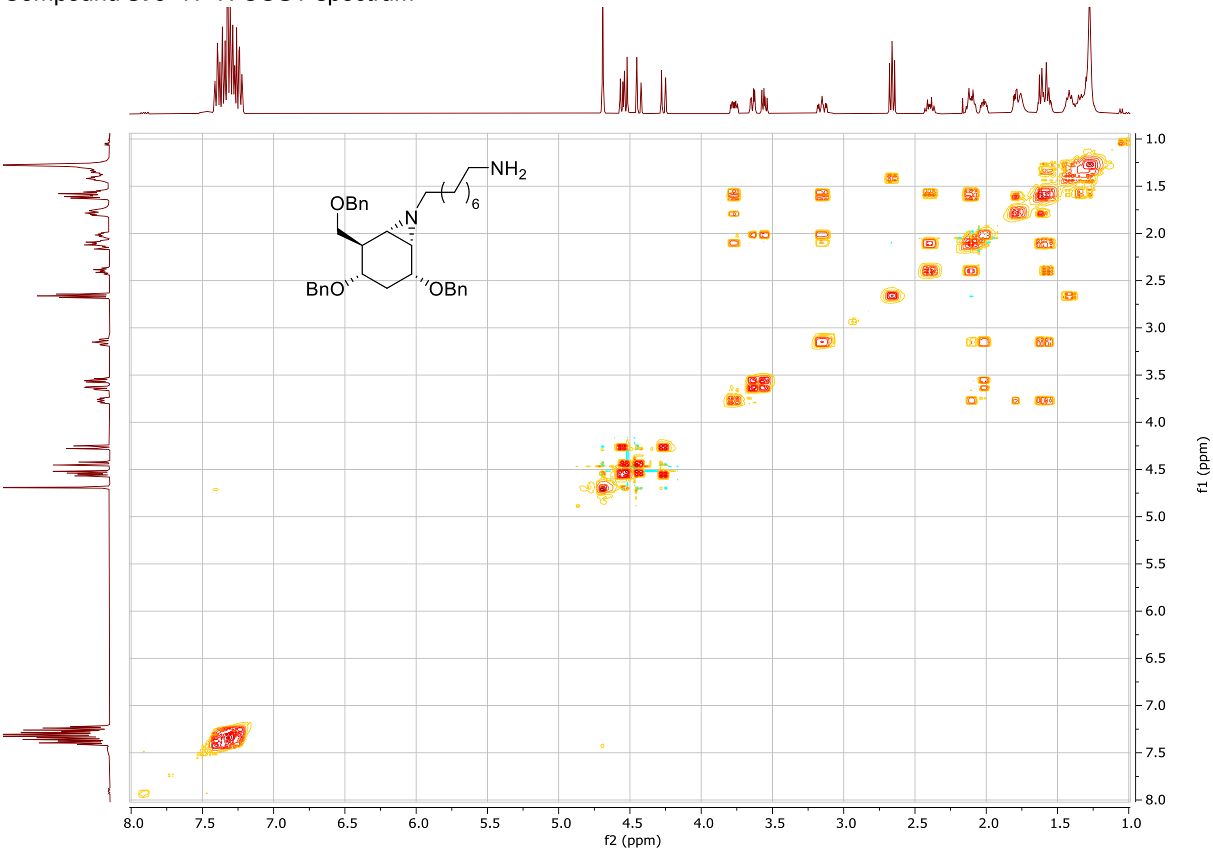

Compound **s79** ^{1}H-^{13}C HSQC spectrum

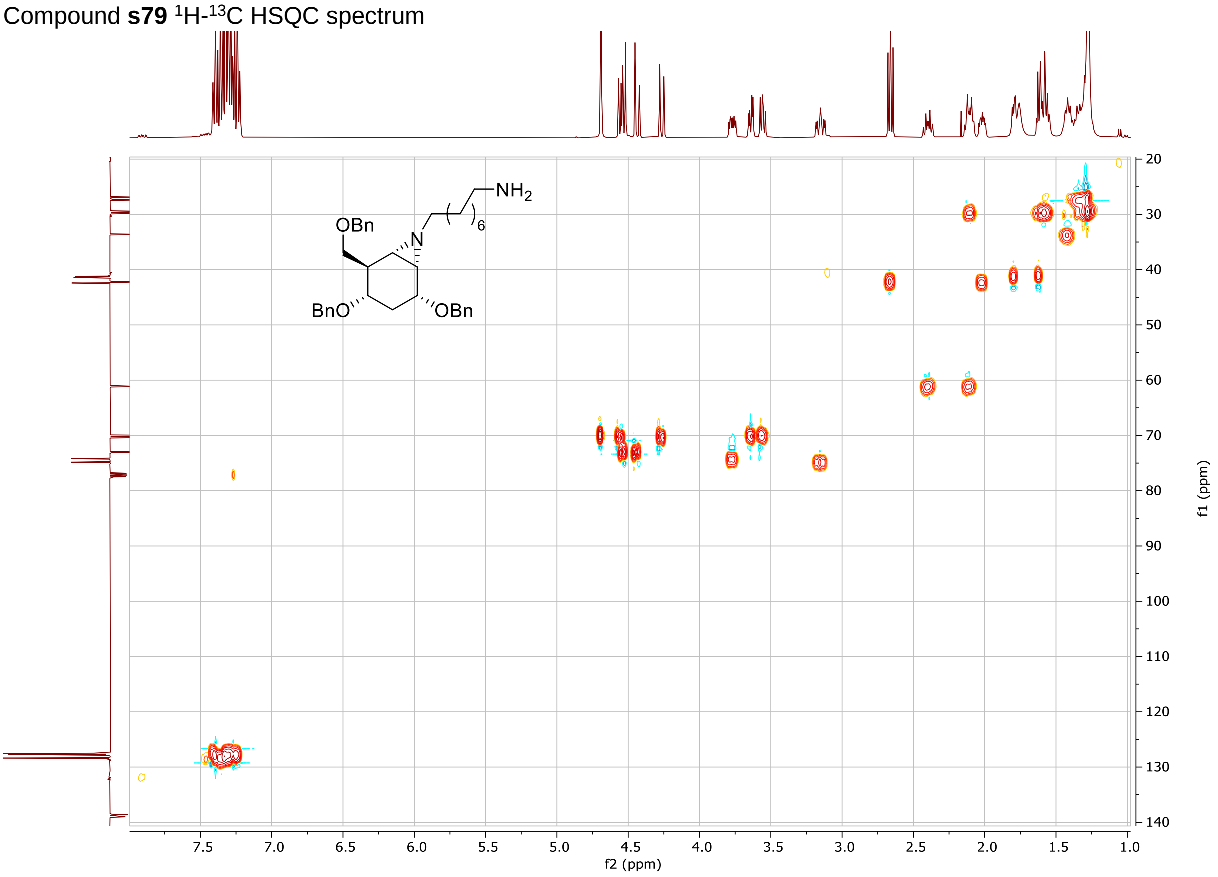

Compound **s80** ^{1}H NMR spectrum

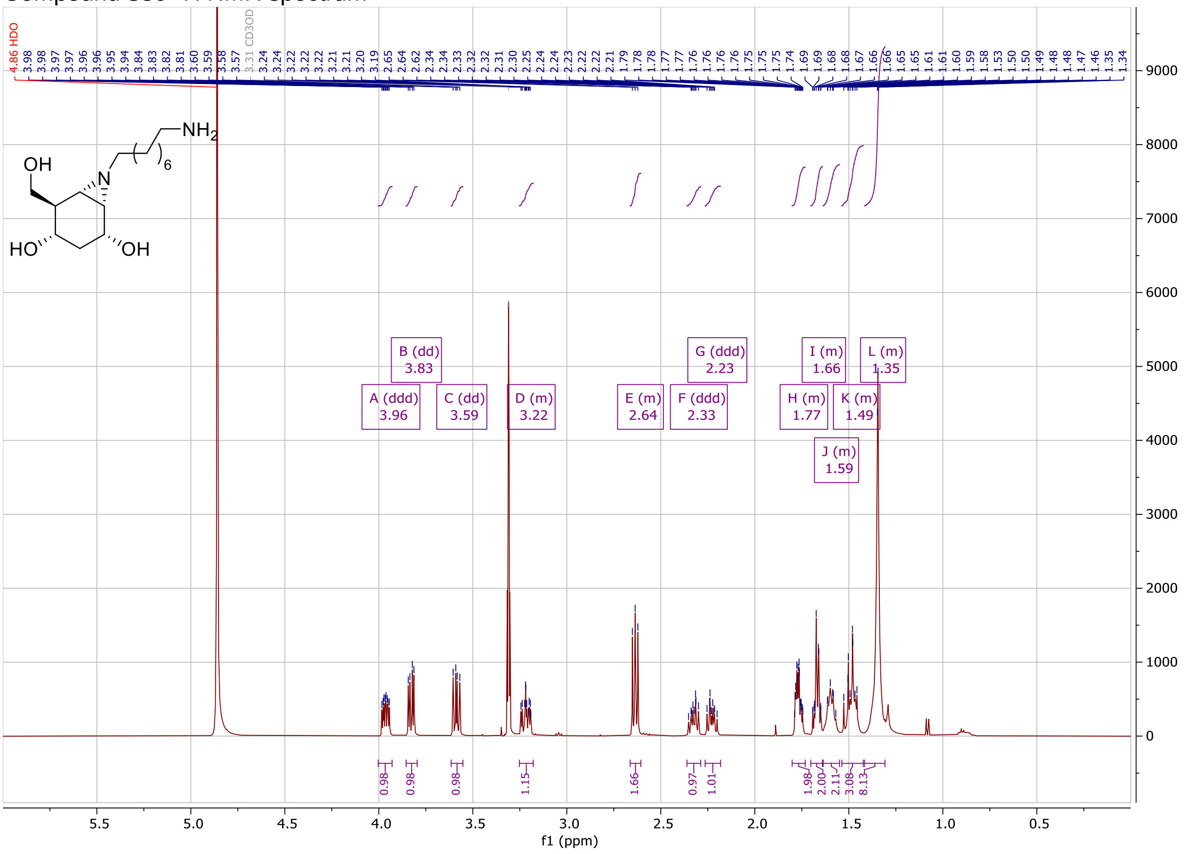

Compound **s80** ^{13}C NMR APT spectrum

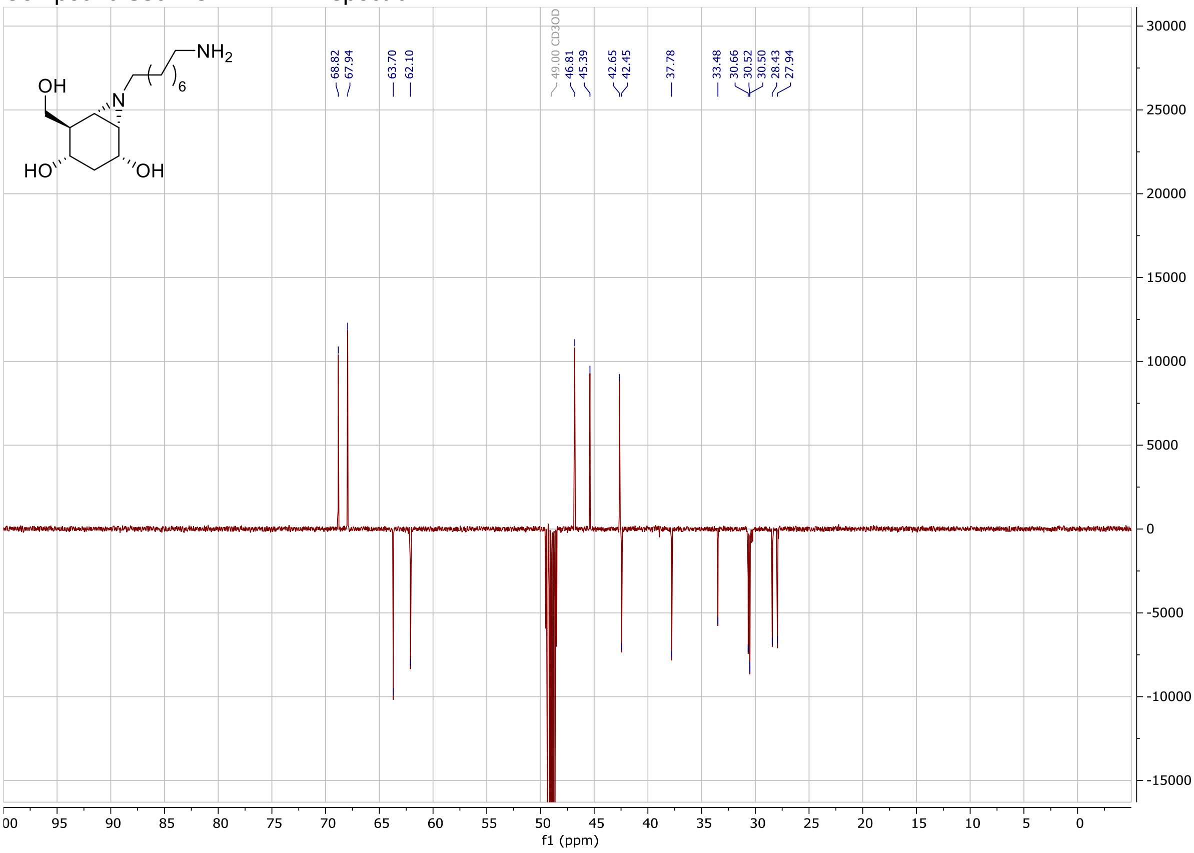

Compound **s80** ^{1}H-^{1}H COSY spectrum

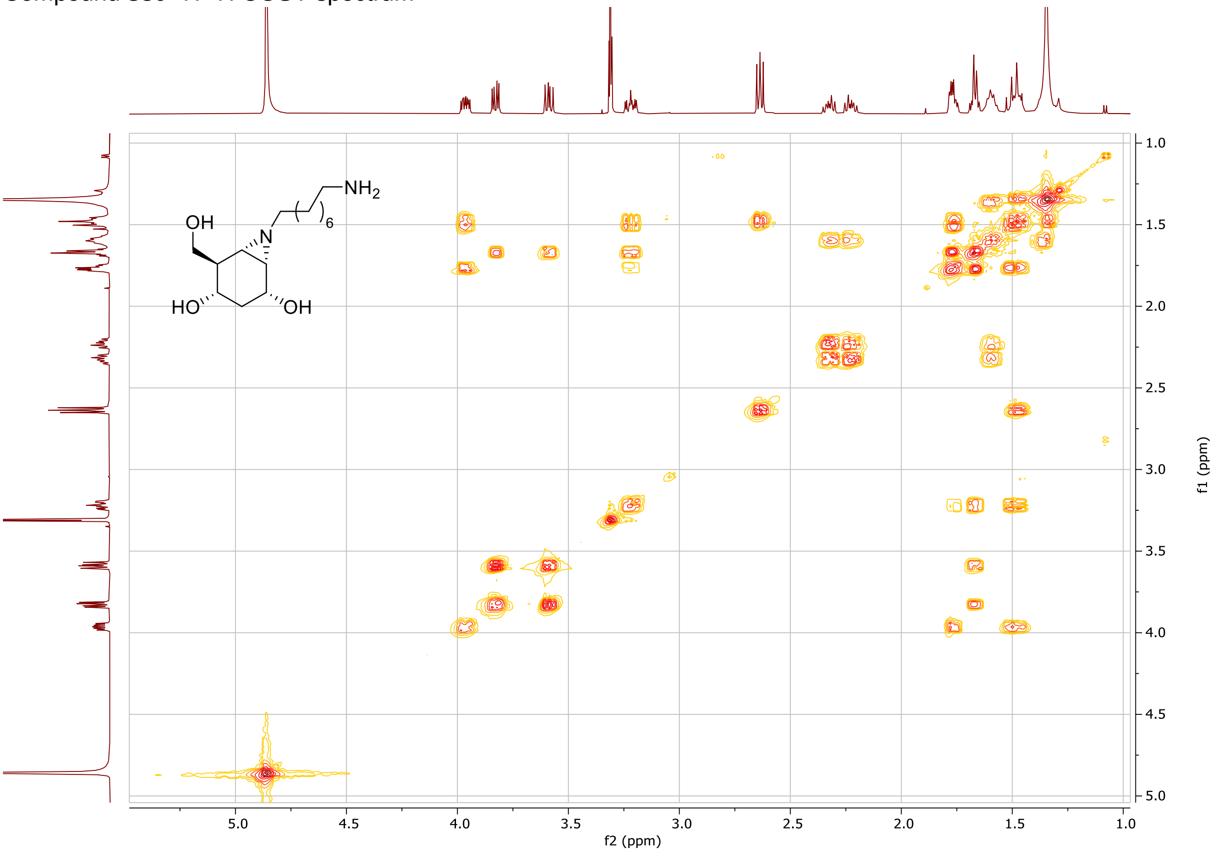

Compound **s80** ^{1}H-^{13}C HSQC spectrum

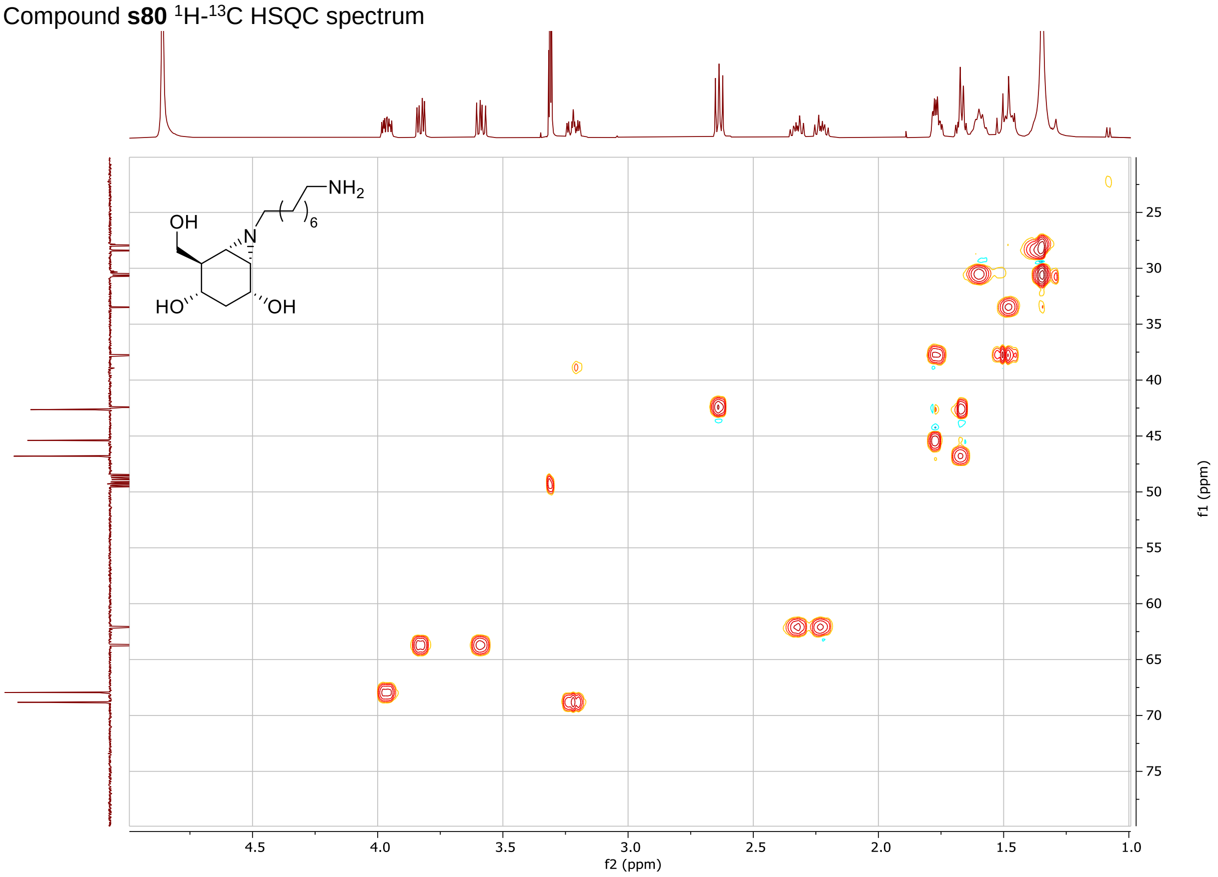

Compound **51** ^{1}H NMR spectrum

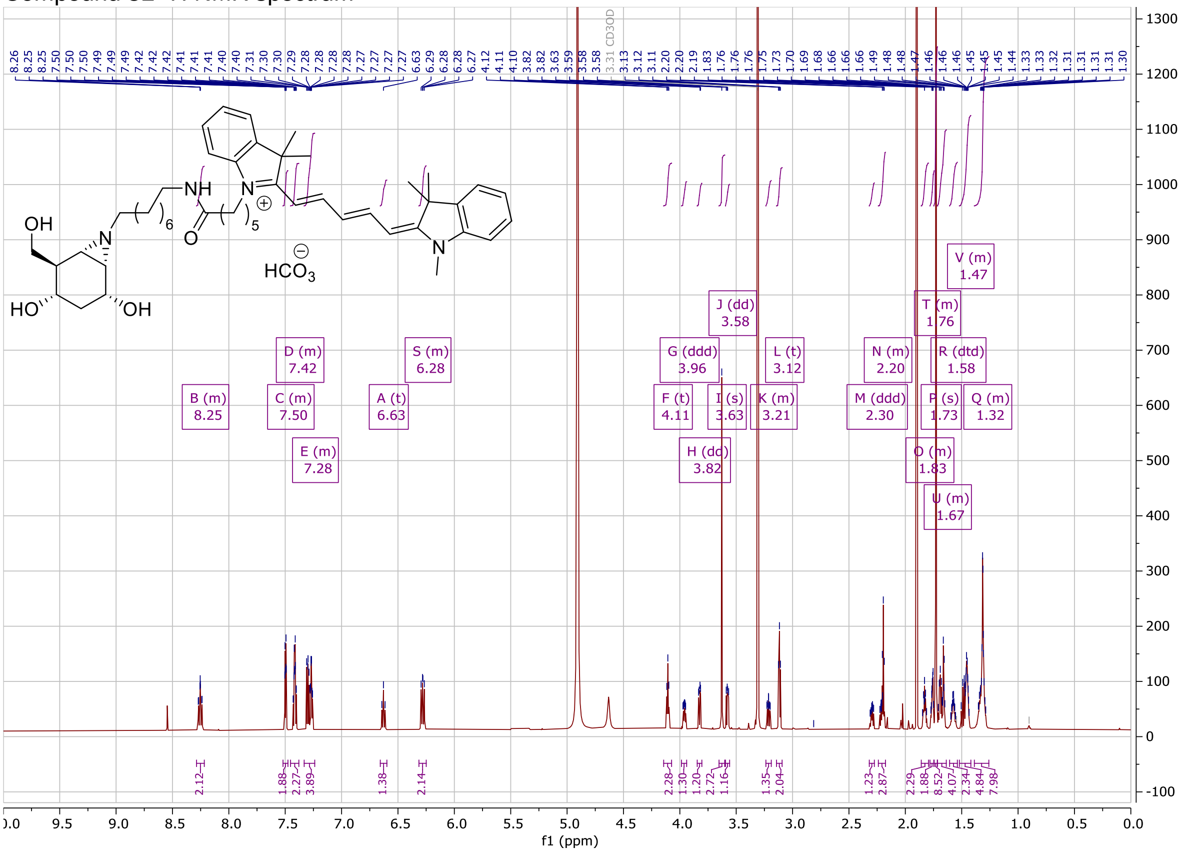

Compound **51** ^{13}C NMR APT spectrum

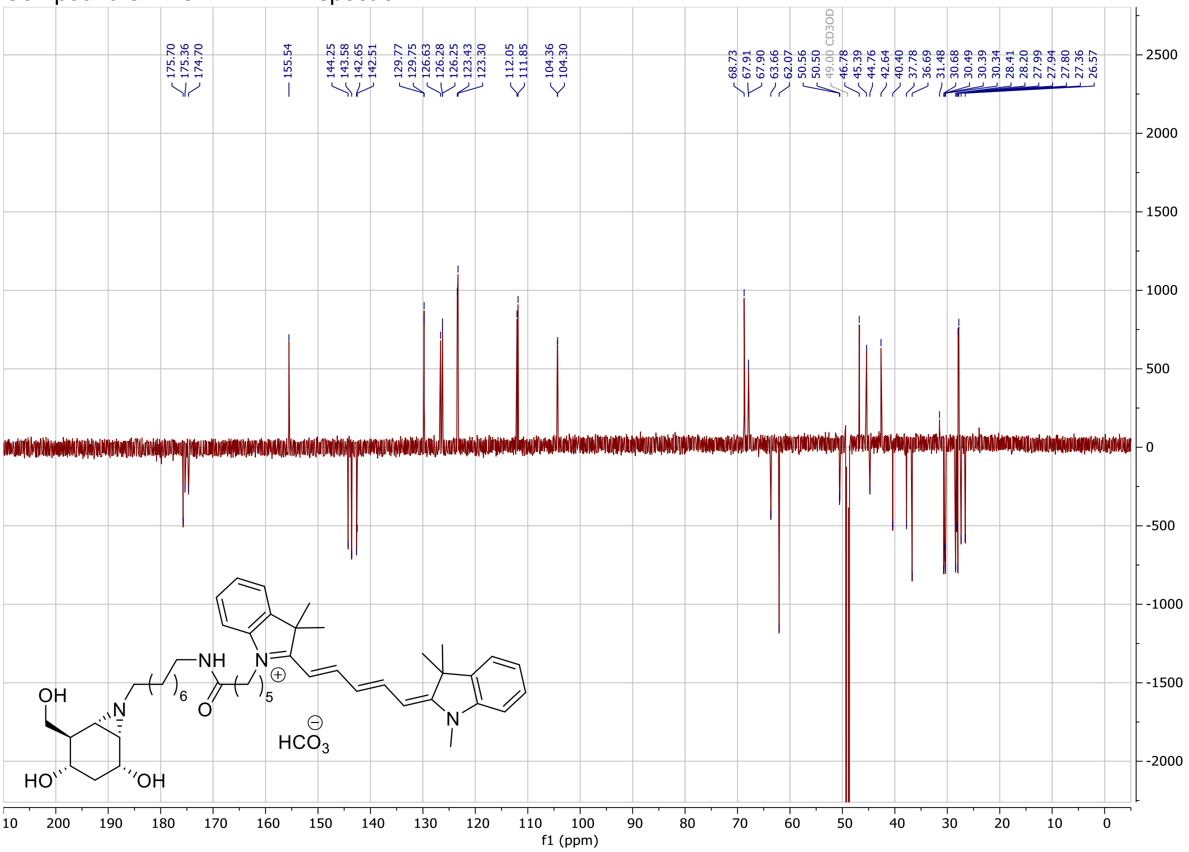

Compound **51** ^{1}H-^{1}H COSY spectrum

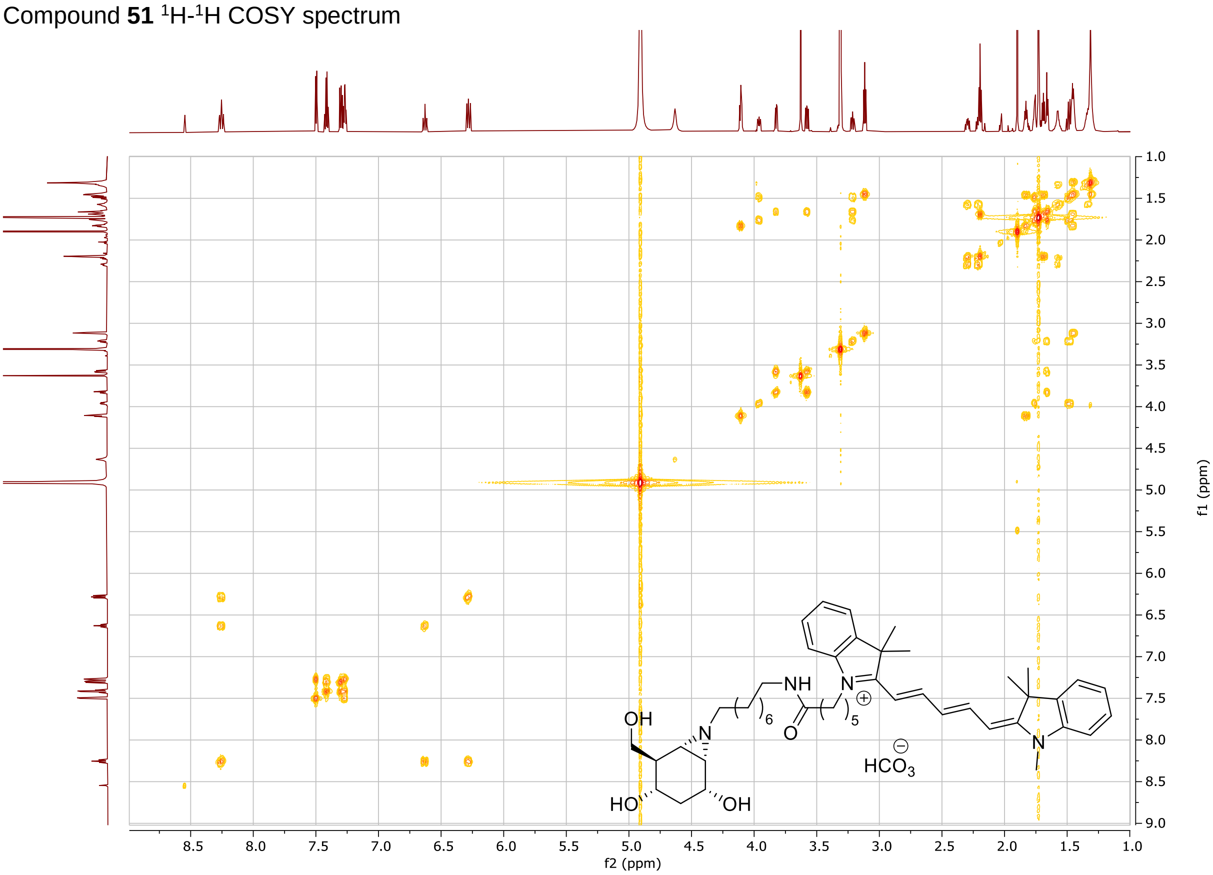

Compound **51** ^{1}H-^{13}C HSQC spectrum

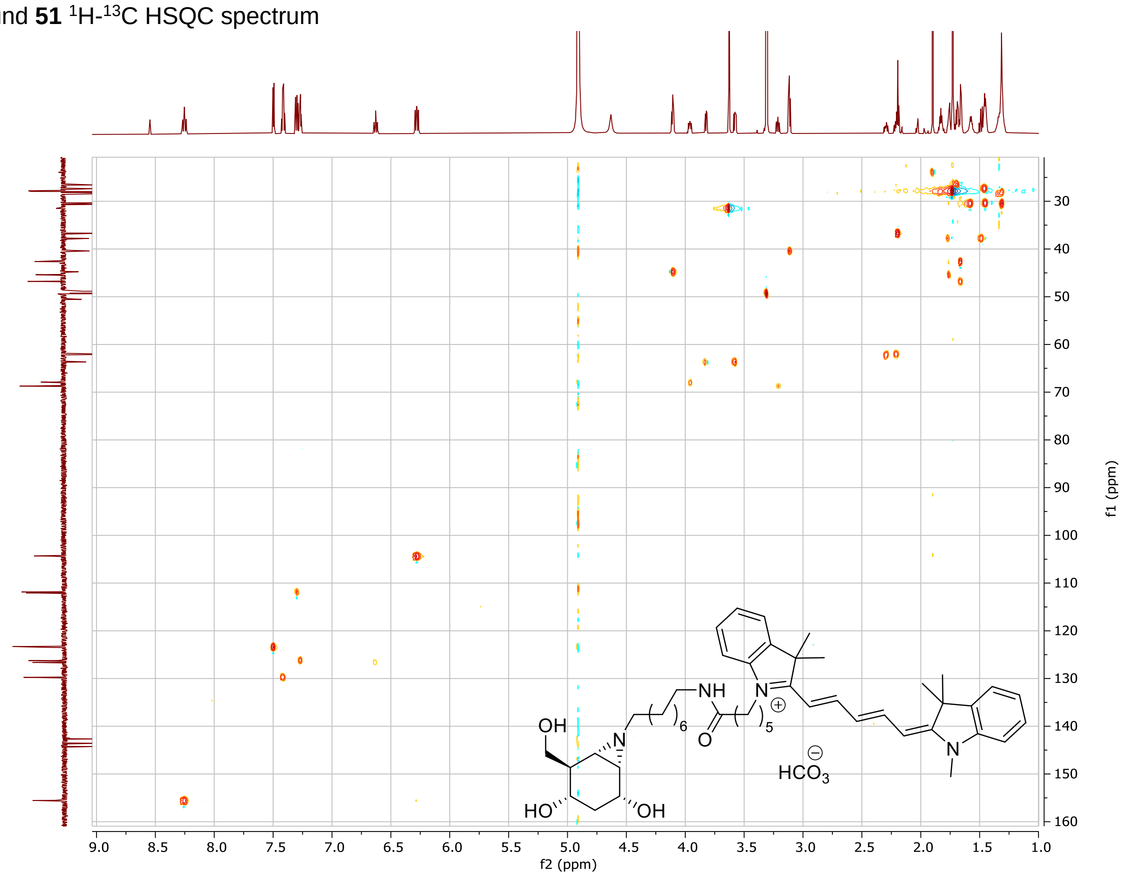

Compound **58** ^{1}H NMR spectrum

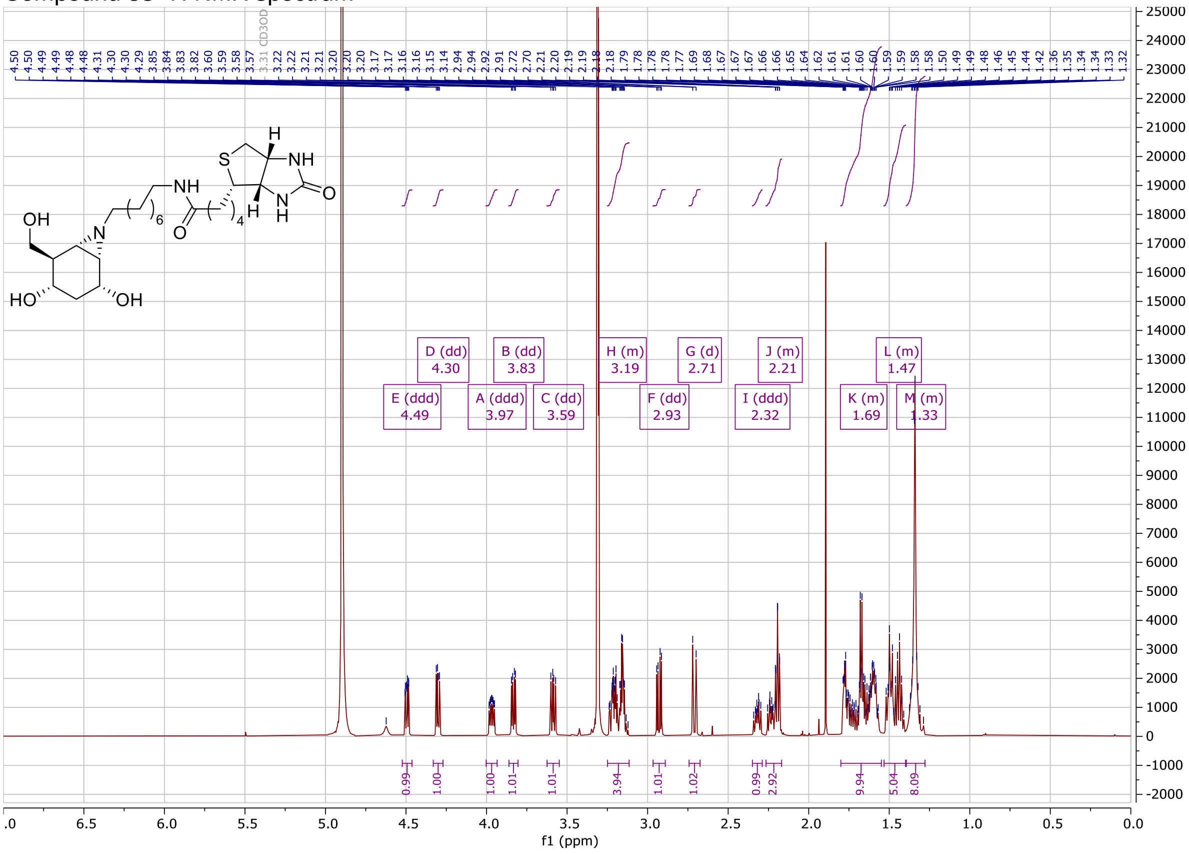

Compound **58** ^{13}C NMR APT spectrum

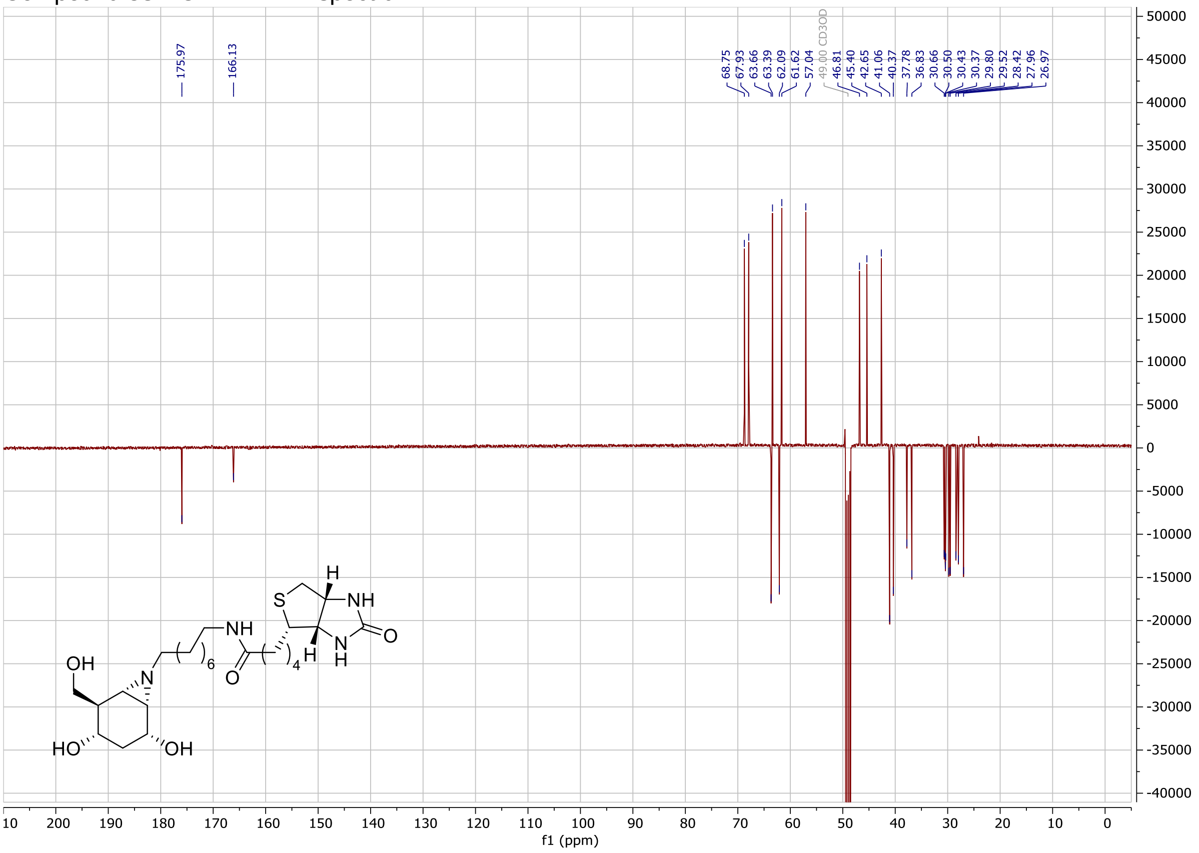

Compound **58** ^{1}H-^{1}H COSY spectrum

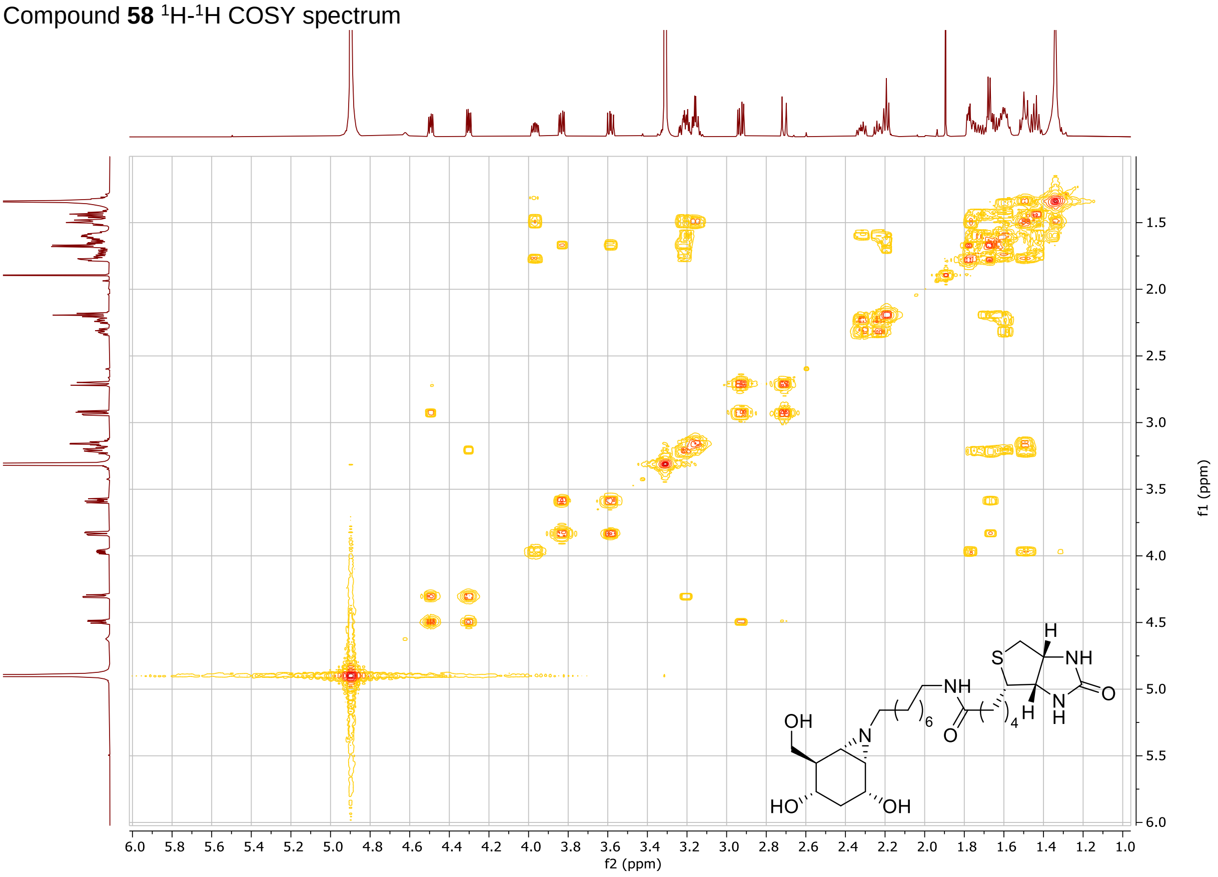

Compound **58** ^{1}H-^{13}C HSQC spectrum

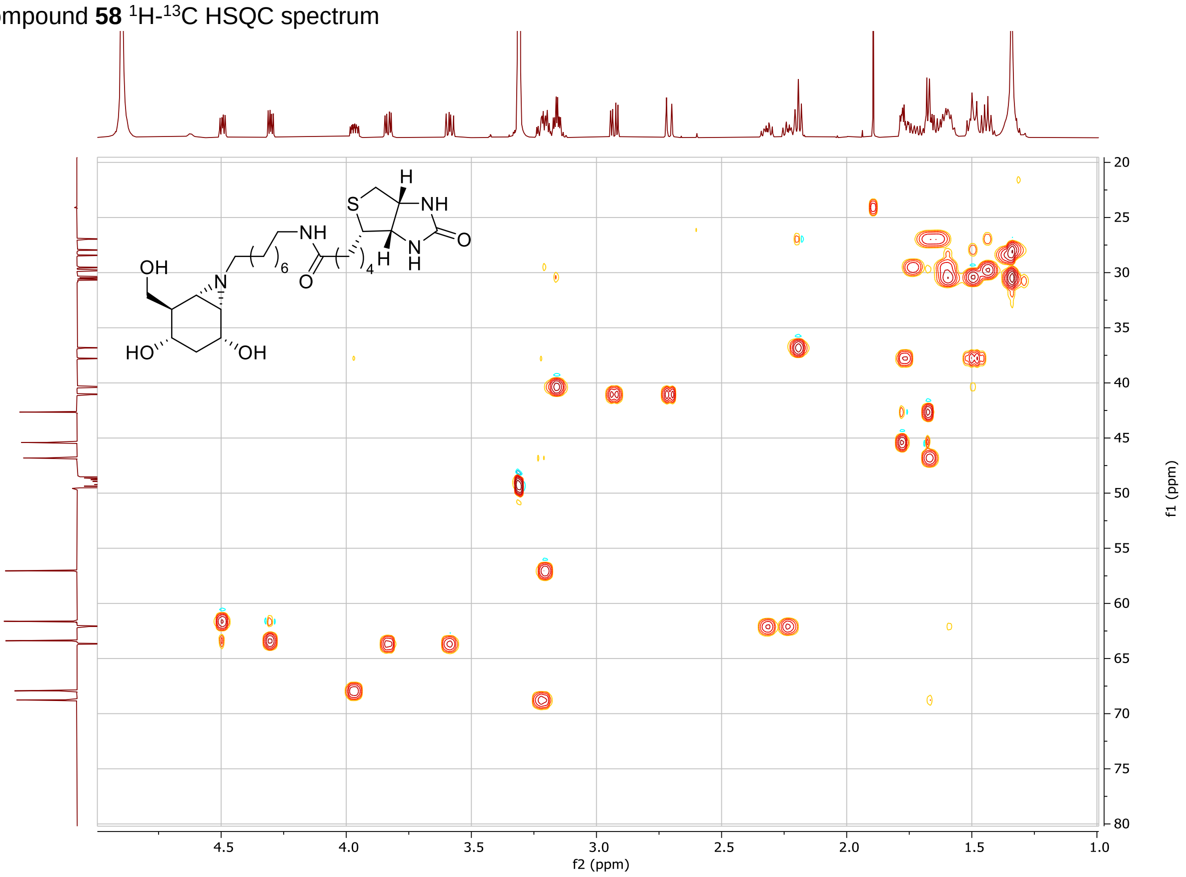

Compound **s81** ^{1}H NMR spectrum

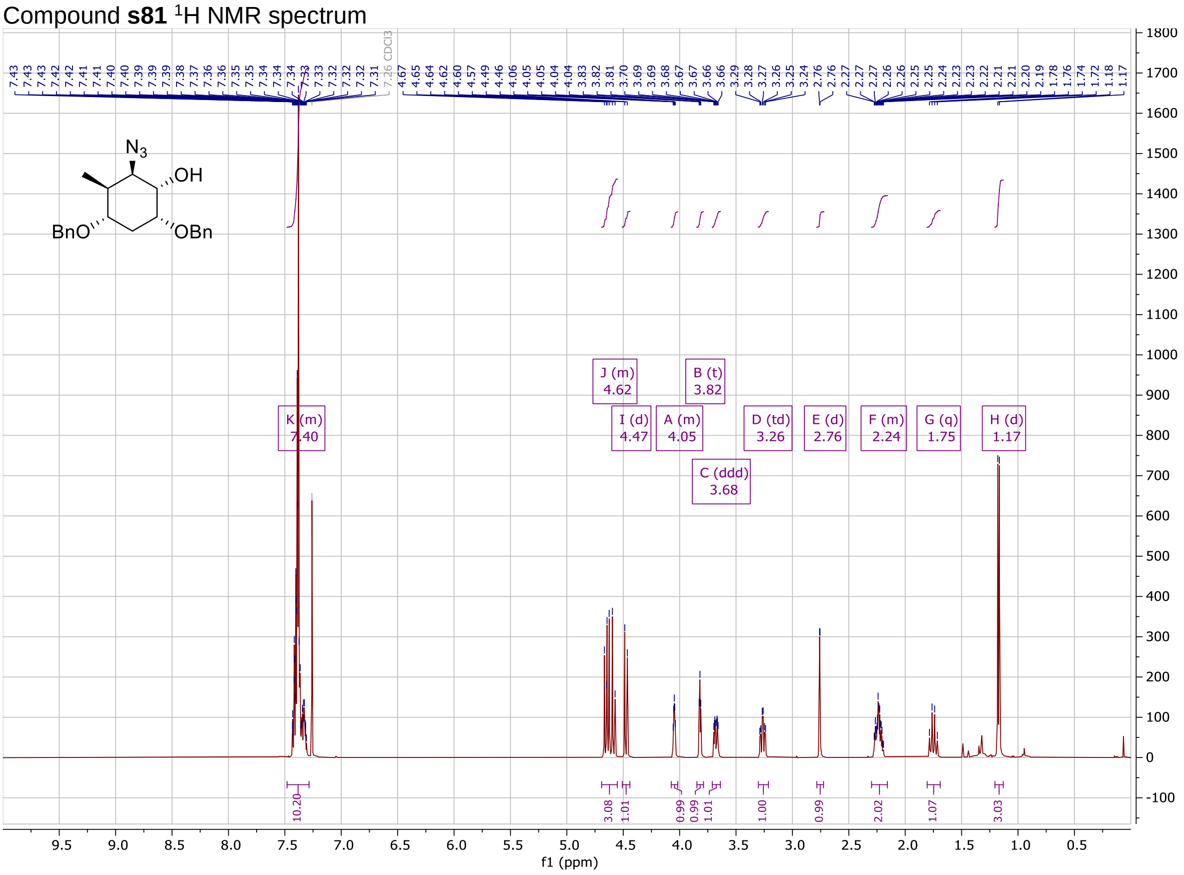

Compound **s81** ^{13}C NMR APT spectrum

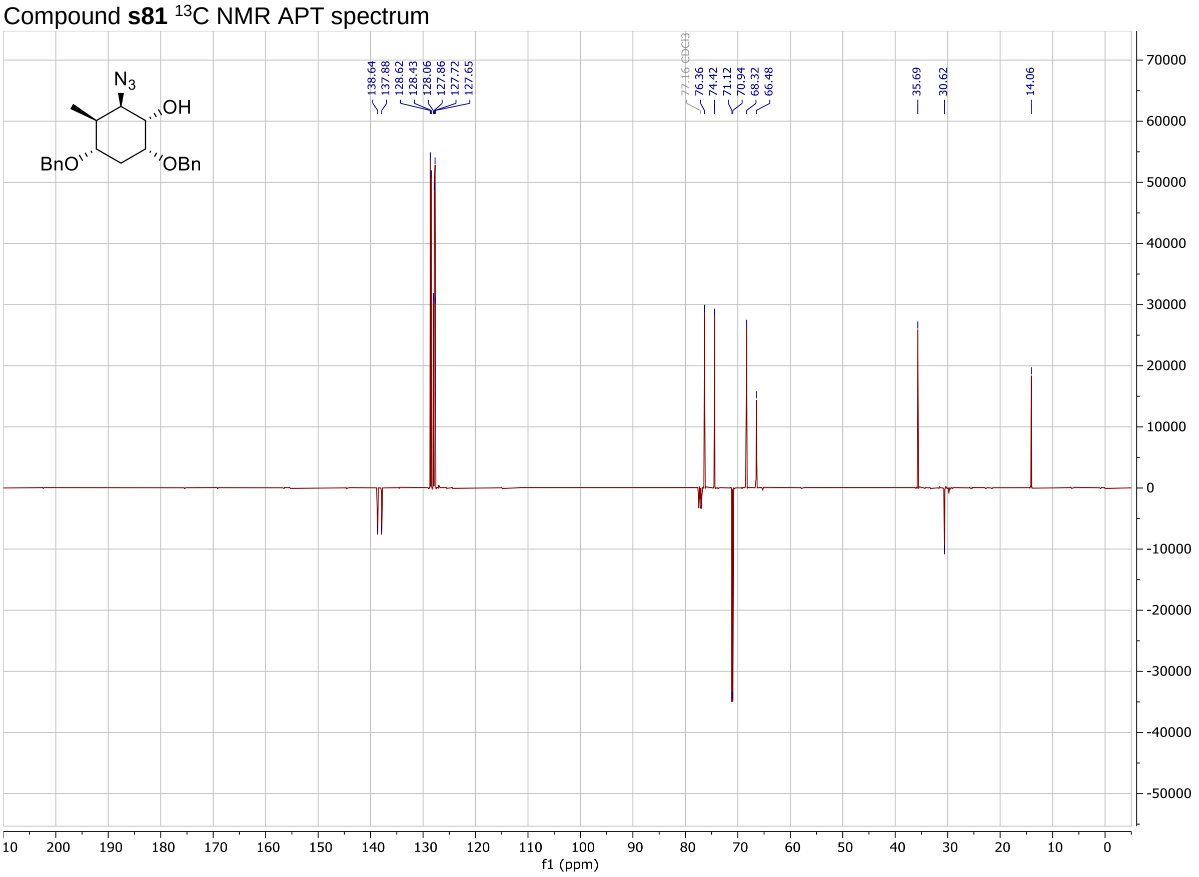

Compound **s81** ^{1}H-^{1}H COSY spectrum

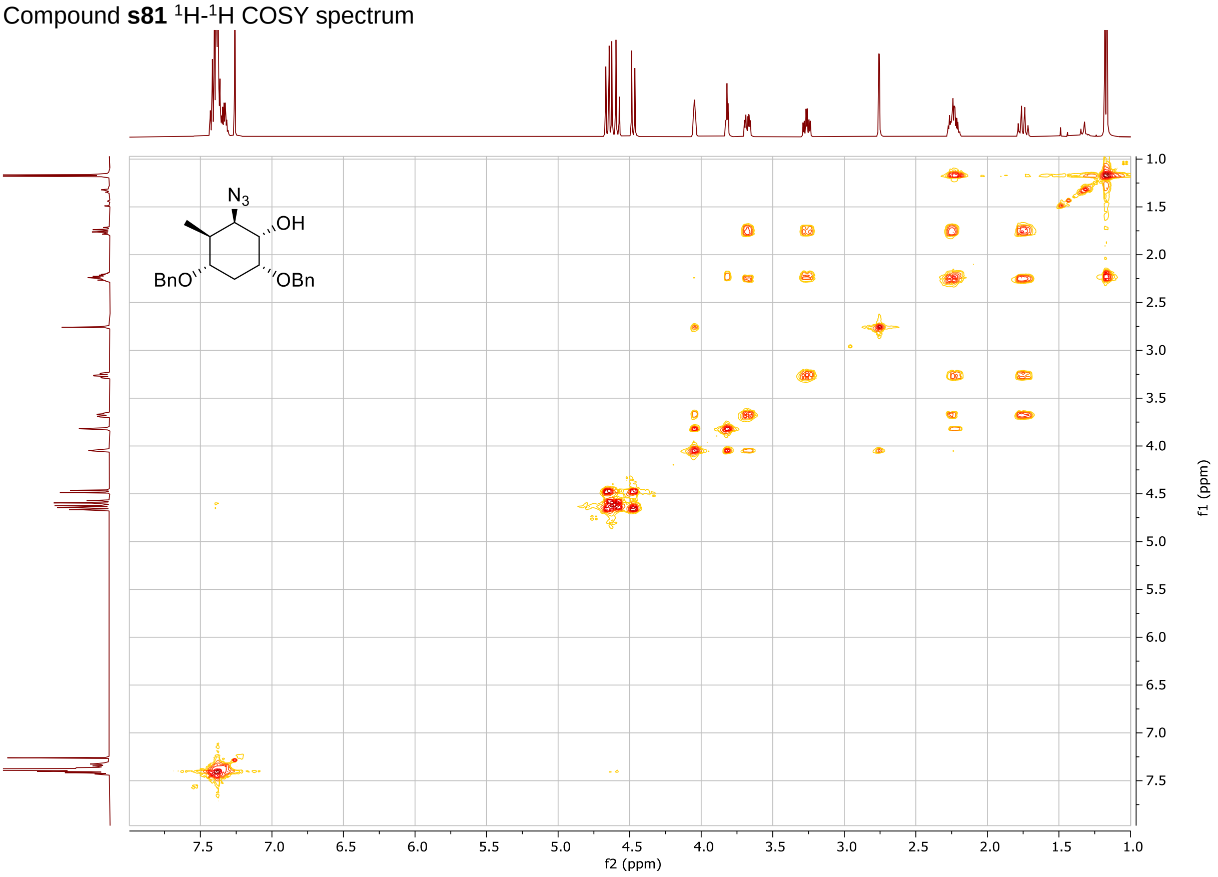

Compound **s81** ^{1}H-^{13}C HSQC spectrum

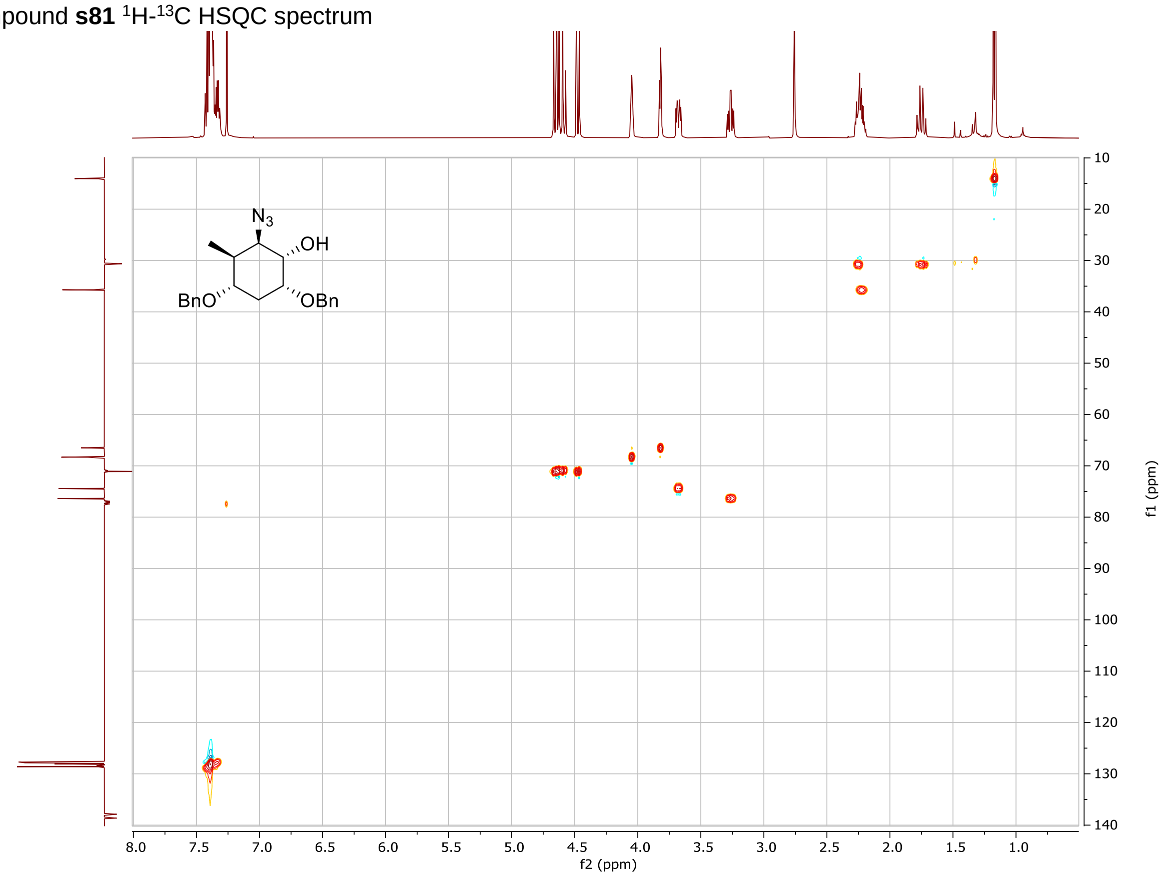

Compound **s81** ^{1}H-^{1}H NOESY spectrum

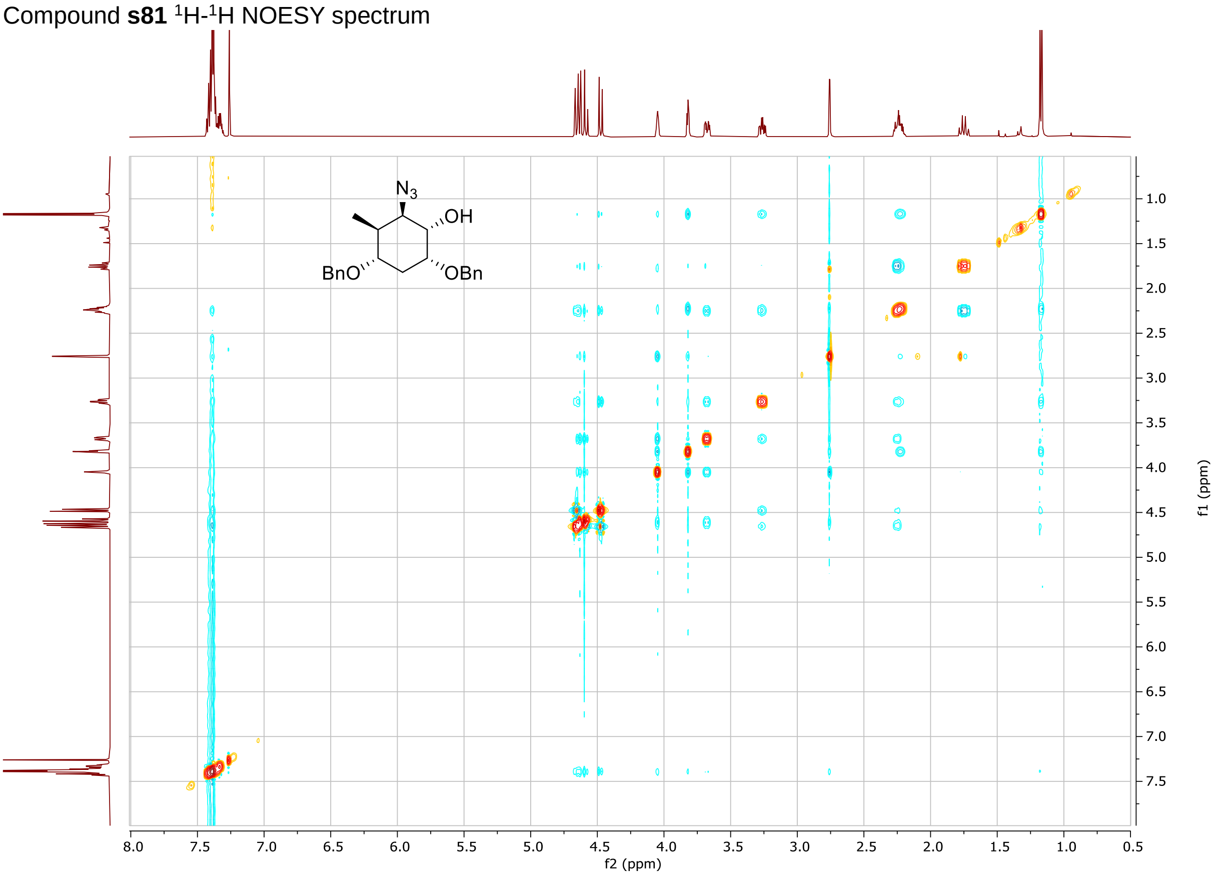

Compound **s82** ^{1}H NMR spectrum

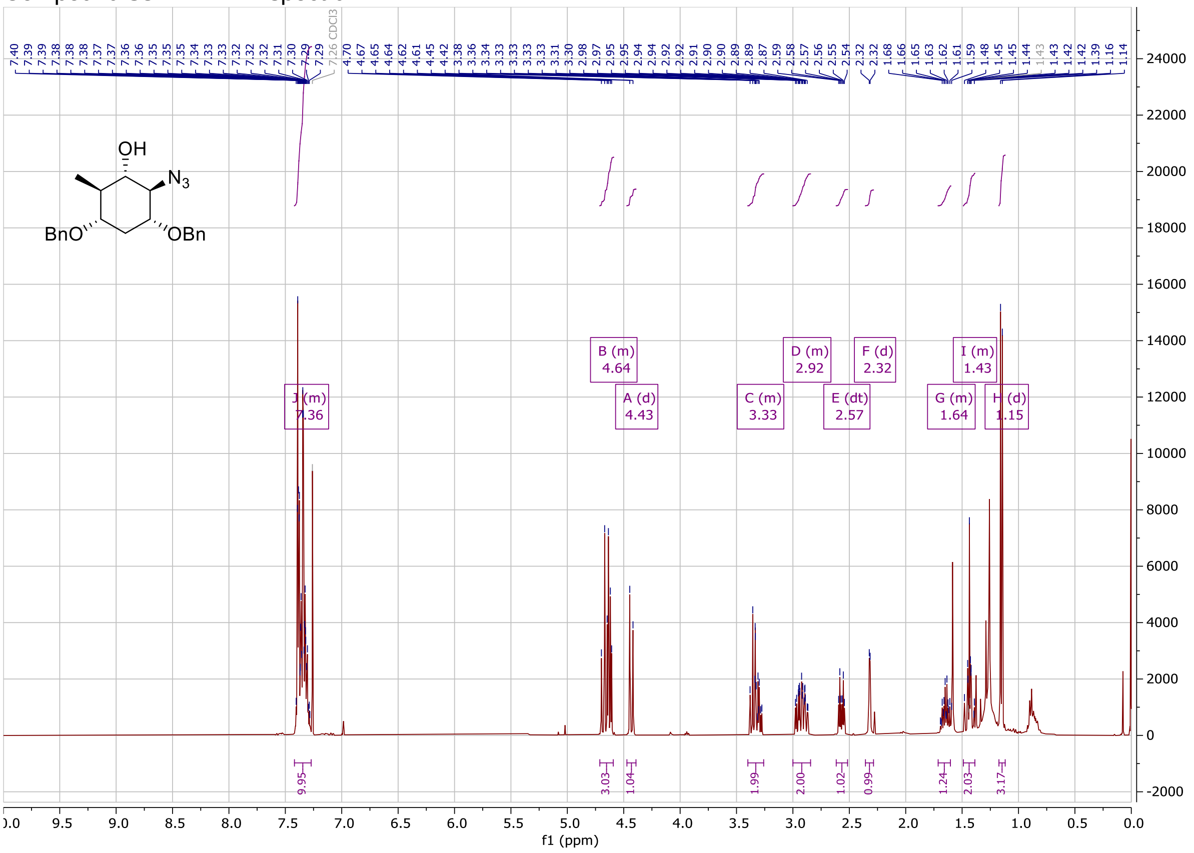

Compound **s82** ^{13}C NMR APT spectrum

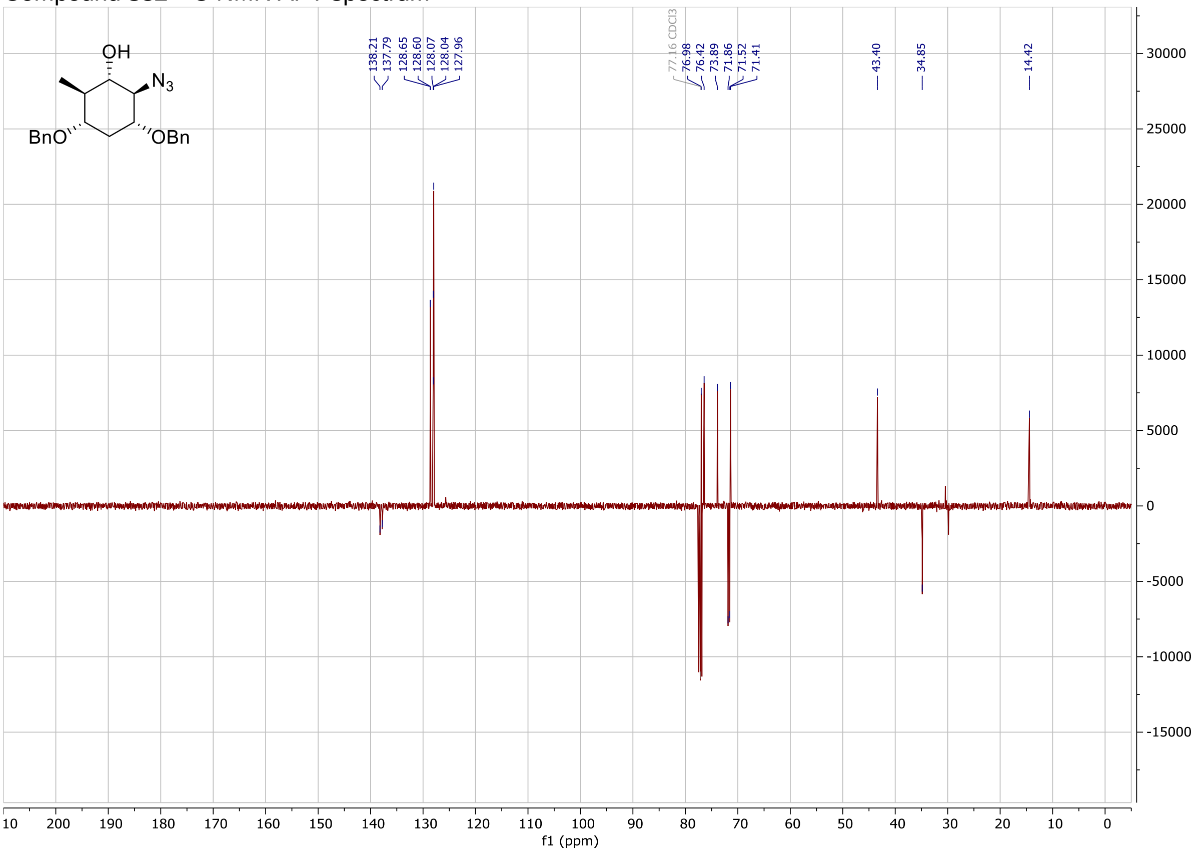

Compound **s82** ^{1}H-^{13}C HSQC spectrum

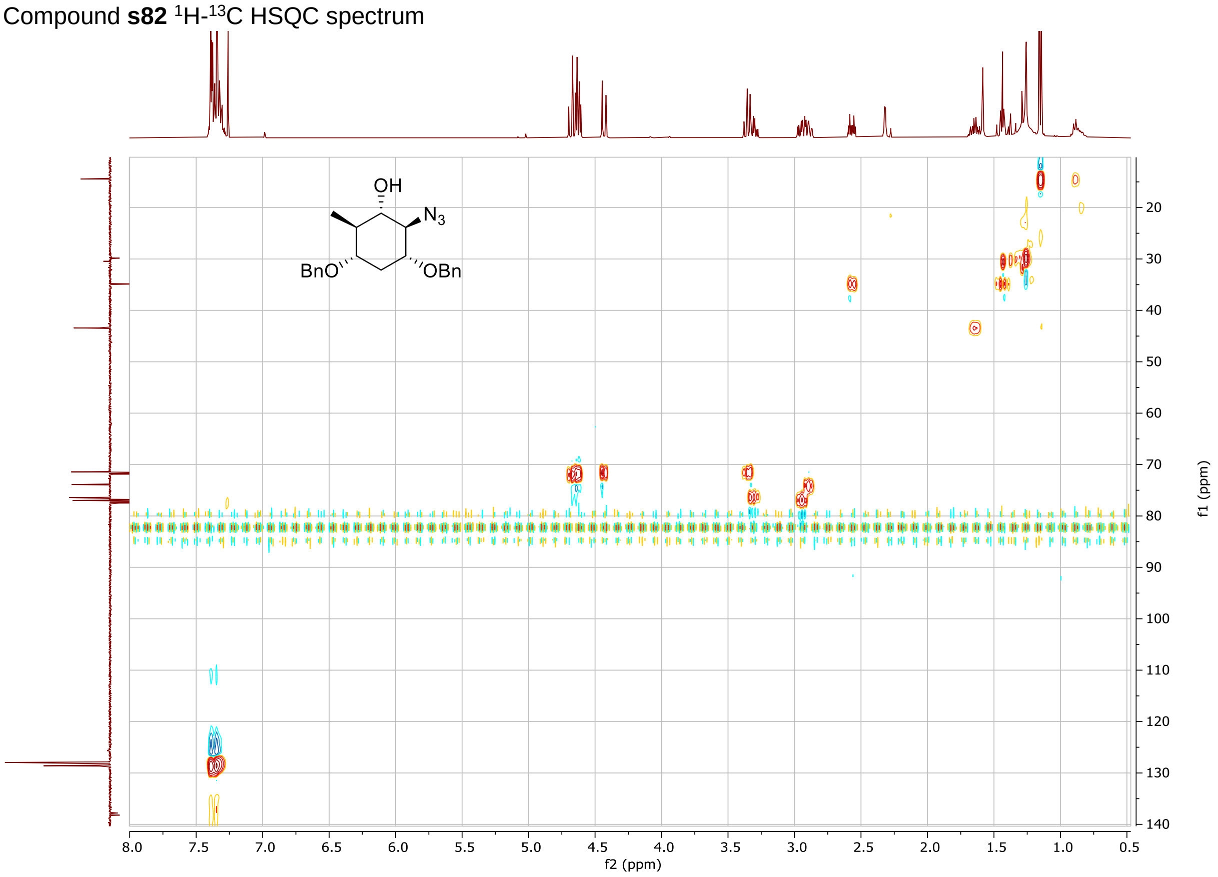

Compound **s84** ^{1}H NMR spectrum

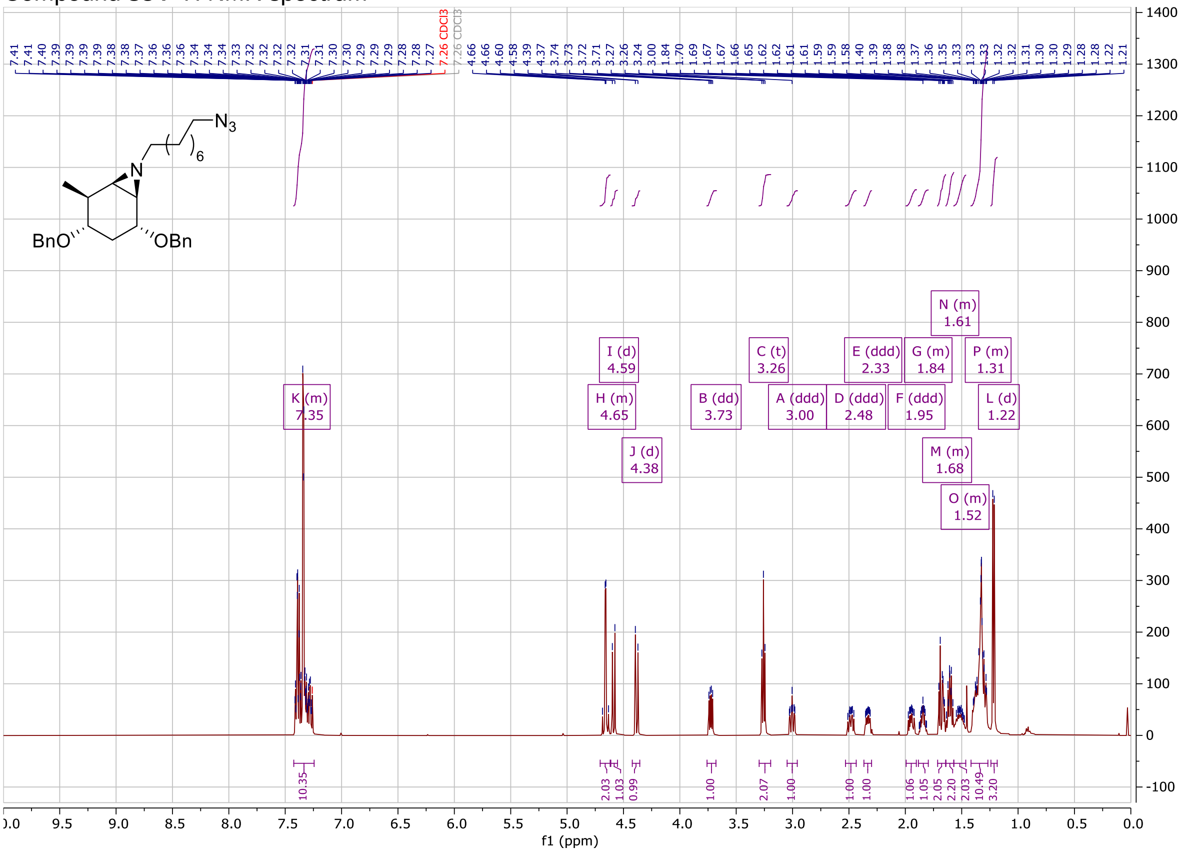

Compound **s84** ^{13}C NMR APT spectrum

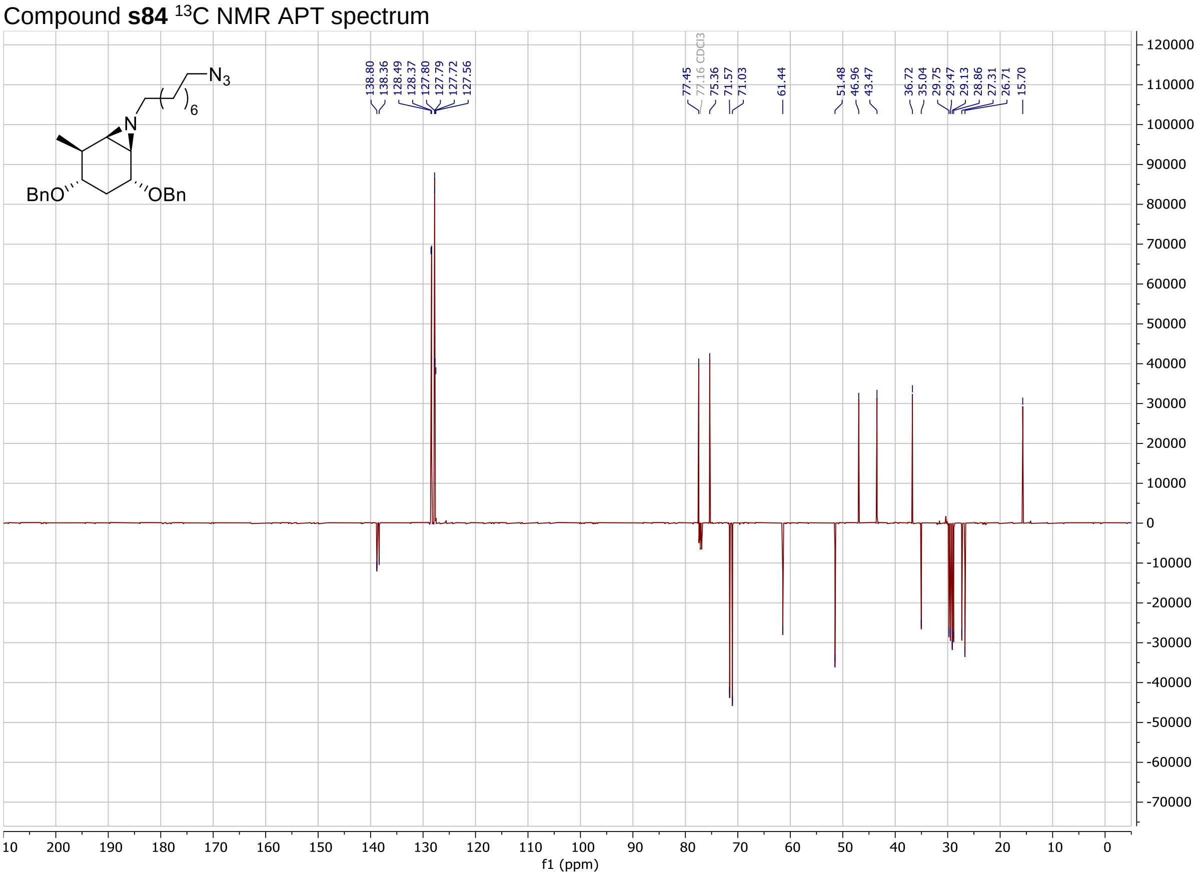

Compound **s84** ^{1}H-^{1}H COSY spectrum

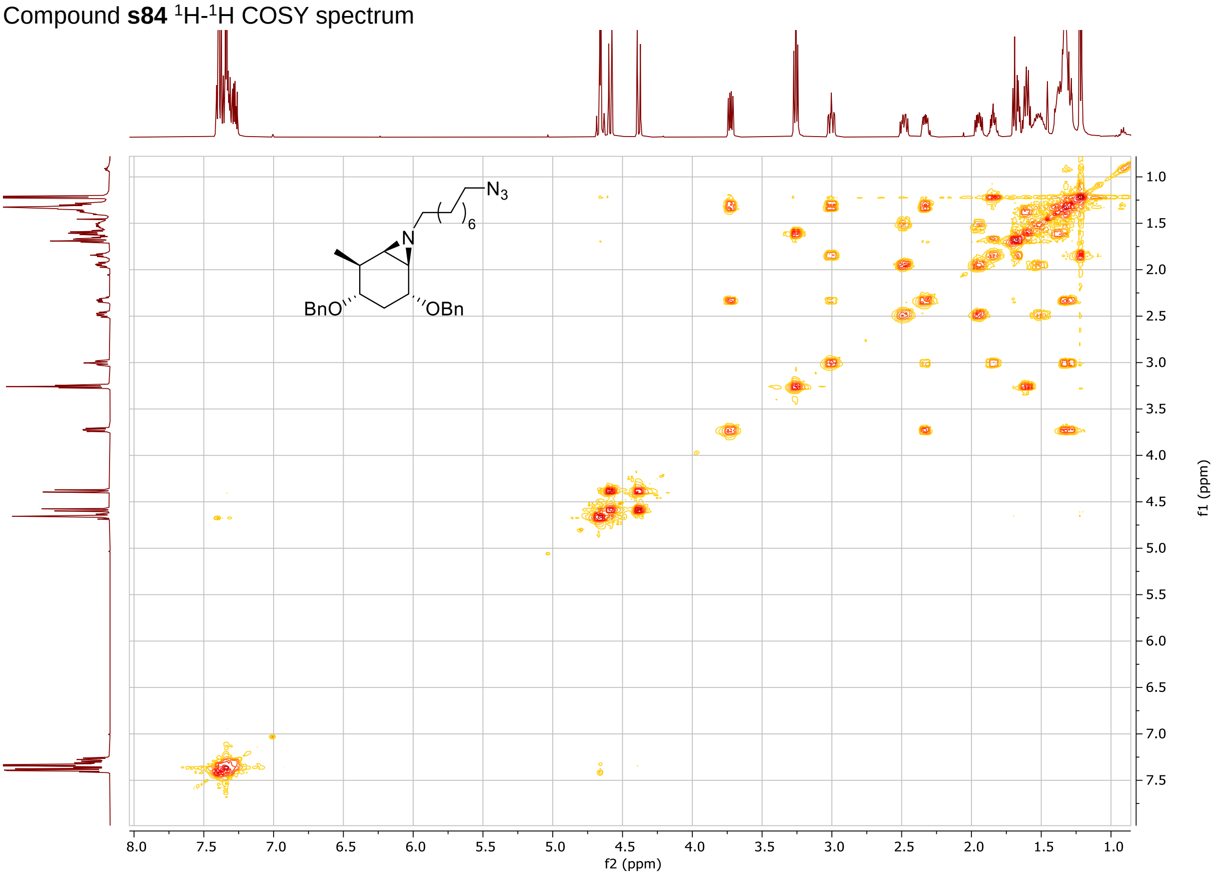

Compound **s84** ^{1}H-^{13}C HSQC spectrum

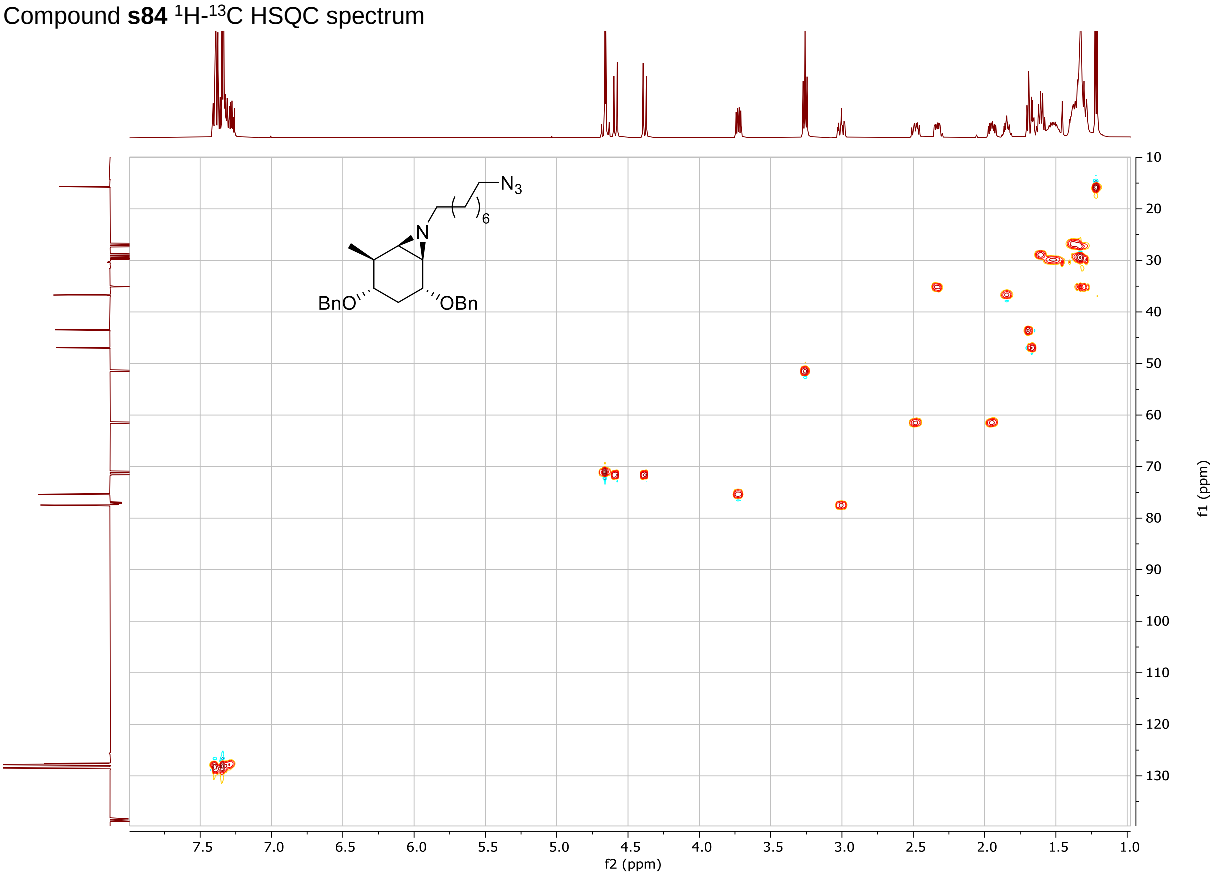

Compound **s85** ^{1}H NMR spectrum

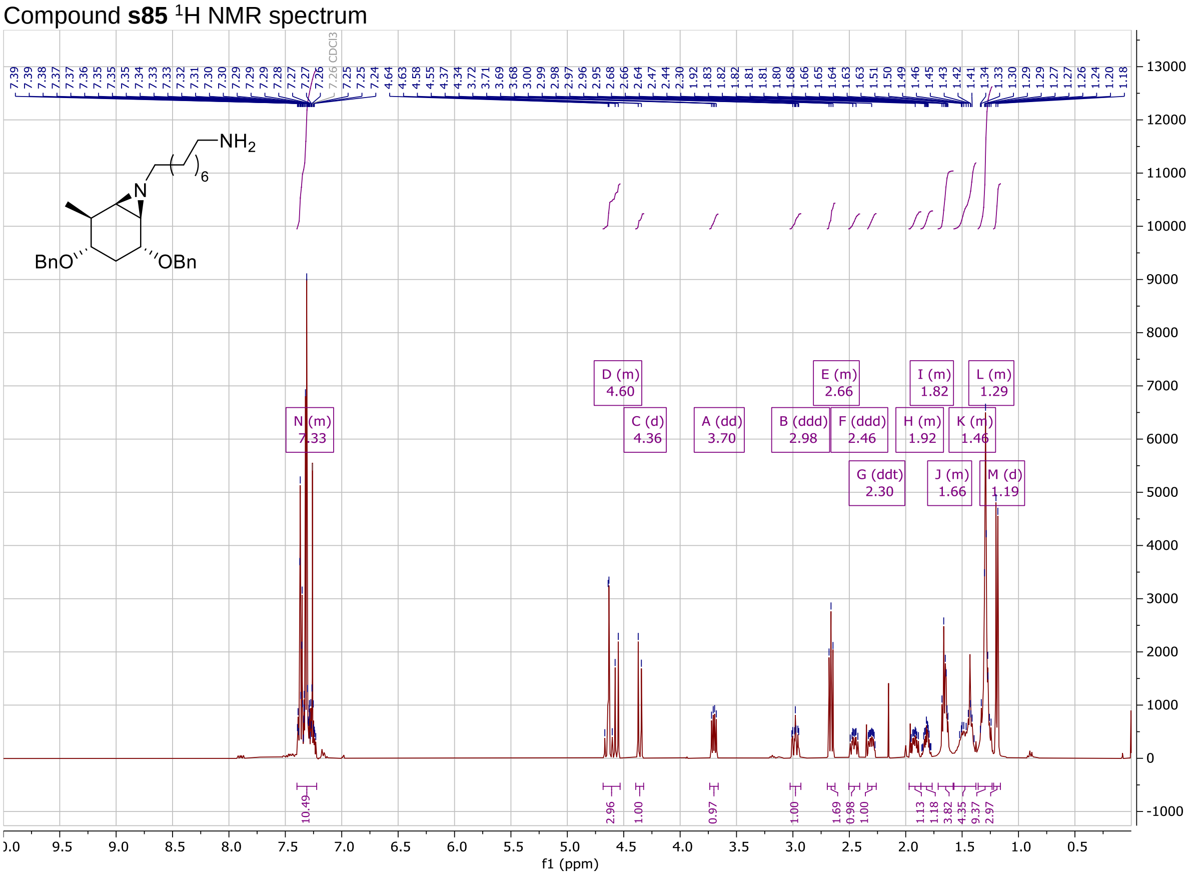

Compound **s85** ^{13}C NMR APT spectrum

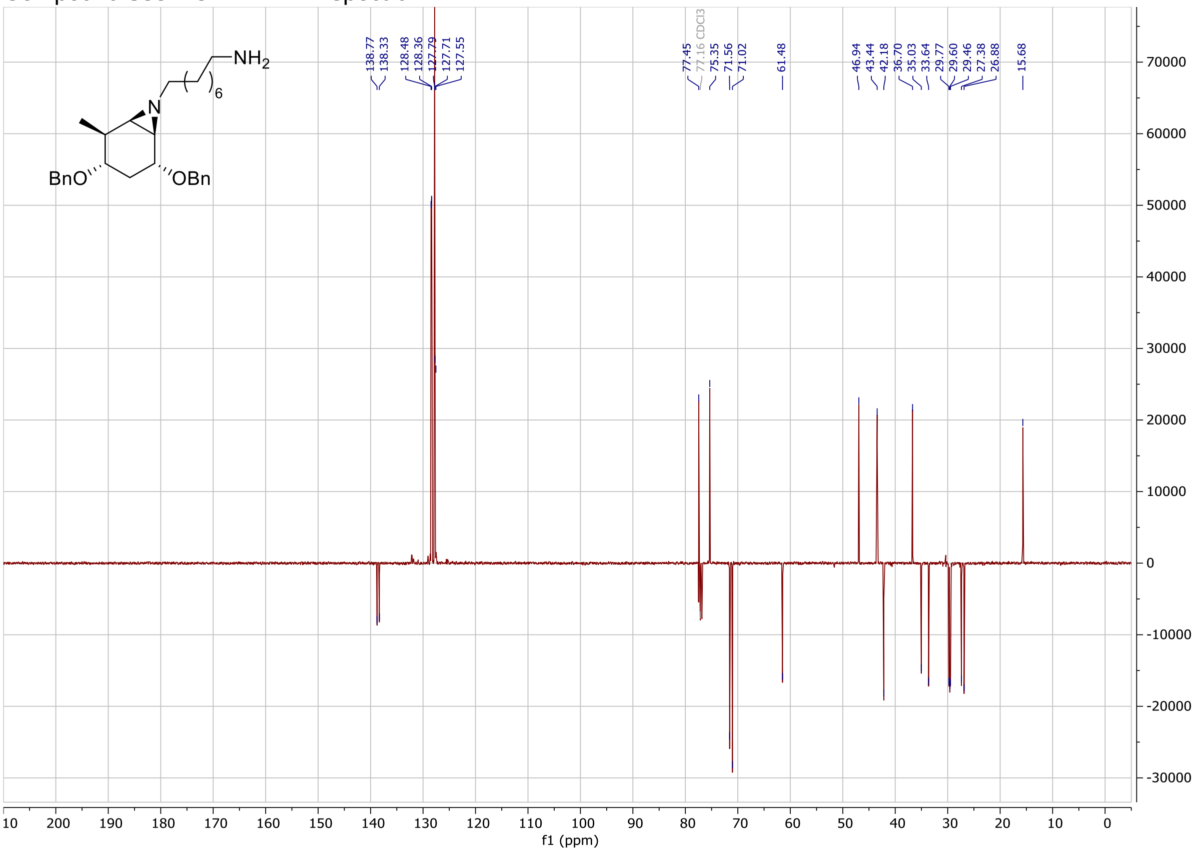

Compound **s85** ^{1}H-^{1}H COSY spectrum

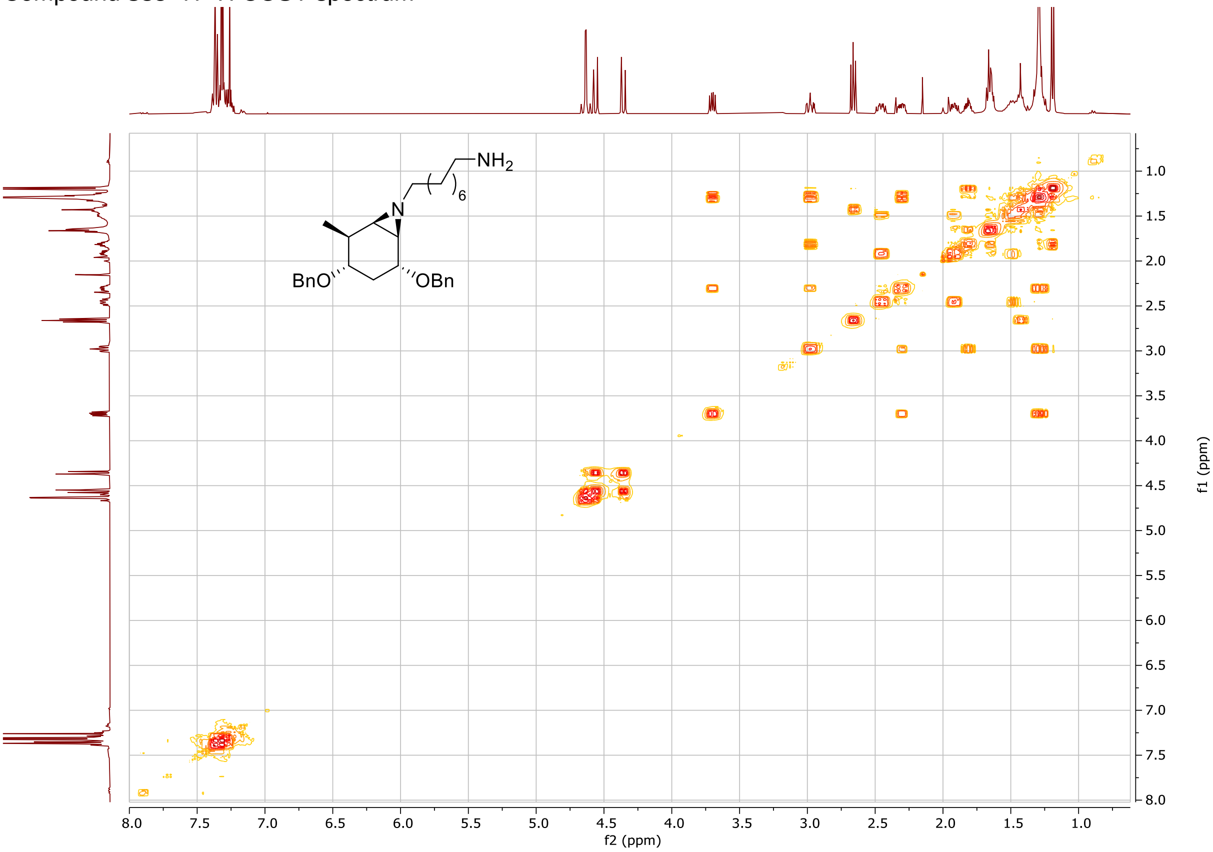

Compound **s85** ^1H-^{13}C HSQC spectrum

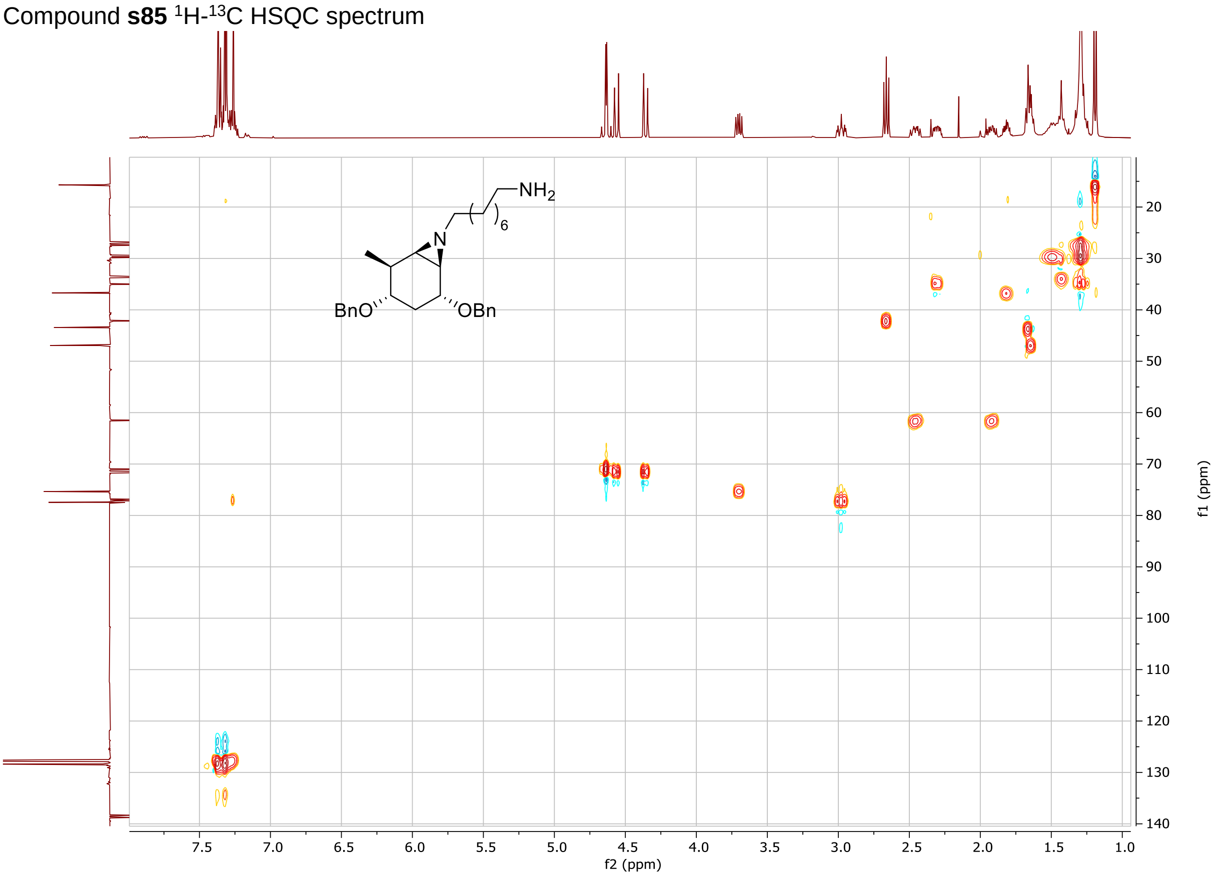

Compound **s86** ^1H NMR spectrum

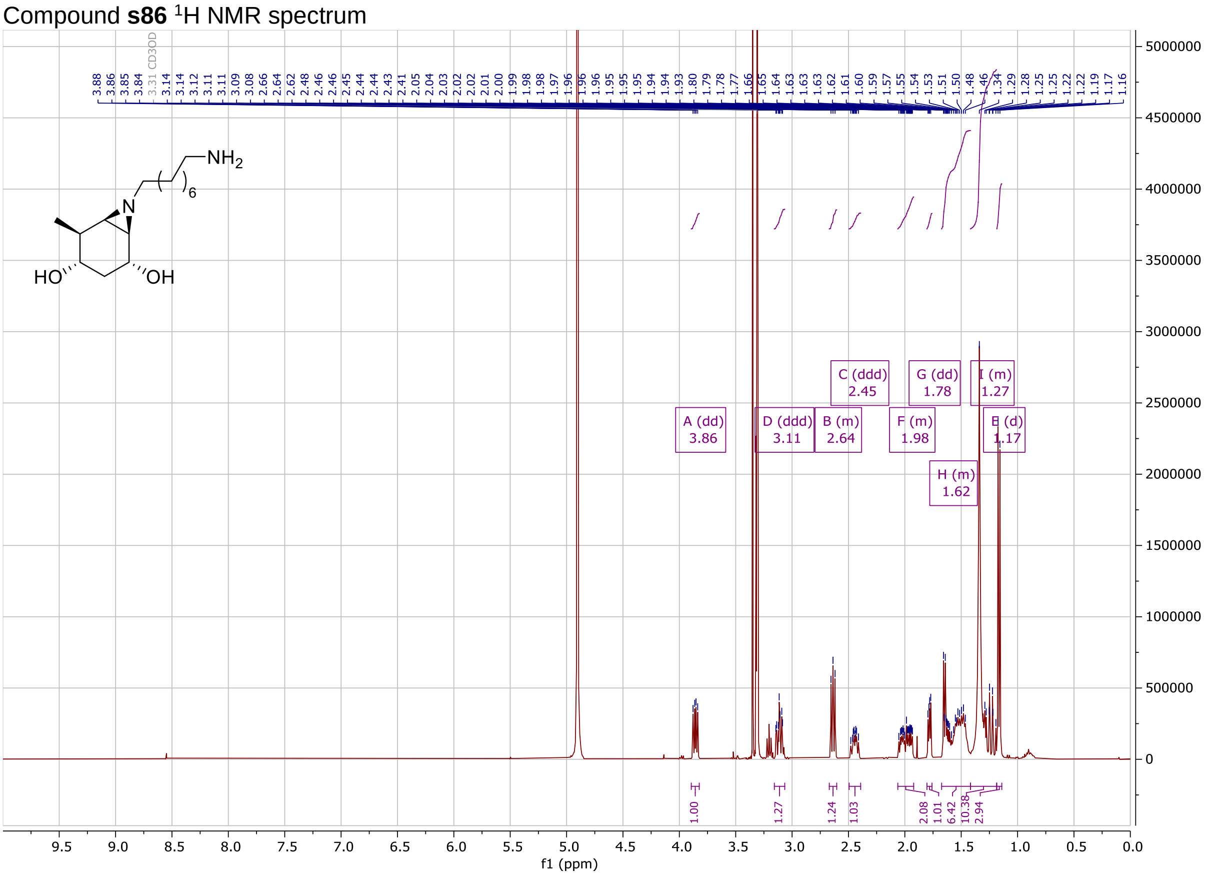

Compound **s86** ^{13}C NMR APT spectrum

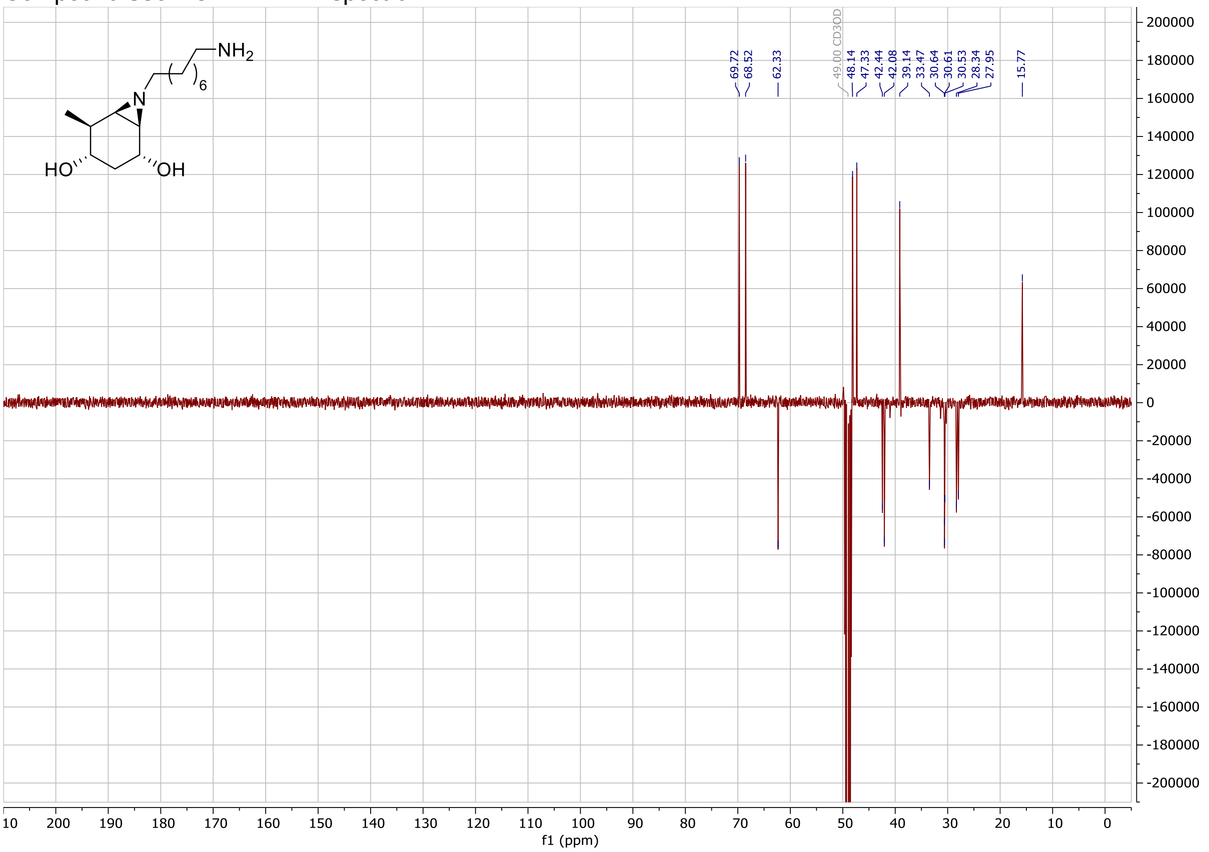

Compound **s86** ^{1}H-^{1}H COSY spectrum

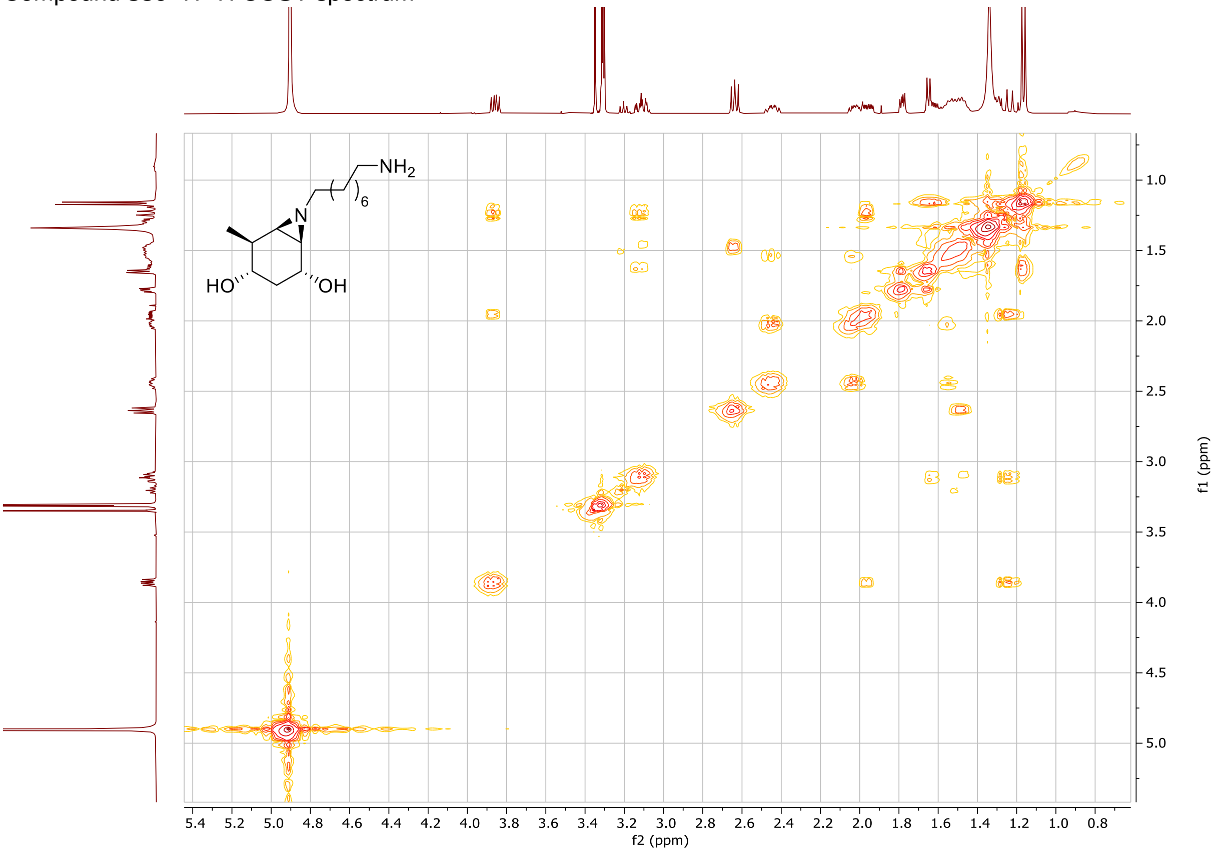

Compound **s86** ^{1}H-^{13}C HSQC spectrum

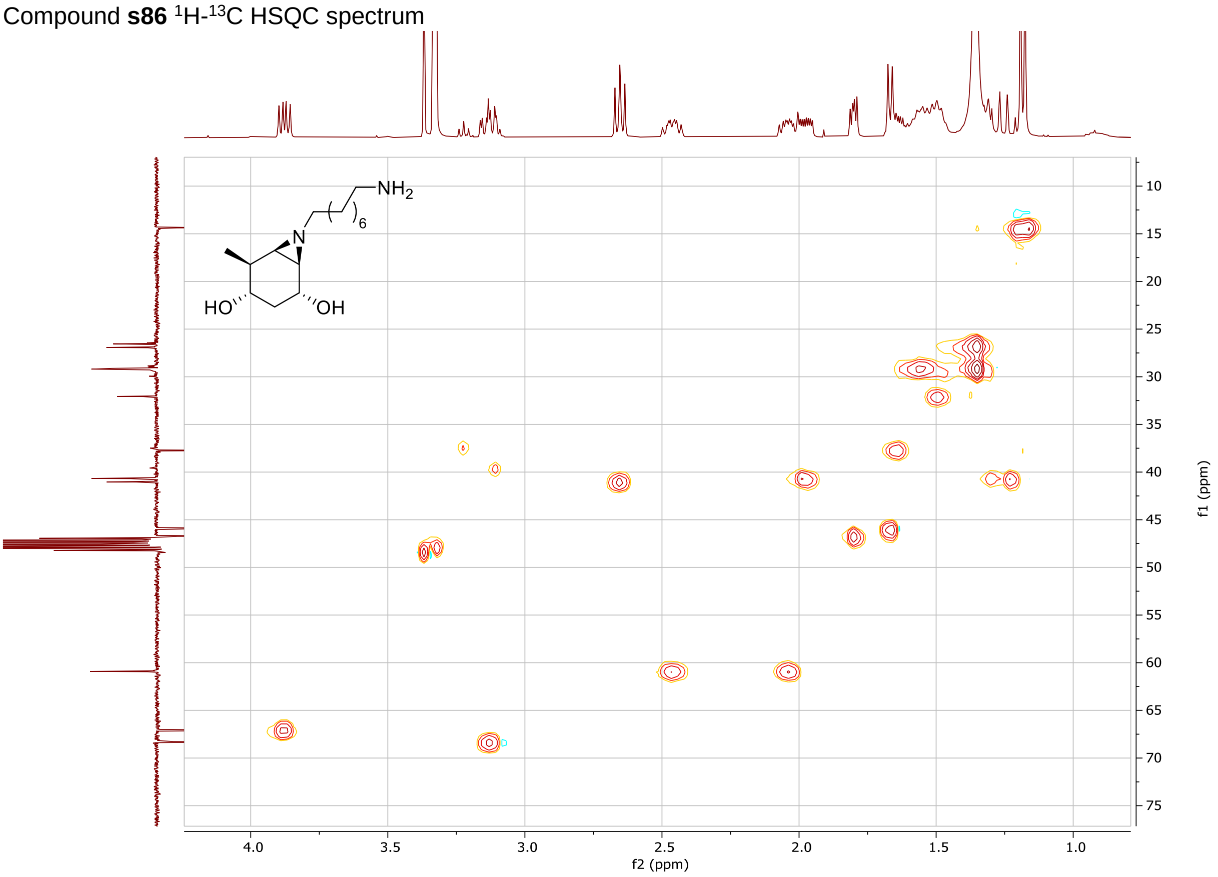

Compound **53** ^{1}H NMR spectrum

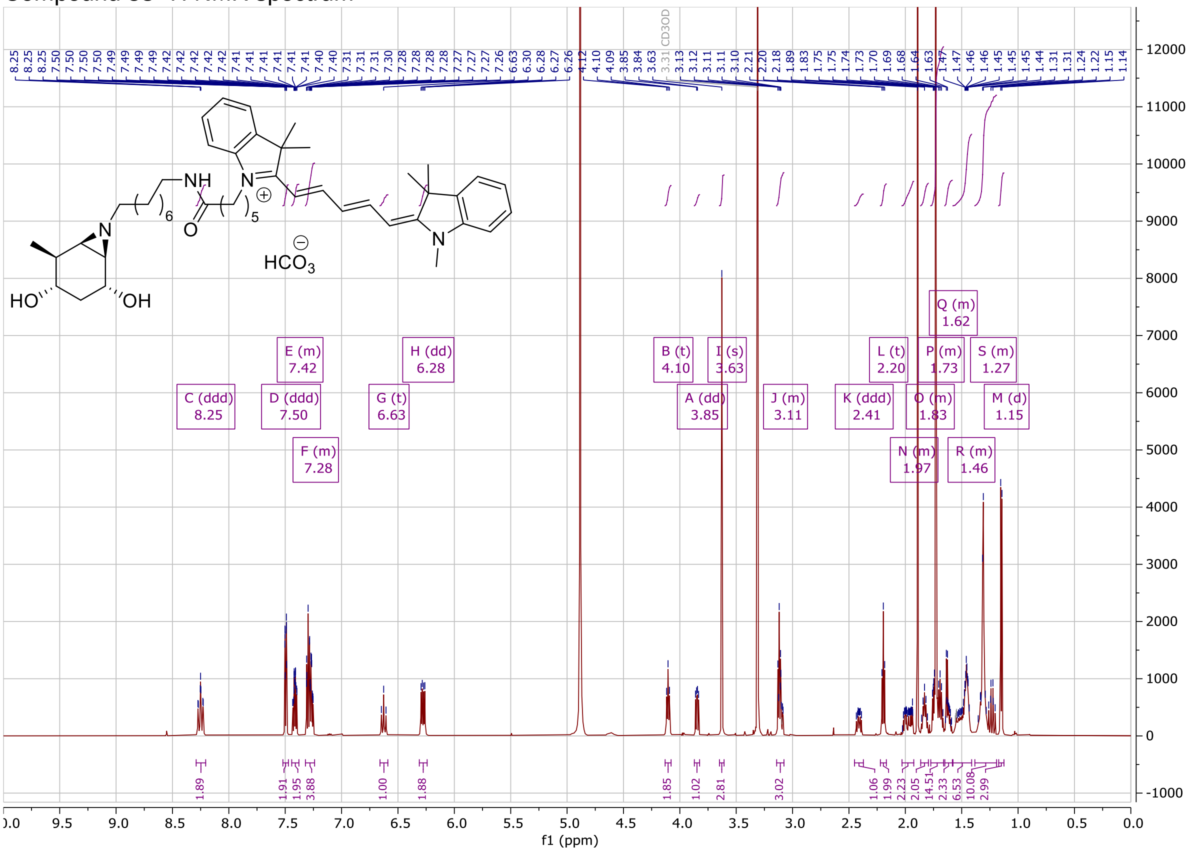

Compound **53** ^{13}C NMR APT spectrum

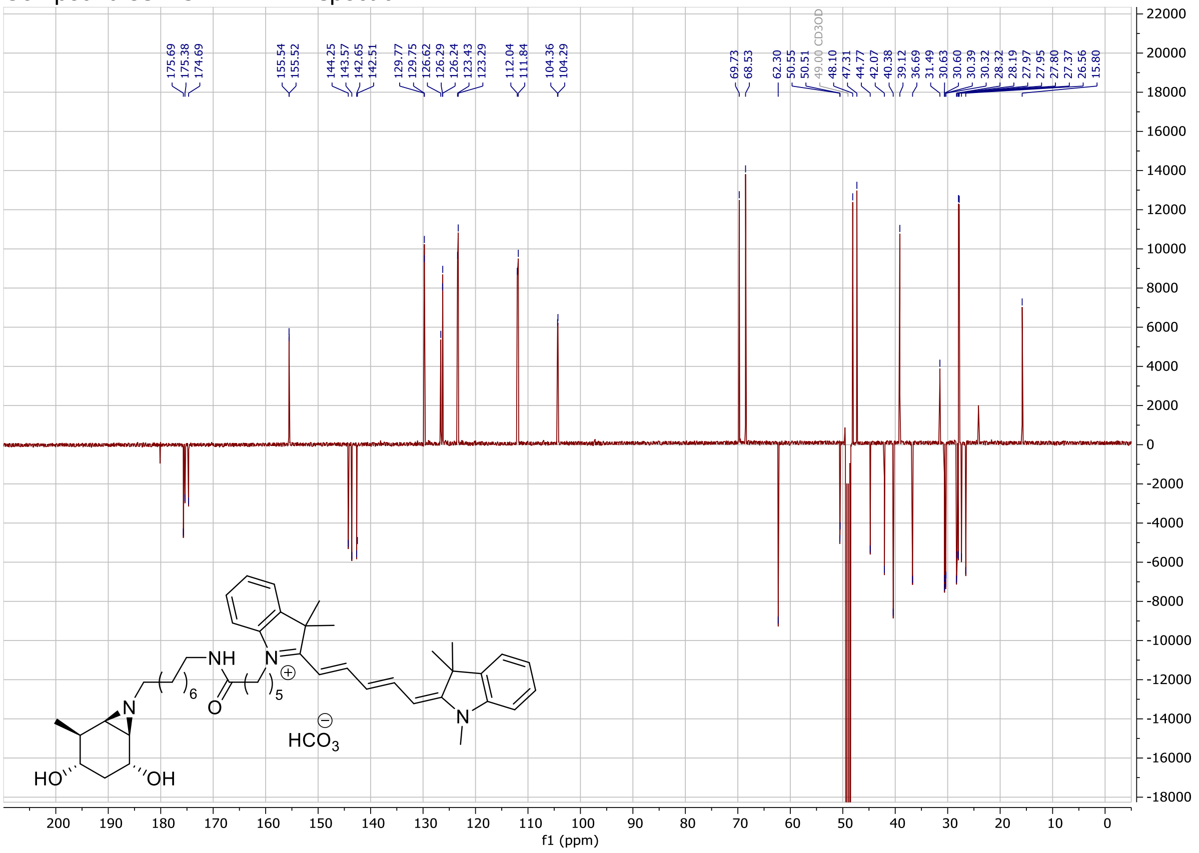

Compound **53** ^{1}H-^{1}H COSY spectrum

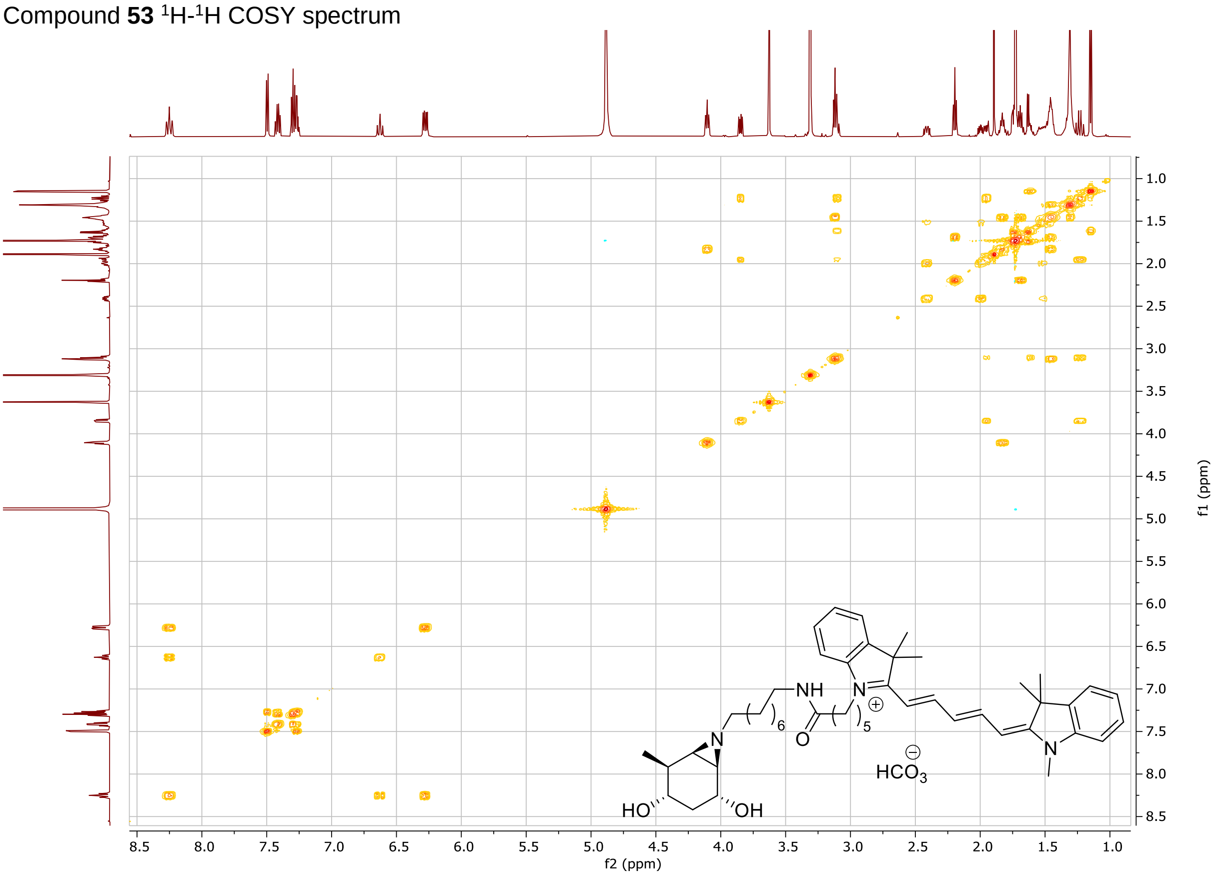

Compound **53** ^{1}H-^{13}C HSQC spectrum

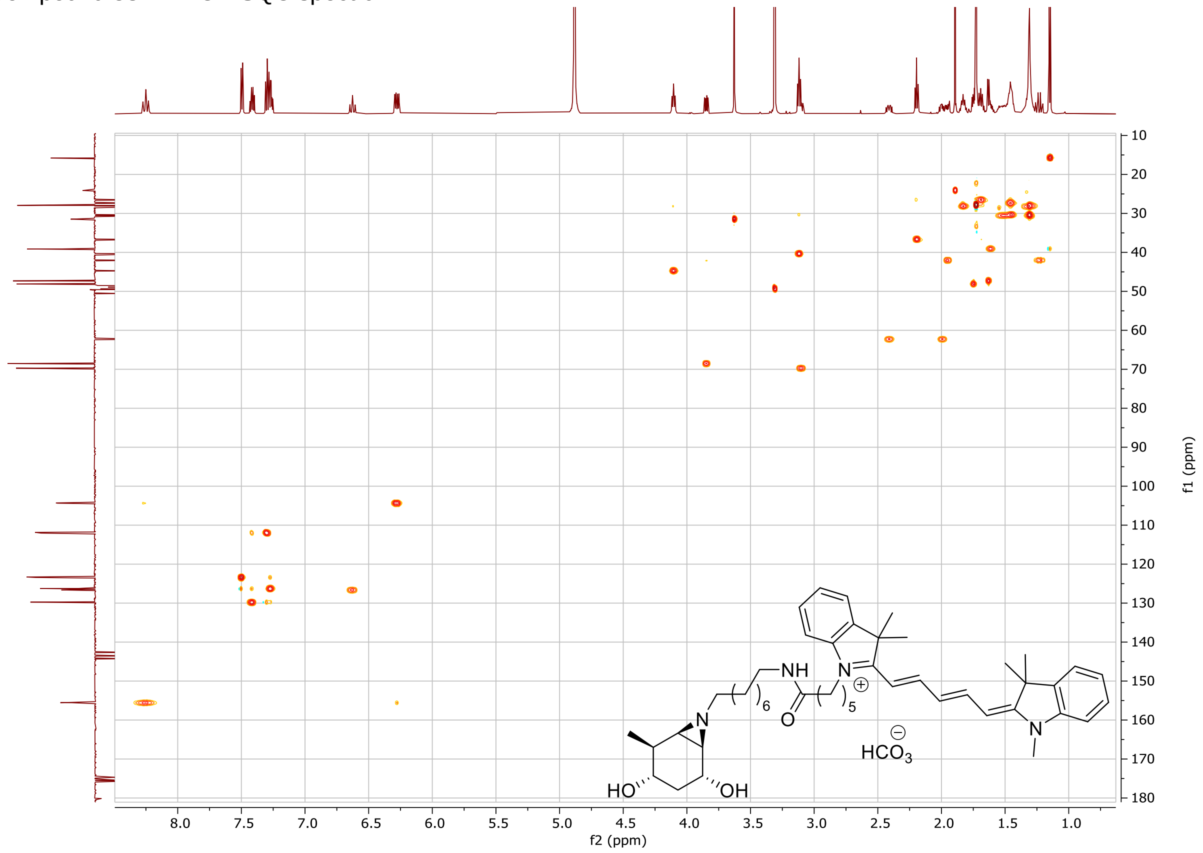

Compound **60** ^{1}H NMR spectrum

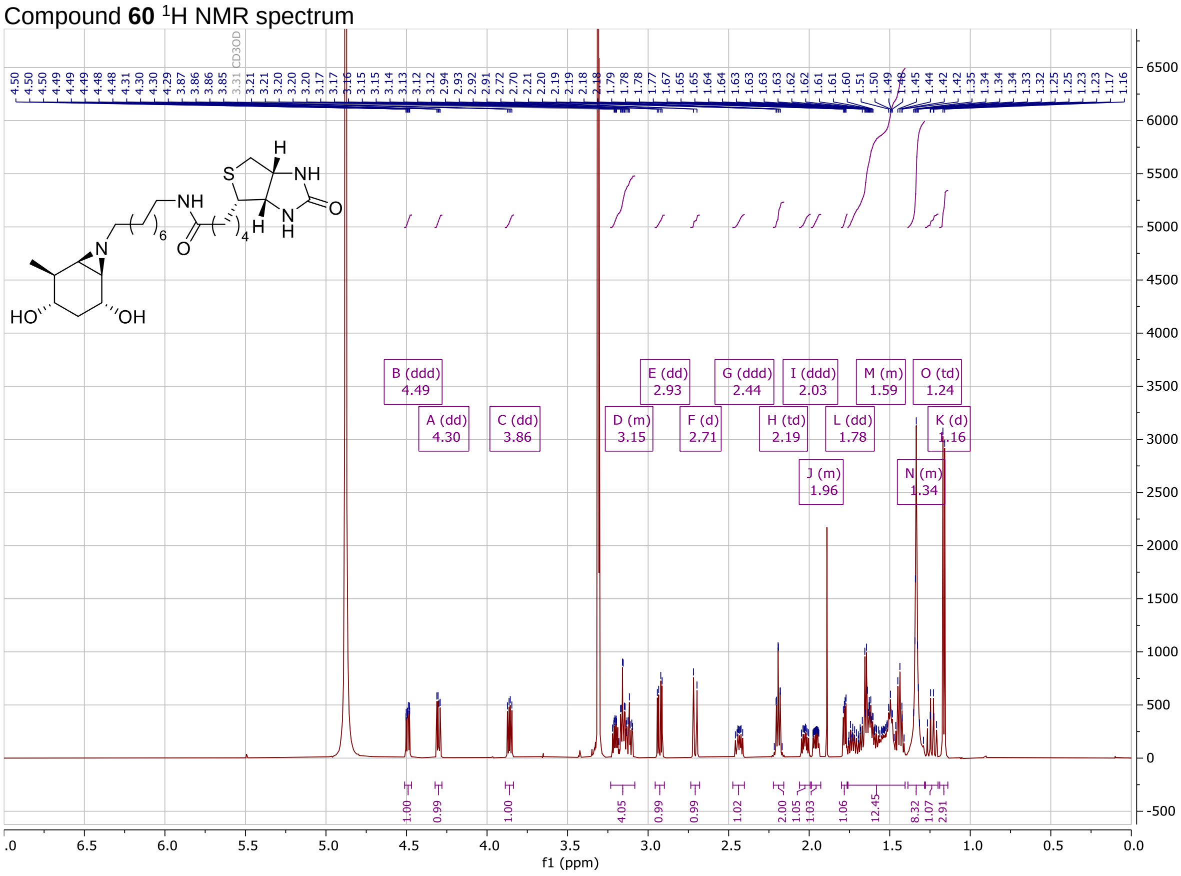

Compound **60** ^{13}C NMR APT spectrum

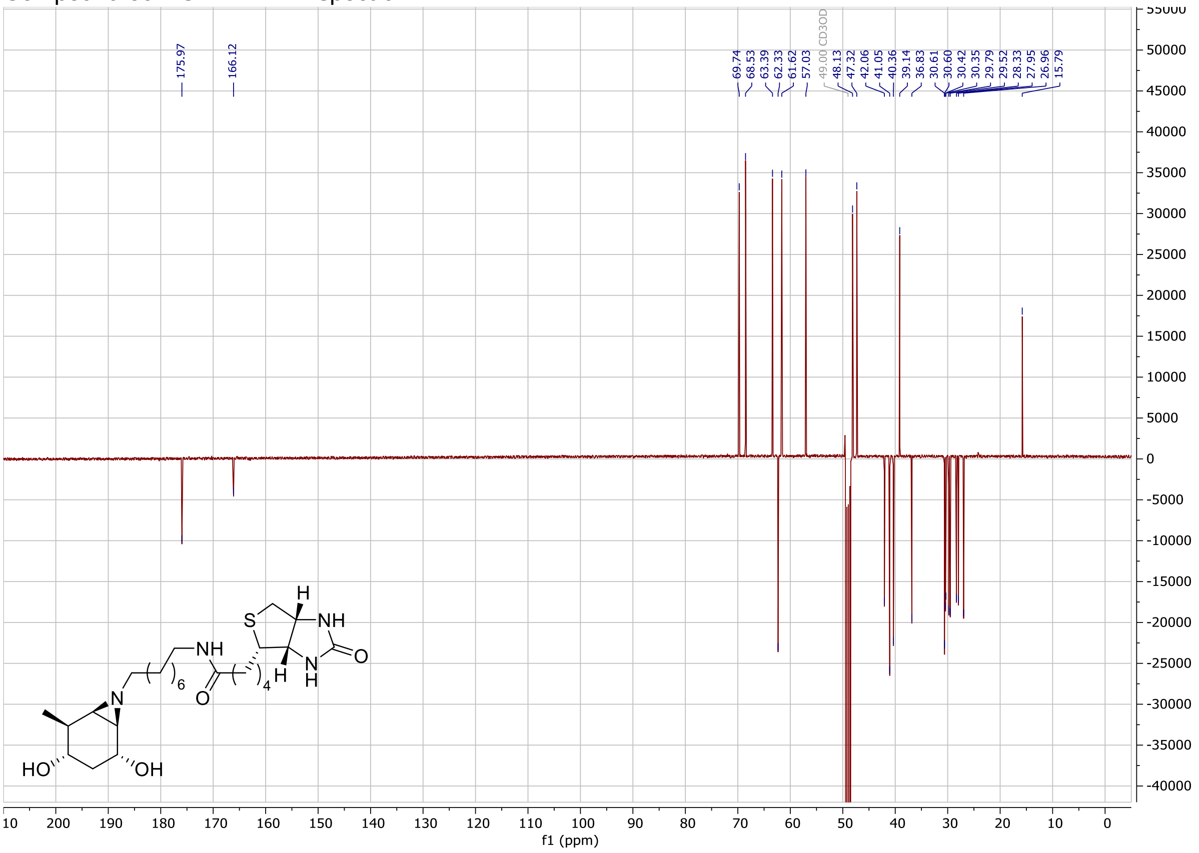

Compound **60** ^{1}H-^{1}H COSY spectrum

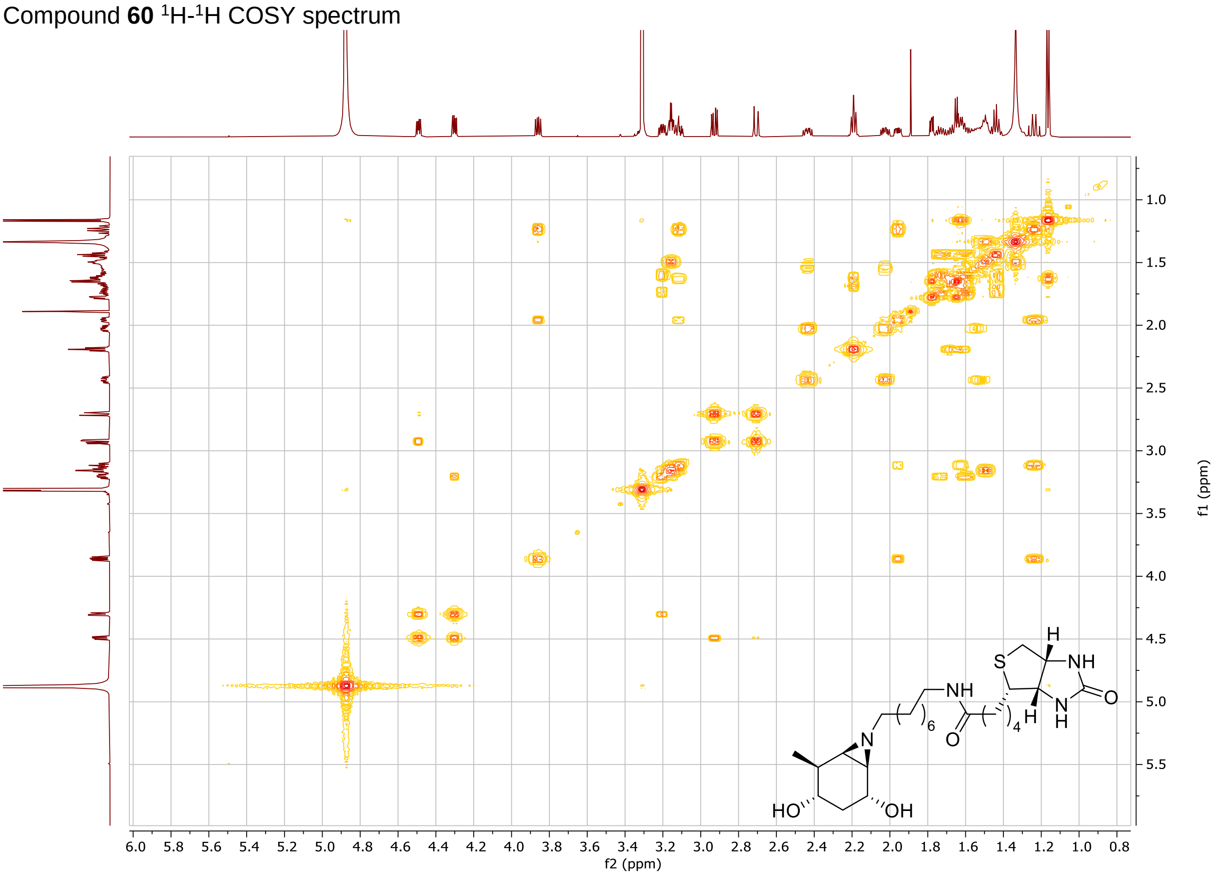

Compound **60** ^{1}H-^{13}C HSQC spectrum

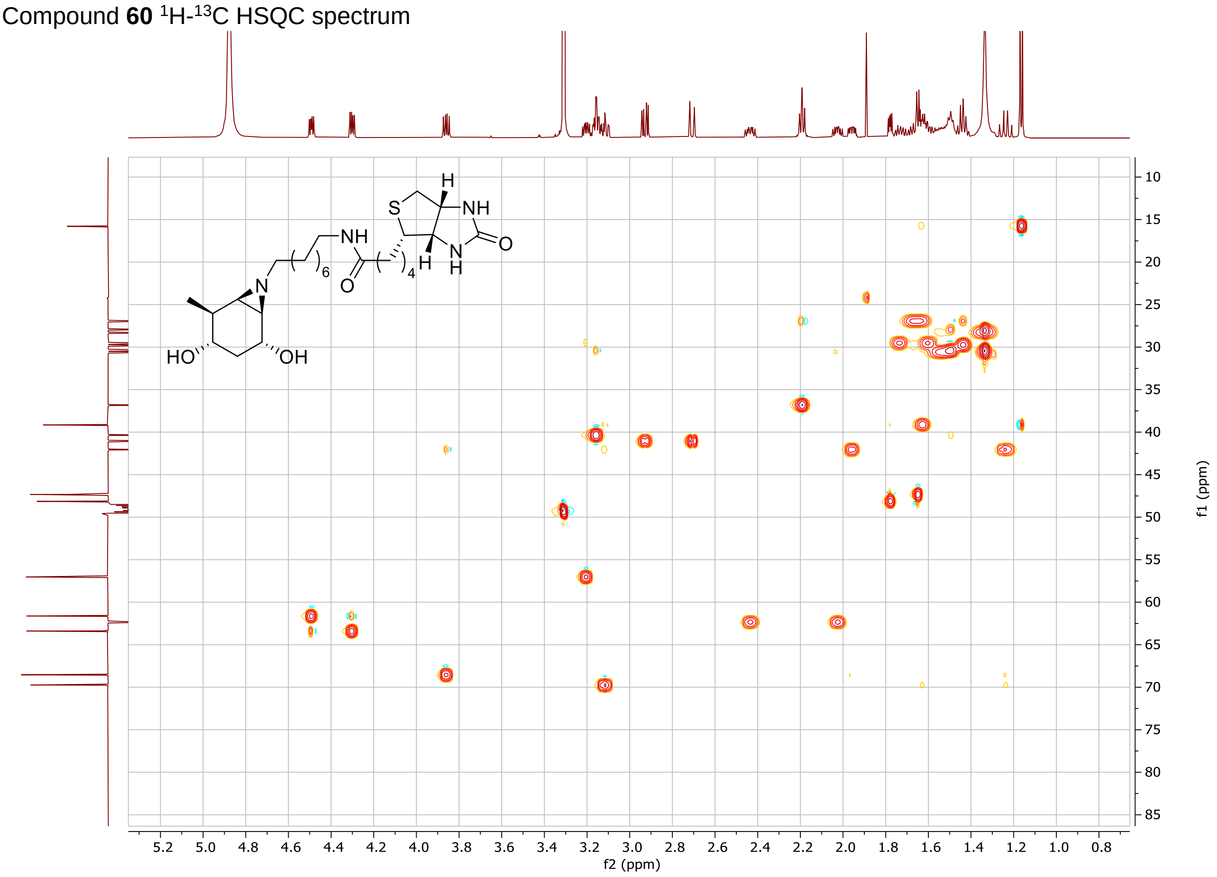

Compound **s87** ^{1}H NMR spectrum

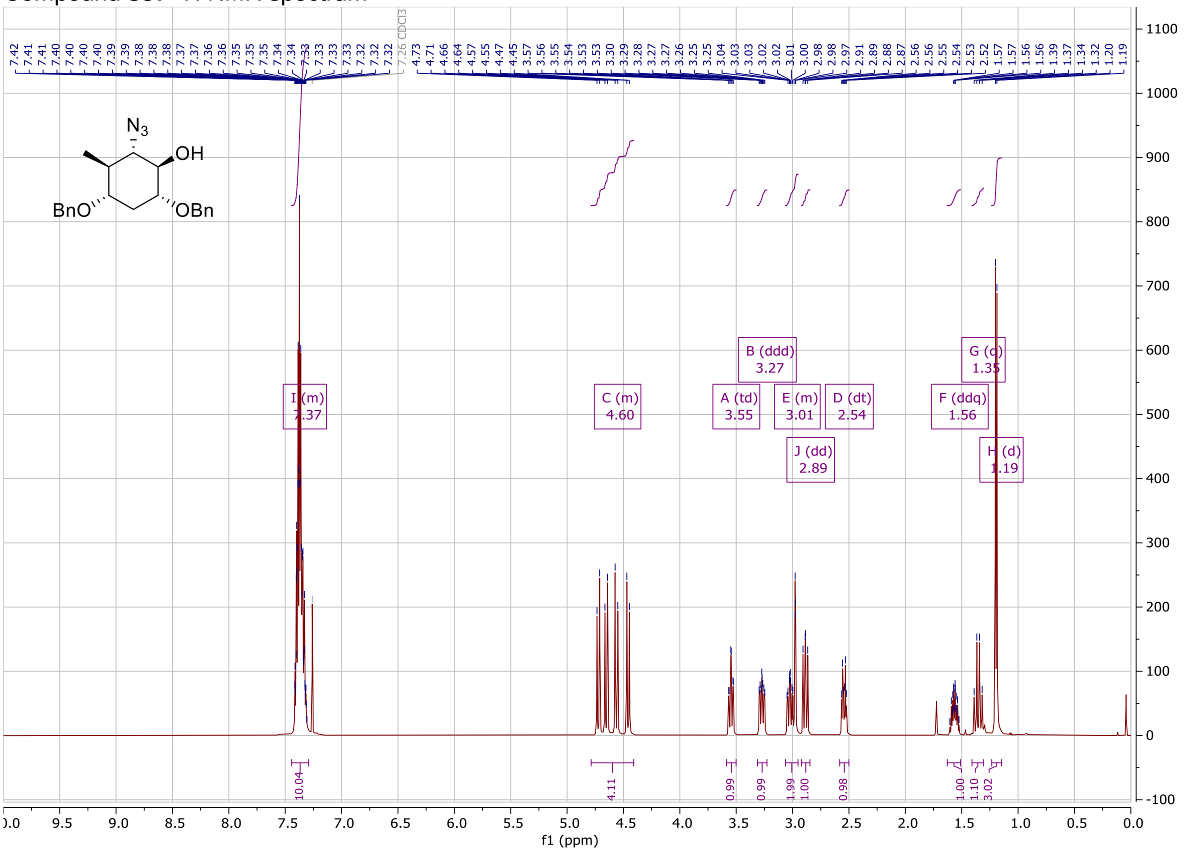

Compound **s87** ^{13}C NMR APT spectrum

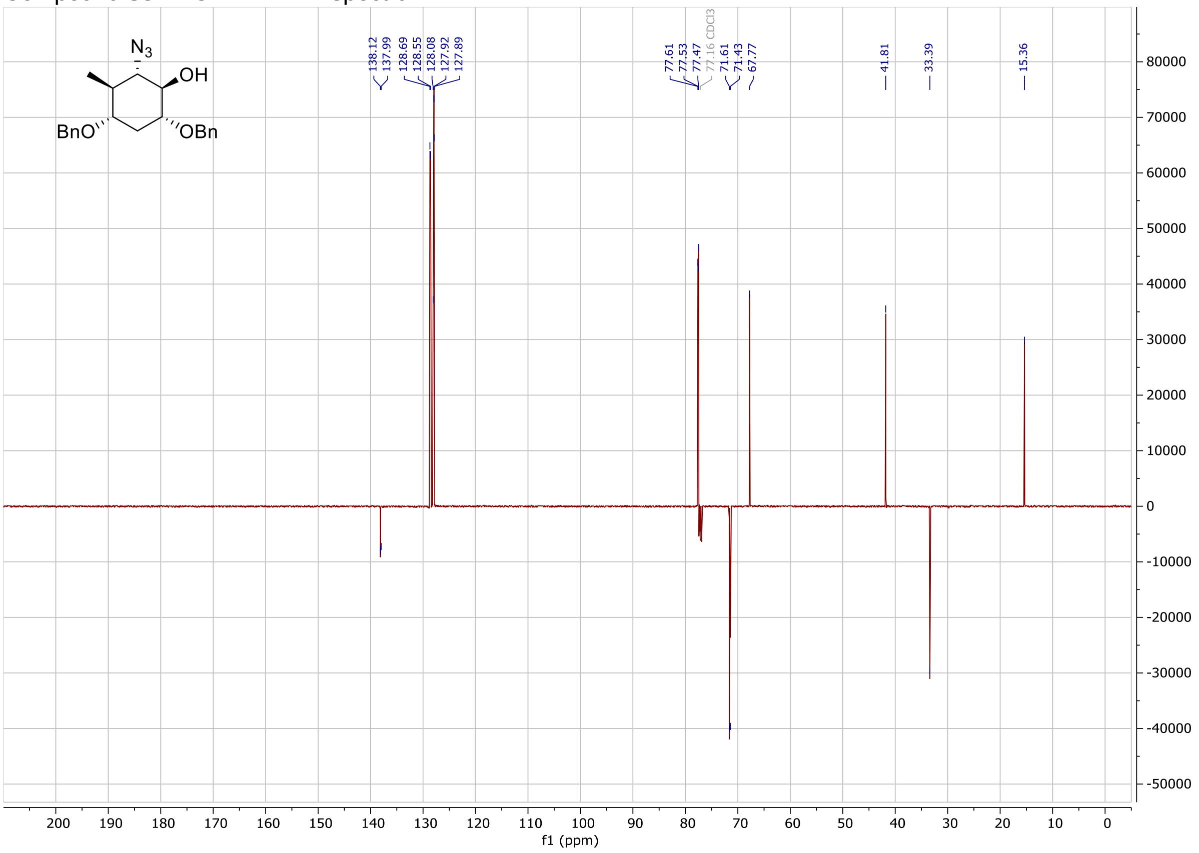

Compound **s87** ^{1}H-^{1}H COSY spectrum

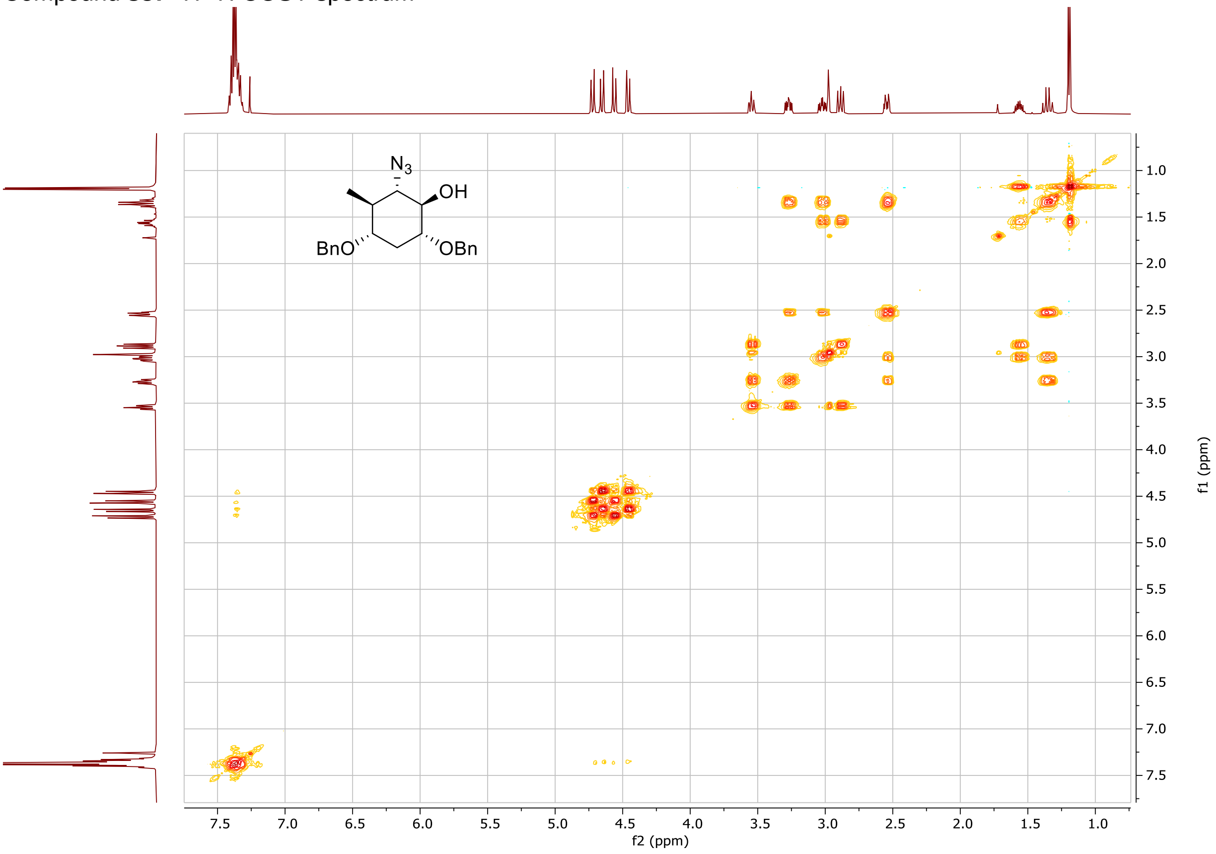

Compound **s87** ^{1}H-^{13}C HSQC spectrum

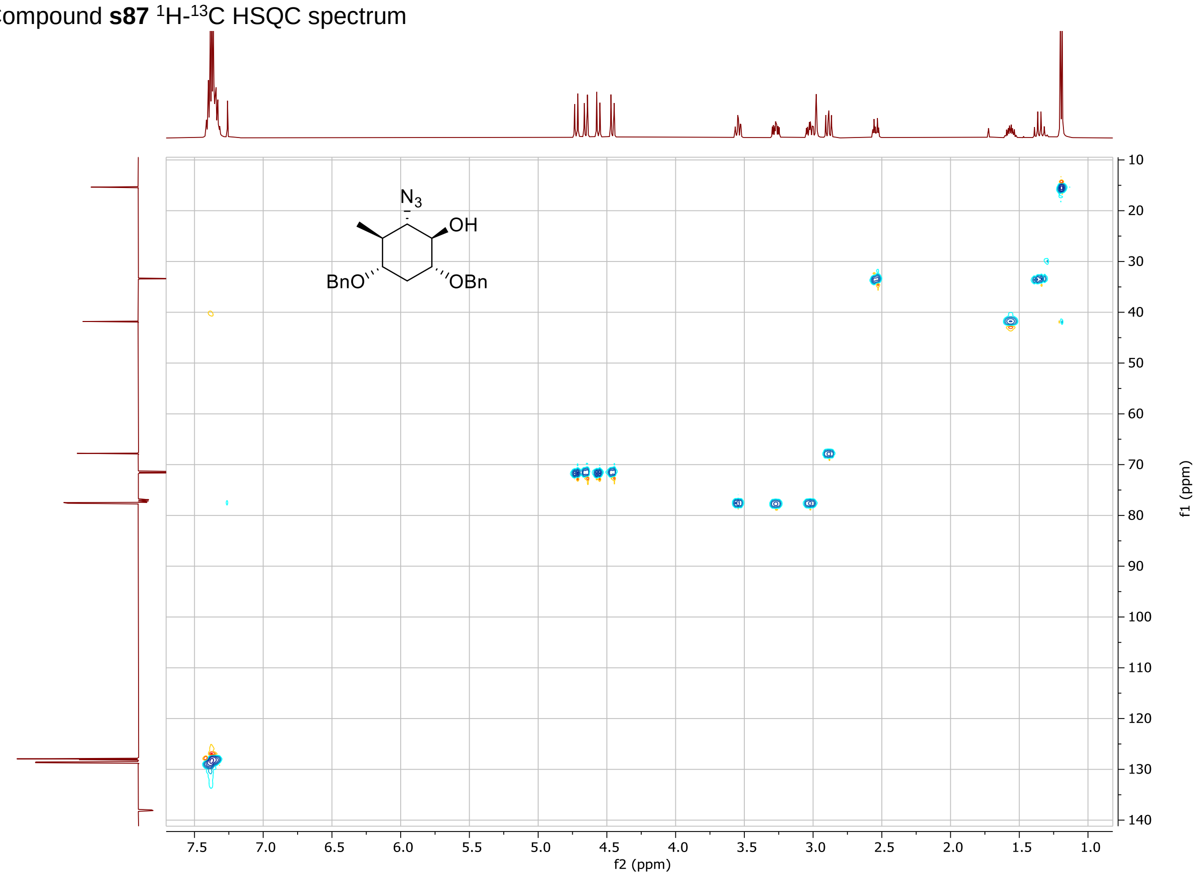

Compound **s87** ^{1}H-^{1}H NOESY spectrum

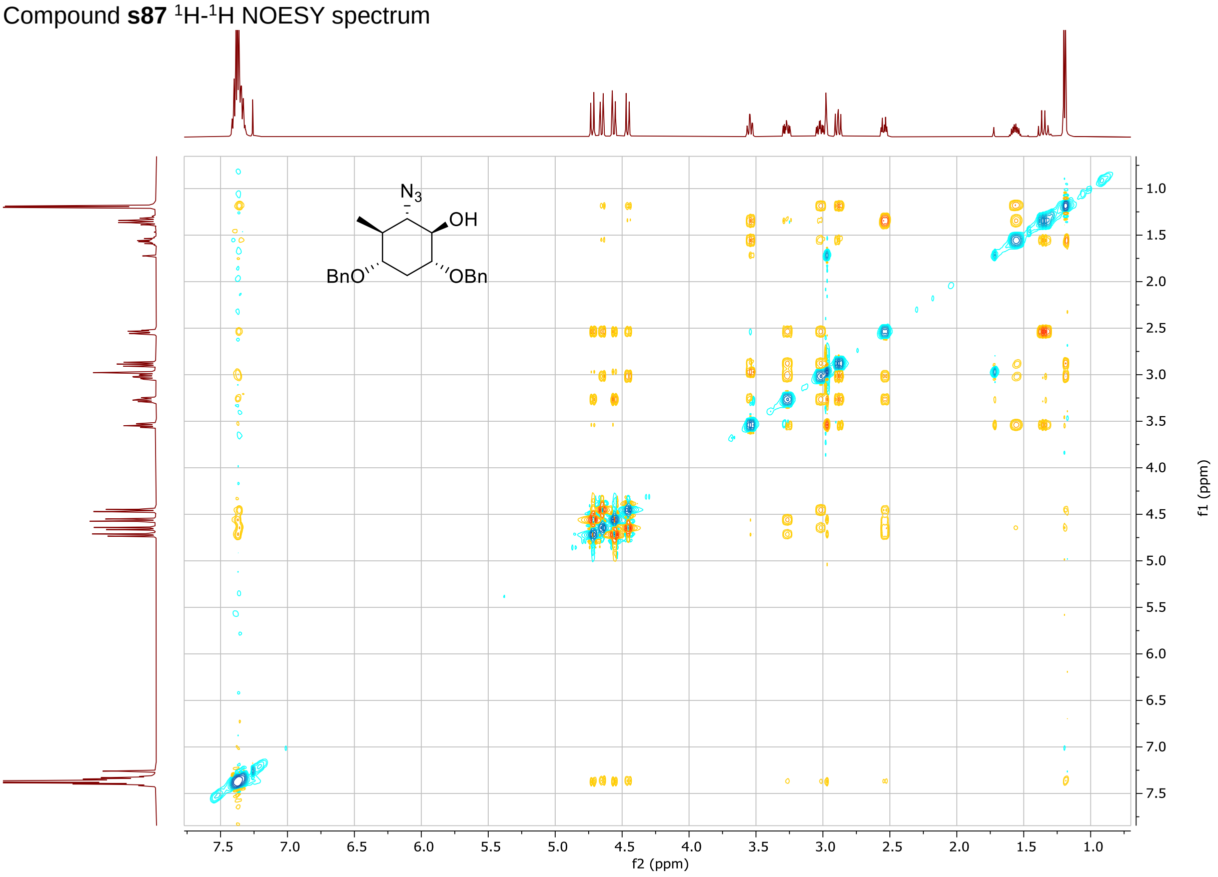

Compound **s87** ^{1}H-^{13}C HMBC spectrum

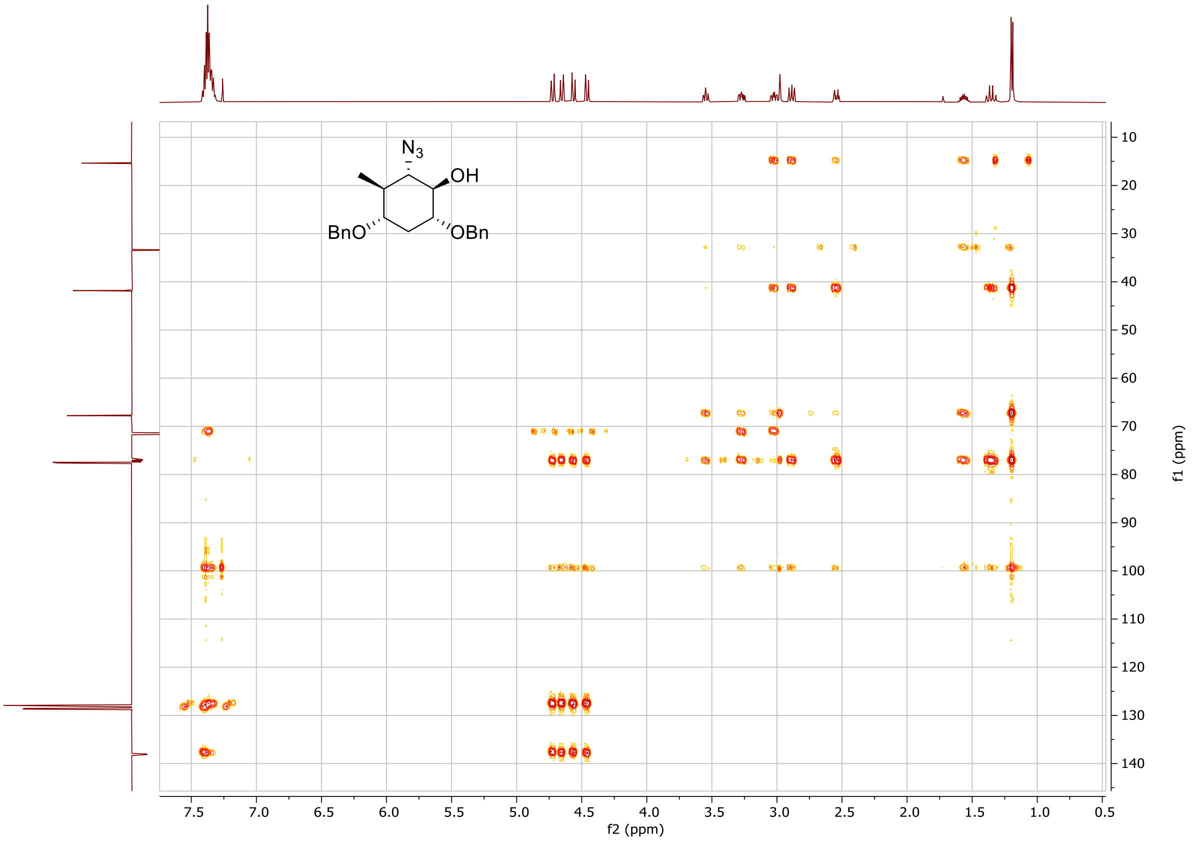

Compound **s88** ^{1}H NMR spectrum

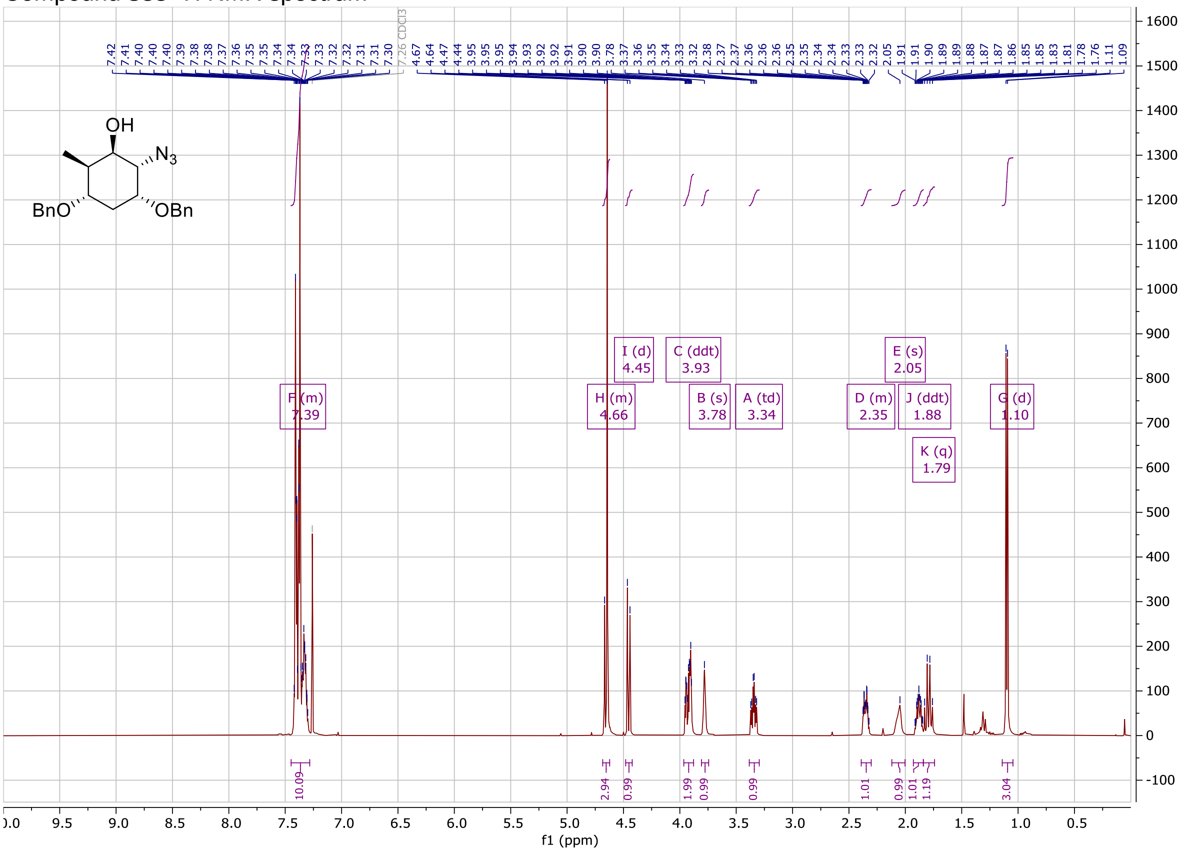

Compound **s88** ^{13}C NMR APT spectrum

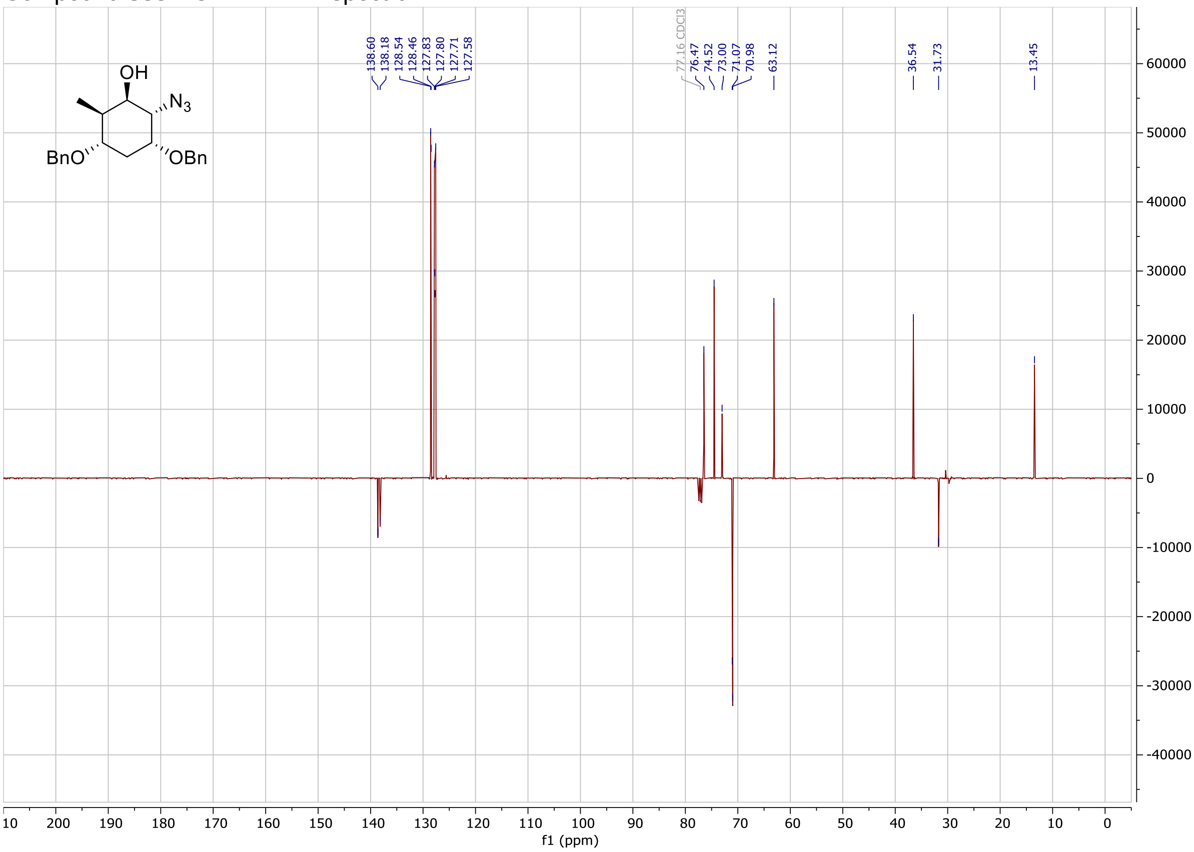

Compound **s88** ^{1}H-^{1}H COSY spectrum

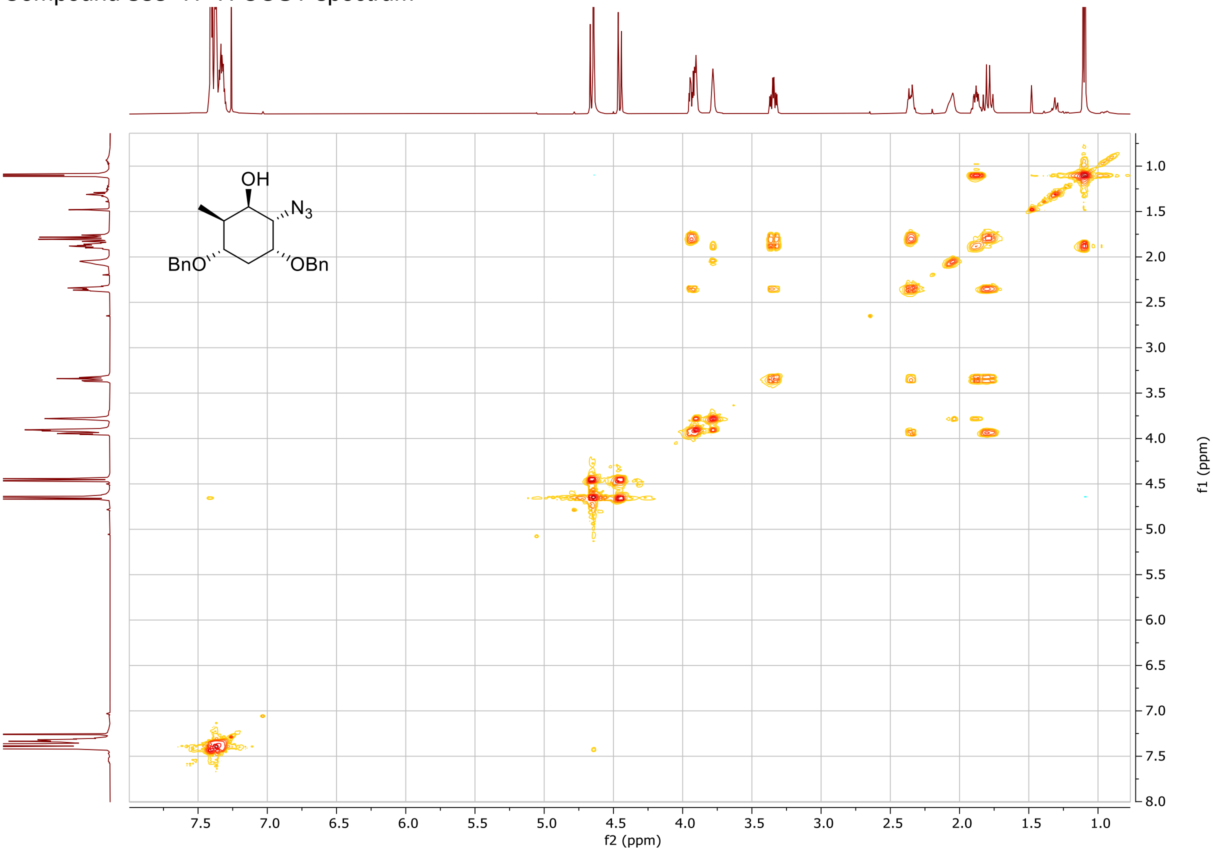

Compound **s88** ^{1}H-^{13}C HSQC spectrum

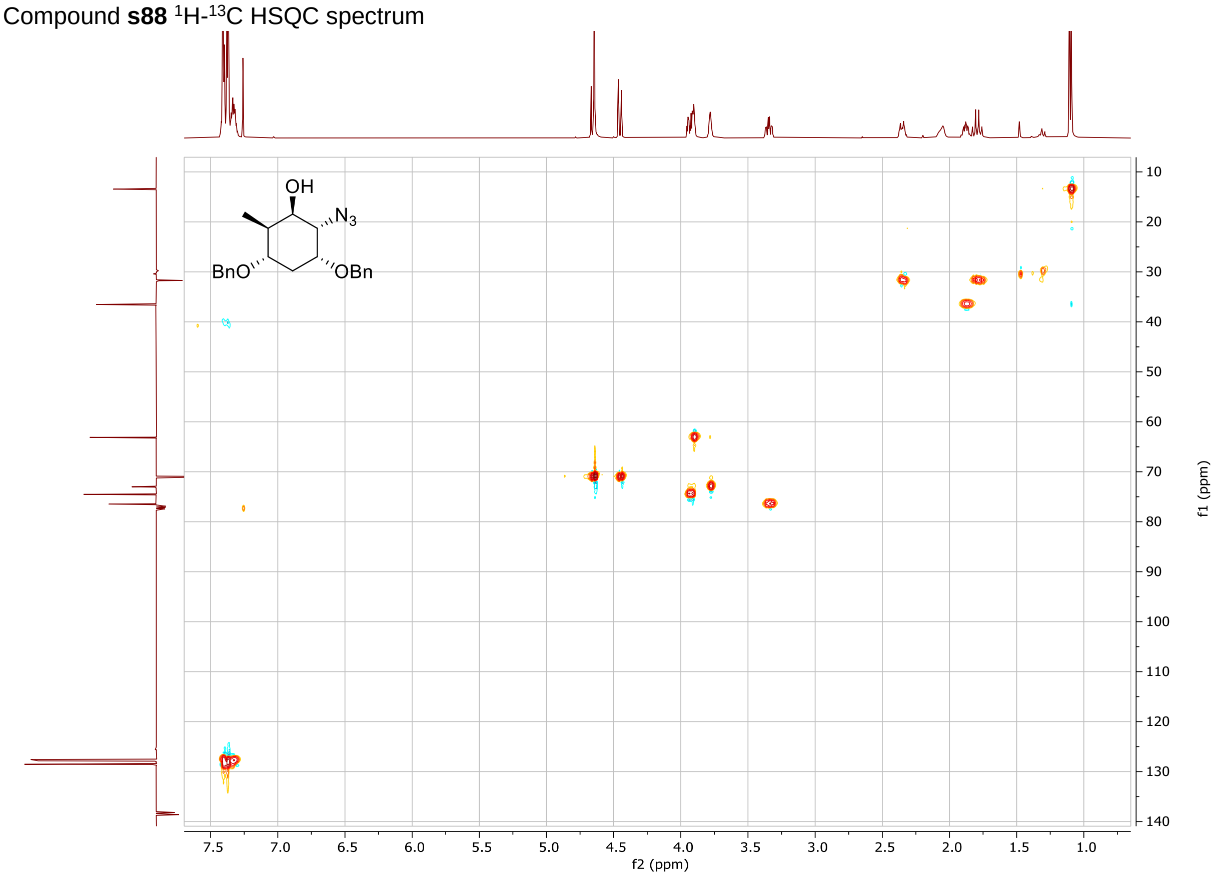

Compound **s88** ^{1}H-^{1}H NOESY spectrum

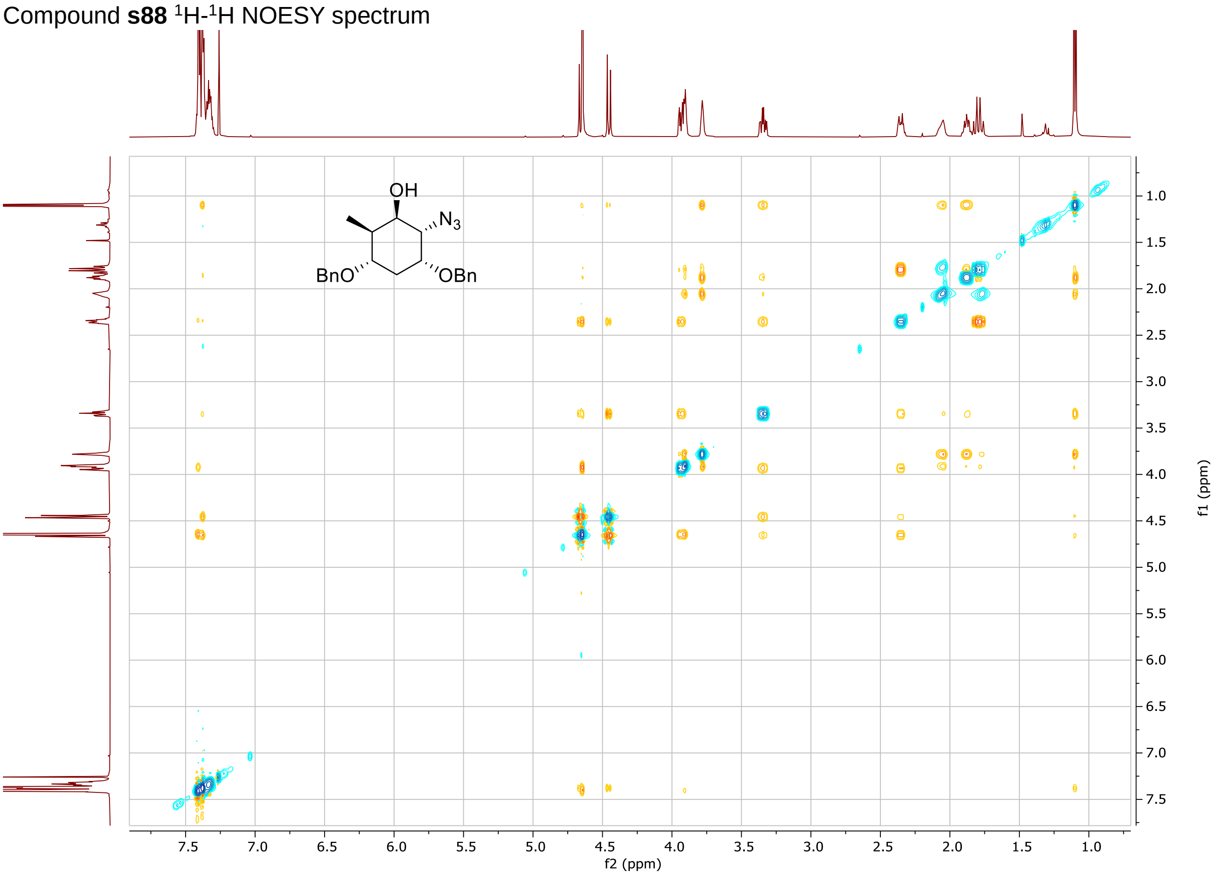

Compound **s88** ^{1}H-^{13}C HMBC spectrum

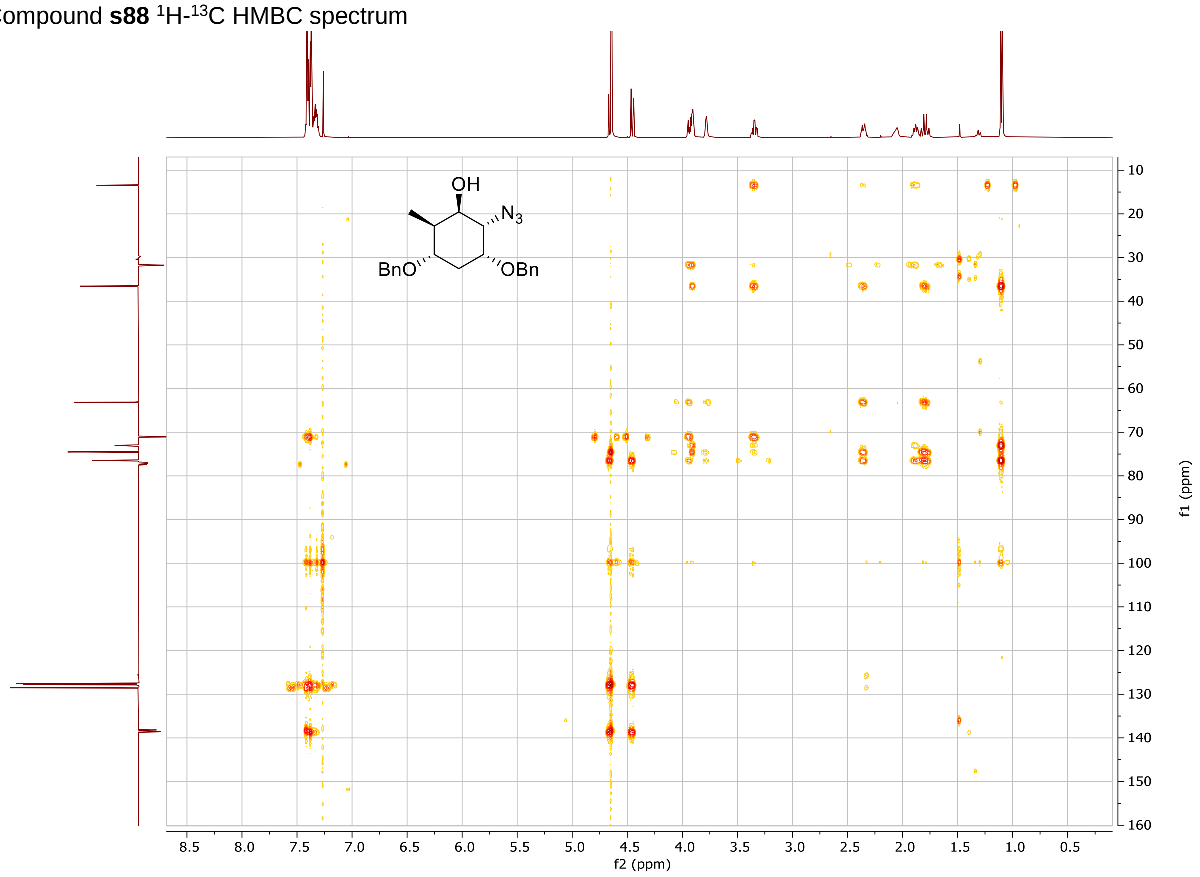

Compound **s90** ^{1}H NMR spectrum

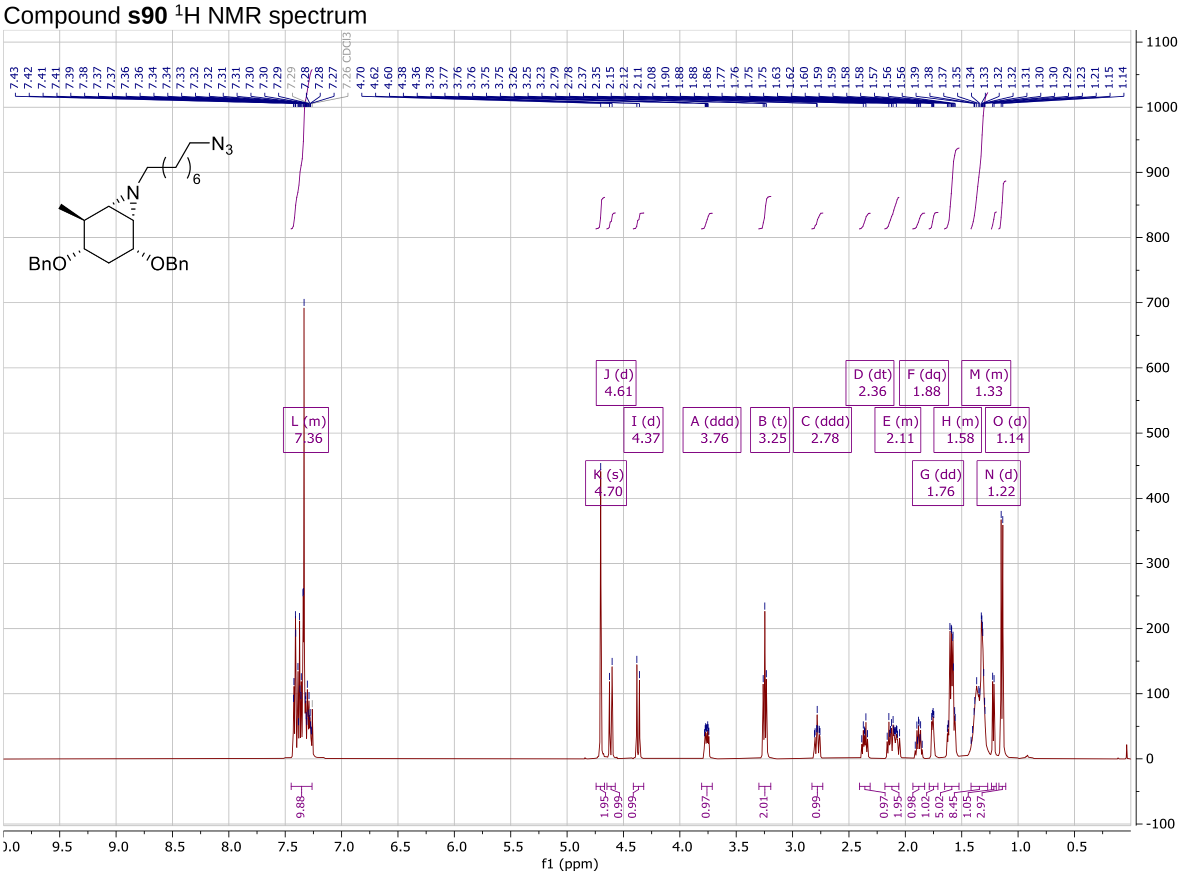

Compound **s90** ^{13}C NMR APT spectrum

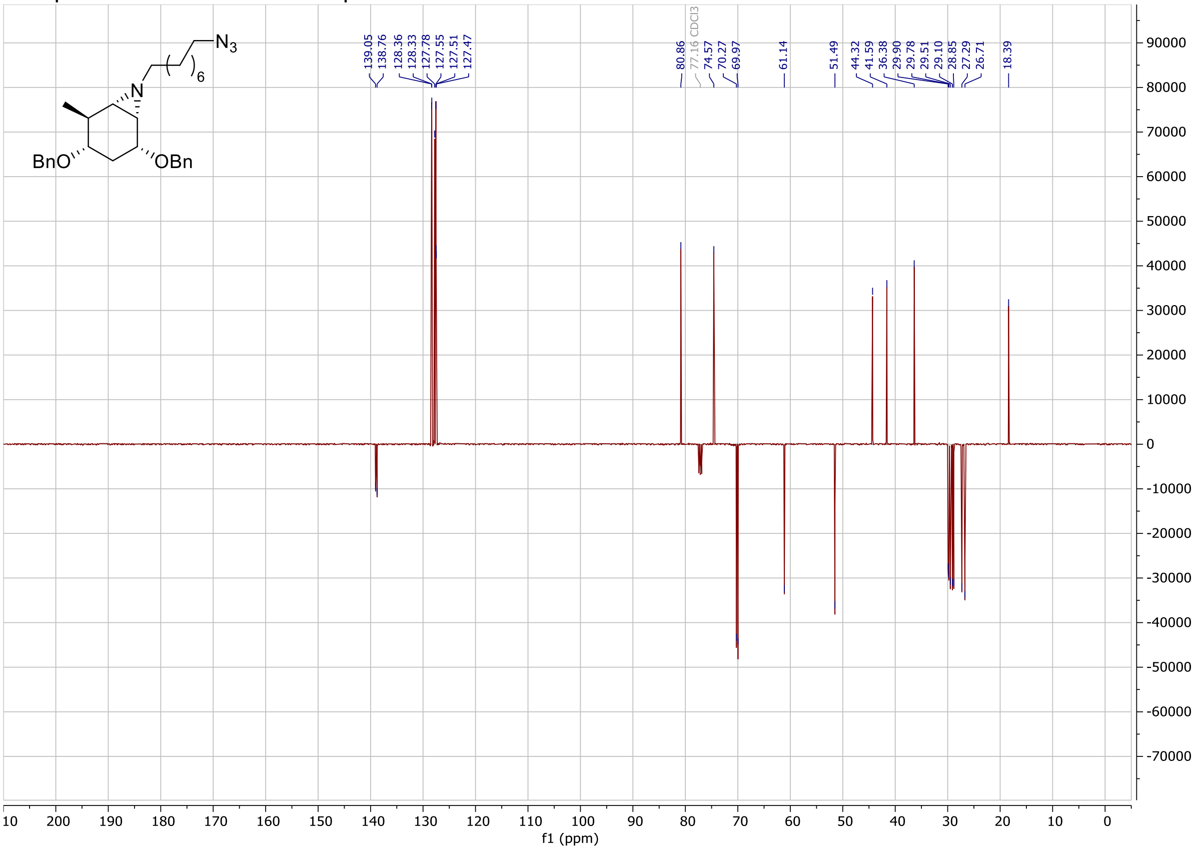

Compound **s90** ^{1}H-^{1}H COSY spectrum

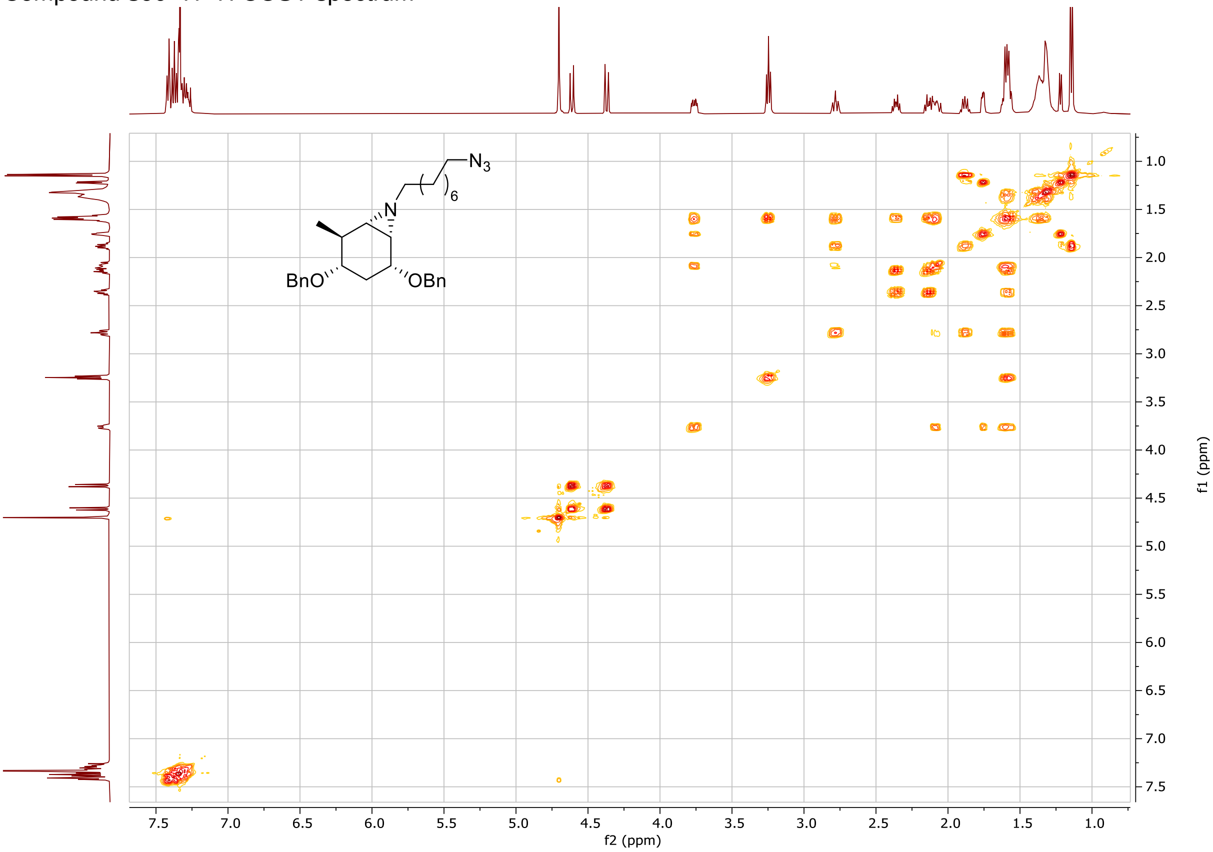

Compound **s90** ^{1}H-^{13}C HSQC spectrum

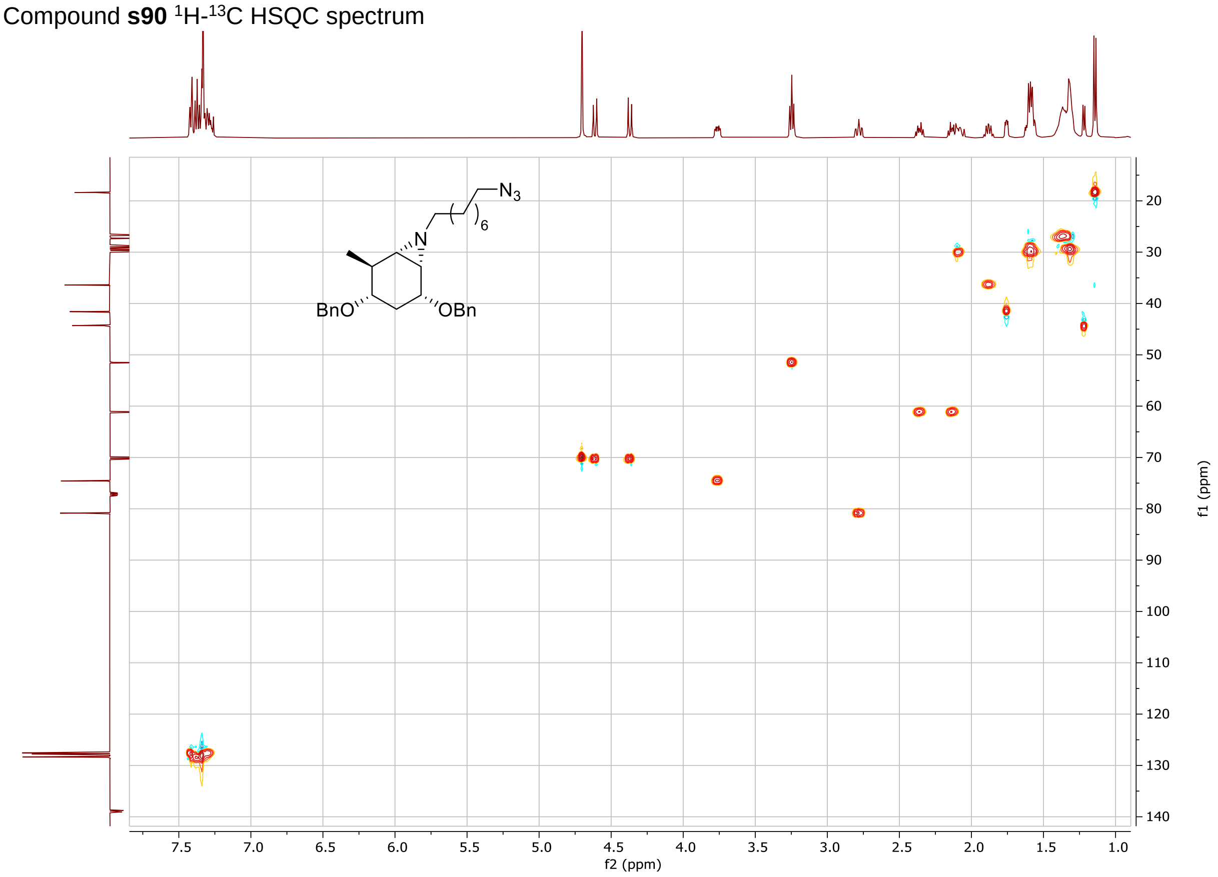

Compound **s90** ^{1}H-^{1}H NOESY spectrum

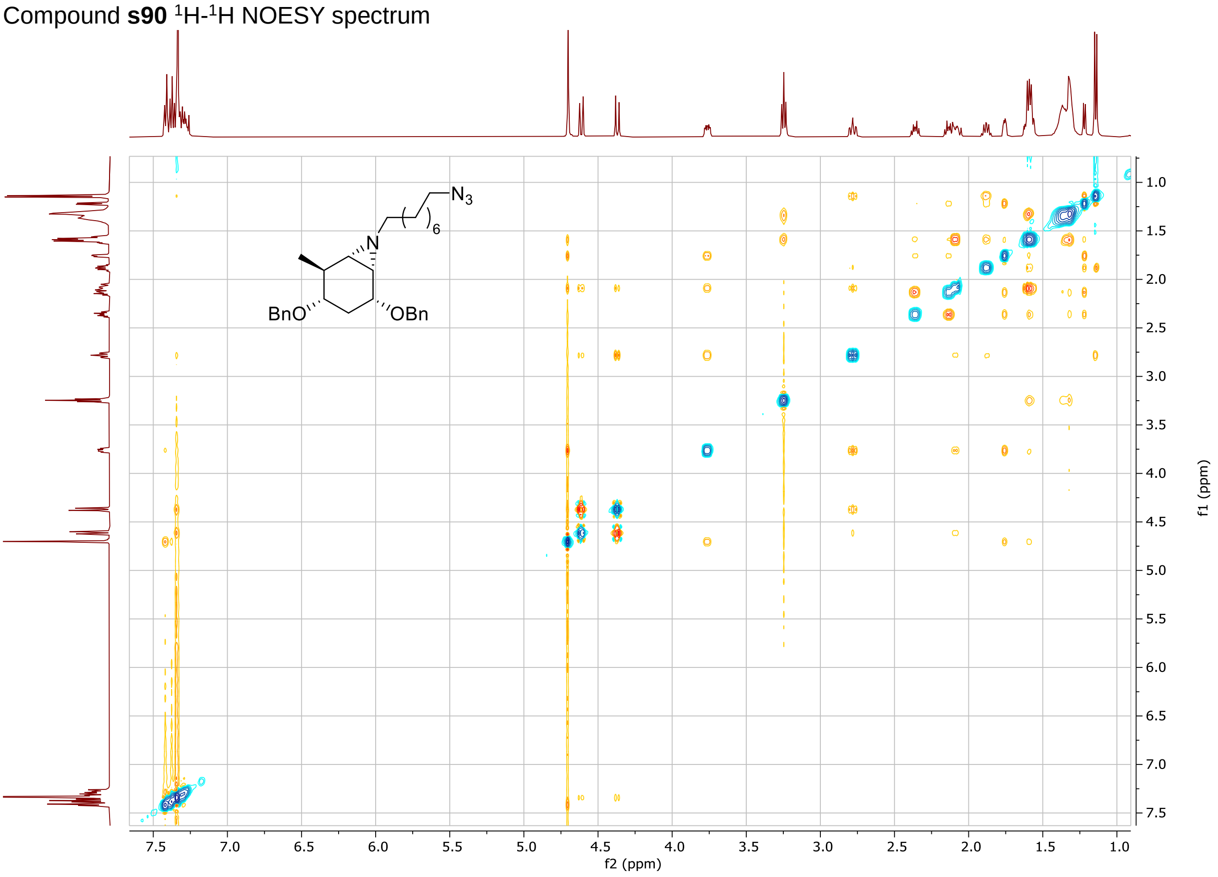

Compound **s91** ^{1}H NMR spectrum

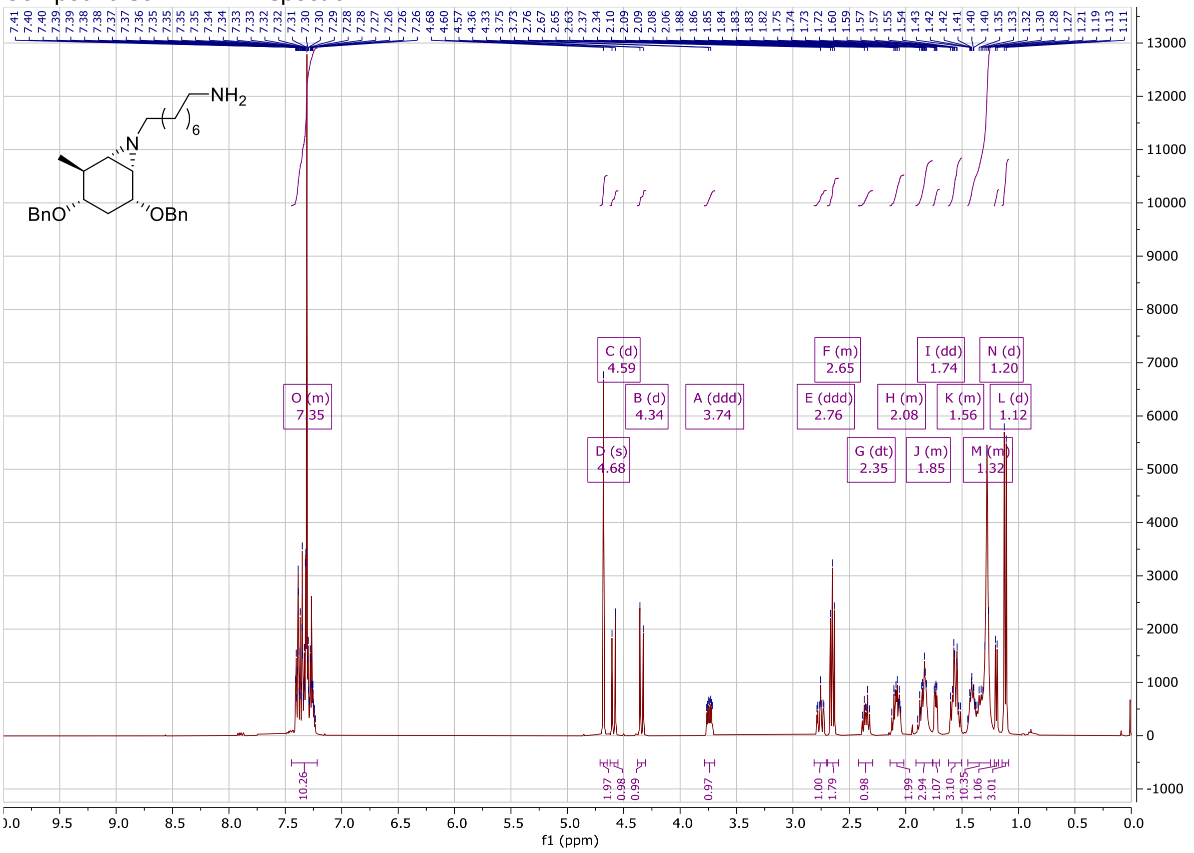

Compound **s91** ^{13}C NMR APT spectrum

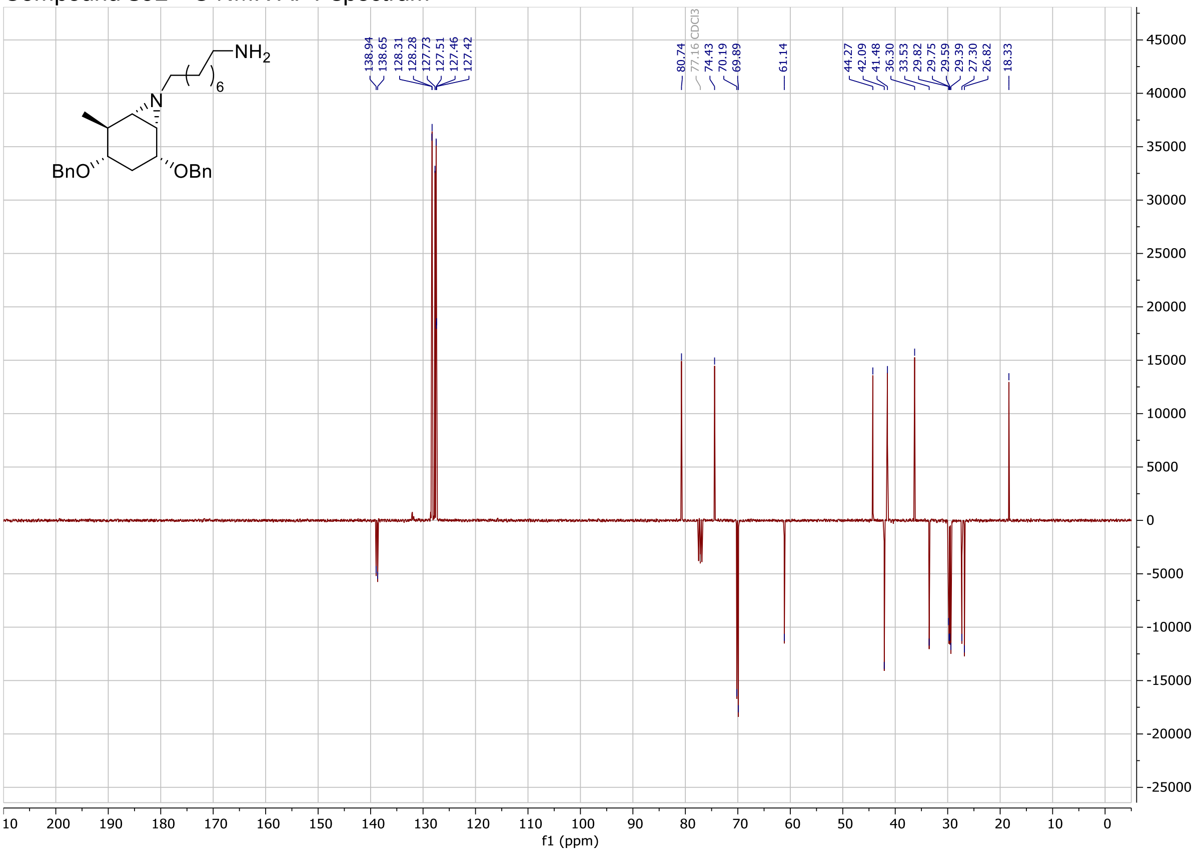

Compound **s91** ^{1}H-^{1}H COSY spectrum

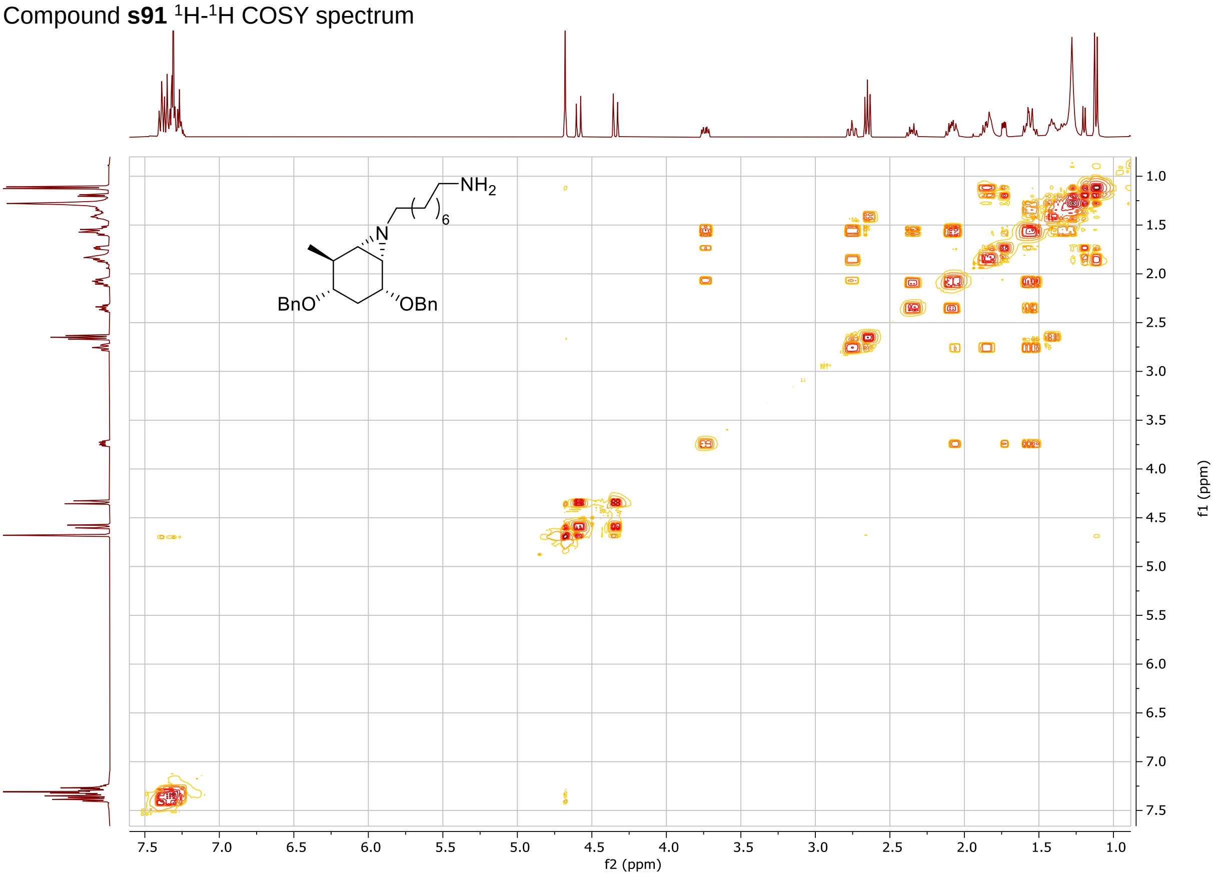

Compound **s91** ^{1}H-^{13}C HSQC spectrum

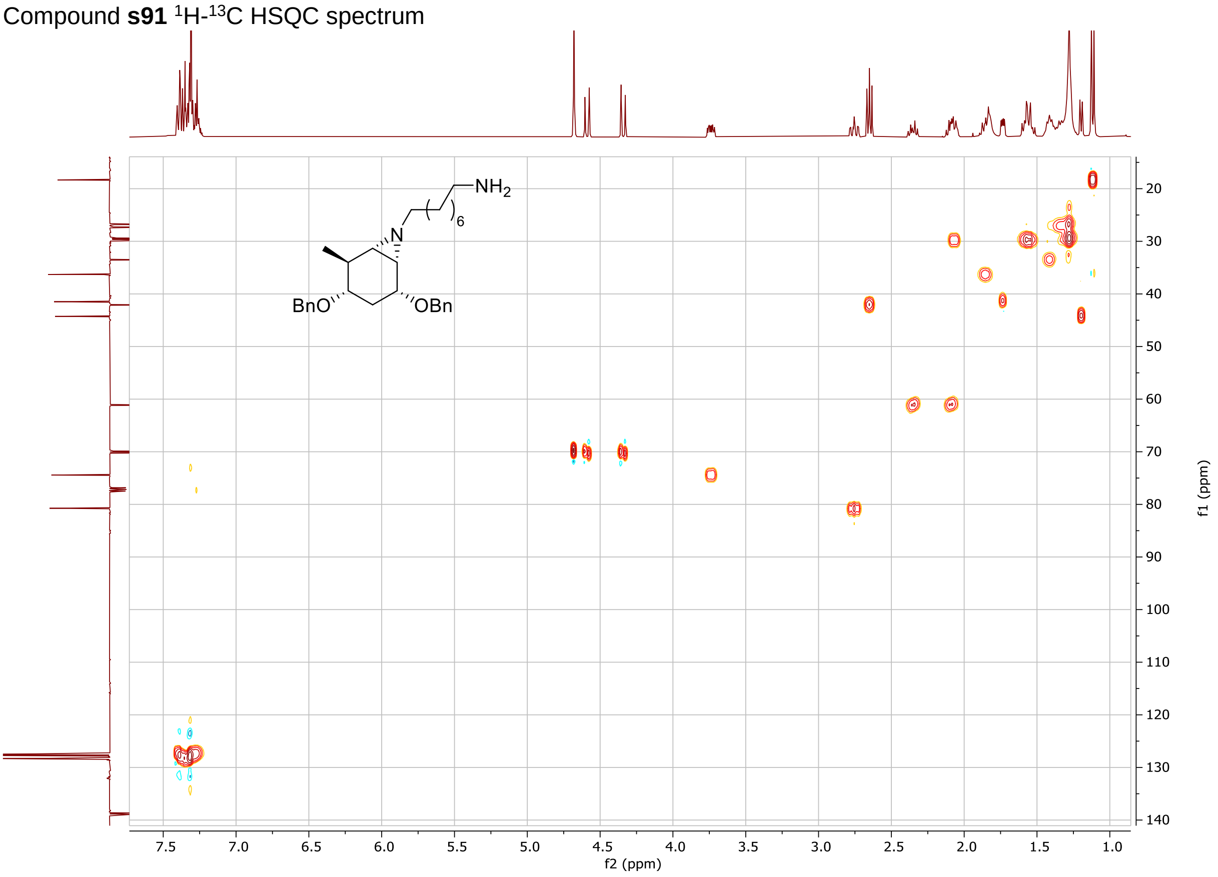

Compound **s92** ^{1}H NMR spectrum

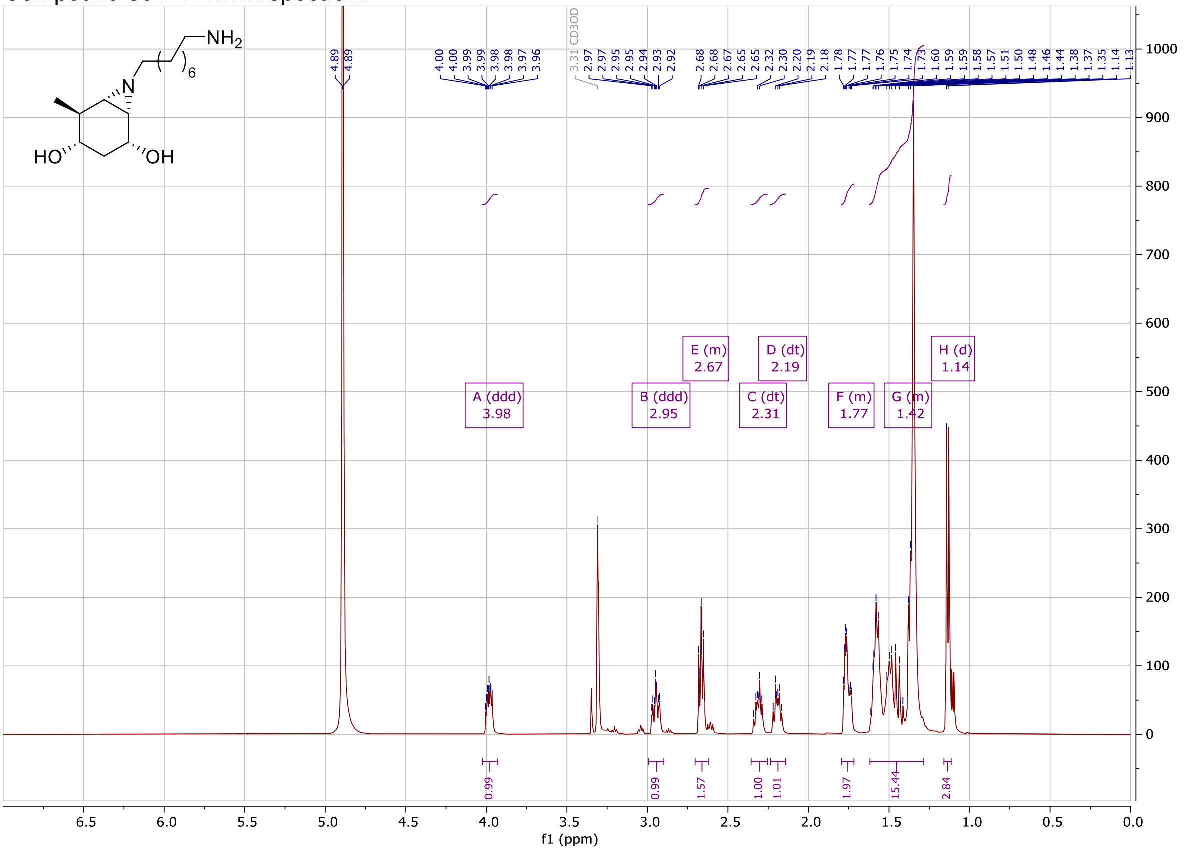

Compound **s92** ^{13}C NMR APT spectrum

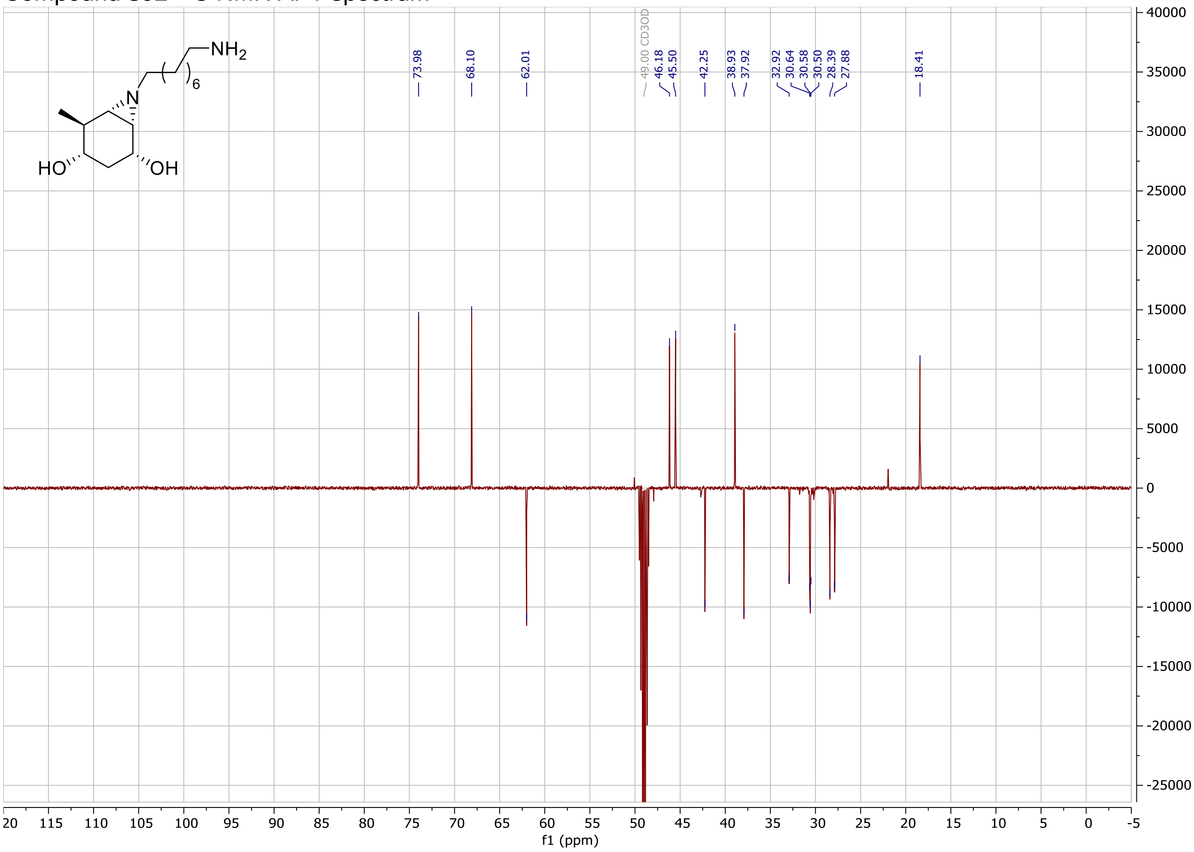

Compound **s92** ^{1}H-^{1}H COSY spectrum

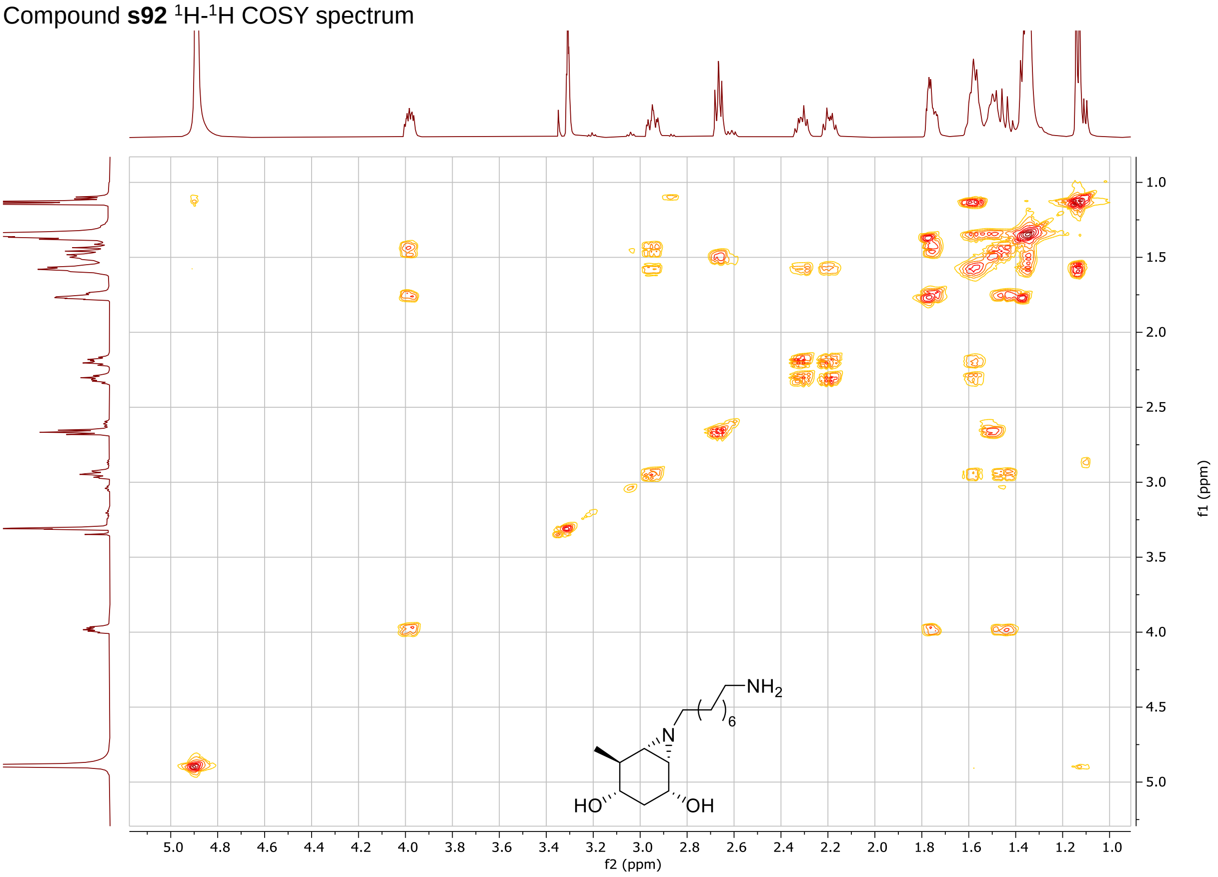

Compound **s92** ^{1}H-^{13}C HSQC spectrum

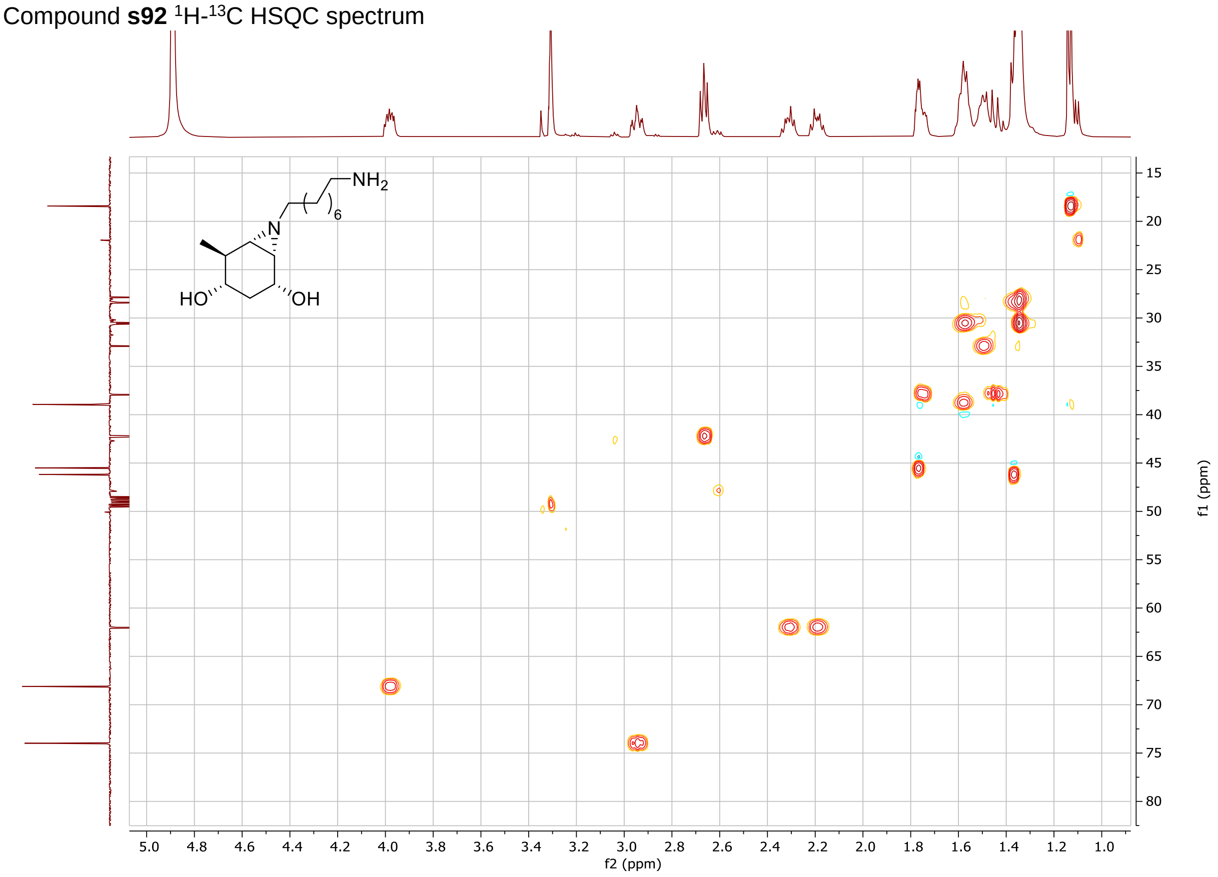

Compound **52** ^{1}H NMR spectrum

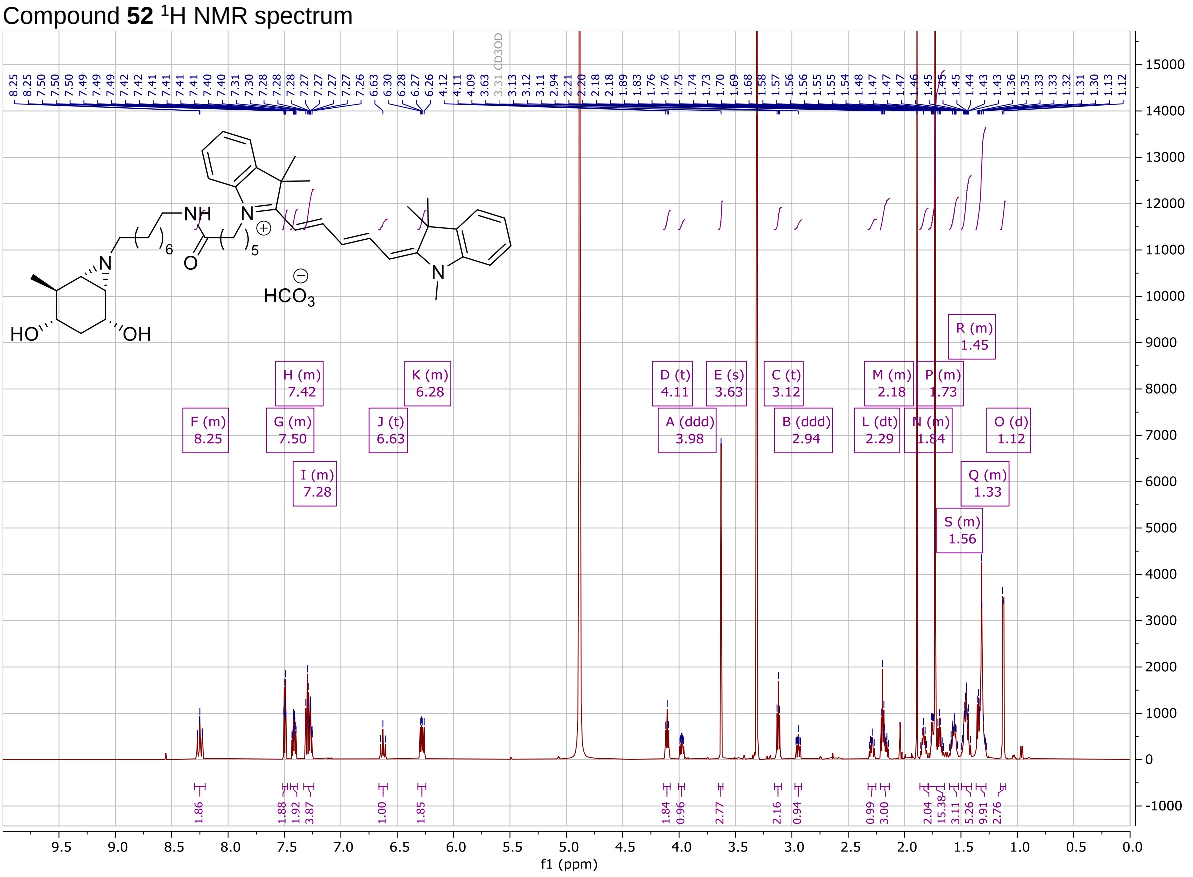

Compound **52** ^{13}C NMR APT spectrum

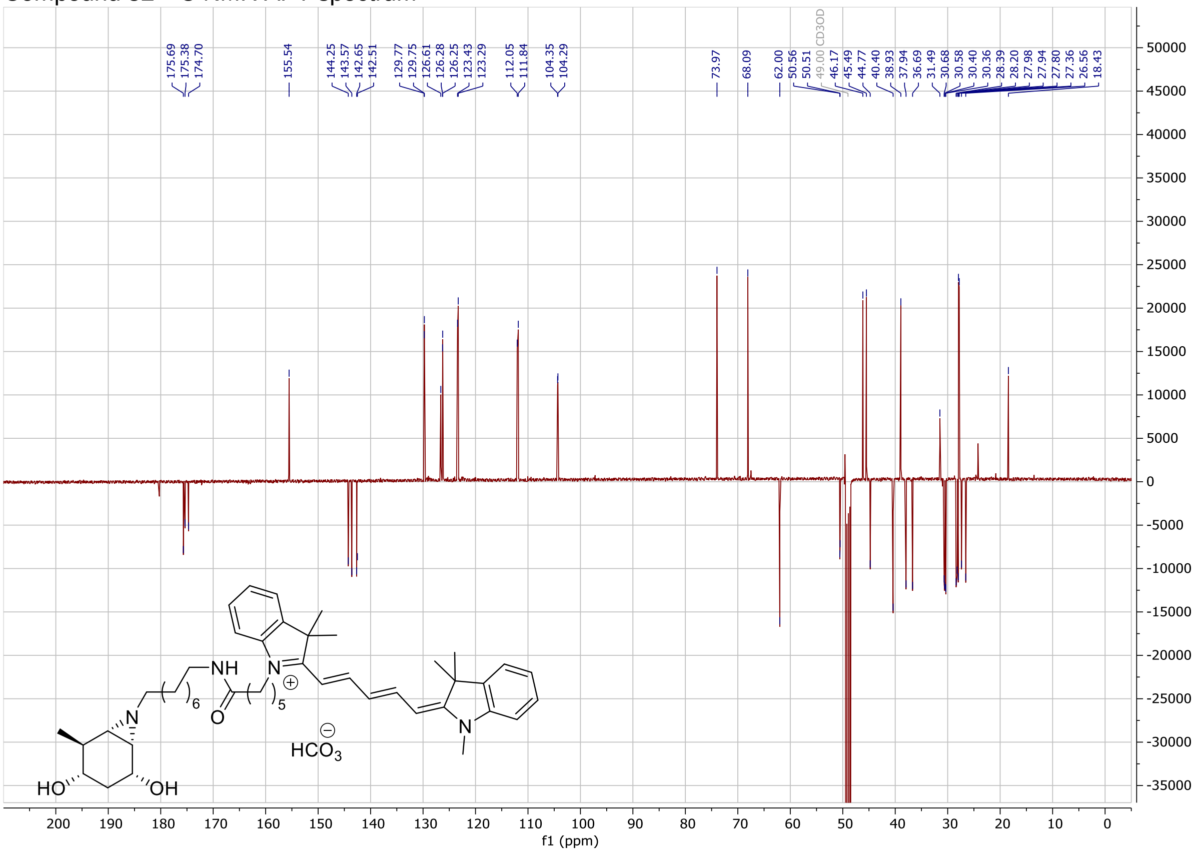

Compound **52** ^{1}H-^{1}H COSY spectrum

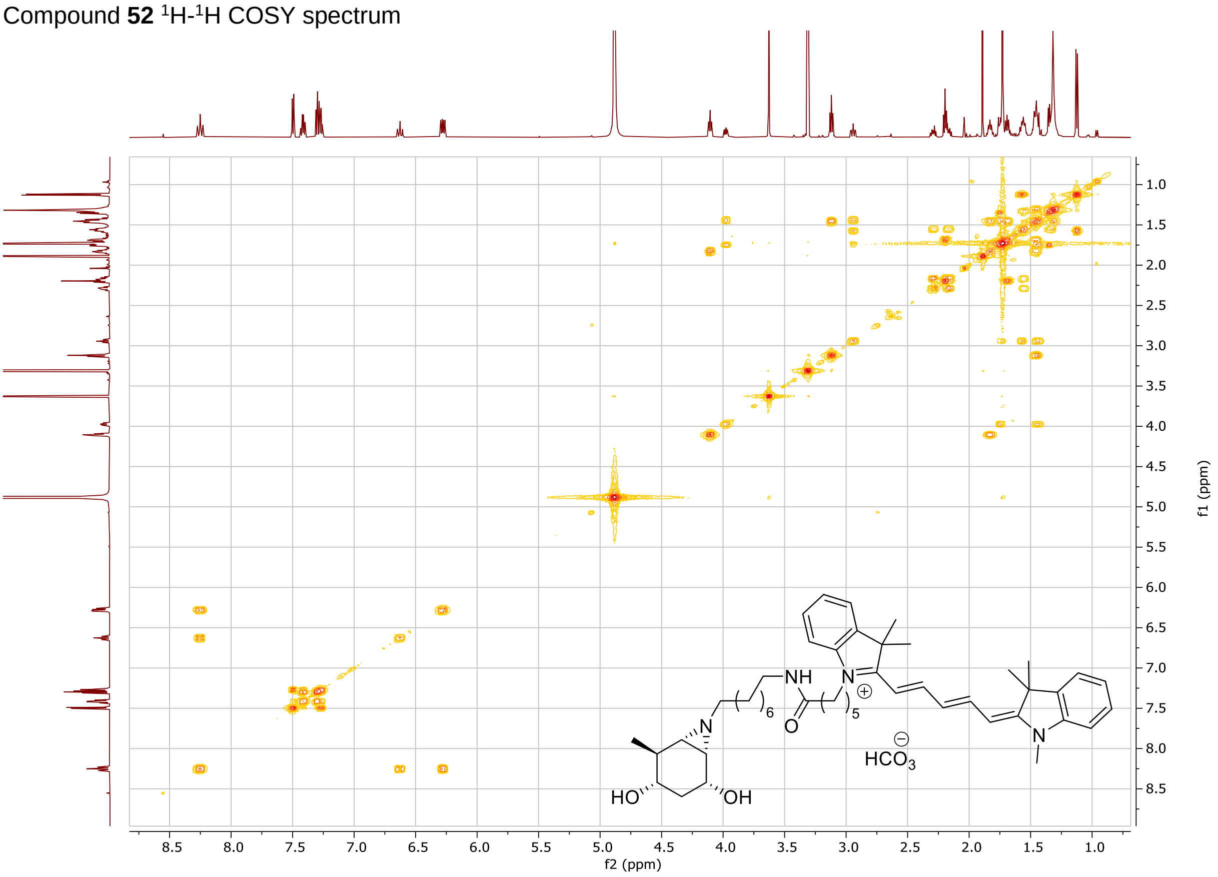

Compound **52** ^{1}H-^{13}C HSQC spectrum

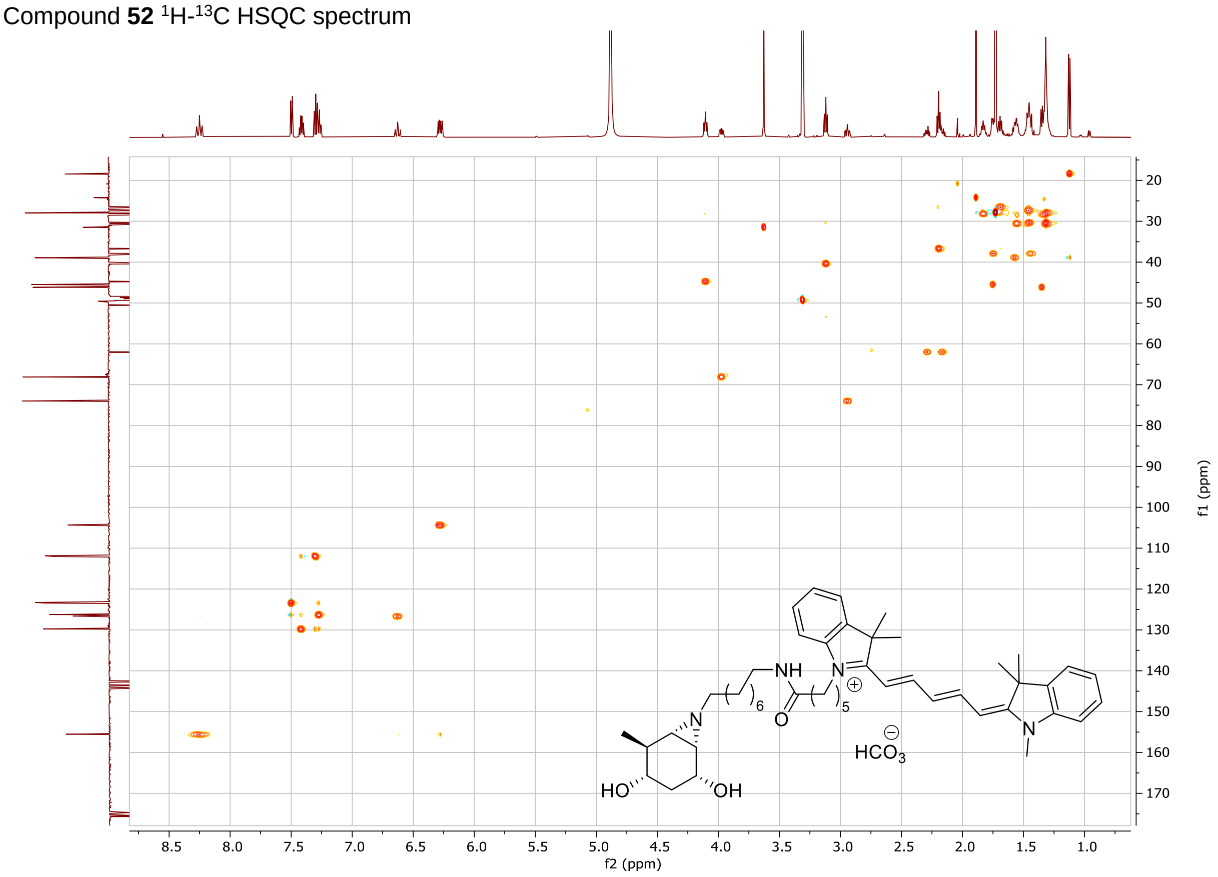

Compound **59** ^{1}H NMR spectrum

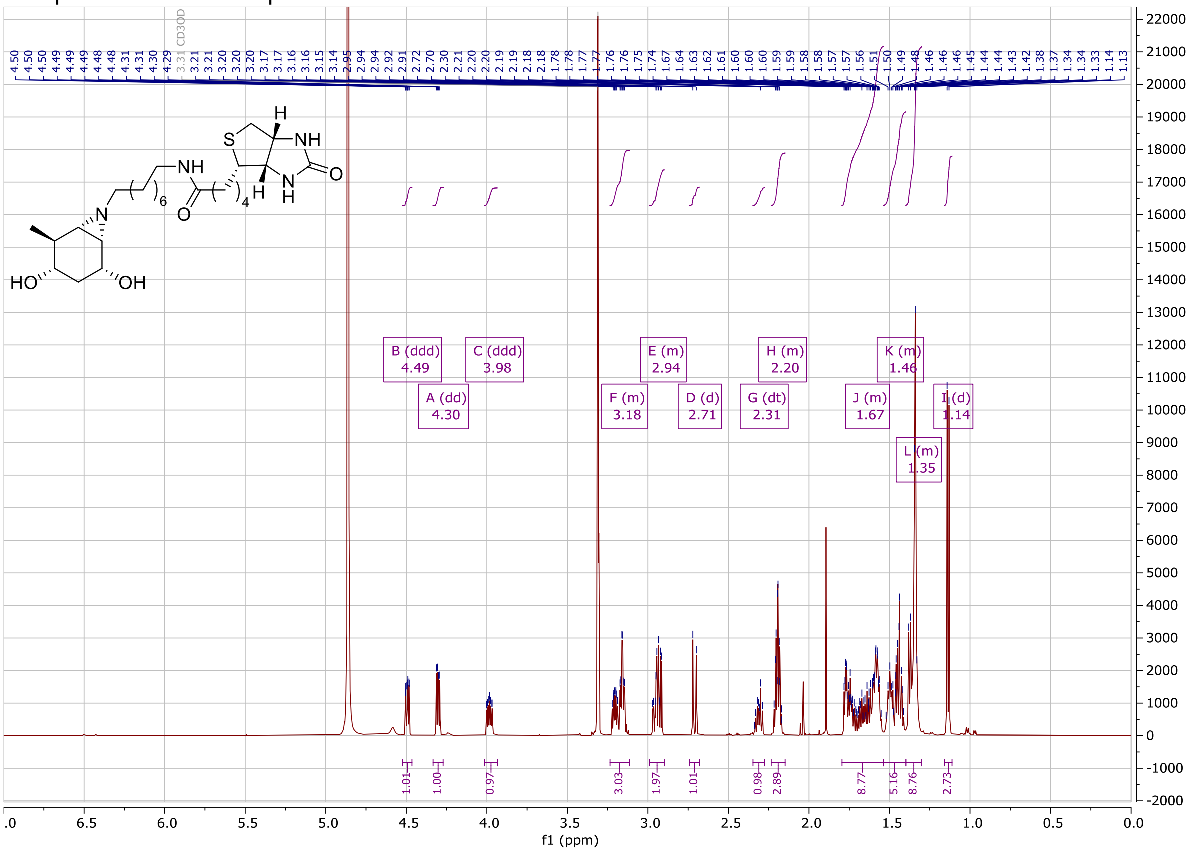

Compound **59** ^{13}C NMR APT spectrum

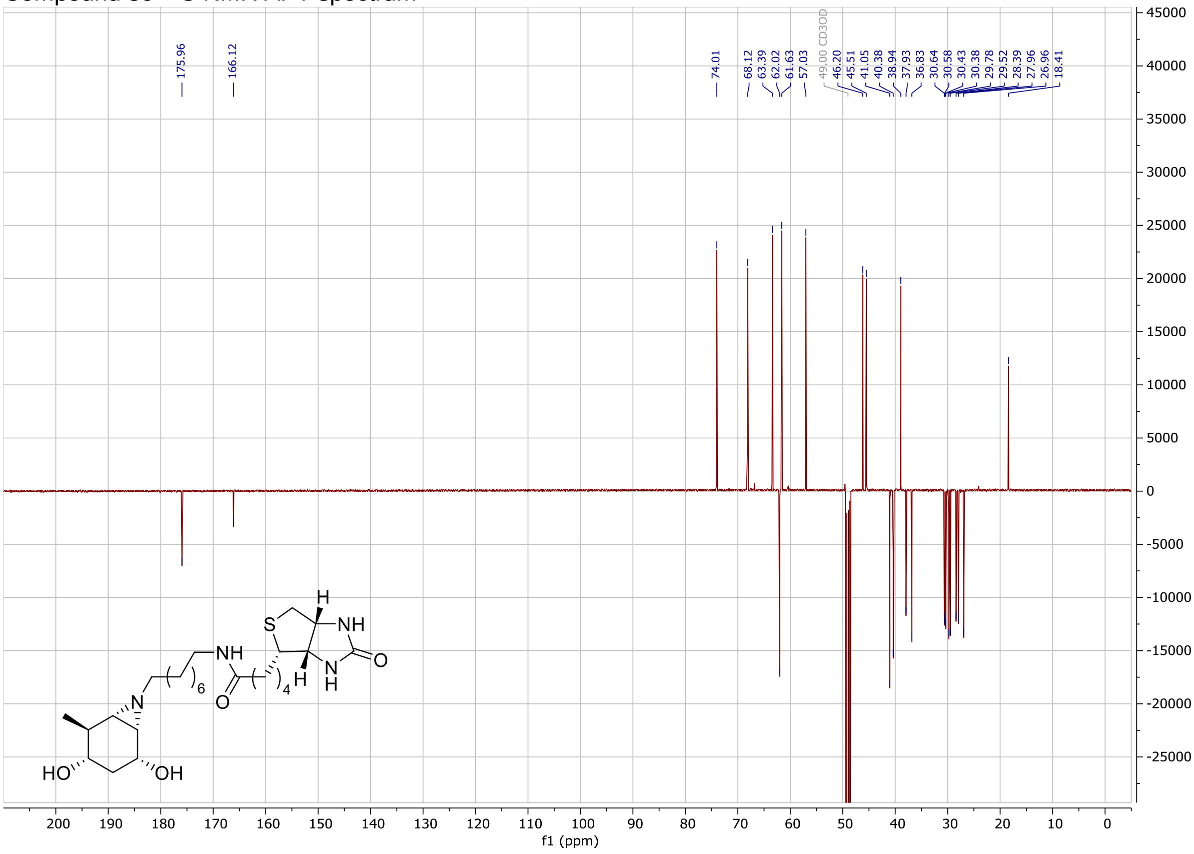

Compound **59** ^{1}H-^{1}H COSY spectrum

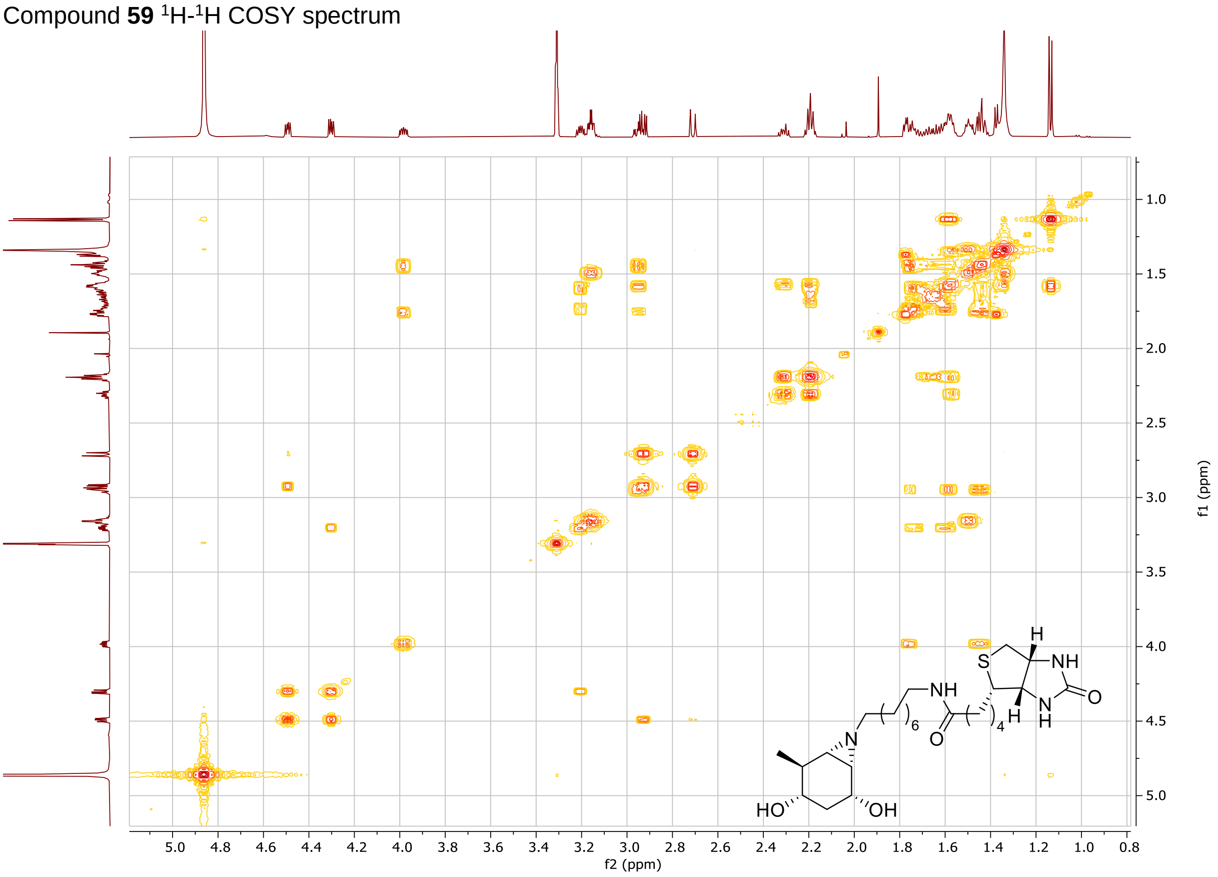

Compound **59** ^{1}H-^{13}C HSQC spectrum

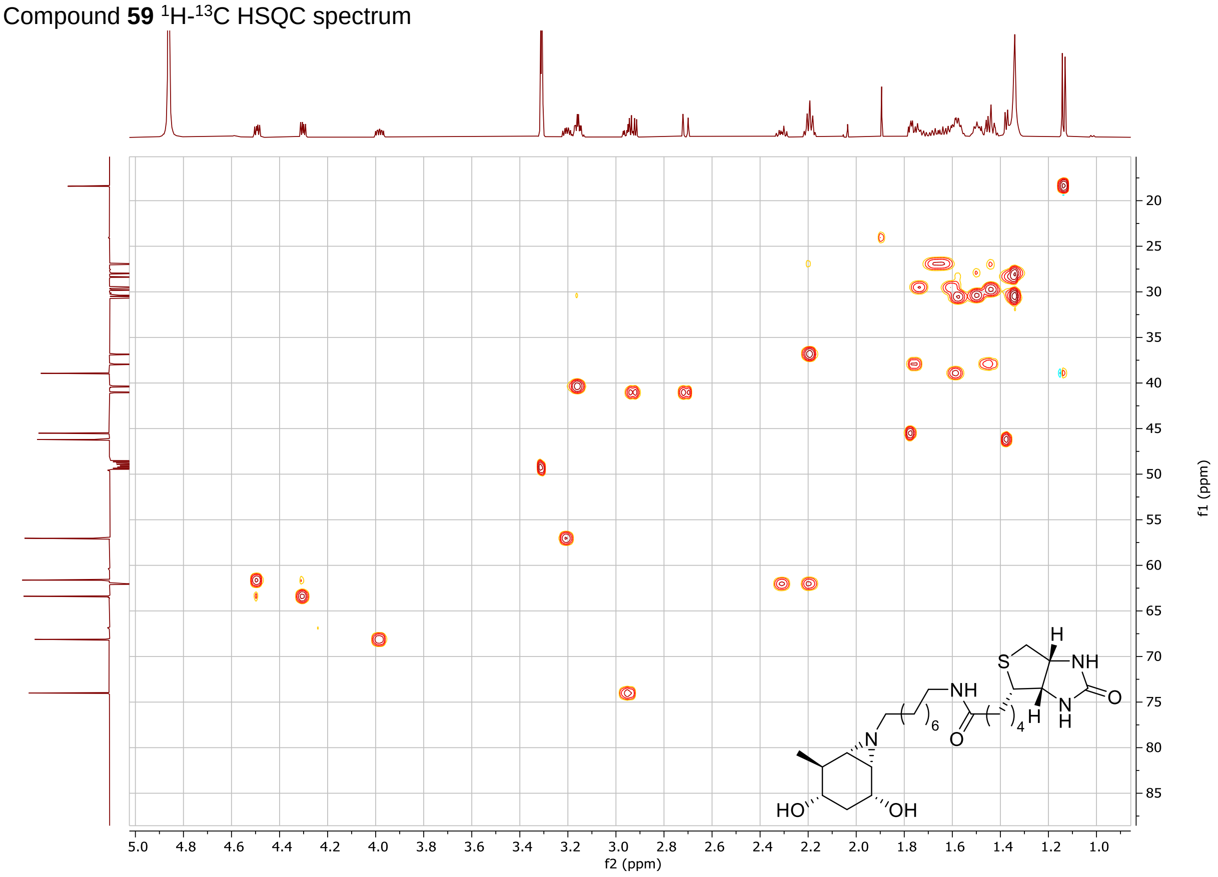

Compounds **s93** and **s94** ^{1}H NMR spectrum (326 K)

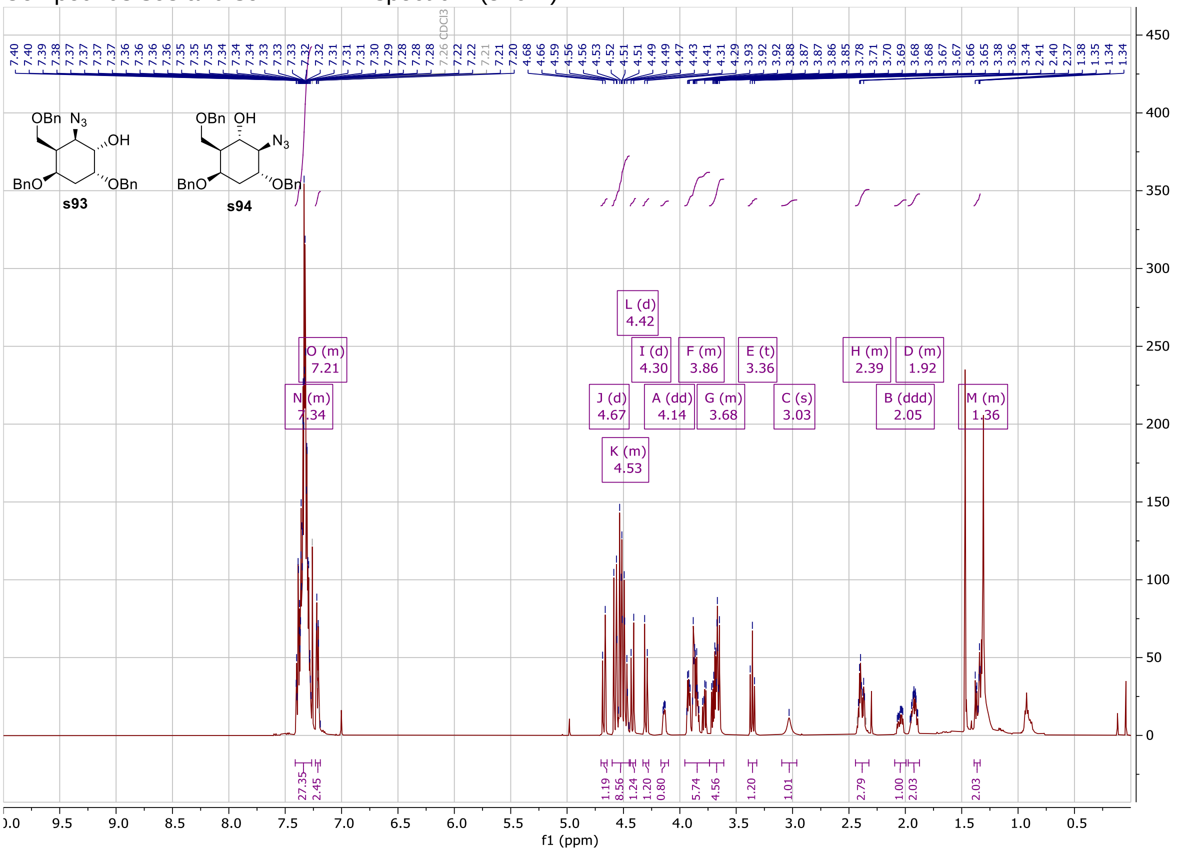

Compounds **s93** and **s94** ^{13}C NMR APT spectrum (326 K)

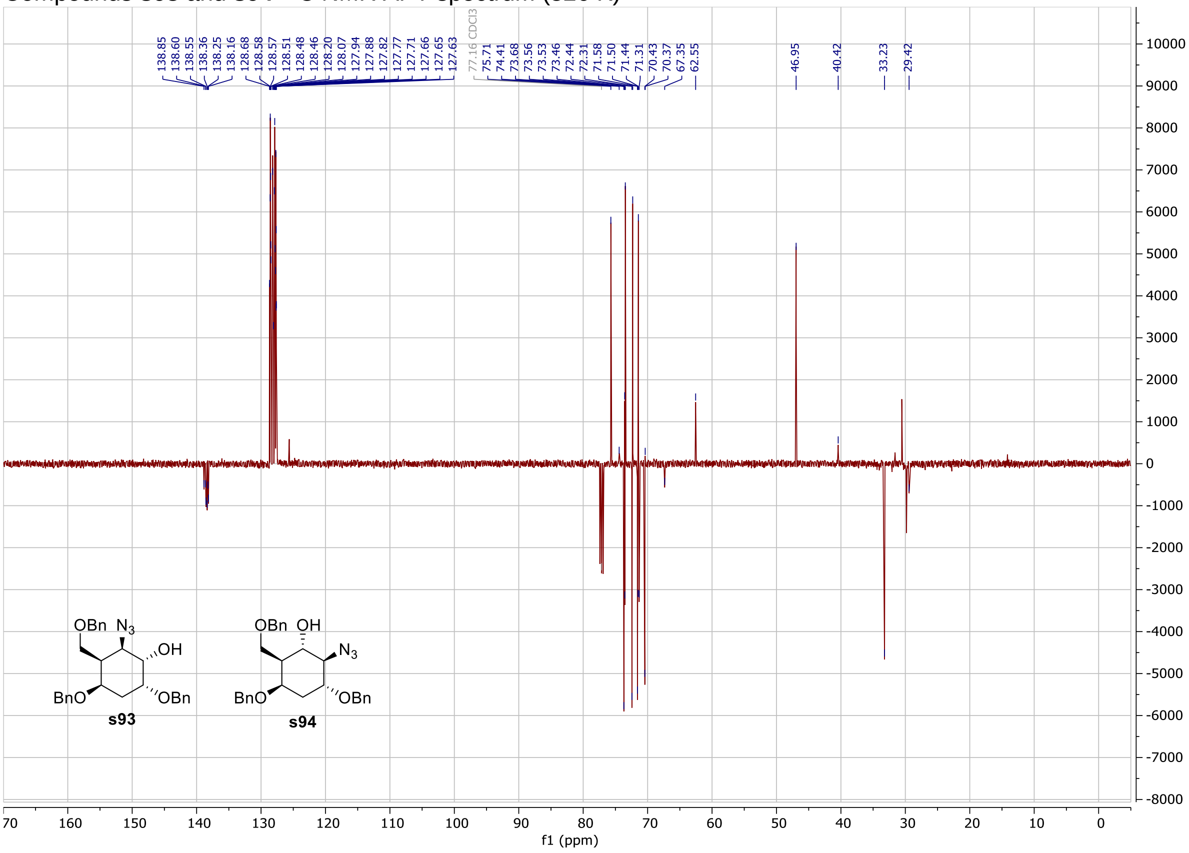

Compounds **s93** and **s94** ^{1}H-^{13}C HSQC spectrum (326 K)

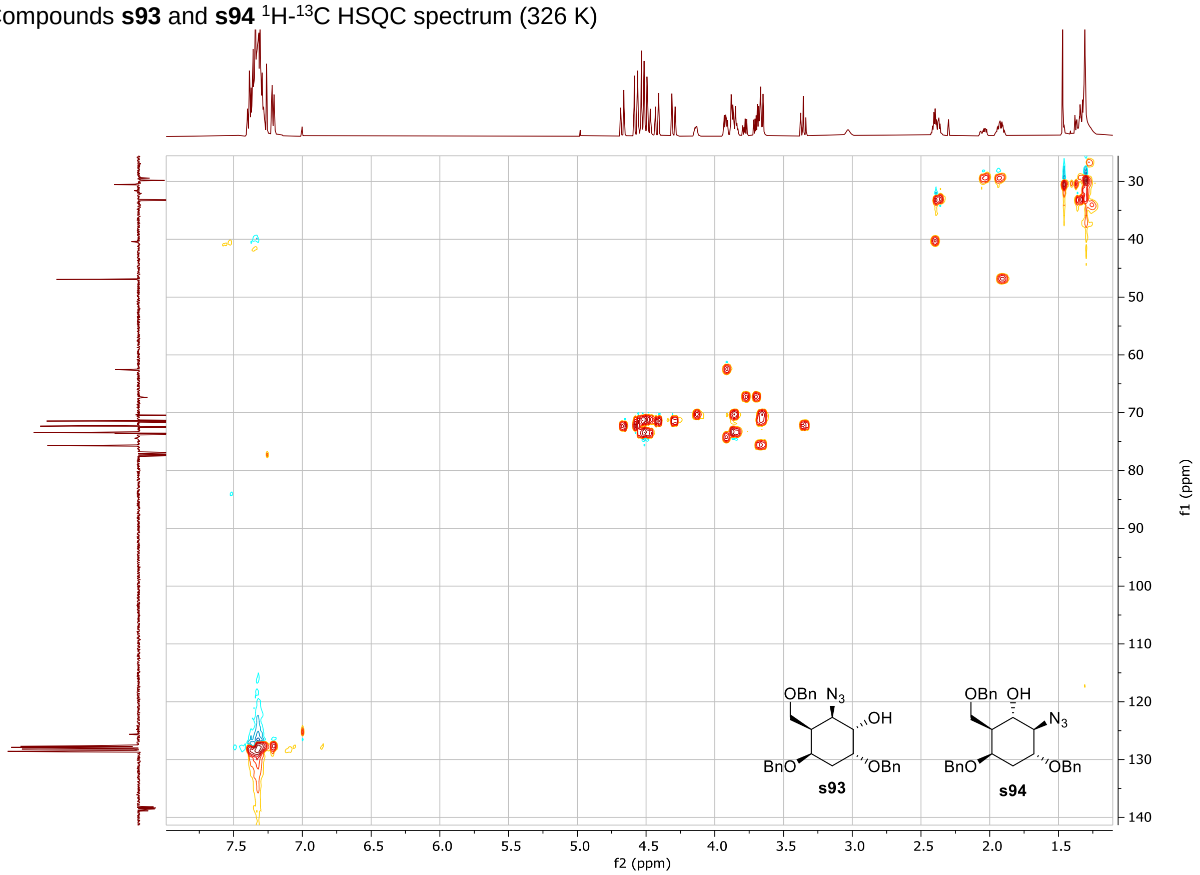

Compound **s95** ^{1}H NMR spectrum

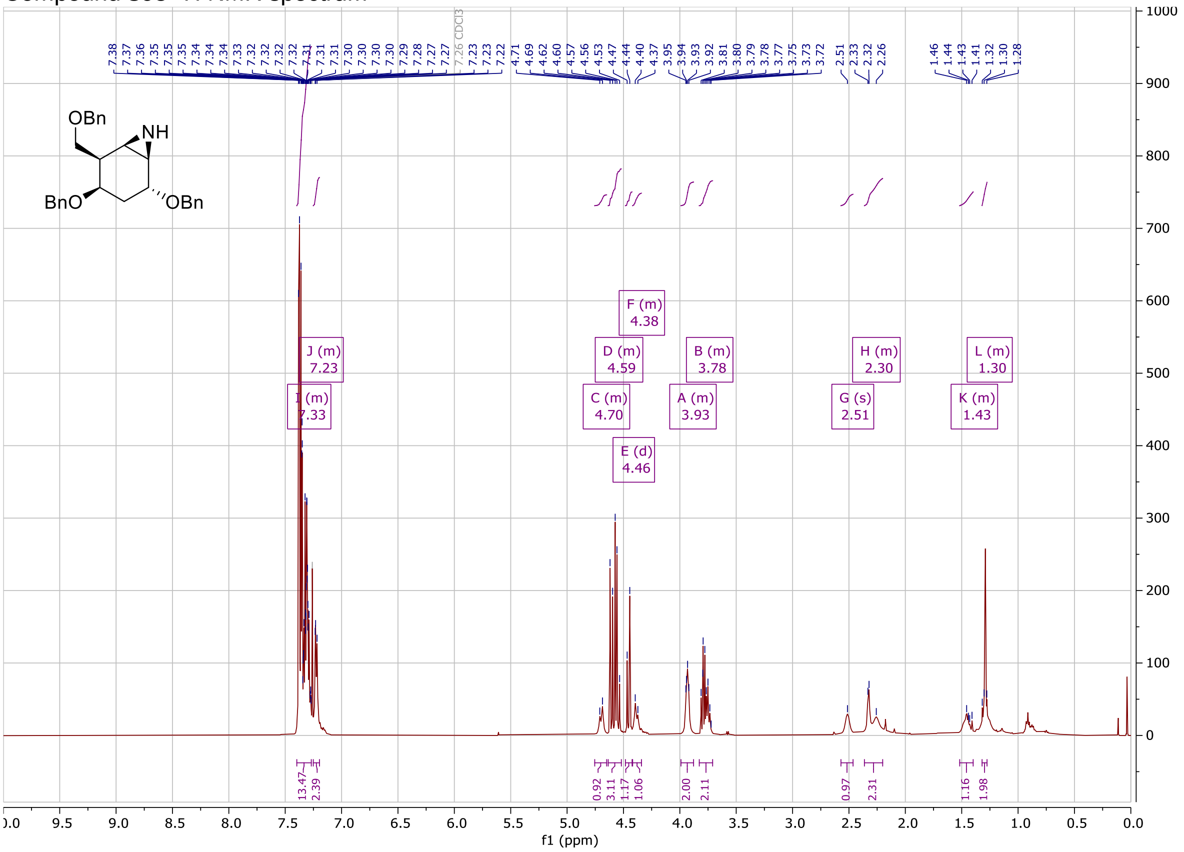

Compound **s95** ^{13}C NMR APT spectrum

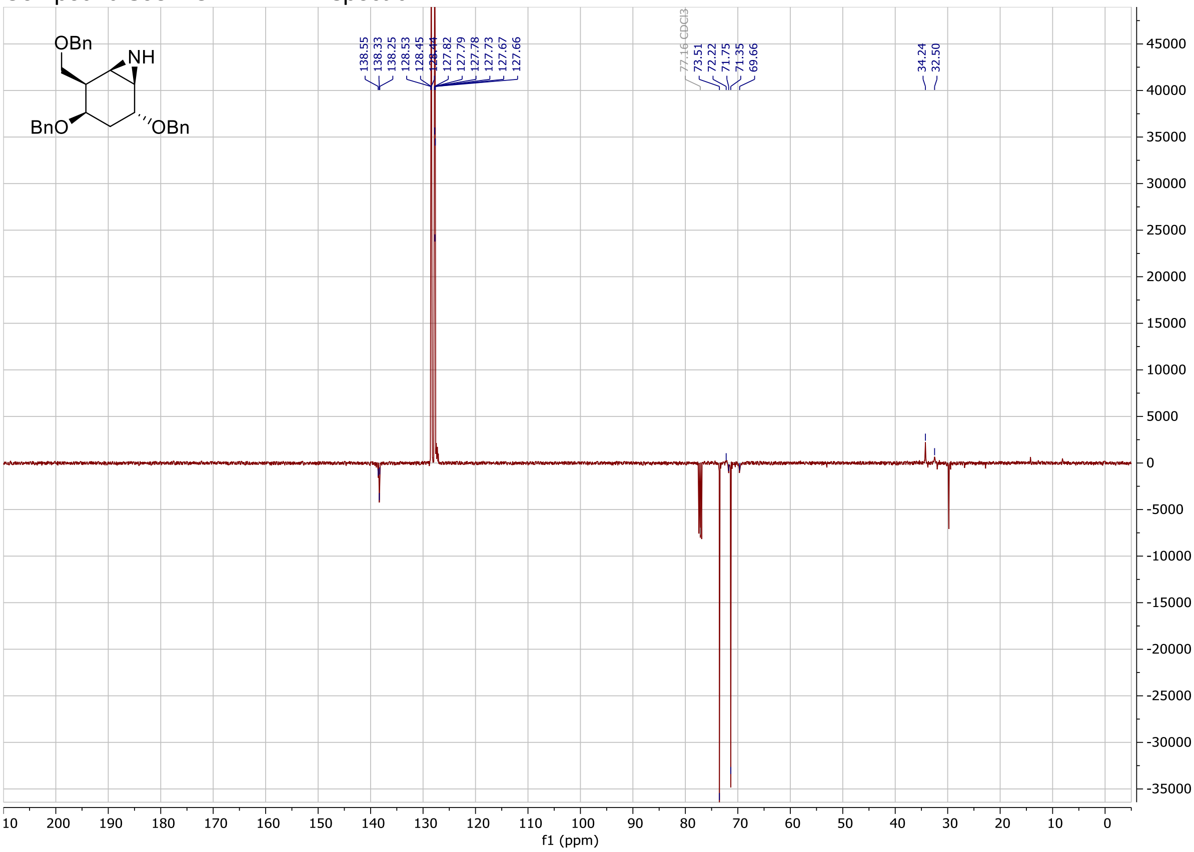

Compound **s95** ^{1}H-^{1}H COSY spectrum

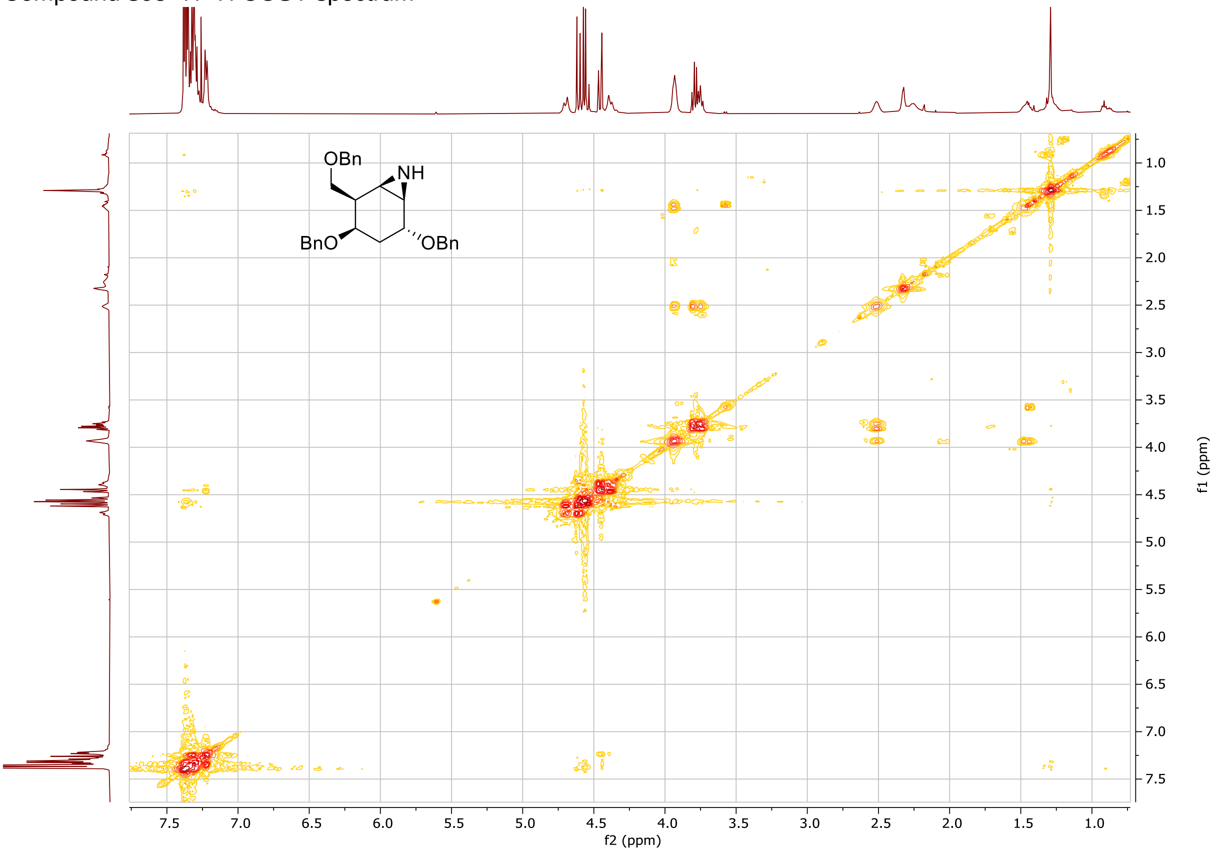

Compound **s95** ^{1}H-^{13}C HSQC spectrum

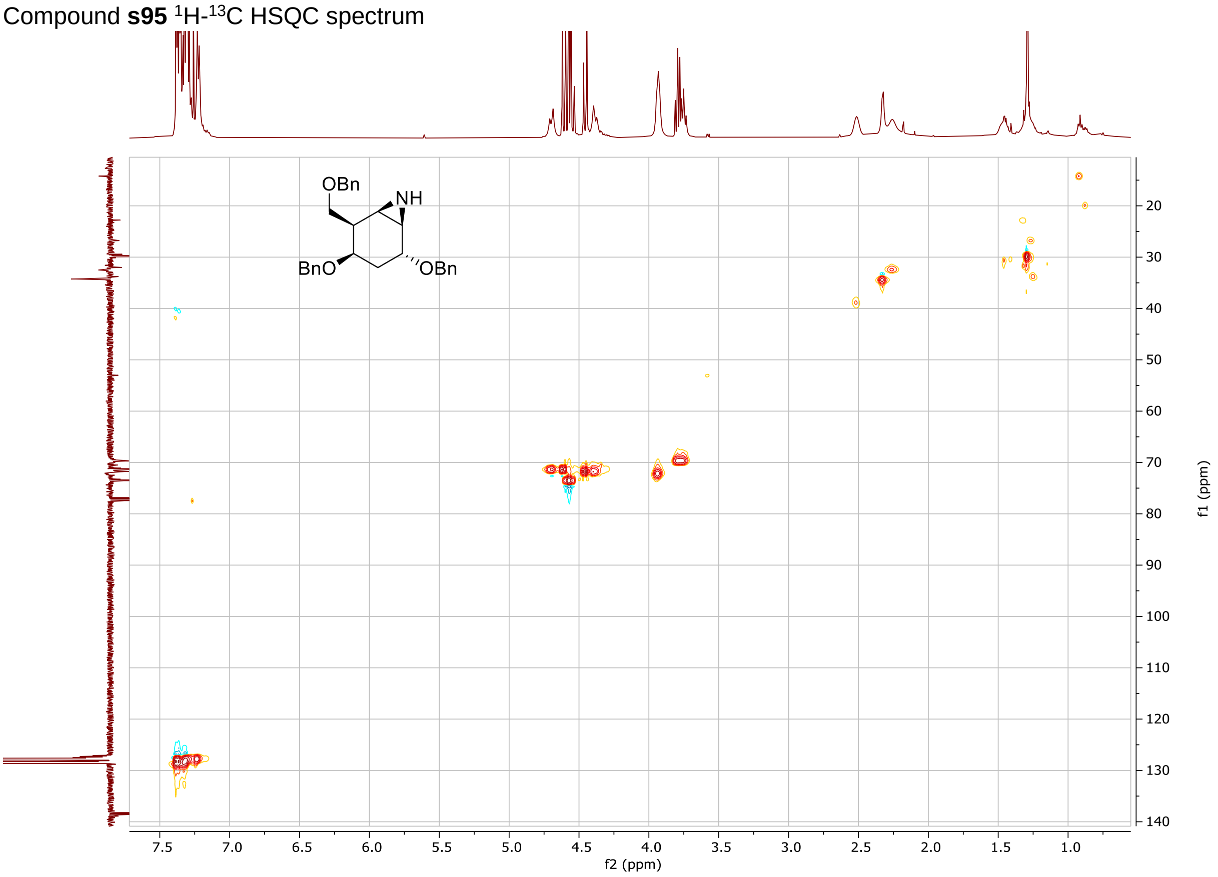

Compound **s96** ^{1}H NMR spectrum

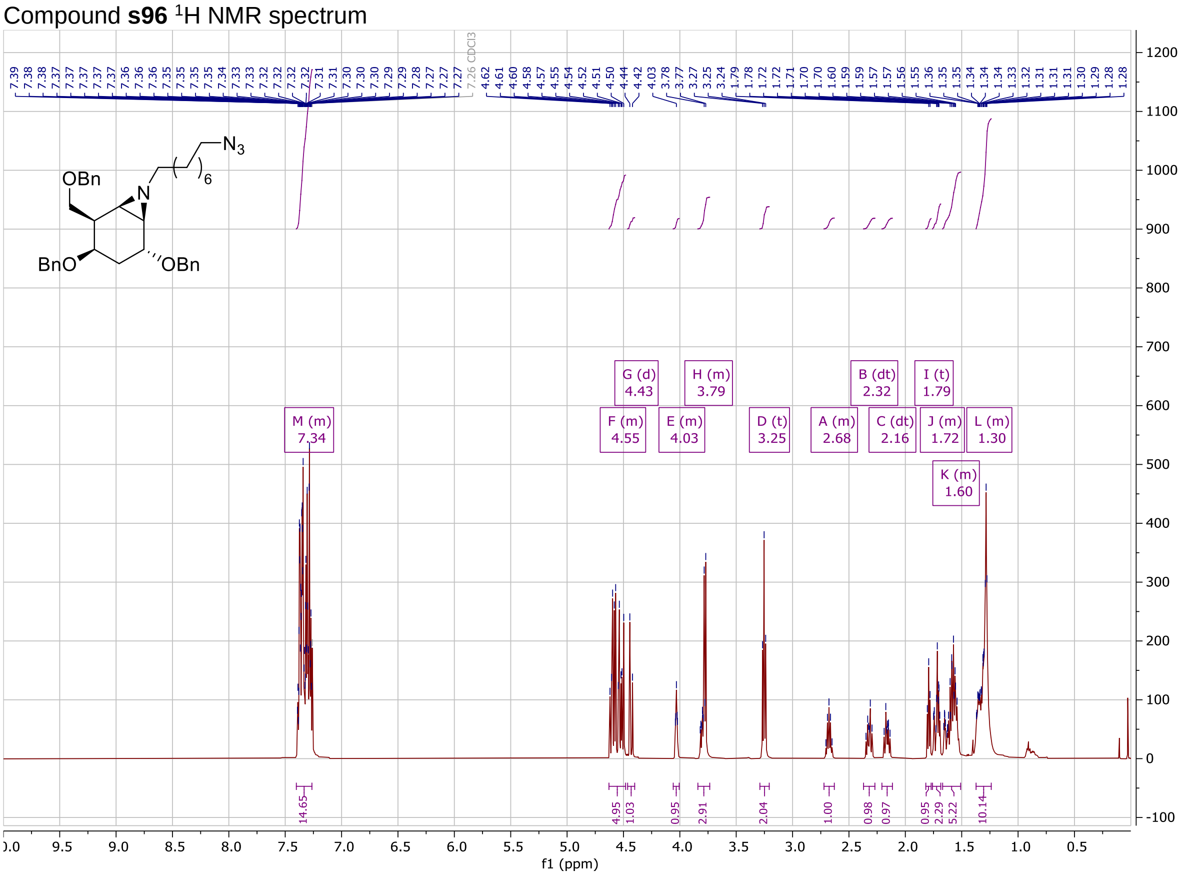

Compound **s96** ^{13}C NMR APT spectrum

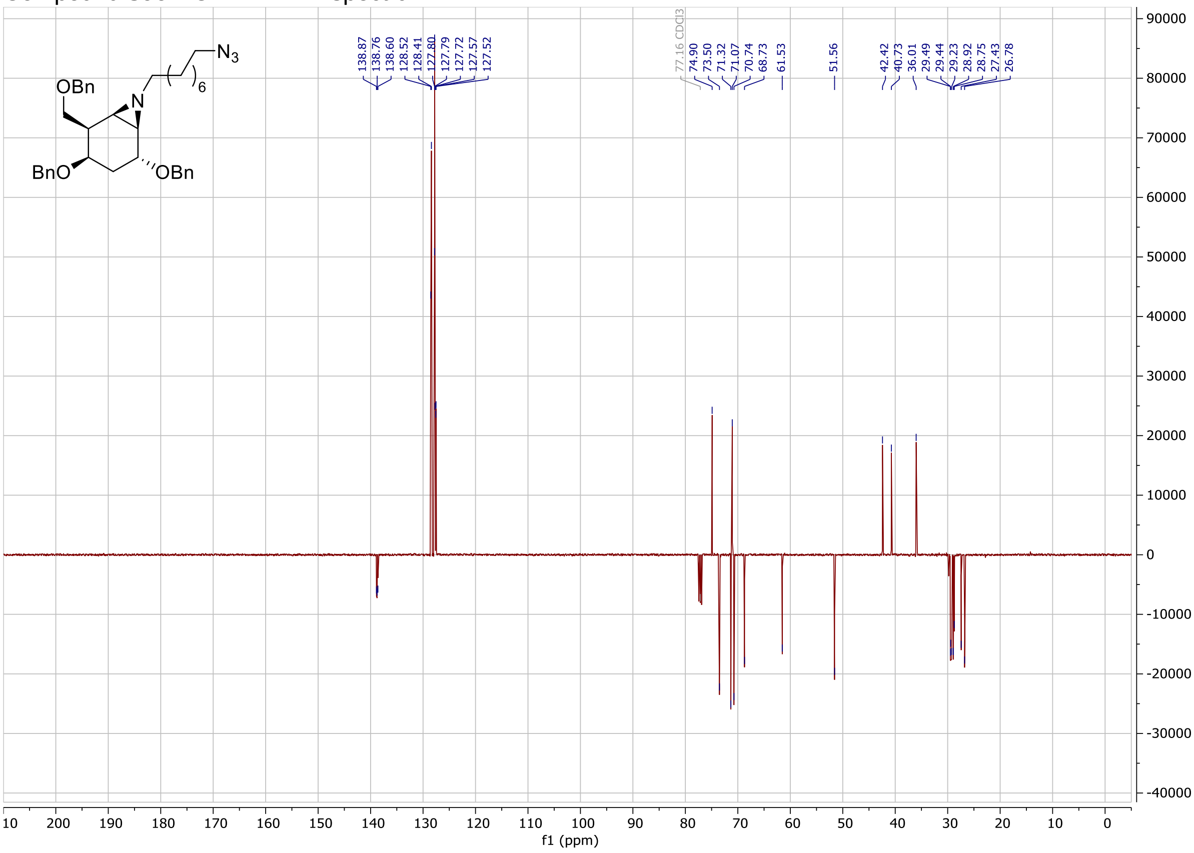

Compound **s96** ^{1}H-^{1}H COSY spectrum

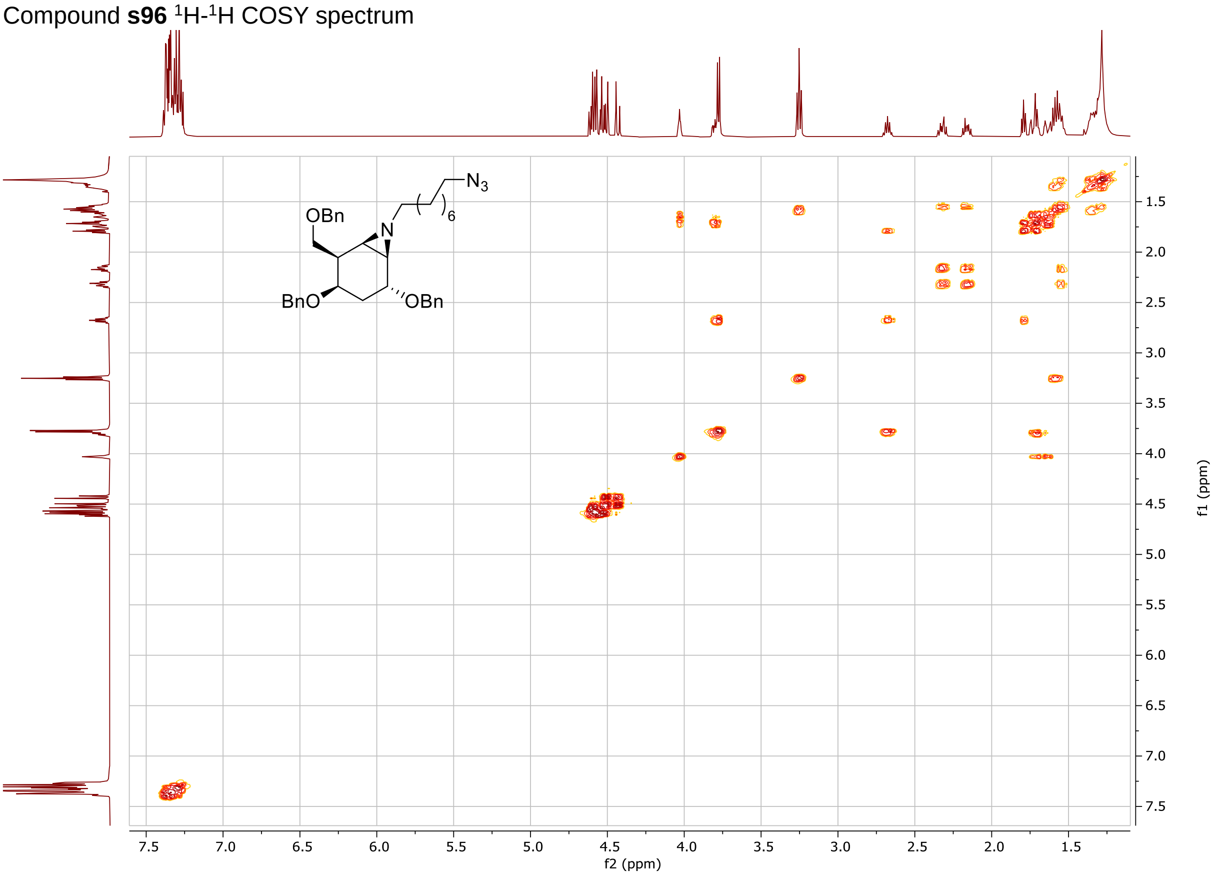

Compound **s96** ^{1}H-^{13}C HSQC spectrum

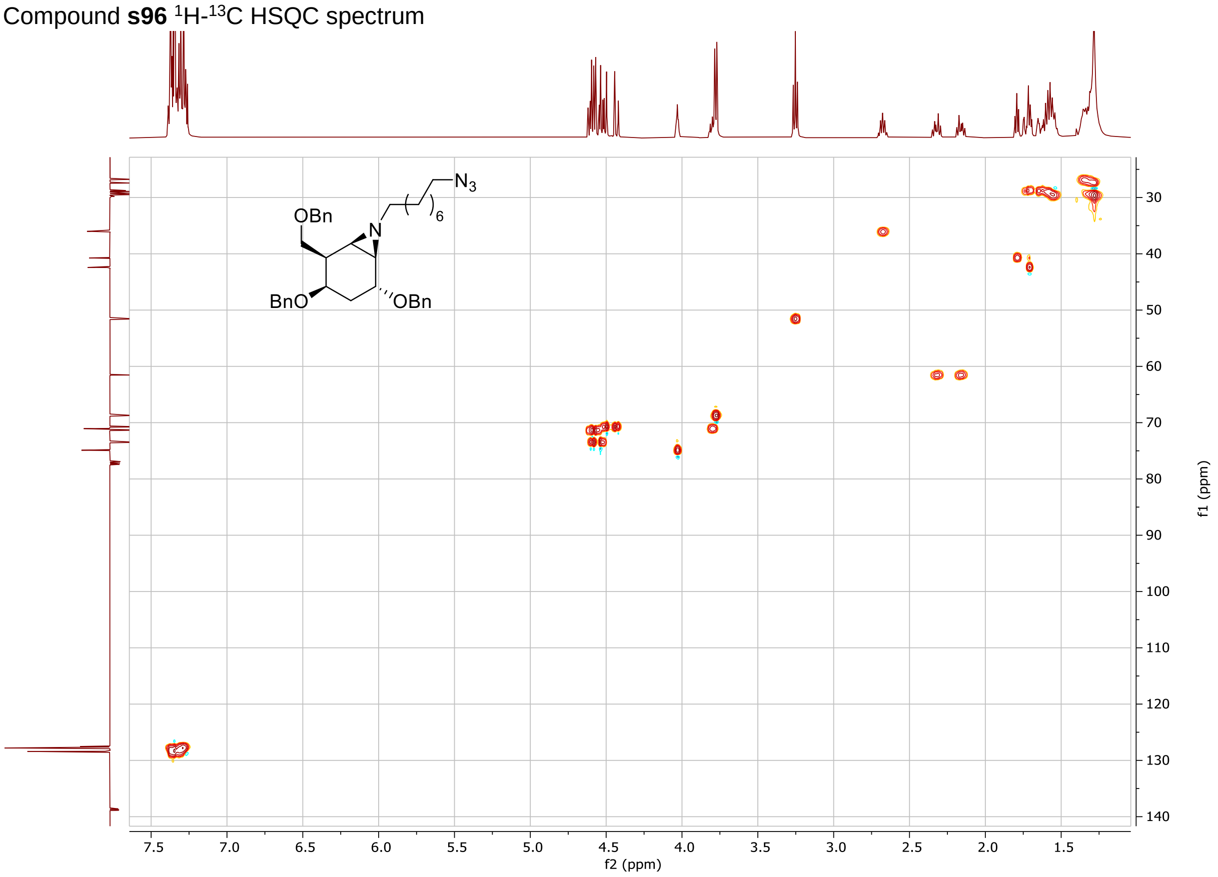

Compound **s96** ^{1}H-^{1}H NOESY spectrum

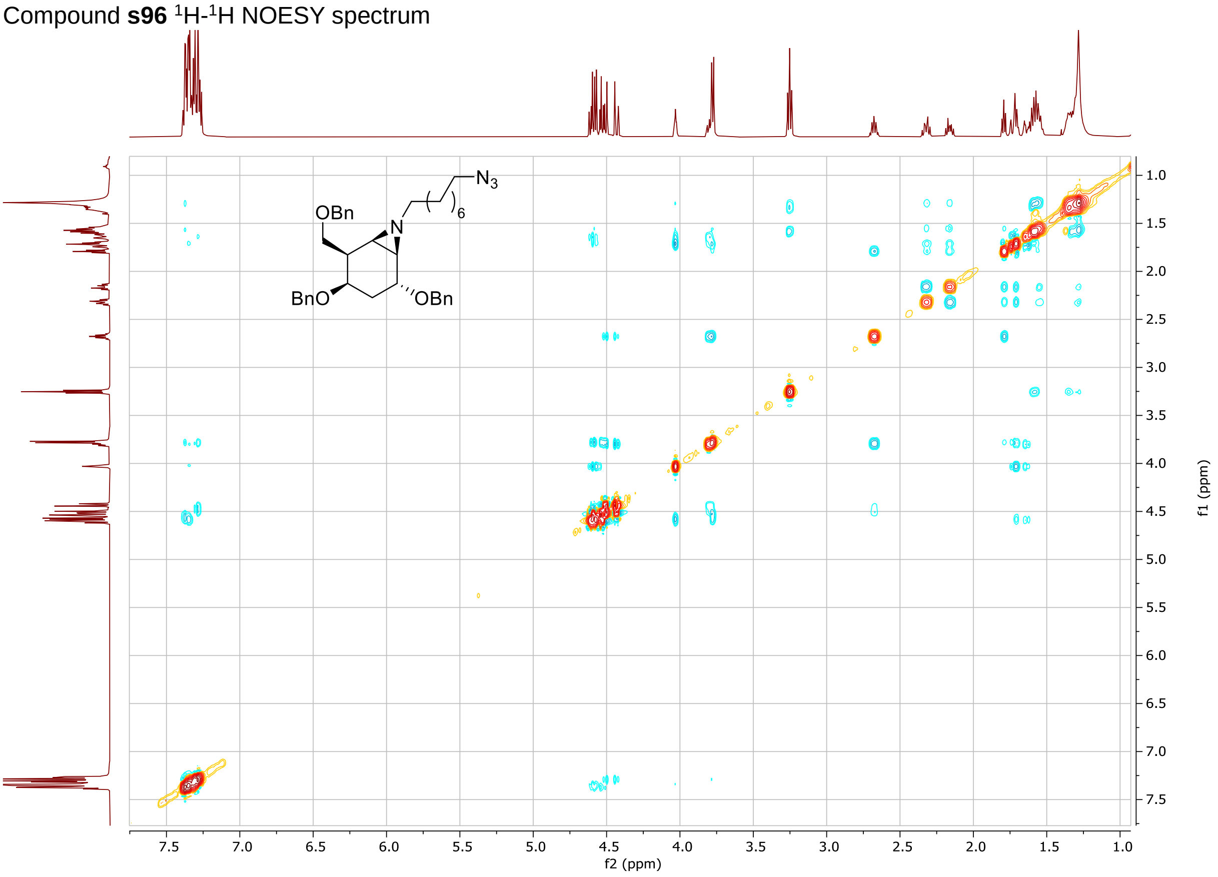

Compound **s97** ^{1}H NMR spectrum

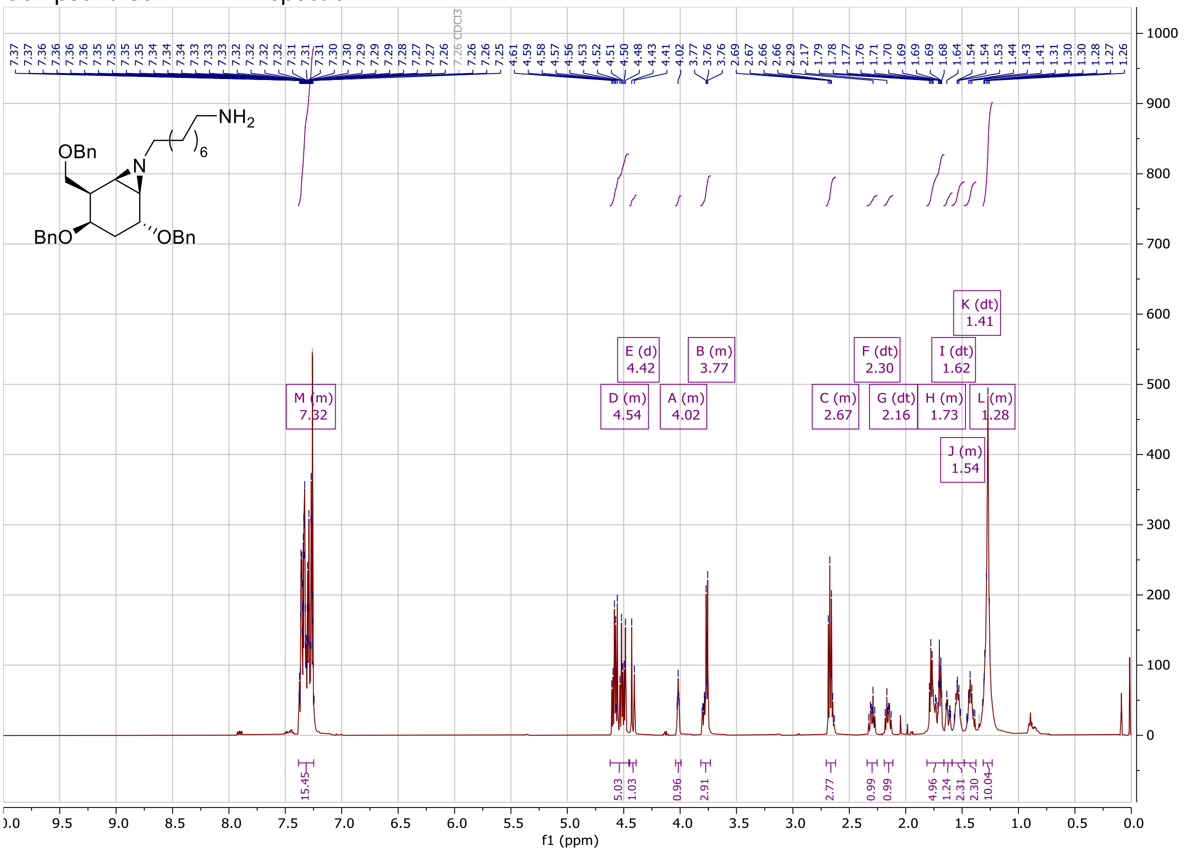

Compound **s97** ^{13}C NMR APT spectrum

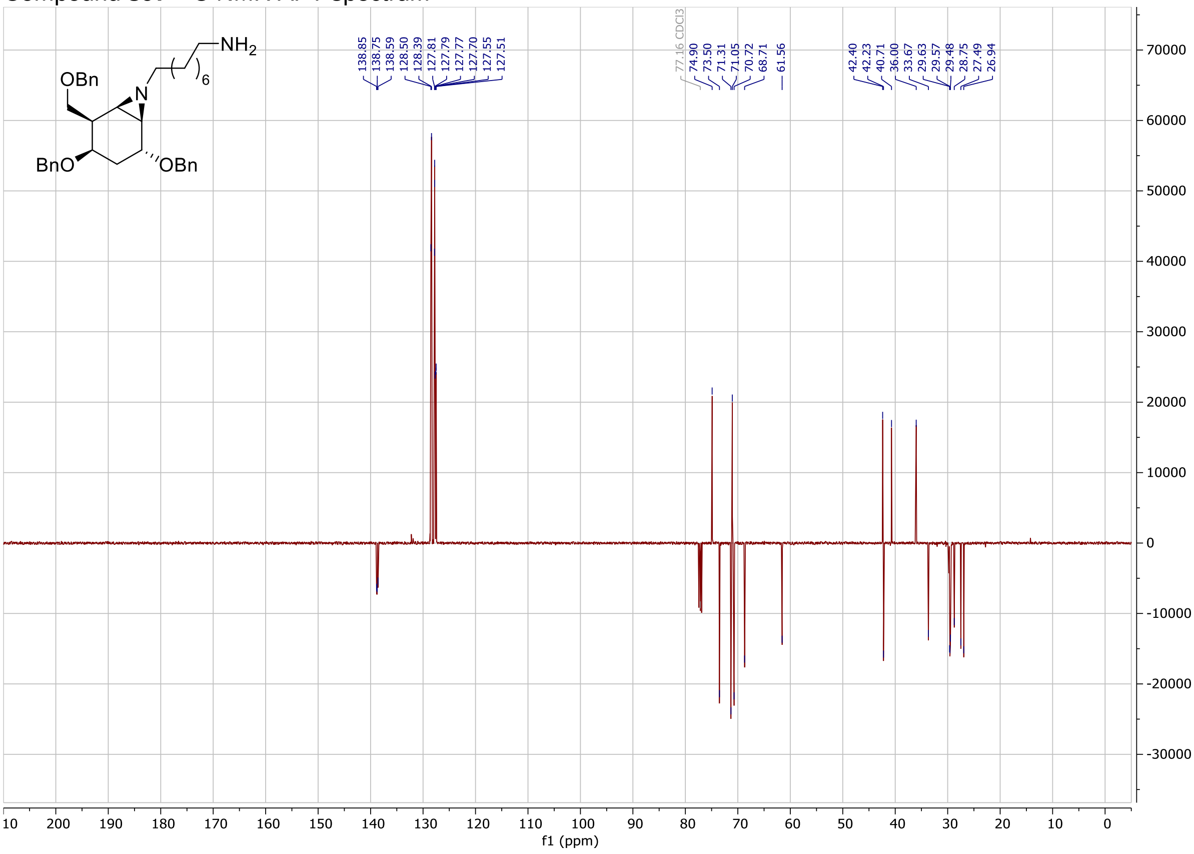

Compound **s97** ^{1}H-^{1}H COSY spectrum

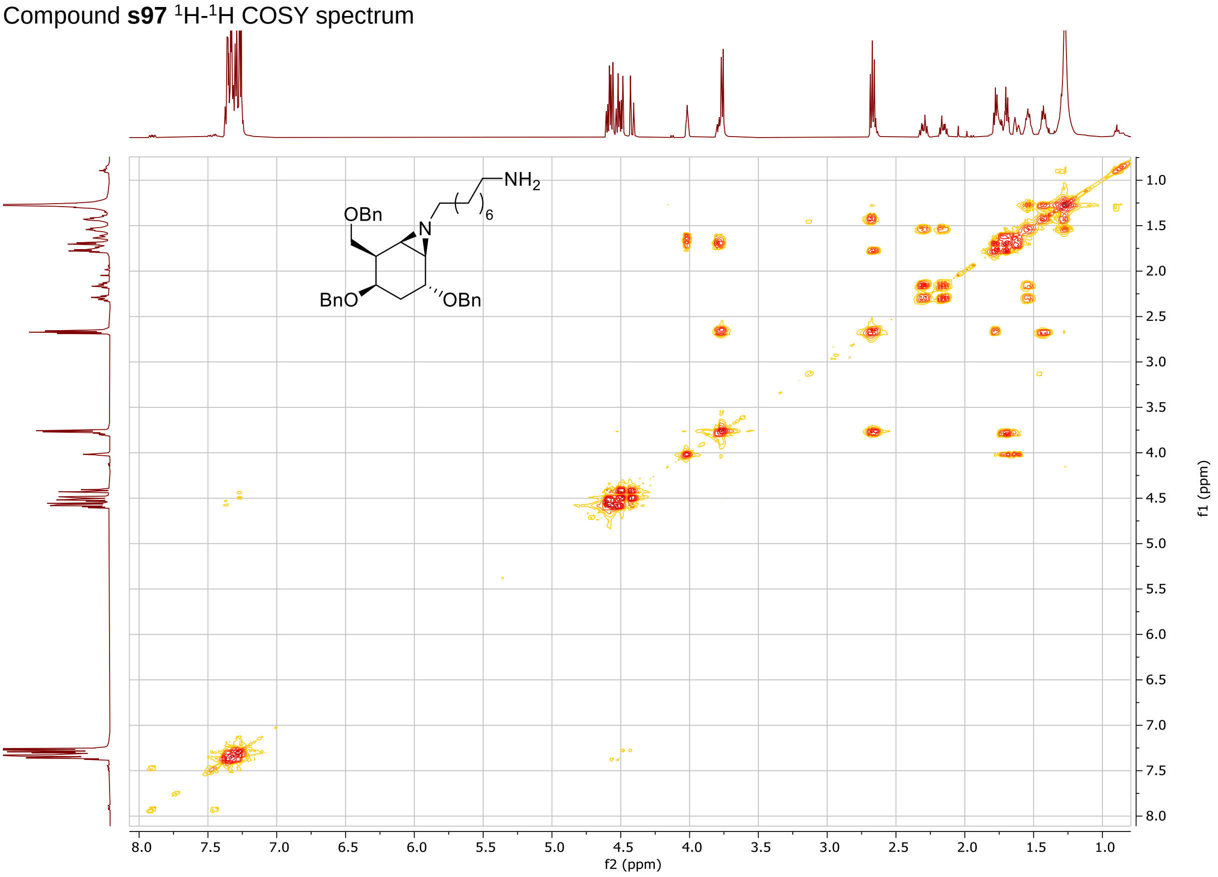

Compound **s97** ^{1}H-^{13}C HSQC spectrum

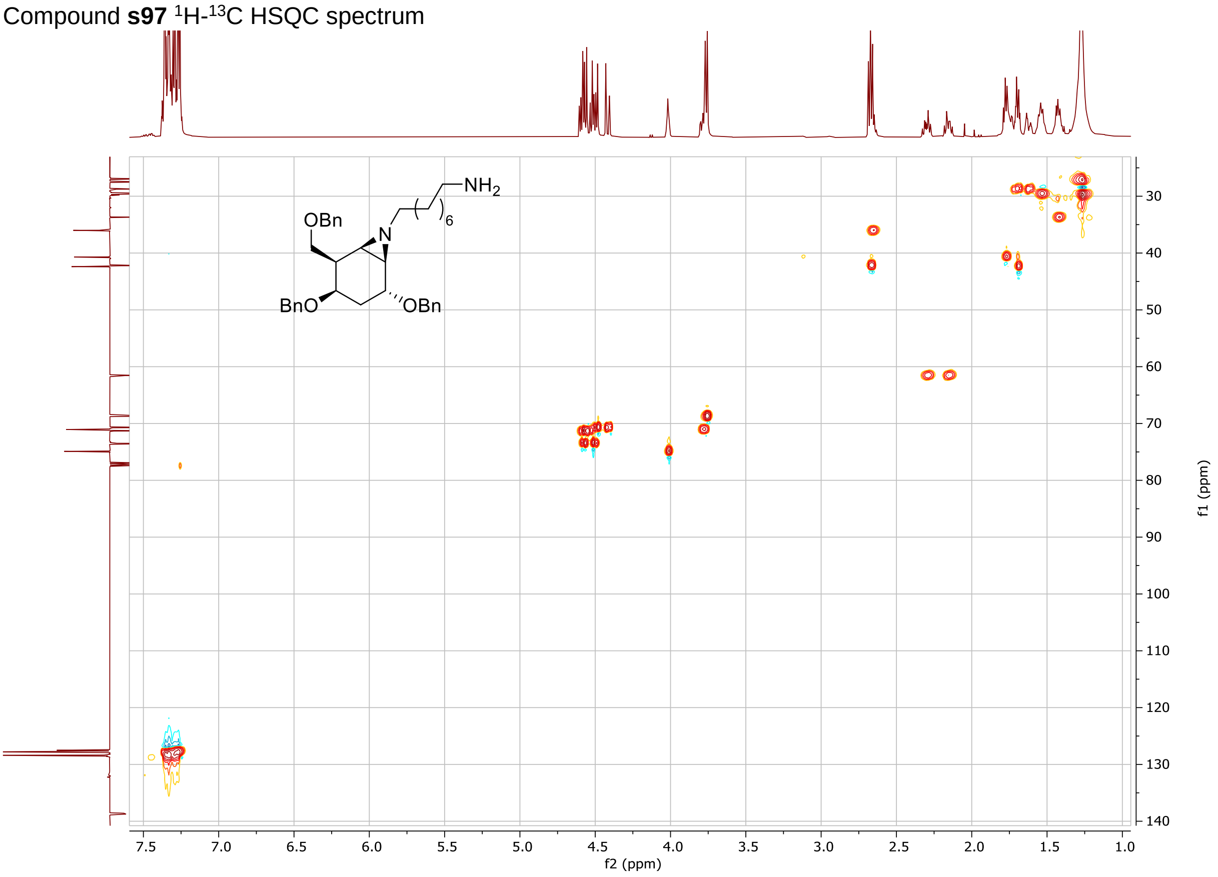

Compound **s98** ^{1}H NMR spectrum

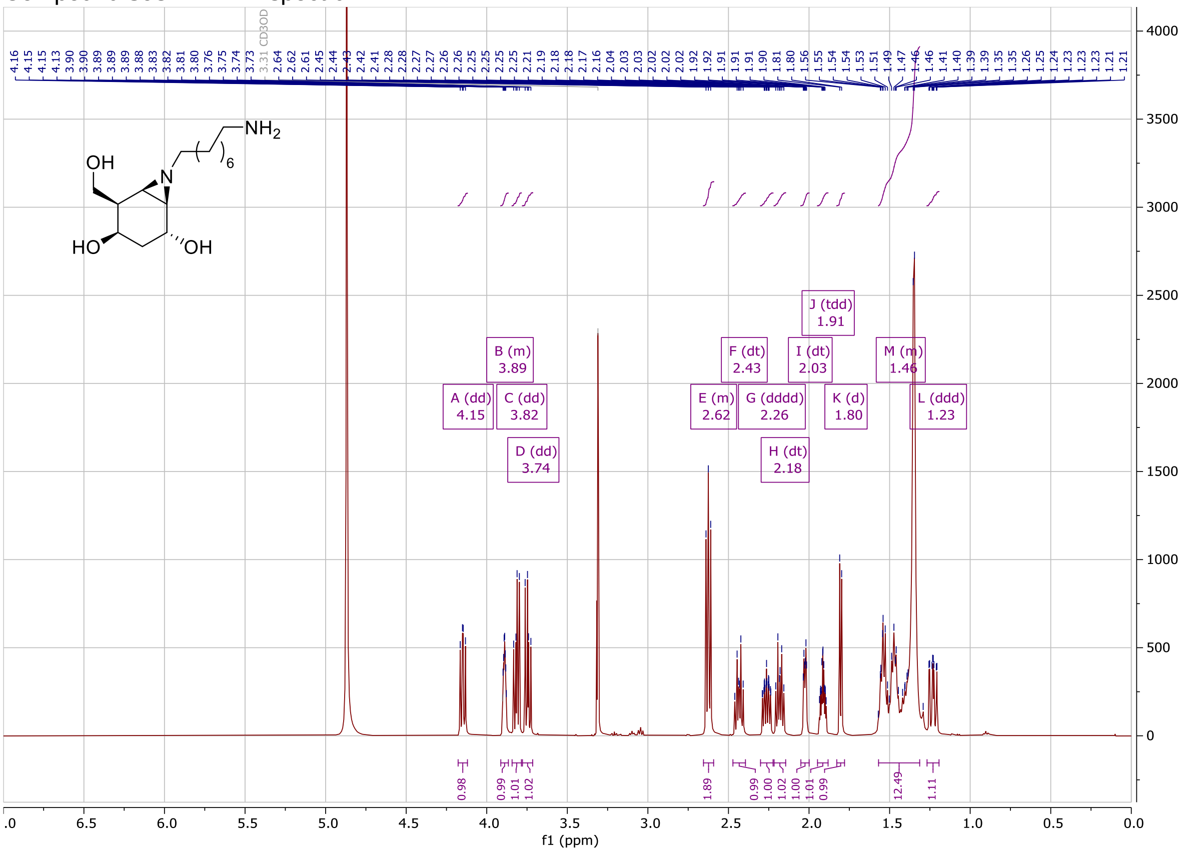

Compound **s98** ^{13}C NMR APT spectrum

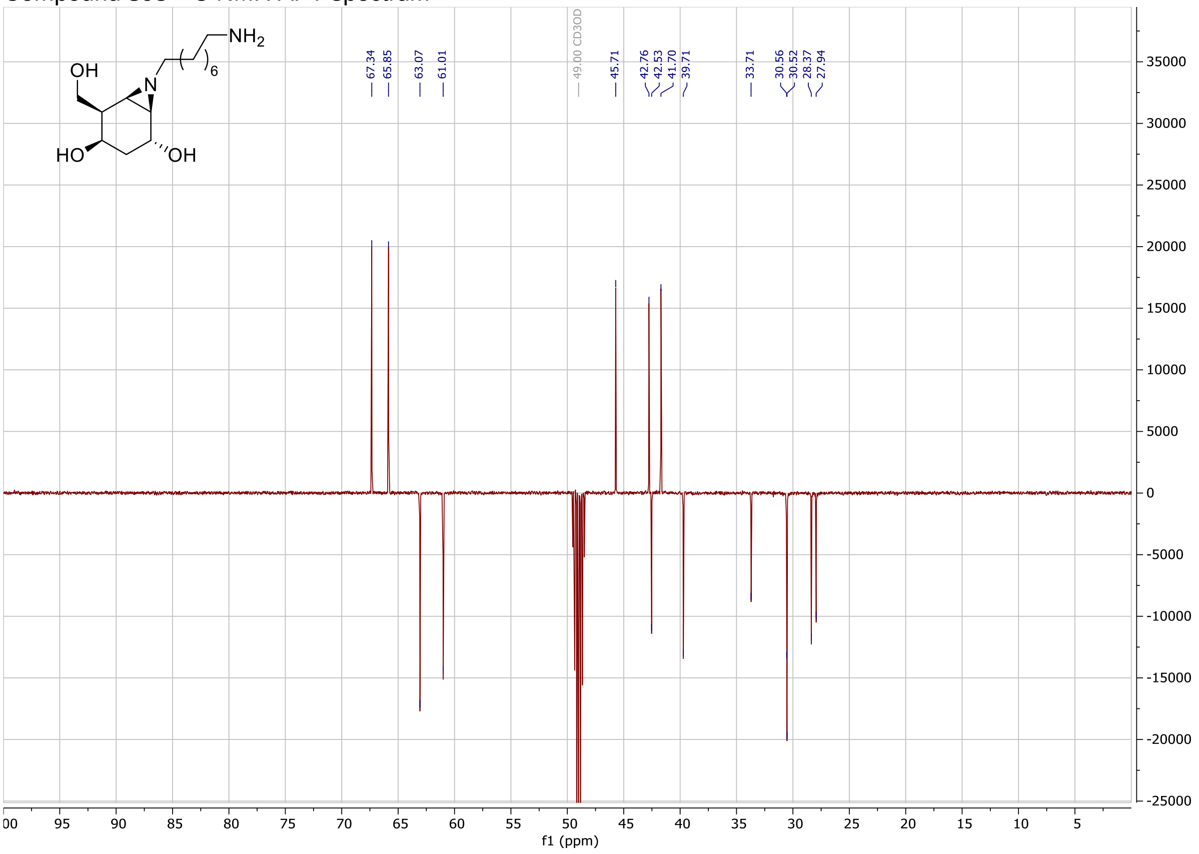

Compound **s98** ^{1}H-^{1}H COSY spectrum

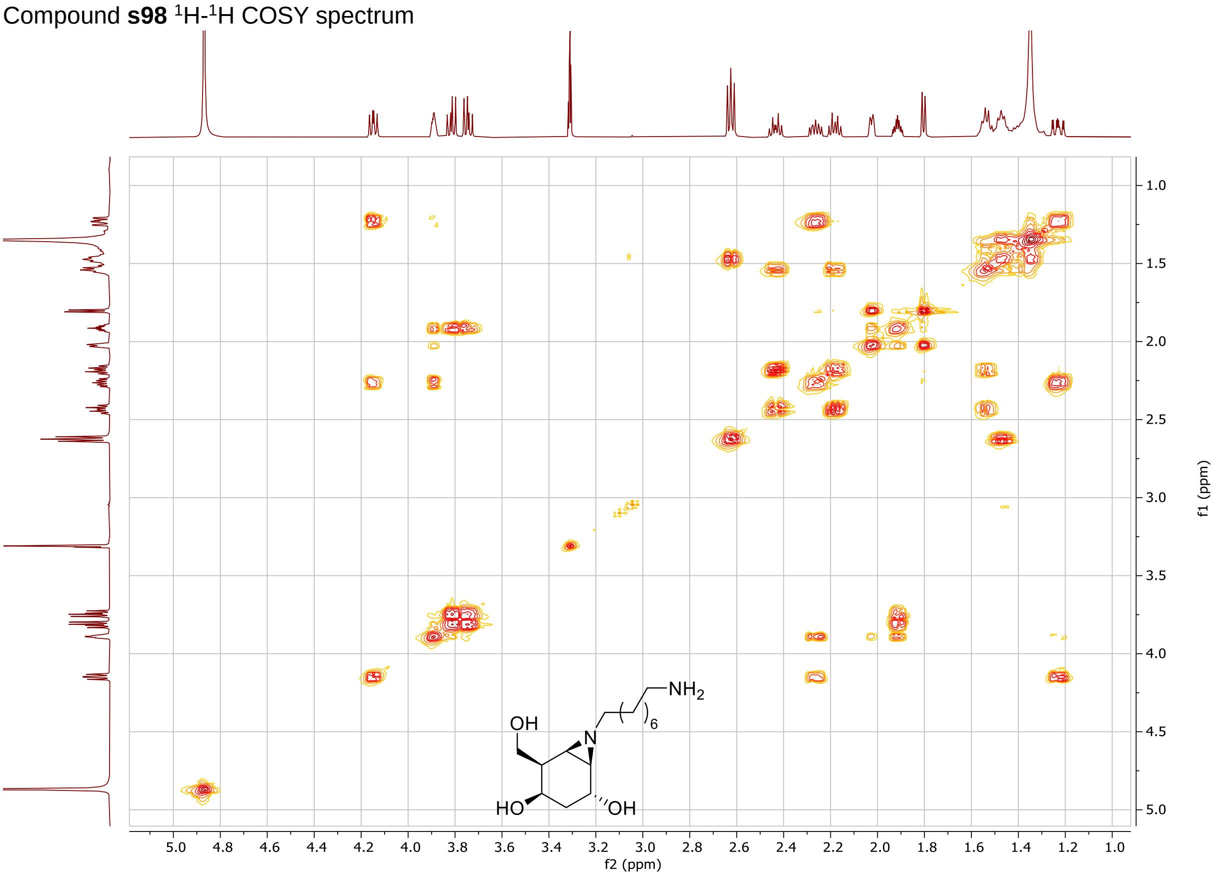

Compound **s98** ^{1}H-^{13}C HSQC spectrum

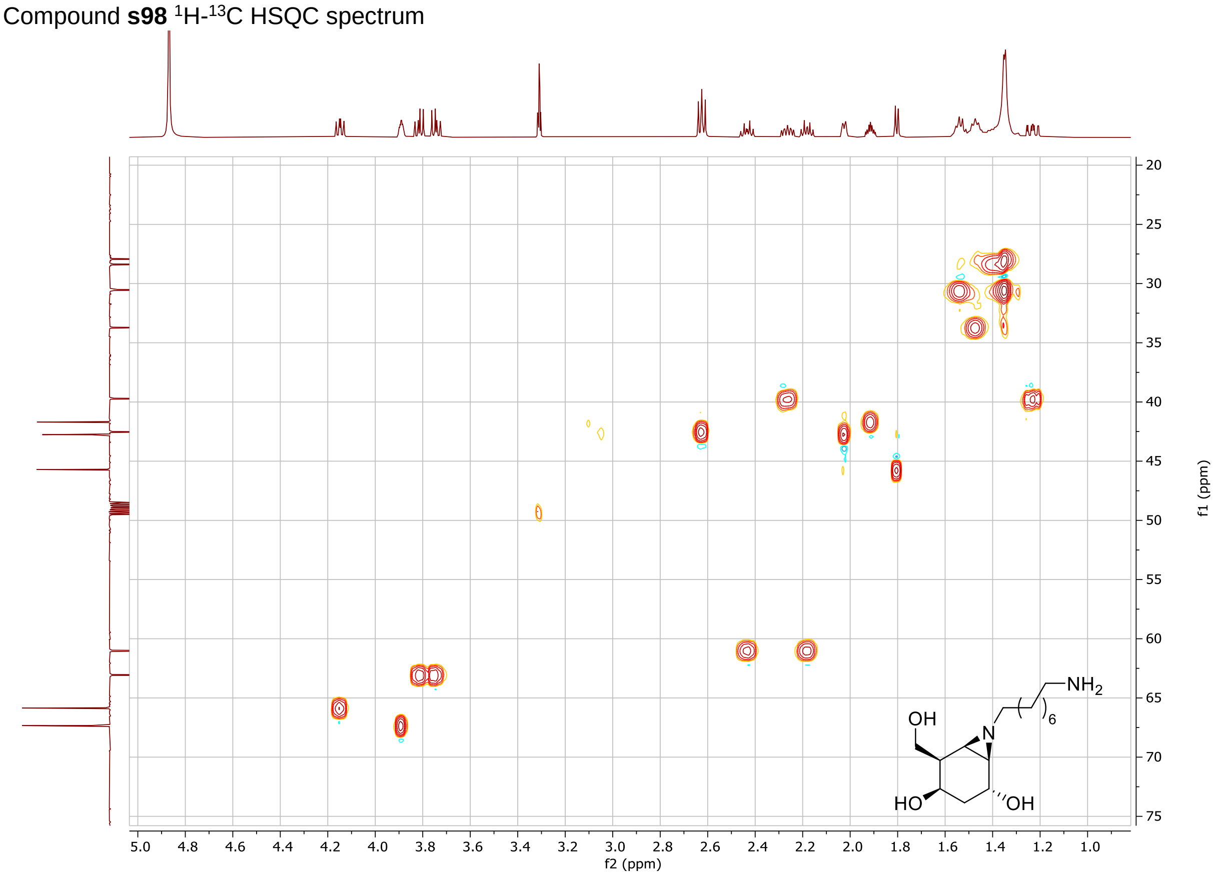

Compound **48** ^{1}H NMR spectrum

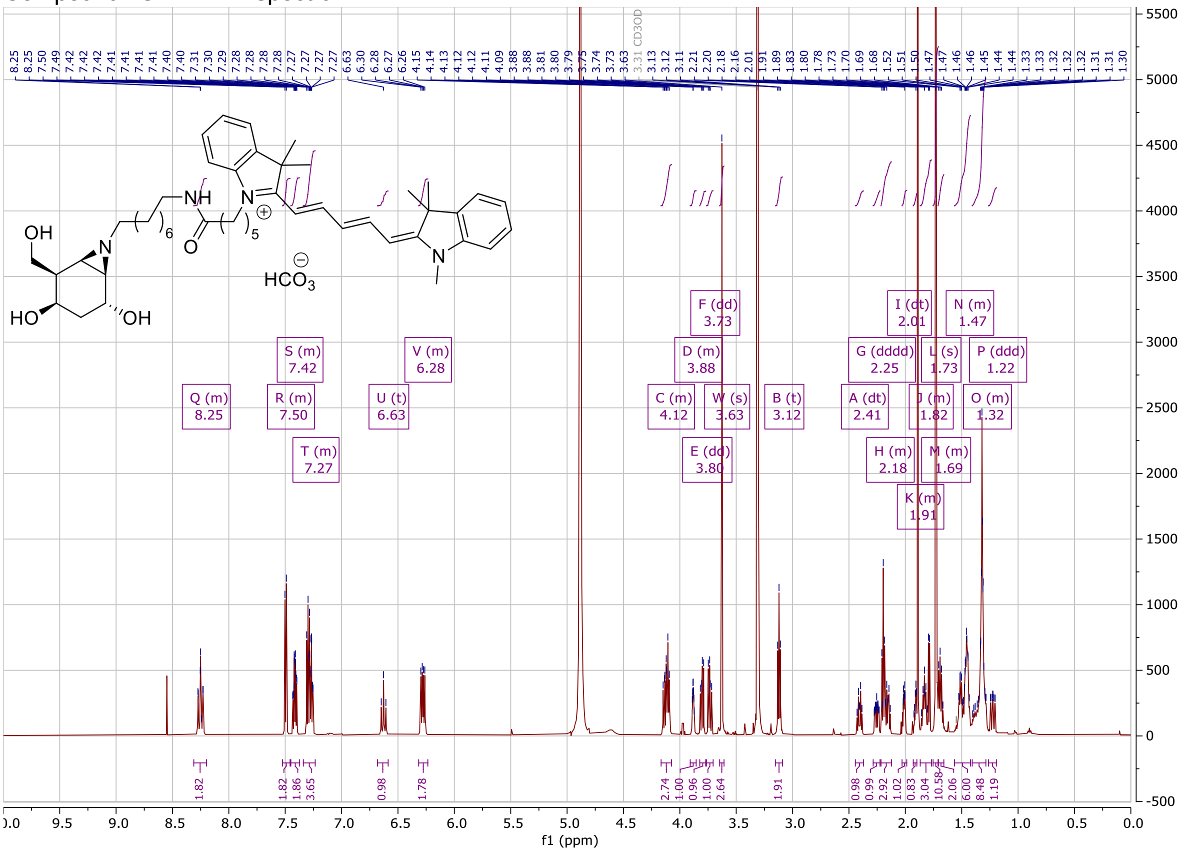

Compound **48** ^{13}C NMR APT spectrum

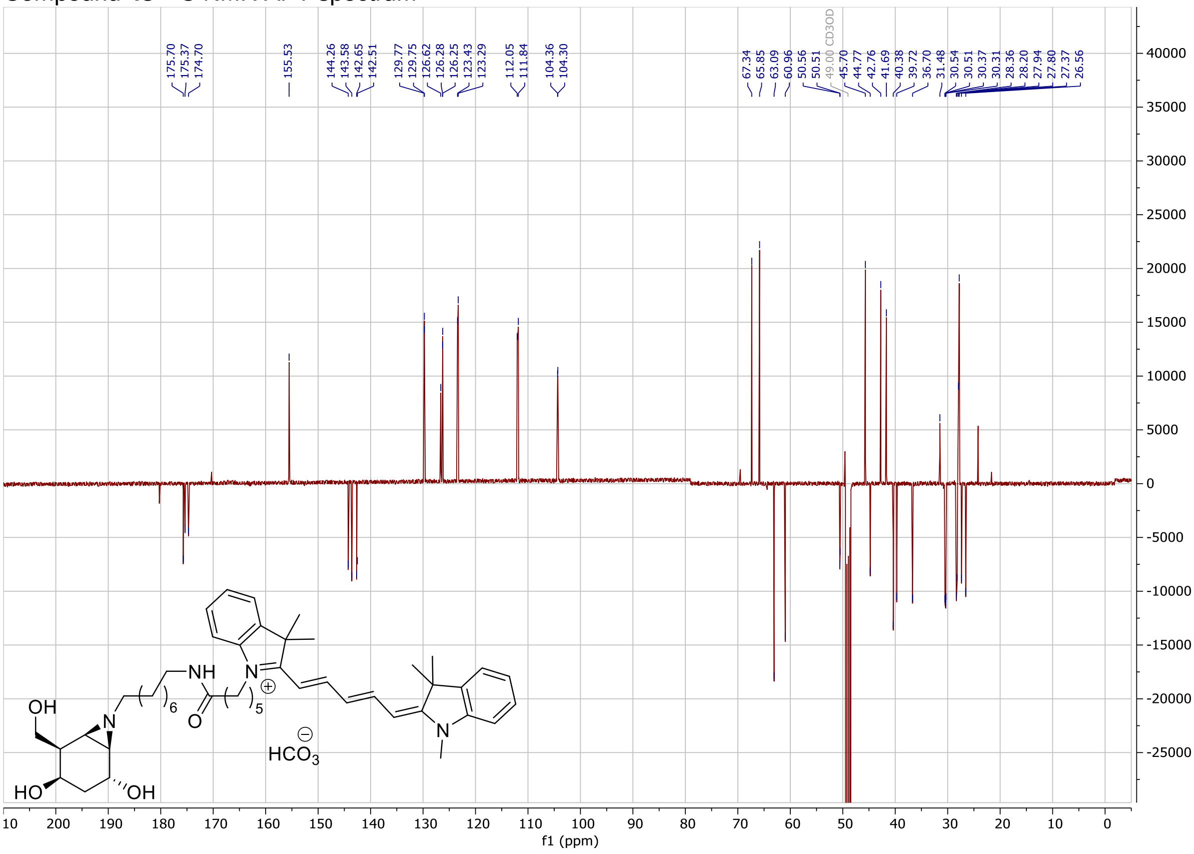

Compound **48** ^{1}H-^{1}H COSY spectrum

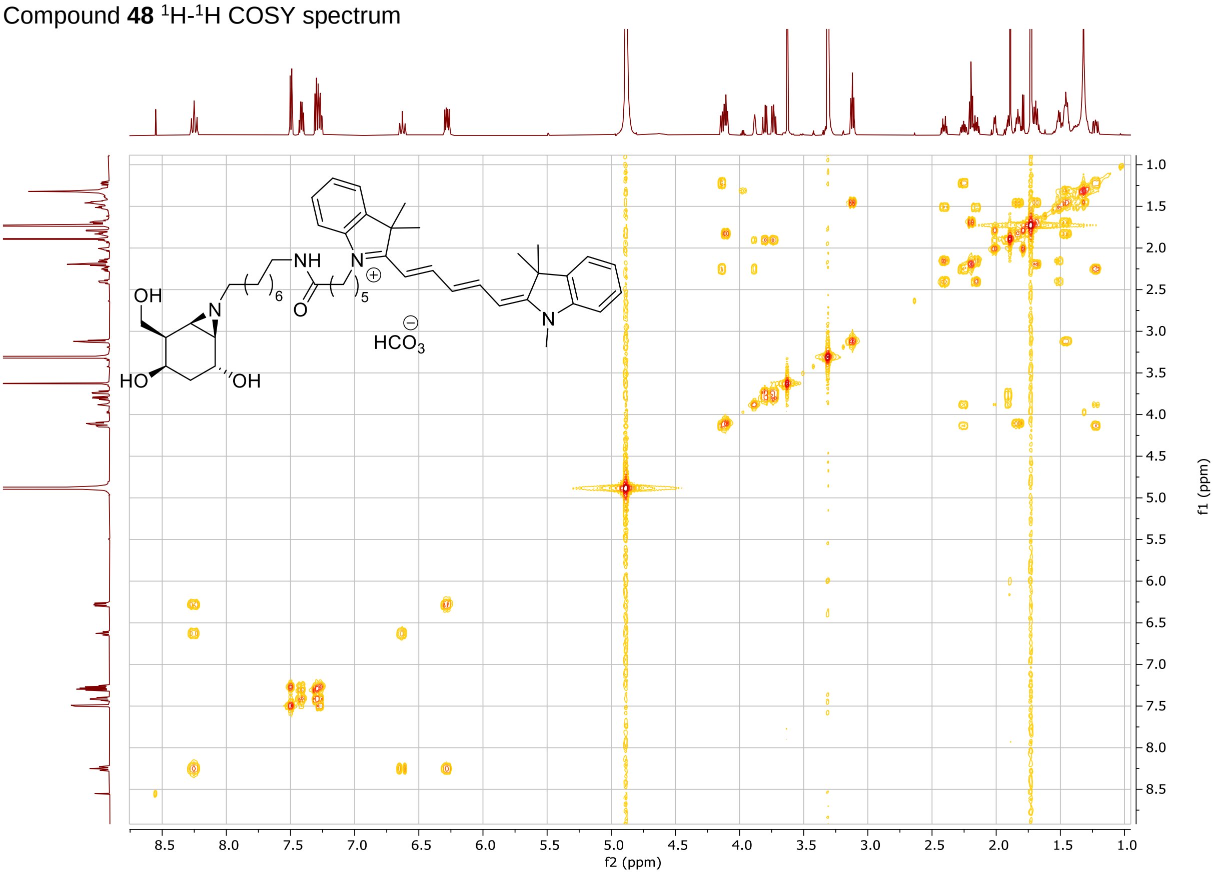

Compound **48** ^{1}H-^{13}C HSQC spectrum

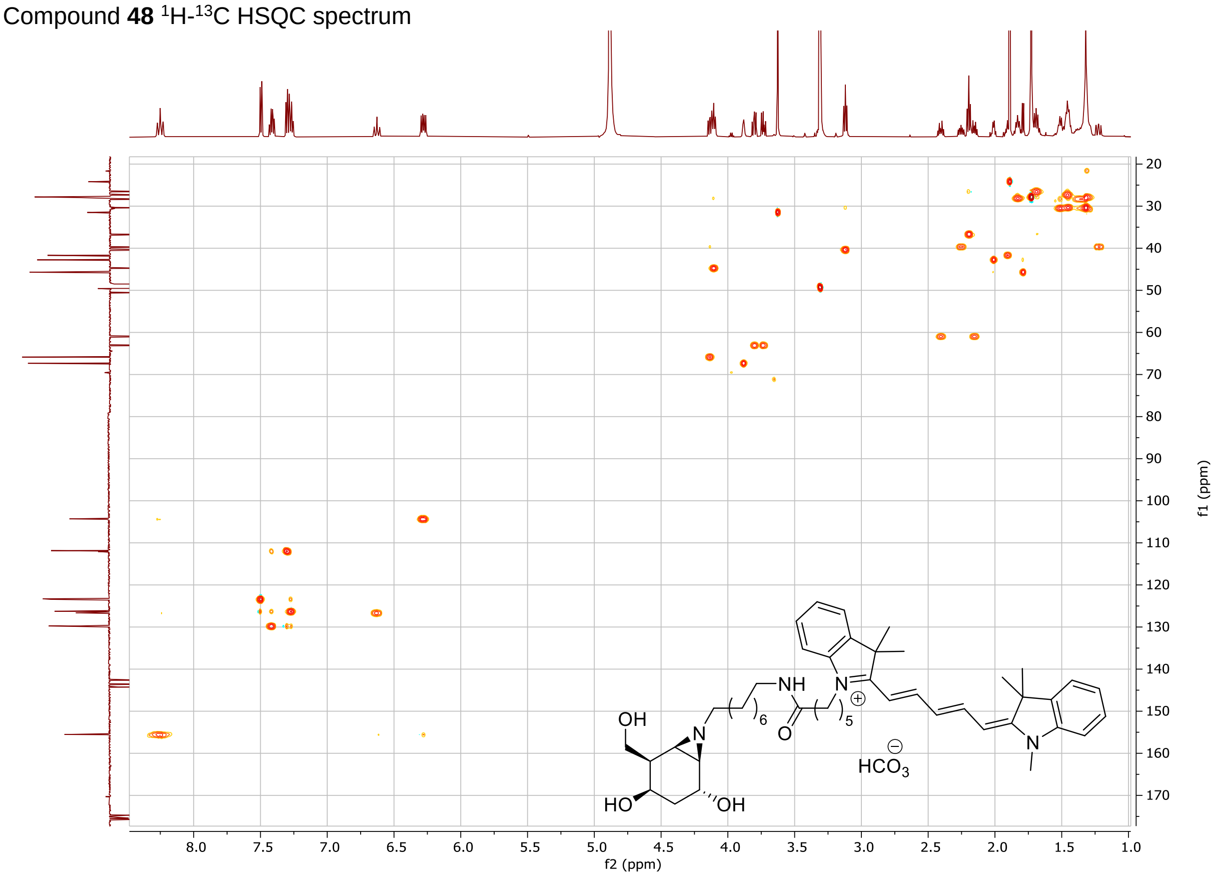

Compound **55** ^{1}H NMR spectrum

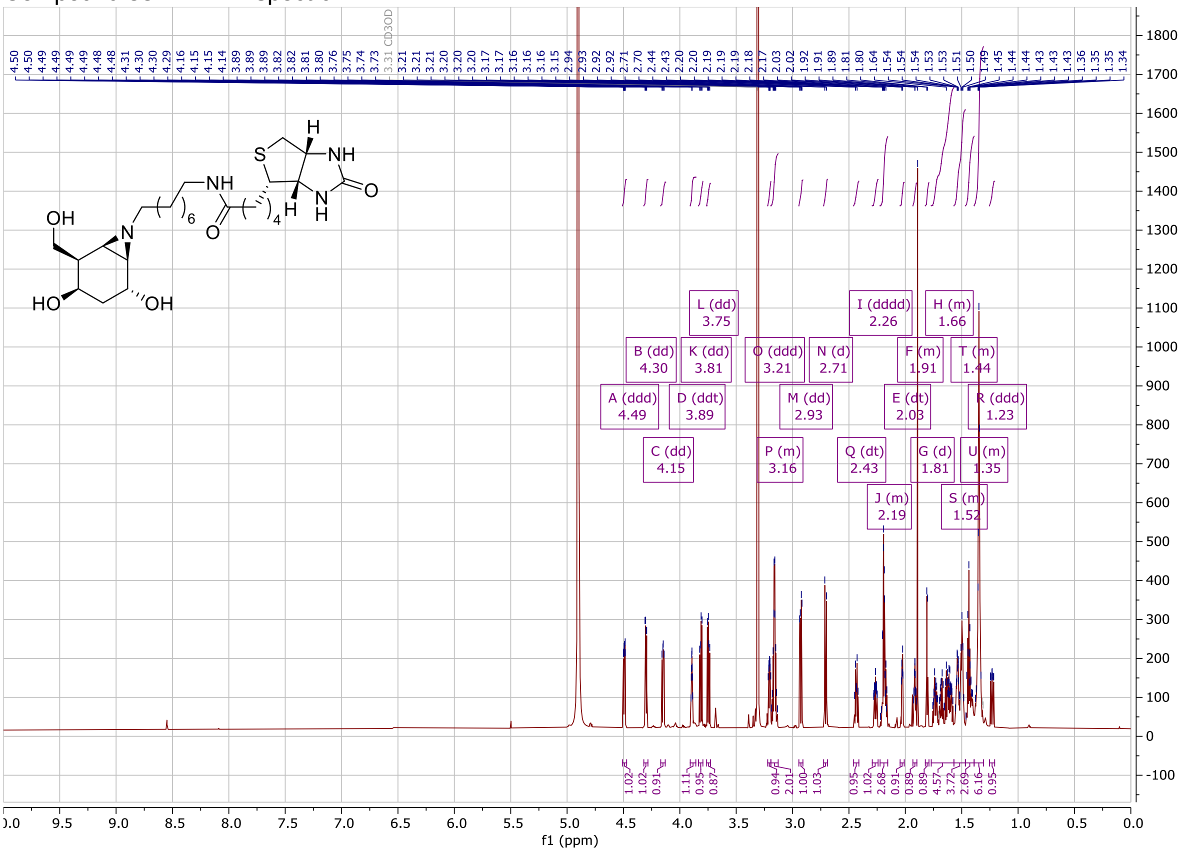

Compound **55** ^{13}C NMR APT spectrum

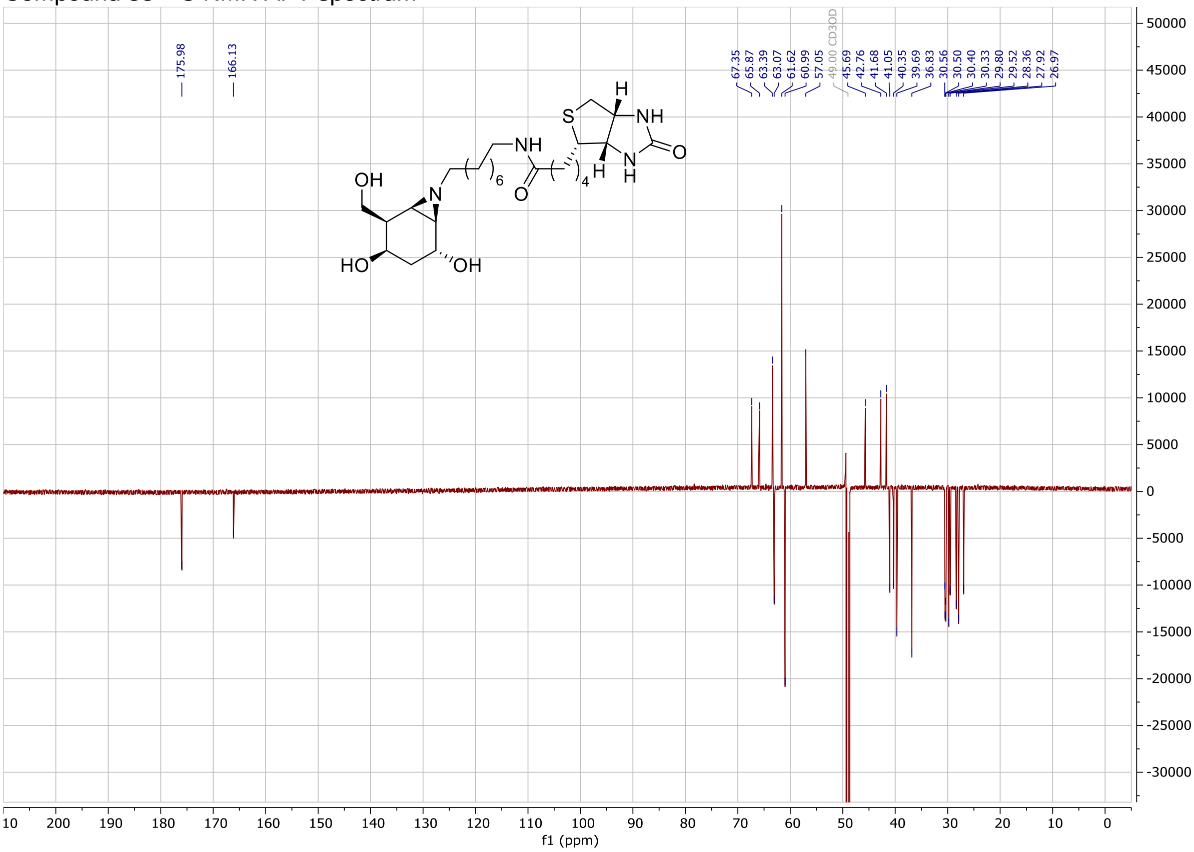

Compound **55** ^{1}H-^{1}H COSY spectrum

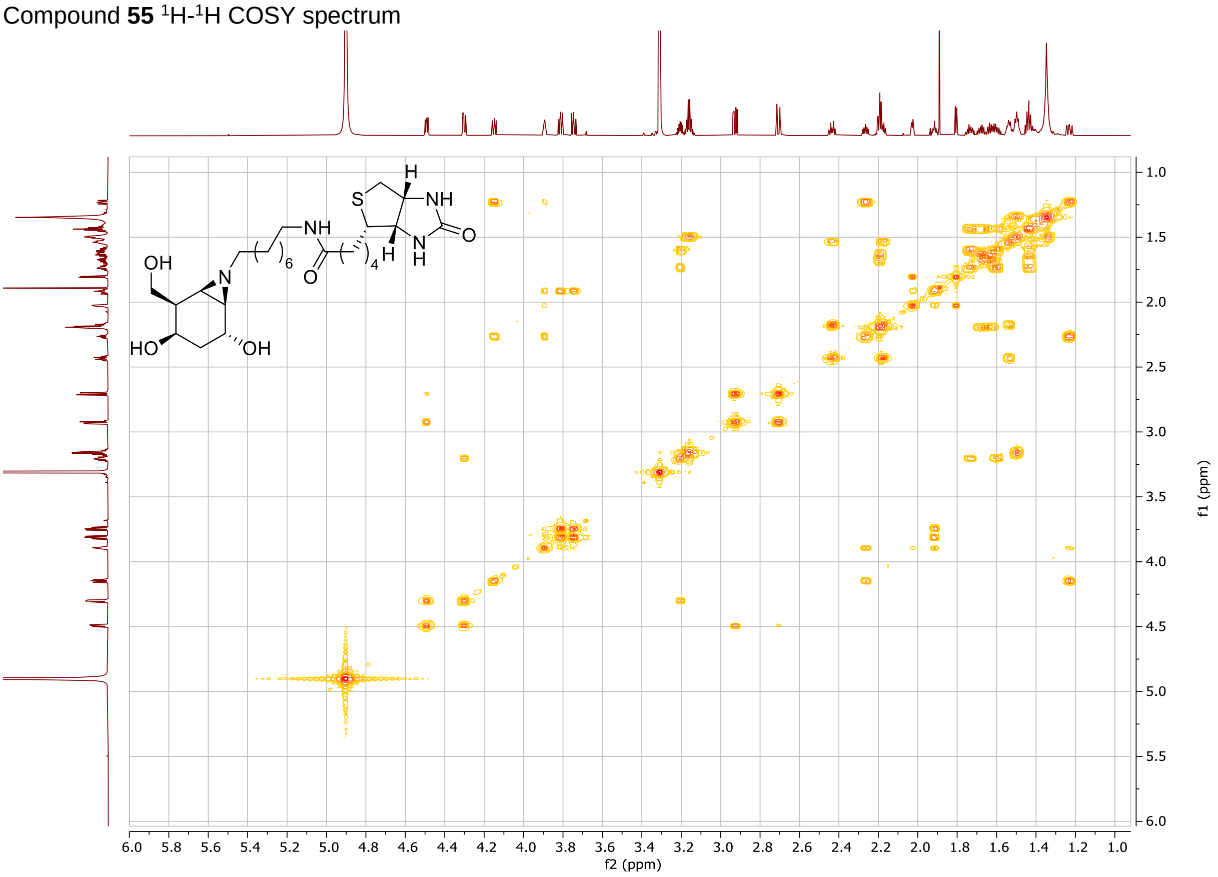

Compound **55** ^{1}H-^{13}C HSQC spectrum

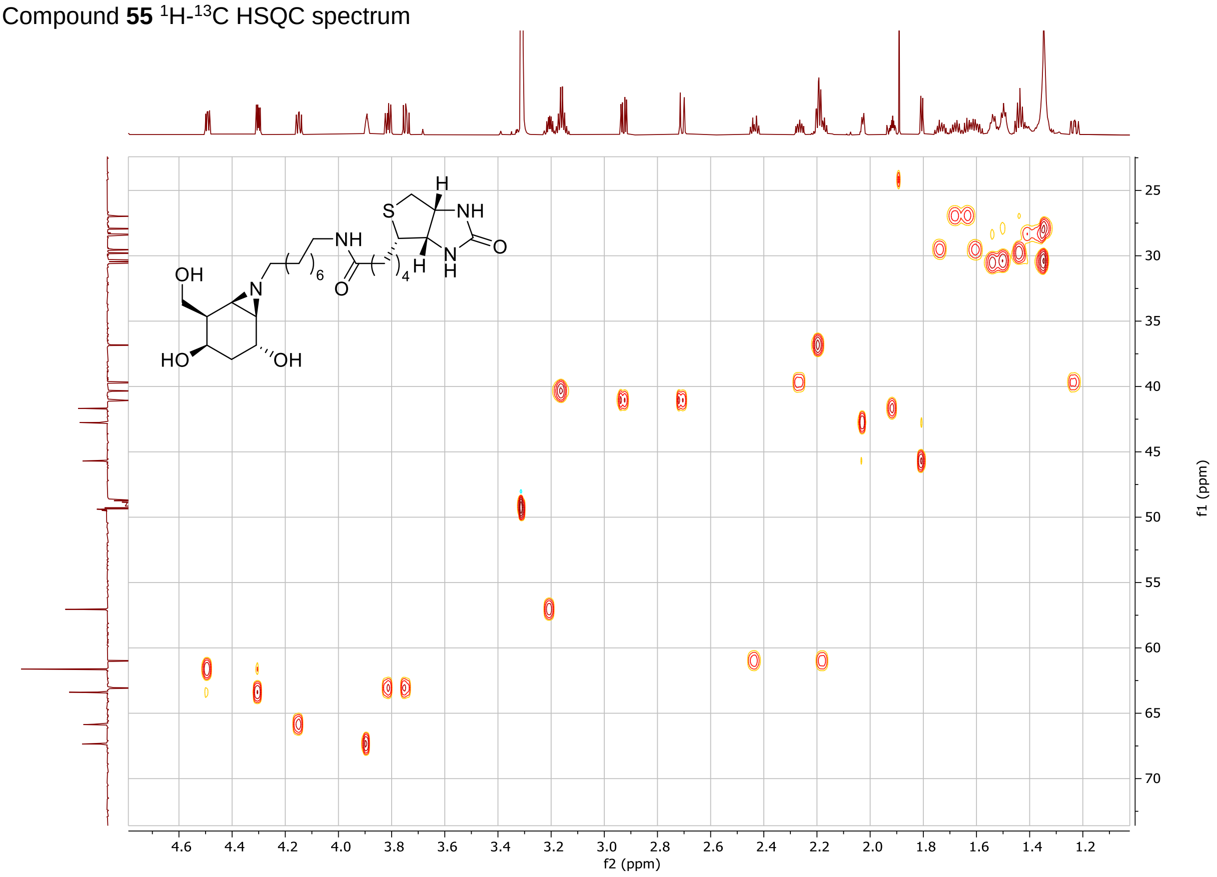

Compound **s99** ^{1}H NMR spectrum

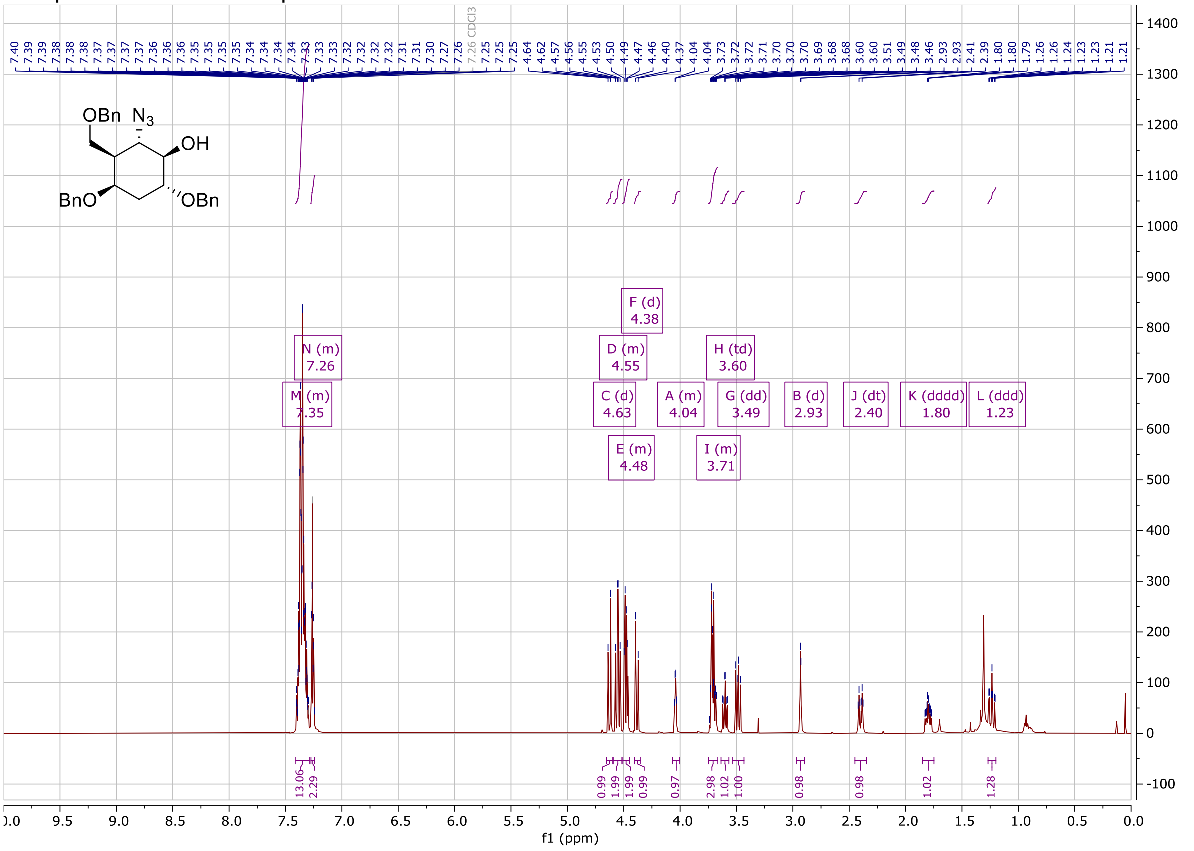

Compound **s99** ^{13}C NMR APT spectrum

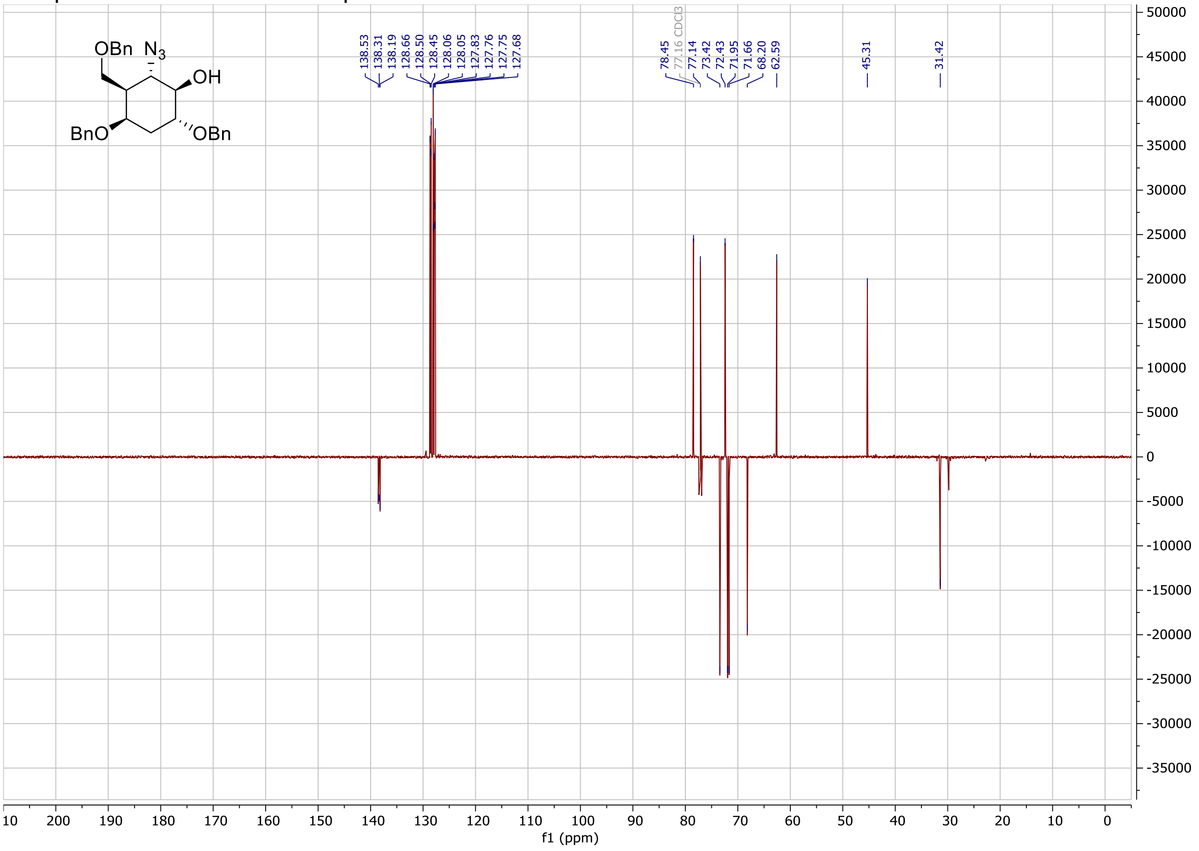

Compound **s99** ^{1}H-^{1}H COSY spectrum

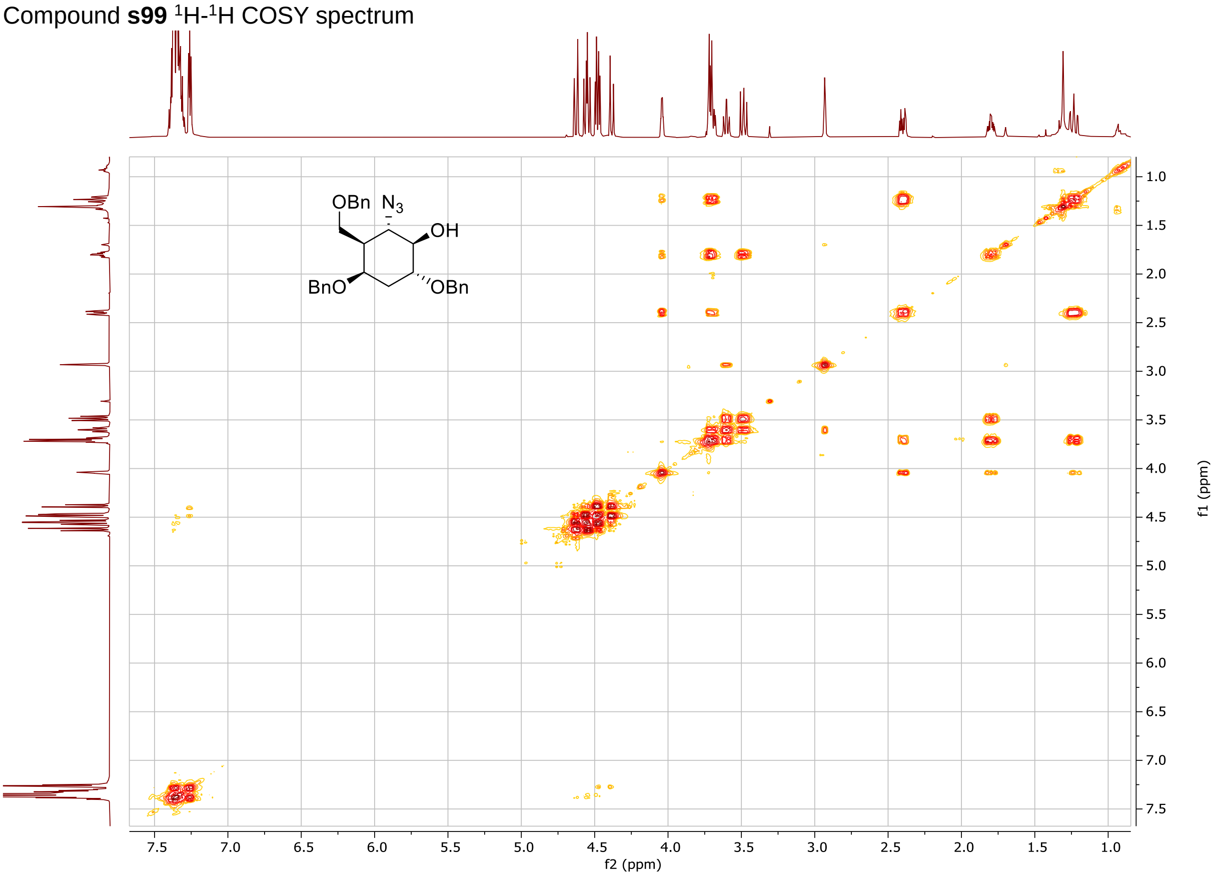

Compound **s99** ^{1}H-^{13}C HSQC spectrum

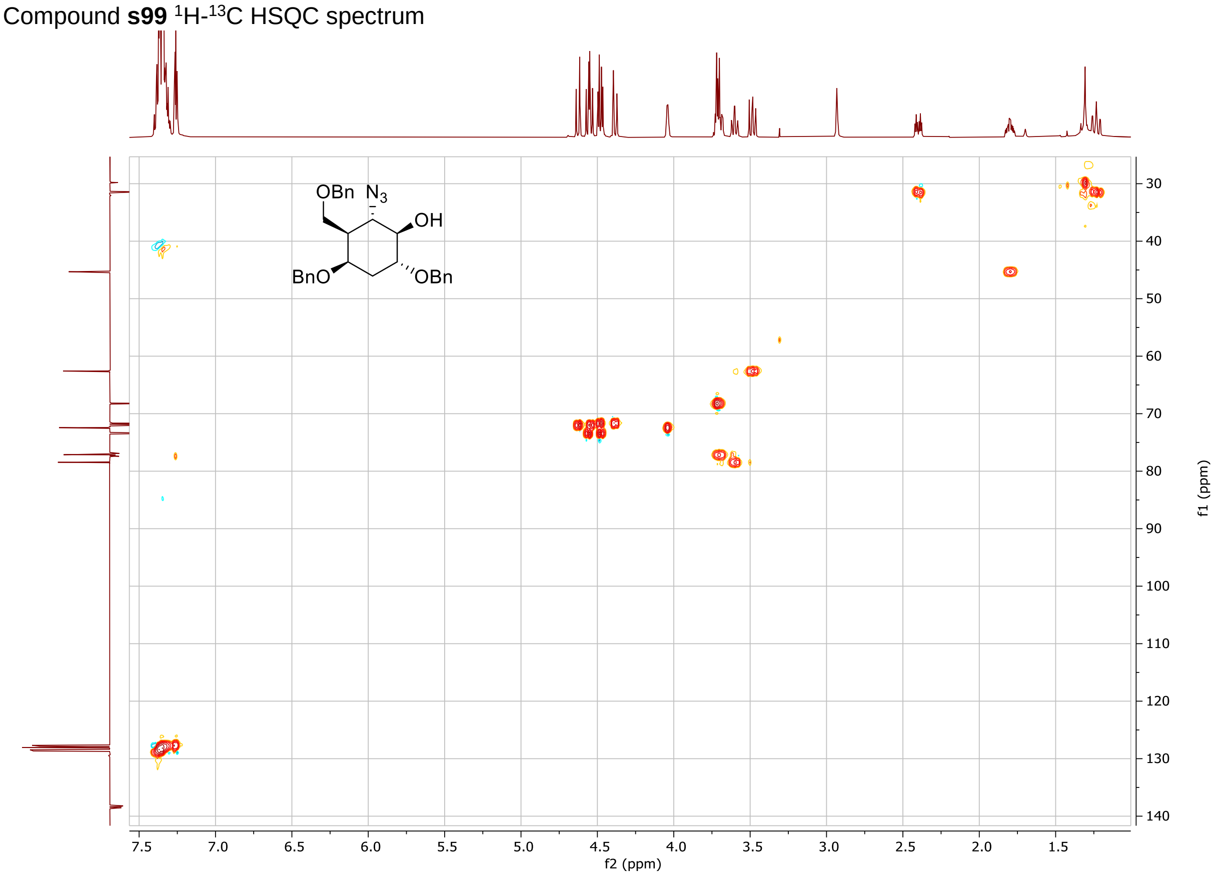

Compound **s99** ^1H-^1H NOESY spectrum

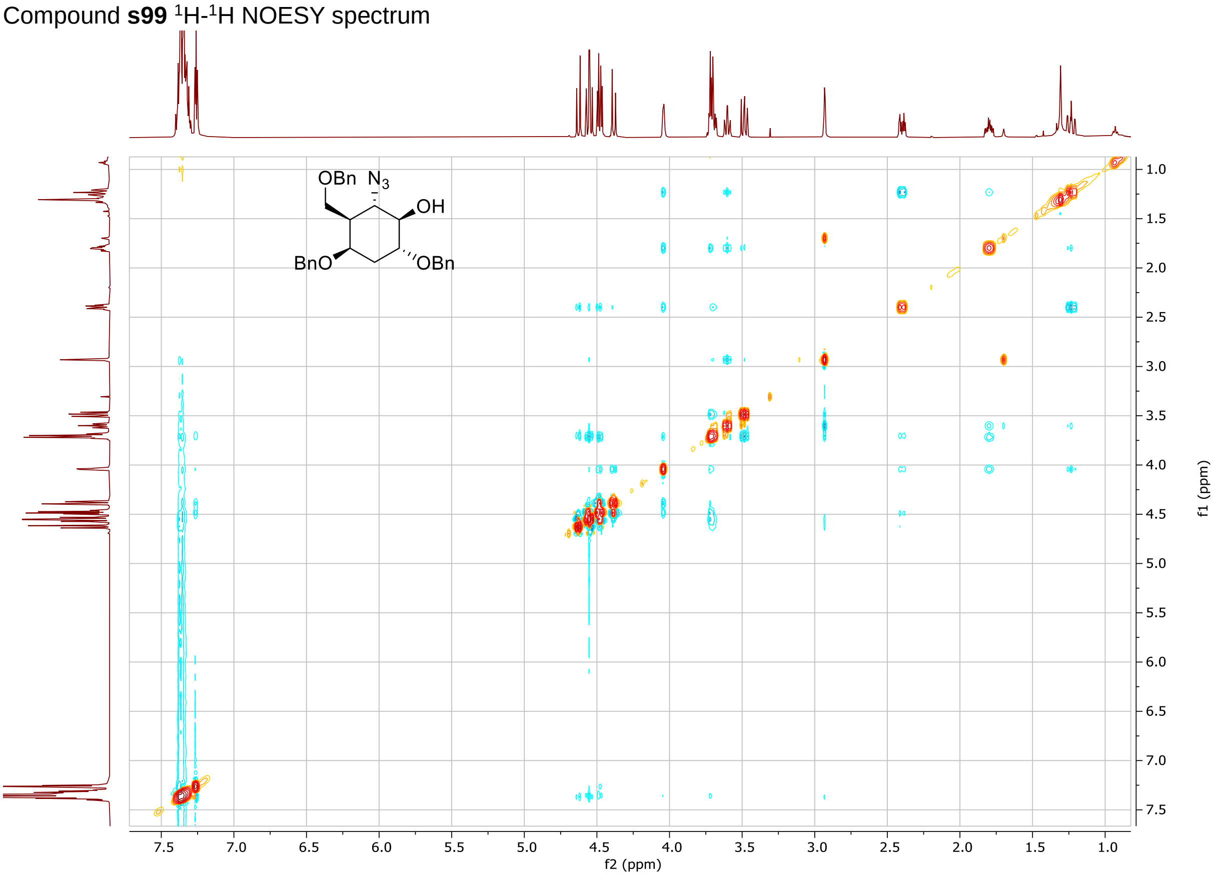

Compound **s99** ^1H-^{13}C HMBC spectrum

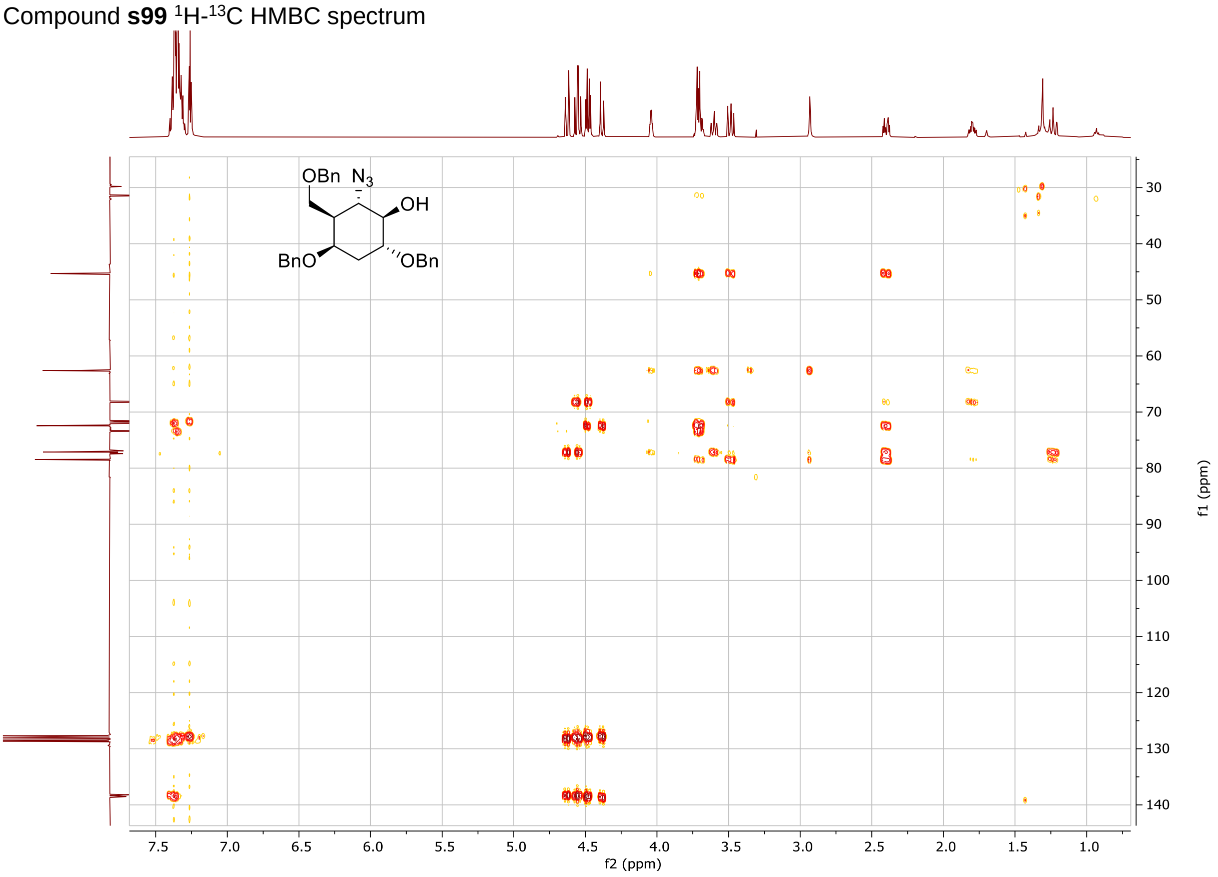

Compound **s100** ^{1}H NMR spectrum

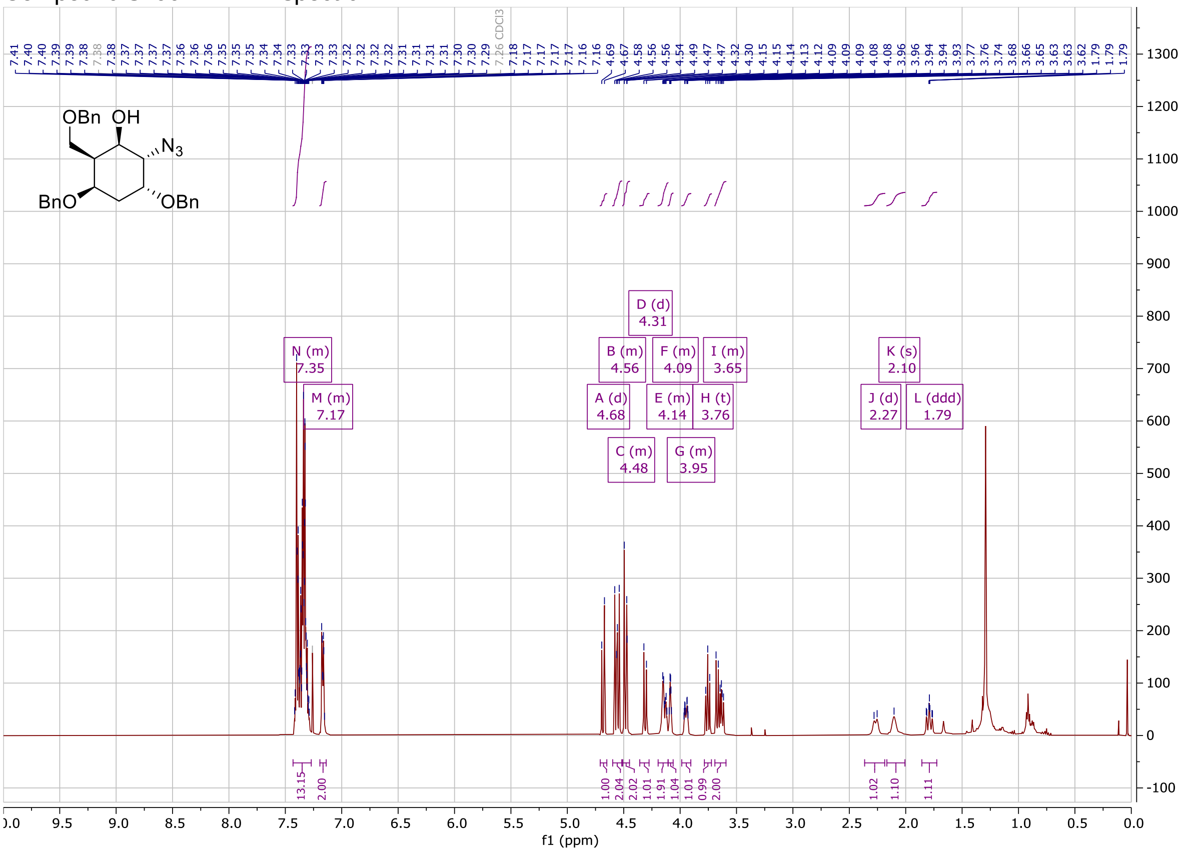

Compound **s100** ^{13}C NMR APT spectrum

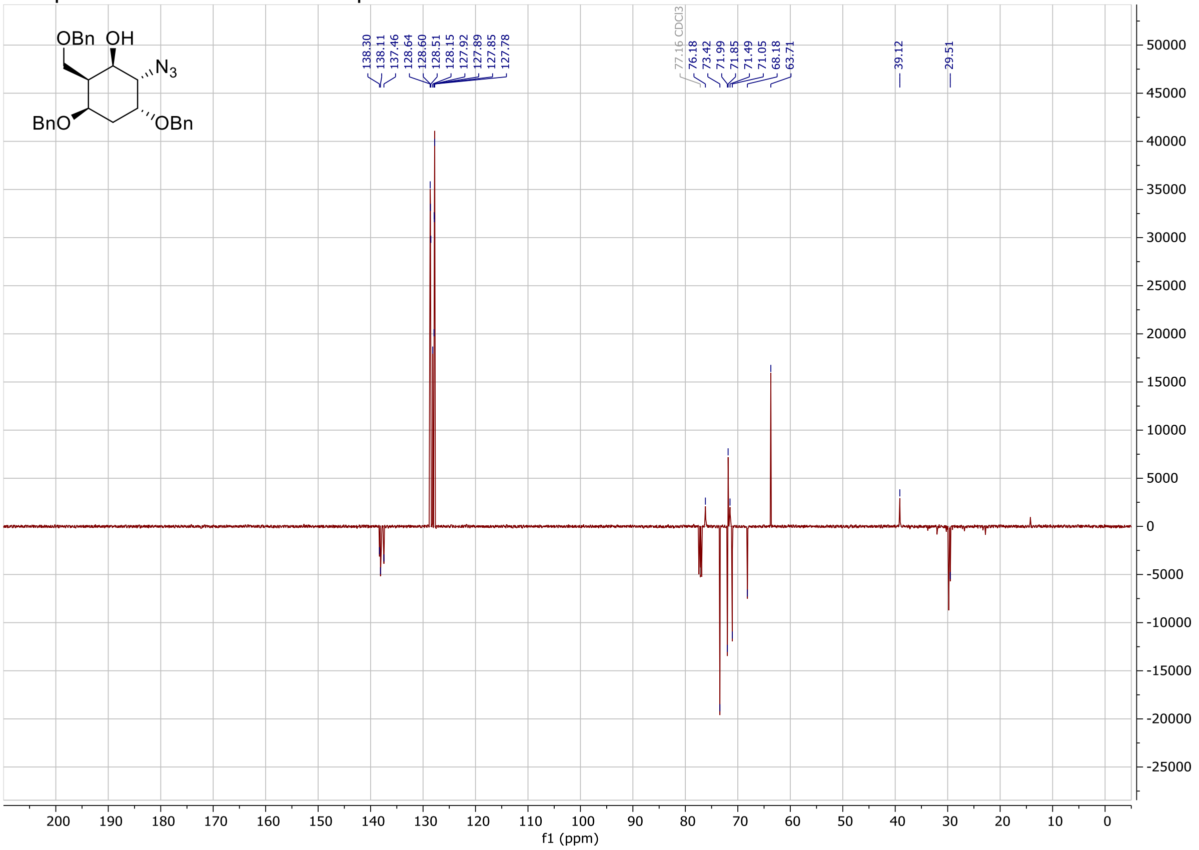

Compound **s100** ^{1}H-^{1}H COSY spectrum

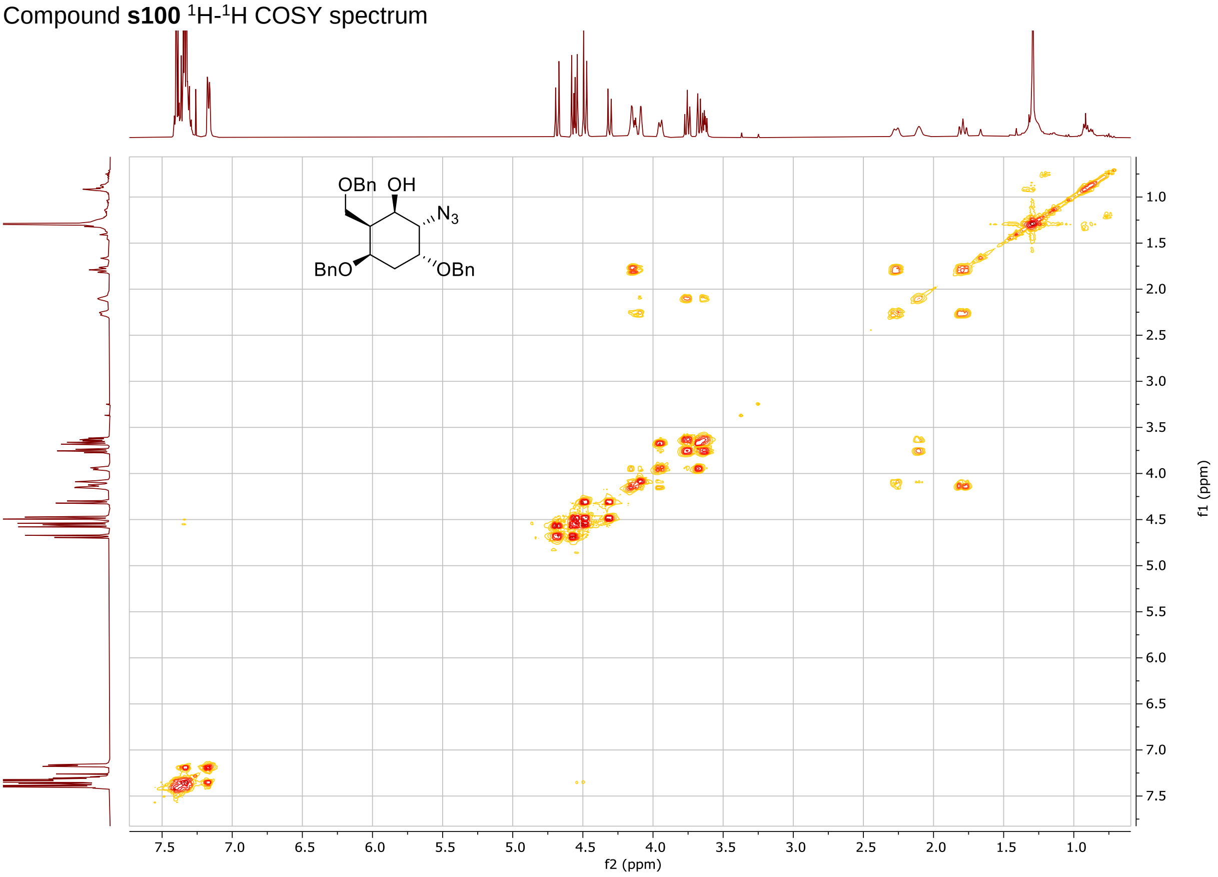

Compound **s100** ^{1}H-^{13}C HSQC spectrum

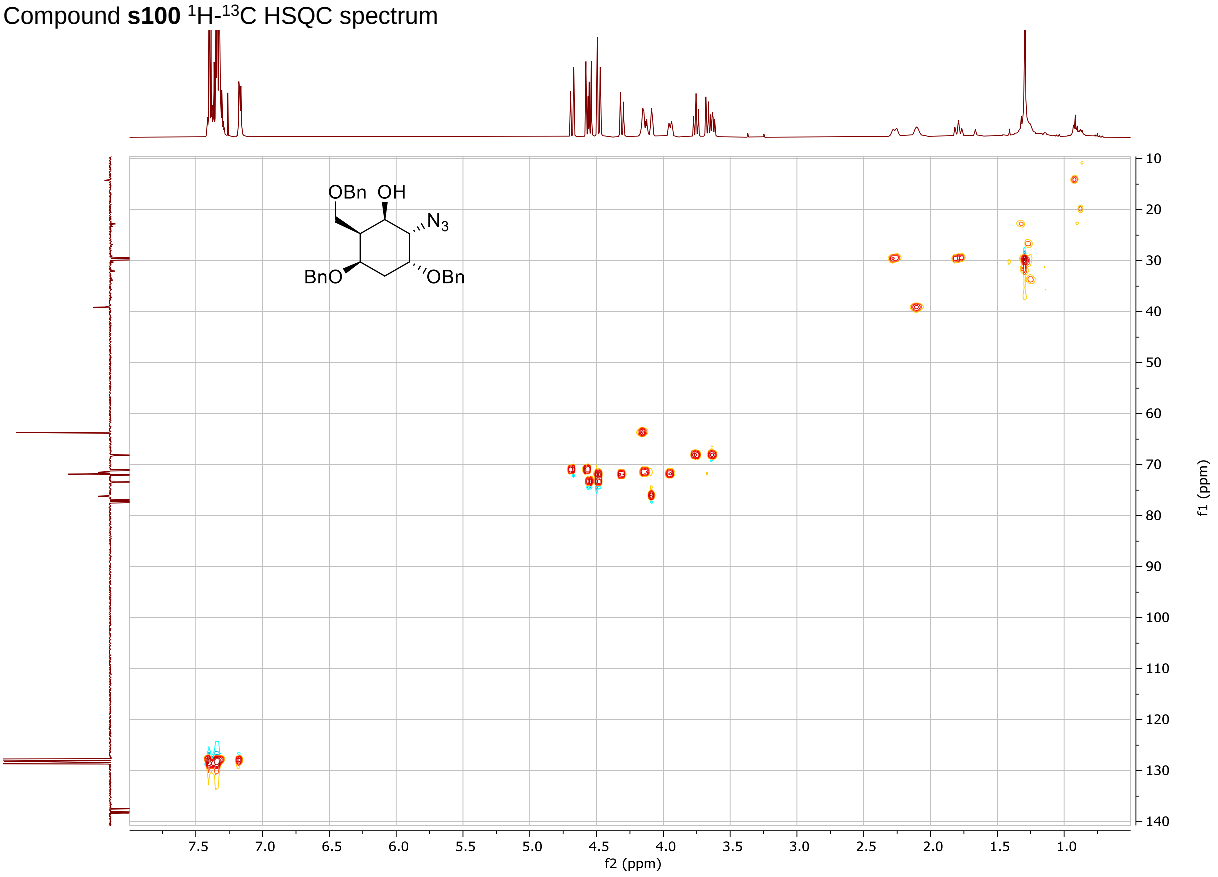

Compound **s100** ^{1}H-^{1}H NOESY spectrum

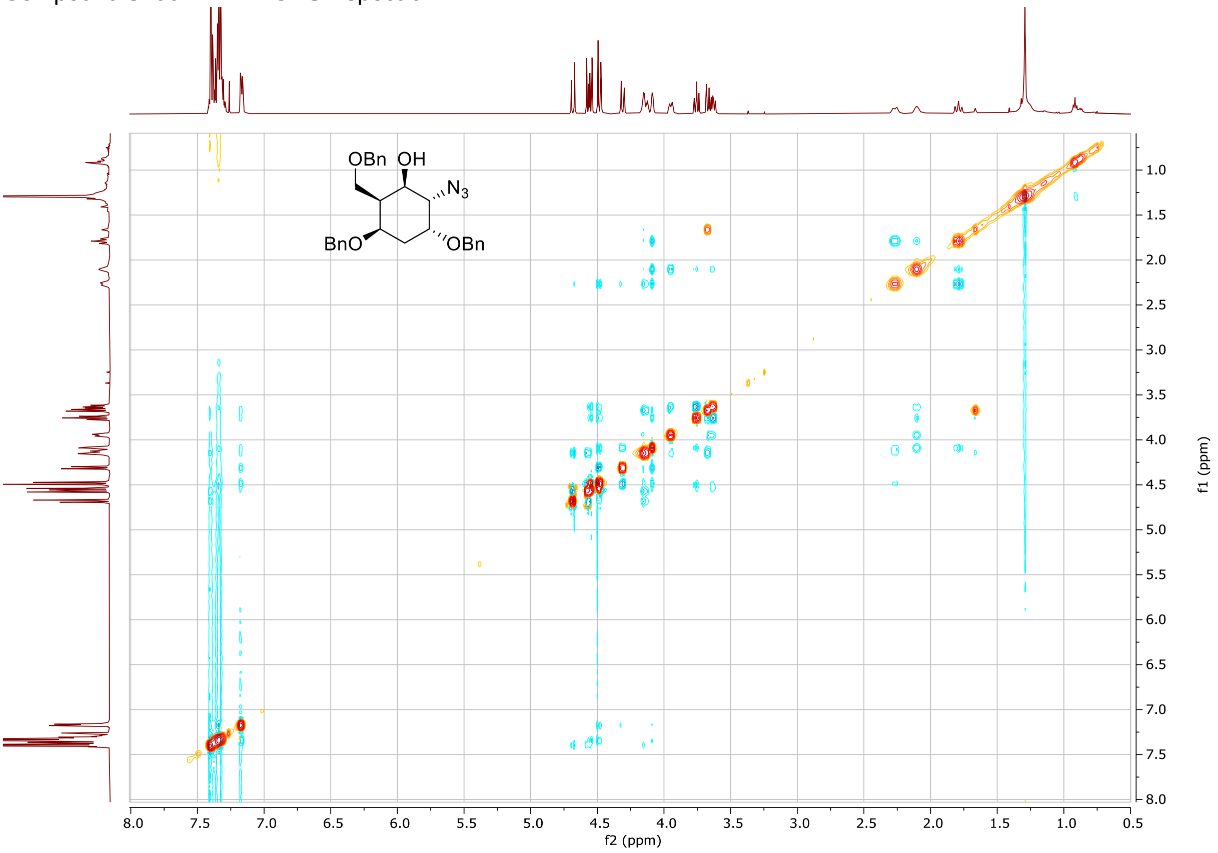

Compound **s100** ^{1}H-^{13}C HMBC spectrum

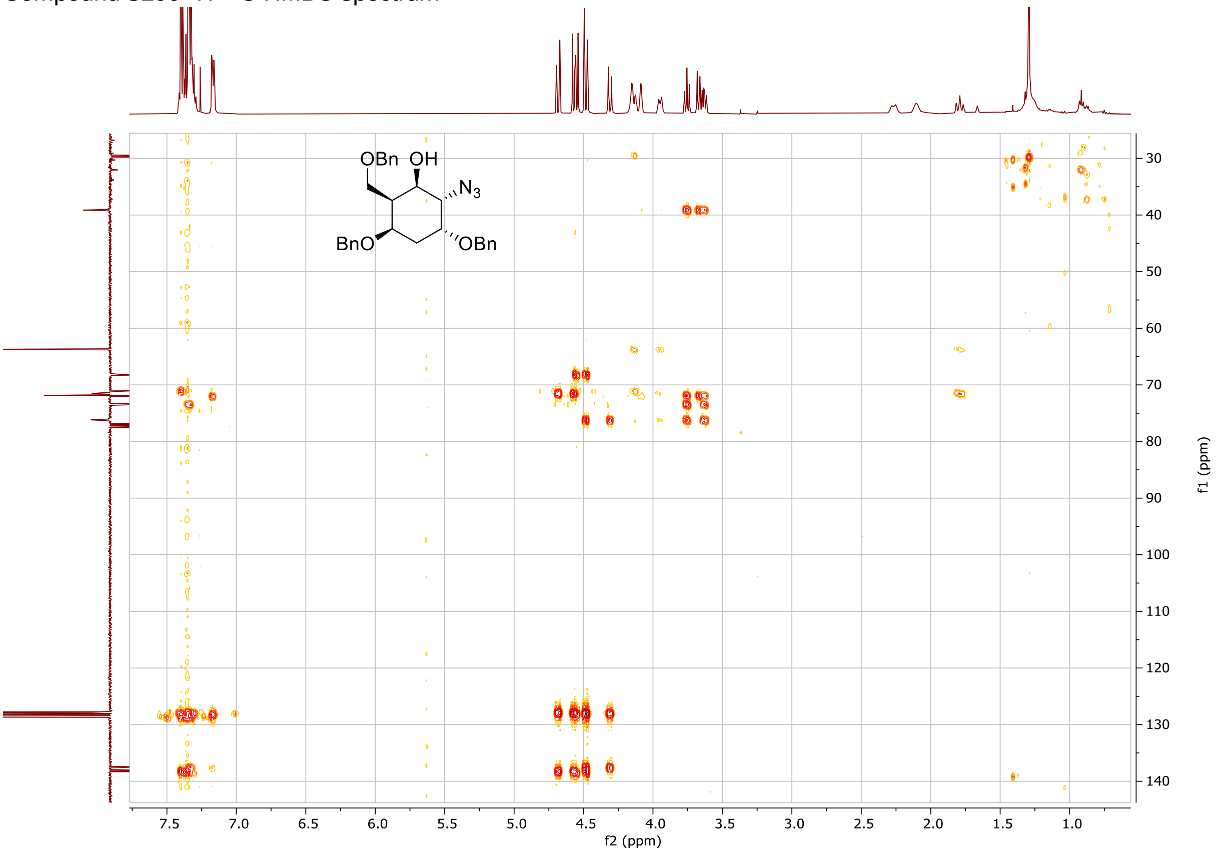

Compound **s101** ^{1}H NMR spectrum

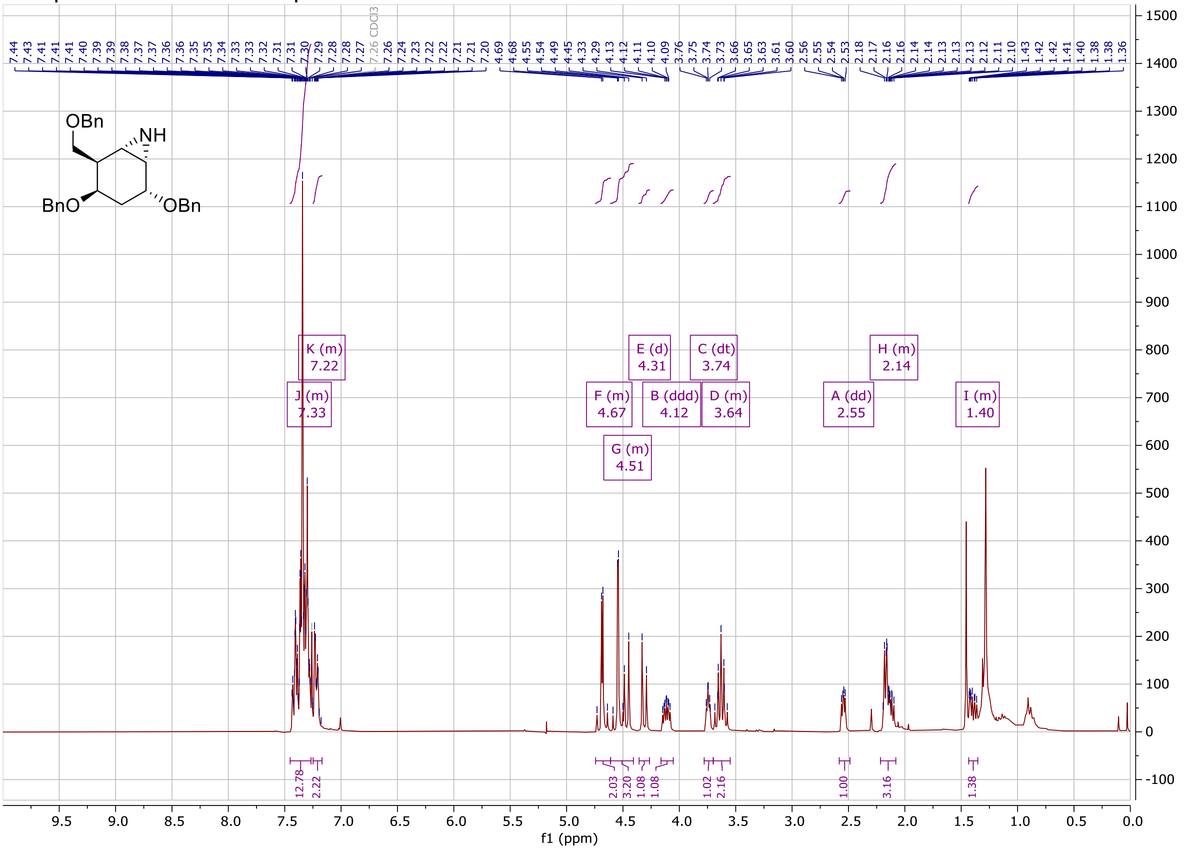

Compound **s101** ^{13}C NMR APT spectrum

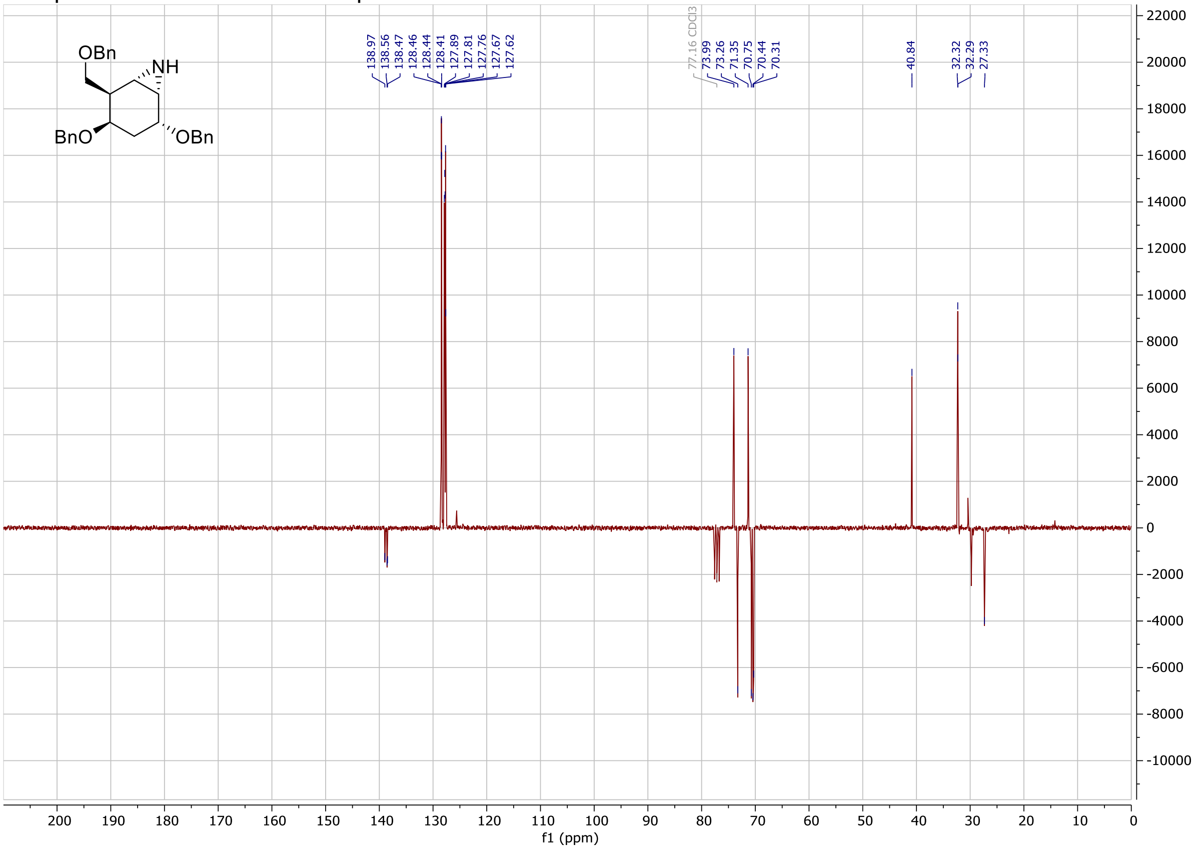

Compound **s101** ^{1}H-^{1}H COSY spectrum

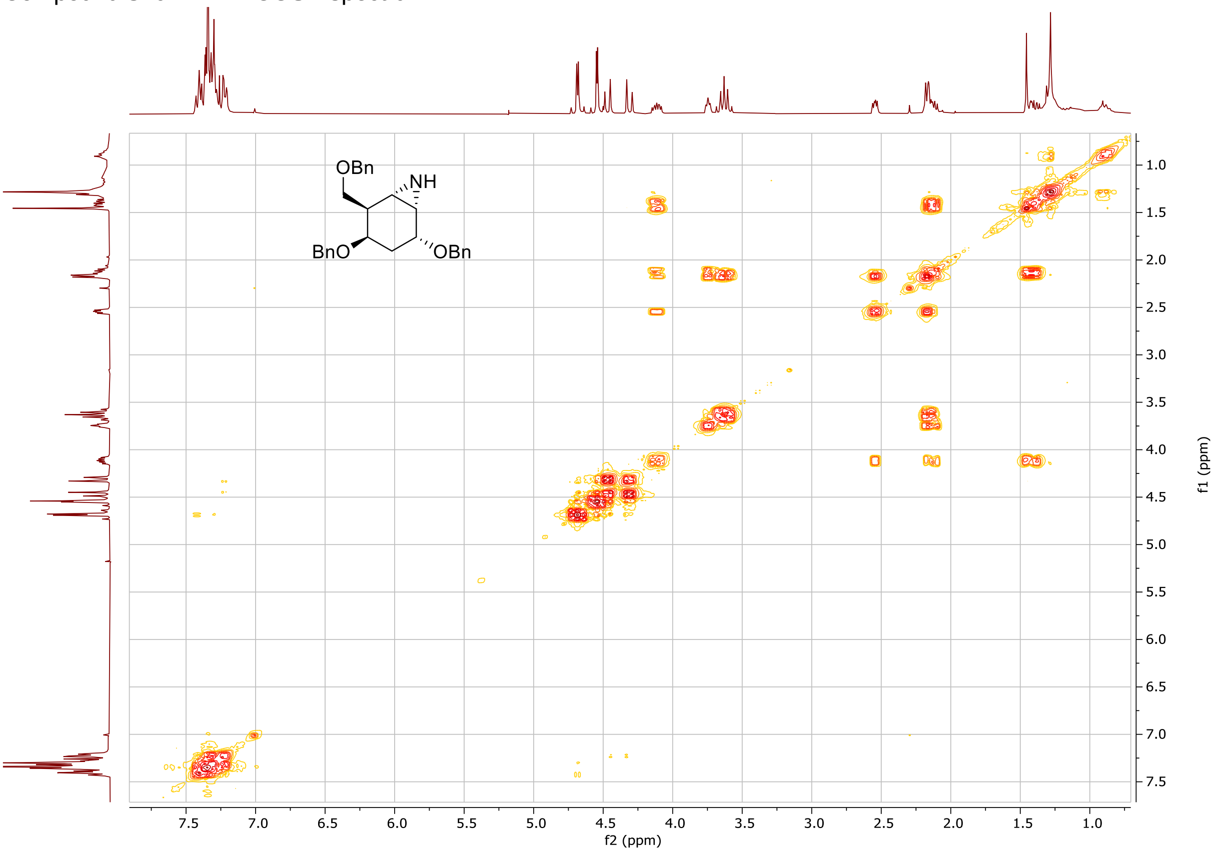

Compound **s101** ^{1}H-^{13}C HSQC spectrum

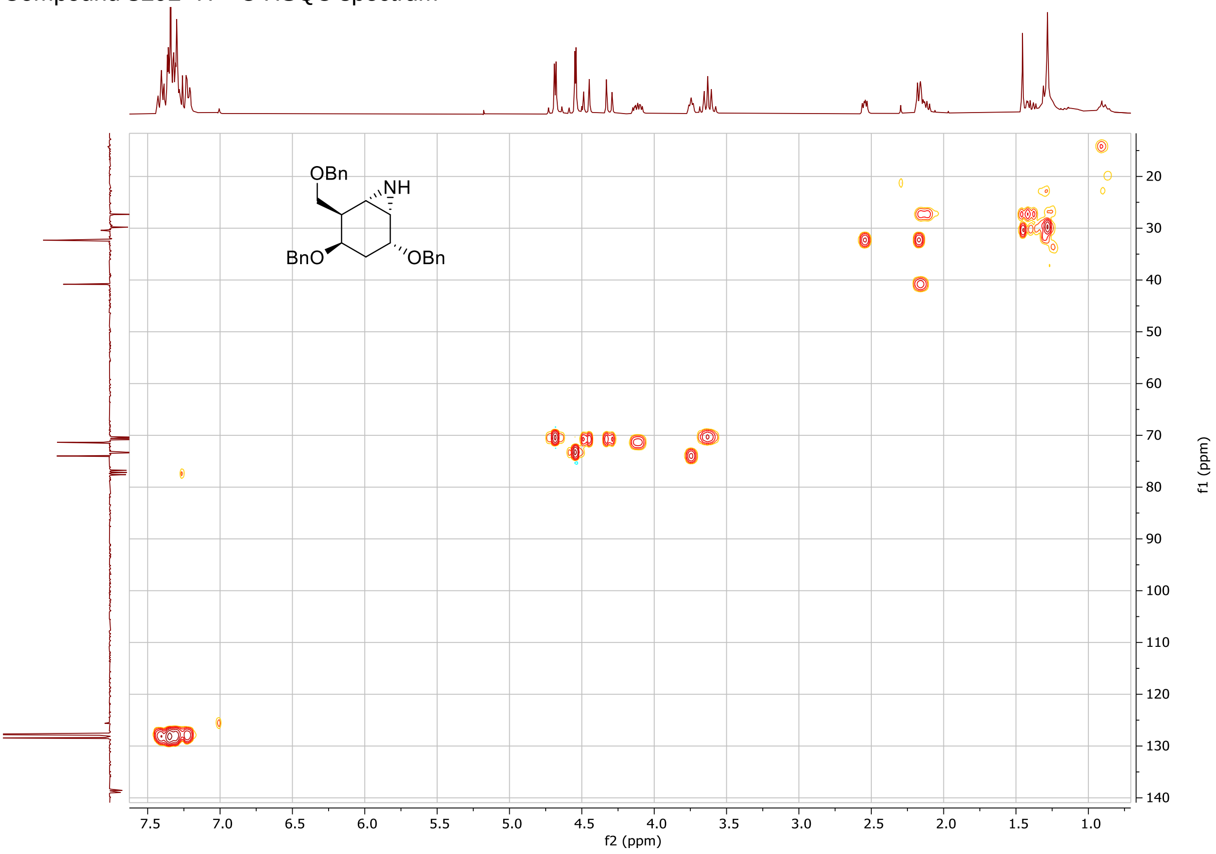

Compound **s102** ^{1}H NMR spectrum

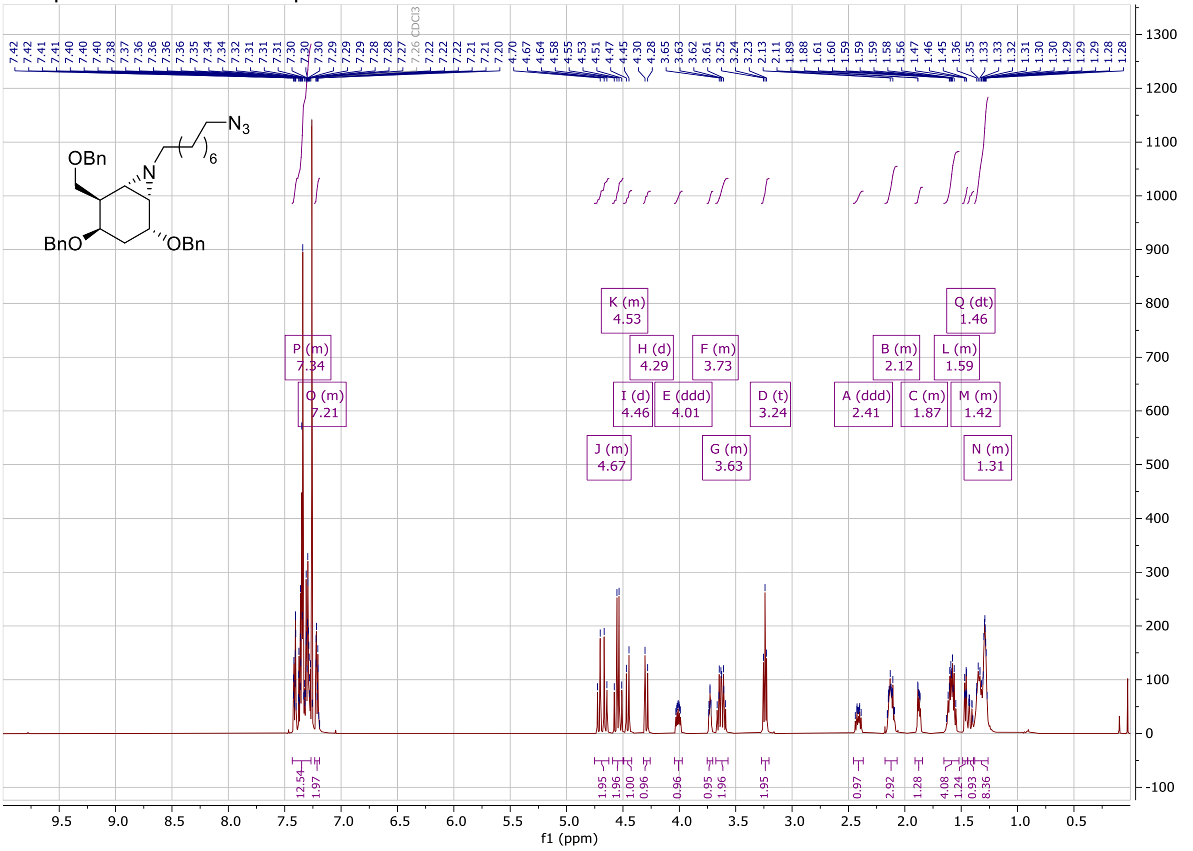

Compound **s102** ^{13}C NMR APT spectrum

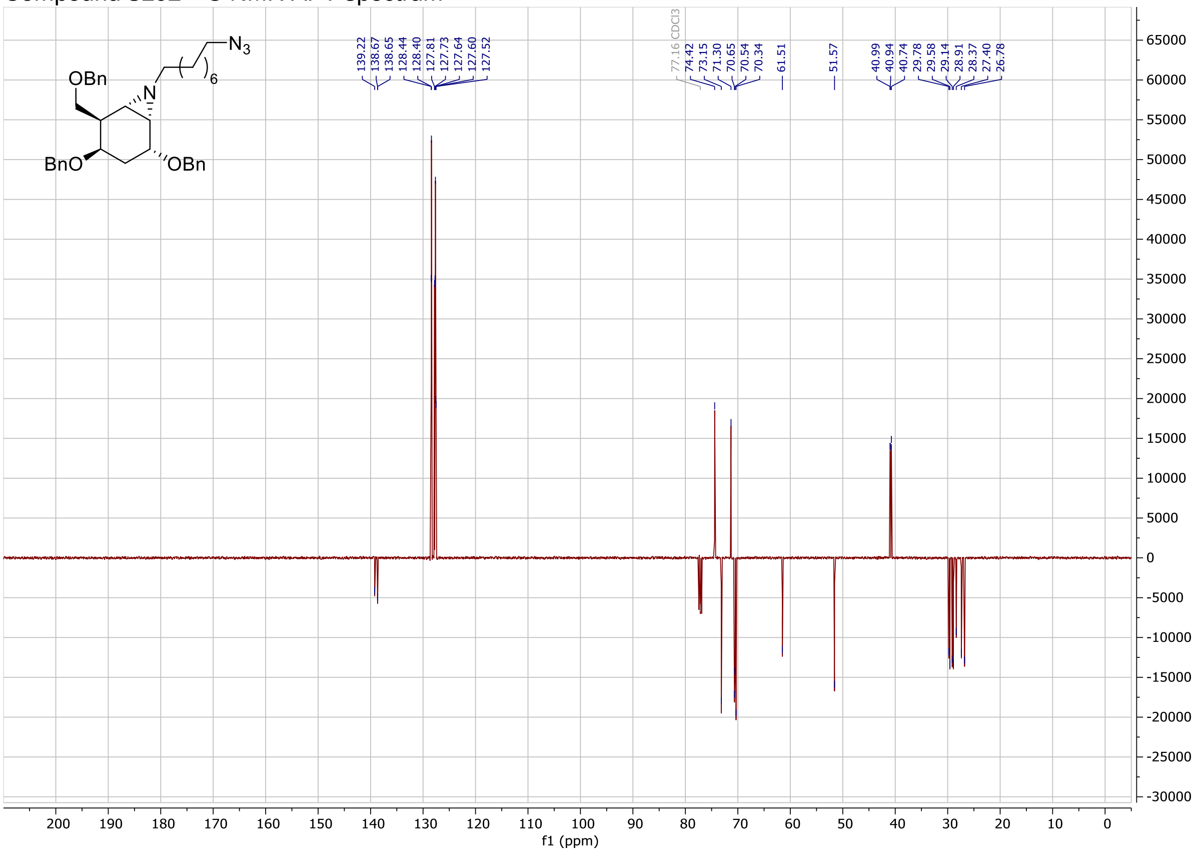

Compound **s102** ^{1}H-^{1}H COSY spectrum

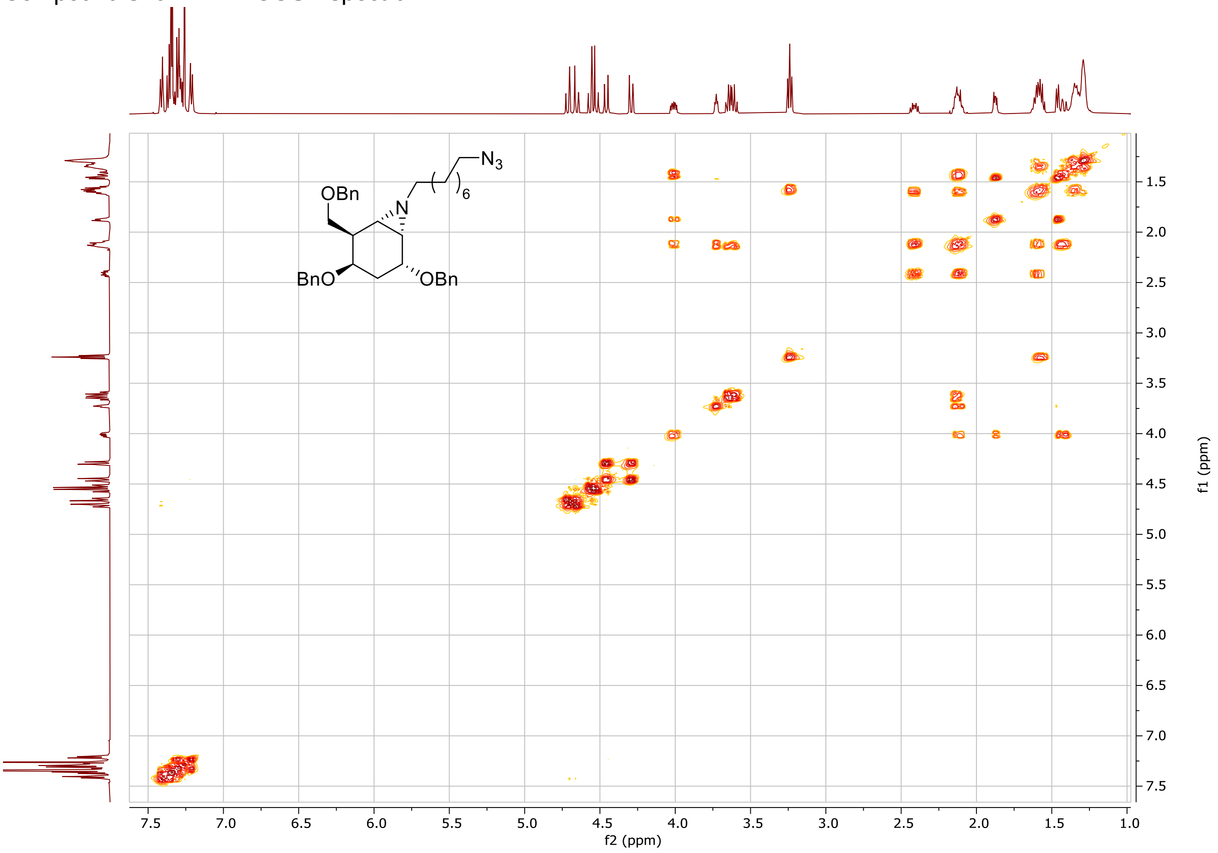

Compound **s102** ^{1}H-^{13}C HSQC spectrum

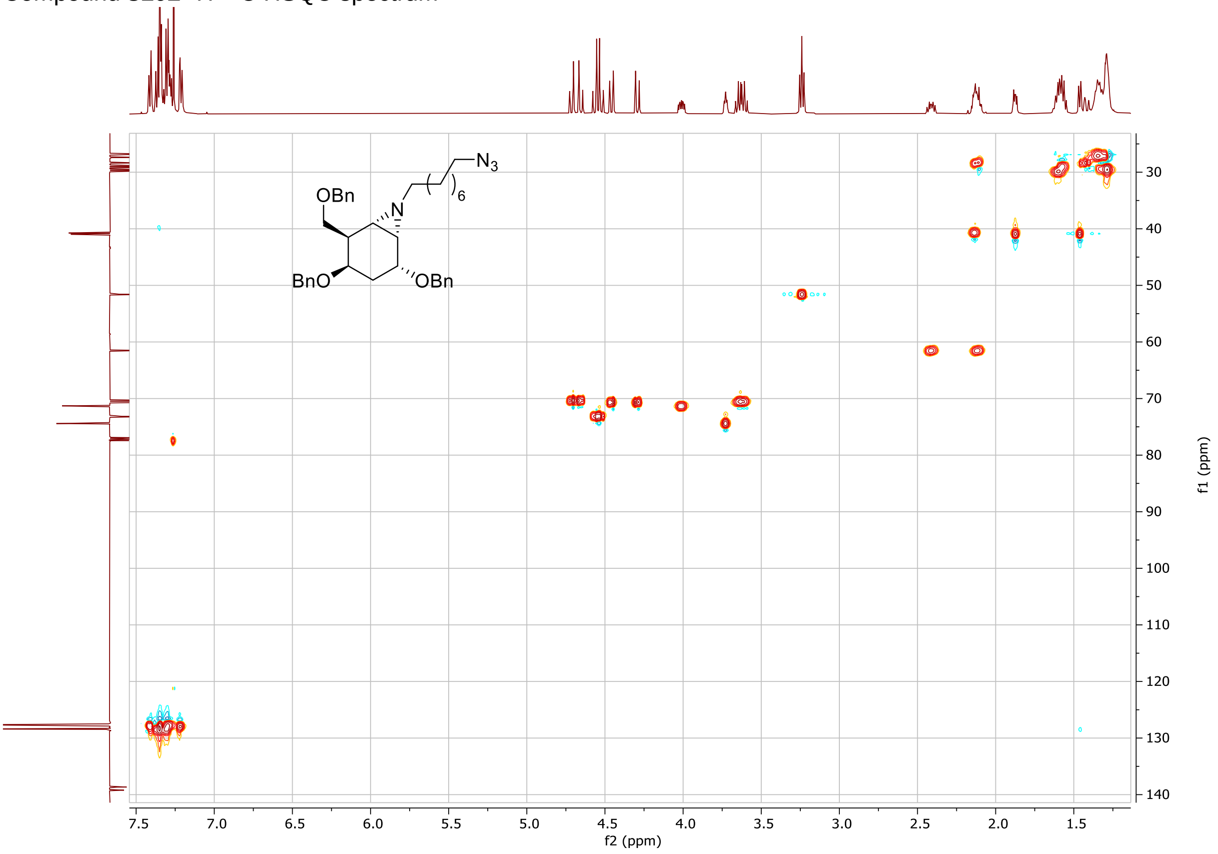

Compound **s102** ^{1}H-^{1}H NOESY spectrum

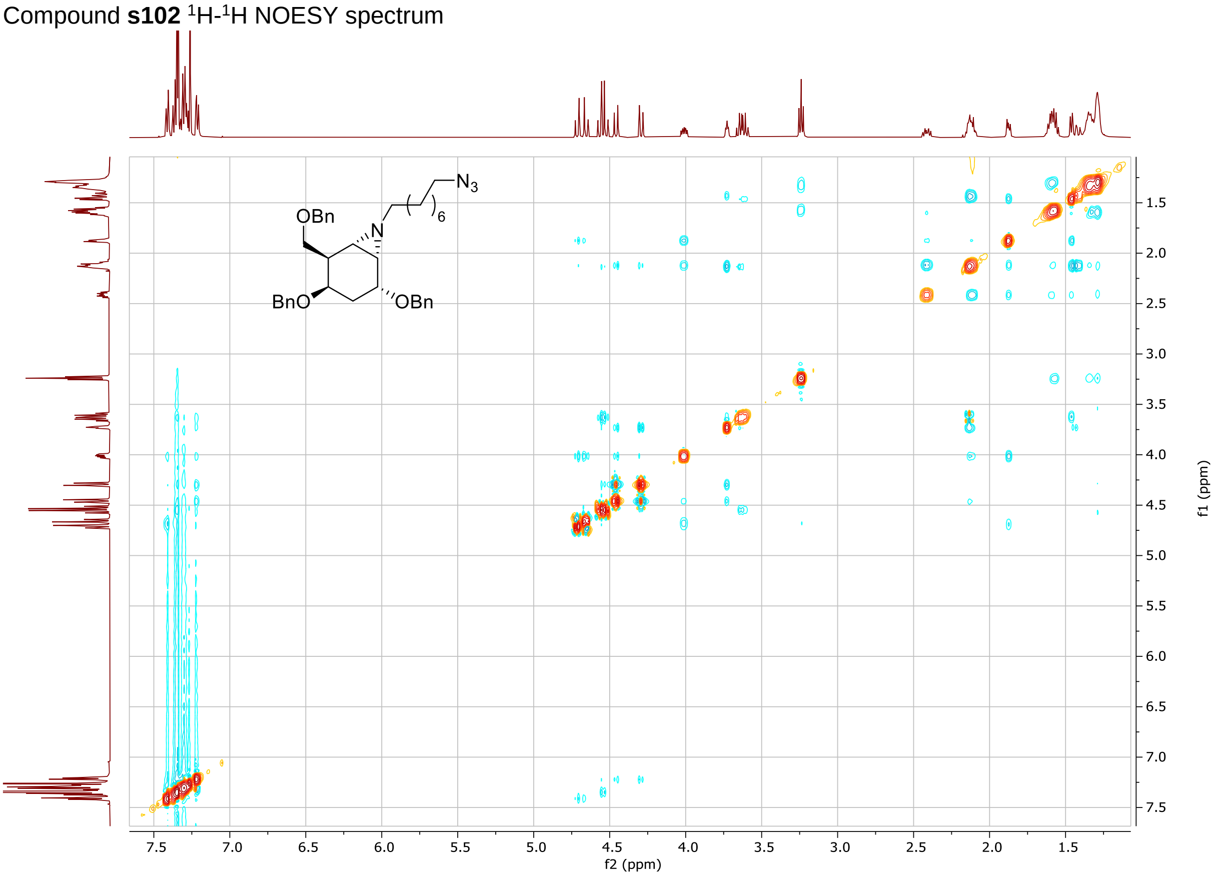

Compound **s103** ^{1}H NMR spectrum

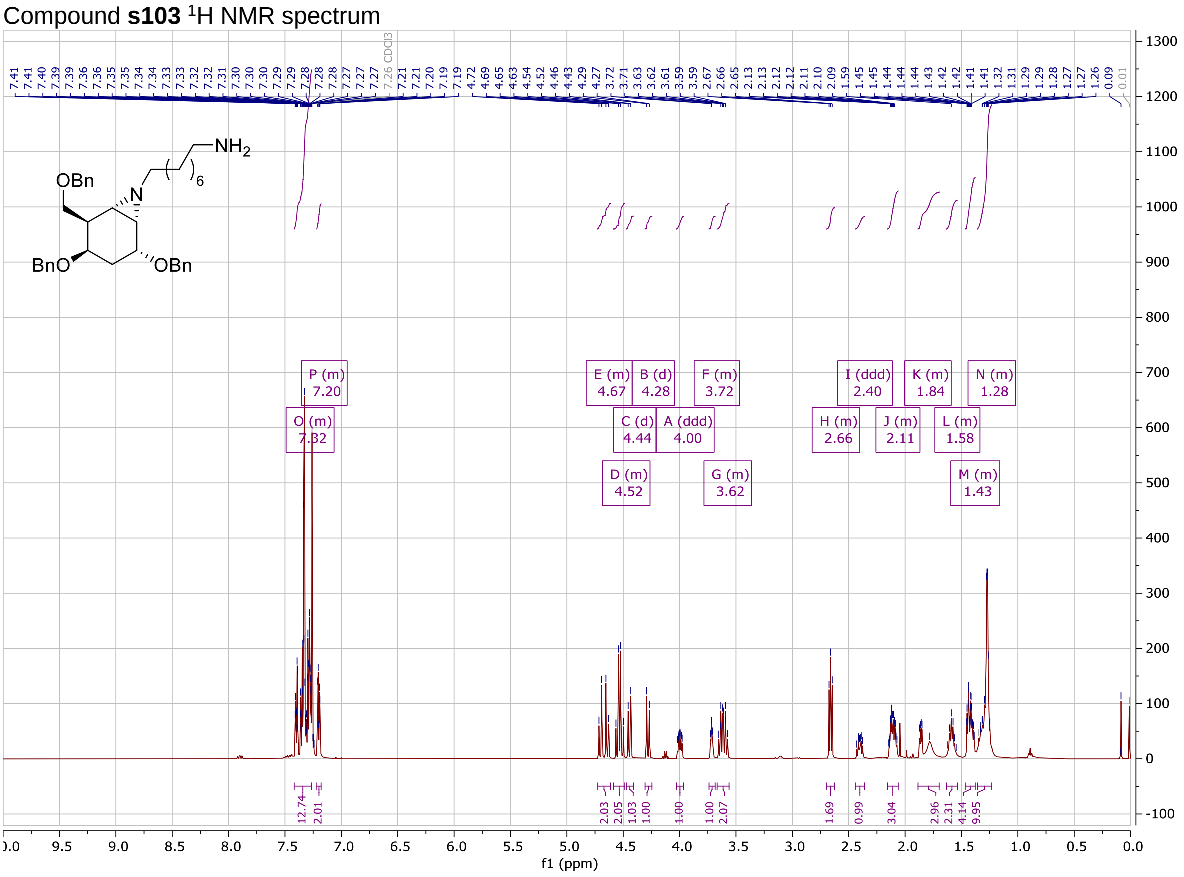

Compound **s103** ^{13}C NMR APT spectrum

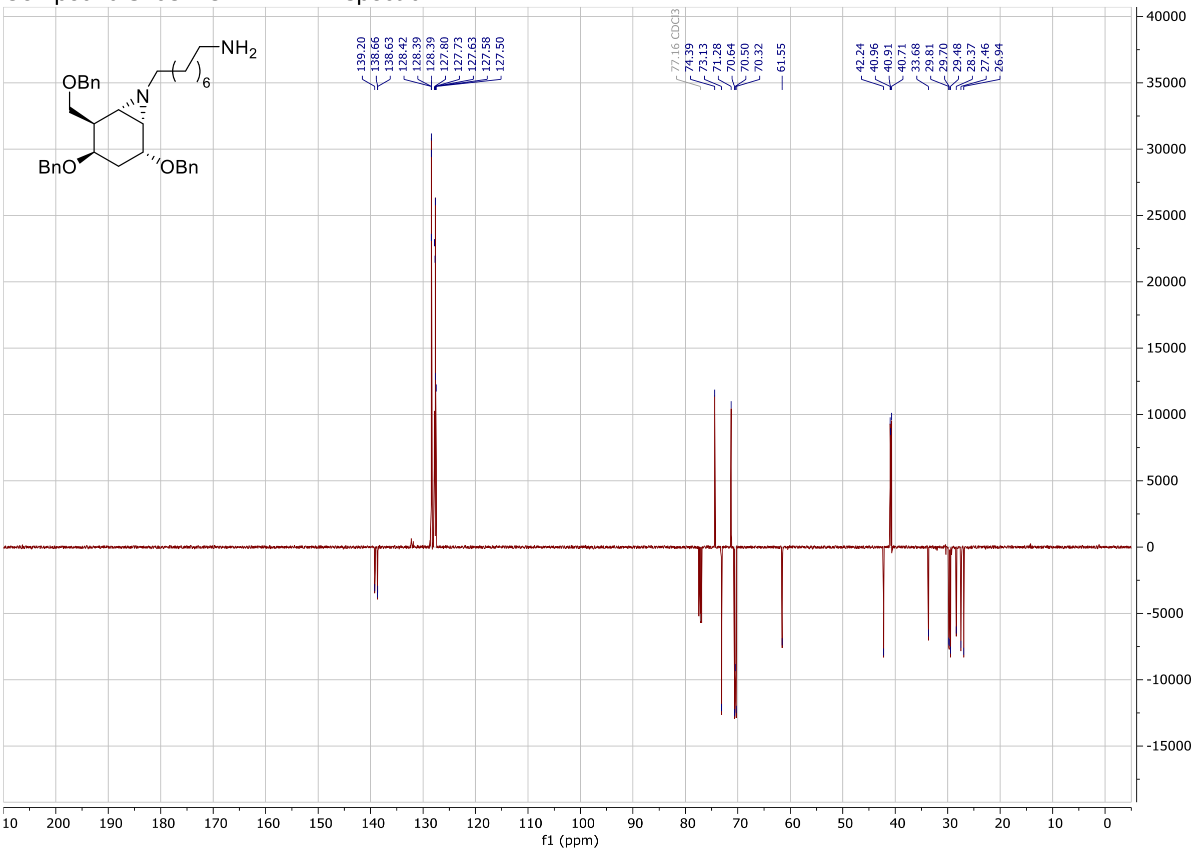

Compound **s103** ^{1}H-^{1}H COSY spectrum

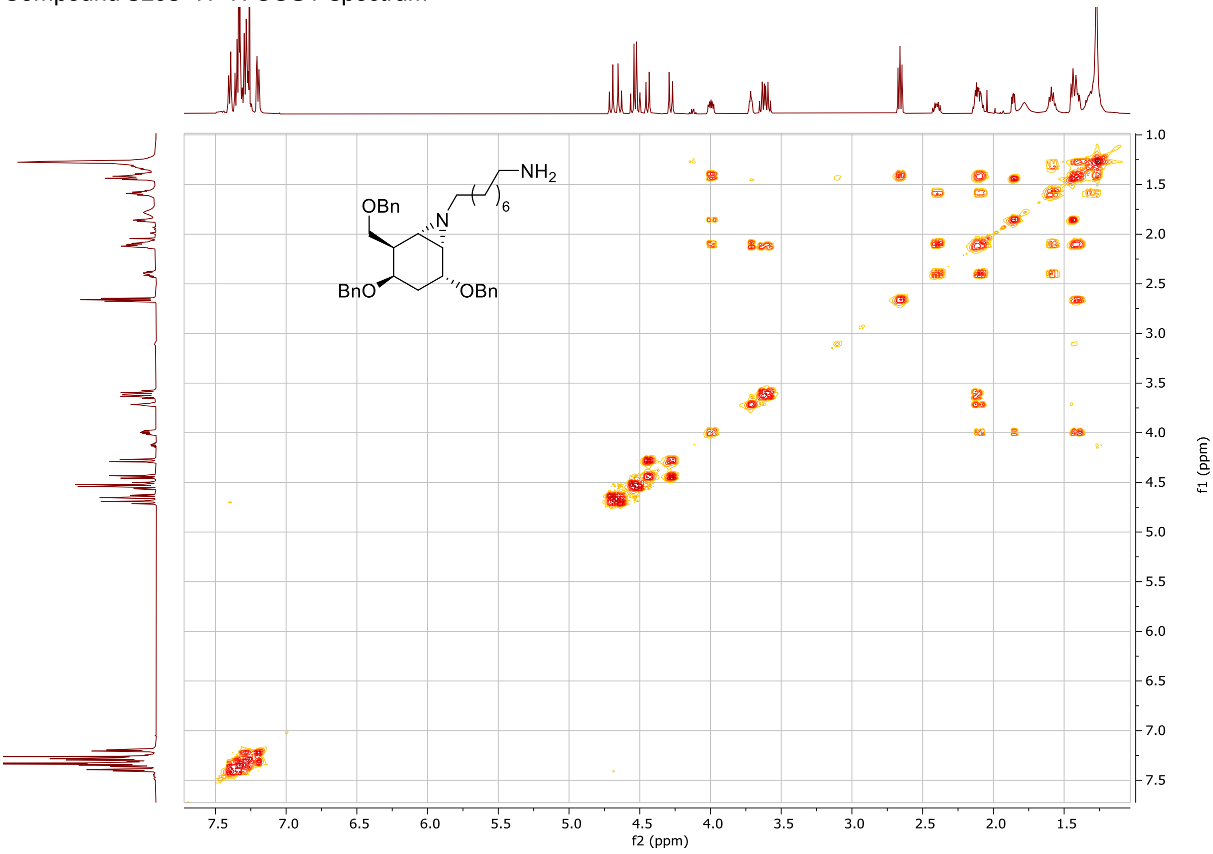

Compound **s103** ^{1}H-^{13}C HSQC spectrum

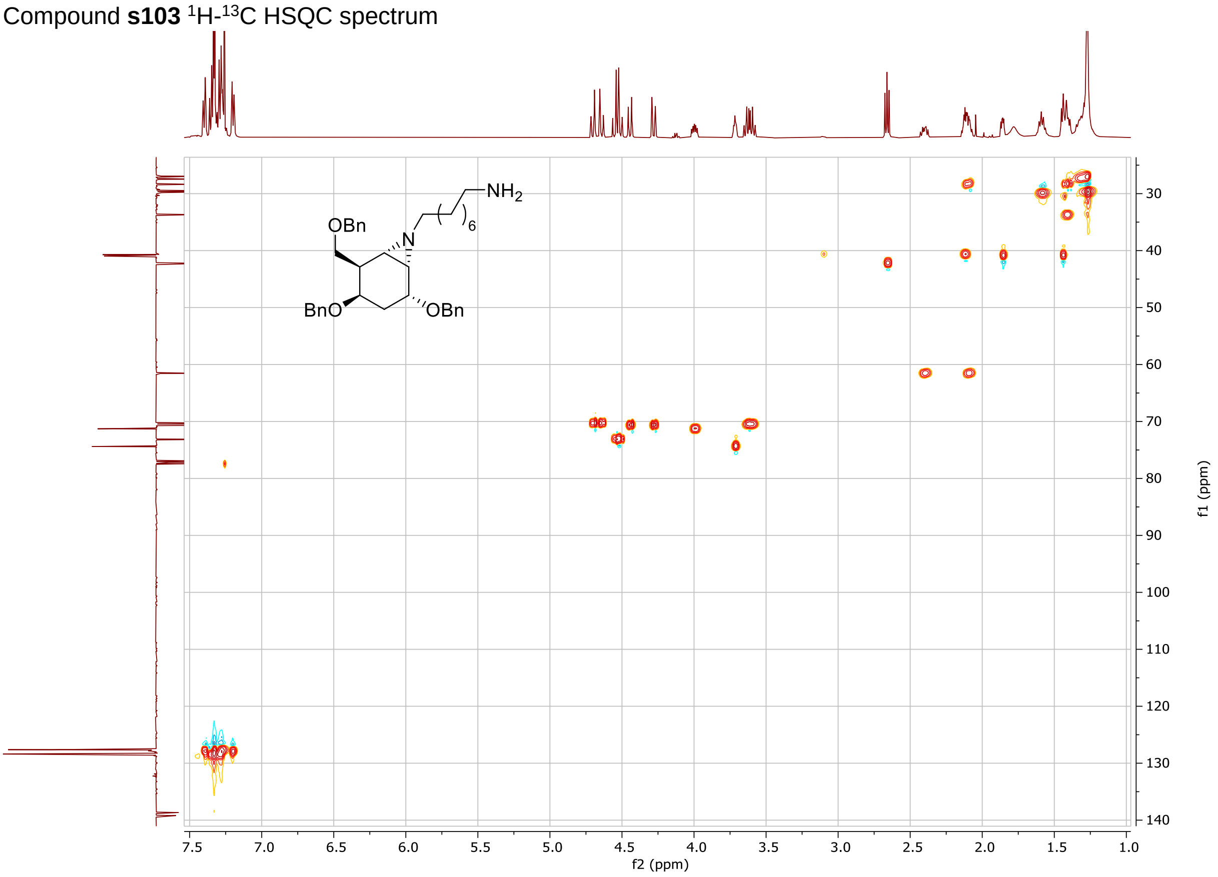

Compound **s104** ^{1}H NMR spectrum

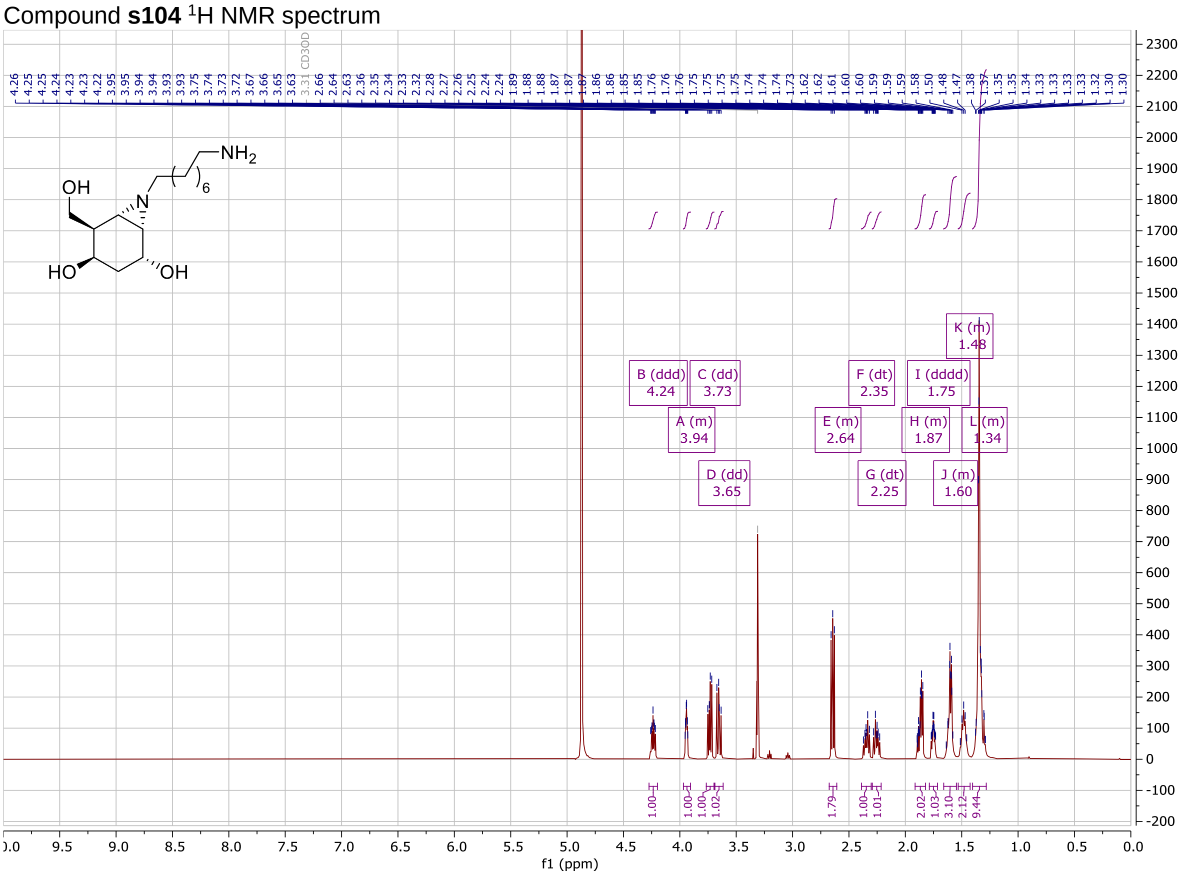

Compound **s104** ^{13}C NMR APT spectrum

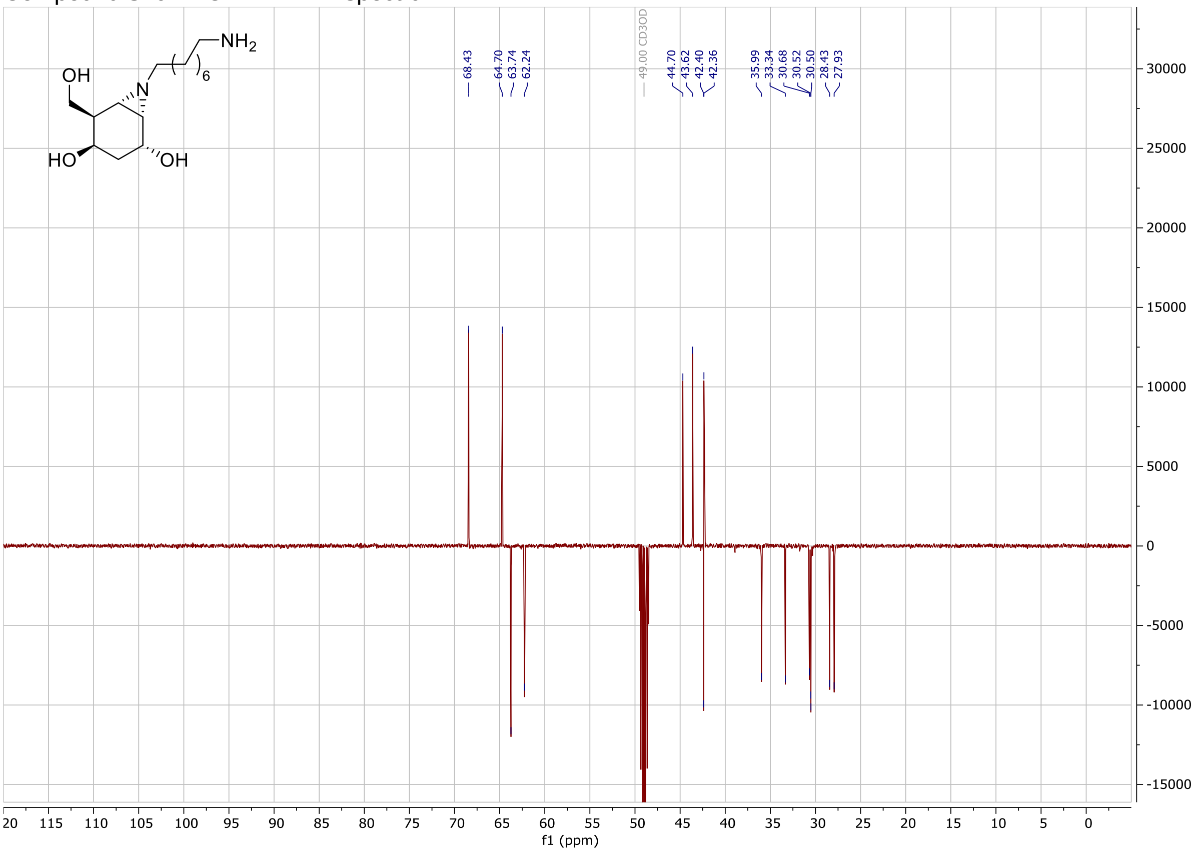

Compound **s104** ^{1}H-^{1}H COSY spectrum

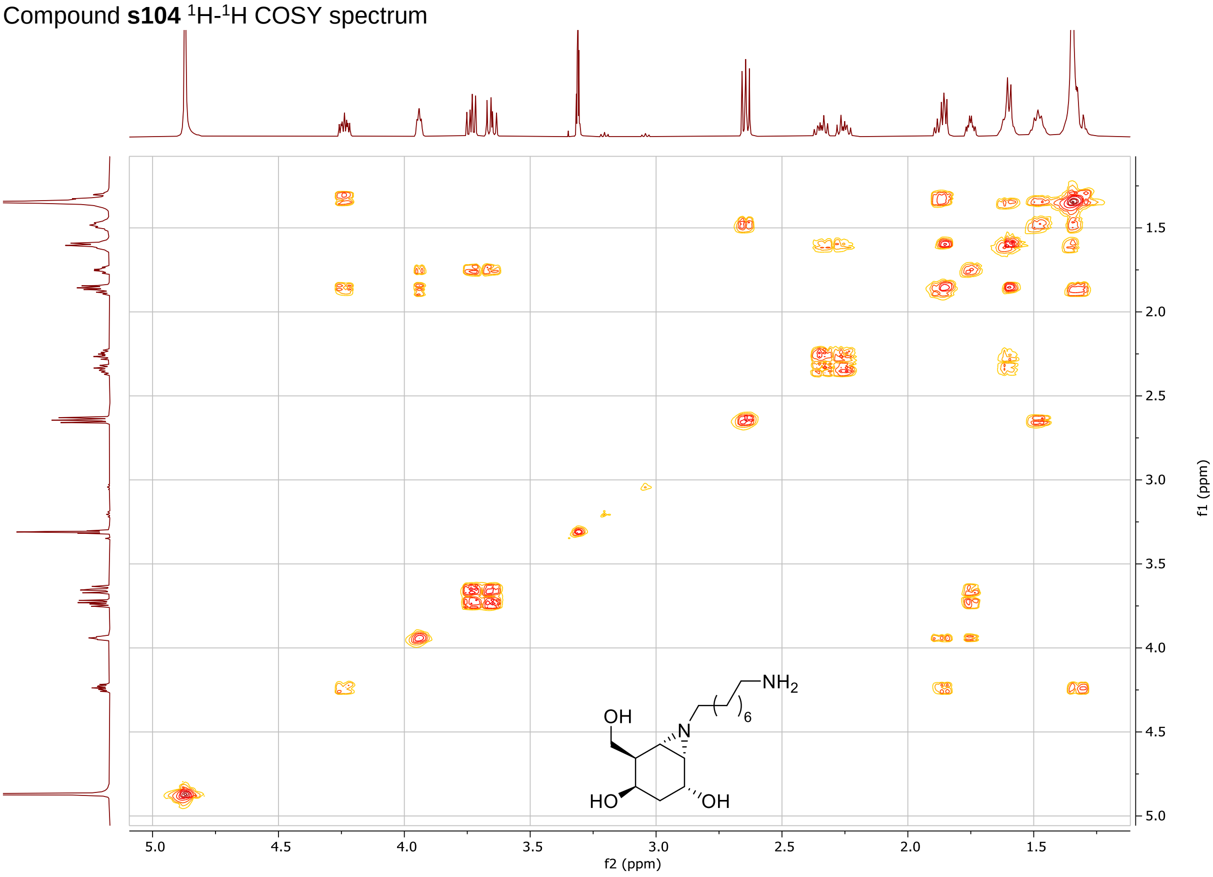

Compound **s104** ^{1}H-^{13}C HSQC spectrum

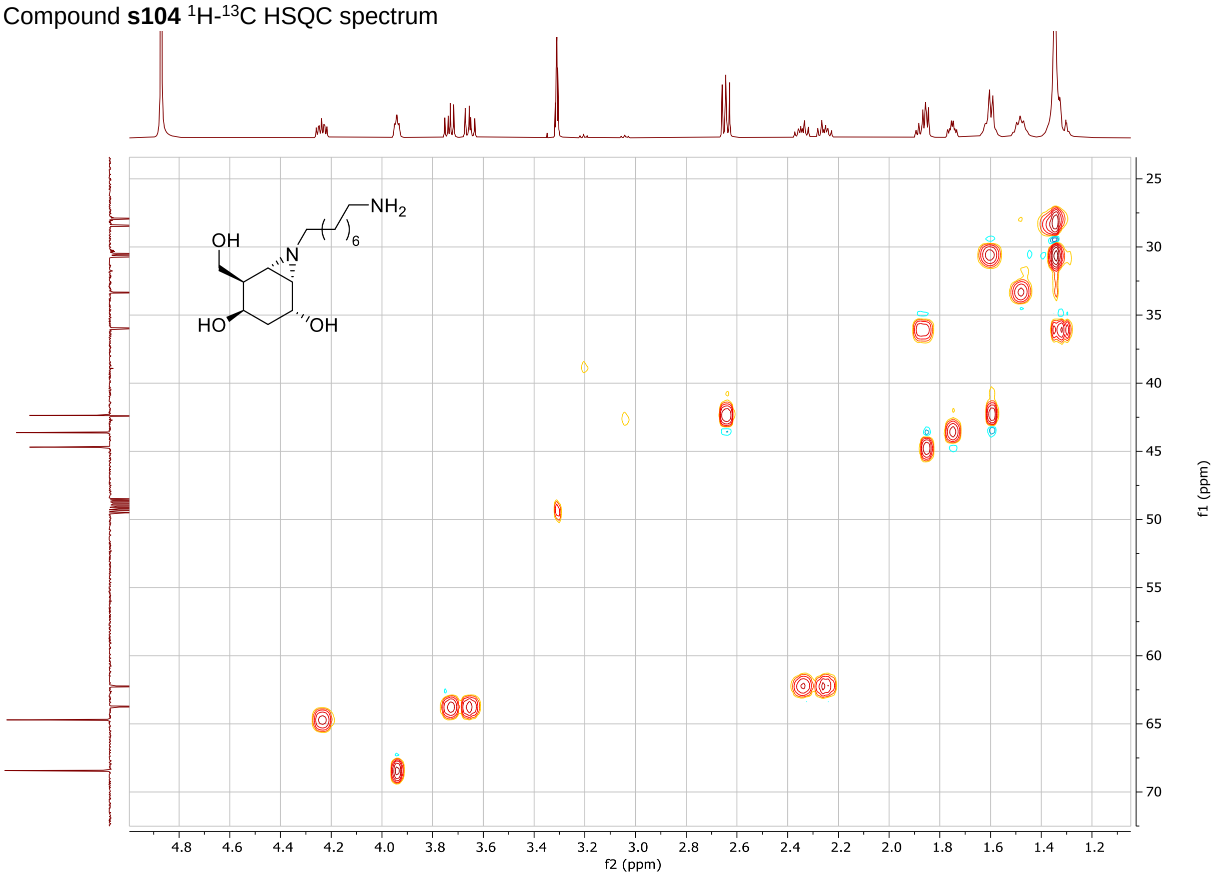

Compound **47** ^{1}H NMR spectrum

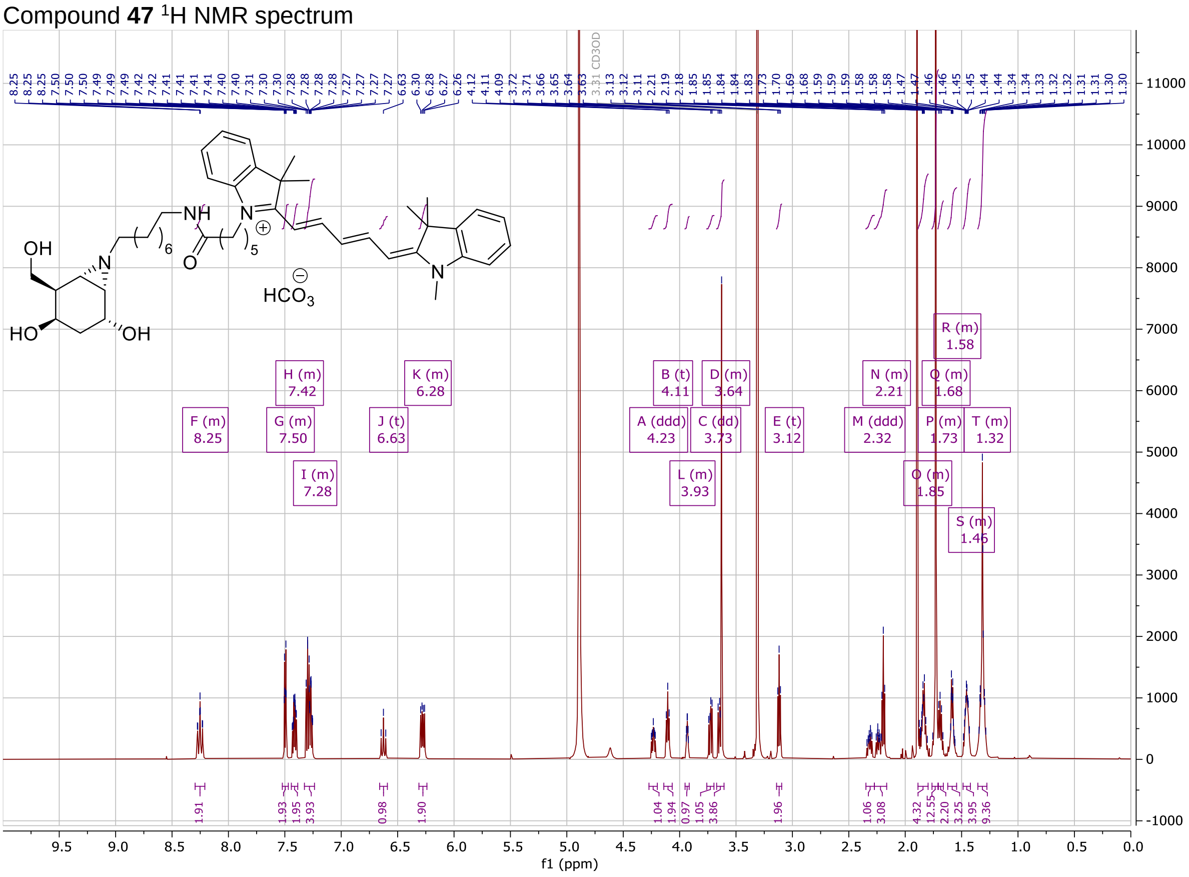

Compound **47** ^{13}C NMR APT spectrum

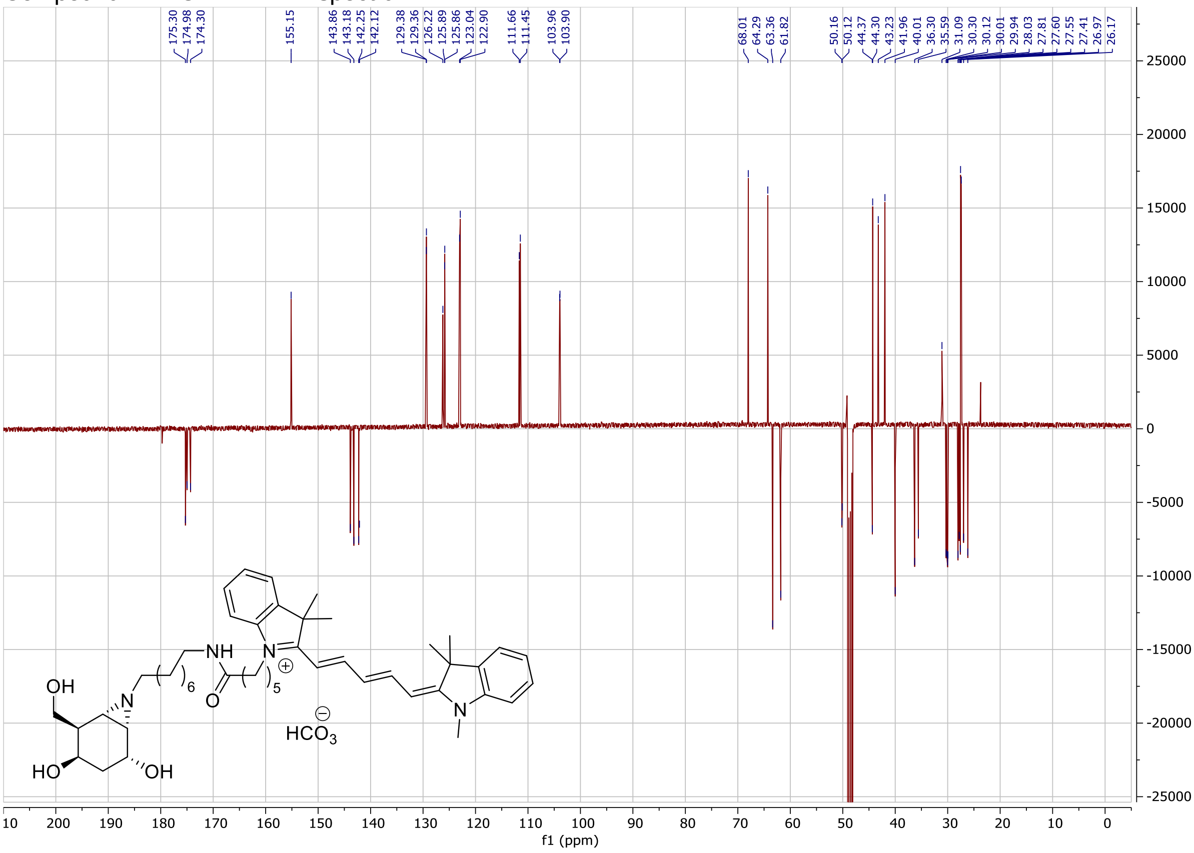

Compound **47** ^{1}H-^{1}H COSY spectrum

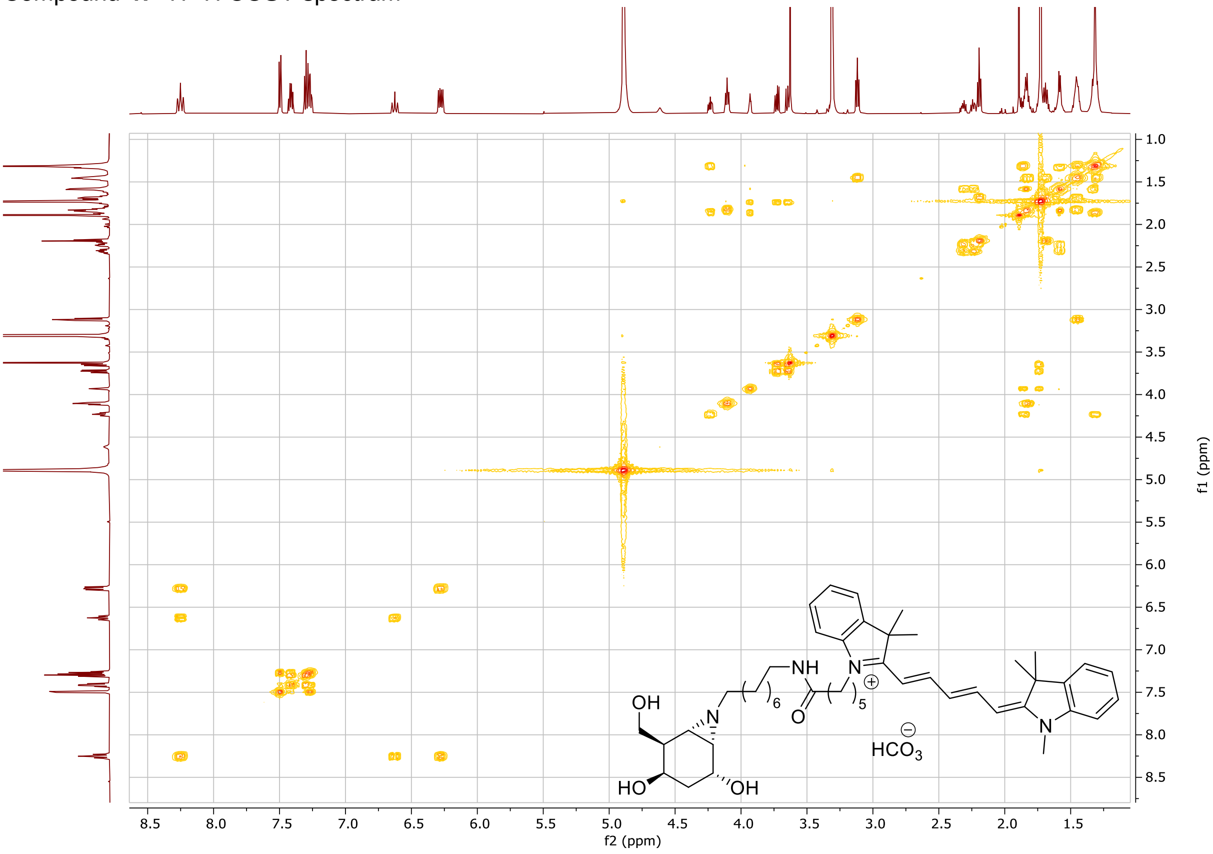

Compound **47** ^{1}H-^{13}C HSQC spectrum

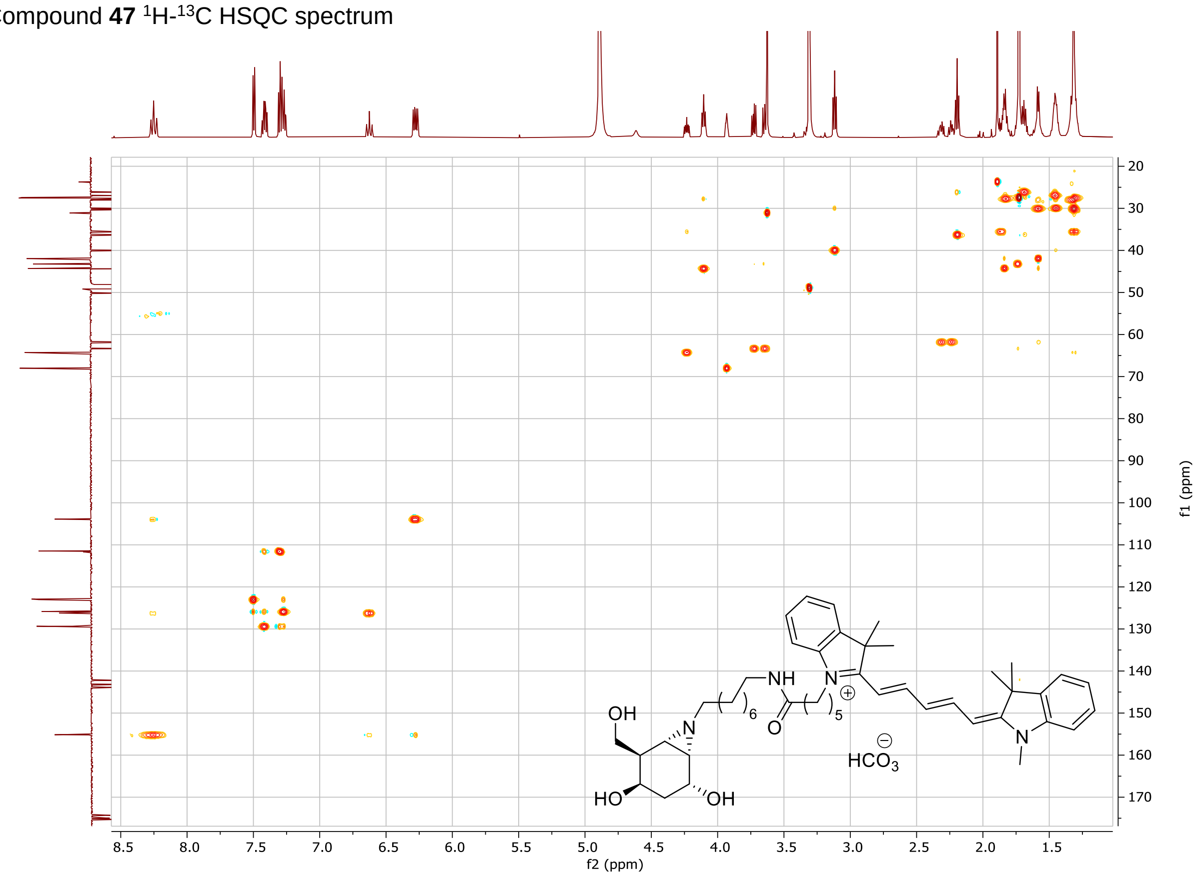

Compound **54** ^{1}H NMR spectrum

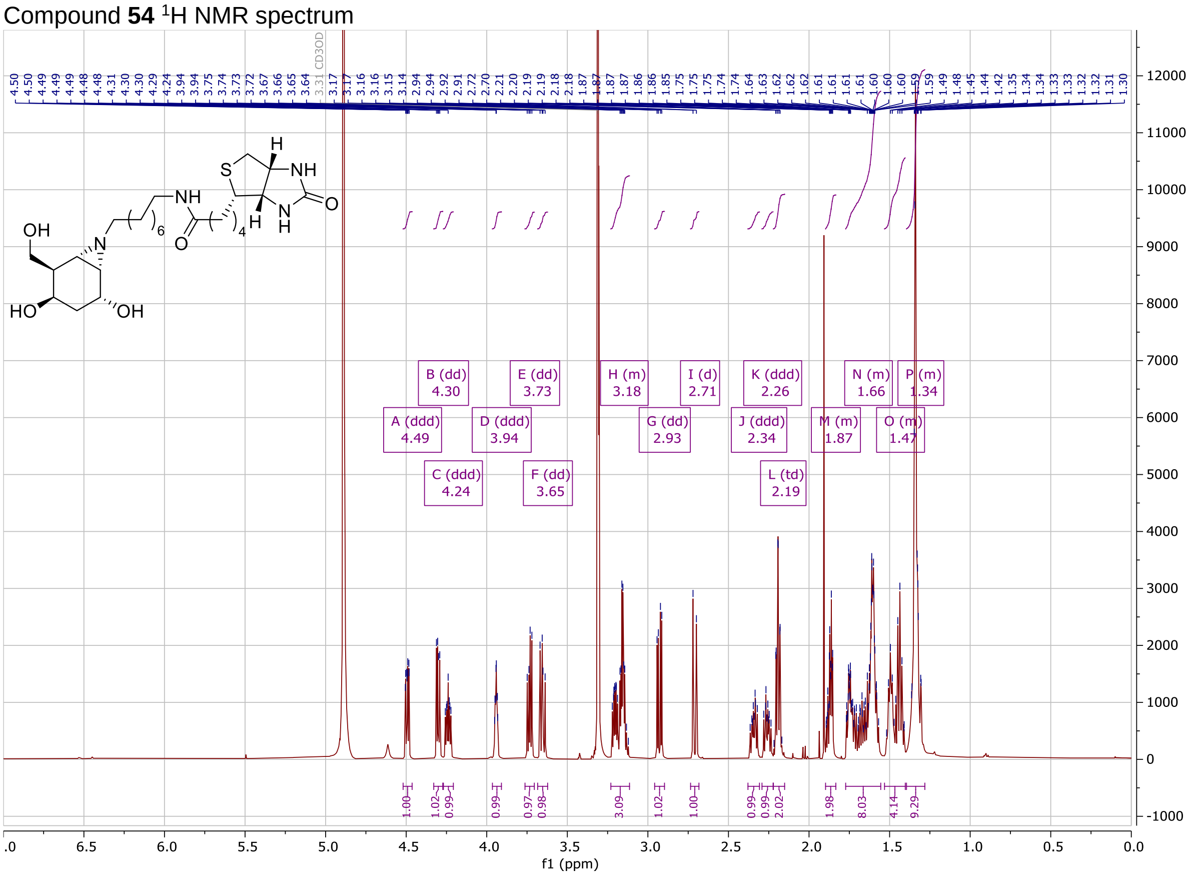

Compound **54** ^{13}C NMR APT spectrum

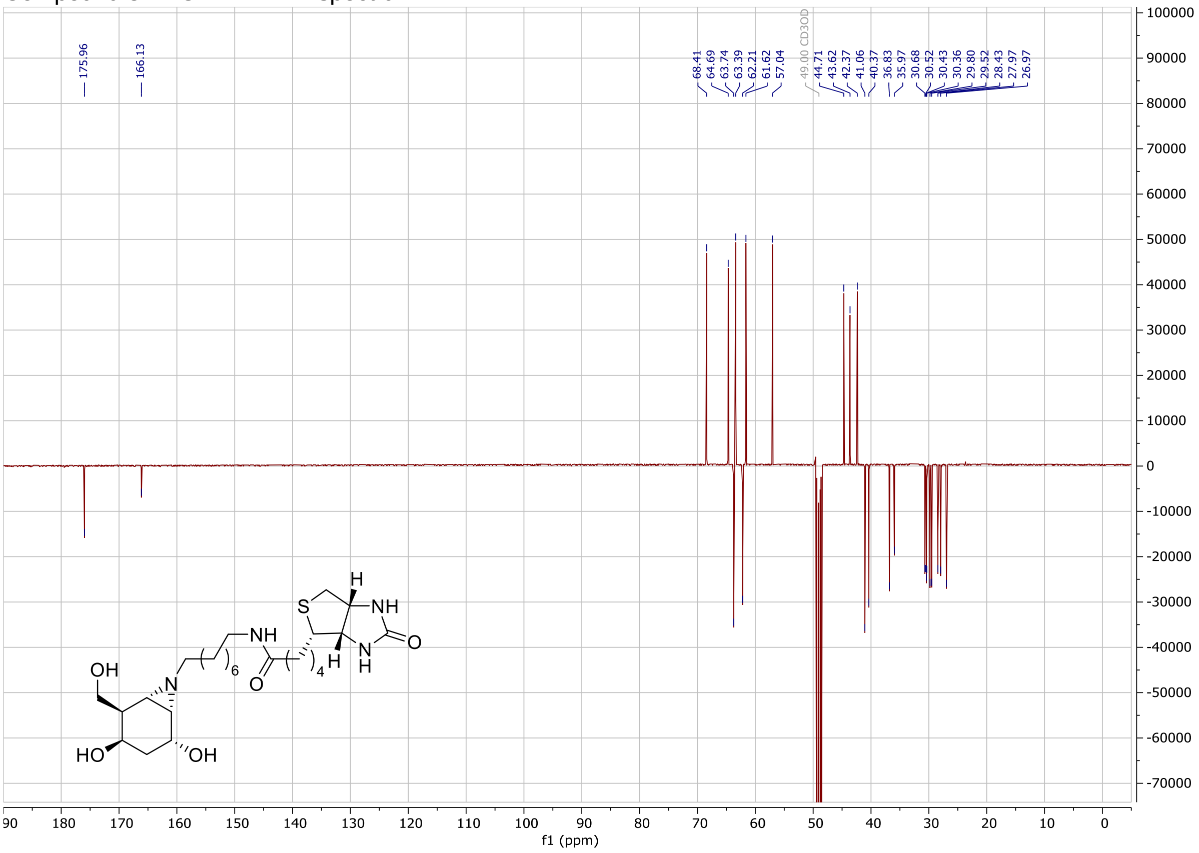

Compound **54** ^{1}H-^{1}H COSY spectrum

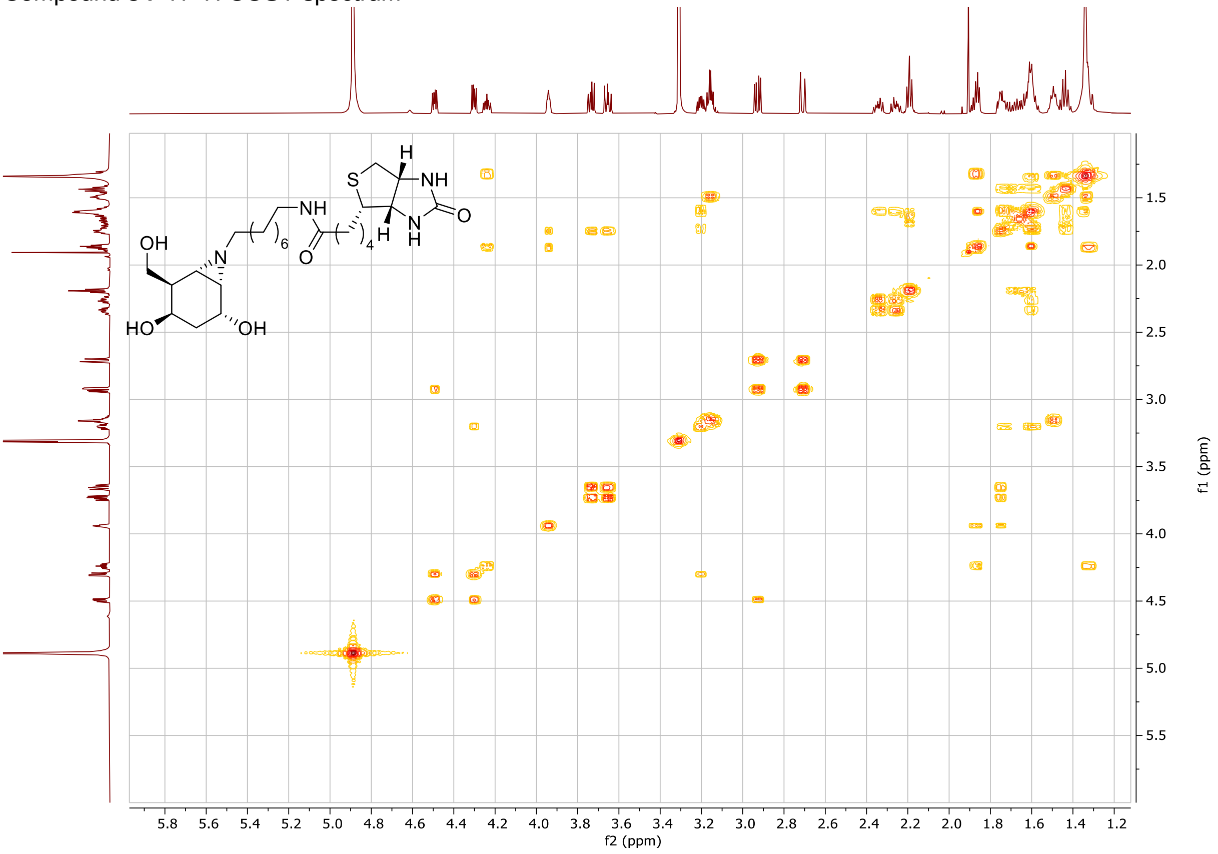

Compound **54** ^{1}H-^{13}C HSQC spectrum

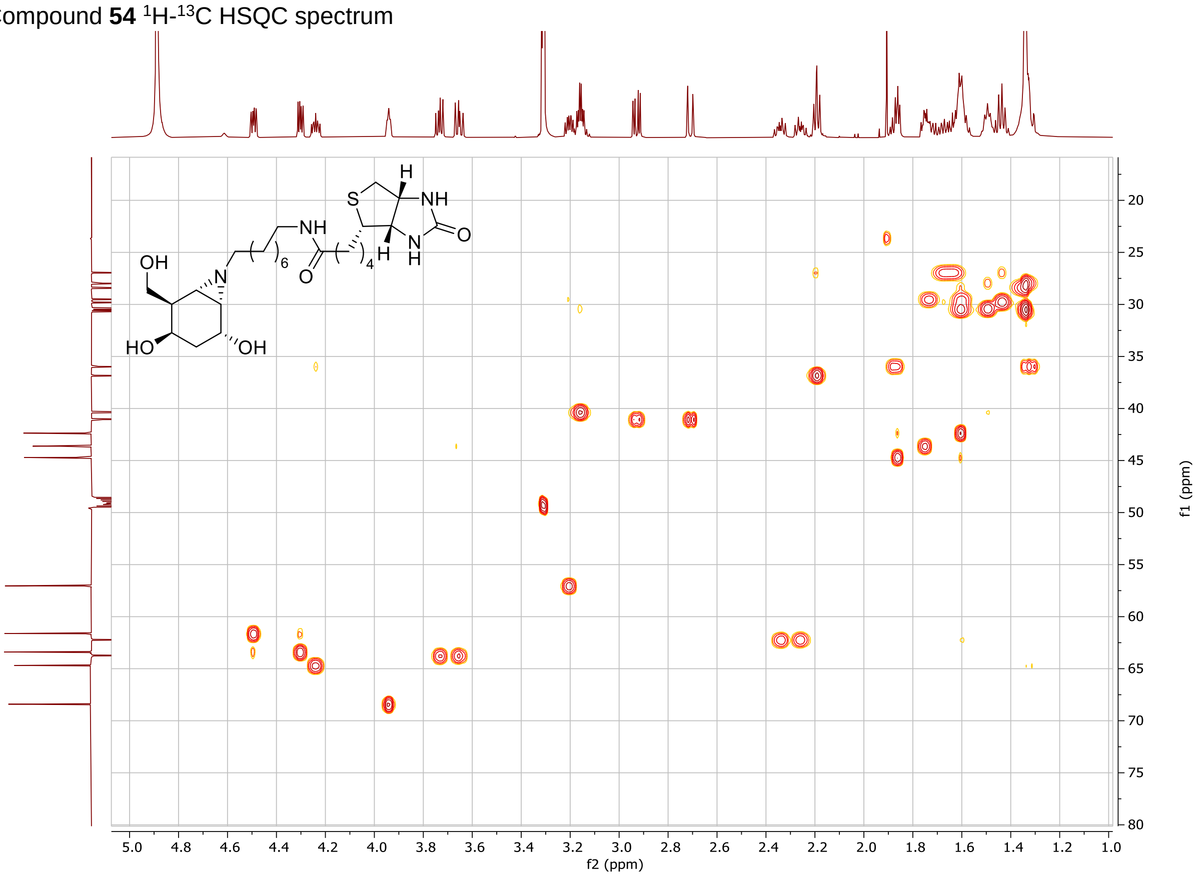

Compound **s105** ^{1}H NMR spectrum

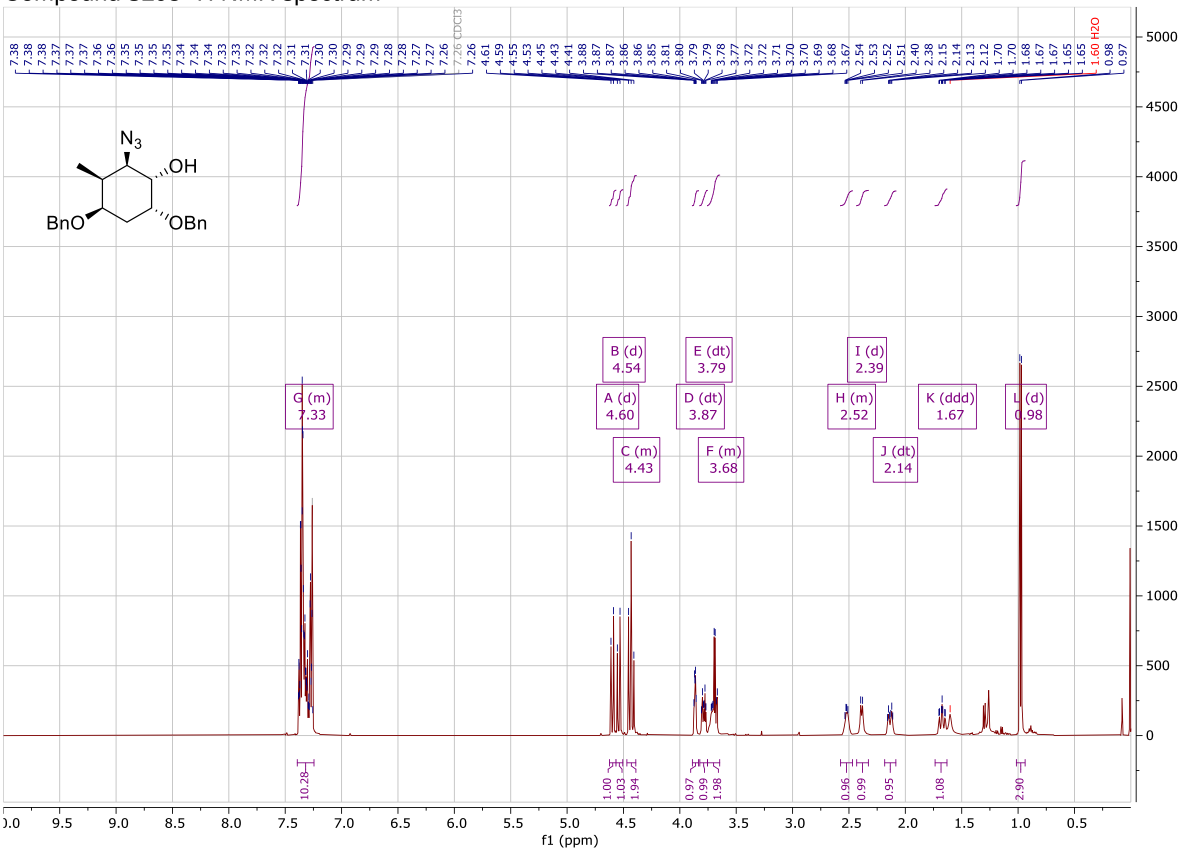

Compound **s105** ^{13}C NMR BBD spectrum

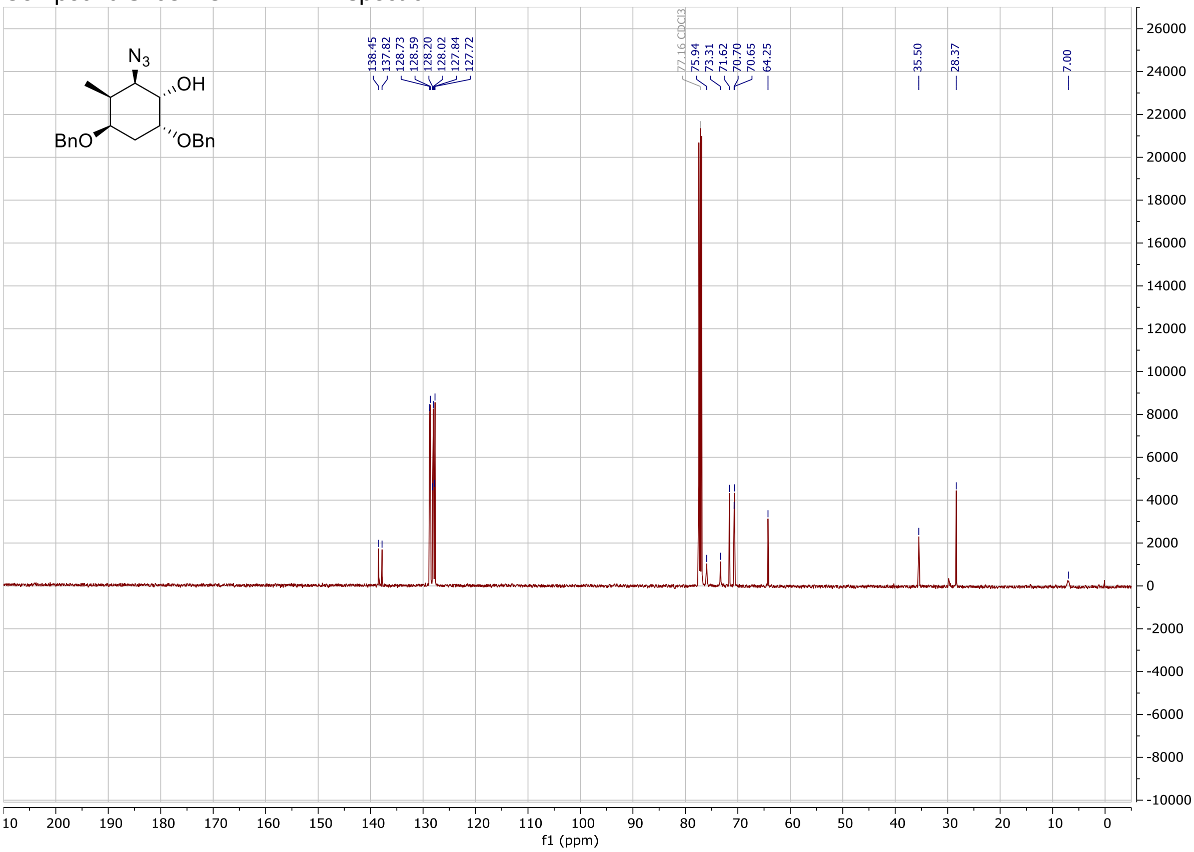

Compound **s105** ^{1}H-^{1}H COSY spectrum

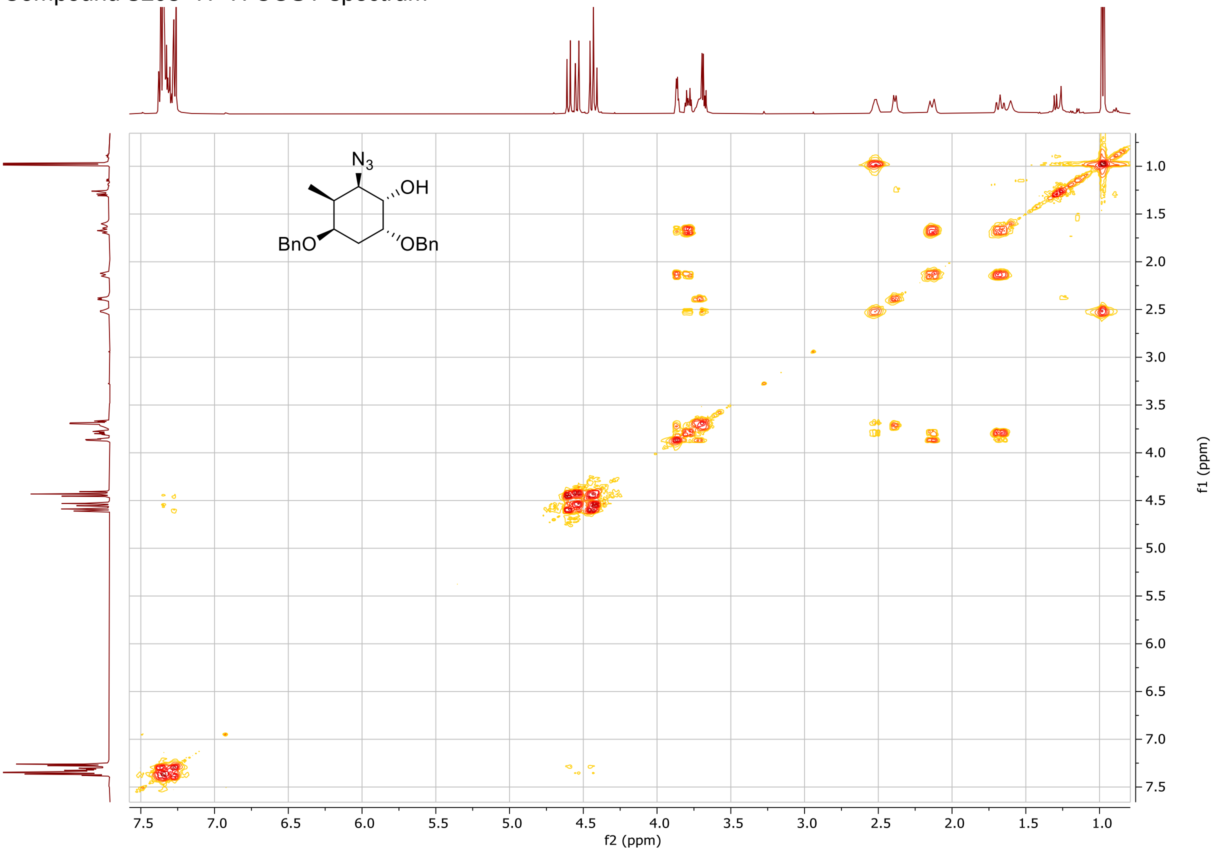

Compound **s105** ^{1}H-^{13}C HSQC spectrum

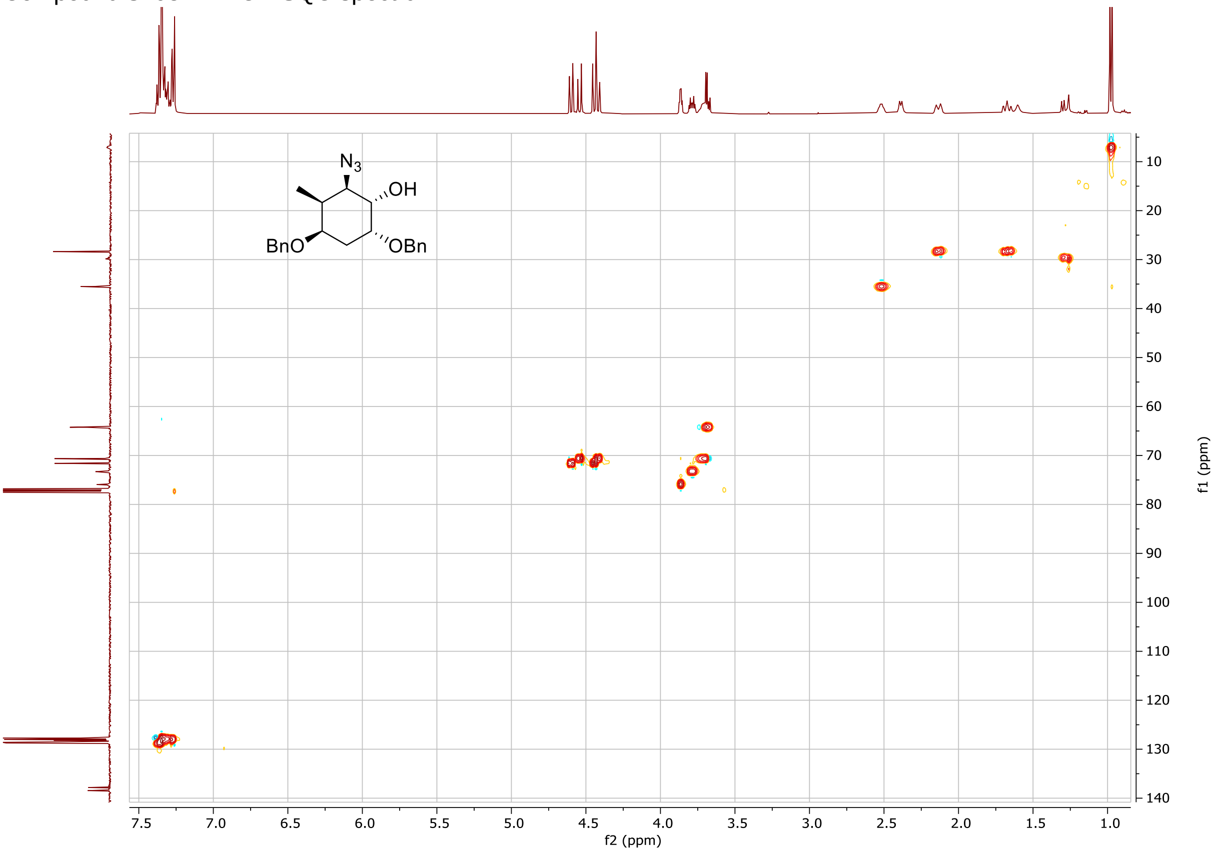

Compound **s105** ^{1}H-^{1}H NOESY spectrum

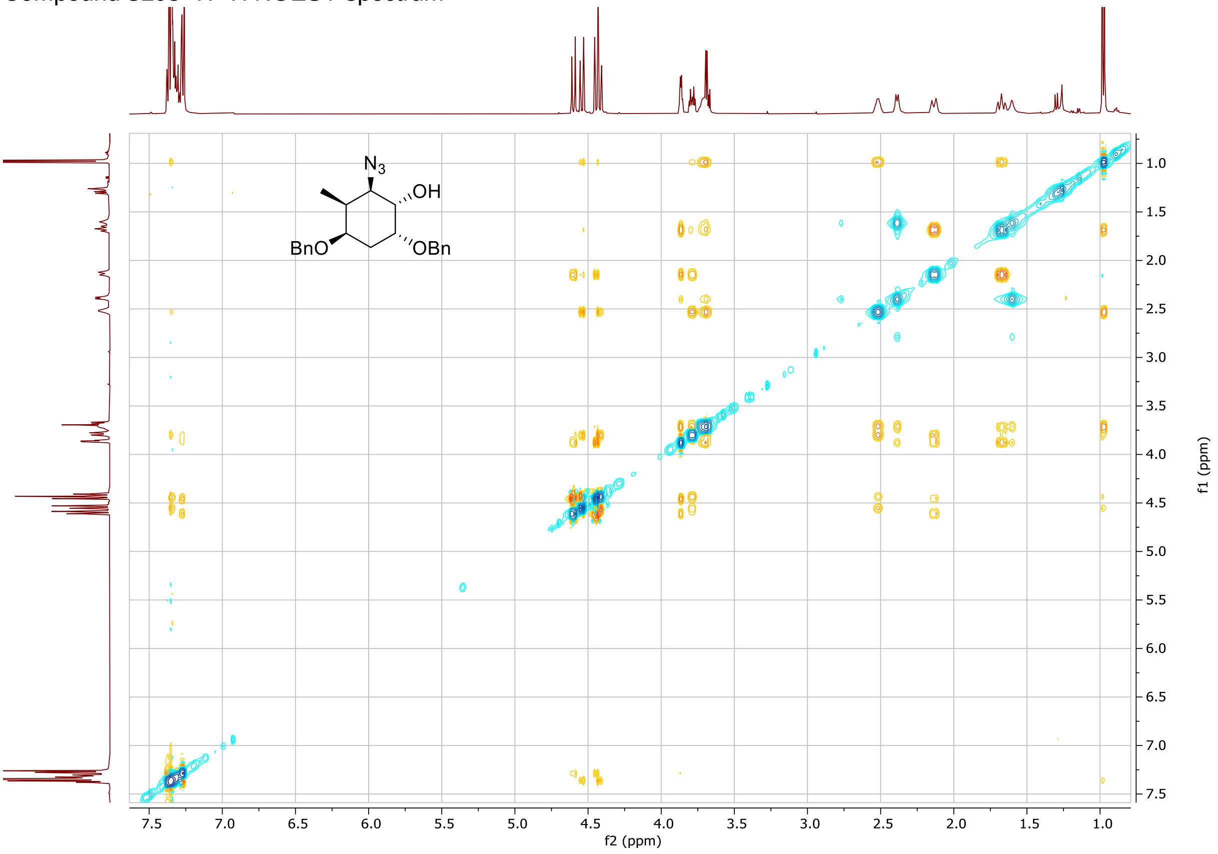

Compound **s105** ^{1}H-^{13}C HMBC spectrum

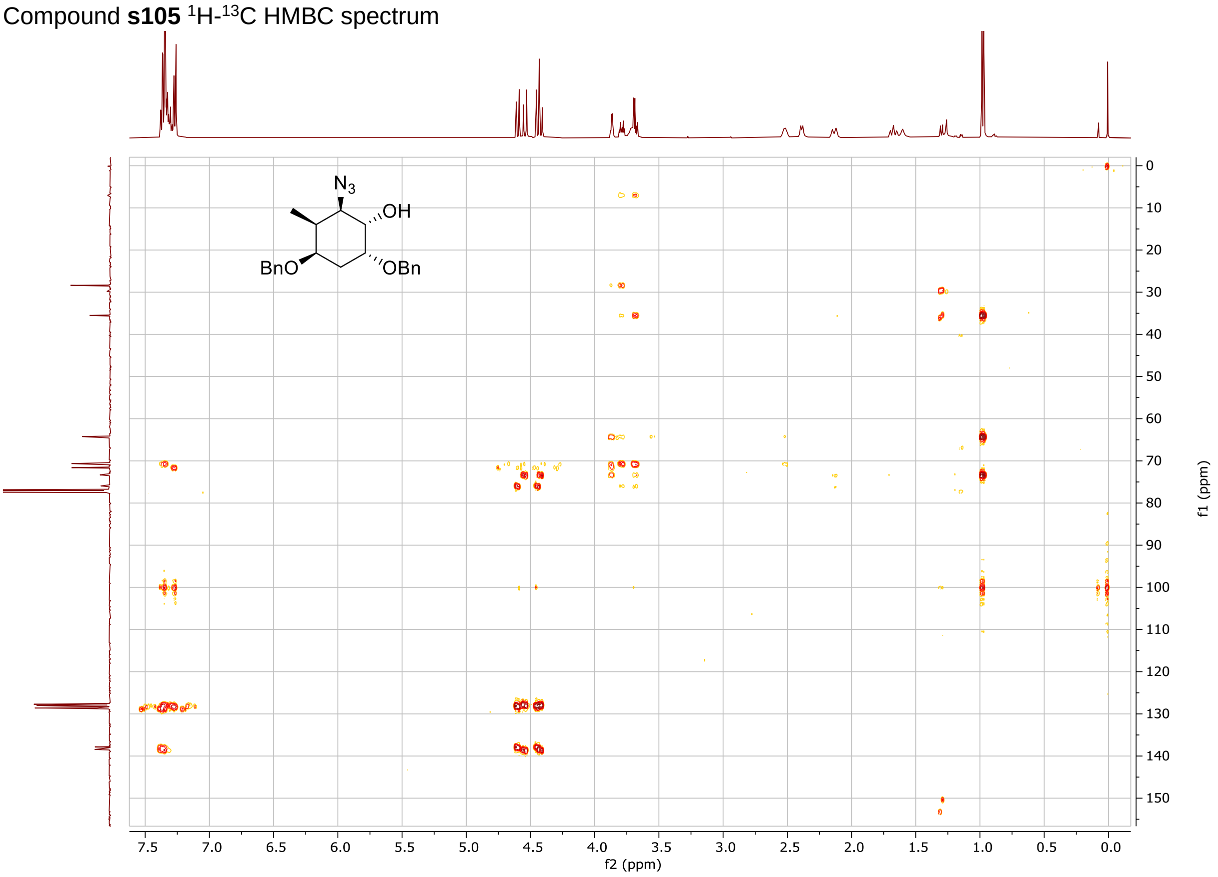

Compound **s106** ^{1}H NMR spectrum

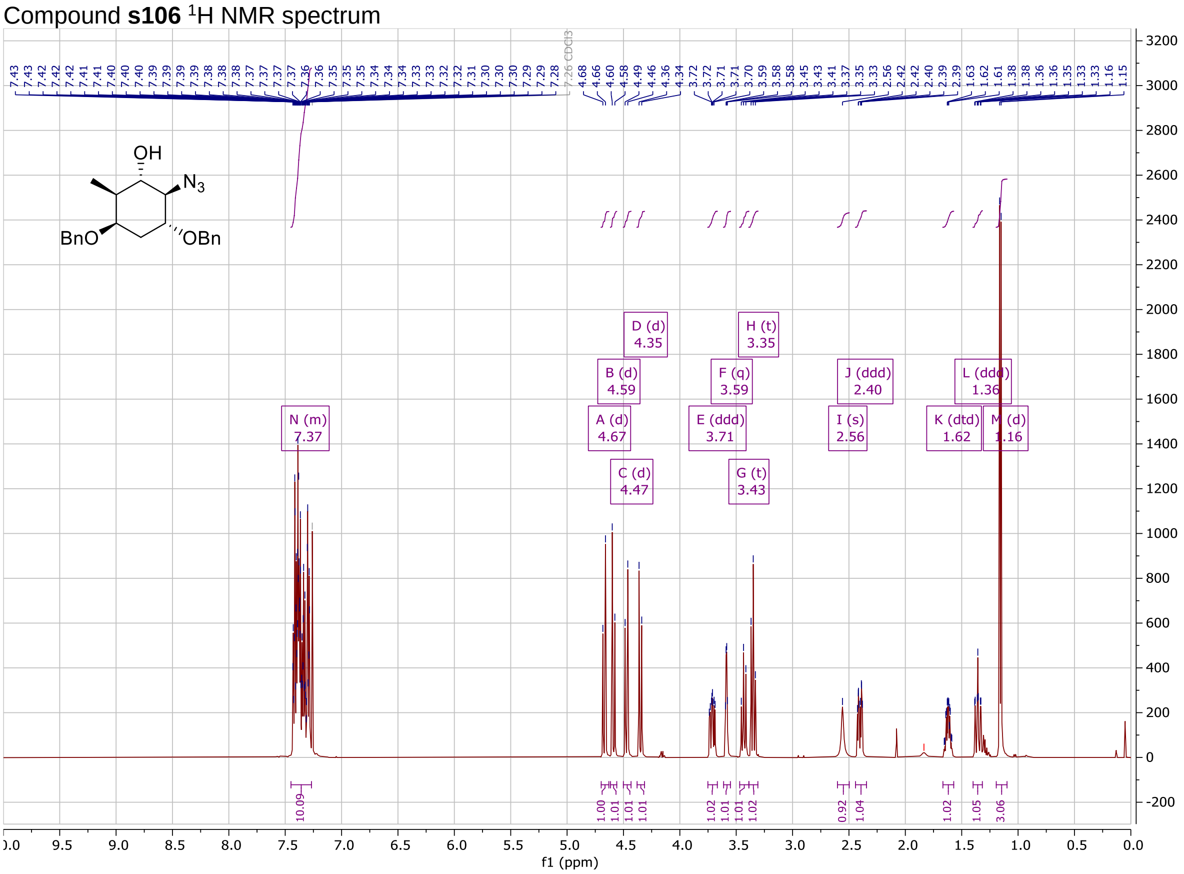

Compound **s106** ^{13}C NMR APT spectrum

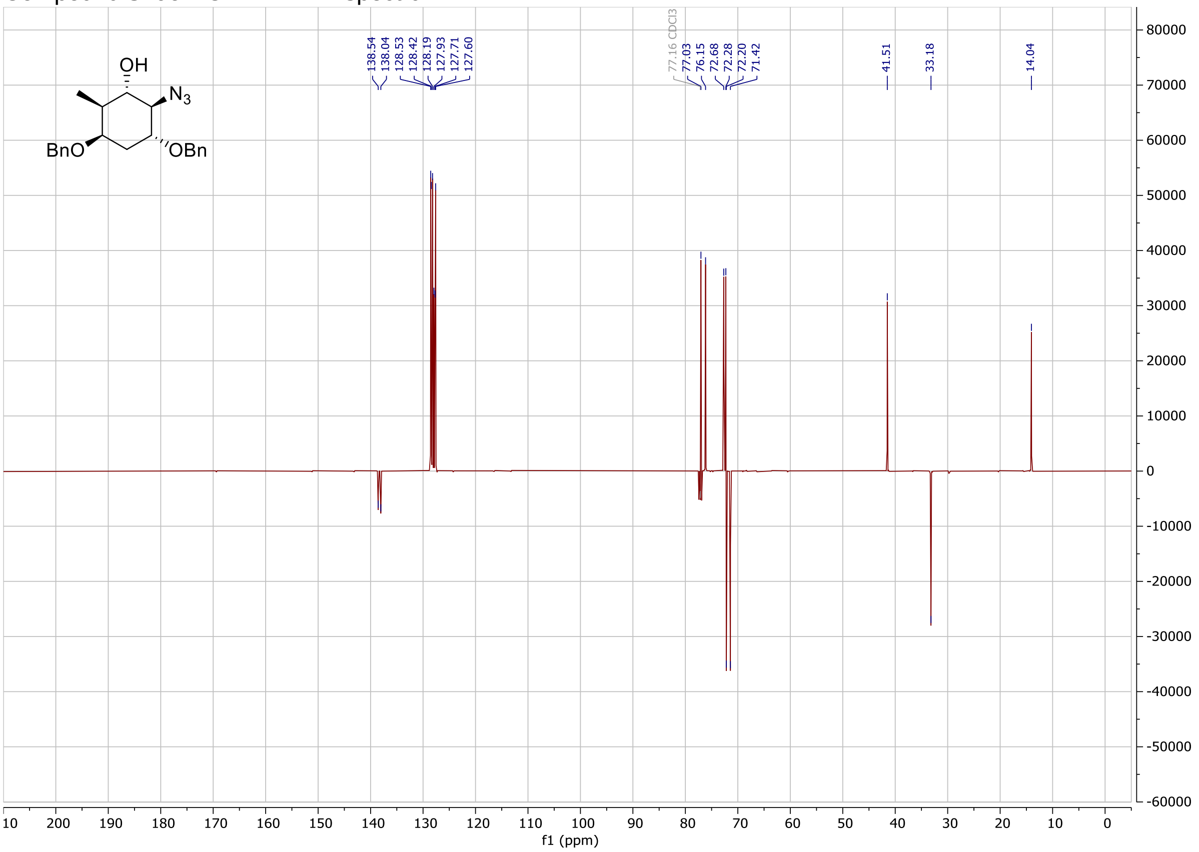

Compound **s106** ^{1}H-^{1}H COSY spectrum

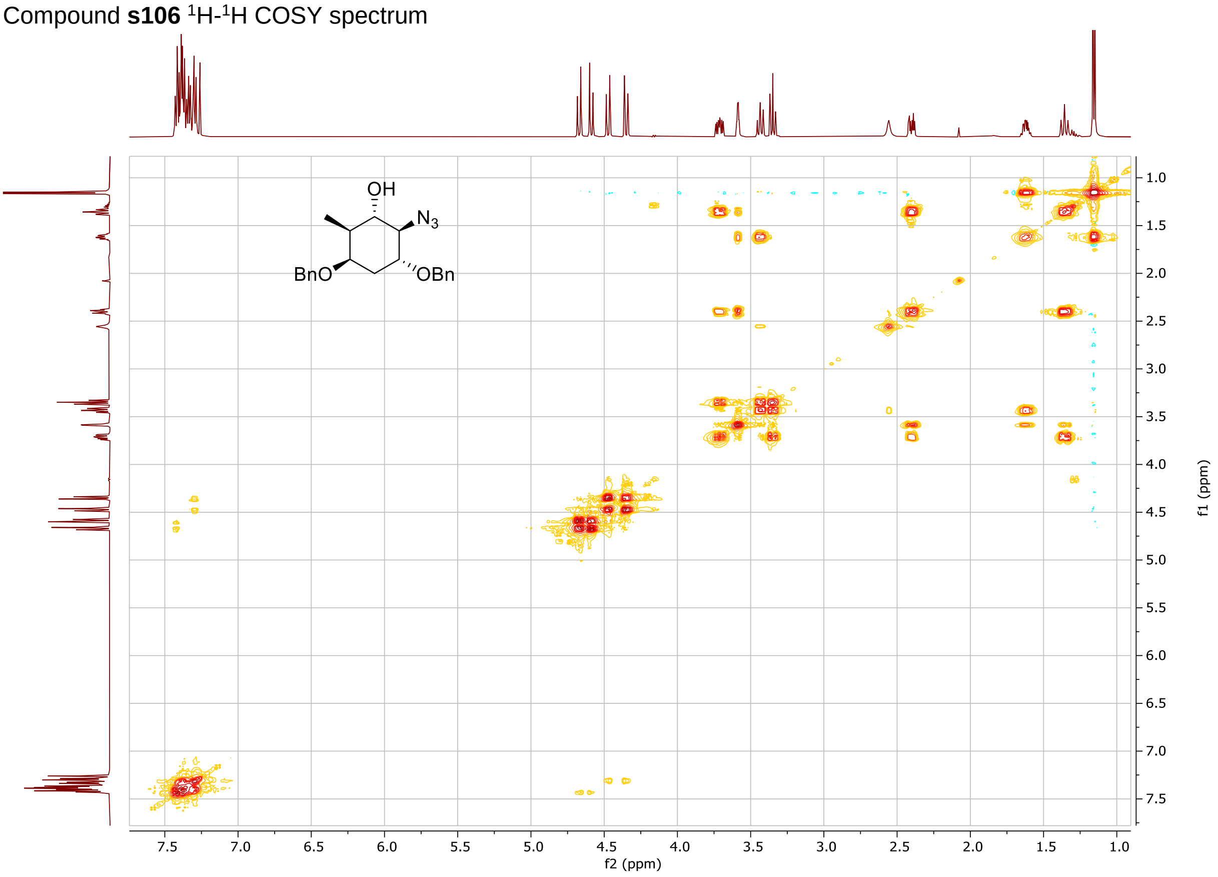

Compound **s106** ^{1}H-^{13}C HSQC spectrum

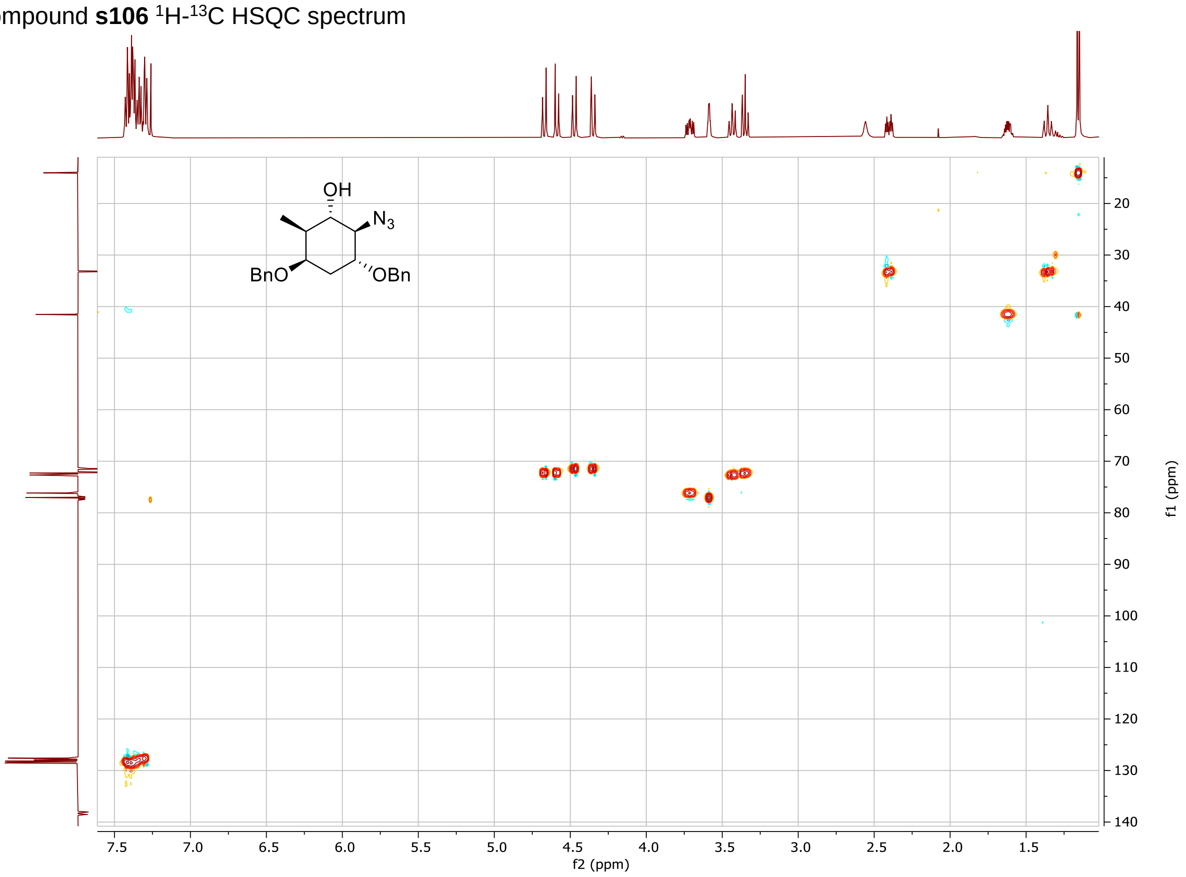

Compound **s106** ^{1}H-^{1}H NOESY spectrum

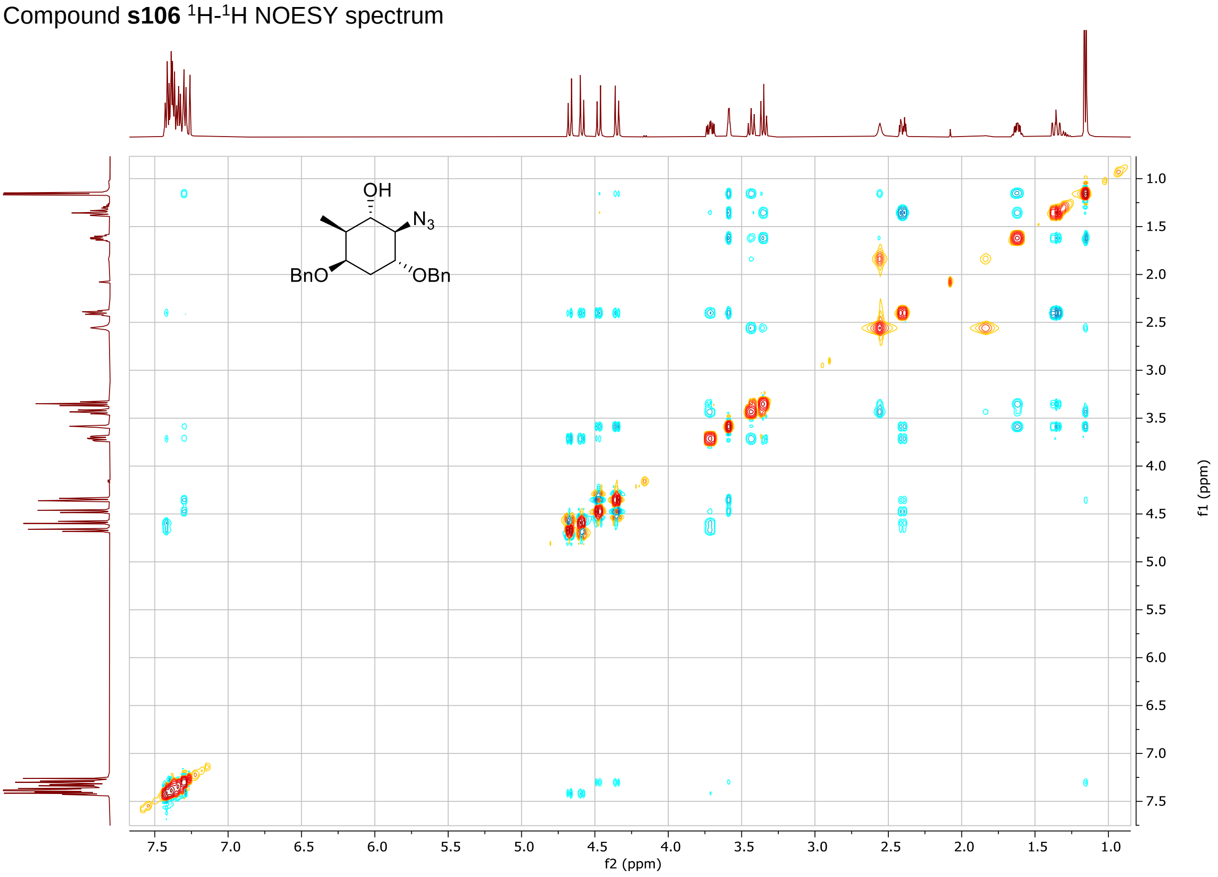

Compound **s106** ^{1}H-^{13}C HMBC spectrum

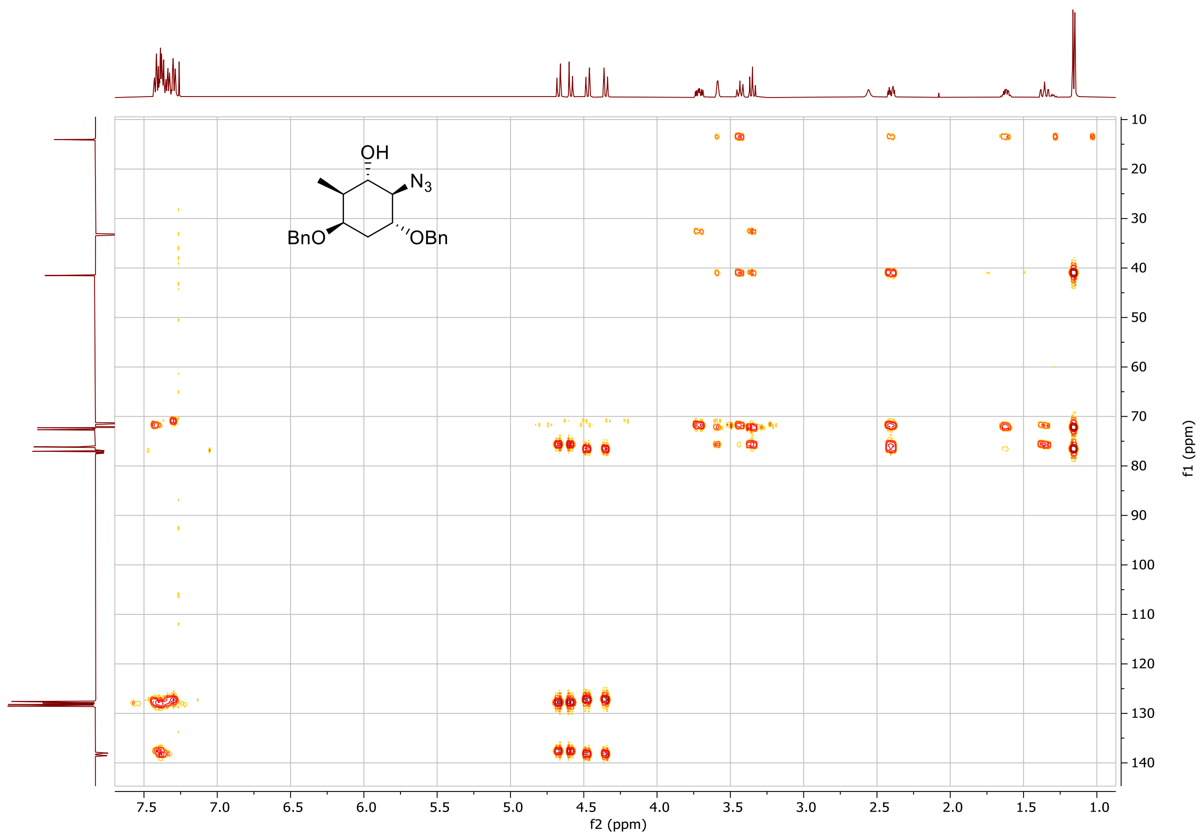

Compound **s107** ^{1}H NMR spectrum (323 K)

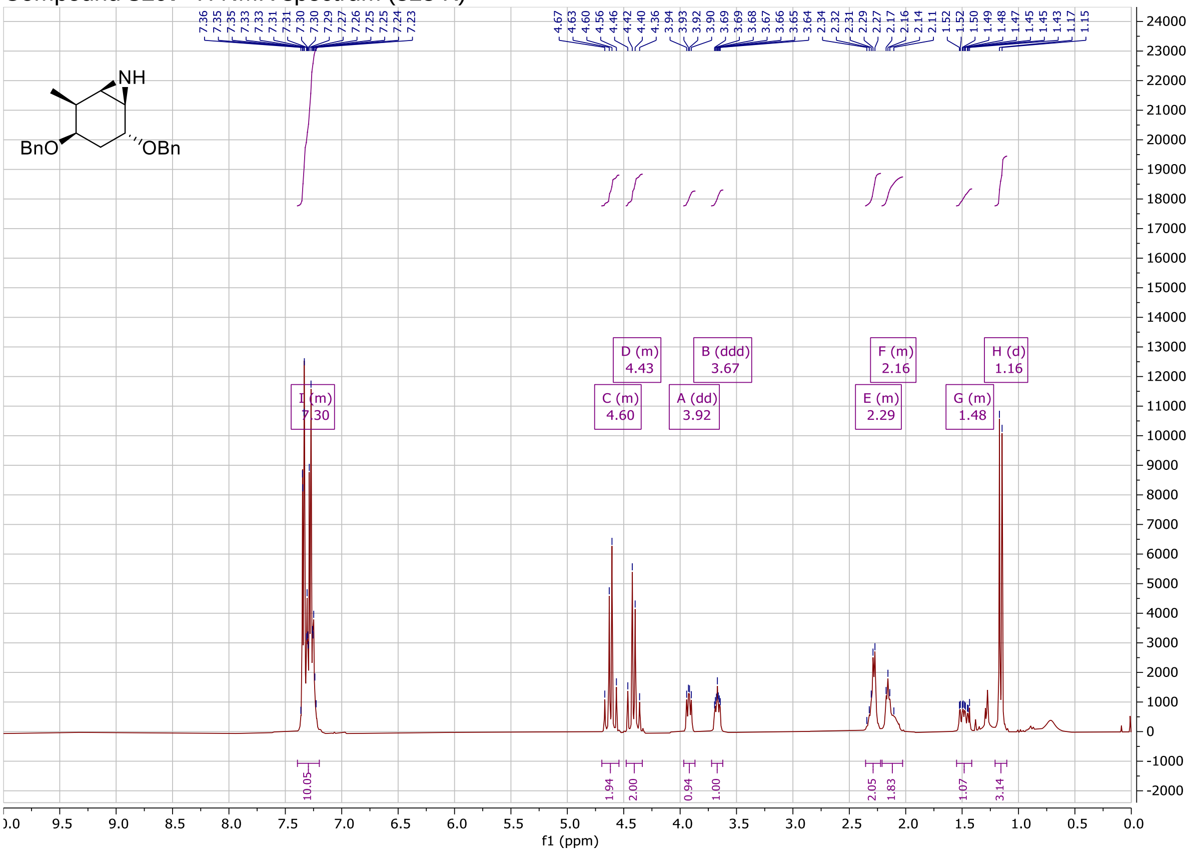

Compound **s107** ^{13}C NMR APT spectrum (323 K)

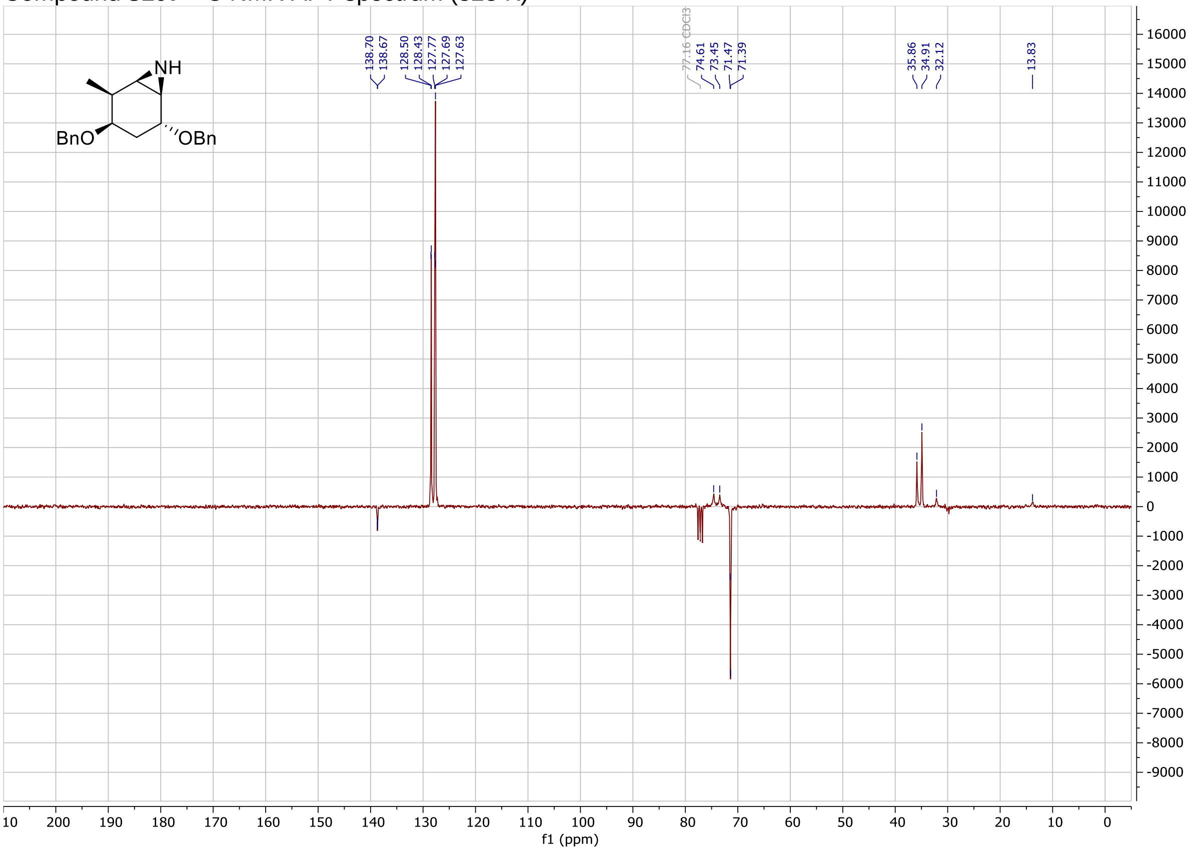

Compound **s107** ^{1}H-^{1}H COSY spectrum (323 K)

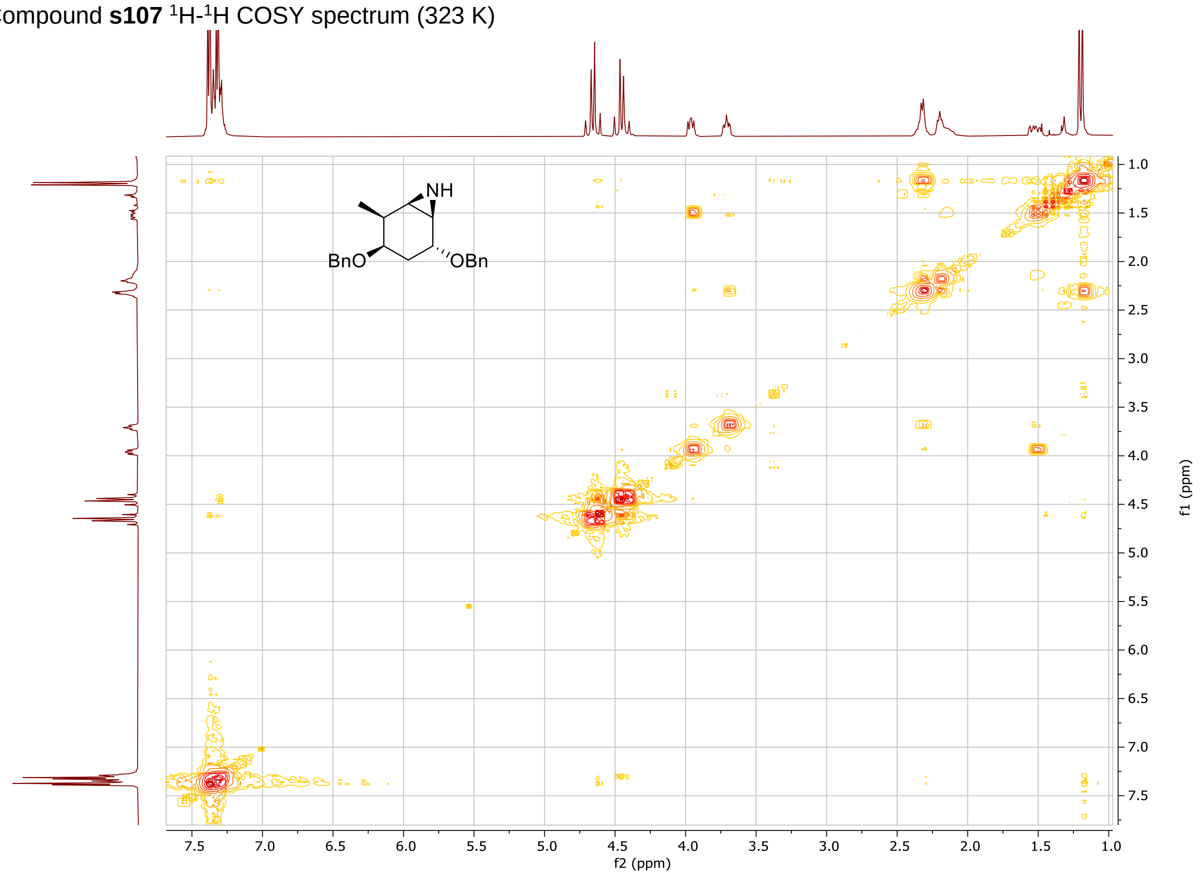

Compound **s107** ^{1}H-^{13}C HSQC spectrum (323 K)

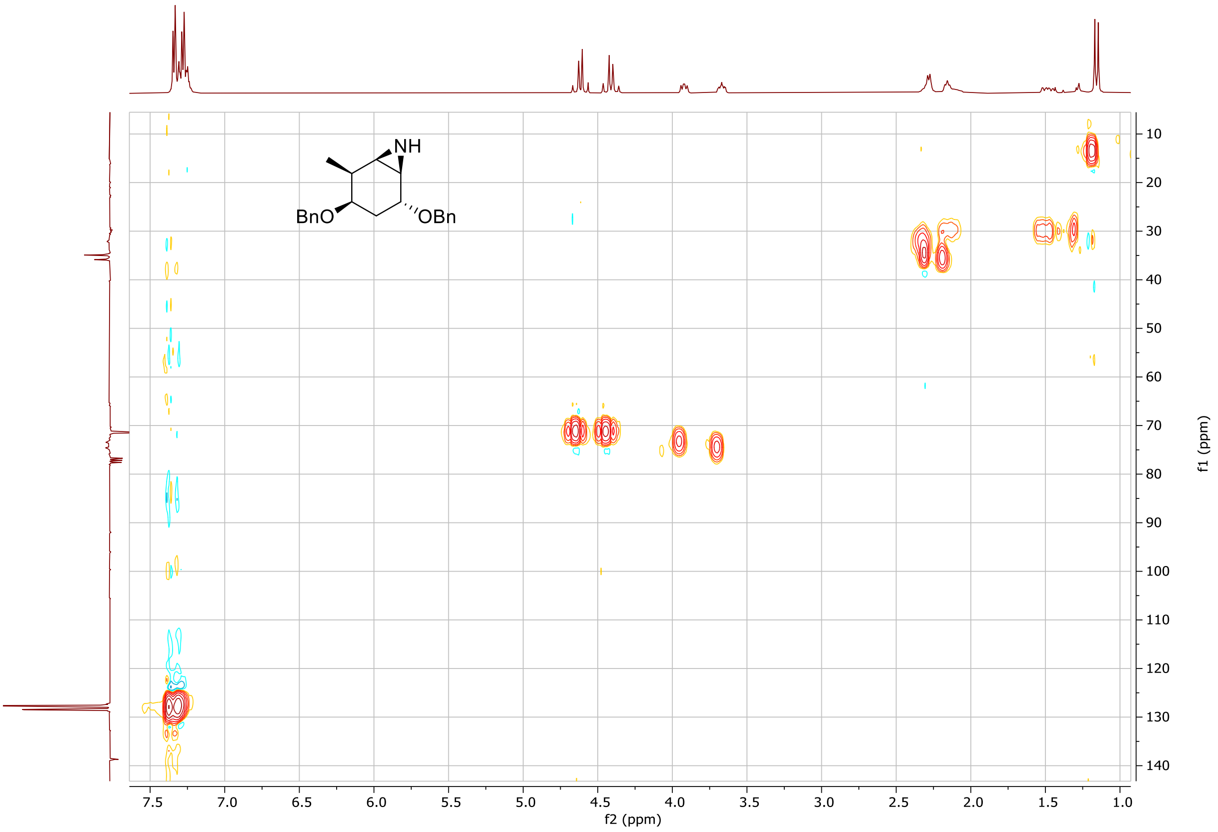

Compound **s108** ^{1}H NMR spectrum

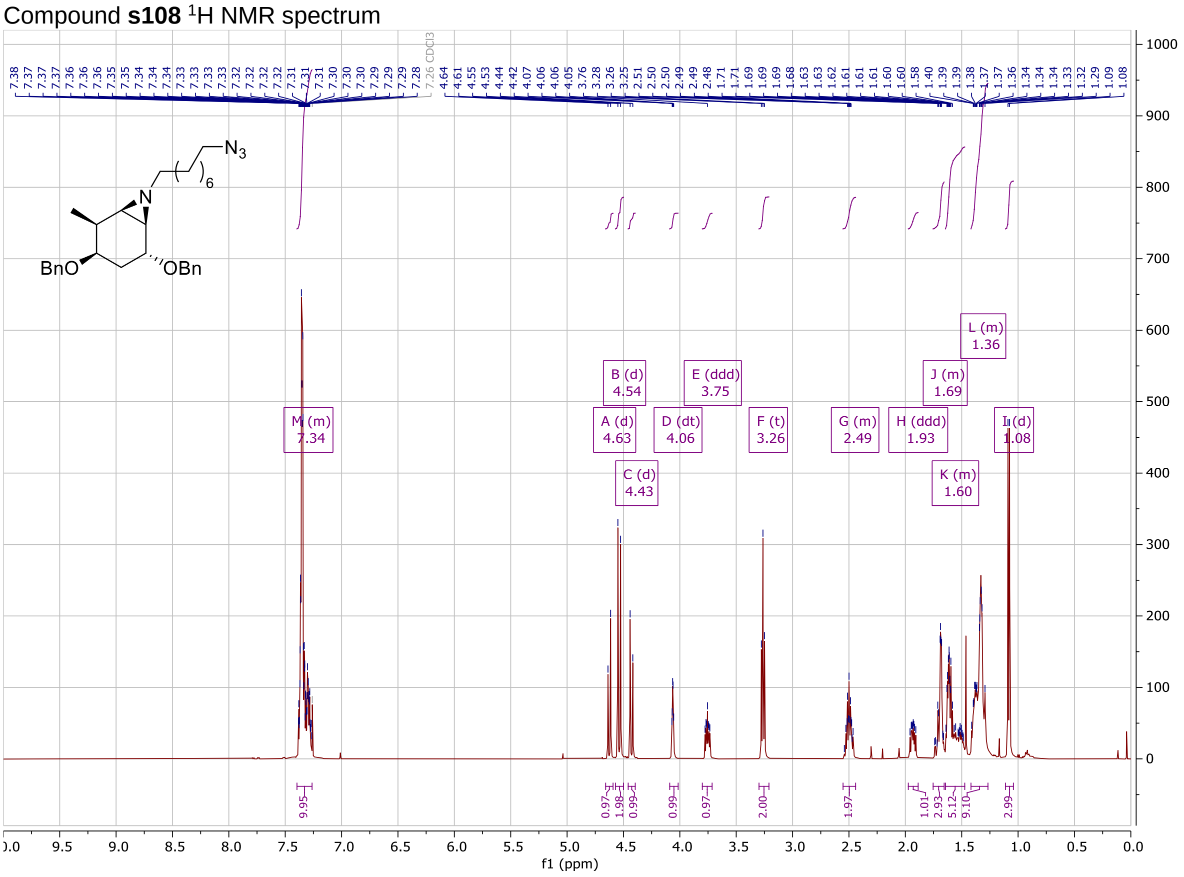

Compound **s108** ^{13}C NMR APT spectrum

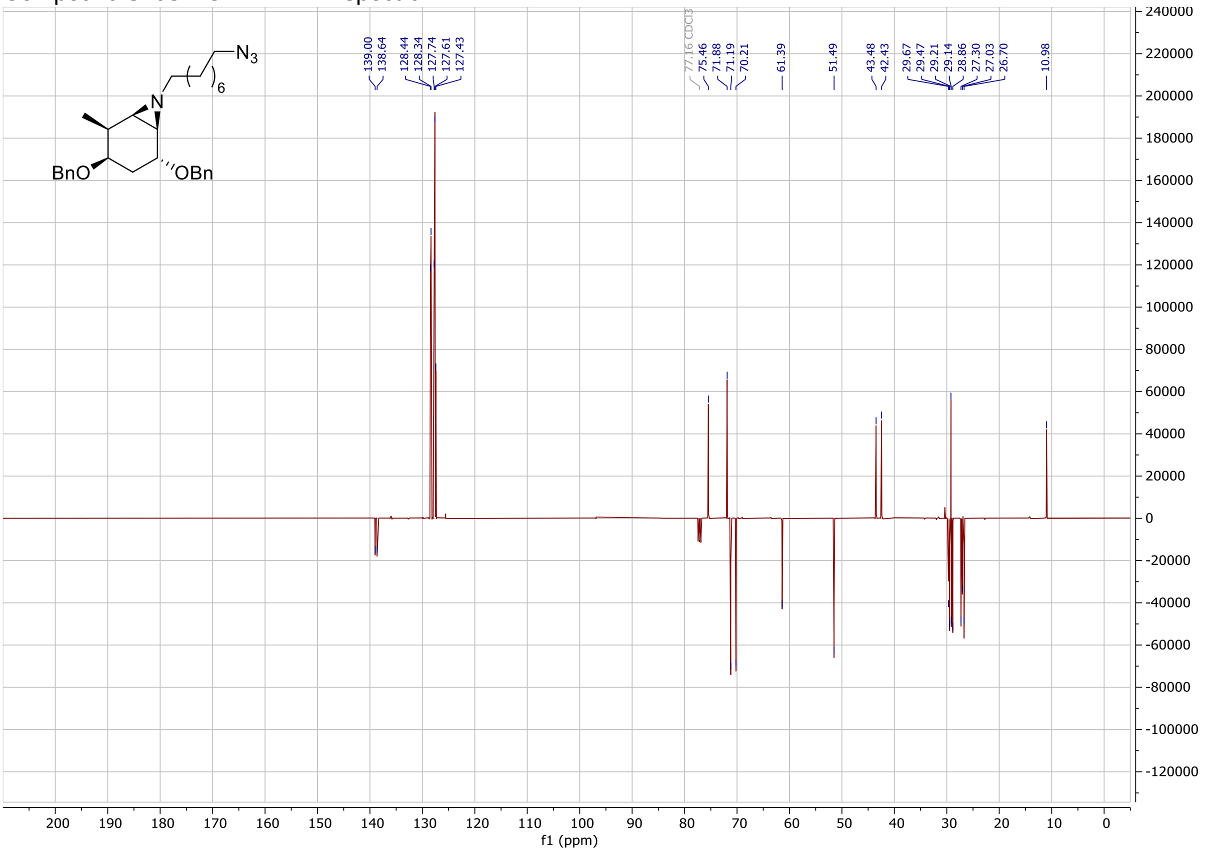

Compound **s108** ^{1}H-^{1}H COSY spectrum

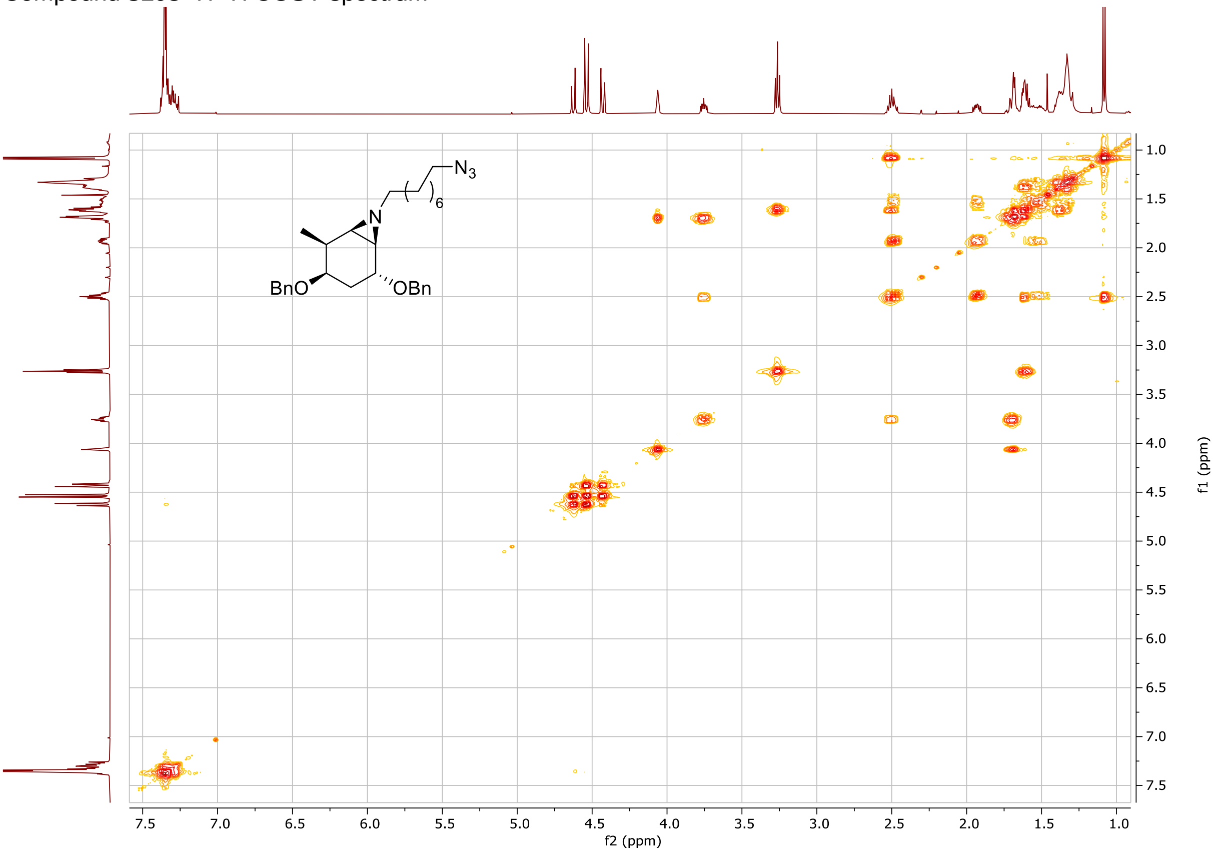

Compound **s108** ^{1}H-^{13}C HSQC spectrum

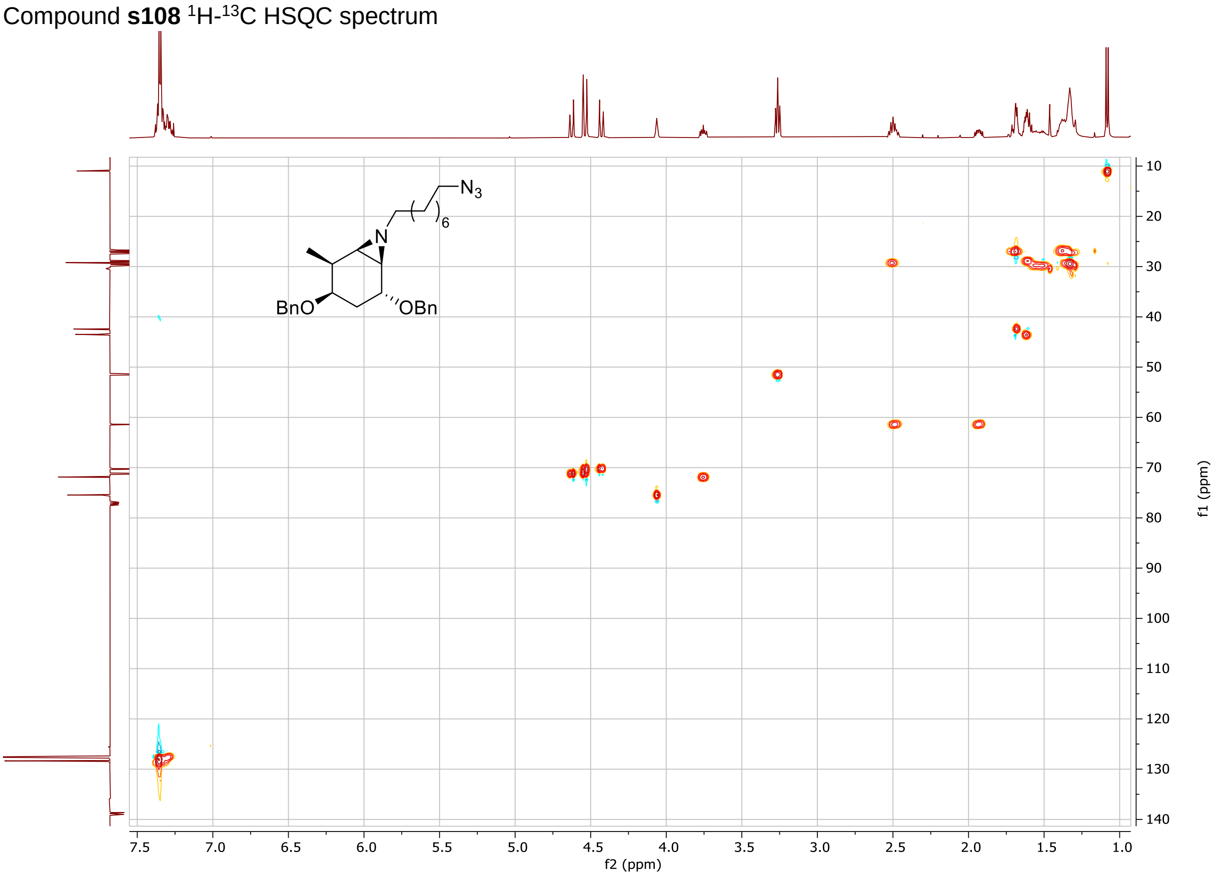

Compound **s108** ^{1}H-^{1}H NOESY spectrum

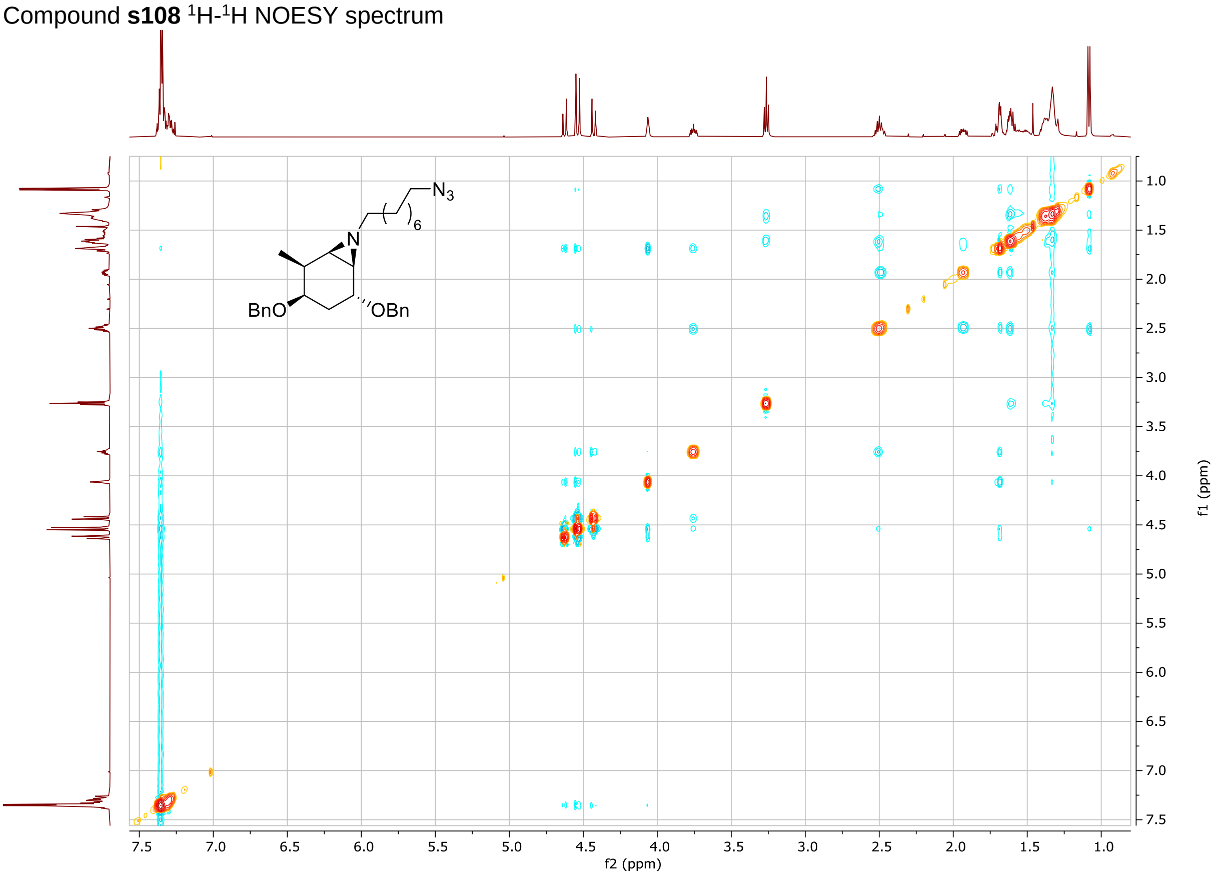

Compound **s108** ^{1}H-^{13}C HMBC spectrum

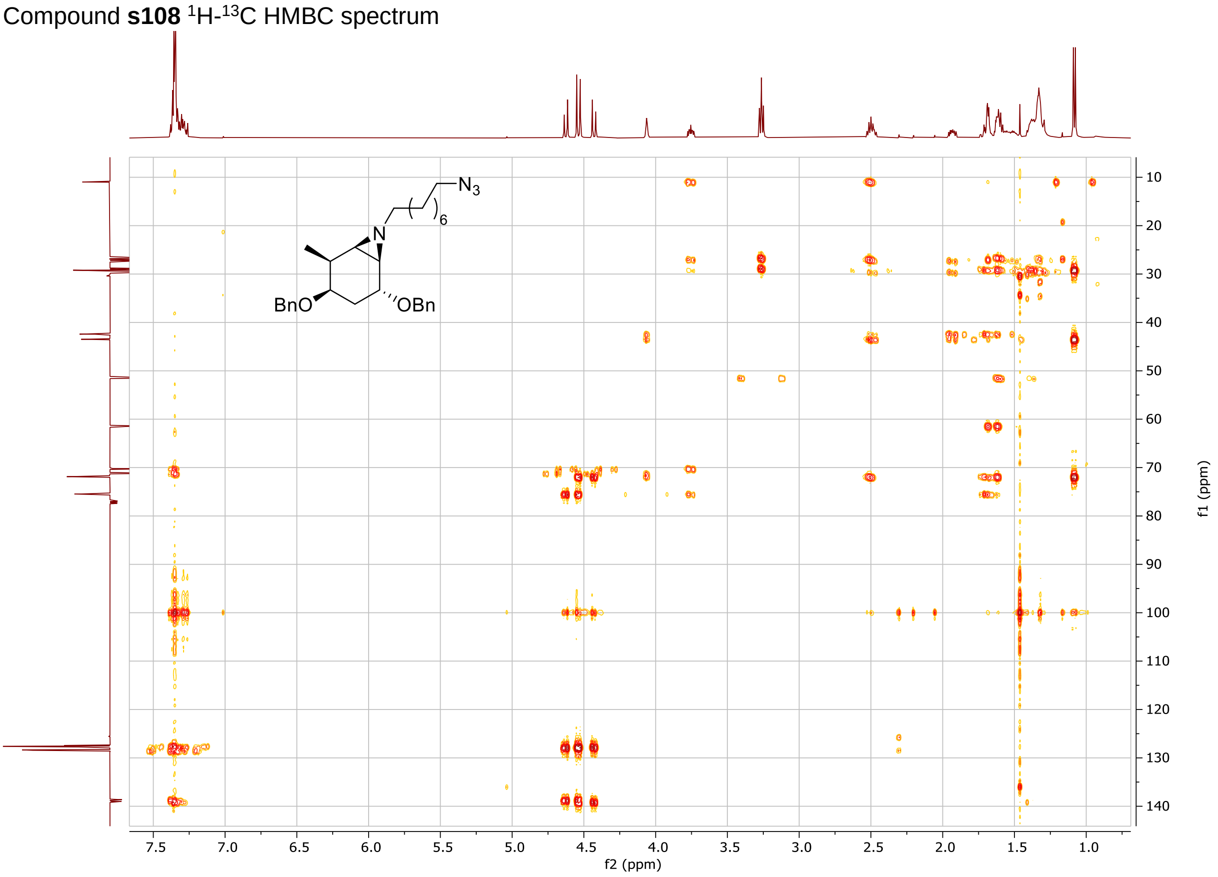

Compound **s109** ^{1}H NMR spectrum

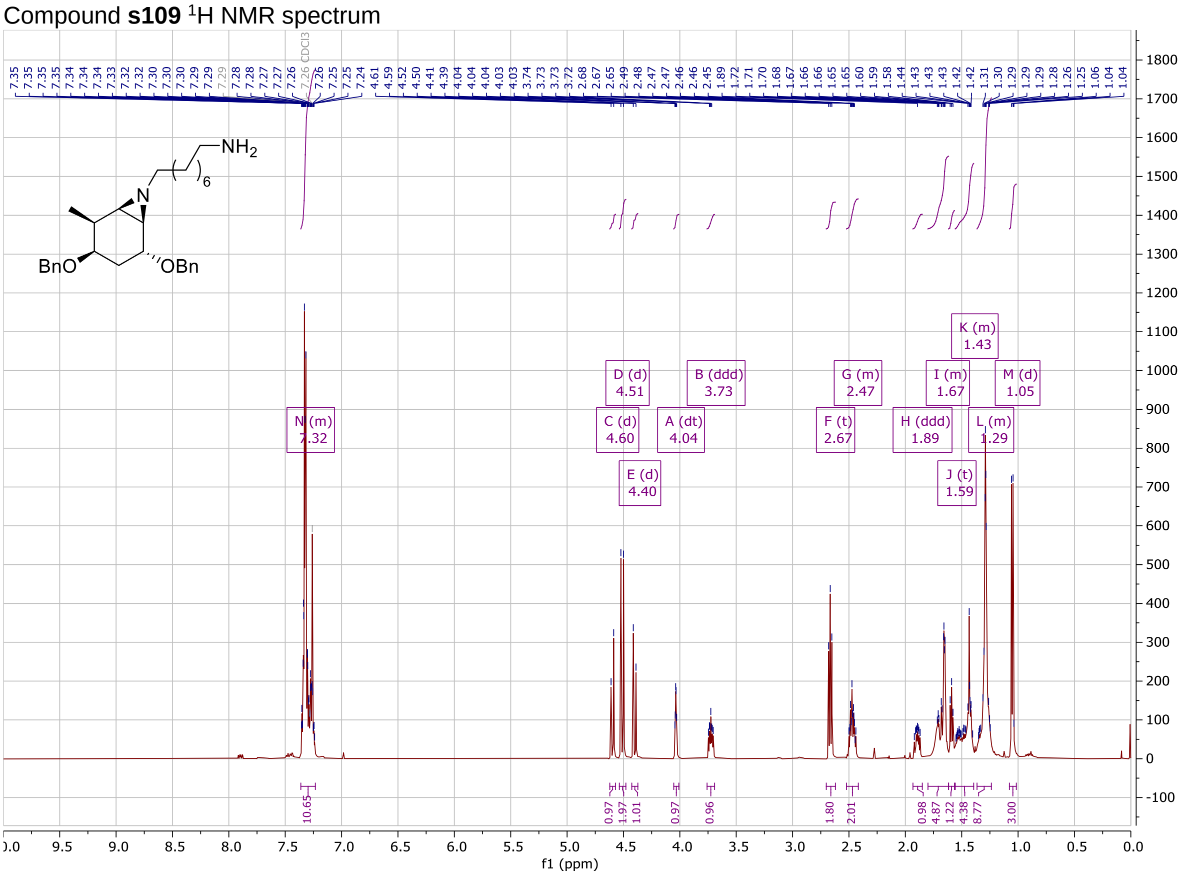

Compound **s109** ^{13}C NMR APT spectrum

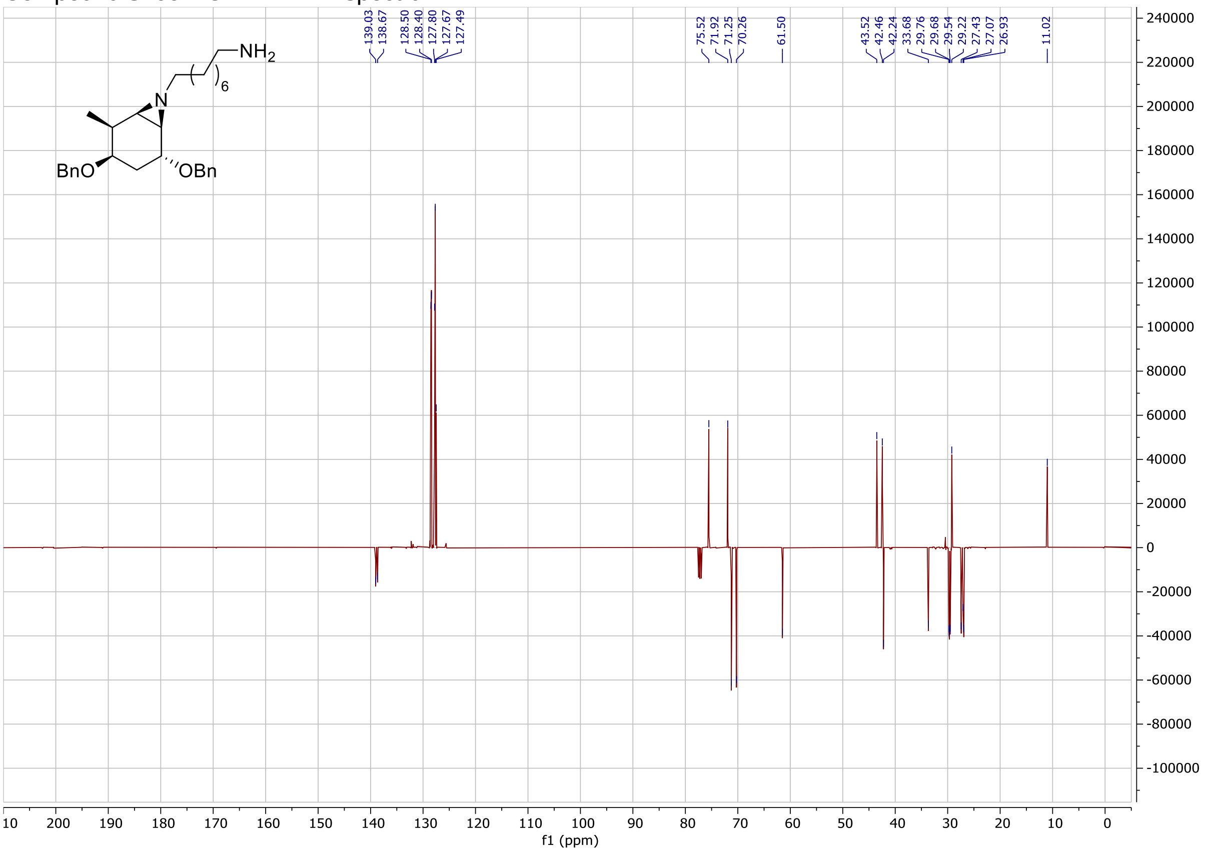

Compound **s109** ^{1}H-^{1}H COSY spectrum

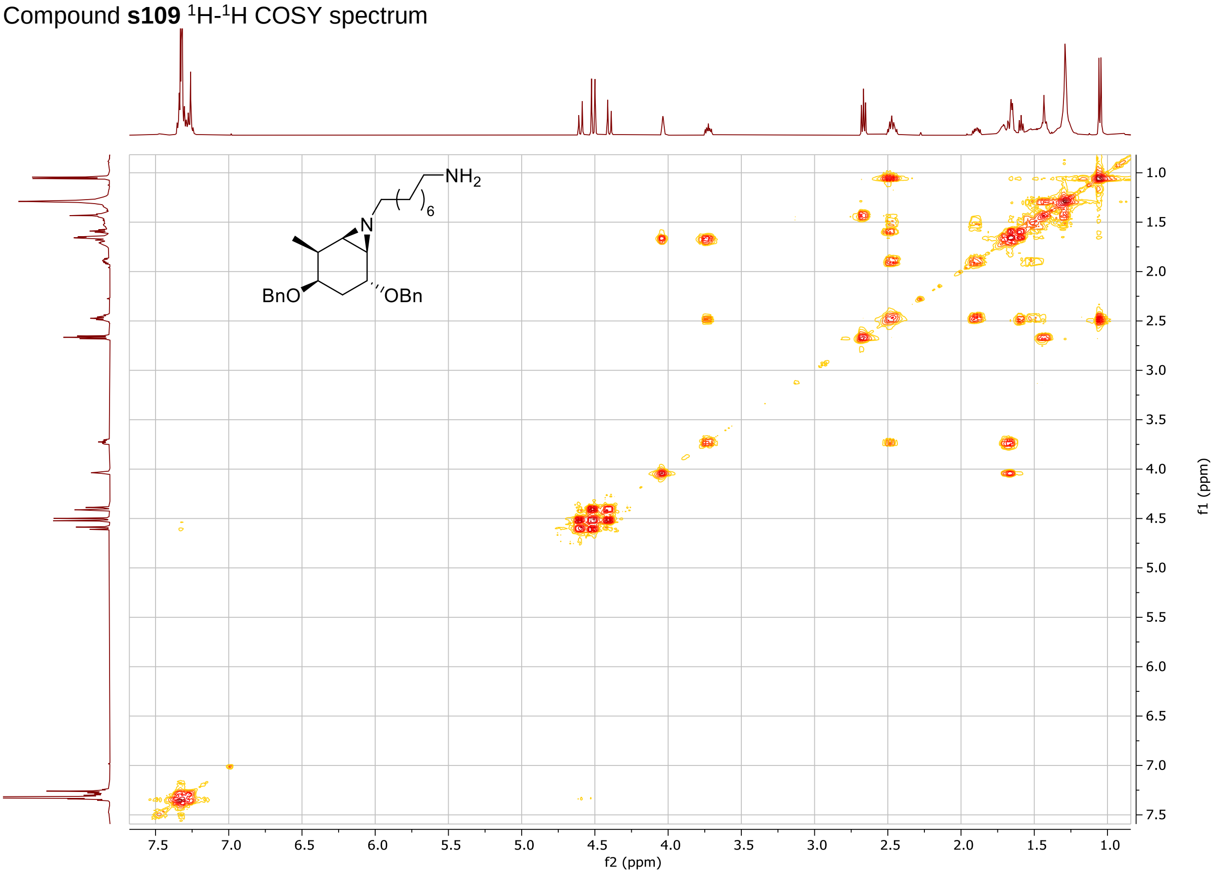

Compound **s109** ^{1}H-^{13}C HSQC spectrum

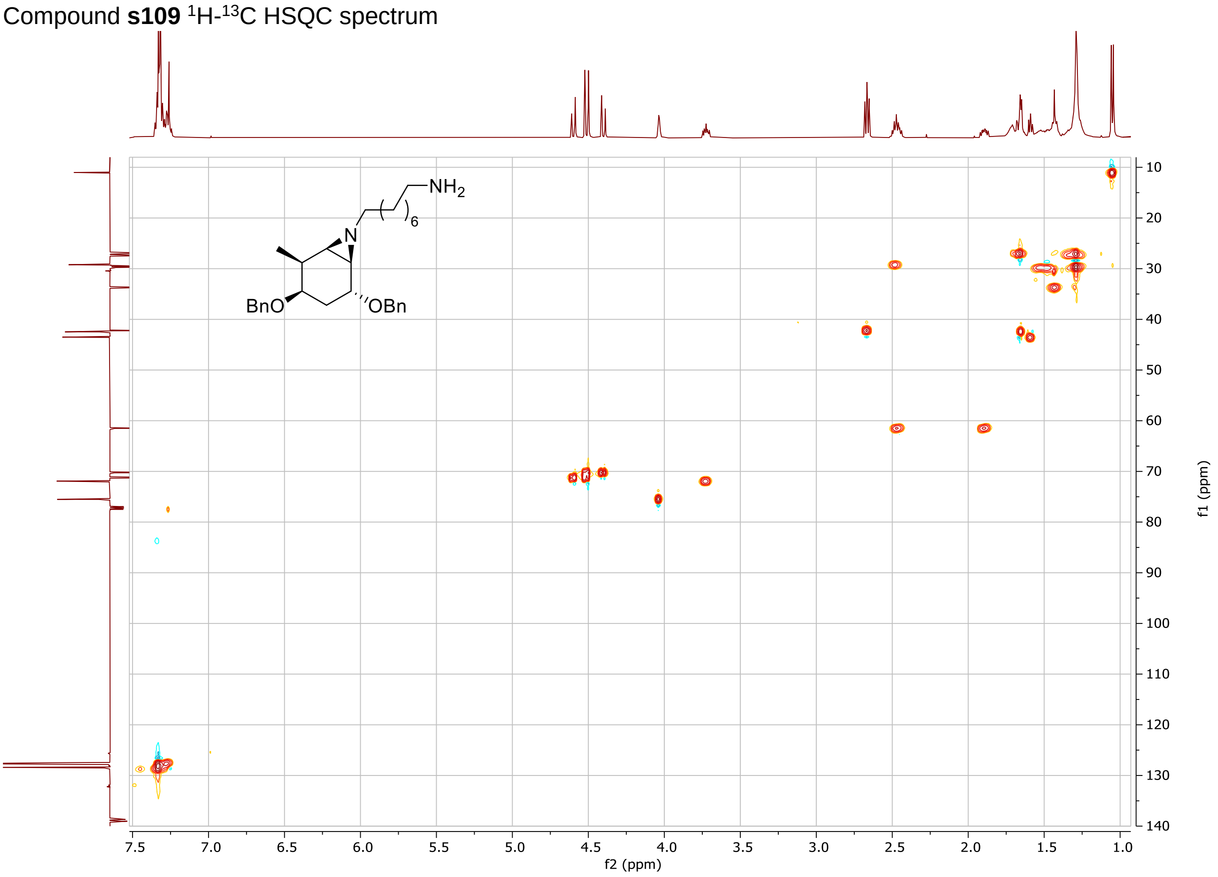

Compound **s110** ^{1}H NMR spectrum

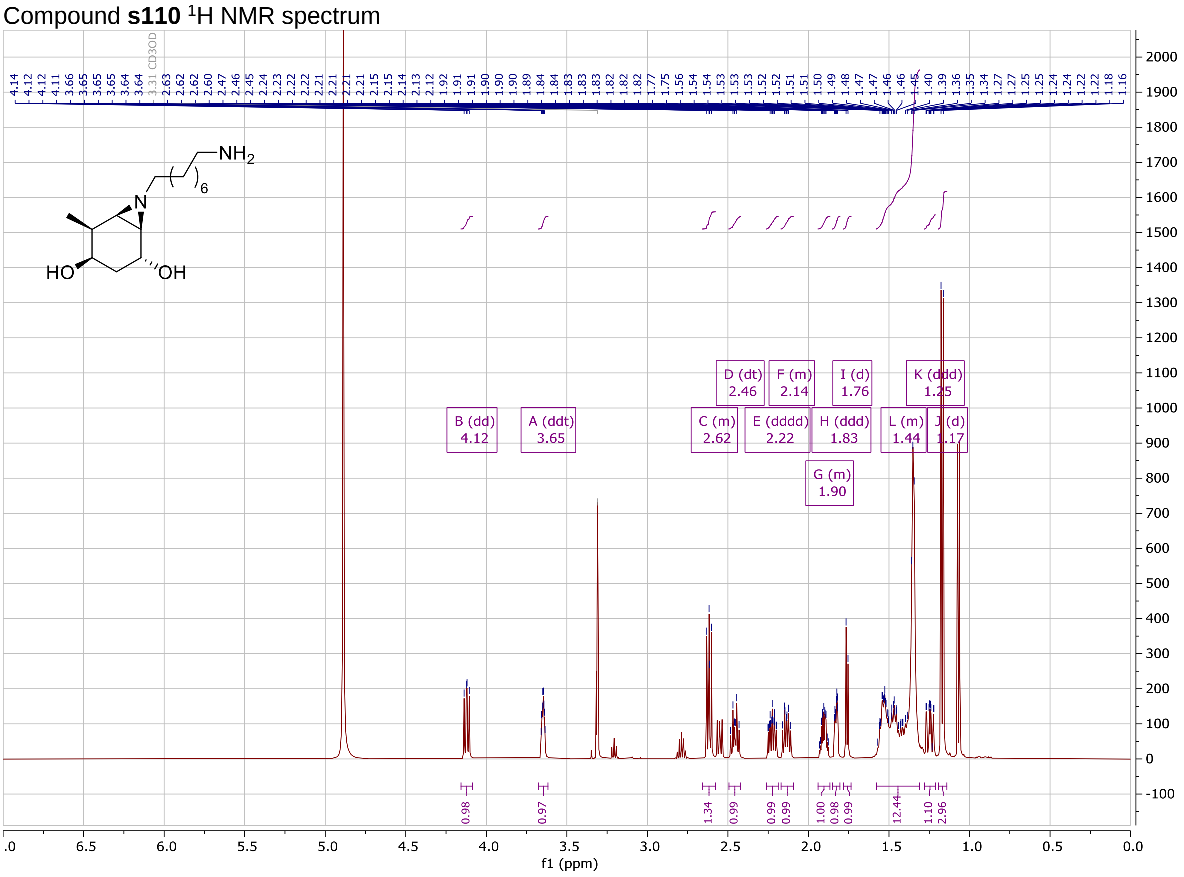

Compound **s110** ^{13}C NMR APT spectrum

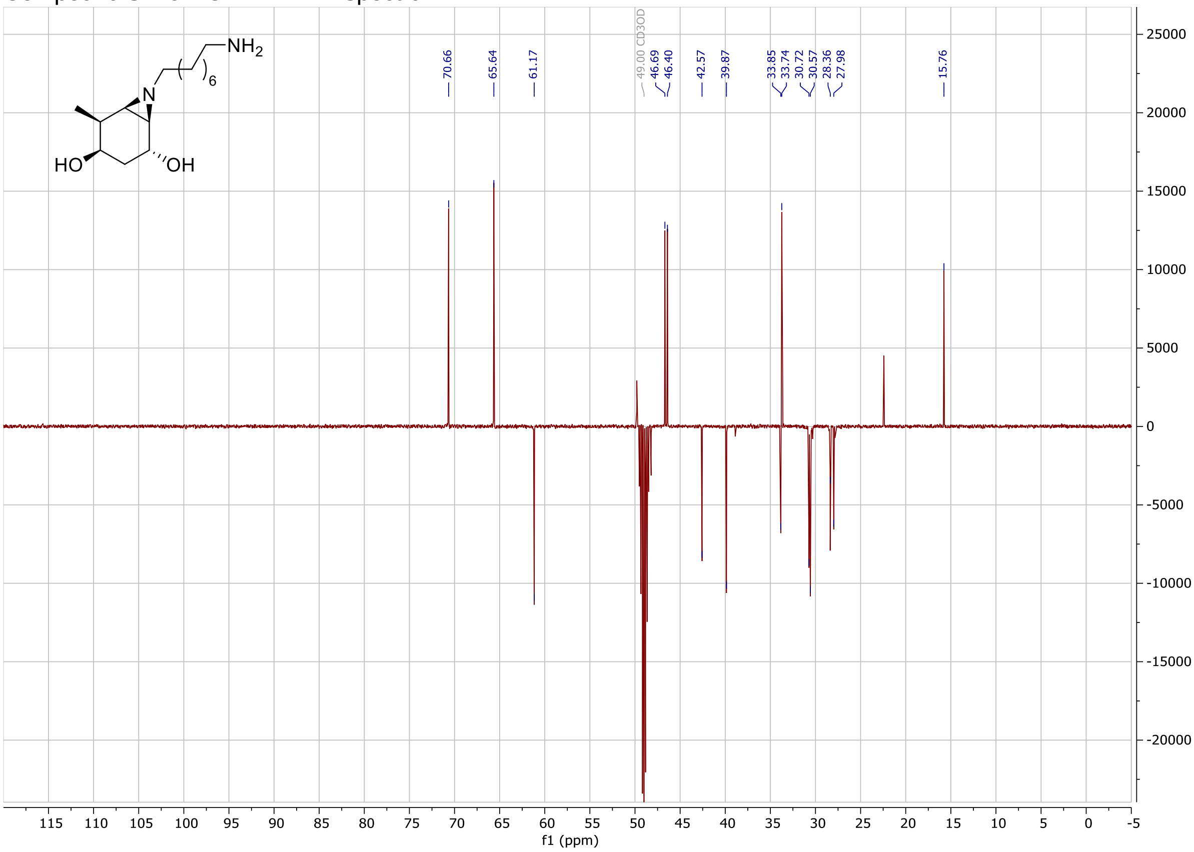

Compound **s110** ^{1}H-^{1}H COSY spectrum

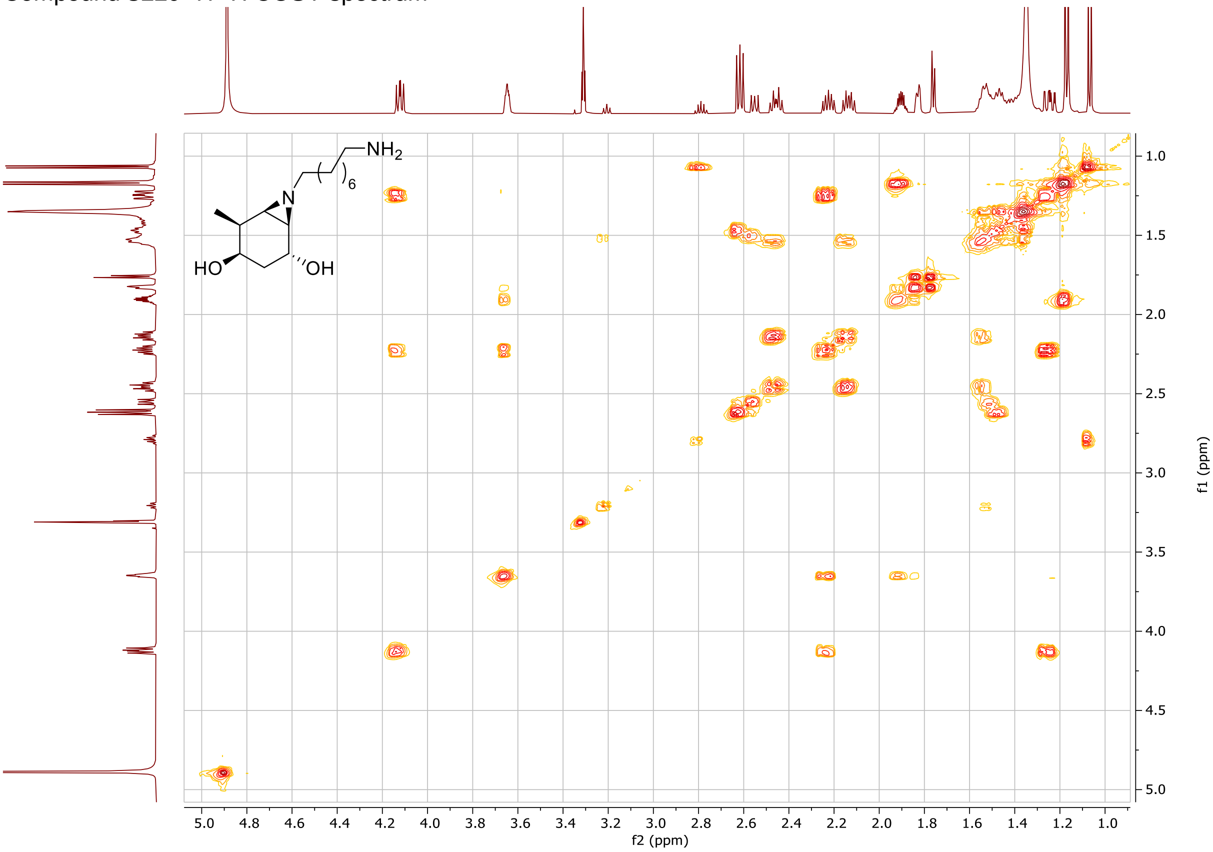

Compound **s110** ^{1}H-^{13}C HSQC spectrum

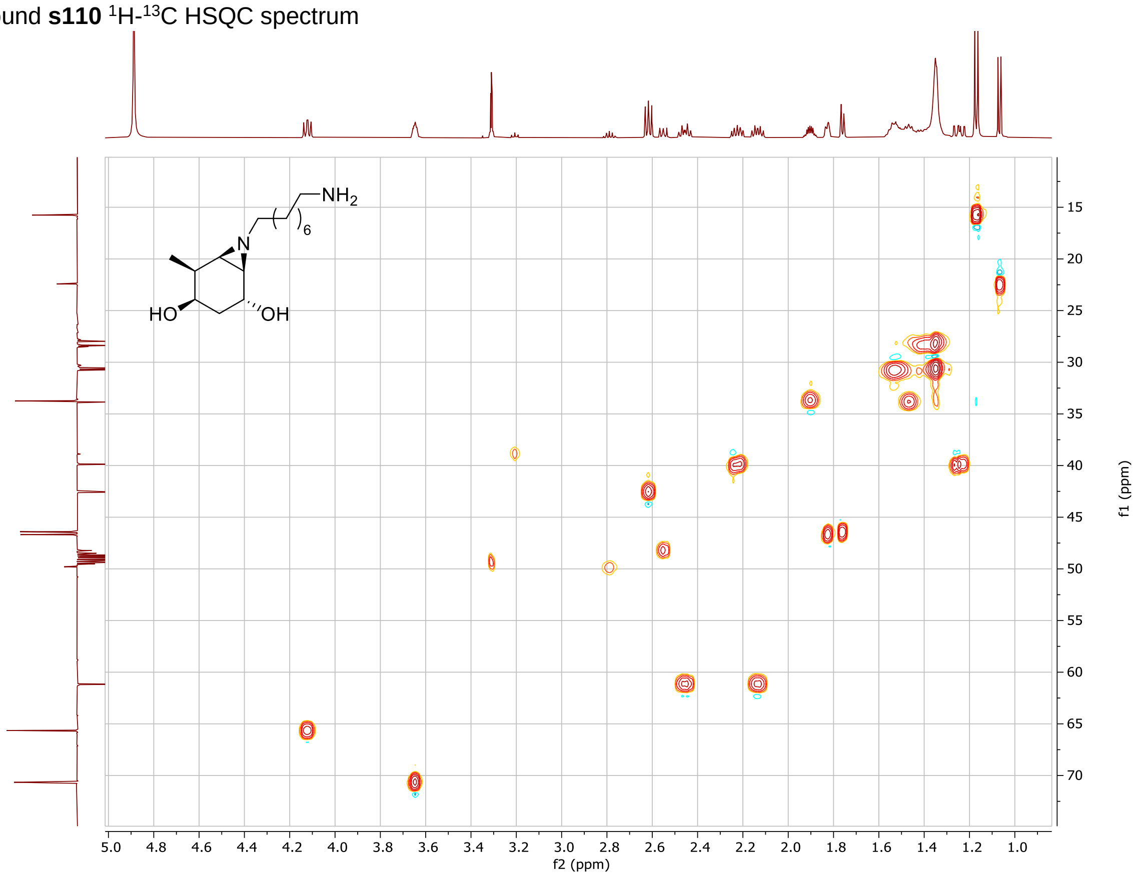

Compound **50** ^{1}H NMR spectrum

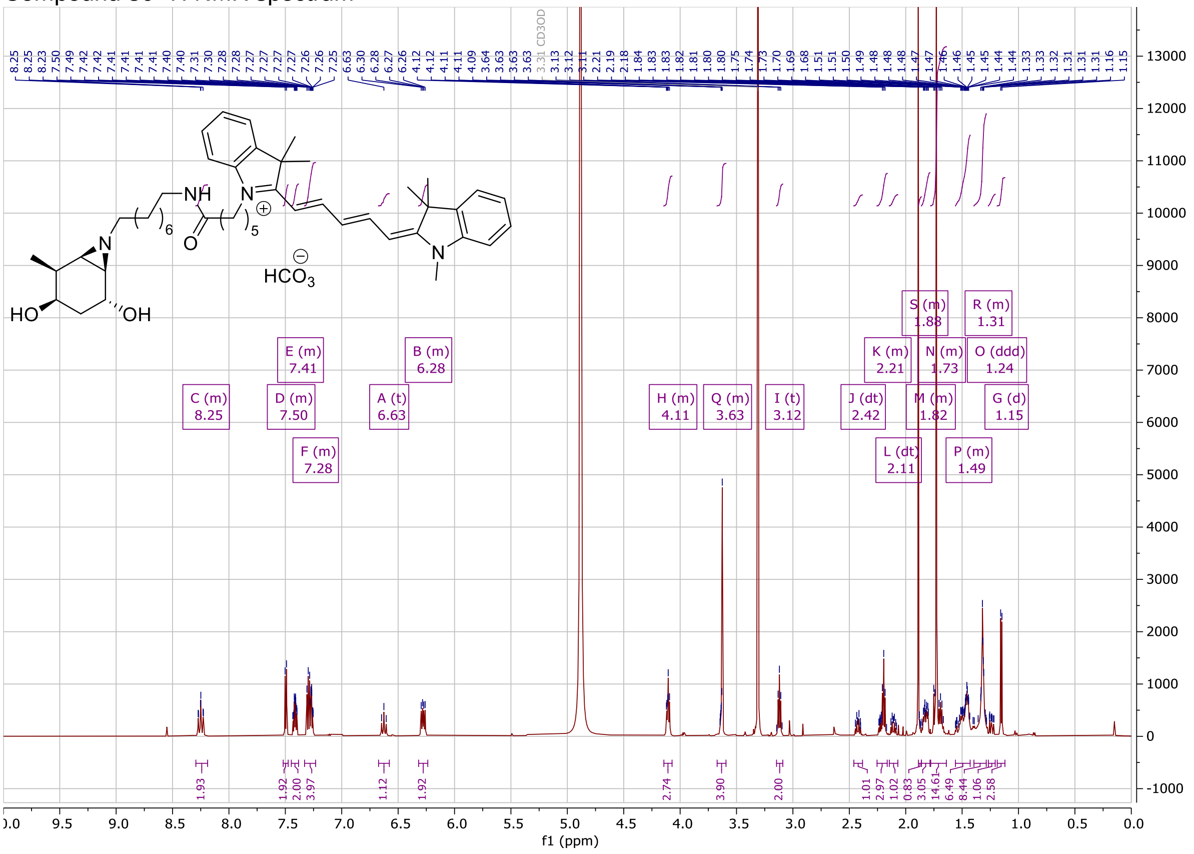

Compound **50** ^{13}C NMR APT spectrum

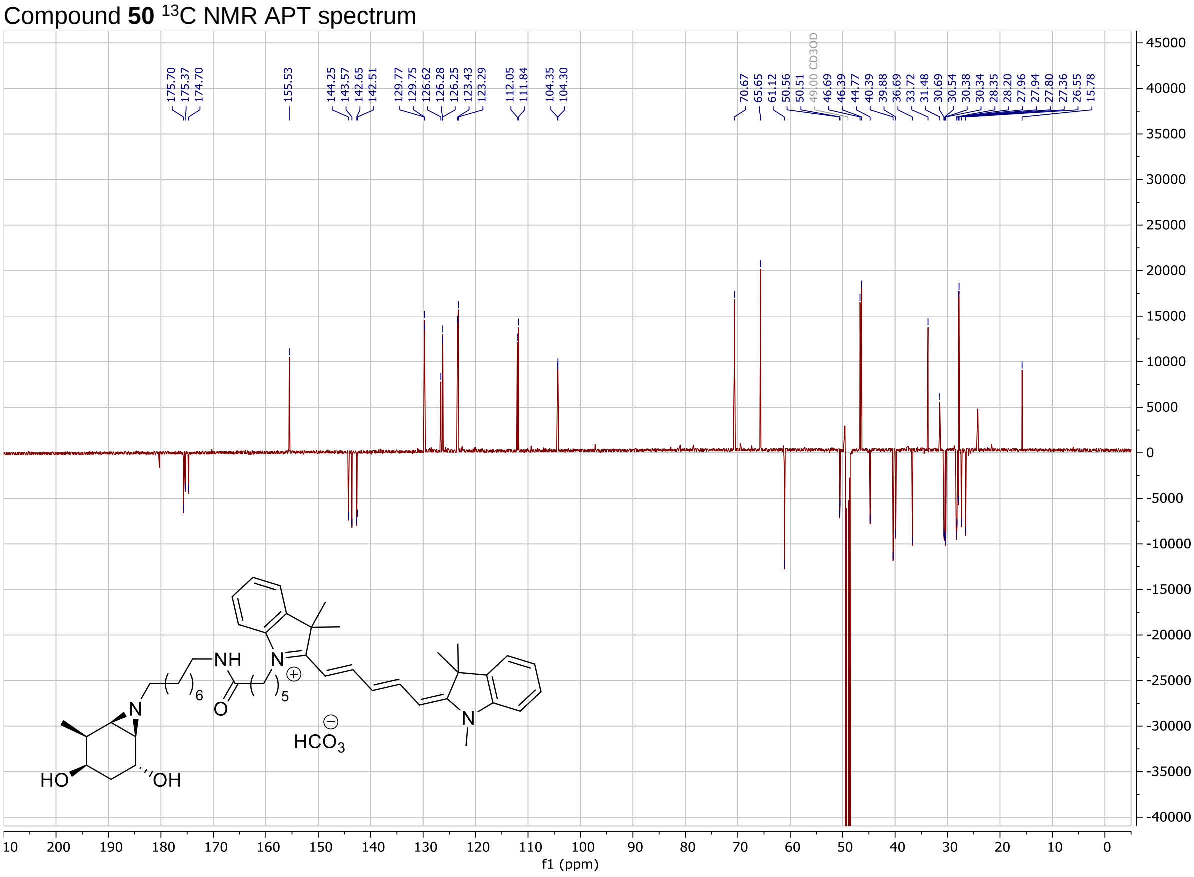

Compound **50** ^{1}H-^{1}H COSY spectrum

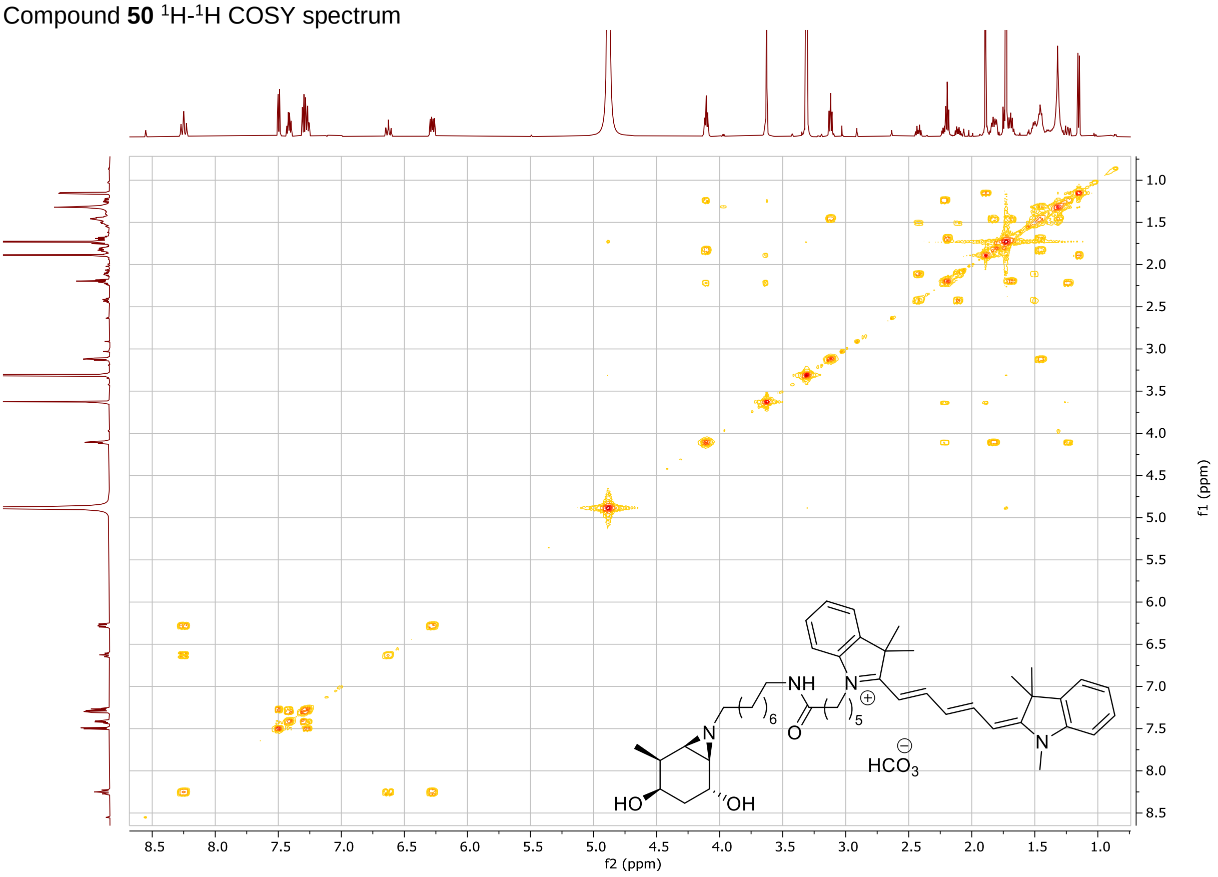

Compound **50** ^{1}H-^{13}C HSQC spectrum

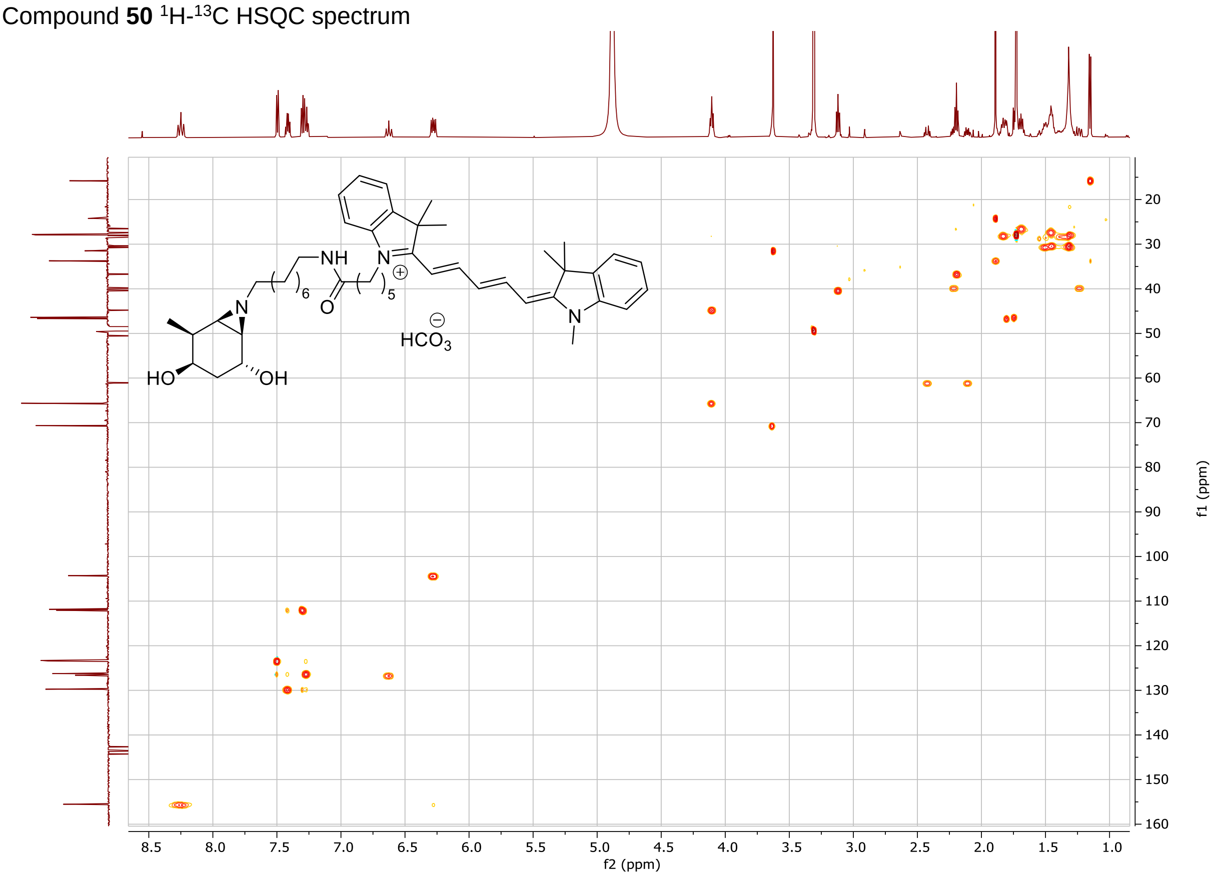

Compound **57** ^{1}H NMR spectrum

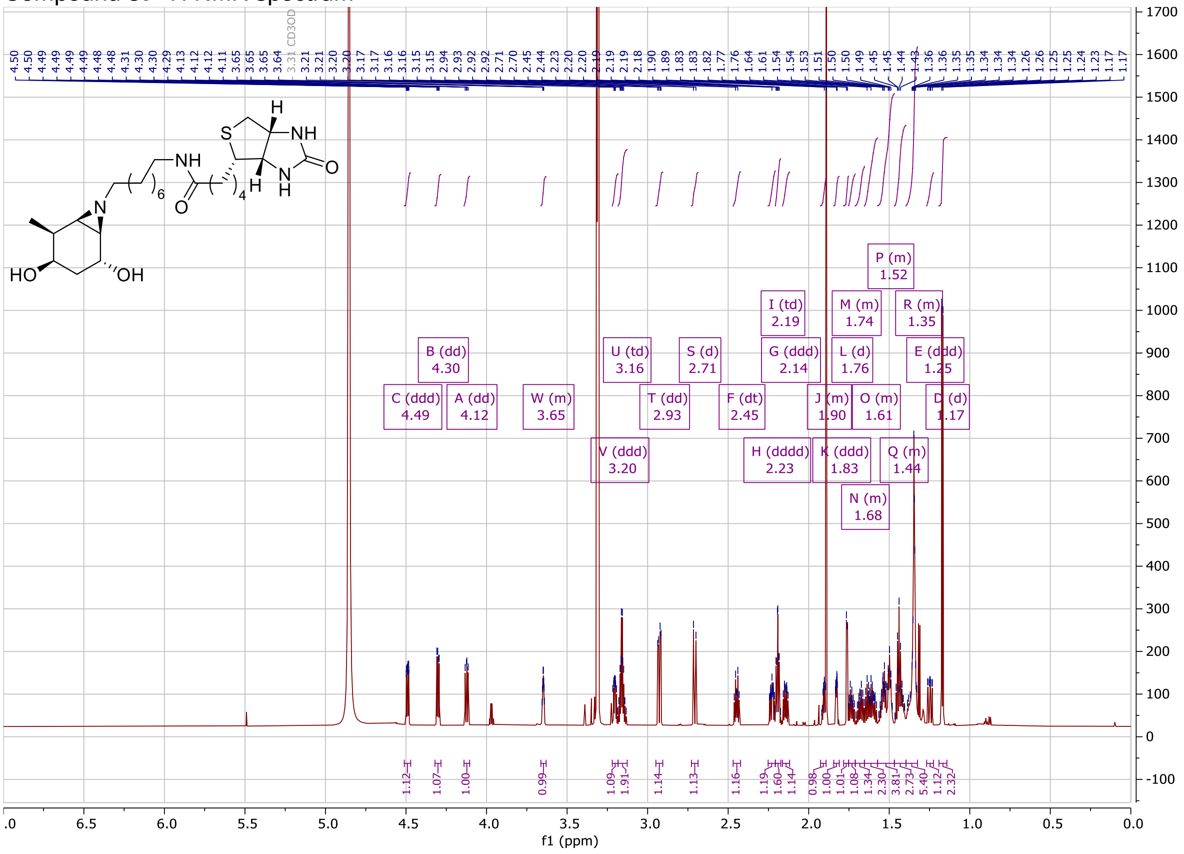

Compound **57** ^{13}C NMR APT spectrum

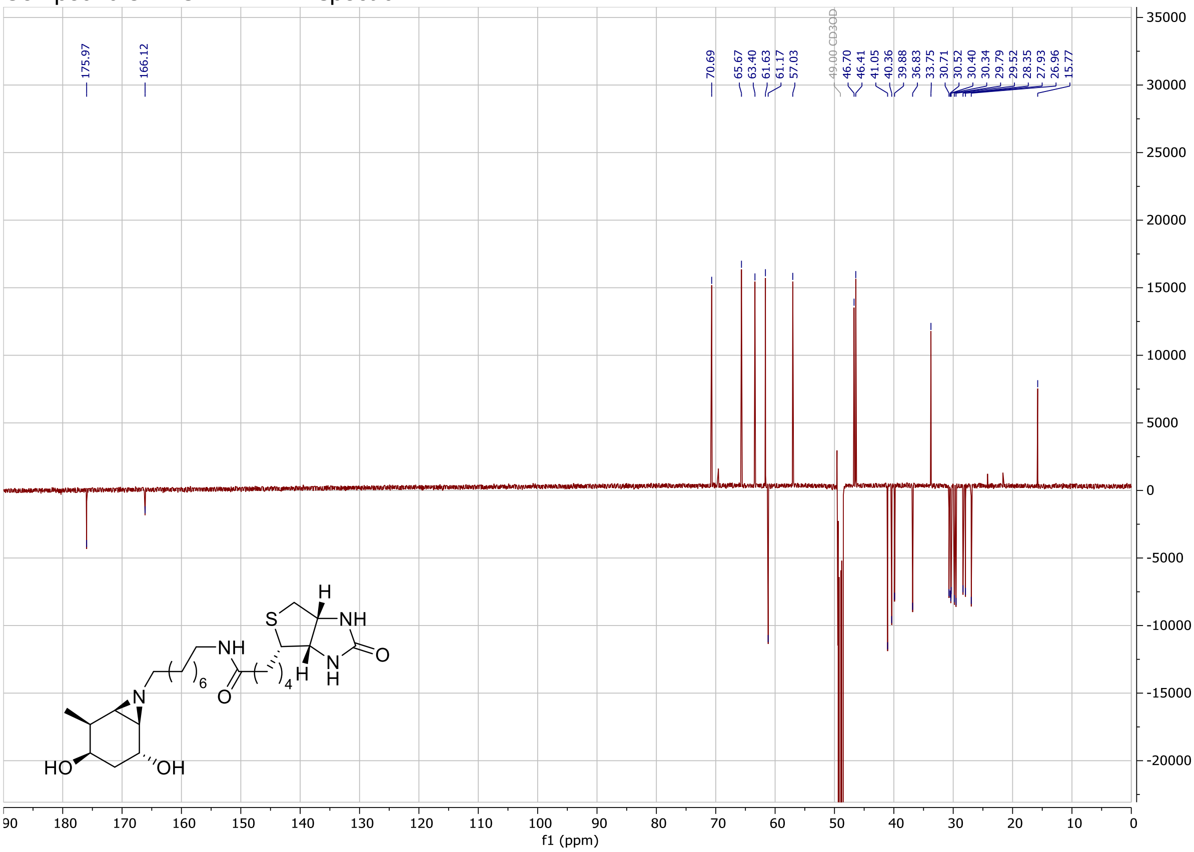

Compound **57** ^{1}H-^{1}H COSY spectrum

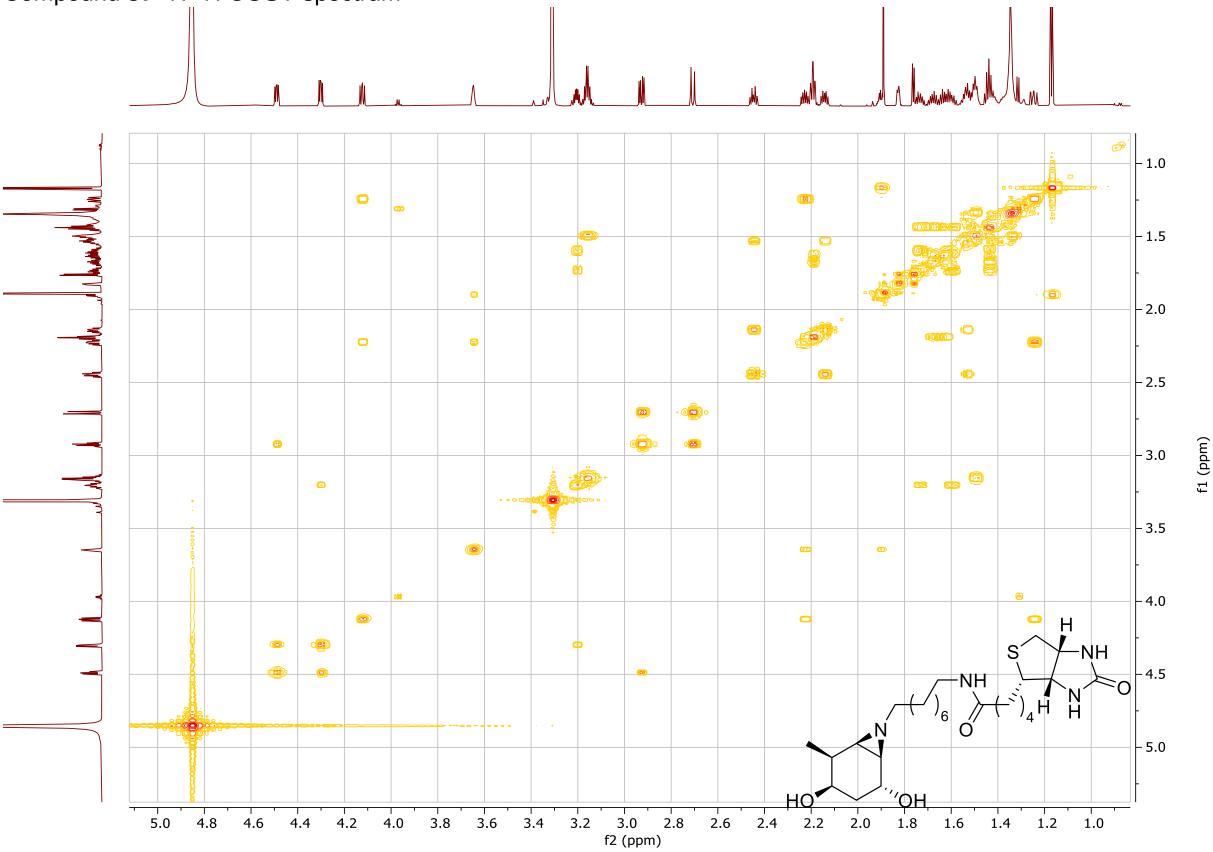

Compound **57** ^{1}H-^{13}C HSQC spectrum

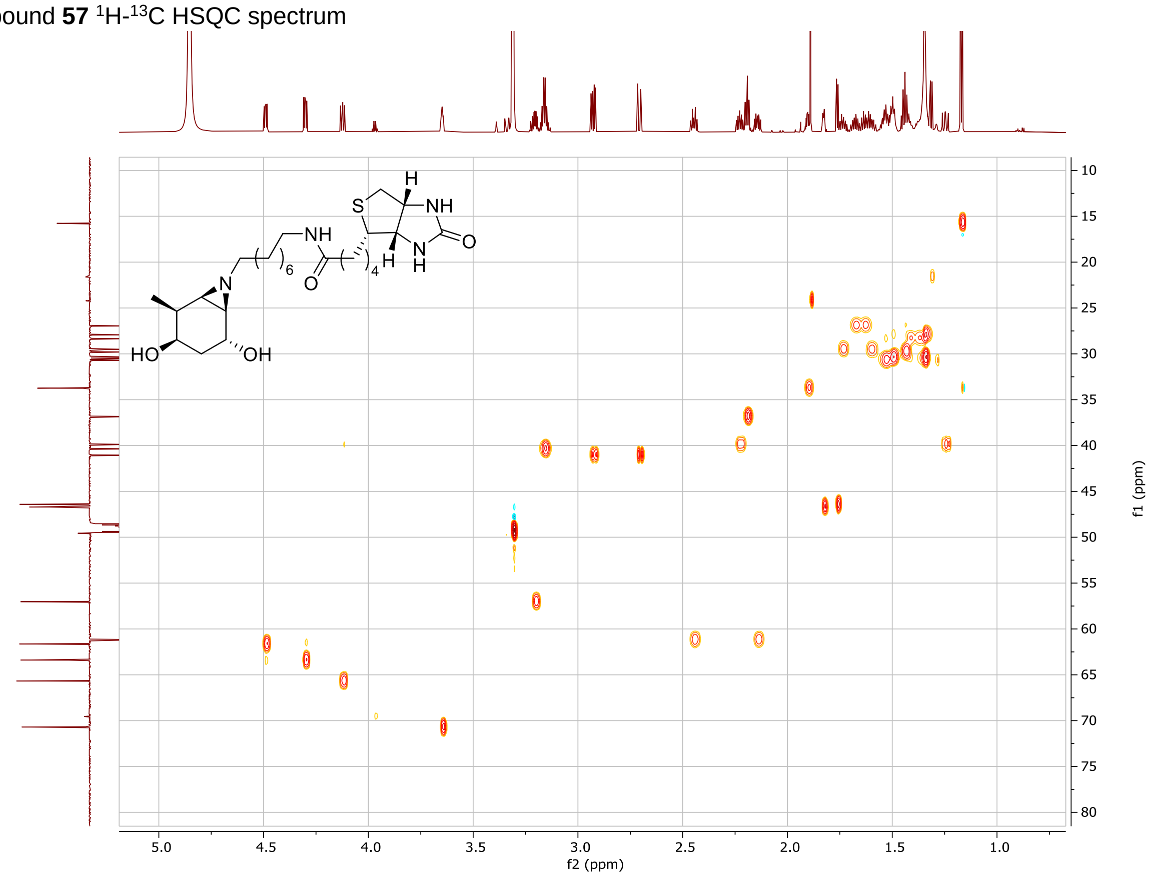

Compound **s111** ^{1}H NMR spectrum

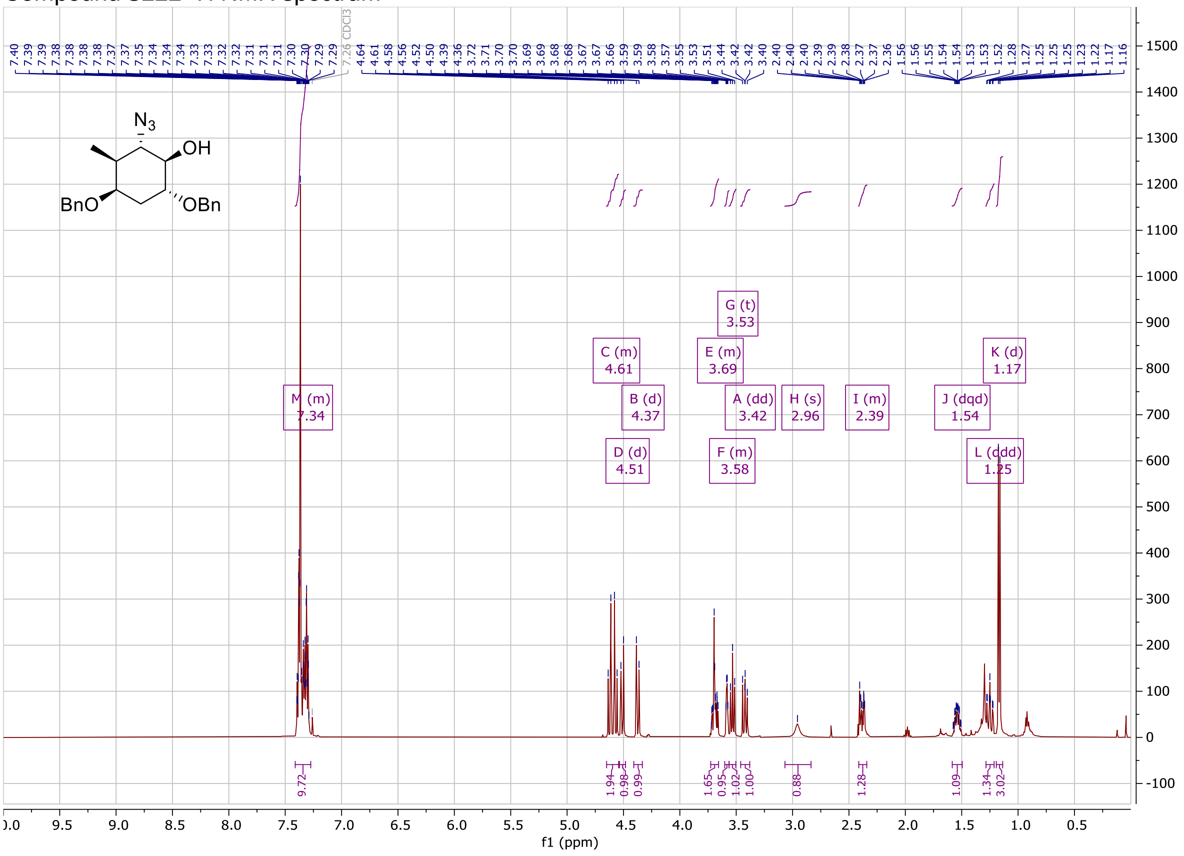

Compound **s111** ^{13}C NMR APT spectrum

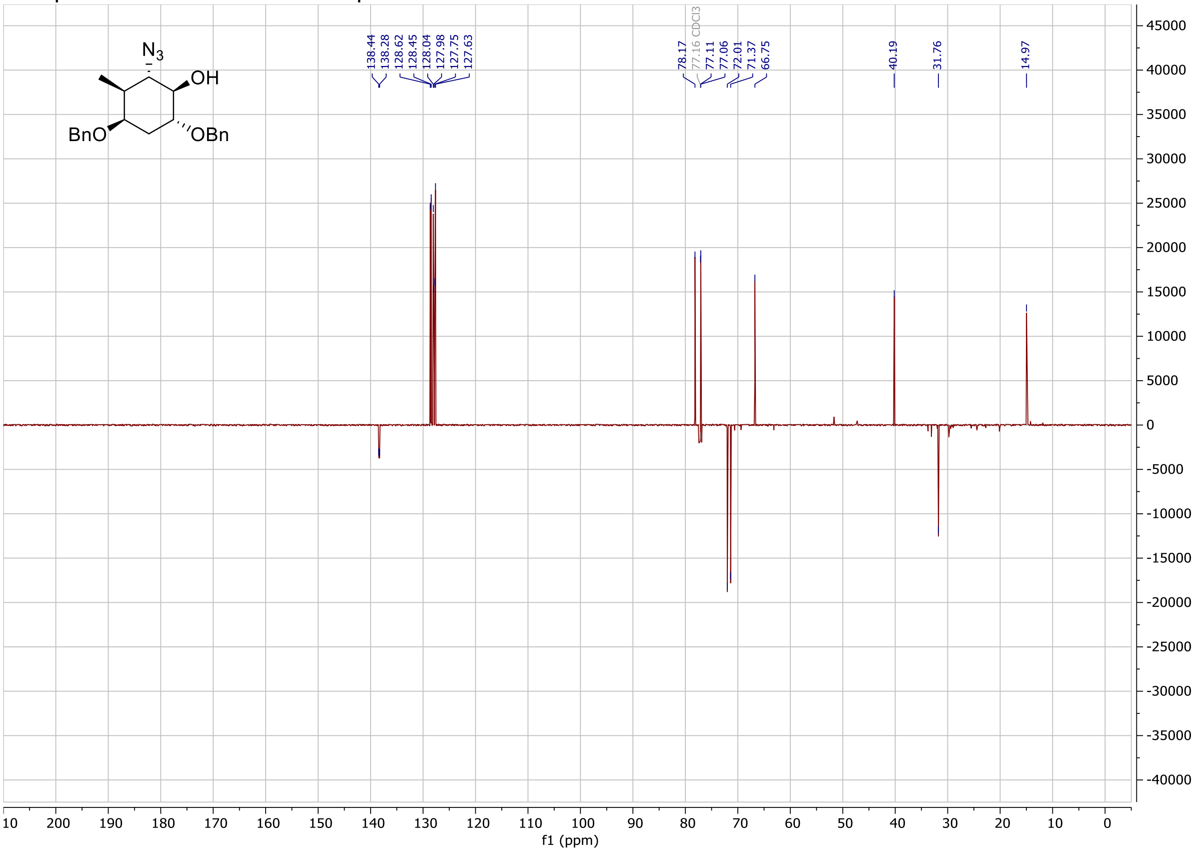

Compound **s111** ^{1}H-^{1}H COSY spectrum

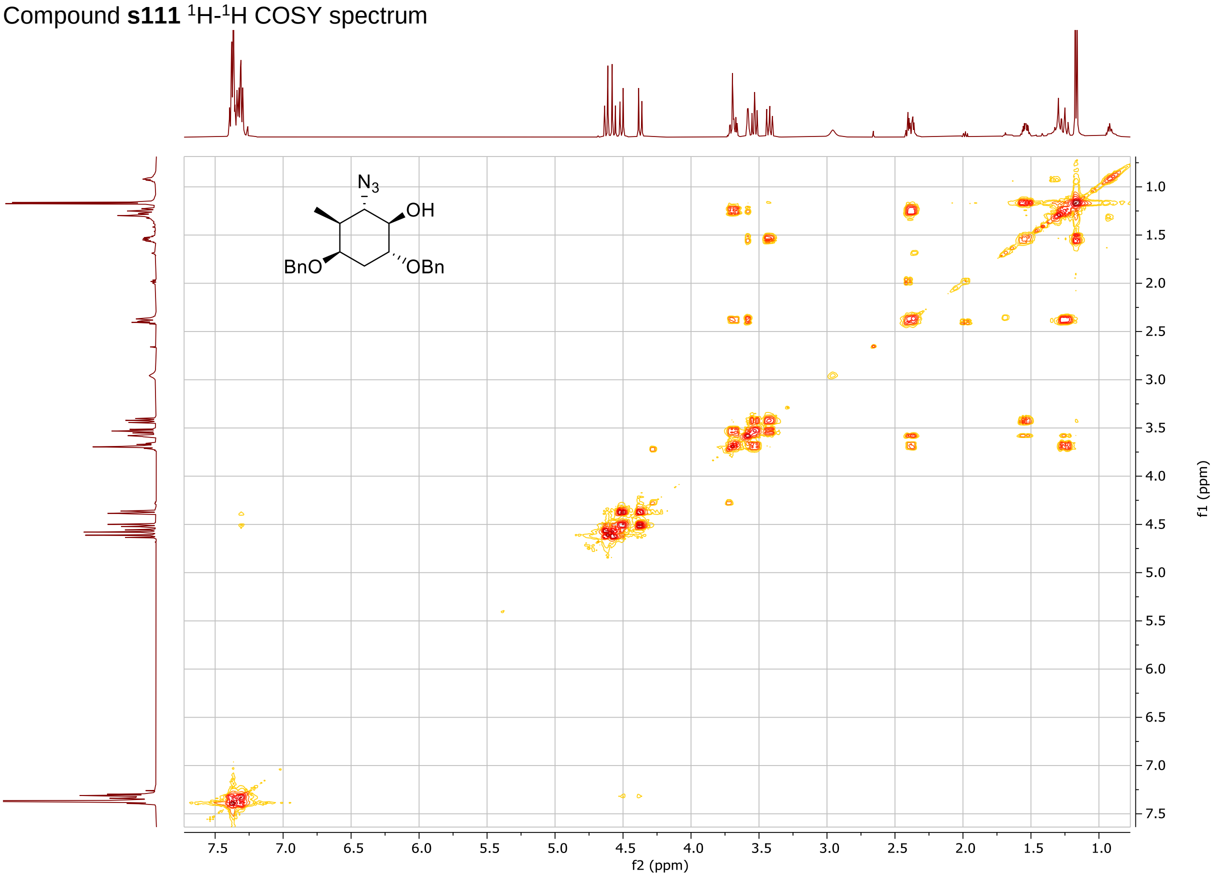

Compound **s111** ^{1}H-^{13}C HSQC spectrum

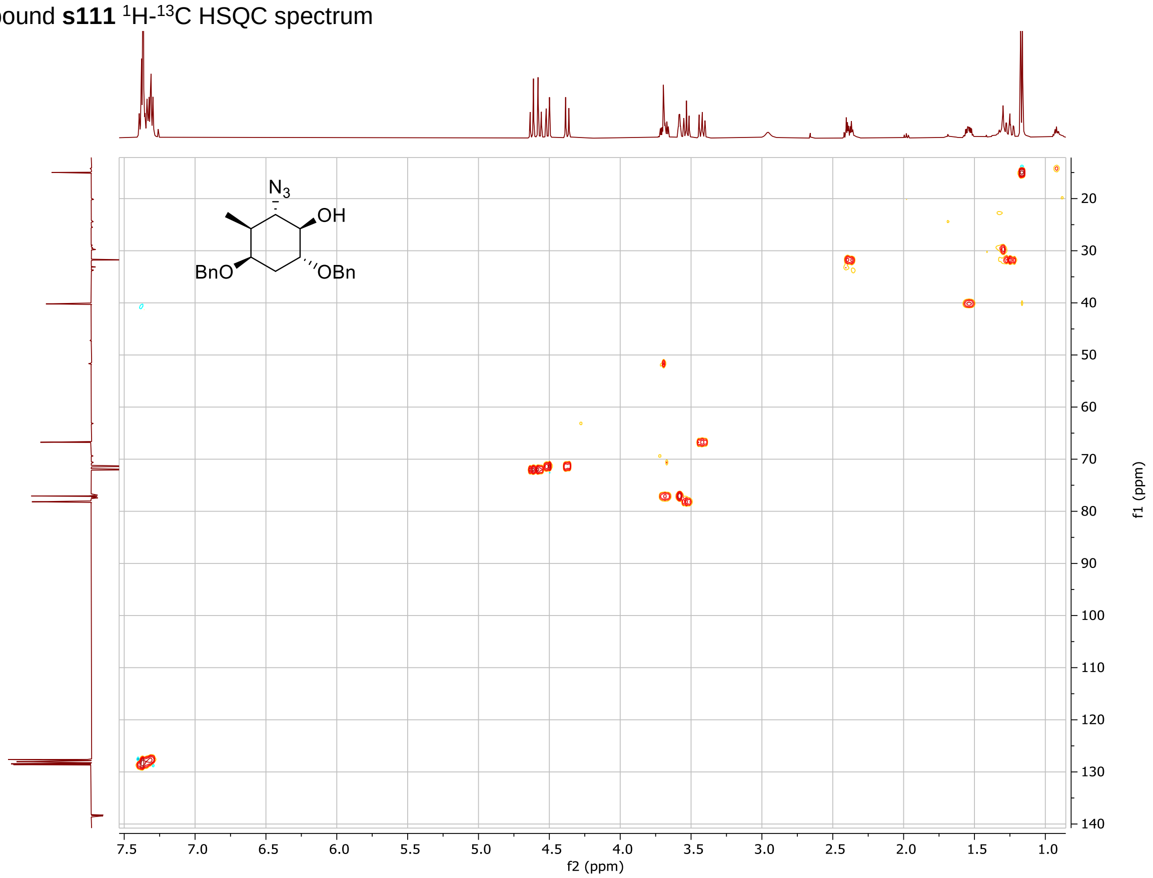

Compound **s111** ^{1}H-^{1}H NOESY spectrum

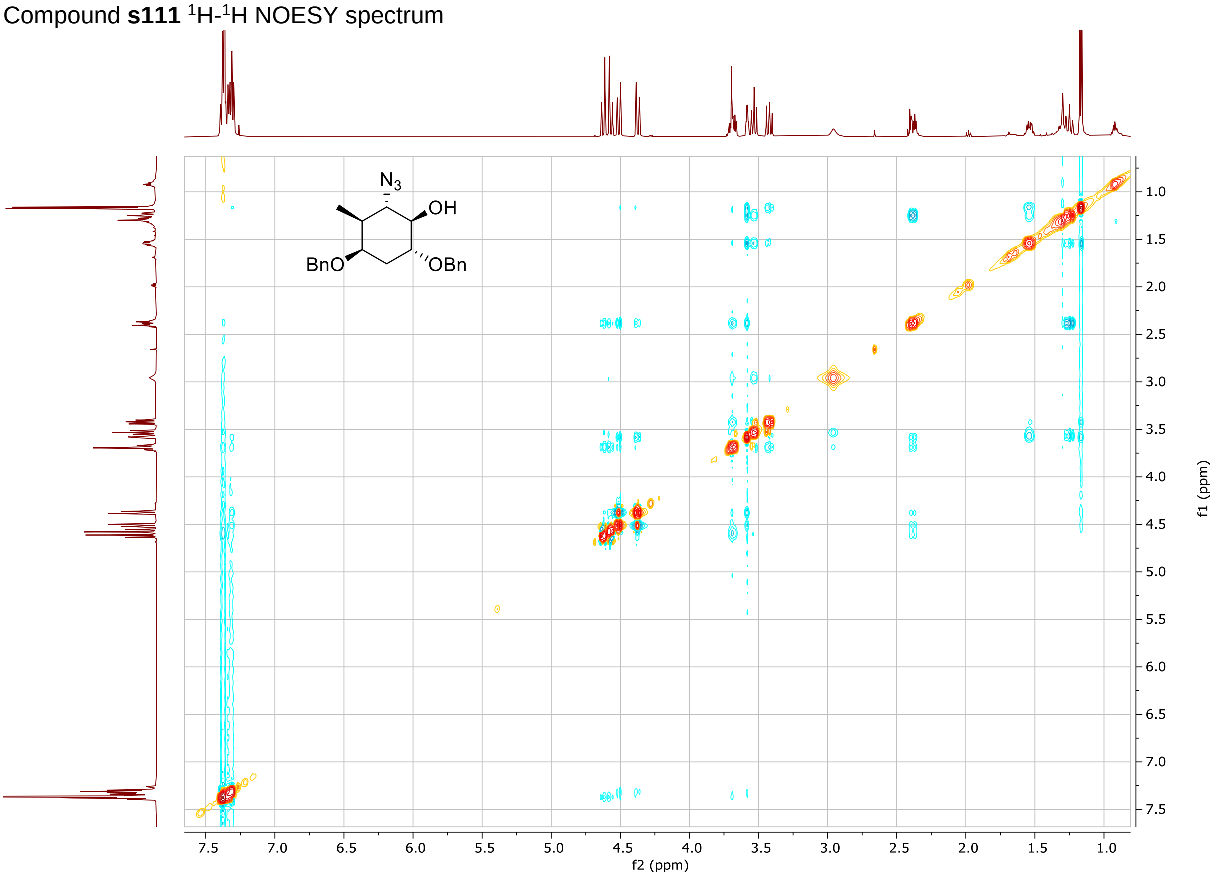

Compound **s111** ^{1}H-^{13}C HMBC spectrum

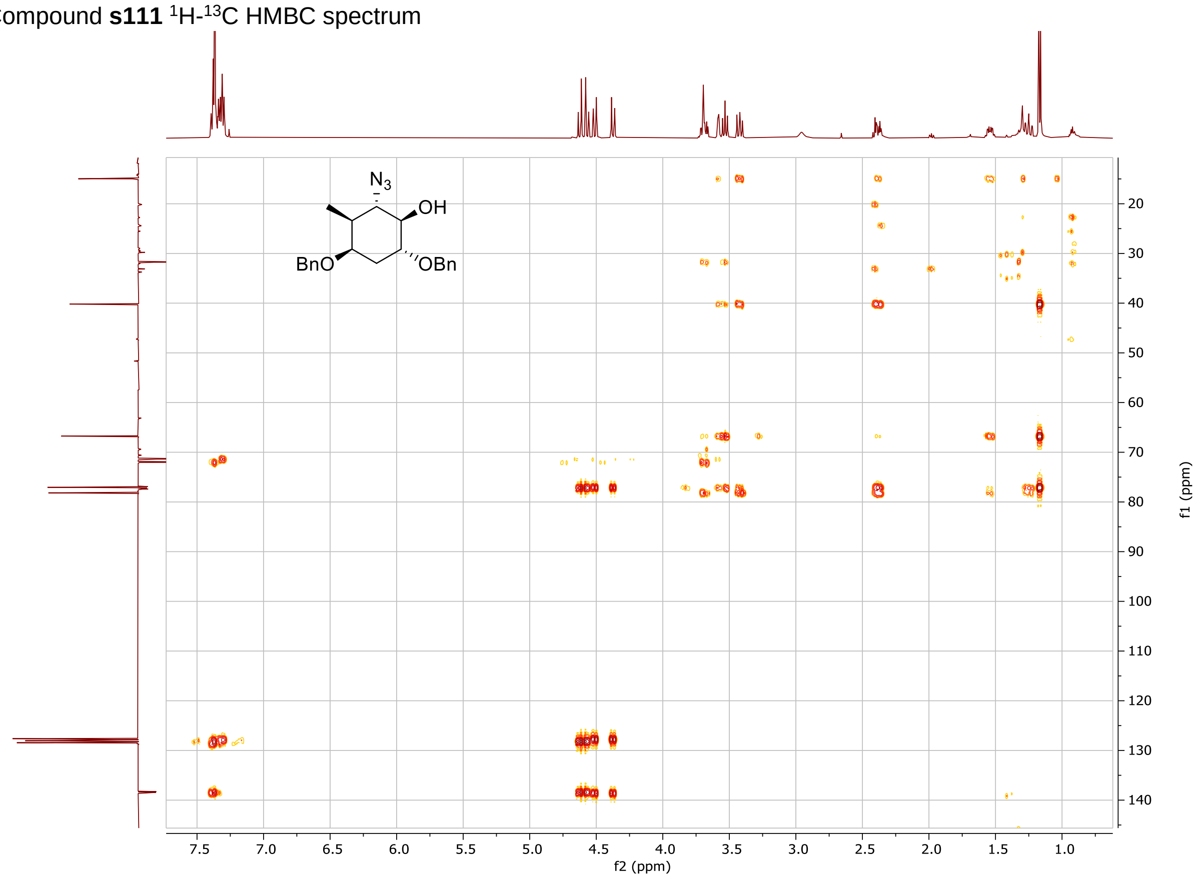

Compound **s112** ^{1}H NMR spectrum (332 K)

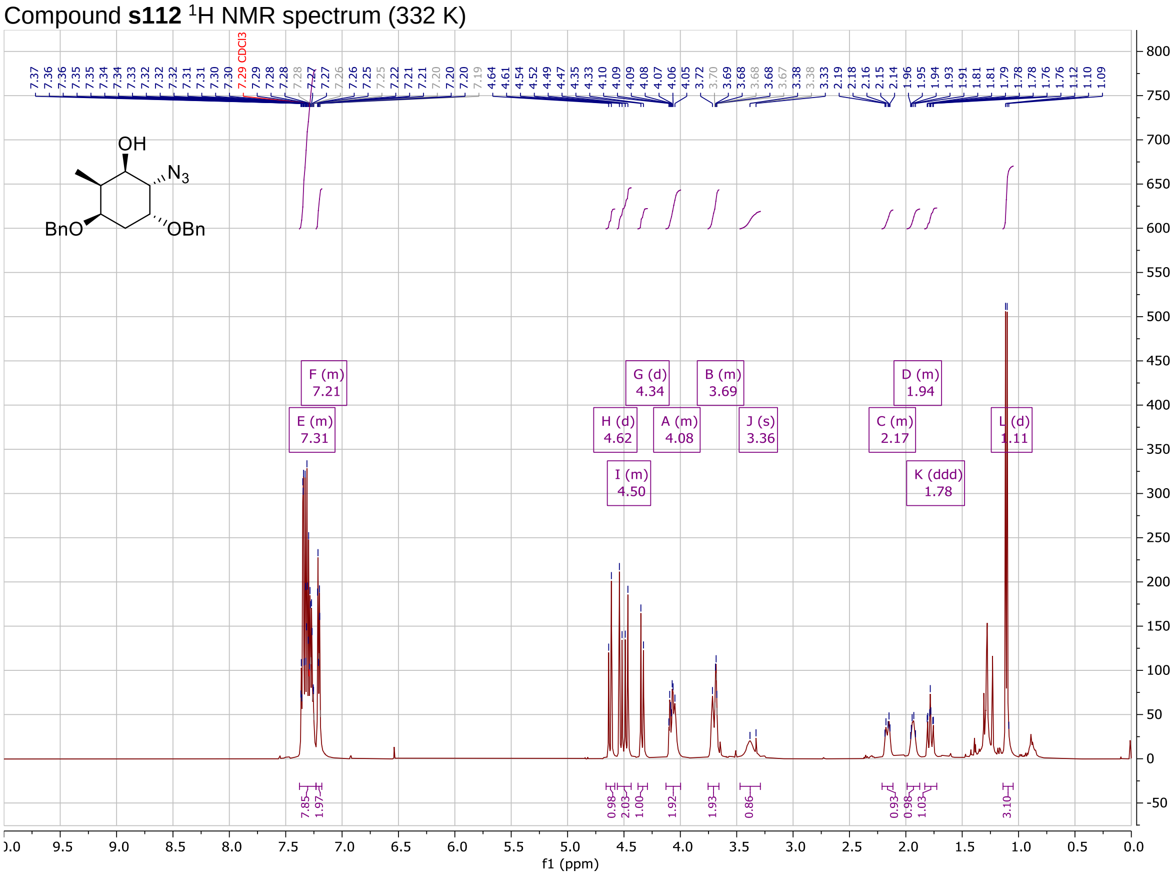

Compound **s112** ^{13}C NMR APT spectrum (332 K)

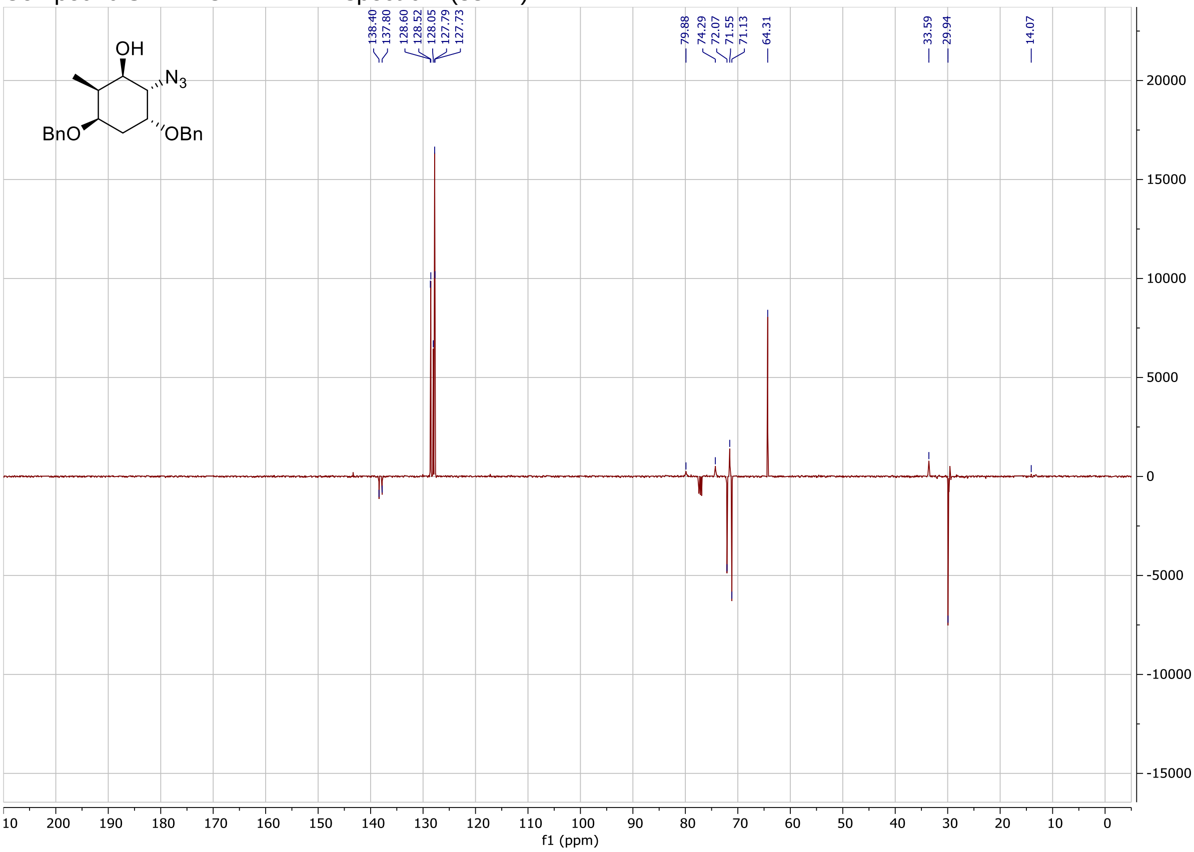

Compound **s112** ^{1}H-^{1}H COSY spectrum (332 K)

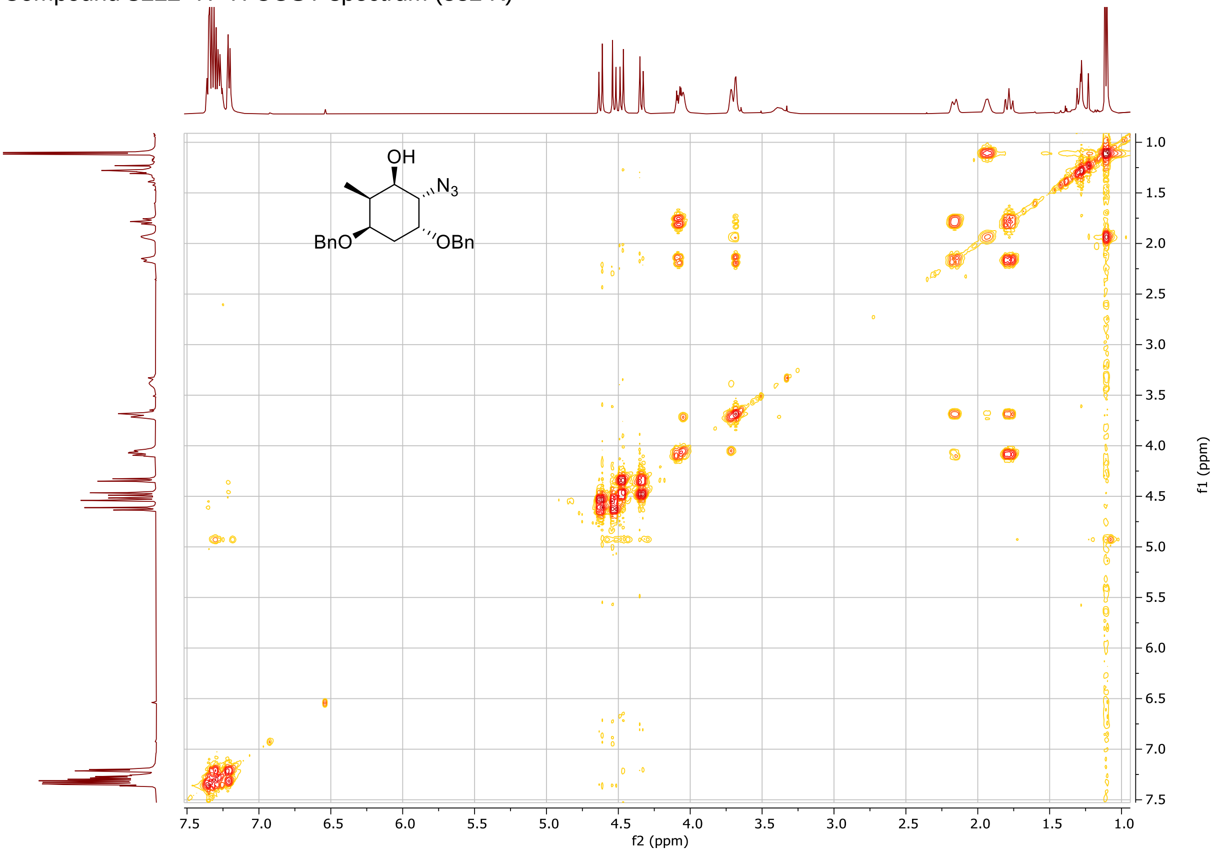

Compound **s112** ^{1}H-^{13}C HSQC spectrum (332 K)

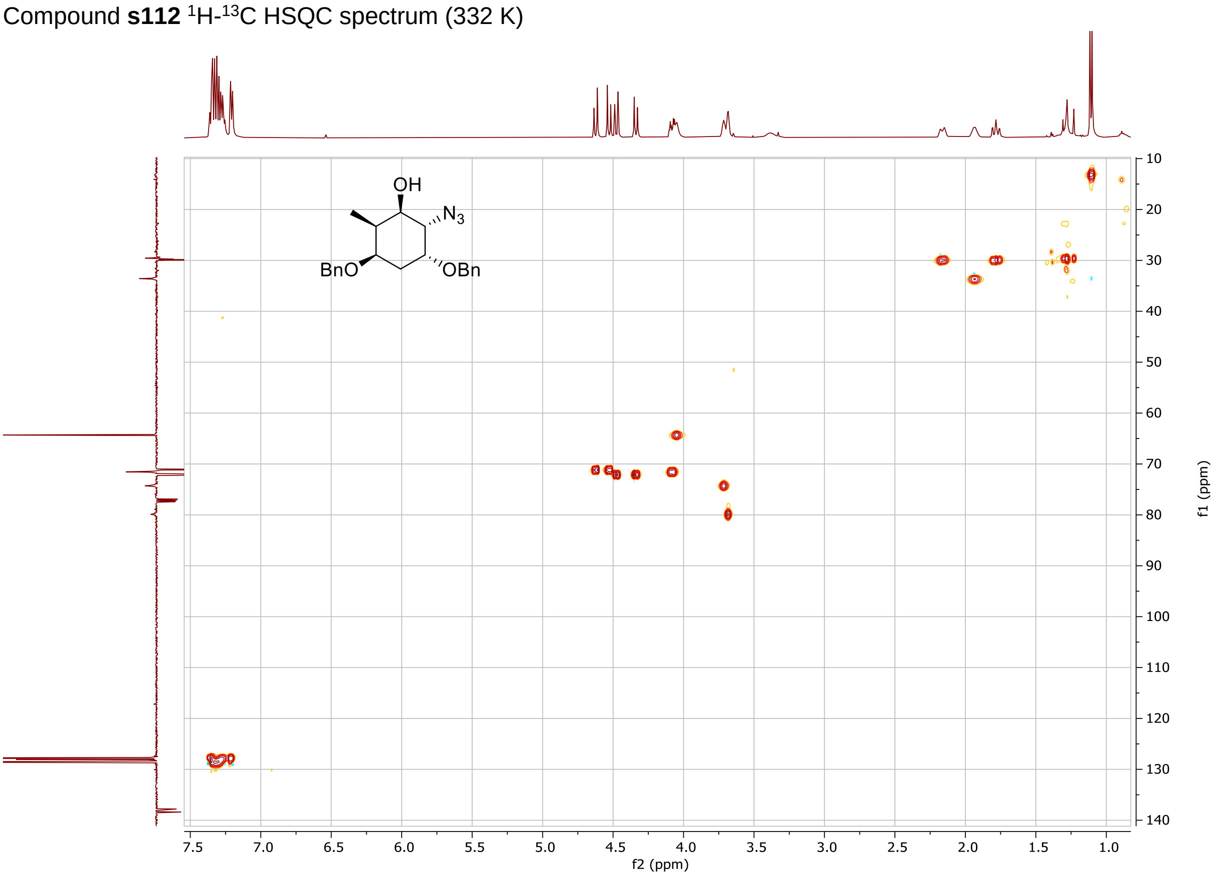

Compound **s113** ^{1}H NMR spectrum

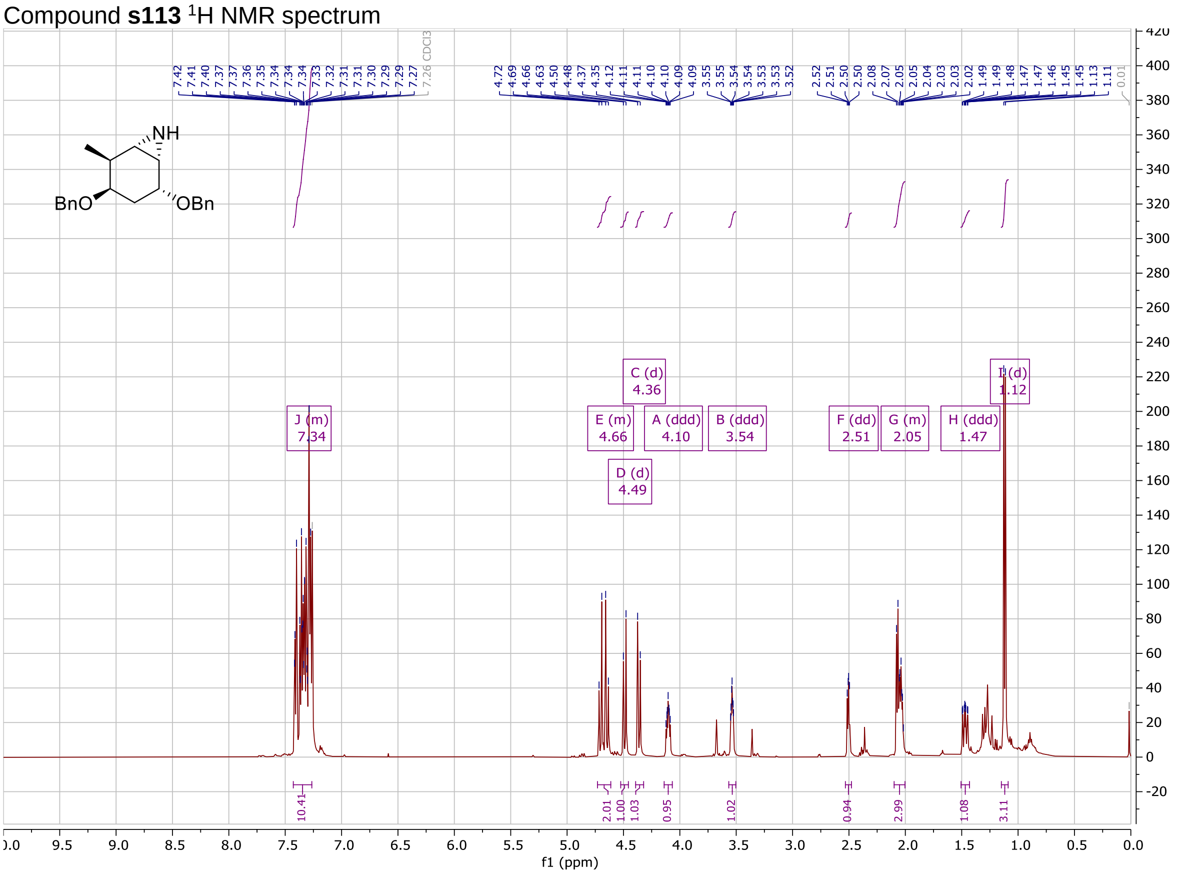

Compound **s113** ^{13}C NMR APT spectrum

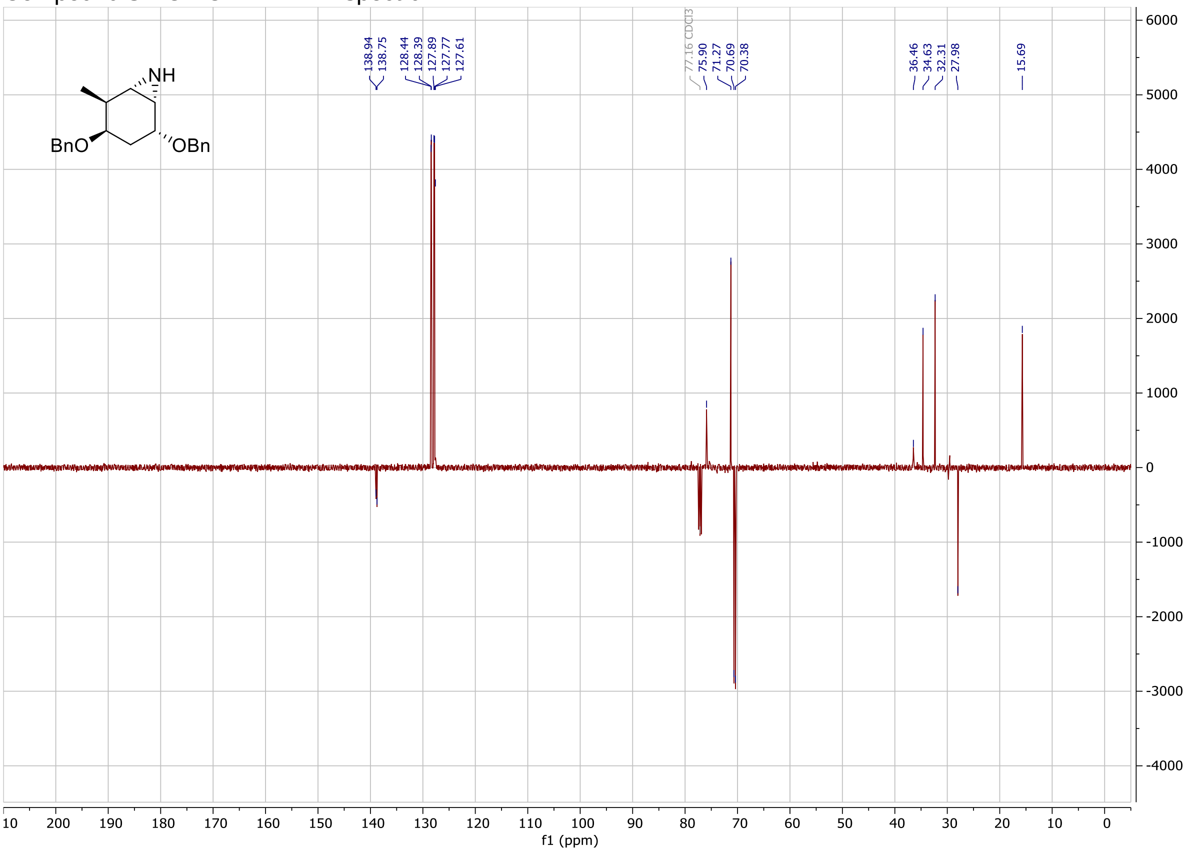

Compound **s113** ^{1}H-^{1}H COSY spectrum

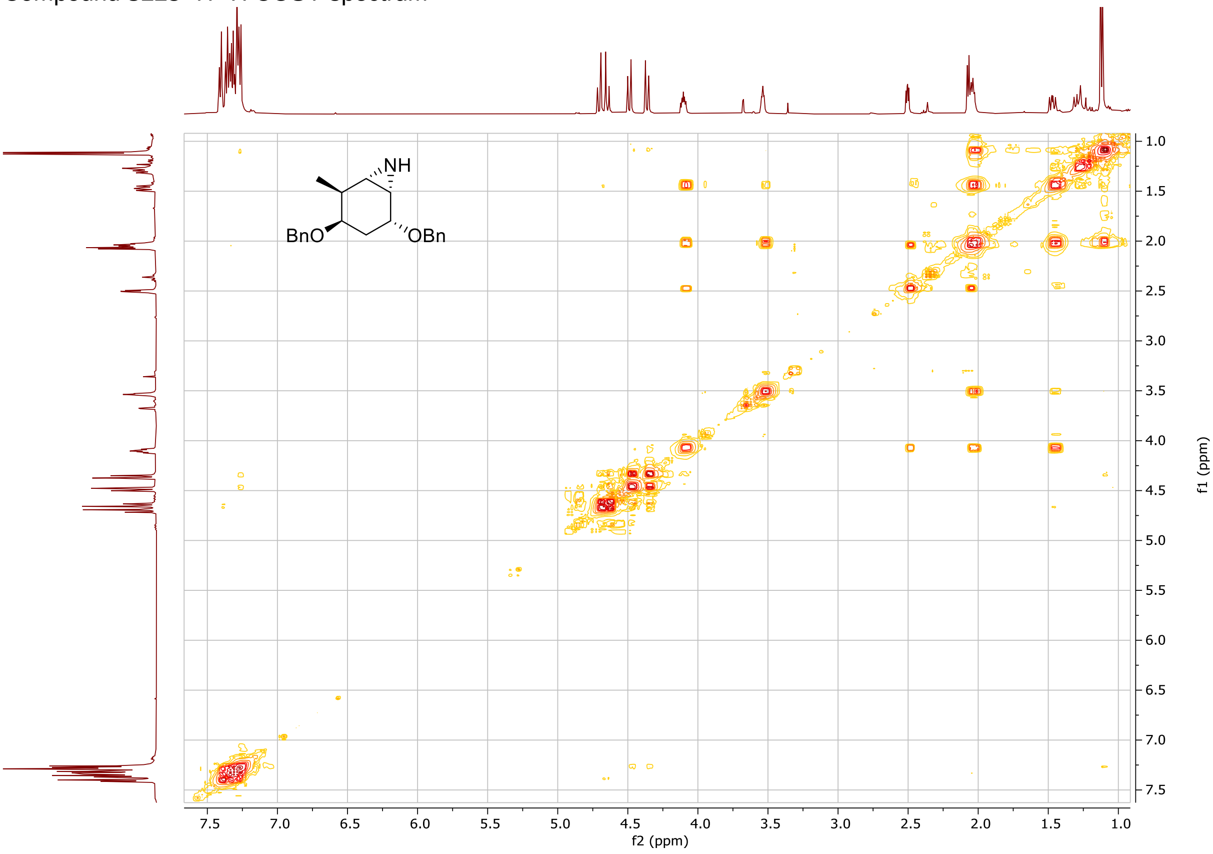

Compound **s113** ^1H-^{13}C HSQC spectrum

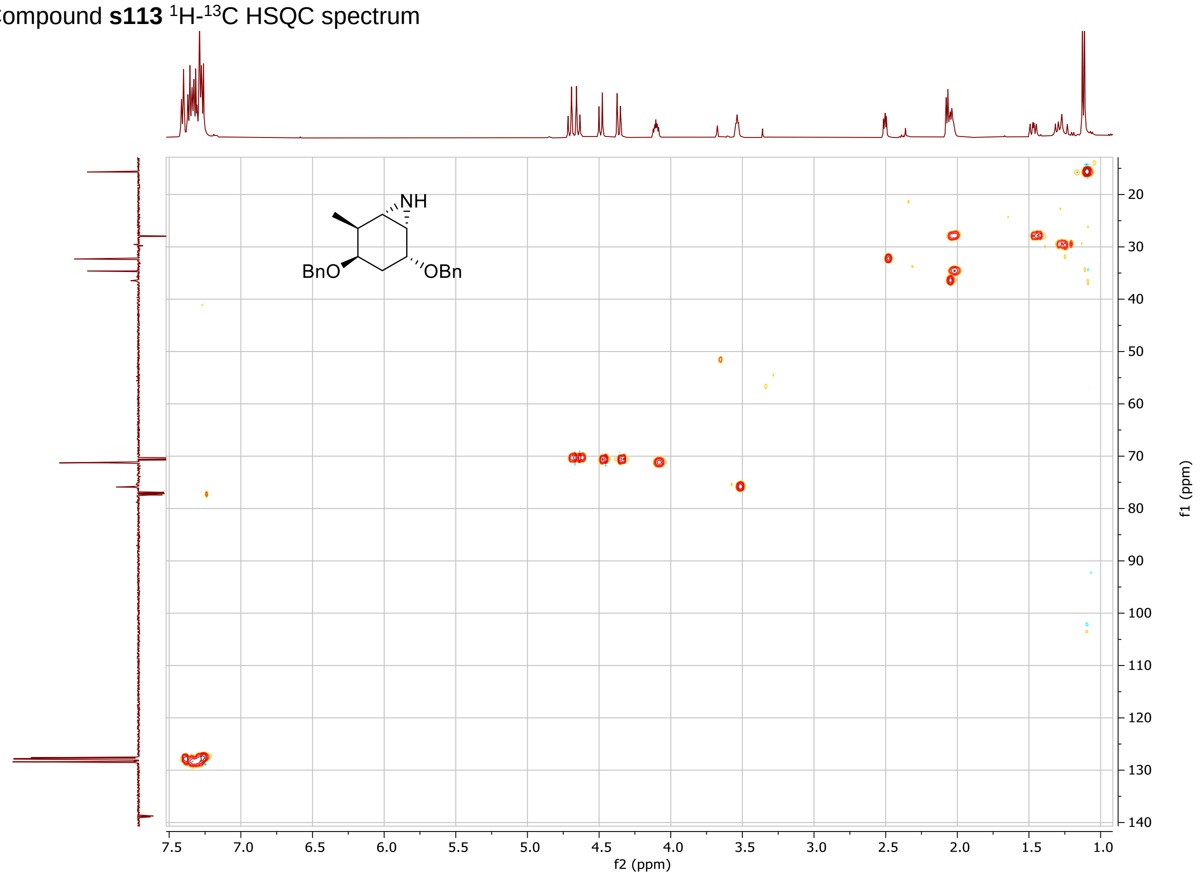

Compound **s114** ^1H NMR spectrum

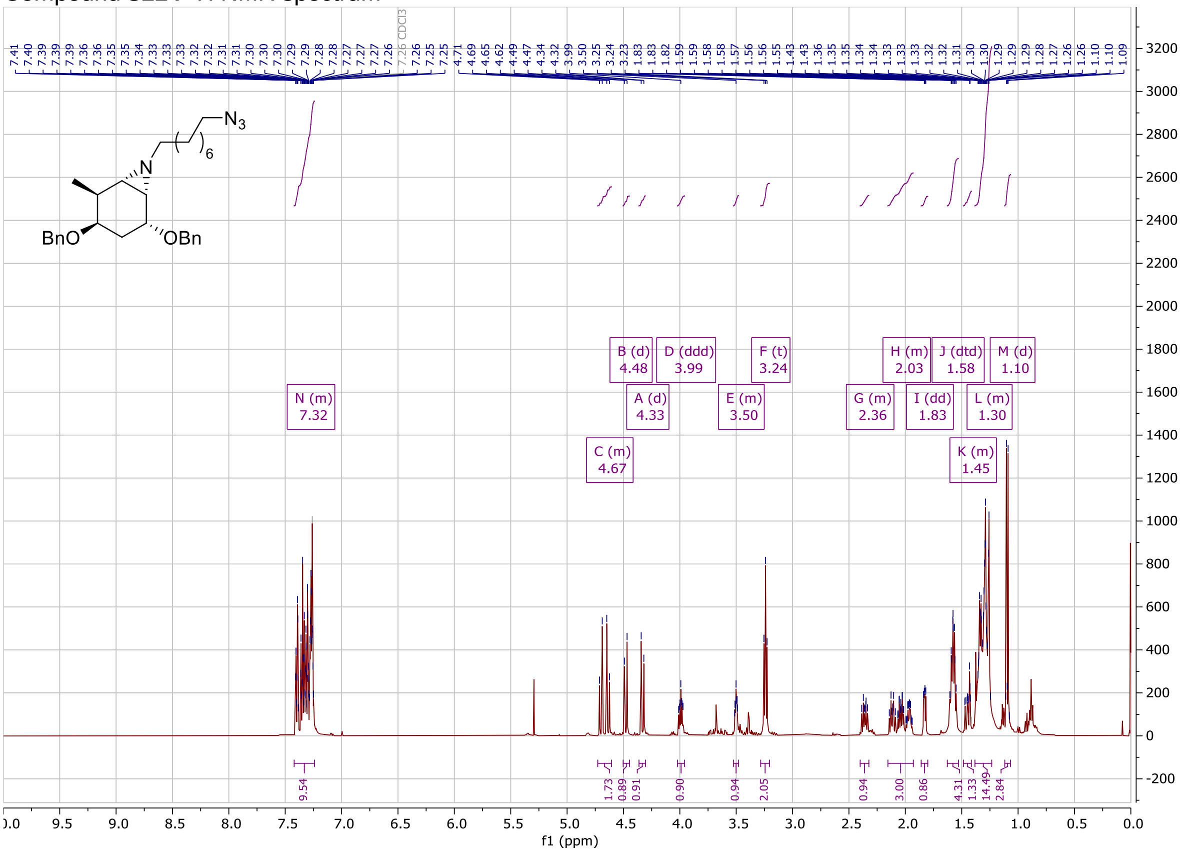

Compound **s114** ^{13}C NMR APT spectrum

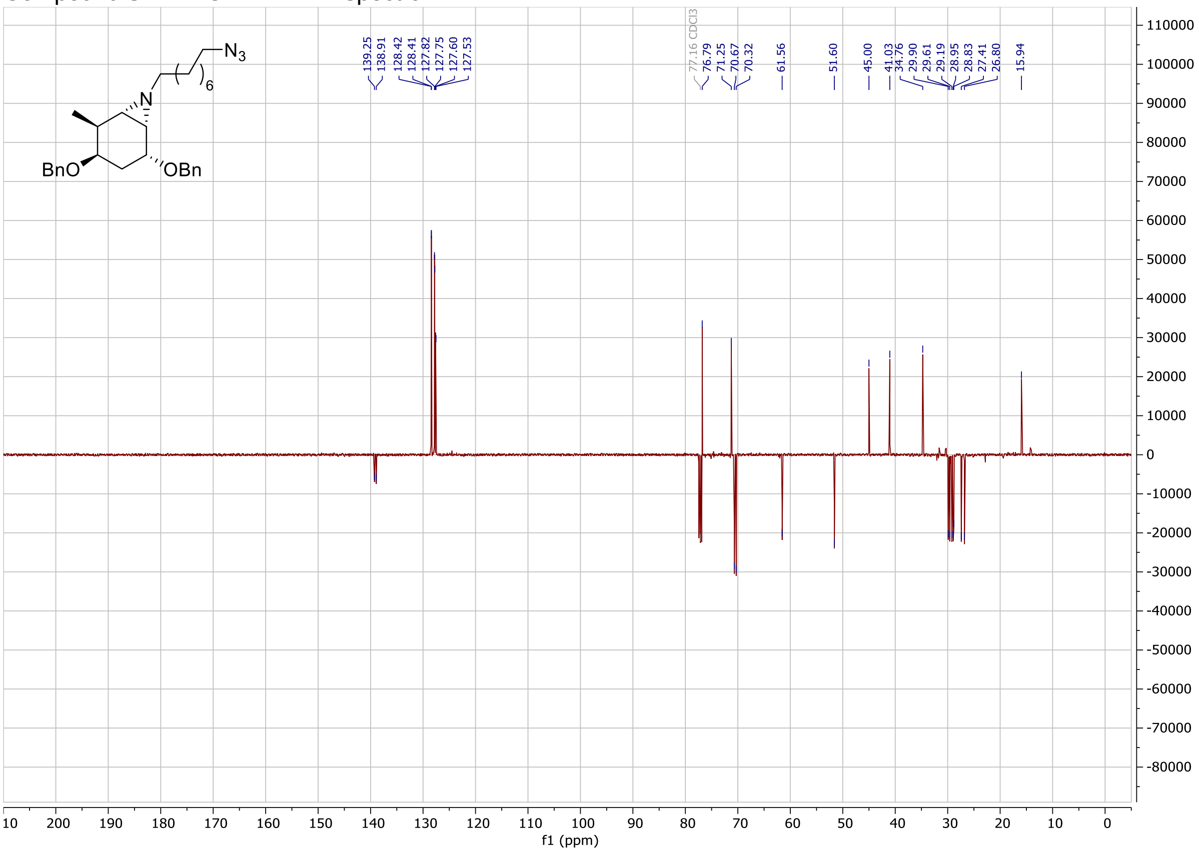

Compound **s114** ^{1}H-^{1}H COSY spectrum

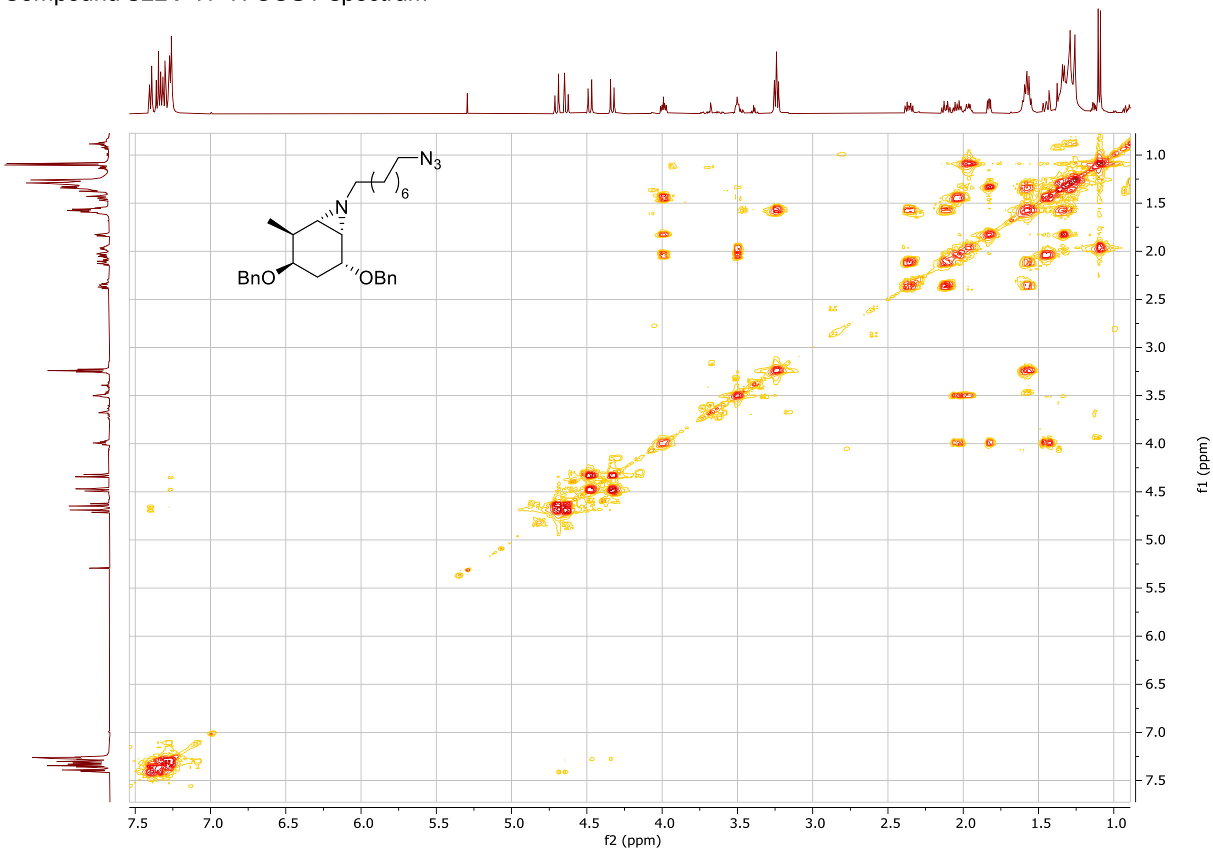

Compound **s114** ^{1}H-^{13}C HSQC spectrum

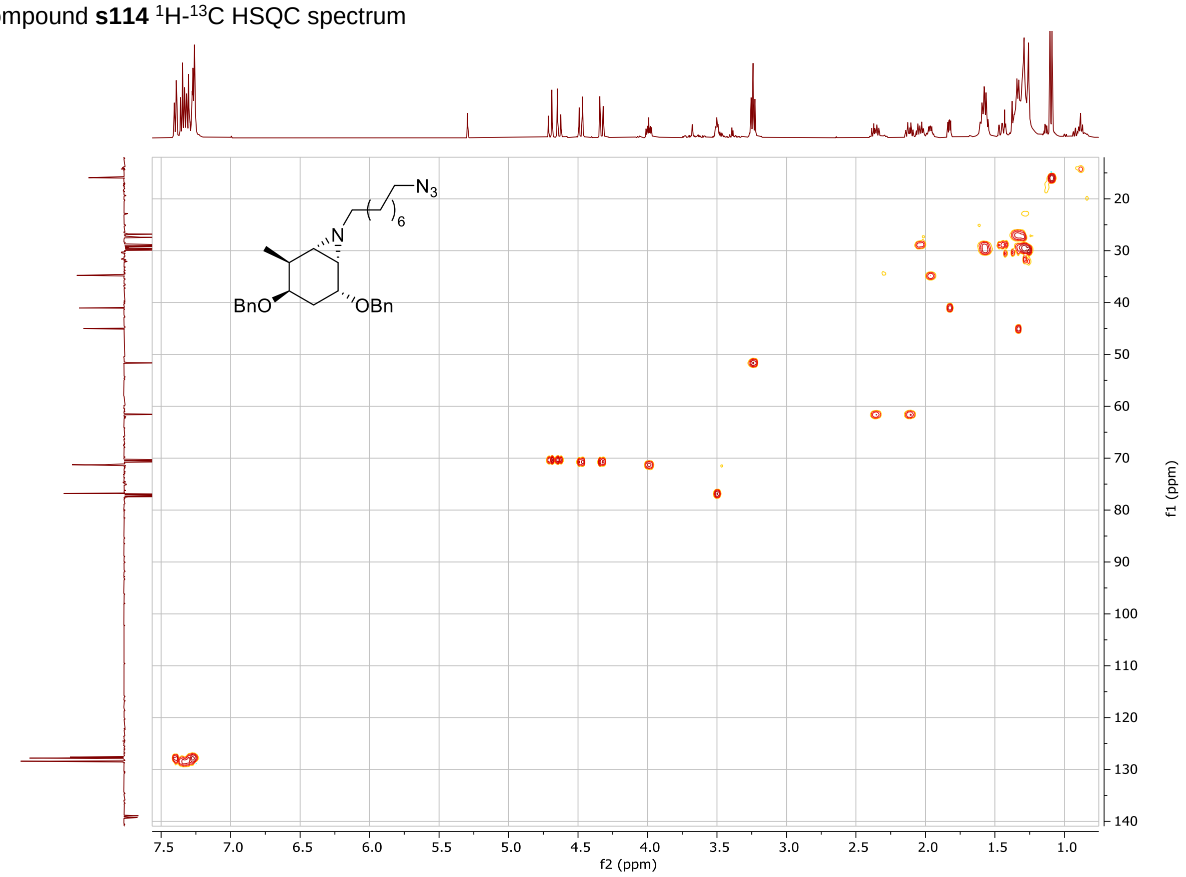

Compound **s114** ^{1}H-^{13}C HMBC spectrum

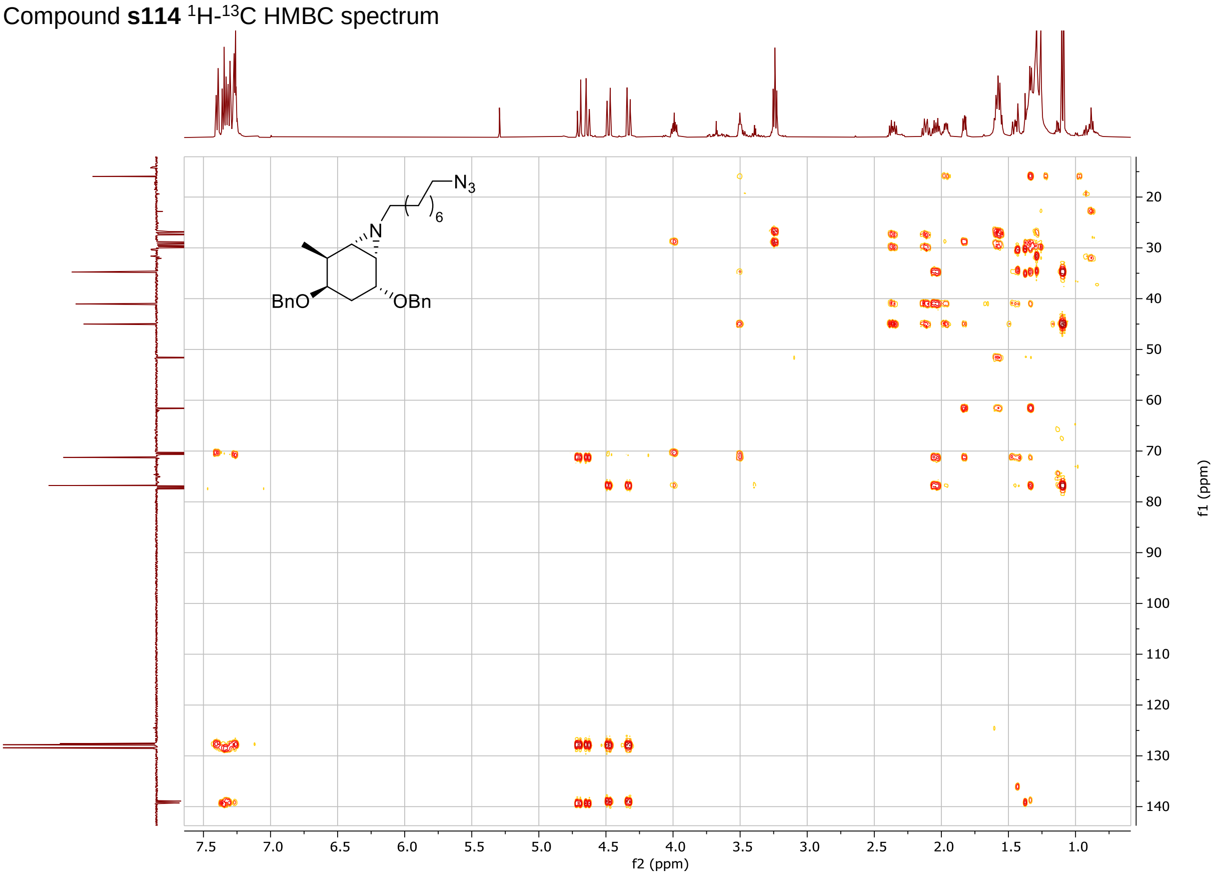

Compound **s115** ^{1}H NMR spectrum

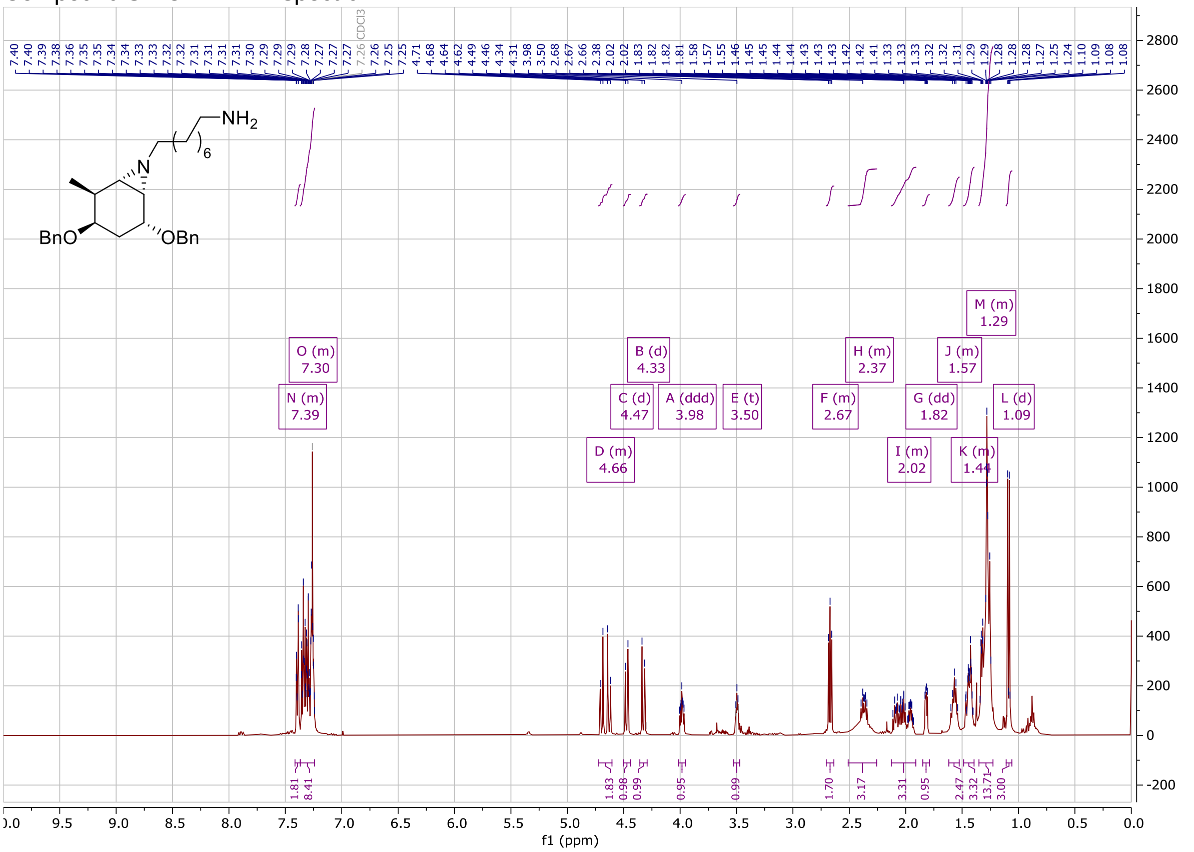

Compound **s115** ^{13}C NMR APT spectrum

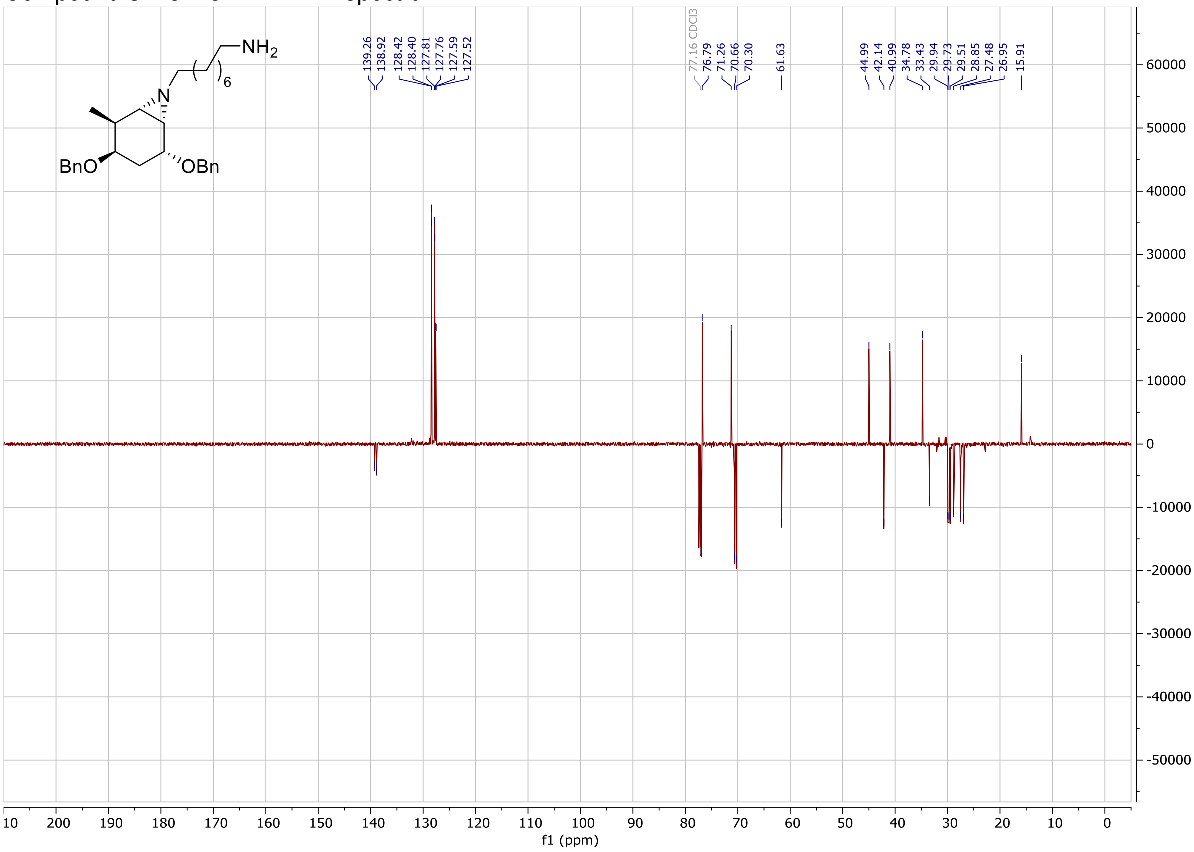

Compound **s115** ^{1}H-^{1}H COSY spectrum

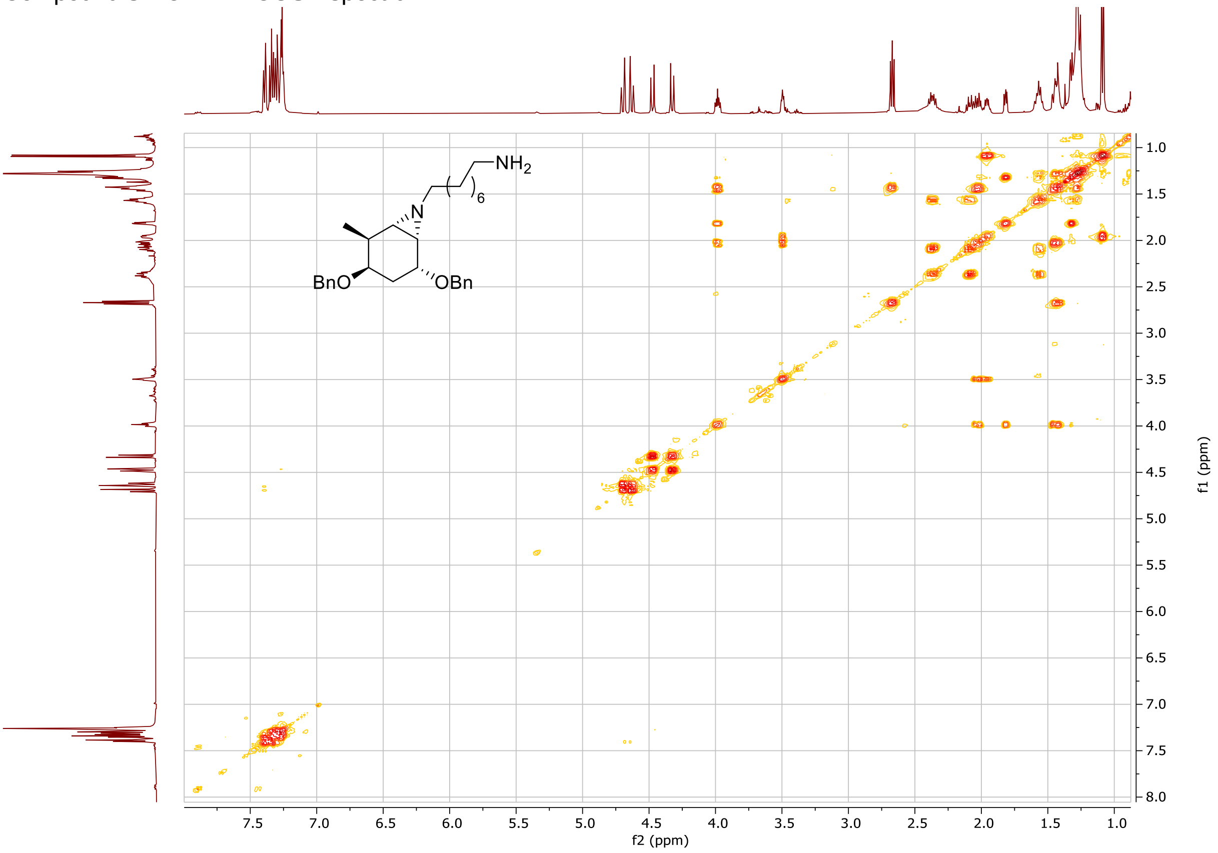

Compound **s115** ^{1}H-^{13}C HSQC spectrum

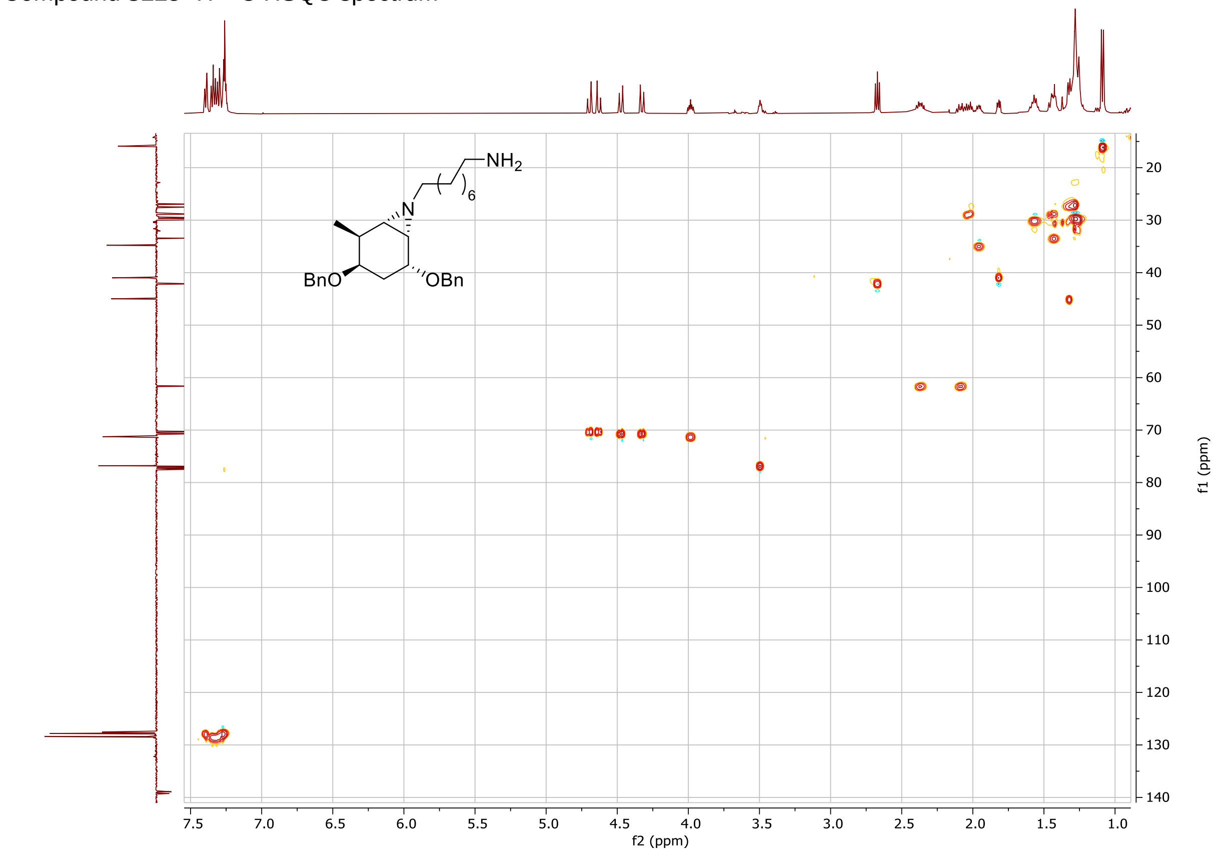

Compound **s115** ^{1}H-^{13}C HMBC spectrum

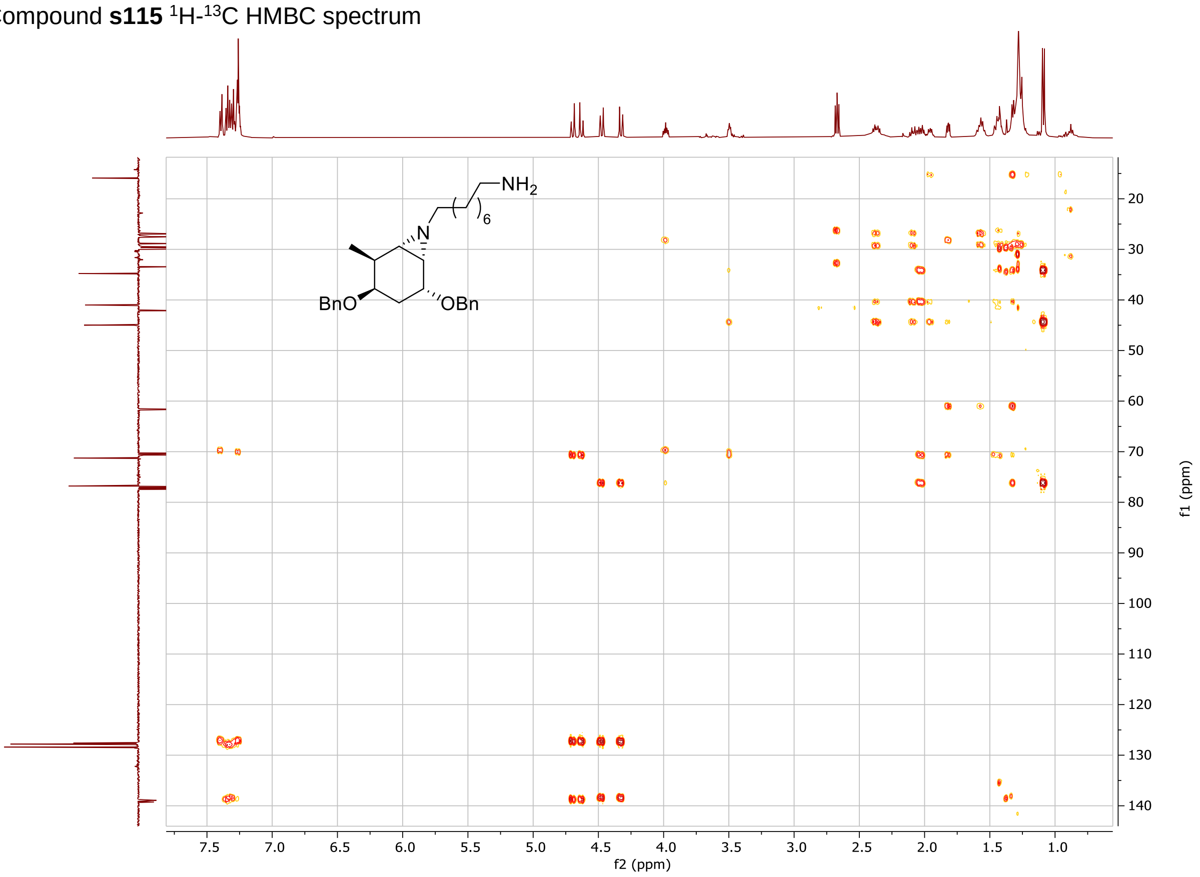

Compound **s116** ^{1}H NMR spectrum

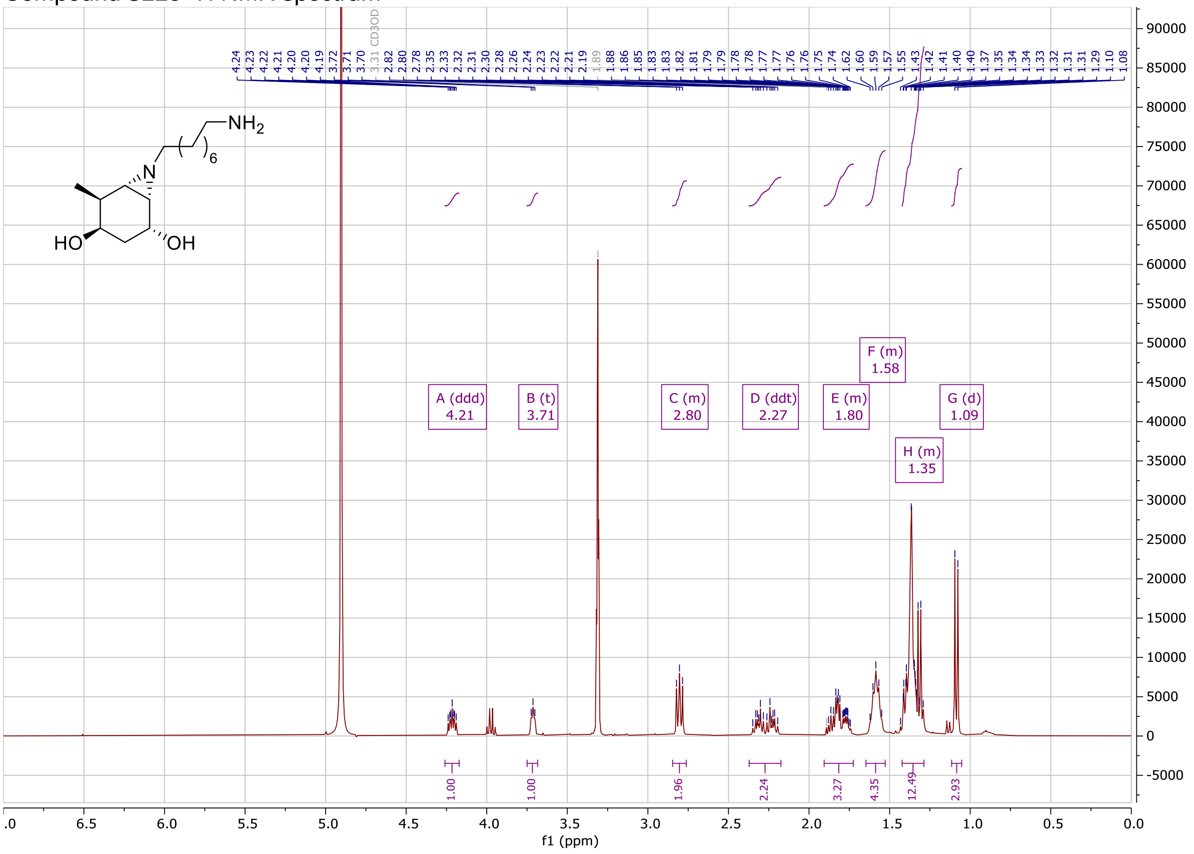

Compound **s116** ^{13}C NMR APT spectrum

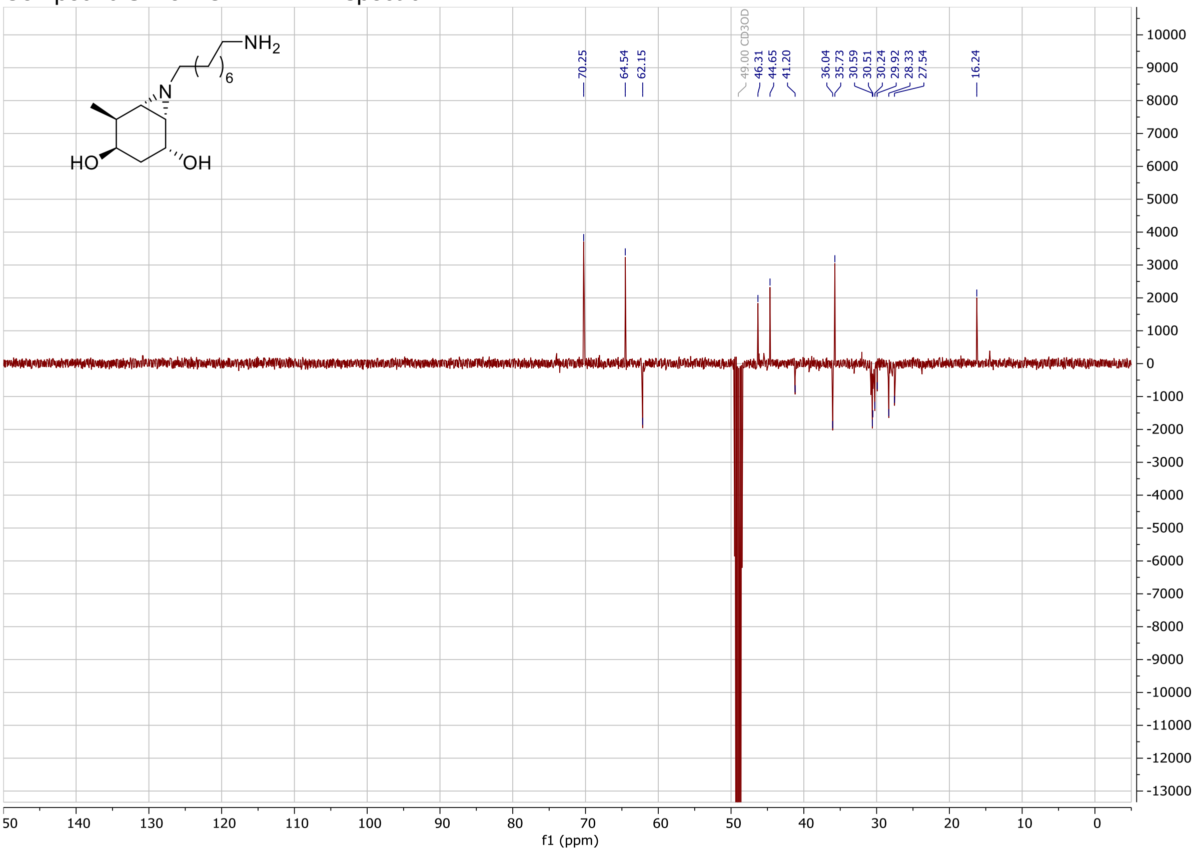

Compound **49** ^{1}H NMR spectrum

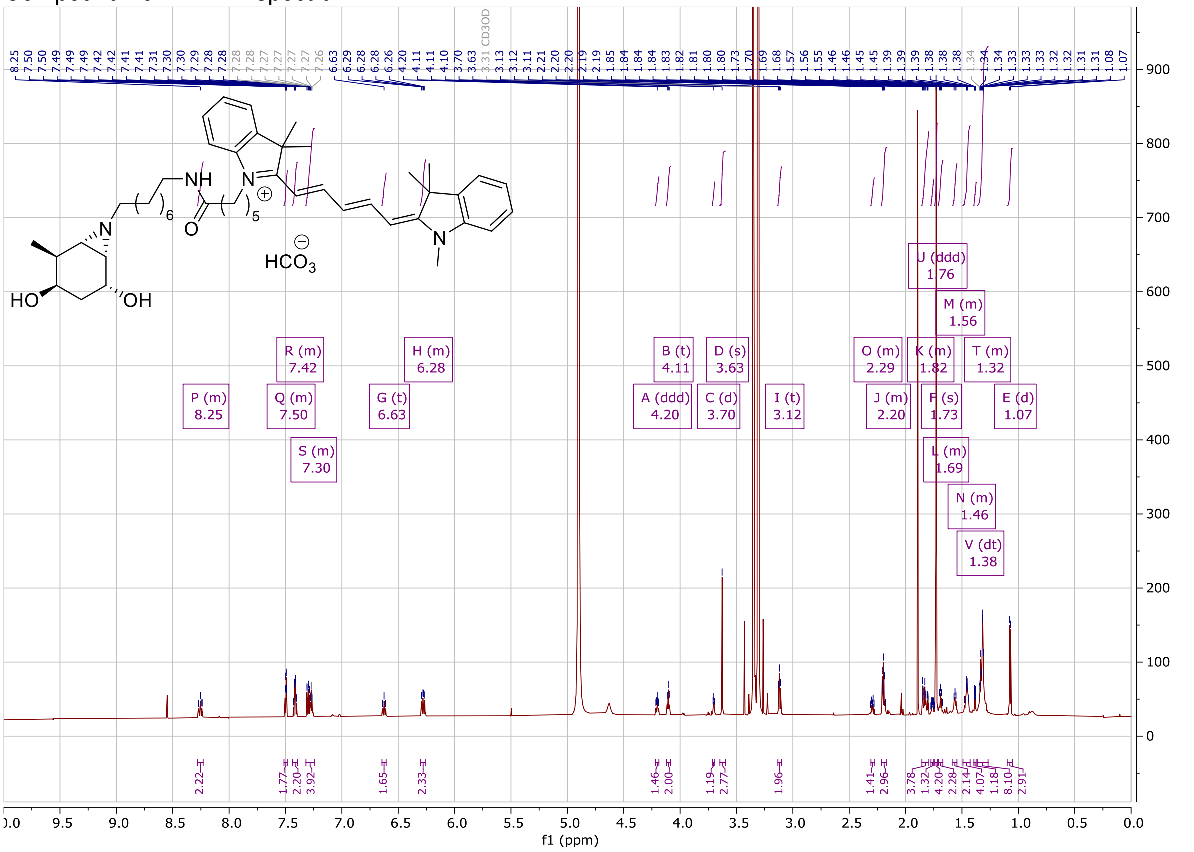

Compound **49** ^{13}C NMR APT spectrum

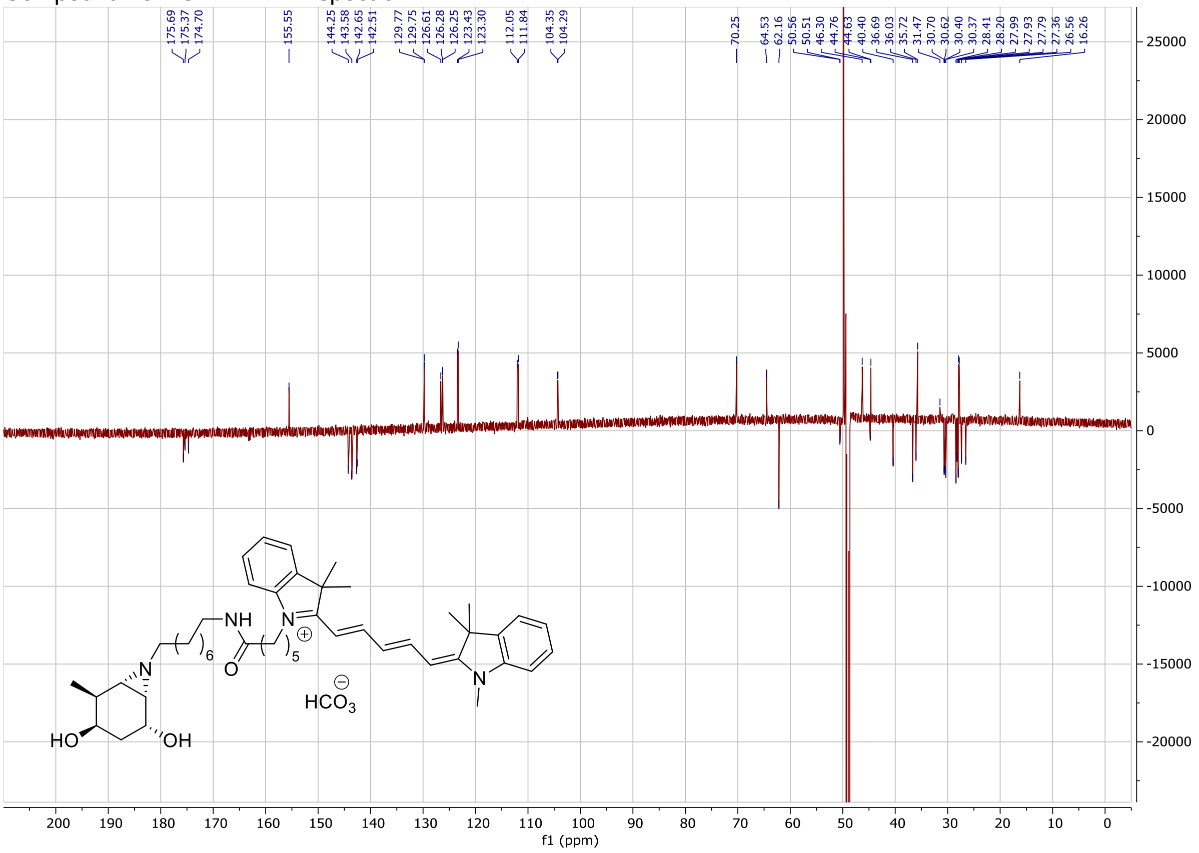

Compound **49** ^{1}H-^{1}H COSY spectrum

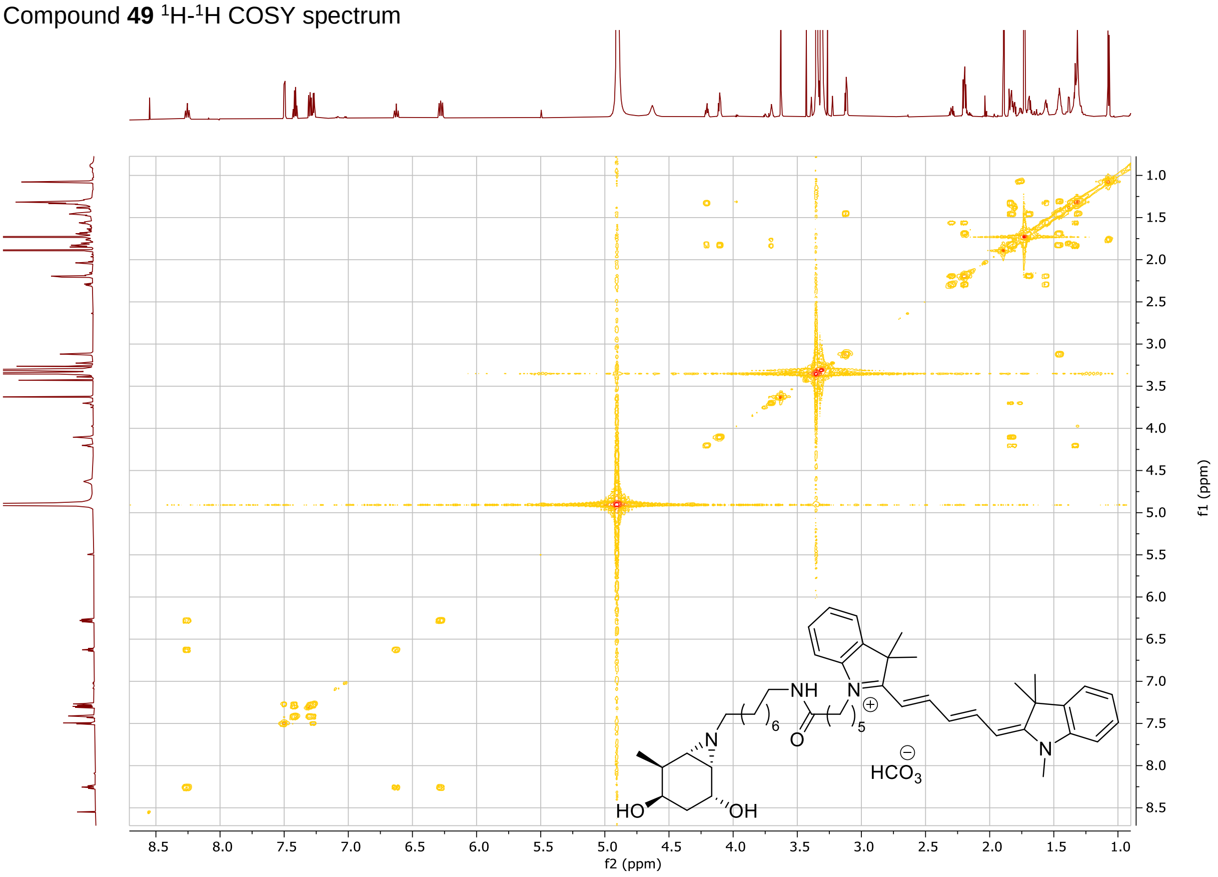

Compound **49** ^{1}H-^{13}C HSQC spectrum

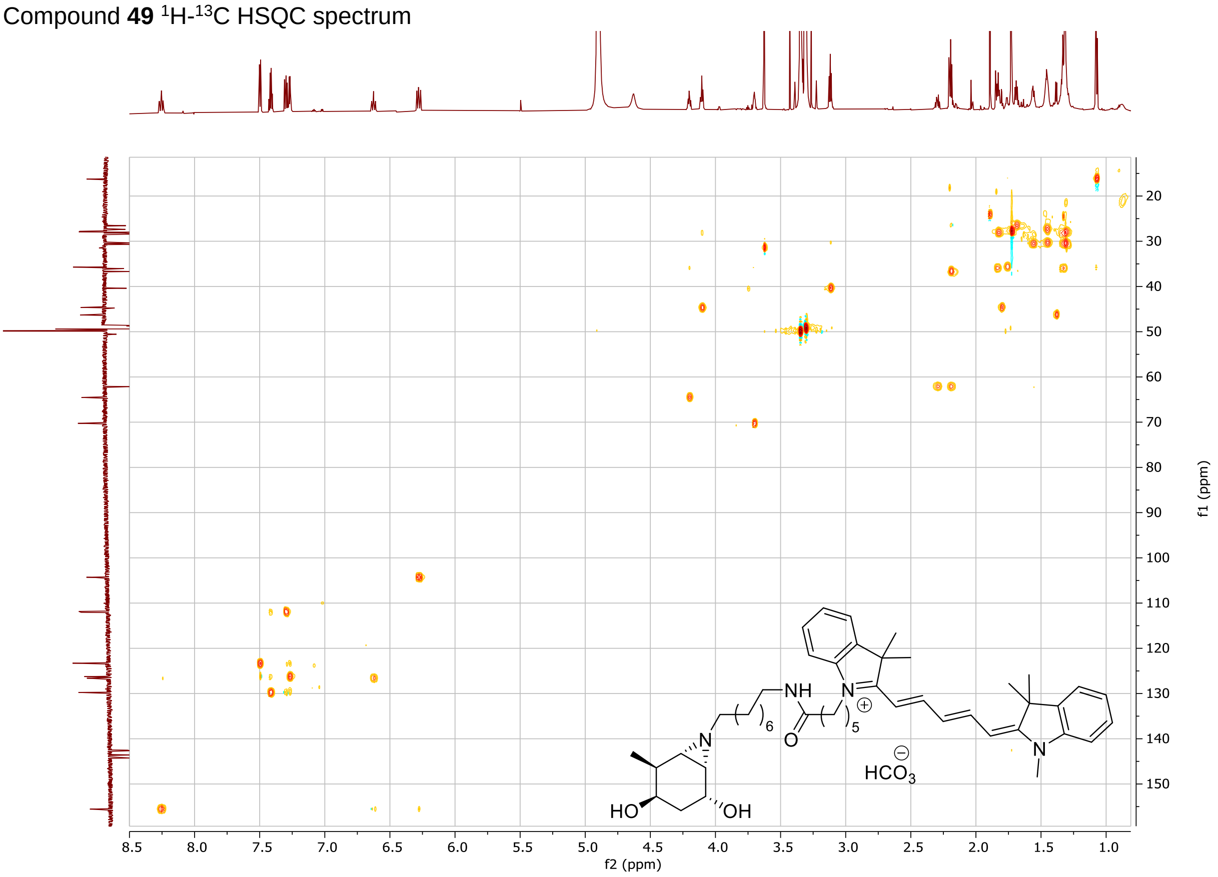

Compound **56** ^{1}H NMR spectrum

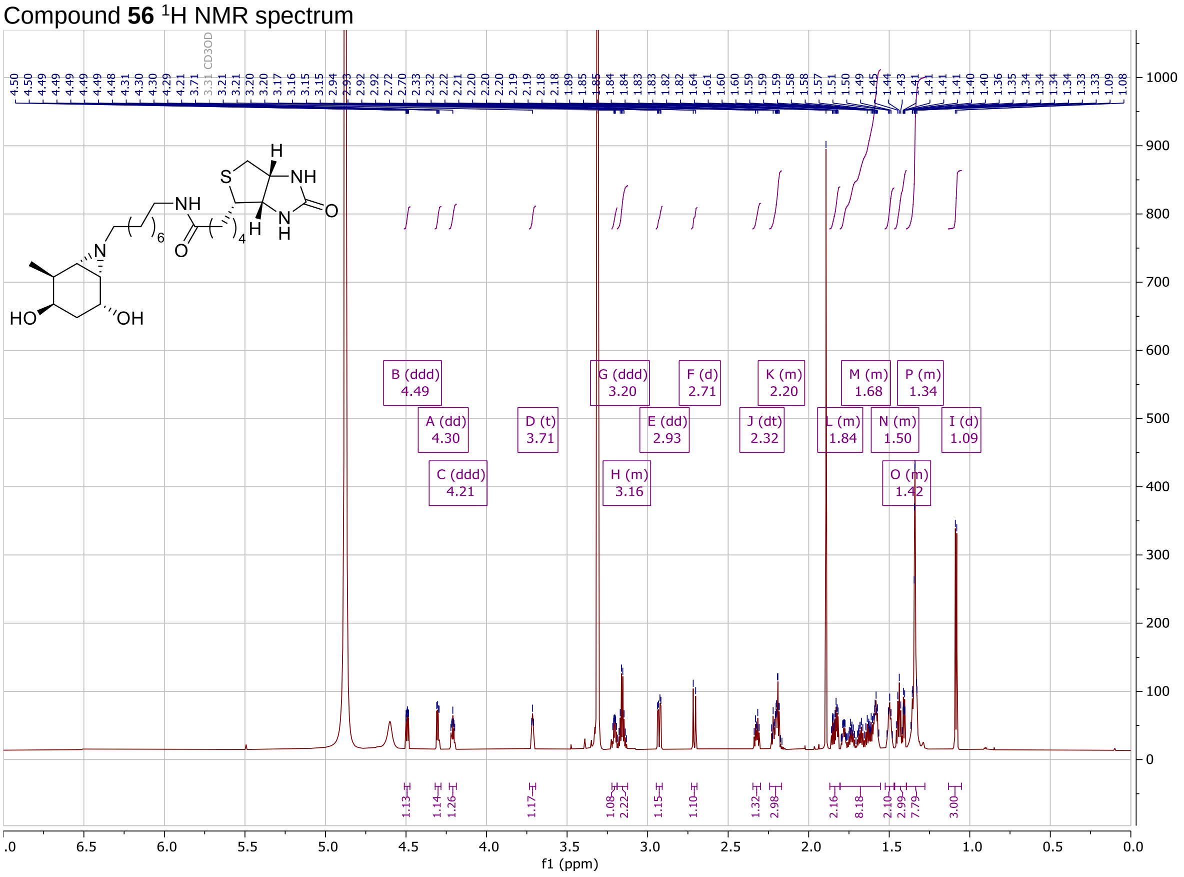

Compound **56** ^{13}C NMR APT spectrum

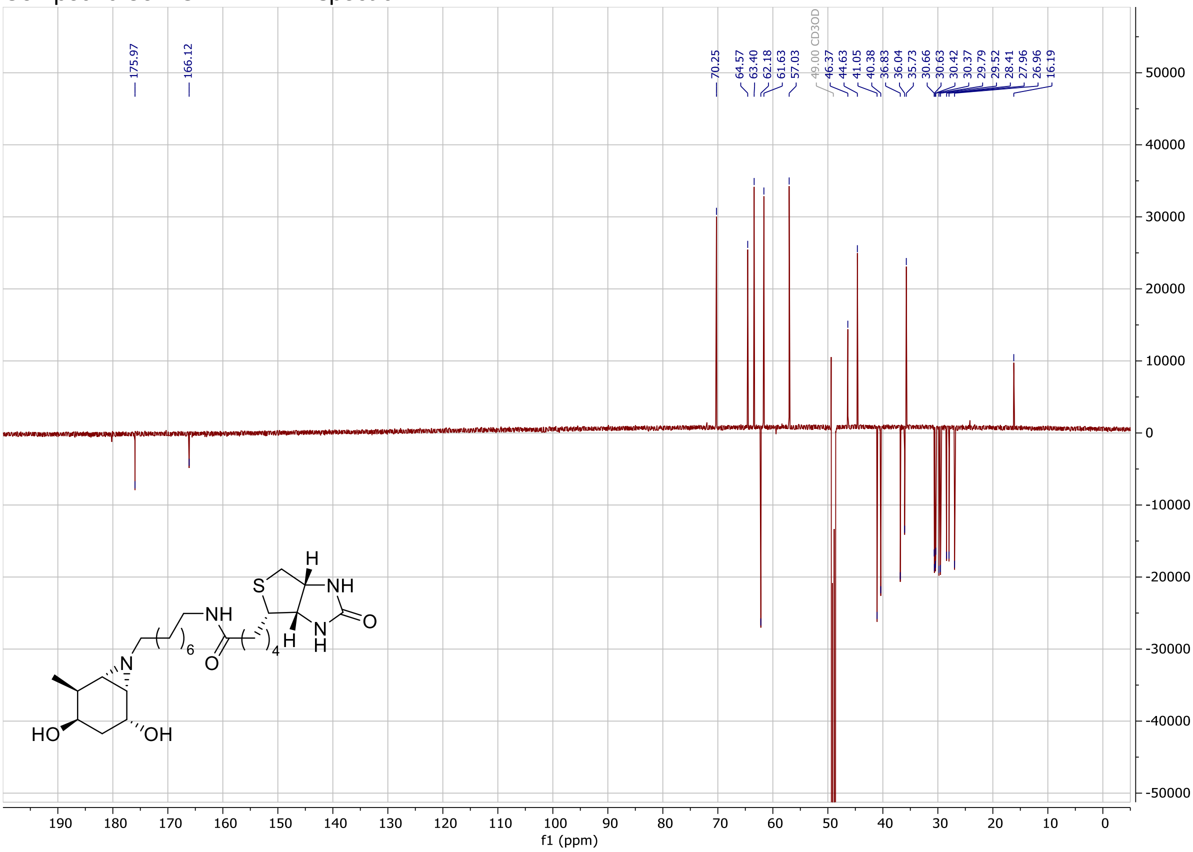

Compound **56** ^{1}H-^{1}H COSY spectrum

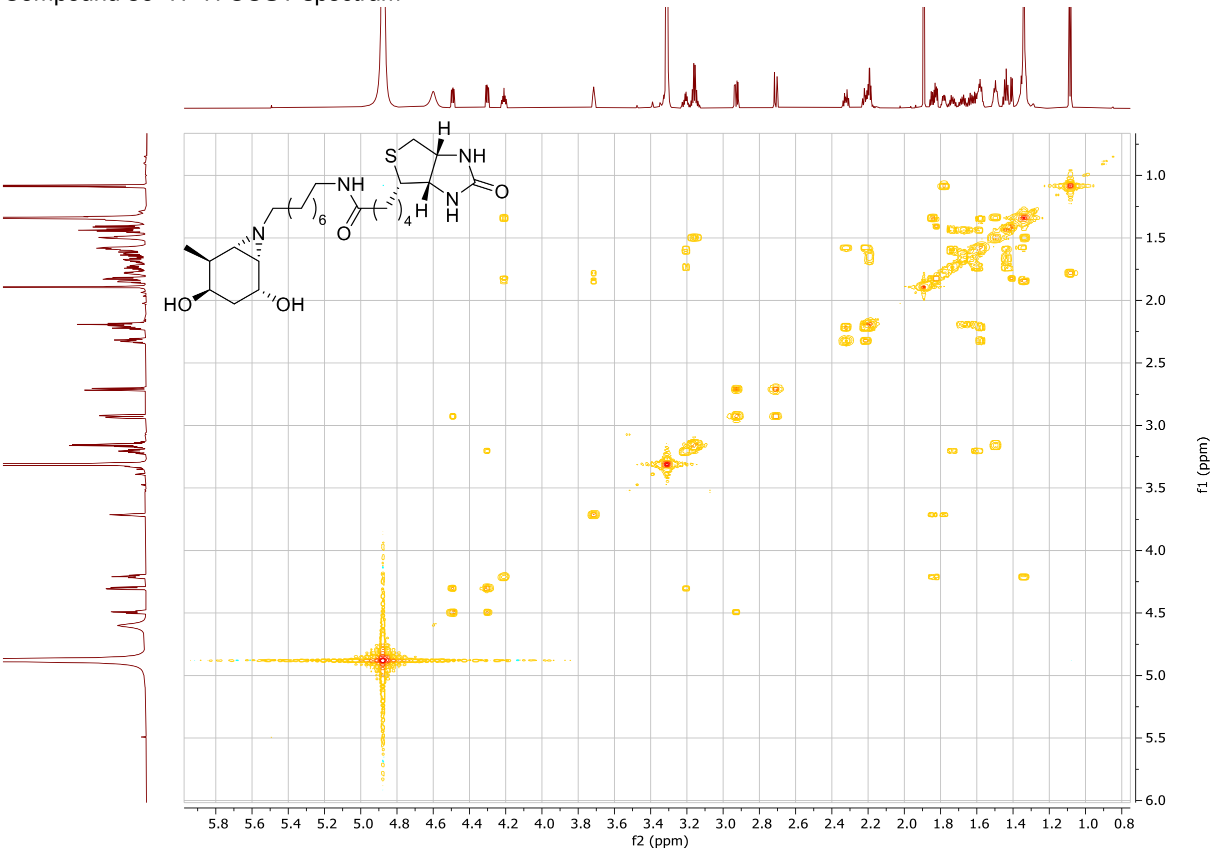

Compound **56** ^{1}H-^{13}C HSQC spectrum

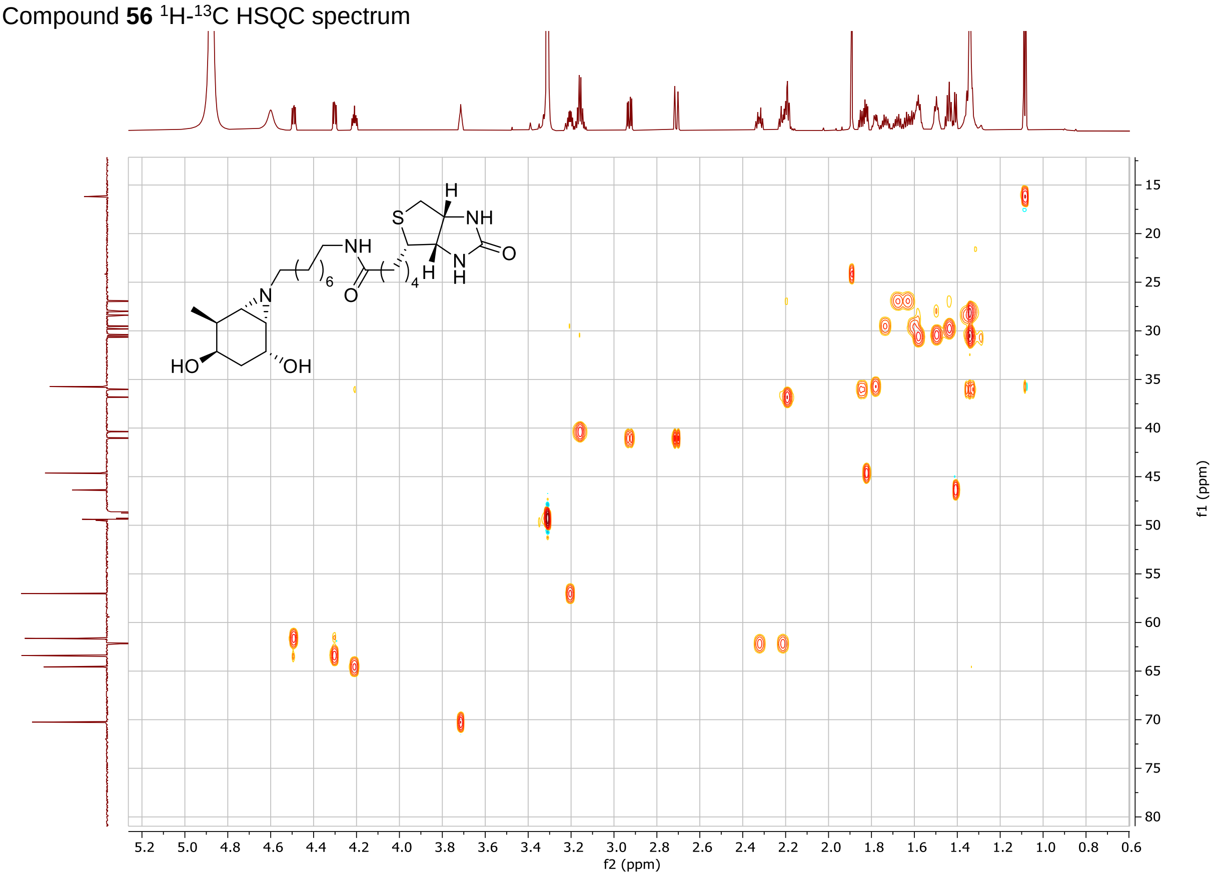

Supplement: Supplementary file 1 — Supporting Information [file CHEM-30-e202402988-s001.pdf]
